# Iterative Dual-Metal and Energy Transfer Catalysis Enables Stereodivergence in Alkyne Difunctionalization: Carboboration as Case Study

**DOI:** 10.1021/acscatal.3c03570

**Published:** 2023-11-03

**Authors:** Javier Corpas, Miguel Gomez-Mendoza, Enrique M. Arpa, Víctor A. de la Peña O'Shea, Bo Durbeej, Juan C. Carretero, Pablo Mauleón, Ramón Gómez Arrayás

**Affiliations:** † Department of Organic Chemistry, Faculty of Science; Institute for Advanced Research in Chemical Sciences (IAdChem); and Centro de Innovación en Química Avanzada (ORFEO−CINQA), Universidad Autónoma de Madrid (UAM), Cantoblanco, 28049 Madrid, Spain; ‡ Photoactivated Processes Unit, IMDEA Energy Institute, Technological Park of Mostoles, Avda. Ramón de la Sagra 3, 28935 Madrid, Spain; § Division of Theoretical Chemistry, IFM, Linköping University, 581 83 Linköping, Sweden

**Keywords:** cooperative catalysis, energy transfer catalysis, stereodivergence, tetrasubstituted olefins, alkenyl boronates, β-boryl acrylates, photoisomerization

## Abstract

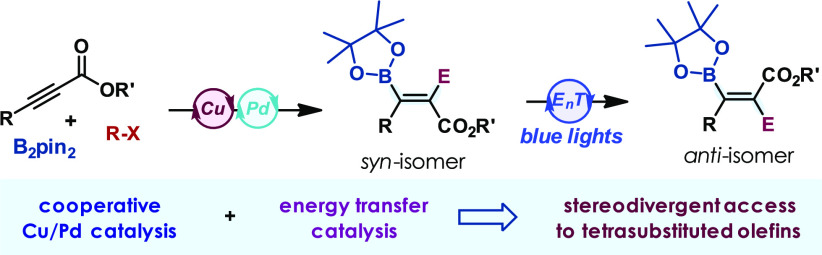

Stereochemically defined tetrasubstituted olefins are widespread structural elements of organic molecules and key intermediates in organic synthesis. However, flexible methods enabling stereodivergent access to *E* and *Z* isomers of fully substituted alkenes from a common precursor represent a significant challenge and are actively sought after in catalysis, especially those amenable to complex multifunctional molecules. Herein, we demonstrate that iterative dual-metal and energy transfer catalysis constitutes a unique platform for achieving stereodivergence in the difunctionalization of internal alkynes. The utility of this approach is showcased by the stereodivergent synthesis of both stereoisomers of tetrasubstituted β-boryl acrylates from internal alkynoates with excellent stereocontrol via sequential carboboration and photoisomerization. The reluctance of electron-deficient internal alkynes to undergo catalytic carboboration has been overcome through cooperative Cu/Pd-catalysis, whereas an Ir complex was identified as a versatile sensitizer that is able to photoisomerize the resulting sterically crowded alkenes. Mechanistic studies by means of quantum-chemical calculations, quenching experiments, and transient absorption spectroscopy have been applied to unveil the mechanism of both steps.

Tetrasubstituted olefins are pivotal building units of biologically active, and functional materials and strategically important for the rapid assembly of chiral (3D) molecules upon selective functionalization of the C–C double bond (Figure 1).^1,2^ However, the development of selective methods for their preparation remains a considerable challenge. Traditional methods for olefin preparation (e.g., Wittig, Peterson, Julia) are inefficient for this class of substrates because the high steric crowding around the C=C generates energetically demanding transition states and deviation of the olefinic core from planarity.^3^ Even more difficult is the development of flexible methods enabling stereodivergent access to densely functionalized tetrasubstituted olefins from the same precursor.^4^ Indeed, the design of stereodivergent methods for the synthesis of tetrasubstituted olefins is very appealing because they can provide orthogonal exit vectors to explore new chemical space, which may be exploited for streamlining diversity-oriented library development.^5^

**Figure 1:**
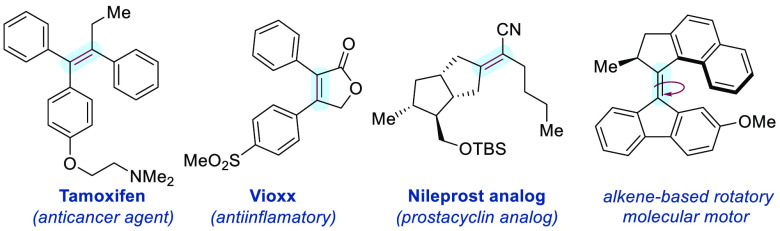
Examples of functional tetrasubstitued alkenes.

The transition-metal-catalyzed difunctionalization of internal alkynes represents an archetypal approach for the assembly of tetrasubstituted olefins.^6^ However, the inherent *syn*-stereochemical outcome of the *syn* insertion across the alkyne of the organometallic species renders this approach unfit for accessing the unconventional *anti*-addition stereochemistry (Scheme 1A).^6a,7^ Therefore, the preparation of both *E* and *Z* isomers often requires using different synthetic routes and two different sets of precursors. The selective photoisomerization of alkenes, which exploits excited-state reactivity via visible-light-mediated energy transfer (E_n_T) catalysis, has recently emerged as a potential solution to this fundamental limitation.^8^ A key approach to gain directionality in the isomerization is the disruption of conjugation of one isomer after triplet sensitization caused by noncovalent interactions, either destabilizing (e.g., A^1,3^ strain in styrenes)^9^ or stabilizing,^10^ thus resulting in a negligible re-excitation of the final product that accumulates in the reaction mixture. However, this approach to geometry control remains largely limited to di- and trisubstituted olefins, while its extension to tetrasubstituted ones is much more challenging because of steric interactions in the ground state (Scheme 1A). Recently, Gilmour et al. devised an elegant photoisomerization of trisubstituted β-boryl acrylates under E_n_T catalysis that takes advantage of an attractive n_O_ → p_B_ interaction between the CO group and B to break conjugation (Scheme 1B).^11^ During the preparation of this manuscript, Gilmour et al. reported the photoisomerization of tetrasubstituted α-fluoro β-borylacrylic acid derivatives.^12^ Nevertheless, steric constraints still present a major hurdle in alkene photoisomerization, and its application to tetrasubstituted alkenes still remains a highly coveted milestone.^10,11^

**Scheme 1:**
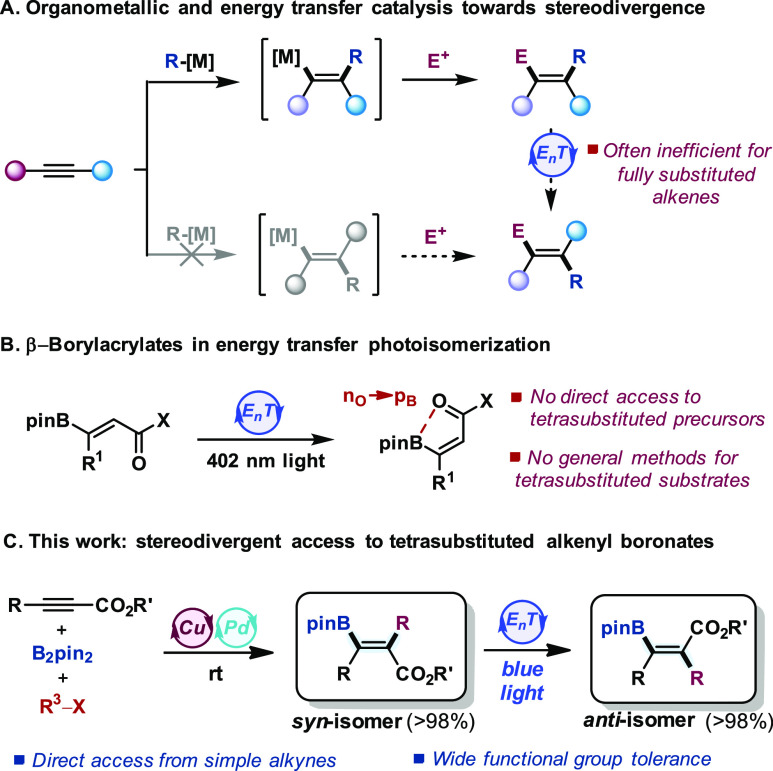
Approaches to Stereodivergence toward the Synthesis of Tetrasubstituted Olefins: (a) Merging Organometallic and Energy Transfer Catalysis, (b) Boron-Enabled Photoisomerization by Energy Transfer Catalysis, and (c) Iterative Dual-Metal and Energy Transfer Catalysis for β-Boryl Acrylates (This Work)

The exceptional versatility of the C–B bond in stereospecific cross-couplings,^13,14^ the rich chemistry of the carbonyl group, and the unique umpolung of the α,β-unsaturated carbonyl system^15^ harnessing the possibility to incorporate electrophiles into the β-position of the enone fragment after elaboration of the C–B bond render tetrasubstituted β-boryl acrylates ideal synthons en route to fully substituted functionalized olefins.^16^ However, their preparation is challenging and typically requires indirect multistep approaches, such as the Miyaura borylation from preformed stereodefined alkenyl halides.^17−20^ The Cu-catalyzed B_2_pin_2_-carboboration of internal alkynes has emerged as one of the most efficient means of generating tetrasubstituted alkenyl boronic esters,^21−23^ but its extension to electron-deficient alkynoates remains largely elusive with no reports to date.^23,24^ This lack of success is likely a consequence of the presence of the CO_2_R group, which on the one hand reduces the already intrinsically poor nucleophilicity of the β-boryl alkenyl–Cu intermediate, thereby preventing its reaction with electrophiles,^25^ and on the other hand may promote *E/Z* isomerization of the alkenyl–Cu complex intermediate via Cu–allenolate species, thus compromising the stereoselectivity.^26^

We sought to develop an iterative catalytic alkyne difunctionalization/E_n_T photoisomerization as a general platform for achieving stereodivergence in the assembly of tetrasubstituted alkenes from the same alkyne precursor, thus circumventing the current need to prepare both isomers by different routes. These expectations were borne out in practice through the sequential catalytic carboboration and E_n_T photoisomerization that provides stereodivergent access to tetrasubstituted β-boryl acrylates from internal alkynoates (Scheme 1C). The synergistic combination of Cu and Pd catalysis^27,28^ facilitates the otherwise unfeasible carboboration of the electron-deficient alkyne via transmetalation of the alkenyl–Cu intermediate with organo-Pd^II^ species. An Ir complex was identified as an efficient sensitizer for the selective photoisomerization of the resulting tetrasubstituted alkenyl boronic esters under blue light irradiation (typically >98% selectivity). The global method combines broad substrate scope and applicability to complex multifunctional drug-like molecules. Transient absorption spectroscopy and quantum chemical calculations provide mechanistic insights for both steps.

## Results and Discussion

### 1 Optimization Studies

#### 1.1 Carboboration

Alkynoate **1a** was subjected to standard conditions for Cu-catalyzed carboboration using B_2_pin_2_ and MeI as electrophile, in combination with CuCl (10 mol %), PCy_3_ as a strong σ-donor ligand (10 mol %), and NaO*t*Bu (1.5 equiv) as base in THF (Table 1).^21,24j^ Although some carboboration (CB) product (*Z*)-**3a** was formed, the reaction gave mainly the hydroboration (HB) product (*Z*)-**6a** (CB/HB = 19:81) with low conversion (32% mixture yield, entry 1). Larger quantities of electrophile or base did not result in significant improvements (see the Supporting Information). This is consistent with the low nucleophilicity of the alkenyl–Cu(I) intermediate. However, a simple addition of 5 mol % PdCl_2_(PPh_3_)_2_ to the reaction media led to a dramatic increase in reactivity and CB-selectivity, which afforded the CB product *syn*-(*Z*)-**3a** in 84% yield (entry 2). This is likely because of the engagement of Pd through transmetalation between alkenyl–Cu(I) and methyl–Pd(II) catalytic intermediates. Other Pd complexes, such as the electron-rich [IPrPdCl_2_]_2_ (69%, entry 3) or the Buchwald precatalyst PCy_3_·Pd·G3 (57%, entry 4), led to slightly lower yields (see the Supporting Information for full studies). The use of Pd(OAc)_2_ in combination with additional PCy_3_ (10 mol %) led to the CB product (*Z*)-**3a** in 91% yield (entry 5), which was established as optimized conditions.

**Table 1:**
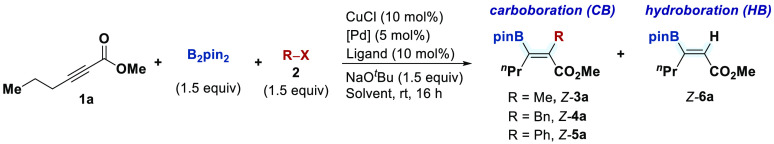
Optimization Studies for the *syn*-Carboboration of Alkyne **1a**

entry	electrophile	[Pd]	ligand	solvent	CB/HB^a^	yield (%)^a^
1	MeI (**2a**)		PCy_3_	THF	19:81	(*Z*)-**3a** + (*Z*)-**6a**, 32
2	MeI (**2a**)	PdCl_2_(PPh_3_)_2_	PCy_3_	THF	>98:2	(*Z*)-**3a**, 84
3	MeI (**2a**)	[^*i*^PrPdCl_2_]_2_	PCy_3_	THF	>98:2	(*Z*)-**3a**, 69
4	MeI (**2a**)	PCy_3_·Pd·G3	PCy_3_	THF	>98:2	(*Z*)-**3a**, 57
5	MeI (**2a**)	Pd(OAc)_2_ + PCy_3_ (10 mol %)	PCy_3_	THF	>98:2	(*Z*)-**3a**, 91
6	BnBr (**2b**)	Pd(OAc)_2_ + PCy_3_ (10 mol %)	PCy_3_	THF	>98:2	(*Z*)-**4a**, 47
7	BnBr (**2b**)	PdCl_2_(PPh_3_)_2_	PCy_3_	THF	>98:2	(*Z*)-**4a**, 62
8	BnBr (**2b**)	PdCl_2_(PPh_3_)_2_	BINAP	THF	>98:2	(*Z*)-**4a**, 45
9	BnBr (**2b**)	PdCl_2_(PPh_3_)_2_	XantPhos	THF	>98:2	(*Z*)-**4a**, 94
10	BnBr (**2b**)	PdCl_2_(PPh_3_)_2_	dppbz	THF	>98:2	(*Z*)-**4a**, 25
11	PhI (**2c**)	Pd(OAc)_2_ + PCy_3_ (10 mol %)	PCy_3_	THF	>98:2	(*Z*)-**5a**, 12
12	PhI (**2c**)	PdCl_2_(PPh_3_)_2_	XantPhos	THF	>98:2	(*Z*)-**5a**, 26
13	PhI (**2c**)	PdCl_2_(PPh_3_)_2_	XantPhos	toluene	>98:2	(*Z*)-**5a**, 54
14	PhI (**2c**)	Pd_2_(dba)_3_·CHCl_3_	XantPhos	toluene	>98:2	(*Z*)-**5a**, 96
15	PhI (**2c**)	PdCl_2_(dppf)	XantPhos	toluene	>98:2	(*Z*)-**5a**, 72
16^b^	PhI (**2c**)	Pd_2_(dba)_3_·CHCl_3_	XantPhos	toluene	>98:2	(*Z*)-**5a**, 21
17	PhBr (**2c′**)	Pd_2_(dba)_3_·CHCl_3_	XantPhos	toluene	>98:2	(*Z*)-**5a**, 56

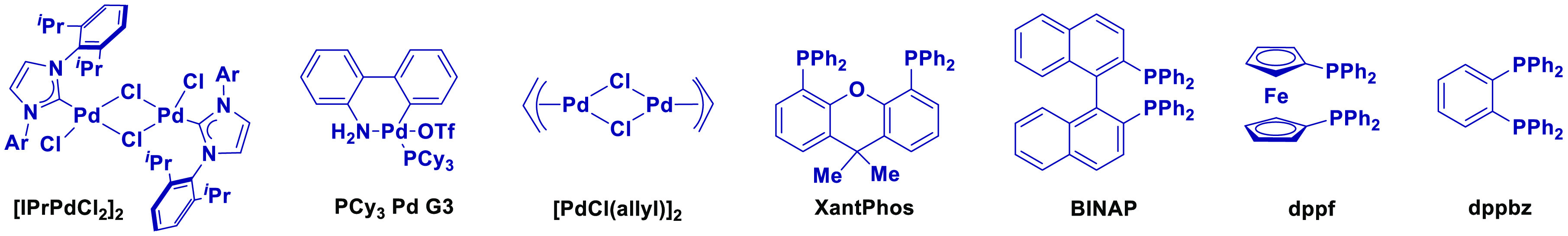

a Determined in the reaction crude by ^1^H NMR spectroscopy using 1,3,5-trimethoxybenzene as internal standard.

b Used 20 mol % XantPhos. In all cases *Z/E* and regioisomeric ratio (*rr*) values were >98:2, as determined by ^1^H NMR spectroscopy of the reaction crude.

The application of this protocol to benzyl bromides was tested in the reaction with **1a** with benzyl bromide, which afforded the CB product (*Z*)-**4a** with complete *syn* stereoselectivity but a low yield (47%, entry 6). This result revealed a significant dependence of the catalyst over the electrophile employed. However, the reactivity was fully restored by simply adjusting the Pd source to PdCl_2_(PPh_3_)_2_ (entries 7 and 8) and the ligand (XantPhos), which afforded (*Z*)-**4a** in 94% yield (entry 9). Other bisphosphine ligands were less efficient (dppbz, 25%, entry 10).

Then, we sought to expand the scope to iodoarenes (Table 1, entries 11–17). The reaction of **1a** with iodobenzene under the optimized conditions for methylboration led to only 12% conversion of the expected product (*Z*)-**5a** (entry 11). The yield was increased to 26% using PdCl_2_(PPh_3_)_2_ in combination with CuCl/XantPhos (entry 12). A brief solvent screening using the combination of PdCl_2_(PPh_3_)_2_ and CuCl/XantPhos, quickly identified toluene as the optimal reaction media [54% yield of (*Z*)-**5a**, entry 13]. Hence, we revisited the Pd screening using toluene as solvent, with Pd_2_(dba)_3_·CHCl_3_ providing the best results [96% yield of (*Z*)-**5a**, entry 14],^28c^ whereas other complexes, such as PdCl_2_(dppf), provided lower efficiency (72%, entry 15). Higher loadings of XantPhos (20 mol %) proved detrimental to reactivity [21% of (*Z*)-**5a**, entry 16], which points out the importance of the metal/ligand speciation for productive catalysis. The use of bromobenzene led to a remarkable drop in the reactivity (56%). Remarkably, when the Pd system was absent when using benzyl bromide or phenyl iodide, only traces of the hydroboration byproduct (*Z*)-**6a** were detected (see the Supporting Information for details). Overall, these results evidence the high flexibility of this catalyst system because it can be easily fine-tuned to achieve high reactivity with diverse reagents.

#### 1.2 Photoisomerization

Having found optimal conditions for the assembly of tetrasubstituted olefins with *syn* stereochemistry in the carboboration reaction, we turned our attention to accessing the opposite stereochemistry. Initially, we tested the model substrate (*Z*)-**3a** in the presence of different photosensitizers (Table 2). When Ru(bpy)_3_Cl_2_·6H_2_O (**PC-1**) was used in THF under either blue or green light irradiation, no isomerization was observed after 24 h (entry 1). Considering the low triplet energy of this catalyst (*E*_T_ = 46 kcal·mol^–1^),^29^ we switched to commercially available Ir-based photosensitizers, which typically display higher triplet energies in the excited state. We observed that the use of photocatalysts with triplet energies below 58 kcal·mol^–1^ {[Ir(^t^Buppy)_2_(dtbbpy)]PF_6_ (**PC-2**), [Ir(ppy)_2_(dtbbpy)]PF_6_ (**PC-3**), and *fac*-Ir(ppy)_3_ (**PC-4**), entries 2–4, respectively)}^30−32^ did not yield the desired *E* isomer. Ir sensitizers with slightly higher triplet energies, such as Ir(Fppy)_3_ (**PC-5**) and Ir(dFppy)_3_ (**PC-6**), showed partial isomerization.^4a^ However, the use of [Ir(dFCF_3_ppy)_2_(bpy)]PF_6_ (**PC-7**) (*E*_T_ = 62 kcal·mol^–1^)^33^ led to full conversion to (*E*)-**2a** (entry 5). We then studied the application of these conditions to substrates bearing different substituents, such as benzyl [(*Z*)-**4a**] and phenyl [(*Z*)-**5a**], which photoisomerized using **PC-7** under blue light irradiation to yield (*E*)-**4a** and (*E*)-**5a**, respectively, with complete conversion and excellent stereoselectivity (*E/Z* > 98%). Notably, the obtention of the latter supports the notion that this isomerization is driven by a n_O_ → p_B_ noncovalent interaction in the *E*-isomer instead of A^1,3^ strain, since *E/Z* mixtures would be obtained in this case.^8,9^

**Table 2:**
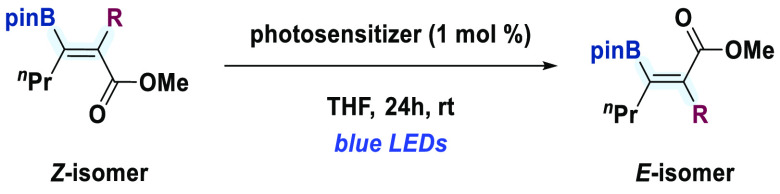
Optimization Studies for *Z*-to-*E* Photoisomerization

entry	photosensitizer (*E*_T_, kcal·mol^–1^)	*E* isomer, R	*E*/*Z*^a^
1	**PC-1** (46)	**3a**, Me	<2:98
2	**PC-2** (50.7)	**3a**, Me	<2:98
3	**PC-3** (51)	**3a**, Me	<2:98
4	**PC-4** (55.2)	**3a**, Me	<2:98
5	**PC-5** (58.6)	**3a**, Me	30:70
6	**PC**-**6** (60.1)	**3a**, Me	45:55
7	**PC-7** (62)	**3a**, Me	>98:2
8	**PC-7** (62)	**4a**, Bn	>98:2
9	**PC-7** (62)	**5a**, Ph	>98:2

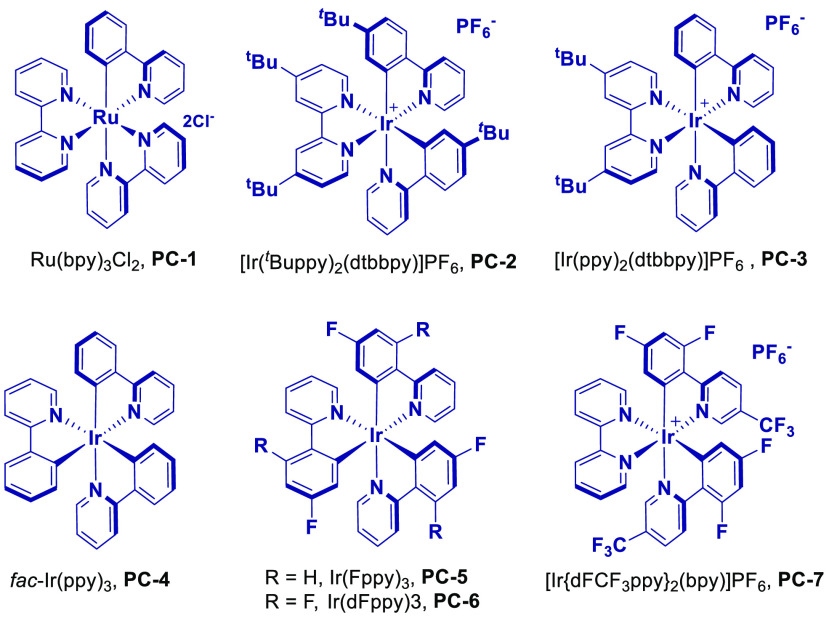

a Determined in the reaction crude by 1H NMR spectroscopy using 1,3,5-trimethoxybenzene as internal standard.

### 2 General scope

#### 2.1 Carboboration

**Scheme 2:**
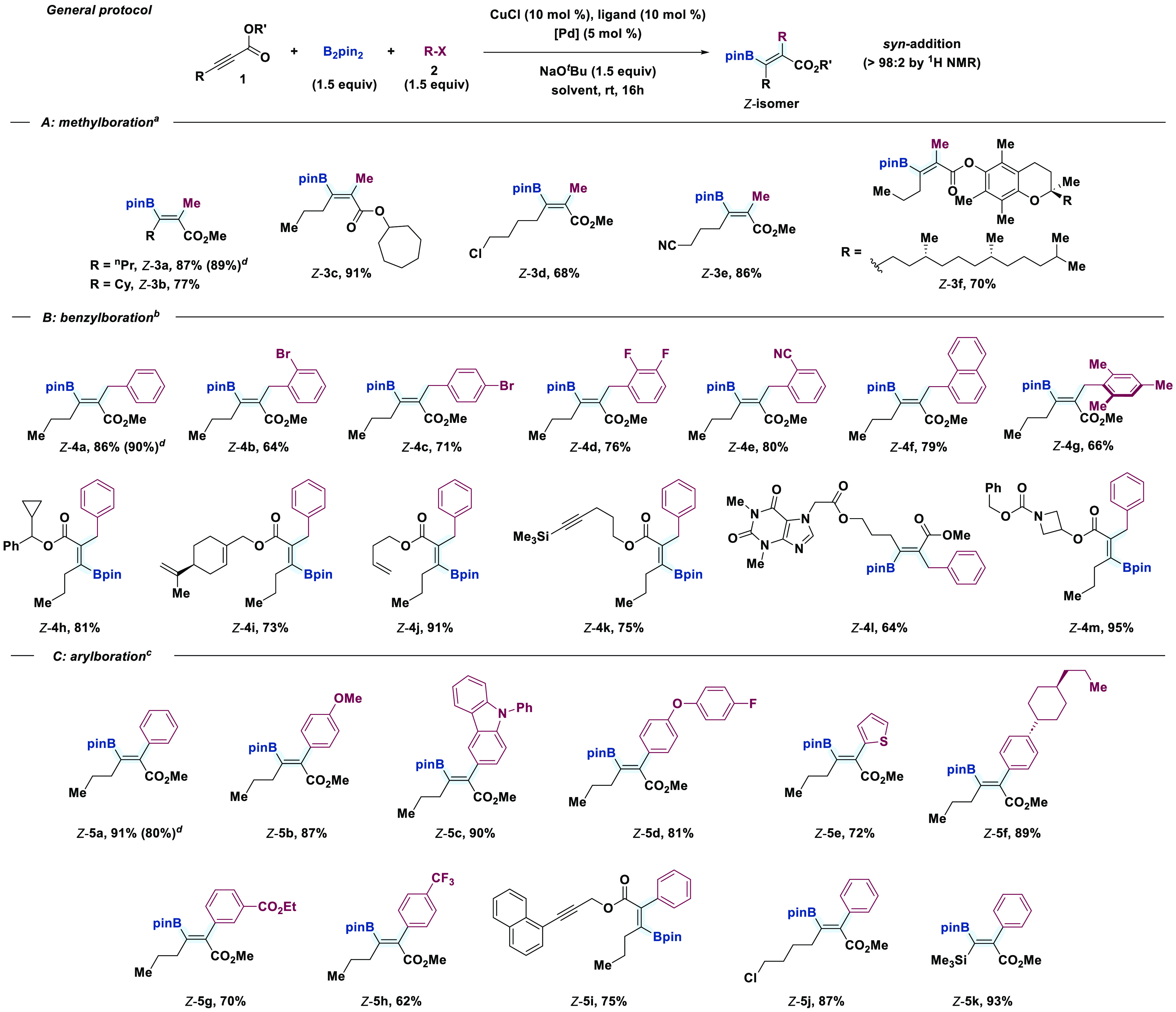
Substrate Scope for the *syn*-Carboboration of Internal Alkynoates: (a) Examples for Methylboration, (b) Examples for Benzylboration, and (c) Examples for Arylboration Ligand = PCy_3_, [Pd] = Pd(OAc)_2_ + PCy_3_ (10 mol %), solvent = THF. Ligand = XantPhos, [Pd] = PdCl_2_(PPh_3_)_2_, solvent = THF. Ligand = XantPhos, [Pd] = Pd_2_(dba)_3_·CHCl_3_, solvent = toluene. Reaction performed at 2 mmol scale. All reactions were conducted on a 0.1 mmol scale unless otherwise noted. The *syn* stereoselectivity was determined by ^1^H NMR spectroscopy of the reaction crude. Reaction yields measured after purification by flash column chromatography.

The results of an examination of the scope of the B_2_pin_2_-carboboration of various alkynes are presented in Scheme 2, which show complete regio- and *syn* stereoselectivity for all the substrates examined. Initially, we focused our attention to the methylboration protocol owing to the privileged presence of methyl groups in many drug candidates due to its ability to modulate physicochemical properties of pharmaceuticals.^34^ Generally, uniformly good yields were observed for several alkynes (Scheme 2A), including those bearing α-branched alkyl substituents at either the carbon triple bond or the ester group [(*Z*)-**3b**, 77% and (*Z*)-**3c**, 91%]. The method is tolerant of potentially sensitive groups in the presence of Pd, such as alkyl chlorides [(*Z*)-**3d**, 68%], and base-sensitive aliphatic nitriles [(*Z*)-**3e**, 86%]. Alkynes embedded in complex chiral molecules, such as the α-tocopherol derivative, underwent methylboration in good yield with complete preservation of the stereochemical integrity [(*Z*)-**3f**, 70%].

The scope of the benzylboration revealed wide tolerance to substitution at the aromatic ring, regardless of electronic properties or position, thereby leading to the carboboration products from good to excellent yields (62–91%, Scheme 2B). The chemoselectivity was nicely illustrated by the fact that sensitive functional groups, such as aryl bromides [(*Z*)-**4b** and (*Z*)-**4c**, 64% and 71%], electron-poor fluorinated scaffolds [(*Z*)-**4d**, 76%], and aryl nitriles [(*Z*)-**4e**, 80%], were well tolerated. Extended π-systems and sterically hindered benzylic derivatives were also well accommodated without erosion of the regio- and stereoselectivity [(*Z*)-**4f** and (*Z*)-**4g**, 79% and 66%, respectively]. Regarding the substitution at the alkyne partner, cyclopropyl ring [(*Z*)-**4h**, 81%], alkenes [(*Z*)-**4i**, 73%, and (*Z*)-**4j**, 91%], and alkynes [(*Z*)-**4k**, 75%] were perfectly accommodated at either coupling partner. Crucially, in these latter cases, the borylation occurred exclusively at the most activated unsaturation system. Potentially coordinating substrates, such as xanthine [(*Z*)-**4l**, 64%], and strained azetidine [(*Z*)-**4m**, 95%] also demonstrated excellent functional group tolerance under the reaction conditions.

Subsequently, a range of *para*- and *meta*-substituted iodoarenes with diverse electronic properties (ethers, esters, CF_3_, and heteroaryl) were readily exploited in the aryl-boration [(*Z*)-**5a**–**h**, 62–91%, Scheme 2C]. Potentially competitive groups attached at the alkyne partner, such as a C–C triple bond [(*Z*)-**5i**, 75%], and chloride-containing alkyl pendant groups [(*Z*)-**5j**, 87%] were also compatible with the reaction conditions. Finally, we explored silyl–alkynes as a substrate, which led to the expected tetrasubstituted olefin with excellent yield [(*Z*)-**5k**, 93%] and provided a useful handle for orthogonal elaboration of the products. A 2 mmol scale carboboration of **1a** using MeI [(*Z*)-**3a**, 89%], BnBr [(*Z*)-**4a**, 90%], or PhI [(*Z*)-**5a**, 80%] demonstrated that the reaction can be scaled up with a similar efficiency (see Scheme 2). Unfortunately, other alkyl and allyl electrophiles could not be used in this protocol (see the Supporting Information for details).

#### 2.2 Photoisomerization

**Scheme 3:**
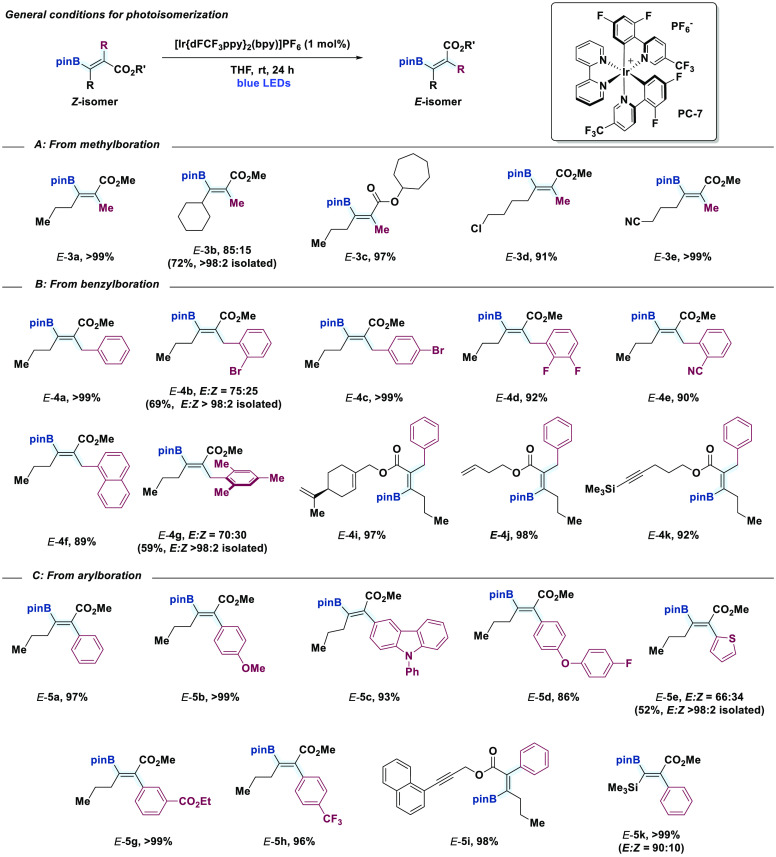
Substrate Scope for the Ir-Catalyzed Photoisomerization of Tetrasubstituted Alkenyl Boronic Esters: (a) Examples for Methylboration, (b) Examples for Benzylboration, and (c) Examples for Arylboration Stereoselectivity determined by ^1^H NMR spectroscopy of the reaction crude. Reaction yields after purification by flash column chromatography.

We next explored the efficiency of photoisomerization for a selected series of methyl, benzyl, and aryl alkenyl boronic esters. We initially tested alkenyl boron compounds substituted with a methyl group (Scheme 3A, **3a**–**3e**). First, (*E*)-**3a** was isolated as a pure stereoisomer (*E/Z* > 98:2) in quantitative yield after irradiation. The cyclohexyl-derived alkenyl boronic ester yielded the corresponding product with lower stereoselectivity (*E/Z* = 85:15), likely because of the strong deviation from planarity in (*Z*)-**3b** imposed by the cyclohexyl chain. However, stereochemically pure (*E*)-**3b** could be further isolated by flash chromatography (72% yield, *E/Z* > 98:2). Isomerization of a cycloheptyl alcohol ester afforded (*E*)-**3c** as a pure stereoisomer in excellent yield (97%). Finally, when Cl- and CN-containing (*Z*)-**3d** and (*Z*)-**3e** were submitted to the isomerization conditions, the corresponding *E* isomers were isolated with complete stereochemistry in 91% and quantitative yields, respectively.

Second, we explored selected benzylborylated products from Scheme 2 (Scheme 3B, **4a**–**k**). As previously mentioned, benzyl derivative (*E*)-**4a** was isolated as a spectroscopically pure isomer (*E/Z* > 98:2) in quantitative yield under the optimized reaction conditions. Structural effects were observed during the photoisomerization of (*Z*)-**4b**, which resulted in a mixture of both isomers in an *E/Z* = 75:25 ratio in the photostationary state. Despite this, the desired (*E*)-**4b** product could be isolated as a pure stereoisomer in 69% yield.^18^ When the bromine atom was located at the *para* position, photoisomerization to (*E*)-**4c** took place in quantitative yield with complete stereoselectivity. Related substrates, such as a bis(fluorinated benzyl) derivative, afforded (*E*)-**4d** in 92% yield. Interestingly, when other groups were placed at the *ortho* position of the phenyl ring, the steric hindrance was well tolerated [(*E*)-**4e** and (*E*)-**4f**, 90% and 89%, respectively], with the formation of the *E* isomer in spectroscopically pure form (*E/Z* > 98:2). On the contrary, a more sterically hindered 2,4,6-trimethyl-benzyl-containing olefin underwent incomplete isomerization, albeit the (*Z*)-**4g** product could be isolated in its stereochemically pure form in 59% yield after purification. The reaction tolerated the presence of both internal and terminal alkenes, such as those contained in products (*E*)-**4i** and (*E*)-**4j** (97% and 98%, respectively), and internal alkynes, as in the case of product (*E*)-**4k** (92% yield).

Finally, tetrasubstituted olefins bearing an aryl unit were subjected to photoisomerization (Scheme 3C, **5a**–**k**). Notably, the reaction took place regardless of the electronic nature of the aryl substituent to afford the corresponding *E* isomer in high yields and complete stereoselectivity. Products bearing a phenyl group [(*E*)-**5a**, 97% yield], electron-rich aromatic rings [(*E*)-**5b**–**d**, 93% quantitative, and 87% yields, respectively), and electron-withdrawing substituents [(*E*)-**5g**,**h**, quantitative and 96% yield, respectively) were isolated as single stereoisomers. When we studied a 2-thiophene derivative, we observed a *E/Z* = 66:34 ratio in the photostationary state, although stereochemically pure (*E*)-**5e** was isolated in 52% yield. This result suggests a strong S–B interaction in the *Z* isomer, which is in competition with the O–B dative bond in the *E* isomer. Finally, the alkyne-containing (*E*)-**5i** product was obtained with excellent stereoselectivity, and the silyl-substituted internal alkene afforded (*E*)-**5k**, thereby showing a synthetic alternative for the stereodivergent access to 1,1-bismetalated tetrasubstituted alkenes.^35^

#### 2.3 Formal Stereodivergent Approach to Complexity

**Scheme 4:**
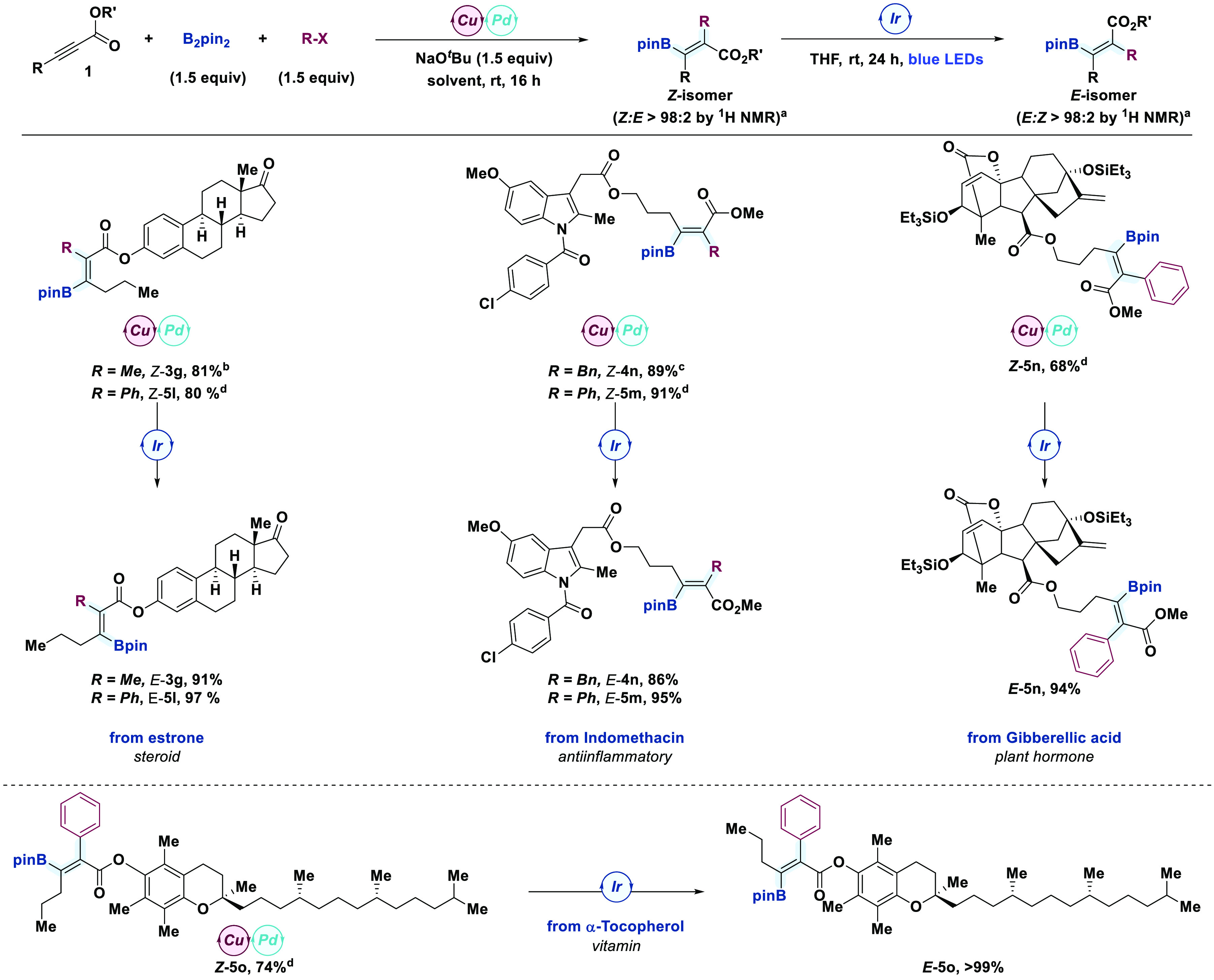
Application of the Formal Stereodivergent Carboboration to Complex Molecules The stereoselectivity was determined by ^1^H NMR spectroscopy of the reaction crude. Reaction yields were determined after purification by flash column chromatography. Ligand = PCy_3_, [Pd] = Pd(OAc)_2_ + PCy_3_ (10 mol %), solvent = THF. Ligand = XantPhos, [Pd] = PdCl_2_(PPh_3_)_2_, solvent = THF. Ligand = XantPhos, [Pd] = Pd_2_(dba)_3_·CHCl_3_, solvent = toluene

To further highlight the utility of this two-step strategy, we sought to prepare both stereoisomers of the same complex molecule. Ideally, these would come from alkynoates containing elaborate substituents with base-sensitive stereocenters and functional groups of diverse nature, thereby taking advantage of all the possible elements of structural diversity (Scheme 4). The assembly of both *E* and *Z* isomers of the tetrasubstituted alkenyl boronate contained in estrone derivatives **3g** and **5l** could be easily performed when MeI and BnBr were employed as the reactive electrophiles. Importantly, we observed complete functional tolerance under the reaction conditions since no side reactions from boryl-copper-promoted addition to the ketone or alkylation at the α-position through enolate formation were observed. Then, we studied derivatives from indomethacin, an anti-inflammatory drug, by using BnBr and PhI as model electrophiles (compounds **4n** and **5m**, respectively). The corresponding *Z* isomers of both products were obtained with complete *syn* stereoselectivity and high yields (89% and 91%, respectively). Equally efficient was the modification of the C–C double bond geometry of the tetrasubstituted olefin core using our optimized reaction conditions for the photoisomerization step, which led to the desired products (*E*)-**4n** and (*E*)-**5m** with complete *E* stereoselectivity and yields higher than 80%. The Gibberellic acid derivative (*Z*)-**5n** decorated with a pendant alkynoate motif was also obtained in 68% yield by means of the carboboration reaction using PhI as the electrophile. Complete stereoselectivity was obtained for the inversion of the stereochemistry at the tetrasubstituted alkene moiety after isomerization with **PC-7** under blue light, thereby enabling the isolation of (*E*)-**5n** in 94% yield. Importantly, all other stereocenters and functionalities were well tolerated. Finally, we explored the stereodivergent synthesis of the tetrasubstituted alkenyl boronate **5o** using a derivative of α-tocopherol, a form of vitamin E, and PhI as the electrophile, which led to the corresponding *Z* and *E* isomers in 74% and quantitative yield, respectively.

### 3 Mechanistic Studies

#### 3.1 Computational Studies

**Figure 2:**
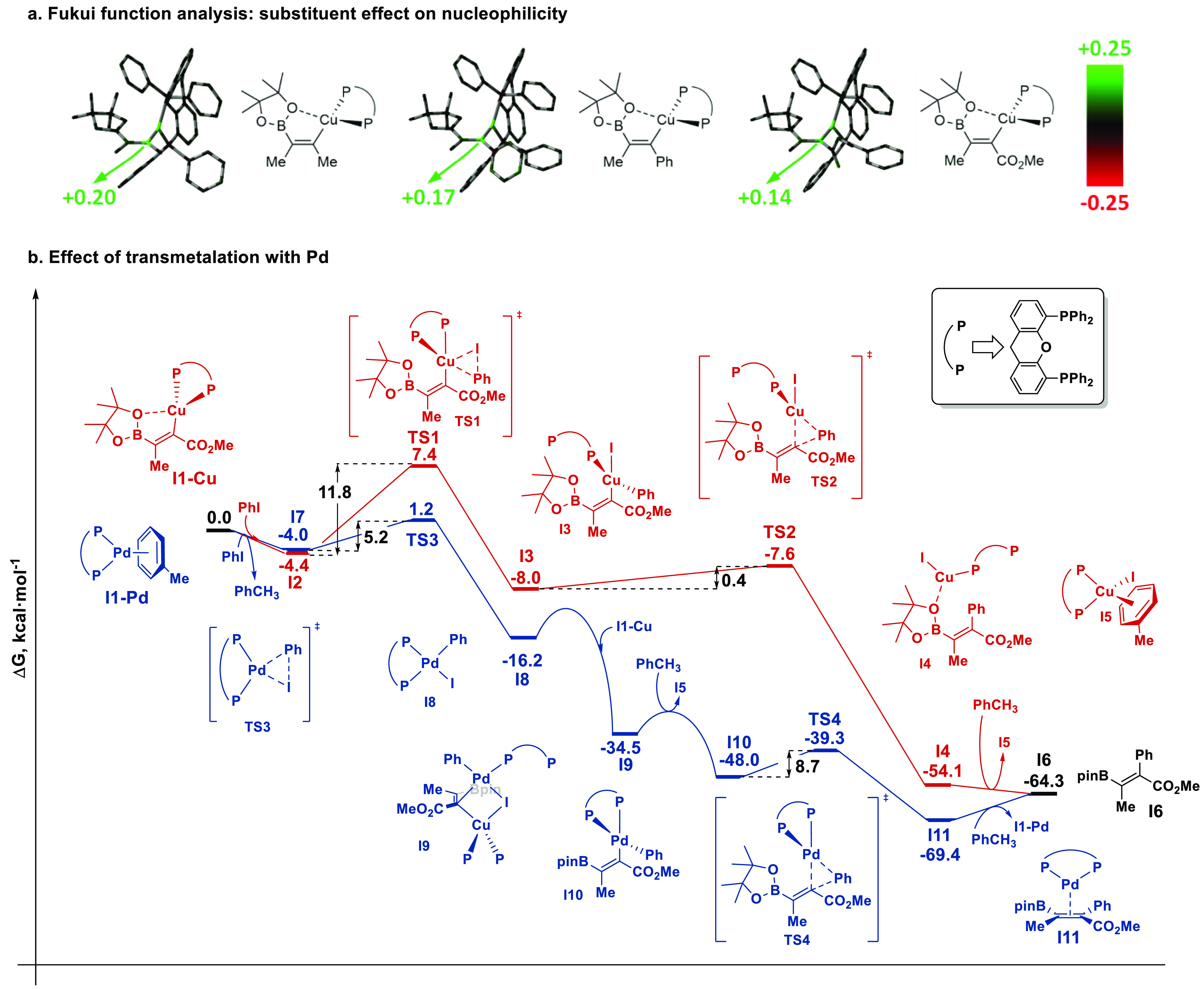
Computational studies on the carboboration reaction. (a) Nucleophilic condensed Fukui functions for alkenyl boronates bearing a methyl (left), phenyl (middle), and methyl ester (right) substituents at the α-position. Larger values are related to larger nucleophilicity (hydrogen atoms are omitted for clarity). (b) Reaction profiles for the Cu-catalyzed cross-coupling (red) and Cu/Pd-catalyzed cross-coupling (blue). Gibbs free energies at 298.15 K are given relative to the sum of all reagents (**I1–Cu** + **I1–Pd** + PhI + PhCH_3_) at infinite distance.

To further understand the mechanisms and observed selectivities of the *syn*-carboboration and photoisomerization reactions discussed in the preceding sections, we also performed quantum chemical calculations (see the Supporting Information for computational details). First, we sought to investigate to what extent the presence of the ester functionality next to the C(sp^2^)—Cu bond affects the nucleophilicity of the intermediate alkenyl–Cu(I). To this end, we compared the susceptibility for an electrophilic attack by calculating the corresponding nucleophilic Fukui functions (*f*_*_*_)^36^ for model substrates bearing methyl, phenyl and methyl ester substituents (Figure 2a). From this analysis, we observed that the nucleophilicity of the C—α position decreases from the methyl (+0.20) to the phenyl (+0.17) and methyl ester (+0.14) depending on the capacity to delocalize the negative charge at this position, which points to a less reactive intermediate with electrophiles in the latter case.

Then, we compared the reaction profiles of Cu-catalyzed carboboration (Figure 2b, red pathway) and the effect of transmetalation with palladium (Figure 2b, blue pathway). To reduce the computational cost, we made three simplifications: (a) we used phenyl iodide as the sole electrophile, (b) we replaced the propyl chain of **1a** with a methyl group, and (c) we removed the *gem*-dimethyl groups of XantPhos. None of these changes have a substantial impact on the reactivity, thus, still providing valid insight. First, we explored the Cu-catalyzed reaction. Coordination of PhI to the alkenyl–Cu(I) **I1–Cu** leads to the formation of a weakly bound van der Waals complex **I2**. Oxidative addition of PhI takes place to form **I3**, an intermediate Cu(III) species in which the XantPhos ligand adopts monodentate coordination. This is the rate-determining step of the reaction with an activation barrier of 11.8 kcal mol^–1^. Reductive elimination, followed by decoordination of the Cu catalyst, would lead to formation of the final adduct **I6**.

Next, we investigated the effect of Pd transmetalation from the intermediate alkenyl–Cu(I) **I1–Cu.** Starting from the Pd(0)/XantPhos complex (**I1–Pd**), the oxidative addition of PhI proceeds through a lower activation barrier of 5.3 kcal mol^–1^ because it is both kinetically and thermodynamically more favorable than the equivalent process with Cu. Transmetalation, which occurs via the formation of a bimetallic species upon reaction with **I1–Cu**, is a highly exergonic process in which the Cu center is displaced to form the alkenyl–Pd(II) intermediate **I10**. Then, reductive elimination and Pd detachment yield the alkenyl boronate **I6**.

From a comparison of the two pathways, the reactivity is enhanced upon the addition of palladium because of two key features: first, the oxidative addition step in which the large difference (6.5 kcal mol^–1^) between the activation barriers for Cu and Pd clearly promotes the reaction with the latter, and second, transmetalation, which is thermodynamically very favorable, thereby meaning that as soon as the alkenyl–Cu(I) **I1–Cu** is formed, it will be converted into the alkenyl–(II) intermediate **I10**. Therefore, with both **I1–Cu** and **I1–Pd** present, PhI will preferentially react with the latter and, once **I8** is formed, Cu–Pd transmetalation and reductive elimination have a clearly downhill energy profile.

**Figure 3:**
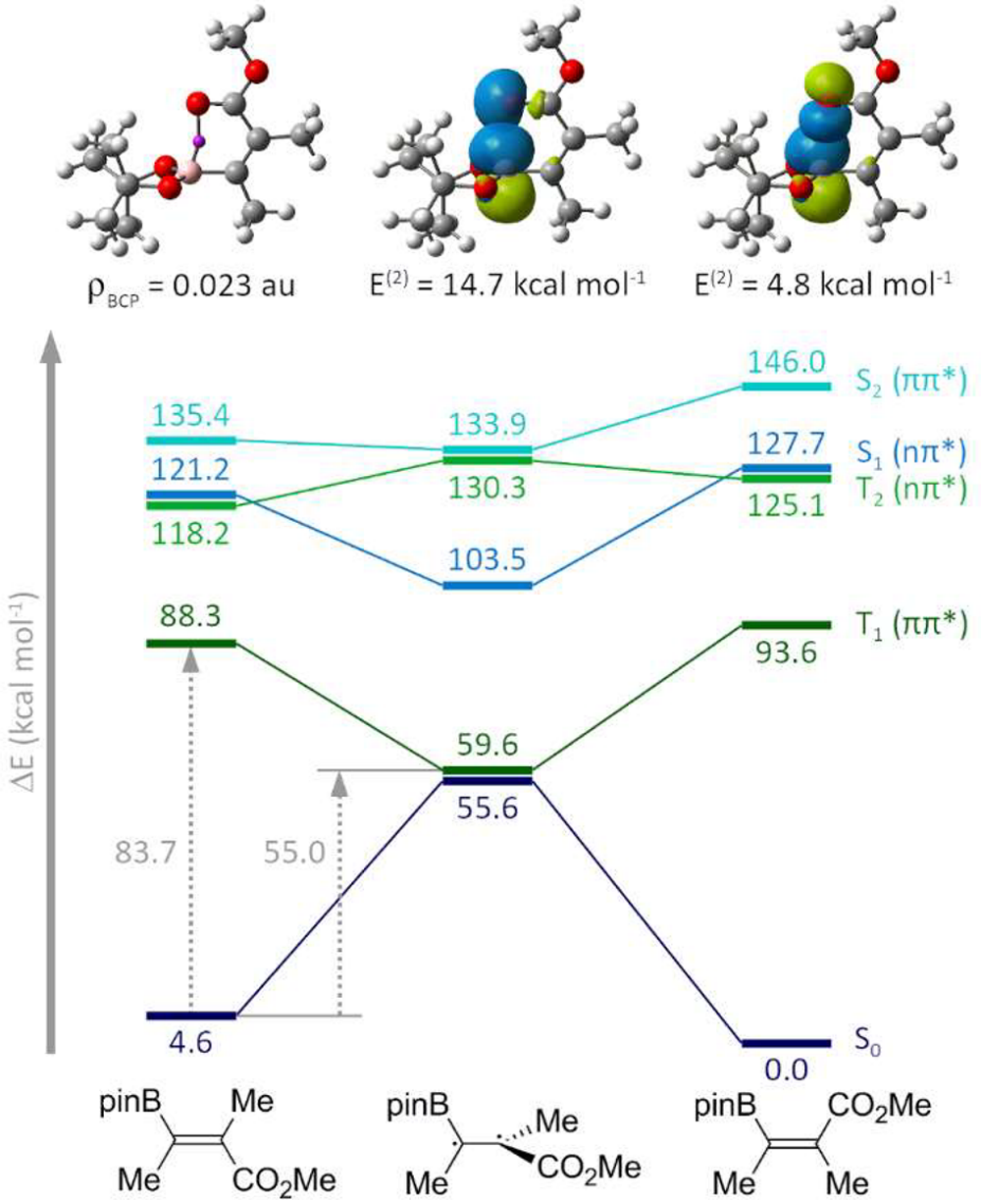
Computational studies on photoisomerization. Top: M06-L/cc-pVTZ electron density at the B–O bond critical point and second-order interaction energies between represented NBO orbital pairs for (*E*)-*s-cis*-**3a′**. Bottom: MS-CASPT2//SA-CASSCF relative energies of the lowest-lying singlet and triplet states at the critical points along the *Z* → *E* photoisomerization of *s-cis*-**3a′**. Gray, dashed arrows show the vertical and adiabatic triplet energies of (*Z*)-*s-cis*-**3a′**.

We next sought to unveil the origin of the excellent stereoselectivity of Ir-catalyzed photoisomerization. A density functional theory (DFT)-based analysis showed that, regardless of the substituent at the C_α_ position (Me, Bn, or Ph), the *E* isomers are ca. 4 kcal mol^–1^ more stable than their *Z* counterparts (see the Supporting Information for further details). In the *Z* isomers, the atoms of the Bpin group are coplanar with those of the acrylate moiety. In contrast, in *E* isomers, the Bpin group adopts a perpendicular orientation. This gives rise to a stabilizing intramolecular interaction between the p_B_ orbital and the lone pairs at the oxygen in the methyl ester, as corroborated by NBO and wave function topological analyses (Figure 3, top).^11^

To gain further insight into the mechanism of the photoisomerization and the role of the photocatalyst, we mapped the topography of the lowest-lying excited singlet and triplet states of *s-cis*-**3a′** using multiconfigurational approaches (Figure 3, bottom). Pleasingly, MS-CASPT2//SA-CASSCF predicts the same relative stability of the *E* and *Z* isomers as DFT. By starting from either of the ground-state minima, (*Z*)-*s-cis*-**3a′** and (*E*)-*s-cis*-**3a′**, optimization of the T_1_ (π,π*) state leads to the population of an excited-state minimum at 59.6 kcal mol^–1^. This structure is characterized by a 90° twist of the C–C double bond, thereby suggesting the formation of a triplet biradical species. At this point, the S_0_ and T_1_ states are nearly degenerate, which allows intersystem crossing to the ground state and regeneration of the planar acrylate.

Importantly, the participation of excited singlet states in the photoisomerization can be safely ruled out because of their high energy, which surpasses both the employed excitation wavelength (ca. 450 nm, 63.5 kcal·mol^–1^) and the triplet energy of the photocatalyst **PC-7** (62 kcal·mol^–1^). This is also true for the lowest ^3^n,π* state. Therefore, the reaction must proceed through the T_1_ state. Under this scenario, at the position of the (*Z*)-*s-cis*-**3a′** minimum, the energy difference between the S_0_ and T_1_ states (83.7 kcal·mol^–1^) prevents a direct vertical triplet energy transfer mechanism with **PC-7**. Therefore, the photosensitization process likely occurs through a nonclassical triplet energy transfer.^37^ This model has been previously proposed for other endothermic energy transfer processes,^38^ and implies the population of excited rotovibrational levels at the S_0_ state by thermal activation (“hot-band” model) involving single and double bond torsions. From the thermally activated state of the acceptor, the pathway for the population of the T_1_ state will be lower in energy, for which the existence of a minimum in the triplet potential energy surface is not required.^39^ After photoisomerization, the corresponding (*E*)-*s-cis*-**3a′** product displays a strong interaction between the carbonyl group and the boron atom, which is translated into a higher energy difference between the S_0_ and T_1_ states. Additionally, this interaction is likely responsible for preventing torsions of the single and double bonds that allow for the thermal activation required in the nonclassical energy transfer, explaining the selective sensitization of the *Z* isomer. The steric shielding effect of the Bpin group over the double bond after photoisomerization could also prevent orbital overlap between the olefin and the photocatalyst, which is an aspect crucial for the energy transfer to take place.^4a^ The same qualitative analysis applies for *s-cis*-**5a′** (bearing a phenyl group, see the Supporting Information for further details) that, because of the lack of conjugation between the phenyl group and the acrylate moiety (with dihedral angles around 54°), the substituent at Cα does not have a substantial effect on the triplet energies. Therefore, after nonclassical energy transfer with the *Z*-alkenyl boronate, the triplet manifold would be populated. From that point forward, intersystem crossing to the ground state would lead to the formation of the *E* isomer in a thermodynamically driven process.

### 3.2 Photophysical Studies

**Figure 4:**
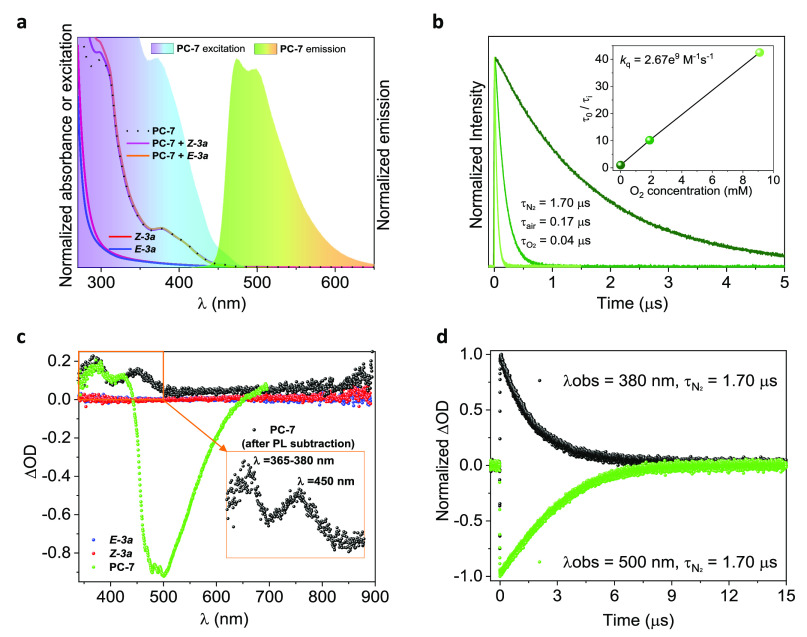
Photophysical characterization of **PC-7** and (*Z*/*E*)-**3a** alkenes in acetonitrile. (a) Normalized absorption (black dot line), excitation (violet–blue), and luminescence (λ_exc_= 400 nm, green) spectra for **PC-7** (30 μM). Normalized absorption spectra for (*Z*)-**3a** (red, pink) and (*E*)-**3a** (blue, royal) alkenes (10 mM) in the absence (red, blue) or in the presence (pink, royal) of **PC-7** (30 μM). (b) Time-resolved luminescence traces for **PC-7** (30 μM) in deaerated (dark green), aerated (green), or purged by O_2_ (light green) environment. Inset: Stern–Volmer plot. (c) Transient absorption spectra (TAS, λ_exc_ = 355 nm, N_2_) for **PC-7** (50 μM) with (black) or without (green) luminescence subtraction. TAS for (*Z*)-**3a** (red) and (*E*)-**3a** (blue) alkenes (10 mM) is included for comparison. Inset: zoomed-in image for **PC-7** after luminescence subtraction. In all cases the data was registered immediately after laser pulse (0 μs). (d) Transient lifetime traces (λ_exc_ = 355 nm, N_2_) for **PC-7** (50 μM) at λ_obs_ = 380 (black) or 500 (green) nm.

To further understand the photoisomerization step, we conducted some photophysical studies using both isomers of alkenyl boronate **3a** and the photocatalyst **PC-7** (Figure 4). The UV–vis spectrum of **PC-7** shows a broad shoulder at ∼400 nm, while its luminescence spectrum (λ_exc_ = 400 nm) displays two maximum peaks at ∼475 and 500 nm. A calculated triplet excited-state energy (*E*_T_) value of 63 kcal·mol^–1^ (Figure 4a) agrees with the reported value.^33^ In addition, a luminescence lifetime (τ_L_) of 167 ns (Figure 4b) and a luminescence quantum yield (φ_L_) of 0.10 in aerated acetonitrile were obtained. Quenching experiments with O_2_ (Figure 4b, inset) suggested the triplet nature of the observed signal with lifetimes of τ = 1700 and 40 ns in deaerated and purged O_2_ solutions, respectively, and a quenching constant of *k*_q_ = 2.67 × 10^9^ M^–1^ s^–1^. Indeed, this is in accordance with the fact that these systems undergo ultrafast intersystem crossing to populate the triplet excited state by means of the heavy atom effect.^40^ Transient absorption spectroscopy (TAS) measurements for **PC-7** (λ_exc_ = 355 nm) under an inert atmosphere (where φ_L_ increased up to 0.90) revealed a positive transient absorption (TA) band at 350 nm and a negative TA band at 475–500 nm, which correspond to the triplet and phosphorescence, respectively (Figure 4c), which present a first-order kinetic transient (and luminescence) lifetime (τ) of 1700 ns (Figure 4d).

**Figure 5:**
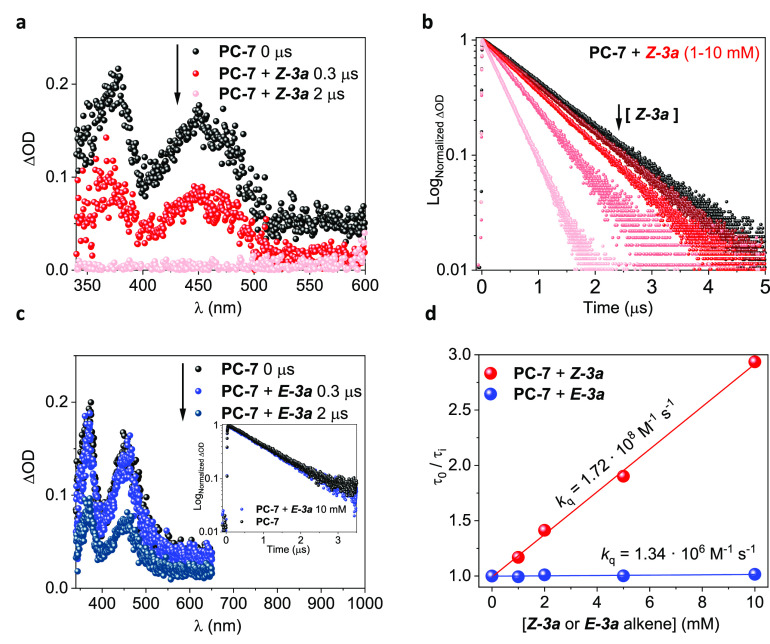
Triplet quenching studies for **PC-7** with model substrate (*Z*)- or (*E*)-**3a** in acetonitrile. (a) TAS (λ_exc_ = 355 nm, N_2_) for **PC-7** in the absence (black) or presence (red) of (*Z*)-**3a** (10 mM) after 0.3 and 2 μs laser pulse. (b) Transient decay traces (λ_exc_ = 355 nm, λ_obs_ = 380 or 500 nm, N_2_) for **PC-7** upon addition of increasing concentrations of (*Z*)-**3a** alkene (up to 10 mM). (c) TAS (λ_exc_ = 355 nm, N_2_) for **PC-7** in absence (black) or presence (blue) of (*E*)-**3a** (10 mM) after 0.3 and 2 μs laser pulse. Inset: corresponding transient decay traces (λ_exc_ = 355 nm, λ_obs_ = 380 or 500 nm, N_2_). (d) Stern–Volmer plots for **PC-7** upon addition of increasing concentrations of (*Z*)-**3a** (red) or (*E*)-**3a** (blue).

Then, triplet quenching experiments by TAS were performed employing (*Z*)**-3a** or (*E*)**-3a** alkenes as quenchers (Figure 5). The addition of (*Z*)**-3a** promoted a highly efficient quenching of the ^**3**^PC-7* TA signal (Figure 5a) with *k*_q_ = 1.72 × 10^8^ M^–1^ s^–1^ (Figure 5d). We did not observe new signals resulting from **PC-7** upon addition of (*Z*)**-3a** (see the Supporting Information for details). In addition, the dynamic quenching observed on the transient decay traces at 380 and 500 nm followed monoexponential functions (Figure 5b). Consequently, we discarded the possibility of a photoinduced electron transfer (PET) process.^41^ Additionally, the absorption of either (*Z*)- or (*E*)-**3a** alkenes and the emission band of the **PC-7** do not overlap (Figure 4a), so a Förster resonance energy transfer (FRET) mechanism is not likely to occur under the reaction conditions.^42^ These pieces of evidence strongly suggest a Dexter energy transfer as the operative mechanism for the photoisomerization process.^42^ In contrast, no changes were observed upon addition of the (*E*)**-3a** isomer (Figure 5c), thereby showing a negligible interaction between the substrate and the photosensitizer after isomerization (*k*_q_ = 1.34 × 10^6^ M^–1^ s^–1^, Figure 5d), which is consistent with the computational studies.

## Conclusions

In summary, this work showcases the potential of the iterative combination of synergistic dual-metal catalysis and visible-light-mediated E_n_T photocatalytic isomerization as an efficient platform for achieving stereodivergence in the 1,2-difunctionalization of internal alkynes. The B_2_pin_2_-carboboration of electron-deficient internal alkynes was selected as a suitable test for this design principle for two main reasons: (1) The unreactive nature of internal alkynoates toward catalytic carboboration, which was addressed by using cooperative Cu/Pd catalysis, is a strategy that overcomes the sluggish reactivity of the β-boryl alkenyl–Cu intermediate and its configurational lability to provide the first general selective carboboration of electron-deficient alkynes with carbon electrophiles, including MeI, benzyl bromides, or ArI. (2) The photoisomerization of the resulting tetrasubstituted β-boryl acrylates via E_n_T catalysis has been accomplished with excellent stereocontrol upon identification of an Ir complex that is competent as a versatile sensitizer for this class of challenging, sterically crowded alkenes. This platform enables late-stage modifications of complex alkynoates and enables stereodivergent access to *E* and *Z* isomers of densely functionalized tetrasubstituted β-boryl acrylates with high synthetic versatility. Computational analysis supports the viability of a phosphine-assisted Cu/Pd transmetalation step in the carboboration process, as well as the thermodynamic preference for the *E* isomer via a n_O_ → p_B_ interaction. Analysis of the excited-state potential energy surfaces state suggests that sensitization from the triplet excited state of the Ir likely occurs through “nonclassical” energy transfer. Finally, photophysical studies show that the reaction occurs by quenching of the triplet excited state of the Ir photocatalyst via a Dexter-type mechanism.

## Supporting Information Available

Experimental details, including complete reaction optimization studies, experimental procedures, and spectral data for products, and computational details, including methodology, conformational analysis, and complete excited-state potential energy surfaces and Cartesian coordinates of optimized structures (PDF)
