# Correction to “Iterative Dual-Metal and Energy Transfer Catalysis Enables Stereodivergence in Alkyne Difunctionalization: Carboboration as Case Study”

**DOI:** 10.1021/acscatal.4c00200

**Published:** 2024-01-22

**Authors:** Javier Corpas, Miguel Gomez-Mendoza, Enrique M. Arpa, Víctor A. de la Peña O’Shea, Bo Durbeej, Juan C. Carretero, Pablo Mauleón, Ramón Gómez Arrayás

There is an error in the list of funding sources. The text should be as given here.

