# Supplementary material for: Iterative Dual-Metal and Energy Transfer Catalysis Enables Stereodivergence in Alkyne Difunctionalization: Carboboration as Case Study
Source: ACS Catal. 2023 Nov 3;13(22):14914–27. doi: 10.1021/acscatal.3c03570 (PMC10662505; doi:10.1021/acscatal.3c03570)
Supplement: Supplementary file 1 — cs3c03570_si_001.pdf [file cs3c03570_si_001.pdf]

Iterative Dual-Metal and Energy Transfer Catalysis enables Stereodivergence in Alkyne Difunctionalization: Carboboration as Case Study

Javier Corpas,[a] Miguel Gómez Mendoza,[b] Enrique M. Arpa,[c] Víctor de la Peña O`Shea,[b] Bo Durbeej,[c] Juan C. Carretero,[a] Pablo Mauleón,*[a] and Ramón Gómez Arrayás,*[a]

[a]Department of Organic Chemistry, Faculty of Science; Institute for Advanced Research in Chemical Sciences (IAdChem); and Centro de Innovación en Química Avanzada (ORFEO-CINQA), Universidad Autónoma de Madrid (UAM), Cantoblanco 28049, Madrid, Spain.

[b]Photoactivated Processes Unit, IMDEA Energy Institute, Technological Park of Mostoles, Avda. Ramón de la Sagra 3, 28935, Madrid, Spain.

[c]Division of Theoretical Chemistry, IFM, Linköping University, 581 83 Linköping, Sweden.

*e-mail for P.M.: pablo.mauleon@uam.es
*e-mail for R.G.A.: ramon.gomez@uam.es

TABLE OF CONTENTS

1. General methods. 3
2. Synthesis of starting materials 3
3. Catalytic Carboboration of Propiolates.................... 9
3.1. Cu/Pd-catalyzed carboboration 9
3.1.1 Optimization studies performed on substrate 1a: methylboration reaction.................... 9
3.1.2 Optimization studies performed on substrate 1a: benzylboration reaction 12
3.1.3 Optimization studies performed on substrate 1a: arylboration reaction.................... 13
3.1.4 Unsuccessful electrophiles 14
3.2 Cu/Pd-catalyzed *syn*-carboboration 15
4 Photoisomerization of tetrasubstituted olefins. 27
5 Determination of stereochemistry 37
6 Computational Studies 42
7 Photophysical Studies 96
8 References 99
9 NMR spectra.................... 101

1. General methods.

Dichloromethane, toluene, tetrahydrofurane, and acetonitrile were taken from a PureSolv MD purification system. Ligands and boronic acids were purchased from commercial sources. Palladium (II) acetate was purchased from commercial sources and used as received. Copper(I) chloride was purchased from commercial sources and purified following a standard procedure.[1] Bis(pinacolato)diboron (B_2pin_2) was generously donated by Frontier Scientific and was used after washing with a diluted solution of HCl.[2] All other compounds were purchased from commercial sources and were used without further purification.

All reactions were carried out in anhydrous solvents and under oxygen or inert atmosphere, indicated in each case. Column liquid chromatographies were performed on silica gel (230-400 mesh ASTM). TLC analysis was performed on 0.2 mm aluminium based plates (60 230-400 mesh). ^{1}H, and ^{13}C NMR spectra were recorded in $CDCl_3$ solutions at 25 °C on AV-300 and AVII-300 (300, 75 MHz, respectively) spectrometers (δ, ppm; J, Hz). ^{1}H and ^{13}C NMR spectra were referenced using the solvent signal as internal standard. In all boron containing compounds, the carbon attached to boron was not observed due to quadrupole broadening caused by the ^{11}B nucleus.[3]

HRMS electron ionization (EI+), electrospray ionization (ESI+) and FAB mass spectra were recorded using an MicroToF Q, API-QToF ESI with a mass range from 20 to 3000 m/z and mass resolution 15000 (FWHM). Melting points were determined in open-end capillary tubes.

A custom-made photoreactor setup was used for the photocatalytic reactions developed by *Servicios Generales de Apoyo a la Investigación Experimental* (SEGAINVEX) at *Universidad Autónoma de Madrid* (UAM). Photochemical reactions were carried out using borosilicate glass vials. For reactions at room temperature, the vial was placed inside the fitted well in which irradiation takes place at 465 nm using 350 mW single LEDs located 1 cm beneath the base of the vial (see Figure S1). Reaction temperature is kept at 20-25 ºC using a recirculating chiller.

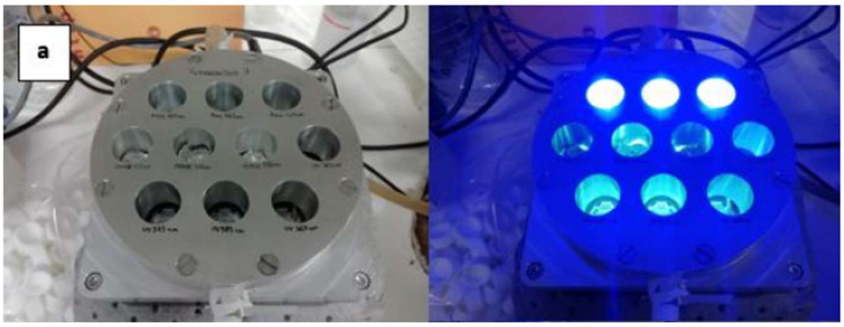

Figure S1. Experimental setup for photoreactions.

2. Synthesis of starting materials

Methyl 6-hydroxyhex-2-ynoate (I4):

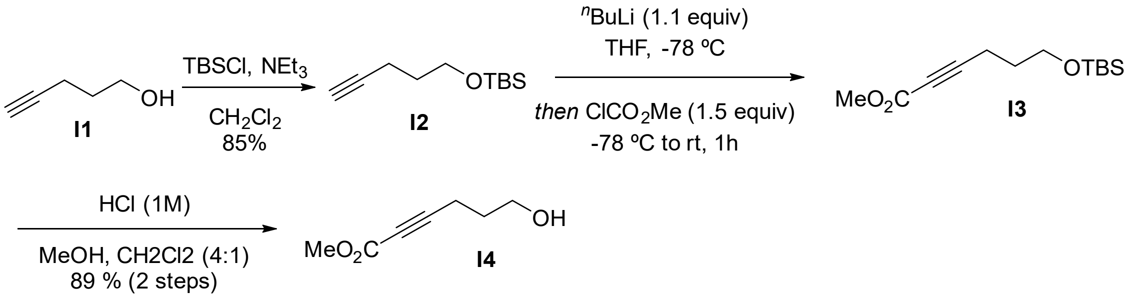

Precursor **I4** was synthesized according to a modified reported method:[4] TBS ether **I2** was prepared from 4-pentyn-1-ol (I1, 0.553 mL, 5.94 mmol, 1.0 equiv) upon treatment with NEt_3 (1.24 mL, 1.5 equiv) and TBSCl (1.07 g, 1.2 equiv) in CH_2Cl_2 (20 mL) under a nitrogen atmosphere at 0 °C. The solution was allowed to stir overnight at room temperature, before being quenched with a saturated solution of NH_4Cl (1x20 mL) and the resulting organic phase was washed with water (1x 20 mL) and brine (1x 20 mL). The resulting organic phase was dried with Na_2SO_4, filtered and the resulting the solvent was removed from vacuo yielding a yellow oil which was further purified by column chromatography (SiO_2, heptane:AcOEt (100:5)), to give silyl ether **I2** (1.0, 85%) as a colorless oil.
Synthesis of methyl ester **I3**: ^{n}BuLi (2.22 mL, 2.5 M in hexanes, 5.56 mmol, 1.1 equiv) was added dropwise to a stirred solution of alkyne **I2** (1.0, 5.05 mmol, 1.0 equiv) in THF (25 mL) under a nitrogen atmosphere at −78 °C. The solution was allowed to stir at −78 °C for 20 min before $ClCO_2Me$ (0.58 mL, 7.58 mmol, 1.5 equiv) was added dropwise. The resulting solution was allowed to warm to 0 °C and stirred for 1.5 h, before being quenched with sat. aq. NH_4Cl (25 mL). The layers were separated, and the aqueous layer was extracted with AcOEt (3 × 25 mL). The combined organic phases were washed with brine (25 mL) and dried over Na_2SO_4. Filtration and removal of the solvent under reduced pressure gave methyl ester **I3** as a yellow oil which was taken directly for the next step without further purification.

Synthesis of **I4**: alkyne **I3** (1.29 g, 5.05 mmol, 1.0 equiv) was treated with a solution of 1.0 M HCl (7.58 mL, 7.58 mmol, 1.5 equiv) was in $MeOH/CH_2Cl_2$ (4:1, 17 mL) at 0 °C. The solution was allowed to warm to room temperature and stirred for 24 h. CH_2Cl_2 (25 mL), brine (25 mL) and H_2O (10 mL) were added, and the layers separated. The aqueous layer was extracted with CH_2Cl_2 (3 × 25 mL), and the combined organic phases were washed with brine (25 mL) and dried over Na_2SO_4. Filtration and removal of the solvent under reduced pressure resulted in a yellow oil, which was purified by flash chromatography (SiO_2, 5% to 50% EtOAc/n-heptane), affording alcohol **I4** (639 mg, 89% over two steps) as an oil. Spectroscopic data were identical to those reported in the literature.[1]

Synthesis of Alkynes (procedure 1):

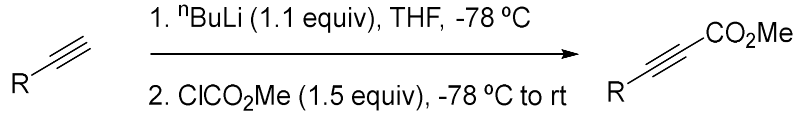

The corresponding terminal alkyne (1 mmol, 1.0 equiv) in THF (50 mL) was treated with ^{n}BuLi (0.44 mL, 2.5 M, 1.1 mmol, 1.1 equiv) by dropwise addition at -78 °C. After that, the reaction was allowed to stir for 30 min at -78 °C and then methyl chloroformate (0.12 mL, 1.5 mmol, 1.5 equiv) was added dropwise at -78 °C. The reaction mixture was stirred at -78 °C for 1 h before reaching room temperature. Then, the reaction mixture was quenched with a saturated solution of NH_4Cl (100 mL) at 0 °C. The layers were separated, and the aqueous layer was extracted with AcOEt (3 × 50 mL). The combined organic phases were washed with brine (100 mL) and dried over Na_2SO_4. Filtration and removal of the solvent under reduced pressure gave an oil which was further purified by silica gel chromatography.

Synthesis of Alkynes (procedure 2):

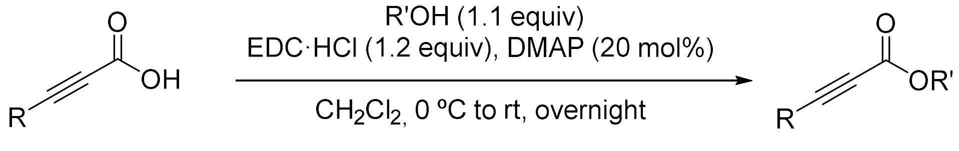

General procedure for the esterification reaction (procedure 2):[5] to a solution of the carboxylic acid (1 mmol, 1.0 equiv) in CH_2Cl_2 (10 mL) was added EDC·HCl (230 mg, 1.2 mmol, 1.2 equiv) at 0 ºC followed by a catalytic amount of DMAP (24 mg, 0.2 mmol, 20 mol%). Then, the corresponding alcohol (1.1 equiv) was added to the mixture and the reaction was allowed to stir for 30 min at 0ºC. After that, the resulting solution was stirred overnight. Then, the reaction mixture was washed with H_2O (3 x 15 mL). The layers were separated, and the aqueous layer was extracted with CH_2Cl_2 (2 × 20 mL). The combined organic phases were washed with brine (15 mL) and dried over Na_2SO_4. Filtration and removal of the solvent under reduced pressure gave a residue which was further purified by silica gel chromatography.

Synthesis of Alkynes (procedure 3):

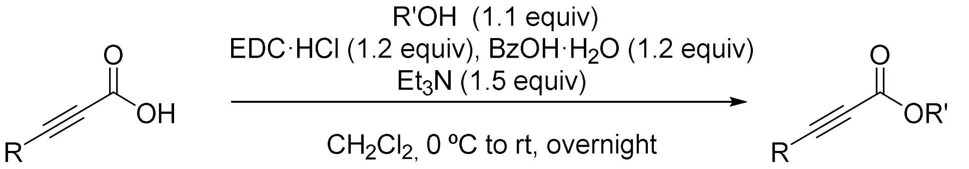

General procedure for the esterification reaction (procedure 3):[6] to a solution of the carboxylic acid (1 mmol, 1.0 equiv) in CH_2Cl_2 (10 mL) was added EDC·HCl (230 mg, 1.2 mmol, 1.2 equiv) at 0 ºC followed by Et_3N (0.21 mL, 1.5 mmol, 1.5 equiv) and 1-hydroxybenzotriazole hydrate (BzOH·H_2O, (162.14 mg, 1.2 mmol, 1.2 equiv). Then, the corresponding alcohol or amine (1.1 equiv) was added to the mixture and the reaction was allowed to stir for 30 min at 0ºC. After that, the resulting solution was stirred overnight. Then, the reaction mixture was washed with H_2O (3 x 15 mL). The layers were separated, and the aqueous layer was extracted with CH_2Cl_2 (2 × 20 mL). The combined organic phases were washed with brine (15 mL) and dried over Na_2SO_4. Filtration and removal of the solvent under reduced pressure gave a residue which was further purified by silica gel chromatography.

Methyl 3-cyclohexylpropiolate (1b):

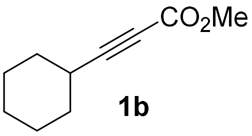

1b

Following the procedure 1, the treatment of cyclohexylacetylene (0.13 mL, 1 mmol, 1.0 equiv) with ^{n}BuLi (0.44 mL, 2.5 M, 1.1 mmol, 1.1 equiv), and methyl chloroformate (0.12 mL, 1.5 mmol, 1.5 equiv) afforded the desired alkyne **1b** after purification by column chromatography (SiO_2, n-heptane:AcOEt, 20:1) as a colorless oil (156 mg, 94% yield). **^{1}H-NMR** (300 MHz, $CDCl_3$): δ 3.68 (s, 3H), 2.45 (tt, J = 8.8, 3.7 Hz, 1H), 1.83 – 1.71 (m, 2H), 1.70-1.58 (m, 2H), 1.54-1.37 (m, 3H), 1.34-1.20 (m, 3H). **^{13}C-NMR** (75 MHz, $CDCl_3$): δ154.3, 93.1, 72.8, 52.4, 31.4, 28.8, 25.6, 24.6. **HRMS** (ESI+): *m/z* $[M + H]^+$ calcd for $C_{10}H_{15}O_2$ 167.1067, found 167.1072.

Cycloheptyl hex-2-ynoate (1c):

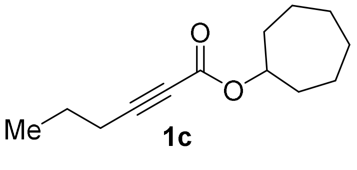

1c

Following the general procedure 2, the treatment of 2-hexynoic acid (0.11 mL, 1 mmol, 1.0 equiv) with EDC·HCl (230 mg, 1.2 mmol, 1.2 equiv) and DMAP (24 mg, 0.2 mmol, 20 mol%) in presence of cycloheptanol (0.13 mL, 1.1 mmol, 1.1 equiv) yielded the titled compound after purification by column chromatography (SiO_2, n-heptane:AcOEt, 20:1). as an oil (168.2 mg, 82%). **^{1}H-NMR** (300 MHz, $CDCl_3$): δ 4.99 (tt, J = 8.3, 4.3 Hz, 1H), 2.29 (t, J = 7.1 Hz, 2H), 1.98 – 1.87 (m, 2H), 1.71 – 1.52 (m, 10H), 1.49-1.36 (m, 2H), 1.00 (t, J = 7.4 Hz, 3H). **^{13}C-NMR** (75 MHz, $CDCl_3$): δ 153.5, 88.9, 77.2, 73.9, 33.8, 28.4, 23.0, 21.3, 20.9, 13.6. **HRMS** (APCI +) *m/z* $[M + H]^+$ calcd for $C_{13}H_{21}O_2$ 209.1536, found 209.1536.

Methyl 7-chlorohept-2-ynoate (1d):

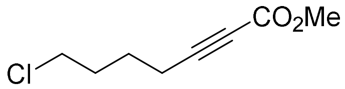

1d

Following the procedure 1, the treatment of 6-chloro-1-hexyne (0.12 mL, 1 mmol, 1.0 equiv) with ^{n}BuLi (0.44 mL, 2.5 M, 1.1 mmol, 1.1 equiv), and methyl chloroformate (0.12 mL, 1.5 mmol, 1.5 equiv) afforded the desired alkyne **1d** after purification by column chromatography (SiO_2, n-heptane:AcOEt, 15:1) as a colorless oil (156 mg, 72% yield). ^{1}**H-NMR** (300 MHz, $CDCl_3$): δ 3.67 (s, 3H), 3.50 (t, *J* = 6.1 Hz, 2H), 2.33 (t, *J* = 6.8 Hz, 2H), 1.91 – 1.76 (m, 2H), 1.75-1.62 (m, 2H). ^{13}**C-NMR** (75 MHz, $CDCl_3$): δ154.0, 88.5, 73.3, 52.5, 44.1, 31.3, 24.7, 17.9. **HRMS** (ESI+) m/z [M + H]+ calcd for $C_8H_{12}ClO_2$, found 175.6323.

Methyl 6-cyanohex-2-ynoate (1e):

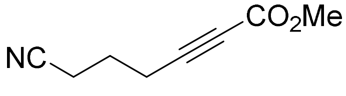

1e

Following the procedure 1, the treatment of 5-hexynenitrile (0.11 mL, 1 mmol, 1.0 equiv) with ^{n}BuLi (0.44 mL, 2.5 M, 1.1 mmol, 1.1 equiv), and methyl chloroformate (0.12 mL, 1.5 mmol, 1.5 equiv) afforded the desired alkyne **1e** after purification by column chromatography (SiO_2, n-heptane:AcOEt, 15:1) as a colorless oil (156 mg, 72% yield). ^{1}**H-NMR** (300 MHz, $CDCl_3$): δ 3.67 (s, 3H), 3.50 (t, *J* = 6.1 Hz, 2H), 2.33 (t, *J* = 6.8 Hz, 2H), 1.91 – 1.76 (m, 2H), 1.75-1.62 (m, 2H). The spectroscopic values matched the reported values.[7]

(*8R,9S,13S,14S*)-13-methyl-17-oxo-7,8,9,11,12,13,14,15,16,17-decahydro-6*H*-cyclopenta[a]phenanthren-3-yl hex-2-ynoate (1f):

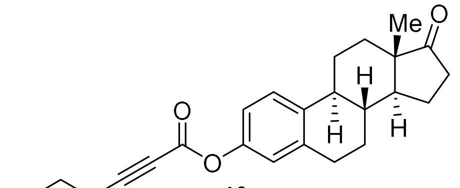

1f

Following the general procedure 2, the treatment of 2-hexynoic acid (0.11 mL, 1 mmol, 1.0 equiv) with EDC·HCl (230 mg, 1.2 mmol, 1.2 equiv) and DMAP (24 mg, 0.2 mmol, 20 mol%) in presence of estrone (CAS: 53-16-7, 297.4 mg, 1.1 mmol, 1.1 equiv) yielded the titled compound after purification by column chromatography (SiO_2, n-heptane:AcOEt, 10:1 to 5:1). as white solid (292 mg, 80%). ^{1}**H-NMR** (300 MHz, $CDCl_3$): δ 7.28 (d, J = 8.5 Hz, 1H), 6.89 (dd, J = 8.4, 2.5 Hz, 1H), 6.85 (d, J = 2.5 Hz, 1H), 2.90 (dd, J = 8.7, 4.1 Hz, 2H), 2.50 (dd, J = 18.3, 8.3 Hz, 1H), 2.37 (t, J = 7.0 Hz, 2H), 2.34 – 2.22 (m, 1H), 2.23-2.11 (m, 1H), 2.10-1.90 (m, 3H), 1.72 – 1.32 (m, 8H), 1.04 (t, J = 7.4 Hz, 3H), 0.90 (s, 3H). ^{13}**C-NMR** (75 MHz, $CDCl_3$): δ 220.8, 152.5, 148.1, 138.3, 138.0, 126.6, 121.5, 118.7, 92.0, 73.1, 50.5, 48.0, 44.3, 38.1, 35.9, 31.7, 29.5, 26.4, 25.8, 21.7, 21.1, 20.9, 13.9, 13.6. **HRMS** (ESI+) m/z [M + H]+ calcd for $C_{24}H_{29}O_3$ 365.2111, found 365.2114.

***rac*-(*R*)-2,5,7,8-tetramethyl-2-((*4R,8R*)-4,8,12-trimethyltridecyl)chroman-6-yl hex-2-ynoate (1g):**

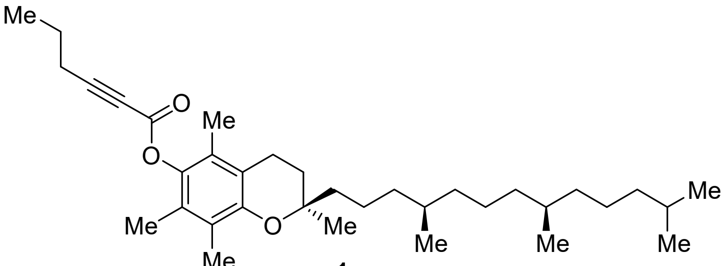

1g

Following the general procedure 2, the treatment of 2-hexynoic acid (0.11 mL, 1 mmol, 1.0 equiv) with EDC·HCl (230 mg, 1.2 mmol, 1.2 equiv) and DMAP (24 mg, 0.2 mmol, 20 mol%) in presence of α-tocopherol (CAS: 10191-41-0, 473.8 mg, 1.1 mmol, 1.1 equiv) yielded the titled compound after purification by column chromatography (SiO_2, n-heptane:AcOEt, 40:1) as yellow oil (509 mg, 97%). ^{1}**H-NMR** (300 MHz, $CDCl_3$): δ 2.60 (t, J = 6.8 Hz, 2H), 2.39 (td, J = 7.1, 1.7 Hz, 2H), 2.10 (s, 3H), 2.07 (s, 3H), 2.03 (s, 3H), 1.78 (ddd, J = 21.4, 14.3, 7.0 Hz, 2H), 1.73 – 1.62 (m, 2H), 1.59 – 1.05 (m, 26H), 0.92-0.84 (m, 13H). ^{13}**C-NMR** (75 MHz, $CDCl_3$): δ 152.8, 149.9, 140.2, 126.8, 125.1, 123.3, 117.6, 91.4, 75.3, 73.1, 39.5, 37.6 (2C), 37.5, 37.4, 32.9, 32.8 (2C), 28.1, 25.0, 24.6, 22.9, 22.8, 21.2, 21.1, 20.9, 20.7, 19.9, 19.8. **HRMS** (ESI+) m/z [M + H]+ calcd for $C_{35}H_{57}O_3$ 525.4302, found 525.4298.

Cyclopropyl(phenyl)methyl hex-2-ynoate (1h):

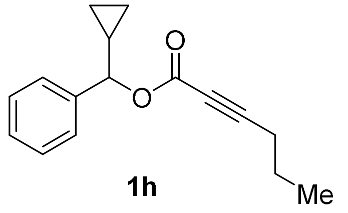

1h

Following the general procedure 2, the treatment of 2-hexynoic acid (0.11 mL, 1 mmol, 1.0 equiv) with EDC·HCl (230 mg, 1.2 mmol, 1.2 equiv) and DMAP (24 mg, 0.2 mmol, 20 mol%) in presence of -cyclopropylbenzyl alcohol (CAS: 1007-03-0, 0.16 mL, 1.1 mmol, 1.1 equiv) yielded the titled compound after purification by column chromatography (SiO_2, n-heptane:AcOEt, 10:1 to 5:1). as white solid (223 mg, 92%). **M.p.:** 101-103 °C. **^{1}H-NMR** (300 MHz, $CDCl_3$): δ 7.32 – 7.21 (m, 5H), 5.18 (d, J = 8.9 Hz, 1H), 2.21 (t, J = 7.1 Hz, 2H), 1.51 (h, J = 7.2 Hz, 2H), 1.36 – 1.23 (m, 1H), 0.92 (t, J = 7.4 Hz, 3H), 0.62 – 0.43 (m, 3H), 0.35-0.27 (m, 1H). **^{13}C-NMR** (75 MHz, $CDCl_3$): δ 153.4, 139.6, 128.5, 128.1, 126.8, 89.8, 81.7, 73.5, 21.1, 20.7, 16.5, 13.6, 4.5, 3.3. **HRMS** (ESI+) *m/z* $[M + Na]^+$ calcd for $C_{16}H_{18}NaO_2$ 265.1199, found 265.1193.

(*S*)-(4-(prop-1-en-2-yl)cyclohex-1-en-1-yl)methyl hex-2-ynoate (1i):

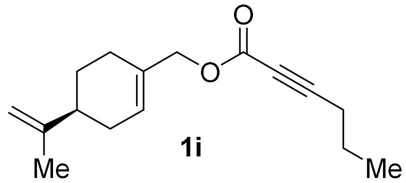

1i

Following the general procedure 2, the treatment of 2-hexynoic acid (0.11 mL, 1 mmol, 1.0 equiv) with EDC·HCl (230 mg, 1.2 mmol, 1.2 equiv) and DMAP (24 mg, 0.2 mmol, 20 mol%) in presence of (*S*)-(-)-perillyl alcohol (CAS: 18457-55-1, 0.18 mL, 1.1 mmol, 1.1 equiv) yielded the titled compound after purification by column chromatography (SiO_2, n-heptane:AcOEt, 15:1). as an oil (224 mg, 91%). **^{1}H-NMR** (300 MHz, $CDCl_3$): δ 5.83 – 5.75 (m, 1H), 4.74-4.69 (m, 2H), 4.54 (s, 2H), 2.30 (t, J = 7.1 Hz, 2H), 2.20 – 2.06 (m, 4H), 2.04 – 1.91 (m, 1H), 1.90 – 1.79 (m, 1H), 1.73 (s, 3H), 1.62 (dt, J = 14.5, 7.3 Hz, 2H), 1.56 – 1.44 (m, 1H), 1.01 (t, J = 7.4 Hz, 3H). **^{13}C-NMR** (75 MHz, $CDCl_3$): δ 154.0, 149.6, 132.0, 127.0, 108.9, 89.6, 73.3, 69.8, 40.8, 30.6, 27.4, 26.5, 21.2, 20.8, 20.7, 13.6. **HRMS** (ESI+) m/z $[M + NH_4]^+$ calcd for $C_{16}H_{26}NO_2$ 264.1958, found 264.1958.

But-3-en-1-yl hex-2-ynoate (1j):

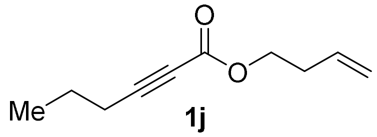

1j

Following the general procedure 2, the treatment of 2-hexynoic acid (0.11 mL, 1 mmol, 1.0 equiv) with EDC·HCl (230 mg, 1.2 mmol, 1.2 equiv) and DMAP (24 mg, 0.2 mmol, 20 mol%) in presence of 3-buten-1-ol (94 mg, 1.1 mmol, 1.1 equiv) yielded the titled compound after purification by column chromatography (SiO_2, n-heptane:AcOEt, 20:1). as an oil (161.2 mg, 97%). **^{1}H-NMR** (300 MHz, $CDCl_3$): δ 5.77 (ddt, J = 17.0, 10.2, 6.7 Hz, 1H), 5.17 – 4.99 (m, 2H), 4.18 (t, J = 6.8 Hz, 2H), 2.41 (qt, J = 6.9, 1.4 Hz, 2H), 2.29 (t, J = 7.1 Hz, 2H), 1.59 (h, J = 7.3 Hz, 2H), 0.99 (t, J = 7.4 Hz, 3H). **^{13}C-NMR** (75 MHz, $CDCl_3$): δ 153.9, 133.6, 117.6, 89.6, 73.3, 64.8, 32.9, 21.2, 20.7, 13.6. **HRMS** (ESI+) m/z $[M + H]^+$ calcd for $C_{10}H_{15}O_2$ 167.1067, found 167.1064.

5-(Trimethylsilyl)pent-4-yn-1-yl hex-2-ynoate (1k):

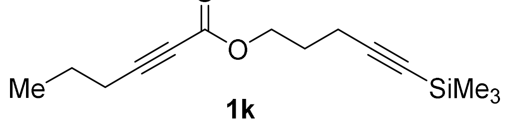

1k

Following the general procedure 2, the treatment of 2-hexynoic acid (0.11 mL, 1 mmol, 1.0 equiv) with EDC·HCl (230 mg, 1.2 mmol, 1.2 equiv) and DMAP (24 mg, 0.2 mmol, 20 mol%) in presence of 5-(Trimethylsilyl)-4-pentyn-1-ol (0.2 mL, 1.1 mmol, 1.1 equiv) yielded the titled compound after purification by column chromatography (SiO_2, n-heptane:AcOEt, 20:1). as an oil (161.2 mg, 97%). **^{1}H-NMR** (300 MHz, $CDCl_3$): δ 4.24 (t, J = 6.3 Hz, 2H), 2.32 (dt, J = 9.9, 7.0 Hz, 4H), 1.87 (p, J = 6.7 Hz, 2H), 1.68-1.53 (m, 2H), 1.01 (t, J = 7.4 Hz, 3H), 0.14 (s, 9H). **^{13}C-NMR** (75

MHz, $CDCl_3$): δ 154.0, 105.6, 89.7, 85.7, 73.3, 64.5, 27.6, 21.2, 20.8, 16.7, 13.6, 0.2. **HRMS** (APCI +) m/z [M + H]+ calcd for $C_{14}H_{23}O_2Si$ 251.1462, found 251.1462.

Methyl 6-(2-(1-(4-chlorobenzoyl)-5-methoxy-2-methyl-1*H*-indol-3-yl)acetoxy)hex-2-ynoate (1l):

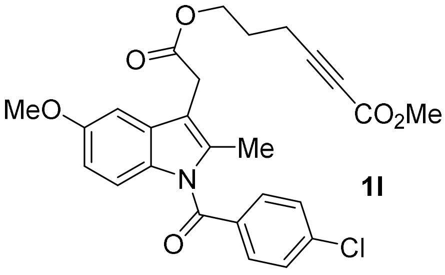

1l

Following the general procedure 2, the treatment of Indomethacin (CAS: 53-86-1, 357.7 mg, 1.0 mmol, 1.0 equiv) with EDC·HCl (230 mg, 1.2 mmol, 1.2 equiv) and DMAP (24 mg, 0.2 mmol, 20 mol%) in presence of precursor **I4** (156.2 mg, 1.1 mmol, 1.1 equiv) yielded the titled compound after purification by column chromatography (SiO_2, n-heptane:AcOEt, 10:1 to 3:1) as yellow solid (434 mg, 90%). **M.p.:** 96-98 °C. **^{1}H-NMR** (300 MHz, $CDCl_3$): δ 7.65 (d, J = 8.4 Hz, 2H), 7.46 (d, J = 8.4 Hz, 2H), 6.94 (t, J = 2.3 Hz, 1H), 6.85 (dd, J = 9.0, 4.7 Hz, 1H), 6.68-6.62 (m, 1H), 4.19 (t, J = 6.1 Hz, 2H), 3.83 (s, 3H), 3.74 (s, 3H), 3.67 (s, 2H), 2.40 – 2.30 (m, 5H), 1.95 – 1.83 (m, 2H). **^{13}C-NMR** (75 MHz, $CDCl_3$): δ 170.8, 168.4, 156.2, 154.0, 139.4, 136.0, 134.0, 131.3, 130.9, 130.7, 129.2, 115.1, 112.5, 111.8, 101.3, 87.9, 73.5, 63.3, 55.8, 52.7, 30.4, 26.8, 15.6, 13.4. **HRMS** (ESI+) m/z [M + H]+ calcd for $C_{26}H_{25}ClNO_6$ 482.1365, found 482.1380.

Methyl 6-(2-(1,3-dimethyl-2,6-dioxo-1,2,3,6-tetrahydro-7H-purin-7-yl)acetoxy)hex-2-ynoate (1h):

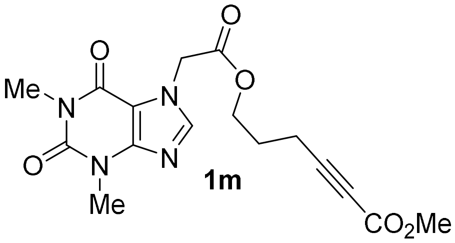

1m

Following the general procedure 2, the treatment of theophylline-7-acetic acid (CAS: 652-37-9, 238.2 mg, 1.0 mmol, 1.0 equiv) with EDC·HCl (230 mg, 1.2 mmol, 1.2 equiv) and DMAP (24 mg, 0.2 mmol, 20 mol%) in presence of precursor **I4** (156.2 mg, 1.1 mmol, 1.1 equiv) yielded the titled compound after purification by column chromatography (SiO_2, n-heptane:AcOEt, 2:1 to AcOEt:MeOH (1%)) as yellow oil (239 mg, 66%). **^{1}H-NMR** (300 MHz, $CDCl_3$): δ 7.64 (s, 1H), 5.12 (s, 2H), 4.34 (t, J = 6.1 Hz, 2H), 3.76 (s, 3H), 3.61 (s, 3H), 3.38 (s, 3H), 2.47 (t, J = 6.9 Hz, 2H), 2.04 – 1.93 (m, 2H). **^{13}C-NMR** (75 MHz, $CDCl_3$): δ 167.0, 155.1, 153.9, 151.5, 148.5, 142.0, 107.0, 87.7, 73.4, 64.5, 52.6, 47.2, 29.7, 27.8, 26.4, 15.4. **HRMS** (ESI+) m/z [M + H]+ calcd for $C_{16}H_{19}N_4O_6$ 363.1299, found 363.1308.

Benzyl 3-(hex-2-ynoyloxy)azetidine-1-carboxylate (1n):

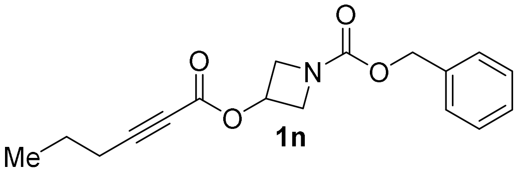

1n

Following the general procedure 2, the treatment of 2-hexynoic acid (0.11 mL, 1 mmol, 1.0 equiv) with EDC·HCl (230 mg, 1.2 mmol, 1.2 equiv) and DMAP (24 mg, 0.2 mmol, 20 mol%) in presence of 1-Cbz-3-Hydroxyazetidine (CAS: 128117-22-6, 228 mg, 1.1 mmol, 1.1 equiv) yielded the titled compound after purification by column chromatography (SiO_2, n-heptane:AcOEt, 6:1). as an oil (262 mg, 87%). **^{1}H-NMR** (300 MHz, $CDCl_3$): δ 7.38 – 7.25 (m, 5H), 5.27 – 5.14 (m, 1H), 5.10 (s, 2H), 4.33 (dd, J = 10.2, 6.8 Hz, 2H), 4.08 – 3.95 (m, 2H), 2.33 (t, J = 7.0 Hz, 2H), 1.61 (p, J = 7.2 Hz, 2H), 1.02 (t, J = 7.4 Hz, 3H). **^{13}C-NMR** (75 MHz, $CDCl_3$): δ 156.3, 152.9, 136.5, 128.6, 128.3, 128.2, 91.4, 72.7, 67.0, 64.8, 56.3, 21.1, 20.8, 13.6. **HRMS** (ESI+) m/z [M + H]+ calcd for $C_{17}H_{20}NO_4$ 302.1387, found 302.1394.

3-(Naphthalen-1-yl)prop-2-yn-1-yl hex-2-ynoate (1o):

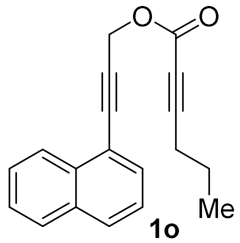
1o

Following the general procedure 2, the treatment of 2-hexynoic acid (0.11 mL, 1 mmol, 1.0 equiv) with EDC·HCl (230 mg, 1.2 mmol, 1.2 equiv) and DMAP (24 mg, 0.2 mmol, 20 mol%) in presence of 3-(1-naphthyl)-2-propyn-1-ol (CAS: 16176-22-0, 200 mg, 1.1 mmol, 1.1 equiv) yielded the titled compound after purification by column chromatography (SiO_2, n-heptane:AcOEt, 15:1). as an oil (260 mg, 94%). **^{1}H-NMR** (300 MHz, $CDCl_3$): δ 8.32 (d, J = 8.3 Hz, 1H), 7.85 (d, J = 8.2 Hz, 2H), 7.70 (d, J = 8.0 Hz, 1H), 7.62-7.48 (m, 2H), 7.42 (dd, J = 8.2, 7.2 Hz, 1H), 5.14 (s, 2H), 2.34 (t, J = 7.1 Hz, 2H), 1.63 (h, J = 7.3 Hz, 2H), 1.03 (t, J = 7.4 Hz, 3H). **^{13}C-NMR** (75 MHz, $CDCl_3$): δ 153.2, 133.5, 133.2, 131.2, 129.5, 128.4, 127.1, 126.6, 126.2, 125.2, 119.7, 91.1, 87.0, 85.4, 72.8, 54.2, 21.1, 20.8, 13.6. **HRMS** (GC EI+) m/z [M]+ calcd for $C_{19}H_{16}O_2$ 276.1150, found 276.1140.

6-Methoxy-6-oxohex-4-yn-1-yl(*2S,4aR,7S,9aS,10S*)-1-methyl-8-methylene-13-oxo-2,7-bis((triethylsilyl)oxy)-1,2,4b,5,6,7,8,9,10,10a-decahydro-4a,1-(epoxymethano)-7,9a-methanobenzo[*a*]azulene-10-carboxylate (1p):

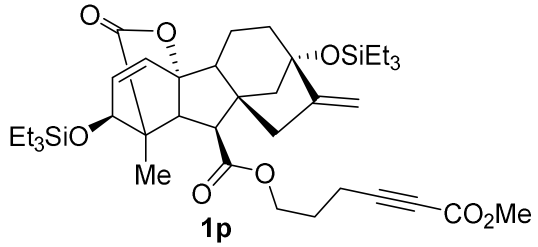
1p

Following the general procedure 2, the treatment of silylated gibberellic acid[8] (574.9 mg, 1.0 mmol, 1.0 equiv) with EDC·HCl (230 mg, 1.2 mmol, 1.2 equiv) and DMAP (24 mg, 0.2 mmol, 20 mol%) in presence of precursor **I4** (156.2 mg, 1.1 mmol, 1.1 equiv) yielded the titled compound after purification by column chromatography (SiO_2, n-heptane:AcOEt, 20:1, 10:1 to 5:1) as an oil (549.6 mg, 71%). **^{1}H-NMR** (300 MHz, $CDCl_3$): δ 6.12 (d, J = 9.3 Hz, 1H), 5.66 (dd, J = 9.3, 3.6 Hz, 1H), 5.13 (s, 1H), 4.79 (s, 1H), 4.13 (td, J = 6.3, 2.7 Hz, 2H), 4.04 (d, J = 3.6 Hz, 1H), 3.66 (s, 3H), 3.22 (d, J = 10.9 Hz, 1H), 2.66 (d, J = 10.9 Hz, 1H), 2.35 (t, J = 7.2 Hz, 2H), 2.07 – 1.74 (m, 9H), 1.09 (s, 3H), 0.91 – 0.81 (m, 18H), 0.57 – 0.45 (m, 12H). **^{13}C-NMR** (75 MHz, $CDCl_3$): δ 179.1, 171.8, 157.0, 154.0, 131.6, 107.5, 90.9, 87.3, 79.2, 73.7, 70.1, 63.2, 54.3, 52.8, 52.7, 51.2, 51.1, 50.8, 44.4, 42.9, 40.7, 26.8, 17.1, 15.7, 15.1, 7.1, 6.9, 6.7, 5.1. **HRMS** (ESI+) m/z [M + H]+ calcd for $C_{38}H_{59}O_8Si_2$ 699.3743, found 699.3765.

3. Catalytic Carboboration of Propiolates

3.1. Cu/Pd-catalyzed carboboration

3.1.1 Optimization studies performed on substrate 1a: methylboration reaction

We started the exploration of the potential carboboration of alkynoate **1a** employing the typical conditions for related transformation as already described in the literature[9] employing MeI as the electrophile (Table S1). When 1.5 equiv. of MeI in combination with NaO^{t}Bu (1.5 equiv) were added using a combination of CuCl and PCy_3 as catalyst system we observed the desired carboboration product *Z*-**3a** (*CB*) in the reaction crude, albeit the main product of the reaction was the hydroborylated product (*HB*). A 32% combined yield and a 19:81 ratio of carboboration *vs* hydroboration products (*CB:HB*) were obtained by using these conditions. Interestingly, increasing the quantity of MeI from 1.5 to 2.5 equivalents a higher chemoselectivity toward the carboborylated product was observed with a 37:63 ratio (entry 2). Following this trend, the addition of 3.0 and 4.0 equivalents of the electrophile raised the quantity of carboborylation product and the overall yield, but mixtures of carboboration and hydroboration products were present in the reaction crude for all the cases (entries 3 and 4, respectively). The addition of more base (4.0 equiv) in presence of high quantities of MeI (4.0 equiv) led to a slight enrichment of the carboboration product (*CB:HB* = 60:40), but a low 33% overall yield, thus indicating that high quantities of base are not well-tolerated (entry 5). Importantly, the use of other solvents did

not improved the observed reactivity. These results suggest that the intermediate alkenyl-copper is very low nucleophilic and therefore the carboboration strategy using a single metal catalyst is not effective. To corroborate this behavior we also tested the reaction using benzyl bromide as the electrophile (4.0 equiv) but no carboboration product was observed, thus reinforcing our hypothesis (entry 7). With these results in our hands, we decided to explore a strategy based on a Cu/Pd transmetallation to ensure the formation of a more active alkenyl-metal species which could be active in the carboborylation process via an alternative mechanism. Satisfyingly, when 5 mol % of $PdCl_2(PPh_3)_2$ was added to the reaction media in presence of low quantities of MeI and base (1.5 equiv. of each) the hydroboration reaction was inhibited and only the corresponding carboboration product *Z*-**3a** was obtained with complete *syn*-stereoselectivity and 84% yield (entry 6).

Table S1. Optimization for methylboration of 1a.

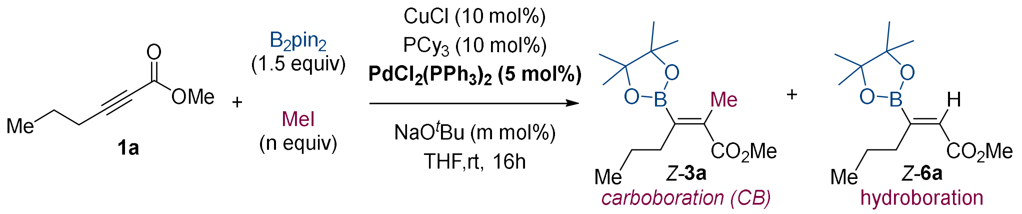

Entry	MeI (n equiv)	NaO^{t}Bu (m equiv)	$PdCl_2(PPh_3)_2$	yield *(%)*[a]	CB:HB *(%)*[a]
1	1.5	1.5	-	32	19:81
2	2.5	1.5	-	41	37:63
3	3.0	2.0	-	57	49:51
4[b]	4.0	2.0	-	62	59:41
5	4.0	4.0	-	33	60:40
6	1.5	1.5	5 mol%	84	> 98:2
7[c]	4.0	2.0	-	16	< 2:98

[a]Determined in the reaction crude by ^{1}H NMR spectroscopy employing 1,3,5-trimethoxybenzene (TMB) as an internal standard. [b]The use of other solvents such as toluene, acetonitrile or DMF did not improved this result. [c]Using BnBr as the electrophile.

Having found suitable conditions to perform the carboboration with propiolate-based systems we then explored the effect of other ligands for the copper salt in presence of $PdCl_2(PPh_3)_2$ (Table S2). When the PCy_3 phosphine was replaced by the bidentate XantPhos ligand a similar result was obtained with 81% yield (entry 2). The use of the more flexible DPEPhos ligand, which has a lower bite angle in comparison with XantPhos, led to the carboboration product with complete stereoselectivity, but a slightly lower yield (74%, entry 3). A similar result was obtained with PPh_3, which is less electron-rich than the PCy_3 analog (77%, entry 4). Other bidentate phosphines such as BINAP harmed the reaction, as a 52% yield was obtained (entry 5). The use of the ^{i}PrCuCl carbene-based copper complex was also less effective in the process (69% yield, entry 6). Importantly, when the reaction was run in the absence of the copper salt a variety of non-identified products was observed in the reaction crude by ^{1}H NMR spectroscopy with the carboborylated product *Z*-**3a** being absent, thus indicating that both Cu- and Pd-catalysts are needed for an efficient carboboration. Finally, we determined that by lowering the catalyst loading from 10 mol% to 5 mol% of copper and from 5 mol% to 2.5 mol% of the palladium catalyst the reaction yield was lowered up to 71% and 74% (entries 8 and 9, respectively). Additionally, heating the reaction to 45 °C the product *Z*-**3a** was formed in 60 % yield, which could be explained by the low stability of the boryl-copper intermediates in the media.

Table S2. Effect of Cu/L in methylboration of 1a.

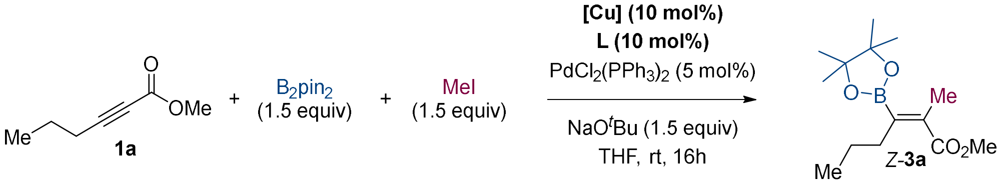

Entry	[Cu]	L	yield (%)[a]
1	CuCl	PCy_3	84
2	CuCl	XantPhos	81
3	CuCl	DPEPhos	74
4	CuCl	PPh_3	77
5	CuCl	BINAP	52
6	*i*PrCuCl	-	69
7	-	-	Complex mixture[b]
8[c]	CuCl	PCy_3	71
9[d]	CuCl	PCy_3	74
10[e]	CuCl	PCy_3	60

[a]Determined in the reaction crude by ^{1}H NMR spectroscopy employing 1,3,5-trimethoxybenzene (TMB) as an internal standard. [b]The carboborylation product was not detected in the reaction crude by ^{1}H NMR spectroscopy. [c]Using 5 mol % of CuCl/PCy_3. [d]Using a 2.5 mol% of the Pd catalyst. [e]Reaction performed at 45 ºC.

With the CuCl/PCy_3 system as an optimal combination for the carboboration process, we then moved to the study of other Pd-complexes in order to determine their effect in the synthesis of the tetrasubstituted boronate *Z*-**3a** (Table S3). In this sense, the addition of either $PdCl_2$(dppp) or [PdCl(allyl)]$_2$ as surrogates for the $PdCl_2(PPh_3)_2$ complex led to a comparable effectiveness (entries 2 and 3, respectively). The use of the more electron-rich [^{i}PrPdCl$_2$]$_2$ complex slightly decreases the reaction yield to 59 % (entry 4). Similar efficiency was observed by using 3rd generation Buchwald Pd-precatalysts such as PCy_3 Pd G3 and PPh_3 Pd G3 (entries 5 and 6). Conversely, the employment of the RuPhos Pd G3 delivered the carboboration product with 84% yield (entry 7), a similar result to that obtained with $PdCl_2(PPh_3)_2$ (entry 1). Interestingly, when $Pd(OAc)_2$ was added in combination with an additional amount of PCy_3 (10 mol%) the overall yield determined in the reaction crude was 91 % (entry 8). With these results in our hands, we conclude that the optimized conditions consist of the addition of CuCl (10 mol%) and $Pd(OAc)_2$ (5 mol%) with a 20 mol% of PCy_3 as a ligand for both metallic systems.

Table S3. Effect of Pd/L in methylboration of 1a.

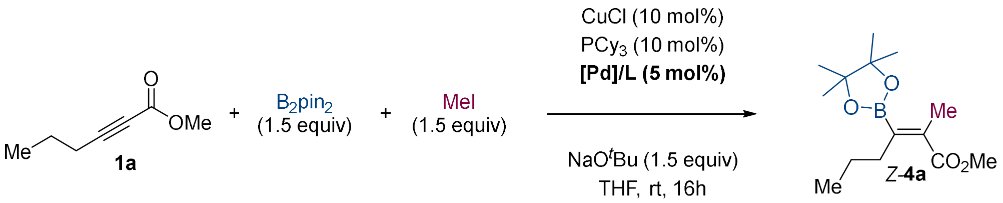

Entry	[Pd]	L	yield (%)[a]
1	$PdCl_2(PPh_3)_2$	-	84
2	$PdCl_2(dppp)_2$	-	81
3	$[PdCl(allyl)]_2$	-	83
4	$[^iPrPdCl_2]_2$	-	69
5	PCy_3 Pd G3	-	57
6	PPh_3 Pd G3	-	64
7	RuPhos Pd G3	-	84
8	$Pd(OAc)_2$	PCy_3 (10 mol%)	91

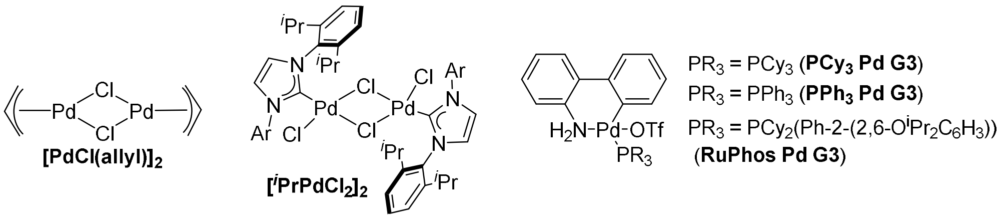

[a]Determined in the reaction crude by ^1H NMR spectroscopy employing 1,3,5-trimethoxybenzene (TMB) as an internal standard. dppp = 1,3-bis/diphenylphosphino)propane.

3.1.2 Optimization studies performed on substrate 1a: benzylboration reaction

The potential of other electrophiles to be engaged in the carboboration process was then studied, with benzyl bromide as the model substrate (Table S4). Surprisingly, when we submitted the alkyne **1a** to the above-mentioned optimized reaction conditions with BnBr (1.5 equiv) as the electrophile we obtained a low 47% yield of the corresponding carboborylated product *Z*-**4a** (entry 1), albeit with complete *syn*-stereoselectivity (> 98:2). This result highlights the exceptional dependence of the Pd/Cu catalytic system with the electrophile used. Encouraged by this result, we studied other ligands and Pd complexes that would be active in increasing the amount of the carboboration product. Changing the $Pd(OAc)_2$ by the $PdCl_2(PPh_3)_2$ species an increased reaction yield was obtained up to 62% (entry 2). For this reason, we continued the optimization studies with this Pd complex. When BINAP was used instead of the PCy_3 phosphine as a ligand for the copper salt a lower yield was obtained (45%, entry 3). However, using SPhos the benzyl borylated product was obtained in 80 % yield (entry 4). An even better result gave the XantPhos ligand, which furnished the desired product with a very high reaction yield of 94% (entry 5). Other bisphosphine ligands rendered the product *Z*-**4a** with lower yields while maintaining the high levels of *syn*-stereoselectivity (entries 6, 7, and 8). Therefore, we concluded that a new catalyst set was necessary for the use of benzylic electrophiles as suitable partners in the carboboration reaction, as shown in entry 5. Finally, the use of $PdCl_2$ in combination with XantPhos led to a 71% (entry 9).

Table S4. Optimization for benzylboration of 1a.

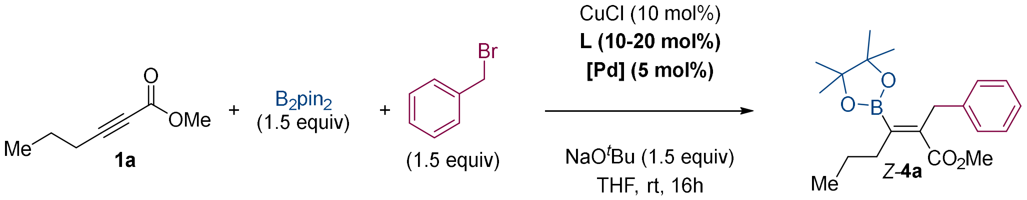

Entry	[Pd]	L	yield (%)^a
1	$Pd(OAc)_2$	PCy_3 (20 mol%)	47
2	$PdCl_2(PPh_3)_2$	PCy_3 (10 mol%)	62
3	$PdCl_2(PPh_3)_2$	BINAP (10 mol%)	45
4	$PdCl_2(PPh_3)_2$	SPhos (10 mol%)	80
5	$PdCl_2(PPh_3)_2$	XantPhos (10 mol%)	94
6	$PdCl_2(PPh_3)_2$	DPEPhos (10 mol%)	50
7	$PdCl_2(PPh_3)_2$	dppf (10 mol%)	33
8	$PdCl_2(PPh_3)_2$	dppbz	25
9	$PdCl_2$	XantPhos	71

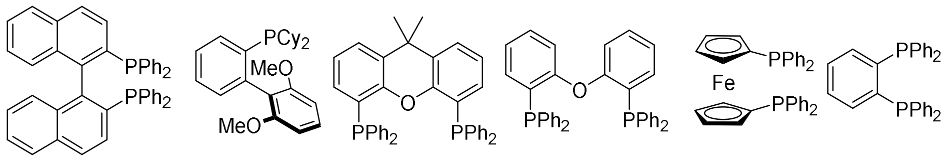

BINAP **SPhos** **XantPhos** **DPEPhos** **dppf** **dppbz**

^{a}Determined in the reaction crude by ^{1}H NMR spectroscopy employing 1,3,5-trimethoxybenzene (TMB) as an internal standard. Abbreviations: dppp = 1,3-bis(diphenylphosphino)propane, dppf = 1,1'-bis(diphenylphosphino)ferrocene.

3.1.3 Optimization studies performed on substrate 1a: arylboration reaction

Intrigued by the observed catalyst set/electrophile dependence we decided to study aryl halides and their congeners as potential substrates for the carboboration of substrate **1a** (Table S5). When the CuCl/$Pd(OAc)_2$/PCy_3 pre-catalyst system was studied in combination with PhI as the electrophile a low yield of 12 % of the *syn*-carboborylated product *Z*-**5a** was obtained (entry 1). This reaction yield was increased to 26 % when $PdCl_2(PPh_3)_2$ was employed as the Pd source and the CuCl/XantPhos system (entry 2). Following the optimization study using this palladium complex, we still observed low yields in CH_3CN and CH_2Cl_2 as solvents (15% and 36% yield, entries 4 and 5, respectively). Conversely, the use of toluene delivered the desired product in a decent 54 % yield (entry 4). With this finding in our hands, we explored other palladium pre-catalysts using toluene as the solvent at room temperature and the CuCl/XantPhos combination (10 mol%). To our delight, the use of the dimer $[PdCl(allyl)]_2$ delivered the carboborylation product with an exceptional yield (83%, entry 6), which was further increased to almost quantitative yield by using $Pd_2(dba)_3{\cdot}CHCl_3$ as the pre-catalyst (96% yield, entry 7). Other complexes such as $PdCl_2$(dppp) and $PdCl_2$(dppf) gave also good results, albeit with lower yields (entries 8 and 9). The use of 3rd generation Buchwald-pre-catalysts delivered the desired product with high yields (74% and 87%) although they did not perform better than the $Pd_2(dba)_3{\cdot}CHCl_3$ complex (entries 10 and 11, respectively). Finally, the use of $Pd(acac)_2$ yielded the product *Z*-**5a** in a 55 % yield. Having identified the optimal Pd complex ($Pd_2(dba)_3{\cdot}CHCl_3$) we studied different modifications of the reaction system. For instance, when an additional amount of XantPhos was added (a total quantity of 20 mol%), a depletion in the reaction efficacy was observed with the carboborylated product *Z*-**5a** obtained in 21% yield (entry 13). This result points out the importance of the metal/ligand speciation in the reaction cocktail. When the PhI was replaced by PhBr under the optimized reaction conditions a 53% yield was obtained, highlighting the importance of the nature of the C(sp^2)—X bond in the process (entry 14). This

hypothesis was further corroborated by employing PhOTf as electrophile species, which did not afford any detectable carboborylated product in the reaction crude (entry 15).

Table S5. Optimization studies for arylboration of 1a.

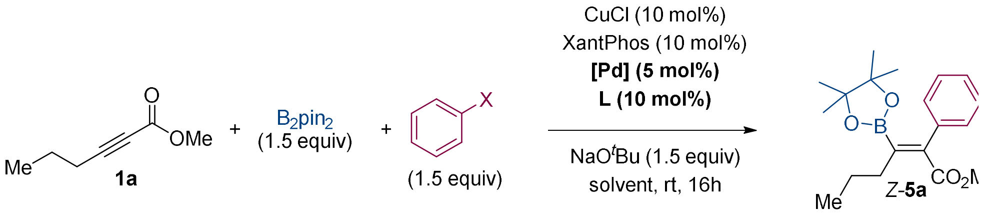

Entry	[Pd]	L	ArX	solvent	yield (%)[a]
1[b]	$Pd(OAc)_2$	PCy_3 (20 mol%)	PhI	THF	12
2	$PdCl_2(PPh_3)_2$	-	PhI	THF	26
3	$PdCl_2(PPh_3)_2$	-	PhI	CH_3CN	< 15 %
4	$PdCl_2(PPh_3)_2$	-	PhI	toluene	54
5	$PdCl_2(PPh_3)_2$	-	PhI	CH_2Cl_2	36
6	$[PdCl(allyl)]_2$	-	PhI	toluene	83
7	$Pd_2(dba)_3 \cdot CHCl_3$	-	PhI	toluene	96
8	$PdCl_2(dppp)$	-	PhI	toluene	56
9	$PdCl_2(dppf)$	-	PhI	toluene	72
10	PPh_3 Pd G3		PhI	toluene	74
11	PCy_3 Pd G3		PhI	toluene	87
12	$Pd(acac)_2$	-	PhI	toluene	55
13	$Pd_2(dba)_3 \cdot CHCl_3$	XantPhos (10 mol%)	PhI	toluene	21
14	$Pd_2(dba)_3 \cdot CHCl_3$		PhBr	toluene	53
15	$Pd_2(dba)_3 \cdot CHCl_3$		PhOTf	toluene	-

[a]Determined in the reaction crude by ^1H NMR spectroscopy employing 1,3,5-trimethoxybenzene (TMB) as an internal standard. [b]Reaction performed in the absence of XantPhos. Abbreviations: dba = dibenzylidenacetone, dppp = 1,3-bis(diphenylphosphino)propane, dppf = 1,1'-bis(diphenylphosphino)ferrocene.

3.1.4 Unsuccessful electrophiles

Besides, we explored other potential electrophiles under similar conditions employing the Cu/Pd combination to expand the chemical space available by using this approach. However, we observed a lack of reactivity or non-selective transformation of a different array of electrophile partners (Scheme S1). Initially, we screened other alkyl electrophiles more than MeI. This study includes the exploration of another primary (BuI and BuBr), secondary (^{i}PrI, ^{sec}BuI, CyI, CyBr), and tertiary (^{t}BuI, ^{t}BuBr) electrophiles. A ligand screening including different monodentate, bidentate, and NHC-based ligands was performed in presence of the model substrate **1a**. These ligands have been employed in other Pd-catalyzed transformations using alkyl electrophiles because of their highly sterically demanding properties with very good σ-donor character, which reduces the tendency of the intermediate alkyl-Pd species to undergo β-hydride elimination. However, we could not detect any product derived from the carboboration of **1a** by analysis of the ^1H NMR spectrum of the reaction crude. The same result was observed when fluorinated alkyl iodides were used, albeit in this case the formation of radicals is expected which are less prone to react with the intermediate alkenyl-copper intermediates. Finally, allyl electrophiles containing bromide, acetate, and carbonate leaving groups were tested. In this case, we did not observe the desired carboboration product, but detectable amounts of the product from the S_N2' reaction between the allyl electrophile and the NaO^{t}Bu were observed in the reaction crude by ^1H NMR

spectroscopy. In the case of acetate- and carbonate-containing allylic electrophiles the use of substoichiometric amounts of base did not afford the desired product.

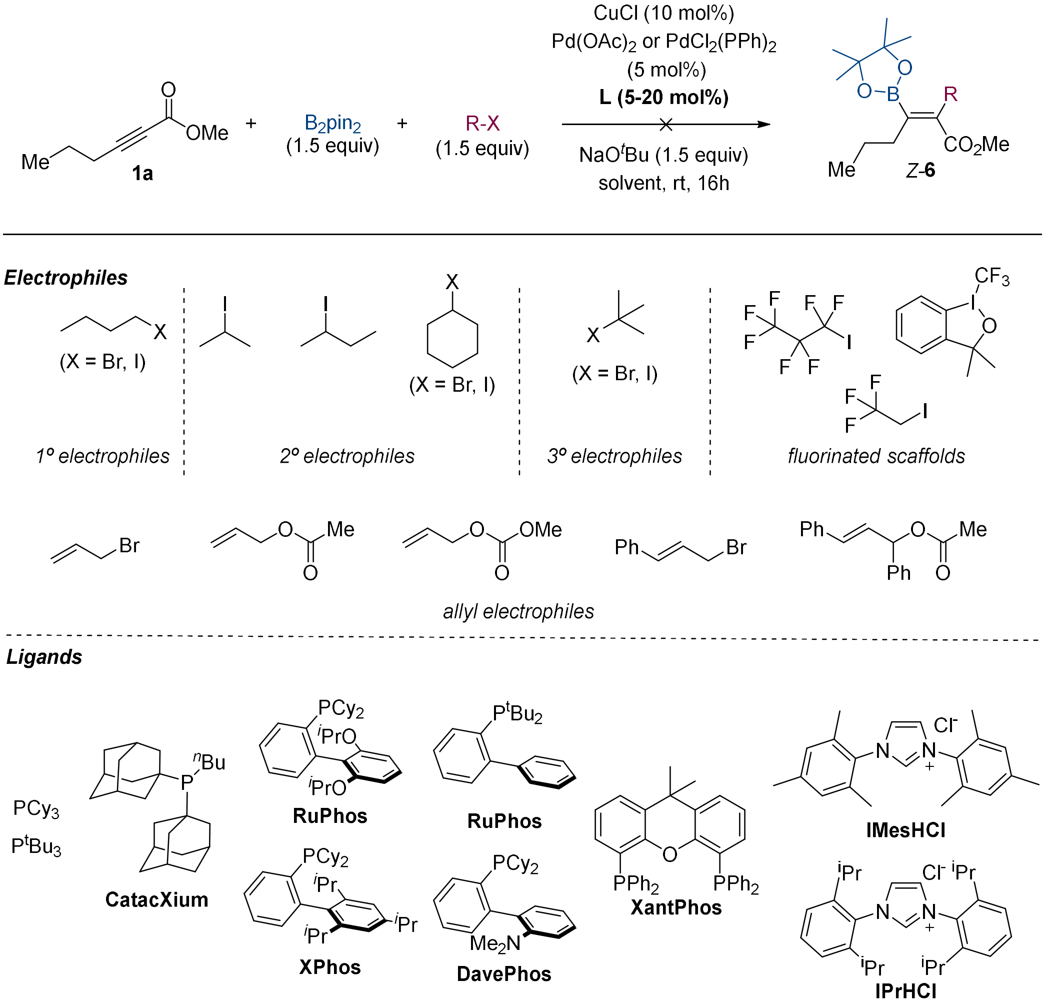

Scheme S1. Non-reactive electrophiles.

3.2 Cu/Pd-catalyzed *syn*-carboboration

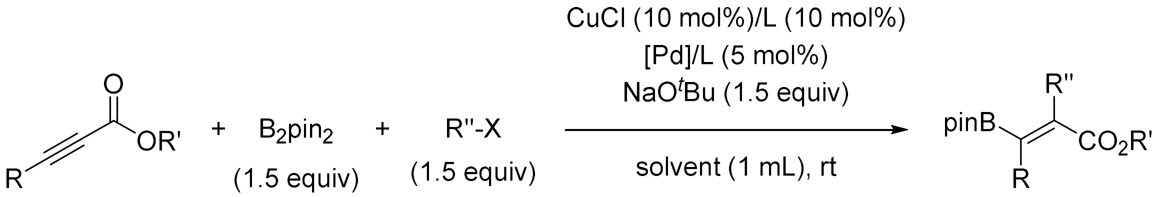

General procedure: in an oven-dried scintillation vial provided with a magnetic stir bar the B_2pin_2 (38 mg, 0.15 mmol, 1.5 equiv), CuCl (1 mg, 0.01 mmol, 10 mol%), the ligand and the palladium source (5 mol % [Pd]) were added, and the vial was closed with a septum. Then, under an Ar atmosphere, NaO^{t}Bu (2M 75 µL, 1.5 mmol, 1.5 equiv), alkyne (0.1 mmol, 1.0 equiv), and the electrophile (0.15 mmol, 1.5 equiv) were added in this order, and the reaction was allowed to stir for overnight. After that, the resulting solution was quenched with NH_4Cl (sat) (10 mL) and diluted with AcOEt (10 mL). The aqueous phase was then extracted with AcOEt (3x 10 mL) and the combined organic phases were washed with brine (20 mL), dried with Na_2SO_4 and filtered off. Finally, the solvent was removed from vacuo and the resulting residue was further purified by column chromatography.

Methyl (*Z*)-2-methyl-3-(4,4,5,5-tetramethyl-1,3,2-dioxaborolan-2-yl)hex-2-enoate (*Z*-3a):

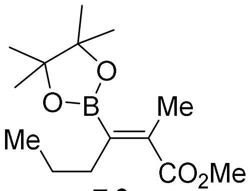

Z-**3a**

Following the general procedure for the *syn*-carboboration reaction, the treatment of alkyne **1a** (13.5 μL, 0.1 mmol, 1.0 equiv) in presence of CuCl (1 mg, 0.01 mmol, 10 mol%), $Pd(OAc)_2$ (1.12 mg, 0.005 mmol, 5 mol%), PCy_3 (37 μL, 20% in toluene, min. 88%, 20 μmol), MeI (9.3 μL, 0.15 mmol, 1.5 equiv) in THF (1.0 mL) yielded the titled compound after purification by flash column chromatography (n-hexane:toluene (3:1) to n-hexane:AcOEt (10:1)) as a colorless oil (23.3 mg, 87%). The reaction delivered the titled compound in 89% yield at 2 mmol scale. **^{1}H-NMR** (300 MHz, $CDCl_3$): δ 3.72 (s, 3H), 2.24 (t, J = 7.6 Hz, 2H), 2.06 (s, 3H), 1.44-1.32 (m, 2H), 1.28 (s, 12H), 0.88 (t, J = 7.3 Hz, 3H). **^{13}C-NMR** (75 MHz, $CDCl_3$): δ 170.6, 138.5, 83.7, 51.4, 35.1, 24.9, 23.2, 19.4, 14.3. **^{11}B NMR** (96 MHz, $CDCl_3$) δ 30.7. **HRMS** (ESI+) m/z [M + H]+ calcd for $C_{14}H_{26}BO_4$ 269.1919, found 269.1915.

Methyl (*Z*)-3-cyclohexyl-2-methyl-3-(4,4,5,5-tetramethyl-1,3,2-dioxaborolan-2-yl)acrylate (*Z*-3b):

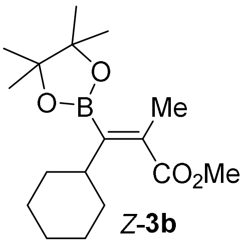

Z-**3b**

Following the general procedure for the *syn*-carboboration reaction, the treatment of alkyne **1b** (16.6 mg, 0.1 mmol, 1.0 equiv) in presence of CuCl (1 mg, 0.01 mmol, 10 mol%), $Pd(OAc)_2$ (1.12 mg, 0.005 mmol, 5 mol%), PCy_3 (37 μL, 20% in toluene, min. 88%, 20 μmol), MeI (9.3 μL, 0.15 mmol, 1.5 equiv) in THF (1.0 mL) yielded the titled compound after purification by flash column chromatography (n-hexane:toluene (3:1) to n-hexane:AcOEt (10:1)) as a colorless oil (23.7 mg, 77%). **^{1}H-NMR** (300 MHz, $CDCl_3$): δ 3.70 (s, 3H), 2.65 – 2.50 (m, 1H), 1.94 (s, 3H), 1.77 – 1.56 (m, 6H), 1.31 (s, 12H), 1.26 – 1.06 (m, 4H). **^{13}C-NMR** (75 MHz, $CDCl_3$): δ 170.2, 133.3, 83.9, 51.3, 42.7, 32.5, 26.5, 26.2, 25.1, 19.9. **HRMS** (APCI+) m/z [M+H]+ calcd for $C_{17}H_{30}BO_4$ 309.2232, found 309.2232.

Cycloheptyl (*Z*)-2-methyl-3-(4,4,5,5-tetramethyl-1,3,2-dioxaborolan-2-yl)hex-2-enoate (*Z*-3c):

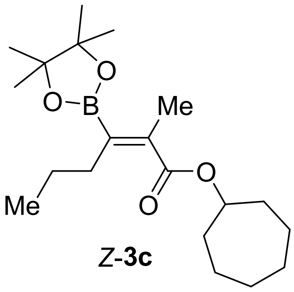

Z-**3c**

Following the general procedure for the *syn*-carboboration reaction, the treatment of alkyne **1c** (20.8 mg, 0.1 mmol, 1.0 equiv) in presence of CuCl (1 mg, 0.01 mmol, 10 mol%), $Pd(OAc)_2$ (1.12 mg, 0.005 mmol, 5 mol%), PCy_3 (37 μL, 20% in toluene, min. 88%, 20 μmol), MeI (9.3 μL, 0.15 mmol, 1.5 equiv) in THF (1.0 mL) yielded the titled compound after purification by flash column chromatography (n-hexane:toluene (3:1) to n-hexane:AcOEt (10:1)) as a colorless oil (31.9 mg, 91%). **^{1}H-NMR** (300 MHz, $CDCl_3$): δ 5.00 (tt, J = 8.3, 4.5 Hz, 1H), 2.22 (t, J = 7.4 Hz, 2H), 2.05 (s, 3H), 1.99-1.85 (m, 3H), 1.75-1.60 (m, 4H), 1.58 – 1.54 (m, 3H), 1.48 – 1.34 (m, 4H), 1.27 (s, 12H), 0.88 (t, J = 7.3 Hz, 3H). **^{13}C-NMR** (75 MHz, $CDCl_3$): δ 167.0, 140.0, 83.6, 75.2, 34.9, 34.0, 28.4, 24.9, 23.4, 23.0, 19.4, 14.4. **HRMS** (APCI+) m/z [M+H]+ calcd for $C_{20}H_{36}BO_4$ 351.2701, found 351.2711.

Methyl (*Z*)-7-chloro-2-methyl-3-(4,4,5,5-tetramethyl-1,3,2-dioxaborolan-2-yl)hept-2-enoate (*Z*-3d):

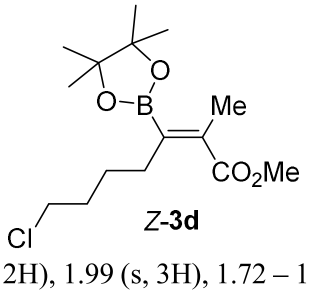

Z-**3d**

Following the general procedure for the *syn*-carboboration reaction, the treatment of alkyne **1d** (17.5 mg, 0.1 mmol, 1.0 equiv) in presence of CuCl (1 mg, 0.01 mmol, 10 mol%), $Pd(OAc)_2$ (1.12 mg, 0.005 mmol, 5 mol%), PCy_3 (37 μL, 20% in toluene, min. 88%, 20 μmol), MeI (9.3 μL, 0.15 mmol, 1.5 equiv) in THF (1.0 mL) yielded the titled compound after purification by flash column chromatography (n-hexane:toluene (3:1) to n-hexane:AcOEt (10:1)) as a colorless oil (21.5 mg, 68%). **^{1}H-NMR** (300 MHz, $CDCl_3$): δ 3.65 (s, 3H), 3.44 (t, J = 6.7 Hz, 2H), 2.22 (t, J = 7.4 Hz, 2H), 1.99 (s, 3H), 1.72 – 1.64 (m, 2H), 1.48 – 1.41 (m, 2H), 1.21 (s, 12H). **^{13}C-NMR** (75 MHz, $CDCl_3$):

δ 170.4, 139.3, 83.8, 51.5, 45.1, 32.6, 32.0, 27.1, 24.9, 19.4. **HRMS** (APCI+) m/z $[M+H]^+$ calcd for $C_{15}H_{27}BClO_4$ 317.1685, found 317.1685.

Methyl (*Z*)-6-cyano-2-methyl-3-(4,4,5,5-tetramethyl-1,3,2-dioxaborolan-2-yl)hex-2-enoate (*Z*-3e):

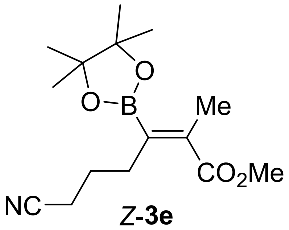

Z-**3e**

Following the general procedure for the *syn*-carboboration reaction, the treatment of alkyne **1e** (15.1 mg, 0.1 mmol, 1.0 equiv) in presence of CuCl (1 mg, 0.01 mmol, 10 mol%), $Pd(OAc)_2$ (1.12 mg, 0.005 mmol, 5 mol%), PCy_3 (37 µL, 20% in toluene, min. 88%, 20 µmol), MeI (9.3 µL, 0.15 mmol, 1.5 equiv) in THF (1.0 mL) yielded the titled compound after purification by flash column chromatography (n-hexane:toluene (3:1) to n-hexane:AcOEt (10:1)) as a colorless oil (25.2 mg, 86%). **^{1}H-NMR** (300 MHz, $CDCl_3$): δ 3.75 (s, 3H), 2.40-2.26 (m, 4H), 2.10 (s, 3H), 1.77 (p, J = 7.3 Hz, 2H), 1.28 (s, 12H). **^{13}C-NMR** (75 MHz, $CDCl_3$): δ 170.2, 141.5, 120.1, 83.9, 51.7, 31.8, 25.7, 24.9, 19.4, 16.9. **HRMS** (ESI+) m/z $[M+H]^+$ calcd for $C_{15}H_{25}BNO_4$ 294.1871, found 294.1869.

(*R*)-2,5,7,8-tetramethyl-2-((*3R,7R*)-3,7,11-trimethyldodecyl)chroman-6-yl (*Z*)-2-methyl-3-(4,4,5,5-tetramethyl-1,3,2-dioxaborolan-2-yl)hex-2-enoate (*Z*-3f):

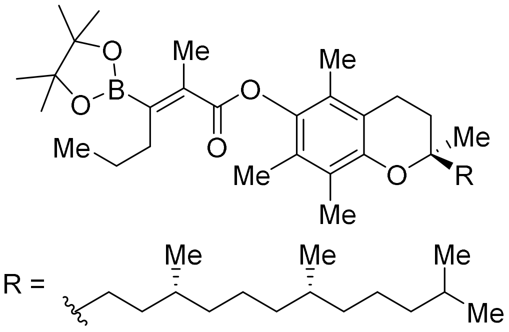

Z-**3f**

Following the general procedure for the *syn*-carboboration reaction, the treatment of alkyne **1g** (51 mg, 0.1 mmol, 1.0 equiv) in presence of CuCl (1 mg, 0.01 mmol, 10 mol%), $Pd(OAc)_2$ (1.12 mg, 0.005 mmol, 5 mol%), PCy_3 (37 µL, 20% in toluene, min. 88%, 20 µmol), MeI (9.3 µL, 0.15 mmol, 1.5 equiv) in THF (1.0 mL) yielded the titled compound after purification by flash column chromatography (n-hexane:toluene (3:1) to n-hexane:AcOEt (10:1)) as a white solid (45.7 mg, 70%). **^{1}H-NMR** (300 MHz, $CDCl_3$): δ 2.60 (t, J = 6.4 Hz, 3H), 2.48 (t, J = 7.5 Hz, 2H), 2.29 (s, 3H), 2.10 (s, 3H), 2.04 (s, 3H), 2.00 (s, 3H), 1.90 – 1.69 (m, 7H), 1.60-1.40 (m, 8H), 1.33 (s, 12H), 1.29-1.23 (m, 9H), 0.92 (t, J = 7.3 Hz, 3H), 0.87 (m, 15H). **^{13}C-NMR** (75 MHz, $CDCl_3$): δ 167.7, 149.5, 140.6, 136.8, 126.9, 125.1, 123.2, 117.5, 83.9, 77.4, 75.1, 39.5, 37.7, 37.7 (2C), 37.5, 37.4, 35.0, 33.0, 32.9, 32.8, 28.1, 25.0, 24.6, 23.4, 22.9, 22.8, 21.2, 208, 19.9, 19.8, 19.7, 14.4, 13.3, 12.4, 12.0. **HRMS** (APCI+) m/z [M+H]+ calcd for $C_{42}H_{72}BO_5$ 667.5467, found 667.5482.

(*8R,9S,13S,14S*)-13-methyl-17-oxo-7,8,9,11,12,13,14,15,16,17-decahydro-6*H*-cyclopenta[*a*]phenanthren-3-yl (*Z*)-2-methyl-3-(4,4,5,5-tetramethyl-1,3,2-dioxaborolan-2-yl)hex-2-enoate (*Z*-3g):

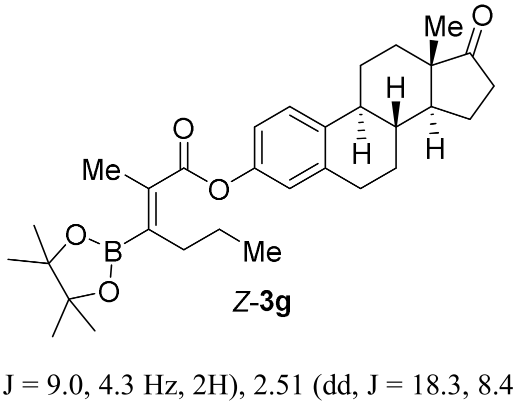

Z-**3g**

Following the general procedure for the *syn*-carboboration reaction, the treatment of alkyne **1f** (36.4 mg, 0.1 mmol, 1.0 equiv) in presence of CuCl (1 mg, 0.01 mmol, 10 mol%), $Pd(OAc)_2$ (1.12 mg, 0.005 mmol, 5 mol%), PCy_3 (37 µL, 20% in toluene, min. 88%, 20 µmol), MeI (9.3 µL, 0.15 mmol, 1.5 equiv) in THF (1.0 mL) yielded the titled compound after purification by flash column chromatography (n-hexane:toluene (3:1) to n-hexane:AcOEt (10:1)) as a white solid (41 mg, 81%). **M.p.:** 112-115 °C.**^{1}H-NMR** (300 MHz, $CDCl_3$): δ 7.29 (d, J = 8.6 Hz, 1H), 6.87 (d, J = 8.6 Hz, 1H), 6.83 (bs, 1H), 2.92 (dd, J = 9.0, 4.3 Hz, 2H), 2.51 (dd, J = 18.3, 8.4 Hz, 1H), 2.40 (t, J = 7.6 Hz, 2H), 2.21 (s, 3H), 2.17 – 1.95 (m, 4H), 1.71 – 1.36 (m, 10H), 1.31 (s, 12H), 0.97 – 0.87 (m, 6H). **^{13}C-NMR** (75 MHz, $CDCl_3$): δ 220.9, 168.4, 148.7, 138.1, 137.4, 137.3, 126.5, 121.8, 119.0, 83.9, 50.6, 48.1, 44.3, 38.2, 36.0, 35.1, 31.7, 29.6,

26.5, 25.9, 24.9, 23.2, 21.7, 19.5, 14.4, 14.0. **HRMS** (APCI+) m/z [M]+ calcd for $C_{31}H_{44}BO_5$ 507.3276, found 507.3293.

Methyl (*Z*)-2-benzyl-3-(4,4,5,5-tetramethyl-1,3,2-dioxaborolan-2-yl)hex-2-enoate (*Z*-4a):

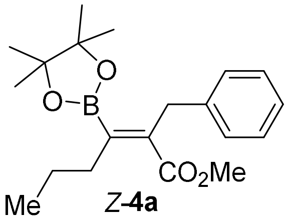
Z-**4a**

Following the general procedure for the *syn*-carboboration reaction, the treatment of alkyne **1a** (13.5 µL, 0.1 mmol, 1.0 equiv) in presence of CuCl (1 mg, 0.01 mmol, 10 mol%), $(PPh_3)_2PdCl_2$ (3.5 mg, 0.005 mmol, 5 mol%), XantPhos (5.78 mg, 0.01 mmol, 10 mol%), and benzyl bromide (17.8 µL, 0.15 mmol, 1.5 equiv) in THF (1.0 mL) yielded the titled compound after purification by flash column chromatography (n-hexane:toluene:AcOEt (10:10:1)) as an oil (29.6 mg, 86%). The reaction delivered the titled compound in 90% yield at 2 mmol scale. **^{1}H-NMR** (300 MHz, $CDCl_3$): δ 7.40 – 7.09 (m, 5H), 3.92 (s, 2H), 3.63 (s, 3H), 2.38 (t, J = 7.6 Hz, 3H), 1.50 (h, J = 7.6 Hz, 2H), 1.33 (s, 12 H), 0.96 (td, J = 7.3, 1.7 Hz, 3H). **^{13}C-NMR** (75 MHz, $CDCl_3$): δ 169.6, 141.9, 139.5, 128.8, 128.3, 126.1, 83.9, 51.2, 39.4, 35.0, 24.9, 23.3, 14.3.**^{11}B NMR** (96 MHz, $CDCl_3$) δ 30.4. **HRMS** (TOF MS ES+) m/z [M]+ calcd for $C_{20}H_{29}BO_4$ 344.2159, found 344.2152.

Methyl (*Z*)-2-(2-bromobenzyl)-3-(4,4,5,5-tetramethyl-1,3,2-dioxaborolan-2-yl)hex-2-enoate (*Z*-4b):

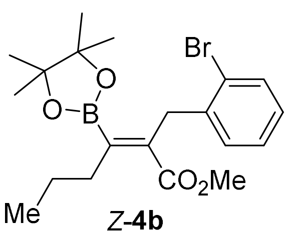
Z-**4b**

Following the general procedure for the *syn*-carboboration reaction, the treatment of alkyne **1a** (13.5 µL, 0.1 mmol, 1.0 equiv) in presence of CuCl (1 mg, 0.01 mmol, 10 mol%), $(PPh_3)_2PdCl_2$ (3.5 mg, 0.005 mmol, 5 mol%), XantPhos (5.78 mg, 0.01 mmol, 10 mol%), and 2-bromobenzyl bromide (37.5 mg, 0.15 mmol, 1.5 equiv) in THF (1.0 mL) yielded the titled compound after purification by flash column chromatography (n-hexane:toluene:AcOEt (10:10:1)) as an oil (27 mg, 64%).**^{1}H-NMR** (300 MHz, $CDCl_3$): δ 7.50 (d, J = 7.8 Hz, 1H), 7.24 – 7.18 (m, 2H), 7.05 – 6.98 (m, 1H), 4.00 (s, 2H), 3.59 (s, 3H), 2.37 (t, J = 7.7 Hz, 2H), 1.52 – 1.43 (m, 2H), 1.21 (s, 12H), 0.93 (t, J = 7.3 Hz, 3H). **^{13}C-NMR** (75 MHz, $CDCl_3$): δ 169.4, 140.2, 139.3, 132.5, 130.8, 130.0, 127.4, 126.8, 83.9, 51.4, 39.0, 35.1, 24.9, 23.3, 14.3. **HRMS** (ESI +) m/z $[M+H]^+$ calcd for $C_{20}H_{29}BBrO_4$ 423.1337, found 423.1341.

Methyl (Z)-2-(4-bromobenzyl)-3-(4,4,5,5-tetramethyl-1,3,2-dioxaborolan-2-yl)hex-2-enoate (*Z*-4c):

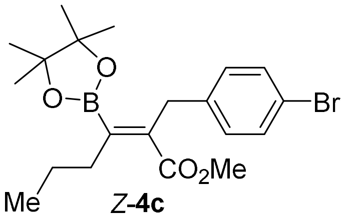
Z-**4c**

Following the general procedure for the *syn*-carboboration reaction, the treatment of alkyne **1a** (13.5 µL, 0.1 mmol, 1.0 equiv) in presence of CuCl (1 mg, 0.01 mmol, 10 mol%), $(PPh_3)_2PdCl_2$ (3.5 mg, 0.005 mmol, 5 mol%), XantPhos (5.78 mg, 0.01 mmol, 10 mol%), and 4-bromobenzyl bromide (37.5 mg, 0.15 mmol, 1.5 equiv) in THF (1.0 mL) yielded the titled compound after purification by flash column chromatography (n-hexane:toluene:AcOEt (10:10:1)) as an oil (30 mg, 71%). **^{1}H-NMR** (300 MHz, $CDCl_3$): δ 7.36 (d, J = 8.4 Hz, 2H), 7.09 (d, J = 8.4 Hz, 2H), 3.80 (s, 2H), 3.59 (s, 3H), 2.35 – 2.26 (m, 2H), 1.51 – 1.39 (m, 2H), 1.27 (s, 12H), 0.90 (t, J = 7.4 Hz, 3H). **^{13}C-NMR** (75 MHz, $CDCl_3$): δ 169.3, 141.1, 138.6, 131.4, 130.6, 120.1, 84.0, 51.3, 38.8, 35.1, 24.9, 23.3, 14.3. **HRMS** (ESI +) m/z $[M+H]^+$ calcd for $C_{20}H_{29}BBrO_4$ 423.1337, found 423.1332.

Methyl (Z)-2-(2,3-difluorobenzyl)-3-(4,4,5,5-tetramethyl-1,3,2-dioxaborolan-2-yl)hex-2-enoate (*Z*-4d):

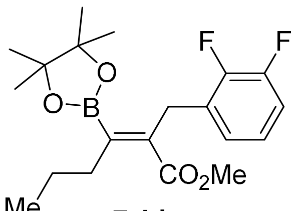

Z-**4d**

Following the general procedure for the *syn*-carboboration reaction, the treatment of alkyne **1a** (13.5 µL, 0.1 mmol, 1.0 equiv) in presence of CuCl (1 mg, 0.01 mmol, 10 mol%), $(PPh_3)_2PdCl_2$ (3.5 mg, 0.005 mmol, 5 mol%), XantPhos (5.78 mg, 0.01 mmol, 10 mol%), and 1-(bromomethyl)-2,3-difluorobenzene (18.6 µL, 0.15 mmol, 1.5 equiv) in THF (1.0 mL) yielded the titled compound after purification by flash column chromatography (n-hexane:toluene:AcOEt (10:10:1)) as an oil (28.9 mg, 76%). **^{1}H-NMR** (300 MHz, $CDCl_3$): δ 7.12 – 6.84 (m, 3H), 3.95 (s, 2H), 3.62 (s, 3H), 2.34 (t, J = 7.7 Hz, 2H), 1.52-1.39 (m, 2H), 1.26 (s, 12H), 0.91 (t, J = 7.3 Hz, 3H). **^{13}C-NMR** (75 MHz, $CDCl_3$): δ 169.2, 151.52 (d, J = 117.4 Hz), 148.5 (d, J = 81.4 Hz), 139.9, 129.4 (d, J = 12.4 Hz), 125.2 (t, J = 3.3 Hz), 123.7 (dd, J = 6.8, 4.8 Hz), 115.0 (d, J = 17.1 Hz), 84.0, 51.4, 35.1, 31.6, 24.9, 23.4, 14.3. **HRMS** (ESI^+) m/z $[M+H]^+$ calcd for $C_{20}H_{28}BF_2O_4$ 381.2043, found 381.2042.

Methyl (*Z*)-2-(2-cyanobenzyl)-3-(4,4,5,5-tetramethyl-1,3,2-dioxaborolan-2-yl)hex-2-enoate (*Z*-4e):

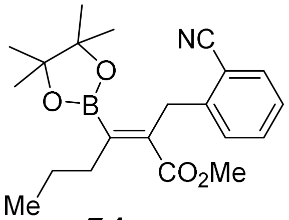

Z-**4e**

Following the general procedure for the *syn*-carboboration reaction, the treatment of alkyne **1a** (13.5 µL, 0.1 mmol, 1.0 equiv) in presence of CuCl (1 mg, 0.01 mmol, 10 mol%), $(PPh_3)_2PdCl_2$ (3.5 mg, 0.005 mmol, 5 mol%), XantPhos (5.78 mg, 0.01 mmol, 10 mol%), and 2-(bromomethyl)benzonitrile (29.4 mg, 0.15 mmol, 1.5 equiv) in THF (1.0 mL) yielded the titled compound after purification by flash column chromatography (n-hexane:toluene:AcOEt (10:10:1)) as an oil (29.5 mg, 80%). **^{1}H-NMR** (300 MHz, $CDCl_3$): δ 7.58 (d, J = 7.7 Hz, 1H), 7.47 (d, J = 7.3 Hz, 1H), 7.39 (d, J = 7.9 Hz, 1H), 7.28-7.22 (m, 1H), 4.12 (s, 2H), 3.62 (s, 3H), 2.46 – 2.35 (m, 2H), 1.53 – 1.43 (m, 2H), 1.24 (s, 12H), 0.93 (t, J = 7.3 Hz, 3H). **^{13}C-NMR** (75 MHz, $CDCl_3$): δ 168.6, 144.0, 139.3, 132.8, 132.7, 129.3, 126.6, 118.1, 112.9, 84.1, 51.5, 37.3, 35.0, 24.8, 23.3, 14.3. **HRMS** (ESI +) m/z $[M+H]^+$ calcd for $C_{21}H_{29}BNO_4$ 370.2184, found 370.2196.

Methyl (*Z*)-2-(naphthalen-1-ylmethyl)-3-(4,4,5,5-tetramethyl-1,3,2-dioxaborolan-2-yl)hex-2-enoate (*Z*-4f):

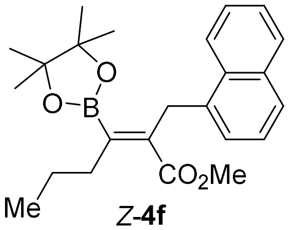

Z-**4f**

Following the general procedure for the *syn*-carboboration reaction, the treatment of alkyne **1a** (13.5 µL, 0.1 mmol, 1.0 equiv) in presence of CuCl (1 mg, 0.01 mmol, 10 mol%), $(PPh_3)_2PdCl_2$ (3.5 mg, 0.005 mmol, 5 mol%), XantPhos (5.78 mg, 0.01 mmol, 10 mol%), and 1-(bromomethyl)naphthalene (33.2 mg, 0.15 mmol, 1.5 equiv) in THF (1.0 mL) yielded the titled compound after purification by flash column chromatography (n-hexane:toluene:AcOEt (10:10:1)) as an oil (31.2 mg, 79%). **^{1}H-NMR** (300 MHz, $CDCl_3$): δ 8.27 – 8.15 (m, 1H), 7.86 – 7.79 (m, 1H), 7.69 (dd, J = 7.7, 2.1 Hz, 1H), 7.51 – 7.46 (m, 2H), 7.37 (d, J = 7.2 Hz, 2H), 4.39 (s, 2H), 3.46 (s, 3H), 2.35 (t, J = 7.3 Hz, 2H), 1.57-1.42 (m, 2H), 1.22 (s, 12H), 0.93 (t, J = 7.3 Hz, 3H). **^{13}C-NMR** (75 MHz, $CDCl_3$): δ 169.9, 142.4, 135.7, 133.8, 132.5, 128.6, 126.9, 126.3, 125.7, 125.6, 125.5, 124.1, 83.8, 51.2, 35.9, 35.2, 24.8, 23.3, 14.3. **HRMS** (ESI +) m/z $[M+H]^+$ calcd for $C_{24}H_{32}BO_4$ 395.2388, found 395.2392.

Methyl (*Z*)-3-(4,4,5,5-tetramethyl-1,3,2-dioxaborolan-2-yl)-2-(2,4,6-trimethylbenzyl)hex-2-enoate (*Z*-4g):

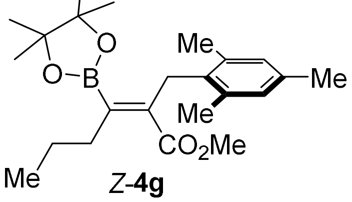

Z-**4g**

Following the general procedure for the *syn*-carboboration reaction, the treatment of alkyne **1a** (13.5 µL, 0.1 mmol, 1.0 equiv) in presence of CuCl (1 mg, 0.01 mmol, 10 mol%), $(PPh_3)_2PdCl_2$ (3.5 mg, 0.005 mmol, 5 mol%), XantPhos (5.78 mg, 0.01 mmol, 10 mol%), and 2-(bromomethyl)-1,3,5-trimethylbenzene (33.2 mg, 0.15 mmol, 1.5 equiv) in THF (1.0 mL) yielded the titled compound after purification by flash column chromatography (n-hexane:toluene:AcOEt (10:10:1)) as an oil (25.5 mg, 66%). **^{1}H-NMR** (300 MHz, $CDCl_3$): δ 6.77 (s, 2H), 3.98 (s, 2H), 3.37 (s, 3H), 2.28 (s, 6H),

2.21 (s, 3H), 2.18 – 2.06 (m, 2H), 1.48 – 1.41 (m, 2H), 1.31 (s, 12H), 0.88 (t, J = 7.3 Hz, 3H). **^{13}C-NMR** (75 MHz, $CDCl_3$): δ 170.2, 144.5, 137.6, 135.6, 131.8, 128.9, 83.6, 50.8, 35.5, 33.0, 25.0, 23.4, 21.0, 20.4, 14.3. **HRMS** (ESI +) m/z $[M+Na]^+$ calcd for $C_{23}H_{35}BNaO_4$ 409.2521, found 409.2519.

Cyclopropyl(phenyl)methyl (Z)-2-benzyl-3-(4,4,5,5-tetramethyl-1,3,2-dioxaborolan-2-yl)hex-2-enoate (*Z*-4h):

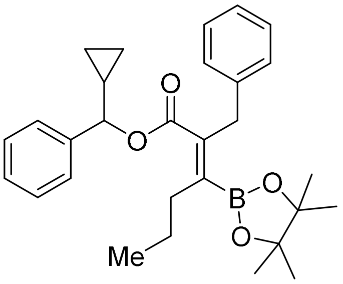

Z-**4h**

Following the general procedure for the *syn*-carboboration reaction, the treatment of alkyne **1h** (24.2 mg, 0.1 mmol, 1.0 equiv) in presence of CuCl (1 mg, 0.01 mmol, 10 mol%), $(PPh_3)_2PdCl_2$ (3.5 mg, 0.005 mmol, 5 mol%), XantPhos (5.78 mg, 0.01 mmol, 10 mol%), and benzyl bromide (17.8 µL, 0.15 mmol, 1.5 equiv) in THF (1.0 mL) yielded the titled compound after purification by flash column chromatography (n-hexane:toluene:AcOEt (10:10:1)) as an oil (37.3 mg, 81%). **^{1}H-NMR** (300 MHz, $CDCl_3$): δ 7.26 – 7.10 (m, 10H), 5.15 (d, J = 8.9 Hz, 1H), 3.93 (d, J = 15 Hz, 1H), 3.8 (d, J = 15 Hz, 1H), 2.36 – 2.22 (m, 2H), 2.17 (s, 2H), 1.51 – 1.37 (m, 2H), 1.28 (s, 12H), 0.84 (t, J = 7.3 Hz, 3H), 0.54 – 0.43 (m, 2H), 0.40 – 0.26 (m, 2H). **^{13}C-NMR** (75 MHz, $CDCl_3$): δ 168.7, 142.5, 140.2, 139.3, 129.1, 128.3, 127.7, 126.8, 126.1, 83.8, 79.9, 39.5, 34.9, 31.0, 24.9, 23.4, 16.6, 14.3, 4.3, 3.3. **HRMS** (ESI +) m/z $[M+H]^+$ calcd for $C_{29}H_{38}BO_4$ 461.2858, found 461.2864.

(*S*)-(4-(Prop-1-en-2-yl)cyclohex-1-en-1-yl)methyl (*Z*)-2-benzyl-3-(4,4,5,5-tetramethyl-1,3,2-dioxaborolan-2-yl)hex-2-enoate (*Z*-4i):

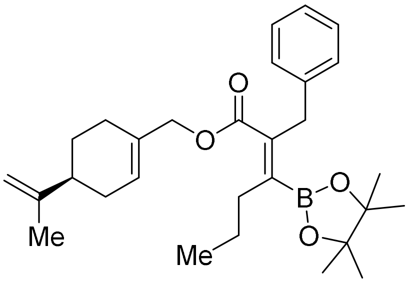

Z-**4i**

Following the general procedure for the *syn*-carboboration reaction, the treatment of alkyne **1i** (24.6 mg, 0.1 mmol, 1.0 equiv) in presence of CuCl (1 mg, 0.01 mmol, 10 mol%), $(PPh_3)_2PdCl_2$ (3.5 mg, 0.005 mmol, 5 mol%), XantPhos (5.78 mg, 0.01 mmol, 10 mol%), and benzyl bromide (17.8 µL, 0.15 mmol, 1.5 equiv) in THF (1.0 mL) yielded the titled compound after purification by flash column chromatography (n-hexane:toluene:AcOEt (10:10:1)) as an oil (33.9 mg, 73%). **^{1}H-NMR** (300 MHz, $CDCl_3$): δ 7.33 – 7.15 (m, 5H), 5.61 (s, 1H), 4.78-4.70 (m, 2H), 4.40 (s, 2H), 3.91 (s, 2H), 2.36 (t, J = 7.5 Hz, 2H), 2.25 – 1.94 (m, 3H), 2.02 – 1.80 (m, 4H), 1.76 (s, 3H), 1.56 – 1.42 (m, 2H), 1.31 (s, 12H), 0.94 (t, J = 7.3 Hz, 3H). **^{13}C-NMR** (75 MHz, $CDCl_3$): δ 169.1, 149.8, 142.1, 139.5, 132.7, 129.0, 128.3, 126.1, 125.9, 108.8, 83.8, 68.2, 40.9, 39.5, 35.1, 30.6, 27.5, 26.4, 24.9, 23.4, 20.9, 14.3. **HRMS** (ESI +) m/z $[M+Na]^+$ calcd for $C_{29}H_{41}BNaO_4$ 487.2990, found 487.2994.

But-3-en-1-yl (*Z*)-2-benzyl-3-(4,4,5,5-tetramethyl-1,3,2-dioxaborolan-2-yl)hex-2-enoate (*Z*-4j):

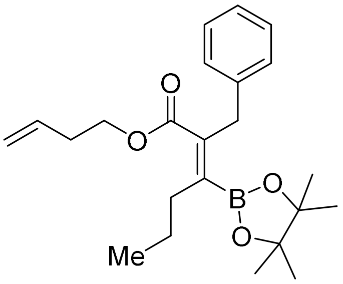

Z-**4j**

Following the general procedure for the *syn*-carboboration reaction, the treatment of alkyne **1j** (16.6 mg, 0.1 mmol, 1.0 equiv) in presence of CuCl (1 mg, 0.01 mmol, 10 mol%), $(PPh_3)_2PdCl_2$ (3.5 mg, 0.005 mmol, 5 mol%), XantPhos (5.78 mg, 0.01 mmol, 10 mol%), and benzyl bromide (17.8 µL, 0.15 mmol, 1.5 equiv) in THF (1.0 mL) yielded the titled compound after purification by flash column chromatography (n-hexane:toluene:AcOEt (10:10:1)) as an oil (35 mg, 91%). **^{1}H-NMR** (300 MHz, $CDCl_3$): δ 7.21-7.10 (m, 5H), 5.65-5.48 (m, 1H), 5.03 – 4.83 (m, 2H), 3.96 (t, J = 6.7 Hz, 2H), 3.80 (s, 2H), 2.28 – 2.15 (m, 4H), 1.44 – 1.35 (m, 2H), 1.21 (s, 12H), 0.85 (t, J = 7.3 Hz, 3H). **^{13}C-NMR** (75 MHz, $CDCl_3$): δ 169.1, 142.0, 139.5, 134.2, 128.9, 128.3, 126.1, 117.1, 83.9, 63.4, 39.4, 35.0, 33.0, 24.9, 23.3, 14.3. **HRMS** (ESI^+) m/z $[M+H]^+$ calcd for $C_{23}H_{34}BO_4$ 385.2545, found 385.2545.

5-(Trimethylsilyl)pent-4-yn-1-yl (Z)-2-benzyl-3-(4,4,5,5-tetramethyl-1,3,2-dioxaborolan-2-yl)hex-2-enoate (*Z*-4k):

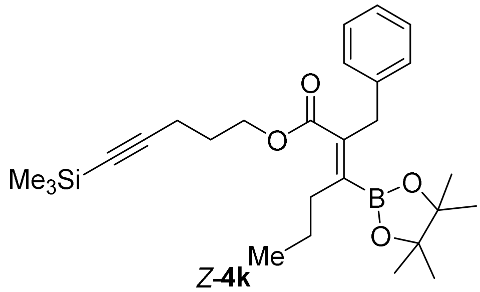

Z-**4k**

Following the general procedure for the *syn*-carboboration reaction, the treatment of alkyne **1k** (25 mg, 0.1 mmol, 1.0 equiv) in presence of CuCl (1 mg, 0.01 mmol, 10 mol%), $(PPh_3)_2PdCl_2$ (3.5 mg, 0.005 mmol, 5 mol%), XantPhos (5.78 mg, 0.01 mmol, 10 mol%), and benzyl bromide (17.8 µL, 0.15 mmol, 1.5 equiv) in THF (1.0 mL) yielded the titled compound after purification by flash column chromatography (n-hexane:toluene:AcOEt (10:10:1)) as an oil (35.1 mg, 75%). **^{1}H-NMR** (300 MHz, $CDCl_3$): δ 7.27 – 7.19 (m, 5H), 4.07 (t, J = 6.1 Hz, 2H), 3.88 (s, 2H), 2.35 – 2.30 (m, 2H), 2.09 (t, J = 7.0 Hz, 2H), 1.72 – 1.64 (m, 2H), 1.50 – 1.43 (m, 2H), 1.29 (s, 12H), 0.93 (t, J = 7.3 Hz, 3H), 0.15 (s, 9H). **^{13}C-NMR** (75 MHz, $CDCl_3$): δ 169.1, 142.2, 139.4, 128.9, 128.3, 126.2, 85.3, 83.9, 83.5, 62.7, 39.5, 35.1, 27.8, 24.8, 23.3, 16.6, 14.3, 0.2. **HRMS** (ESI^+) m/z $[M+H]^+$ calcd for $C_{27}H_{42}BO_4Si$ 469.2940, found 469.2935.

Methyl (*Z*)-2-benzyl-6-(2-(1,3-dimethyl-2,6-dioxo-1,2,3,6-tetrahydro-7*H*-purin-7-yl)acetoxy)-3-(4,4,5,5-tetramethyl-1,3,2-dioxaborolan-2-yl)hex-2-enoate (*Z*-4l):

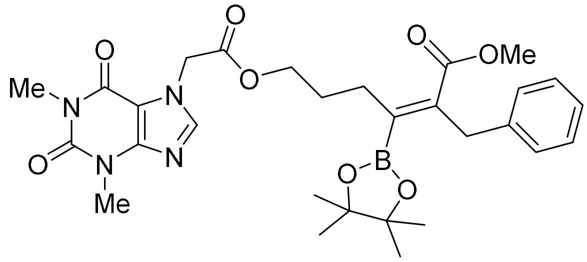

Z-**4l**

Following the general procedure for the *syn*-carboboration reaction, the treatment of alkyne **1m** (36.2 mg, 0.1 mmol, 1.0 equiv) in presence of CuCl (1 mg, 0.01 mmol, 10 mol%), $(PPh_3)_2PdCl_2$ (3.5 mg, 0.005 mmol, 5 mol%), XantPhos (5.78 mg, 0.01 mmol, 10 mol%), and benzyl bromide (17.8 µL, 0.15 mmol, 1.5 equiv) in THF (1.0 mL) yielded the titled compound after purification by flash column chromatography (n-hexane: AcOEt (3:1) to n-hexane:AcOEt:MeOH (3:1:0.1)) as an oil (37.1 mg, 64%). **^{1}H-NMR** (300 MHz, $CDCl_3$): δ 7.58 (s, 1H), 7.26 – 7.11 (m, 5H), 5.05 (s, 2H), 4.20 (t, J = 6.3 Hz, 2H), 3.89 (s, 2H), 3.59 (s, 6H), 3.37 (s, 3H), 2.38 (t, J = 7.4 Hz, 2H), 1.89 – 1.78 (m, 2H), 1.28 (s, 12H). **^{13}C-NMR** (75 MHz, $CDCl_3$): δ 169.3, 167.2, 155.4, 151.8, 148.6, 143.8, 142.2, 139.2, 128.8, 128.4, 126.3, 107.2, 84.1, 66.2, 51.4, 47.3, 39.2, 29.9, 29.3, 28.5, 28.0, 24.9. **HRMS** (ESI +) m/z $[M+H]^+$ calcd for $C_{29}H_{38}BN_4O_8$ 581.2777, found 581.2778.

Benzyl (*Z*)-3-((2-benzyl-3-(4,4,5,5-tetramethyl-1,3,2-dioxaborolan-2-yl)hex-2-enoyl)oxy)azetidine-1-carboxylate (*Z*-4m):

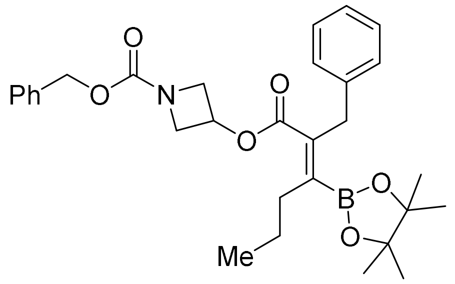

Z-**4m**

Following the general procedure for the *syn*-carboboration reaction, the treatment of alkyne **1n** (30.1 mg, 0.1 mmol, 1.0 equiv) in presence of CuCl (1 mg, 0.01 mmol, 10 mol%), $(PPh_3)_2PdCl_2$ (3.5 mg, 0.005 mmol, 5 mol%), XantPhos (5.78 mg, 0.01 mmol, 10 mol%), and benzyl bromide (17.8 µL, 0.15 mmol, 1.5 equiv) in THF (1.0 mL) yielded the titled compound after purification by flash column chromatography (n-hexane:toluene:AcOEt (5:5:1)) as an oil (49.3 mg, 95%). **^{1}H-NMR** (300 MHz, $CDCl_3$): δ 7.39 – 7.32 (m, 5H), 7.25 – 7.09 (m, 5H), 5.09 (s, 2H), 5.03 (tt, J = 6.8, 4.3 Hz, 1H), 4.18 (dd, J = 10.0, 6.8 Hz, 2H), 3.88 (bs, 2H), 3.66 (s, 2H), 2.40 – 2.27 (m, 2H), 1.53 – 1.41 (m, 2H), 1.29 (s, 12H), 0.91 (t, J = 7.3 Hz, 3H). **^{13}C-NMR** (75 MHz, $CDCl_3$): δ 167.9, 156.2, 140.4, 139.1, 136.6, 129.0, 128.6, 128.4, 128.2, 128.1, 126.4, 84.1, 66.9, 63.3, 44.4, 39.5, 35.9, 24.9, 23.2, 14.3. **HRMS** (ESI +) m/z $[M+H]^+$ calcd for $C_{30}H_{39}BNO_6$ 520.2865, found 520.2863.

Methyl (*Z*)-2-benzyl-6-(2-(1-(4-chlorobenzoyl)-5-methoxy-2-methyl-1*H*-indol-3-yl)acetoxy)-3-(4,4,5,5-tetramethyl-1,3,2-dioxaborolan-2-yl)hex-2-enoate (*Z*-4n):

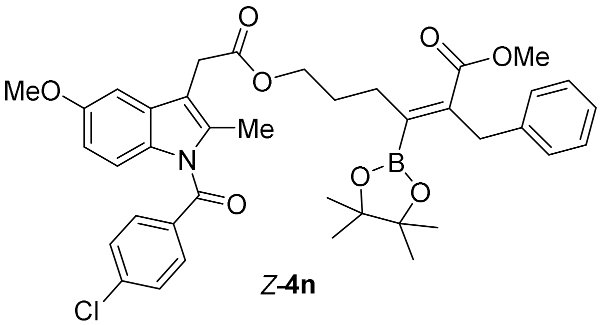

Z-**4n**

Following the general procedure for the *syn*-carboboration reaction, the treatment of alkyne **1l** (48.2 mg, 0.1 mmol, 1.0 equiv) in presence of CuCl (1 mg, 0.01 mmol, 10 mol%), $(PPh_3)_2PdCl_2$ (3.5 mg, 0.005 mmol, 5 mol%), XantPhos (5.78 mg, 0.01 mmol, 10 mol%), and benzyl bromide (17.8 µL, 0.15 mmol, 1.5 equiv) in THF (1.0 mL) yielded the titled compound after purification by flash column chromatography (n-hexane:toluene:AcOEt (10:10:5)) as an oil (62.3 mg, 89%). **^{1}H-NMR** (300 MHz, $CDCl_3$): δ 7.66 (d, J = 8.4 Hz, 2H), 7.46 (d, J = 8.4 Hz, 2H), 7.24-7.12 (m, 5H), 6.98 (d, J = 2.3 Hz, 1H), 6.89 (d, J = 9.0 Hz, 1H), 6.67 (dd, J = 9.0, 2.3 Hz, 1H), 4.11 (t, J = 6.6 Hz, 2H), 3.90 (s, 2H), 3.82 (s, 3H), 3.66 (s, 2H), 3.57 (s, 3H), 2.49 – 2.21 (m, 5H), 1.80 (p, J = 6.7 Hz, 2H), 1.27 (s, 12H). **^{13}C-NMR** (75 MHz, $CDCl_3$): δ 170.9, 169.1, 168.4, 156.2, 143.5, 139.3, 139.2, 135.9, 134.1, 131.3, 130.9, 130.8, 129.2, 128.8, 128.4, 126.2, 115.0, 112.9, 111.8, 101.4, 84.0, 65.1, 55.8, 51.3, 39.2, 30.4, 29.4, 29.0, 24.9, 13.5. **HRMS** (ESI^+) m/z $[M+H]^+$ calcd for $C_{39}H_{44}BClNO_8$ 700.2843, found 700.2847.

Methyl (*Z*)-2-phenyl-3-(4,4,5,5-tetramethyl-1,3,2-dioxaborolan-2-yl)hex-2-enoate (*Z*-5a):

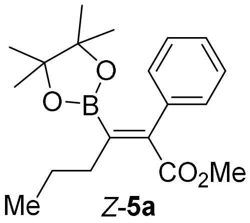

Z-**5a**

Following the general procedure for the *syn*-carboboration reaction, the treatment of alkyne **1a** (13.5 µL, 0.1 mmol, 1.0 equiv) in presence of CuCl (1 mg, 0.01 mmol, 10 mol%), $Pd_2(dba)_3 \cdot CHCl_3$ (2.6 mg, 0.0025 mmol, 5 mol% [Pd]), XantPhos (5.78 mg, 0.01 mmol, 10 mol%), and iodobenzene (16.8 µL, 0.15 mmol, 1.5 equiv) in toluene (1.0 mL) yielded the titled compound after purification by flash column chromatography (n-hexane:toluene:AcOEt (10:10:1)) as an oil (30 mg, 91%). The reaction delivered the titled compound in 80% yield at 2 mmol scale. **^{1}H-NMR** (300 MHz, $CDCl_3$): δ 7.35-7.32 (m, 5H), 3.74 (s, 3H), 2.52 – 2.42 (m, 2H), 1.63 – 1.52 (m, 2H), 1.09 (s, 12H), 1.00 (t, J = 7.3 Hz, 3H). **^{13}C-NMR** (75 MHz, $CDCl_3$): δ 168.8, 141.1, 139.0, 129.2, 128.0, 127.7, 83.9, 51.7, 35.5, 24.7, 22.8, 14.4. **^{11}B NMR** (96 MHz, $CDCl_3$) δ 31.0 **HRMS** (ESI^+) m/z $[M+H]^+$ calcd for $C_{19}H_{28}BO_4$ 331.2075, found 331.2079.

Methyl (*Z*)-2-(4-methoxyphenyl)-3-(4,4,5,5-tetramethyl-1,3,2-dioxaborolan-2-yl)hex-2-enoate (*Z*-5a):

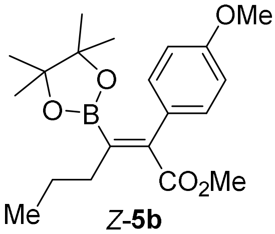

Z-**5b**

Following the general procedure for the *syn*-carboboration reaction, the treatment of alkyne **1a** (13.5 µL, 0.1 mmol, 1.0 equiv) in presence of CuCl (1 mg, 0.01 mmol, 10 mol%), $Pd_2(dba)_3 \cdot CHCl_3$ (2.6 mg, 0.0025 mmol, 5 mol% [Pd]), XantPhos (5.78 mg, 0.01 mmol, 10 mol%), and 4-iodoanisole (35.1 mg, 0.15 mmol, 1.5 equiv) in toluene (1.0 mL) yielded the titled compound after purification by flash column chromatography (n-hexane:toluene:AcOEt (10:10:1)) as an oil (31.3 mg, 87%). **^{1}H-NMR** (300 MHz, $CDCl_3$): δ 7.22 (d, J = 8.5 Hz, 2H), 6.81 (d, J = 8.6 Hz, 2H), 3.79 (s, 3H), 3.71 (s, 3H), 2.44 – 2.35 (m, 2H), 1.58 – 1.50 (m, 2H), 1.09 (s, 12H), 0.96 (t, J = 7.3 Hz, 3H). **^{13}C-RMN** (75 MHz, $CDCl_3$): δ 169.1, 159.4, 140.8, 131.5, 130.4, 113.4, 83.8, 55.4, 51.7, 35.6, 24.7, 22.8, 14.4. **HRMS** (ESI +) m/z [M+H]+ calcd for $C_{20}H_{30}BO_5$ 361.2181, found 361.2179.

Methyl (*Z*)-2-(9-phenyl-9*H*-carbazol-3-yl)-3-(4,4,5,5-tetramethyl-1,3,2-dioxaborolan-2-yl)hex-2-enoate (*Z*-5c):

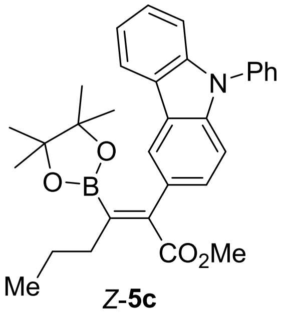

Z-**5c**

Following the general procedure for the *syn*-carboboration reaction, the treatment of alkyne **1a** (13.5 µL, 0.1 mmol, 1.0 equiv) in presence of CuCl (1 mg, 0.01 mmol, 10 mol%), $Pd_2(dba)_3·CHCl_3$ (2.6 mg, 0.0025 mmol, 5 mol% [Pd]), XantPhos (5.78 mg, 0.01 mmol, 10 mol%), and *N*-phenyl-3-iodocarbazole (55.4 mg, 0.15 mmol, 1.5 equiv) in toluene (1.0 mL) yielded the titled compound after purification by flash column chromatography (n-hexane:toluene:AcOEt (10:10:1)) as an oil (44.6 mg, 90%). **^{1}H-NMR** (300 MHz, $CDCl_3$): δ 8.17 – 8.02 (m, 2H), 7.65 – 7.51 (m, 4H), 7.51 – 7.43 (m, 1H), 7.44 – 7.32 (m, 4H), 7.32 – 7.27 (m, 1H), 3.76 (s, 3H), 2.54 – 2.44 (m, 2H), 1.70-1.55 (m, 2H), 1.08-0.99 (m, 15H). **^{13}C-NMR** (75 MHz, $CDCl_3$): δ 169.4, 141.8, 141.3, 140.6, 137.8, 130.9, 130.0, 127.6, 127.1, 126.0, 123.6, 123.1, 121.2, 120.5, 120.1, 109.9, 109.3, 83.8, 51.8, 35.8, 24.7, 22.9, 14.5. **HRMS** (ESI +) m/z [M+H]+ calcd for $C_{31}H_{35}BNO_4$ 496.2654, found 496.2649.

Methyl (*Z*)-2-(4-(4-fluorophenoxy)phenyl)-3-(4,4,5,5-tetramethyl-1,3,2-dioxaborolan-2-yl)hex-2-enoate (*Z*-5d):

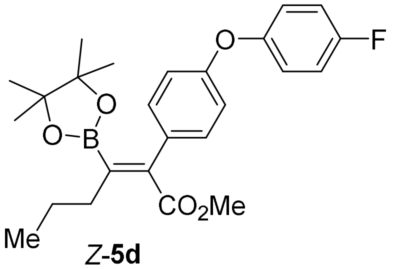

Z-**5d**

Following the general procedure for the *syn*-carboboration reaction, the treatment of alkyne **1a** (13.5 µL, 0.1 mmol, 1.0 equiv) in presence of CuCl (1 mg, 0.01 mmol, 10 mol%), $Pd_2(dba)_3·CHCl_3$ (2.6 mg, 0.0025 mmol, 5 mol% [Pd]), XantPhos (5.78 mg, 0.01 mmol, 10 mol%), and 4-fluoro-4′-iododiphenyl ether (47.1 mg, 0.15 mmol, 1.5 equiv) in toluene (1.0 mL) yielded the titled compound after purification by flash column chromatography (n-hexane:toluene:AcOEt (10:10:1)) as an oil (35.7 mg, 81%). **^{1}H-NMR** (300 MHz, $CDCl_3$): δ 7.22 (t, J = 7.9 Hz, 1H), 7.08 – 6.93 (m, 6H), 6.84 (ddd, J = 8.2, 2.5, 1.1 Hz, 1H), 3.72 (s, 3H), 2.48 – 2.36 (m, 2H), 1.59 – 1.45 (m, 2H), 1.11 (s, 12H), 0.96 (t, J = 7.3 Hz, 3H). **^{13}C-NMR** (75 MHz, $CDCl_3$): δ 168.5, 158.9 (d, J = 241.5 Hz), 157.1, 153.0 (d, J = 2.6 Hz), 140.6 (d, J = 32.4 Hz), 129.3, 124.2, 120.5 (d, J = 8.2 Hz), 119.4, 117.4, 116.4, 83.9, 51.8, 35.6, 24.8, 22.7, 14.4. **HRMS** (ESI +) m/z [M+H]+ calcd for $C_{25}H_{31}BFO_5$ 441.2243, found 441.2242.

Methyl (*E*)-3-(4,4,5,5-tetramethyl-1,3,2-dioxaborolan-2-yl)-2-(thiophen-2-yl)hex-2-enoate (*Z*-5e):

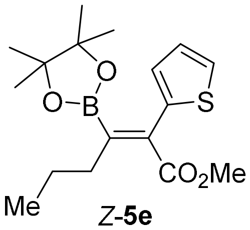

Z-**5e**

Following the general procedure for the *syn*-carboboration reaction, the treatment of alkyne **1a** (13.5 µL, 0.1 mmol, 1.0 equiv) in presence of CuCl (1 mg, 0.01 mmol, 10 mol%), $Pd_2(dba)_3·CHCl_3$ (2.6 mg, 0.0025 mmol, 5 mol% [Pd]), XantPhos (5.78 mg, 0.01 mmol, 10 mol%), and 2-iodothiophene (16.6 µL, 0.15 mmol, 1.5 equiv) in toluene (1.0 mL) yielded the titled compound after purification by flash column chromatography (n-hexane:toluene:AcOEt (10:10:1)) as an oil (24.2 mg, 72%). **^{1}H-NMR** (300 MHz, $CDCl_3$): δ 7.26 – 7.23 (m, 1H), 6.99 (dd, J = 3.5, 1.2 Hz, 1H), 6.92 (dd, J = 5.1, 3.5 Hz, 1H), 3.75 (s, 3H), 2.48 – 2.39 (m, 2H), 1.58 – 1.48 (m, 2H), 1.13 (s, 12H), 0.96 (t, J = 7.3 Hz, 3H). **^{13}C-NMR** (75 MHz, $CDCl_3$): δ 167.9, 140.3, 133.3, 128.0, 126.4, 126.3, 84.1, 51.9, 35.6, 24.8, 22.6, 14.4. **HRMS** (ESI +) m/z [M+H]+ calcd for $C_{17}H_{26}BO_4S$ 337.1639, found 337.1633.

Methyl (*Z*)-2-(4-((*1s,4r*)-4-propylcyclohexyl)phenyl)-3-(4,4,5,5-tetramethyl-1,3,2-dioxaborolan-2-yl)hex-2-enoate (*Z*-5f):

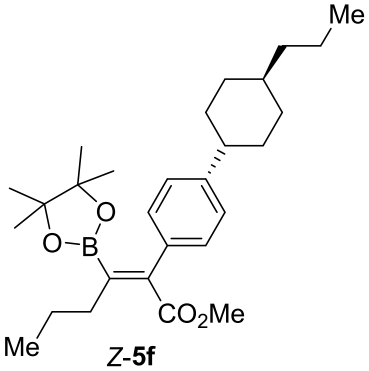

Z-**5f**

Following the general procedure for the *syn*-carboboration reaction, the treatment of alkyne **1a** (13.5 µL, 0.1 mmol, 1.0 equiv) in presence of CuCl (1 mg, 0.01 mmol, 10 mol%), $Pd_2(dba)_3 \cdot CHCl_3$ (2.6 mg, 0.0025 mmol, 5 mol% [Pd]), XantPhos (5.78 mg, 0.01 mmol, 10 mol%), and 1-(trans-*N*-butylcyclohexyl)-4-iodobenzene (51.3 mg, 0.15 mmol, 1.5 equiv) in toluene (1.0 mL) yielded the titled compound after purification by flash column chromatography (n-hexane:toluene:AcOEt (10:10:1)) as an oil (40.4 mg, 89%). **^{1}H-NMR** (300 MHz, $CDCl_3$): δ 7.20 (d, *J* = 8.1 Hz, 2H), 7.11 (d, *J* = 8.2 Hz, 2H), 3.71 (s, 3H), 2.44 – 2.38 (m, 2H), 1.88-1.80 (m, 4H), 1.55 – 1.23 (m, 12H), 1.04 (s, 12H), 0.97 (d, *J* = 7.3 Hz, 3H), 0.91 (d, *J* = 7.0 Hz, 3H). **^{13}C-NMR** (75 MHz, $CDCl_3$): δ 168.9, 147.5, 140.9, 136.5, 129.1, 126.5, 83.8, 51.7, 44.7, 39.9, 37.2, 35.5, 34.5, 33.7, 24.7, 22.8, 20.2, 14.5, 14.4. **HRMS** (ESI +) m/z [M+H]+ calcd for $C_{28}H_{44}BO_4$ 455.3327, found 455.3327.

Ethyl (*Z*)-3-(1-methoxy-1-oxo-3-(4,4,5,5-tetramethyl-1,3,2-dioxaborolan-2-yl)hex-2-en-2-yl)benzoate (*Z*-5g):

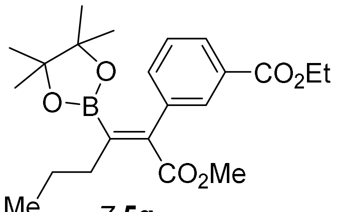

Z-**5g**

Following the general procedure for the *syn*-carboboration reaction, the treatment of alkyne **1a** (13.5 µL, 0.1 mmol, 1.0 equiv) in presence of CuCl (1 mg, 0.01 mmol, 10 mol%), $Pd_2(dba)_3 \cdot CHCl_3$ (2.6 mg, 0.0025 mmol, 5 mol% [Pd]), XantPhos (5.78 mg, 0.01 mmol, 10 mol%), and ethyl 3-iodobenzoate (41.4 mg, 0.15 mmol, 1.5 equiv) in toluene (1.0 mL) yielded the titled compound after purification by flash column chromatography (n-hexane:toluene:AcOEt (10:10:1)) as an oil (28.2 mg, 70%). **^{1}H-NMR** (300 MHz, $CDCl_3$): δ 7.97-7.89 (m, 2H), 7.49-7.42 (m, 1H), 7.37-7.29 (m, 1H), 4.38 – 4.28 (m, 2H), 3.68 (s, 3H), 2.49 – 2.37 (m, 2H), 1.59 – 1.45 (m, 2H), 1.34 (7, J = 7.0 Hz, 3H), 1.01 (s, 12H), 0.94 (t, J = 7.2 Hz, 3H). **^{13}C-NMR** (75 MHz, $CDCl_3$): δ 168.2, 166.4, 140.3, 139.2, 133.4, 130.3, 130.1, 128.8, 127.9, 83.8, 60.9, 51.7, 35.4, 24.5, 22.6, 14.3, 14.2. **HRMS** (ESI +) m/z [M+H]+ calcd for $C_{22}H_{32}BO_6$ 403.2286, found 403.2284.

Methyl (*Z*)-3-(4,4,5,5-tetramethyl-1,3,2-dioxaborolan-2-yl)-2-(4-(trifluoromethyl)phenyl)hex-2-enoate (*Z*-5h):

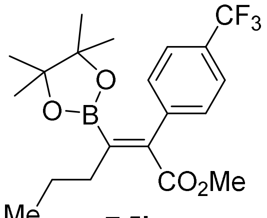

Z-**5h**

Following the general procedure for the *syn*-carboboration reaction, the treatment of alkyne **1a** (13.5 µL, 0.1 mmol, 1.0 equiv) in presence of CuCl (1 mg, 0.01 mmol, 10 mol%), $Pd_2(dba)_3 \cdot CHCl_3$ (2.6 mg, 0.0025 mmol, 5 mol% [Pd]), XantPhos (5.78 mg, 0.01 mmol, 10 mol%), and 4-iodobenzotrifluoride (22 µL, 0.15 mmol, 1.5 equiv) in toluene (1.0 mL) yielded the titled compound after purification by flash column chromatography (n-hexane:toluene:AcOEt (10:10:1)) as an oil (24.7 mg, 62%). **^{1}H-NMR** (300 MHz, $CDCl_3$): δ 7.20 (d, *J* = 8.1 Hz, 2H), 7.11 (d, *J* = 8.2 Hz, 2H), 3.71 (s, 3H), 2.44 – 2.38 (m, 2H), 1.88-1.80 (m, 4H), 1.55 – 1.23 (m, 12H), 1.04 (s, 12H), 0.97 (d, *J* = 7.3 Hz, 3H), 0.91 (d, *J* = 7.0 Hz, 3H). **^{13}C-NMR** (75 MHz, $CDCl_3$): δ 167.9, 142.9, 139.7, 130.0, 129.8, 129.6, 124.8 (q, J = 3.8 Hz), 84.0, 51.8, 35.4, 24.5, 22.7, 14.3. **HRMS** (ESI^+) m/z $[M+H]^+$ calcd for $C_{20}H_{27}BF_3O_4$ 399.1949, found 399.1960.

3-(Naphthalen-1-yl)prop-2-yn-1-yl (Z)-2-phenyl-3-(4,4,5,5-tetramethyl-1,3,2-dioxaborolan-2-yl)hex-2-enoate (*Z*-5i):

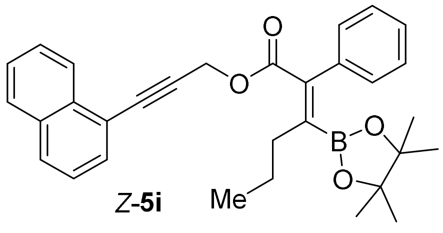
Z-5i

Following the general procedure for the *syn*-carboboration reaction, the treatment of alkyne **1o** (27.6 mg, 0.1 mmol, 1.0 equiv) in presence of CuCl (1 mg, 0.01 mmol, 10 mol%), $Pd_2(dba)_3 \cdot CHCl_3$ (2.6 mg, 0.0025 mmol, 5 mol% [Pd]), XantPhos (5.78 mg, 0.01 mmol, 10 mol%), and iodobenzene (16.8 µL, 0.15 mmol, 1.5 equiv) in toluene (1.0 mL) yielded the titled compound after purification by flash column chromatography (n-hexane:toluene:AcOEt (10:10:1)) as an oil (36 mg, 75%). **^{1}H-NMR** (300 MHz, $CDCl_3$): δ 8.09 (d, J = 7.7 Hz, 1H), 7.66 (d, J = 8.1 Hz, 2H), 7.48 (d, J = 7.1 Hz, 1H), 7.42 – 7.28 (m, 2H), 7.24 – 7.00 (m, 6H), 4.94 (s, 2H), 2.40 – 2.22 (m, 2H), 1.46 – 1.33 (m, 2H), 0.90 (s, 12H), 0.78 (t, J = 7.3 Hz, 3H). **^{13}C-NMR** (75 MHz, $CDCl_3$): δ 167.7, 140.6, 140.6, 138.6, 133.5, 133.2, 130.8, 129.3, 128.3, 128.0, 127.9, 127.0, 126.6, 126.3, 125.2, 120.1, 88.1, 84.6, 83.9, 53.1, 35.6, 24.7, 22.8, 14.4. **HRMS** (ESI +) m/z $[M+H]^+$ calcd for $C_{31}H_{34}BO_4$ 481.2545, found 481.2545.

Methyl (*Z*)-7-chloro-2-phenyl-3-(4,4,5,5-tetramethyl-1,3,2-dioxaborolan-2-yl)hept-2-enoate (*Z*-5j):

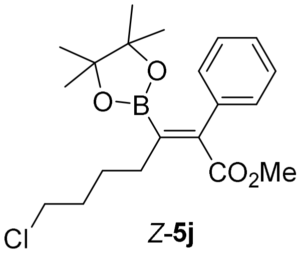
Z-5j

Following the general procedure for the *syn*-carboboration reaction, the treatment of alkyne **1d** (17.4 mg, 0.1 mmol, 1.0 equiv) in presence of CuCl (1 mg, 0.01 mmol, 10 mol%), $Pd_2(dba)_3 \cdot CHCl_3$ (2.6 mg, 0.0025 mmol, 5 mol% [Pd]), XantPhos (5.78 mg, 0.01 mmol, 10 mol%), and iodobenzene (16.8 µL, 0.15 mmol, 1.5 equiv) in toluene (1.0 mL) yielded the titled compound after purification by flash column chromatography (n-hexane:toluene:AcOEt (10:10:1)) as an oil (32.9 mg, 87%). **^{1}H-NMR** (300 MHz, $CDCl_3$): δ 7.28 (s, 5H), 3.71 (s, 3H), 3.57 (t, J = 6.6 Hz, 2H), 2.62 – 2.42 (m, 2H), 1.86 (dt, J = 14.6, 6.7 Hz, 2H), 1.73-1.63 (m, 2H), 1.06 (s, 12H). **^{13}C-NMR** (75 MHz, $CDCl_3$): δ 168.6, 141.6, 138.9, 129.2, 128.0, 127.9, 84.0, 51.9, 45.1, 32.5, 29.9, 26.6, 24.7. **HRMS** (ESI +) m/z $[M+H]^+$ calcd for $C_{20}H_{29}BClO_4$ 379.1842, found 379.1845.

Methyl (*Z*)-2-phenyl-3-(4,4,5,5-tetramethyl-1,3,2-dioxaborolan-2-yl)-3-(trimethylsilyl)acrylate (*Z*-5k):

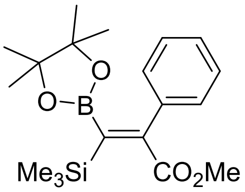
Z-5k

Following the general procedure for the *syn*-carboboration reaction, the treatment of methyl (trimethylsilyl)propiolate (15.6 mg, 0.1 mmol, 1.0 equiv) in presence of CuCl (1 mg, 0.01 mmol, 10 mol%), $Pd_2(dba)_3 \cdot CHCl_3$ (2.6 mg, 0.0025 mmol, 5 mol% [Pd]), XantPhos (5.78 mg, 0.01 mmol, 10 mol%), and iodobenzene (16.8 µL, 0.15 mmol, 1.5 equiv) in toluene (1.0 mL) yielded the titled compound after purification by flash column chromatography (n-hexane:toluene:AcOEt (10:10:1)) as an oil (33.5 mg, 93%). **^{1}H-NMR** (300 MHz, $CDCl_3$): δ 7.39 – 7.19 (m, 5H), 3.69 (s, 3H), 0.99 (s, 12H), 0.24 (s, 9H). **^{13}C-NMR** (75 MHz, $CDCl_3$): δ 168.3, 151.5, 140.8, 129.0, 127.8, 127.8, 83.9, 52.0, 25.2, 0.3. **HRMS** (ESI +) m/z $[M+H]^+$ calcd for $C_{19}H_{30}BO_4Si$ 361.2001, found 361.2008.

(*8R,9S,13S,14S*)-13-Methyl-17-oxo-7,8,9,11,12,13,14,15,16,17-decahydro-6*H*-cyclopenta[a]phenanthren-3-yl (*Z*)-2-phenyl-3-(4,4,5,5-tetramethyl-1,3,2-dioxaborolan-2-yl)hex-2-enoate (*Z*-5l):

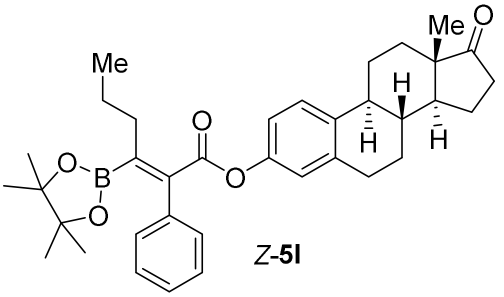
Z-5l

Following the general procedure for the *syn*-carboboration reaction, the treatment of alkyne **1f** (36.5 mg, 0.1 mmol, 1.0 equiv) in presence of CuCl (1 mg, 0.01 mmol, 10 mol%), $Pd_2(dba)_3 \cdot CHCl_3$ (2.6 mg, 0.0025 mmol, 5 mol% [Pd]), XantPhos (5.78 mg, 0.01 mmol, 10 mol%), and iodobenzene (16.8 µL, 0.15 mmol, 1.5 equiv) in toluene (1.0 mL) yielded the titled compound after purification by flash column chromatography (n-hexane:toluene:AcOEt (10:10:1)) as an oil (45.5 mg, 80%). **^{1}H-NMR** (300 MHz,

CDCl3): δ 7.44 – 7.27 (m, 6H), 6.84 (dd, J = 8.5, 2.4 Hz, 1H), 6.79 (d, J = 2.4 Hz, 1H), 2.89 (dd, J = 8.2, 3.7 Hz, 2H), 2.64 – 2.53 (m, 2H), 2.52 – 2.44 (m, 1H), 2.32-2.20 (m, 1H), 2.21 – 2.11 (m, 1H), 2.09 – 1.94 (m, 3H), 1.69 – 1.43 (m, 9H), 1.09 (s, 12H), 1.04 – 0.98 (m, 3H), 0.89 (s, 3H). **^{13}C-NMR** (75 MHz, CDCl3): δ 220.9, 166.7, 148.7, 140.4, 138.7, 138.0, 137.4, 129.3, 128.0, 127.9, 126.4, 121.6, 118.9, 84.0, 50.5, 48.0, 44.2, 38.1, 36.0, 35.6, 31.6, 29.5, 26.4, 25.9, 24.7, 22.7, 21.7, 14.4, 13.9. **HRMS** (ESI +) m/z $[M+H]^+$ calcd for $C_{36}H_{46}BO_5$ 569.3433, found 569.3445.

Methyl (*Z*)-6-(2-(1-(4-chlorobenzoyl)-5-methoxy-2-methyl-1H-indol-3-yl)acetoxy)-2-phenyl-3-(4,4,5,5-tetramethyl-1,3,2-dioxaborolan-2-yl)hex-2-enoate (*Z*-5m):

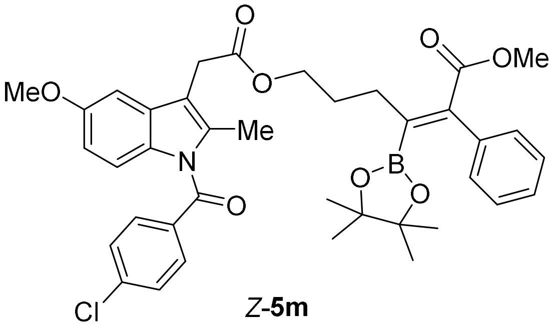

Z-**5m**

Following the general procedure for the *syn*-carboboration reaction, the treatment of alkyne **1l** (48.2 mg, 0.1 mmol, 1.0 equiv) in presence of CuCl (1 mg, 0.01 mmol, 10 mol%), $Pd_2(dba)_3·CHCl_3$ (2.6 mg, 0.0025 mmol, 5 mol% [Pd]), XantPhos (5.78 mg, 0.01 mmol, 10 mol%), and iodobenzene (16.8 µL, 0.15 mmol, 1.5 equiv) in toluene (1.0 mL) yielded the titled compound after purification by flash column chromatography (n-hexane:toluene:AcOEt (2.5:2.5:1)) as an oil (62.4 mg, 91%). **^{1}H-NMR** (300 MHz, CDCl3): δ 7.66 (dd, J = 8.6, 1.9 Hz, 2H), 7.50 – 7.42 (m, 2H), 7.27 (s, 5H), 6.99 (d, J = 2.5 Hz, 1H), 6.89 (d, J = 9.0 Hz, 1H), 6.66 (dd, J = 9.0, 2.5 Hz, 1H), 4.17 (t, J = 6.6 Hz, 2H), 3.83 (s, 3H), 3.72-3.64 (m, 5H), 2.57 – 2.46 (m, 2H), 2.38 (s, 3H), 1.93-1.83 (m, 2H), 1.03 (s, 12H). **^{13}C-NMR** (75 MHz, CDCl3): δ 170.9, 168.4, 168.3, 156.2, 142.0, 139.3, 138.7, 136.0, 134.1, 131.3, 130.9, 130.8, 129.2, 129.1, 128.0, 127.9, 115.1, 112.9, 111.9, 101.4, 84.0, 65.0, 55.8, 51.8, 30.4, 29.7, 28.4, 24.6, 13.5. **HRMS** (ESI +) m/z [M+H]+ calcd for $C_{38}H_{42}BClNO_8$ 686.2687, found 686.2693.

(*Z*)-6-methoxy-6-oxo-5-phenyl-4-(4,4,5,5-tetramethyl-1,3,2-dioxaborolan-2-yl)hex-4-en-1-yl (*2S,4aR,7S,9aS,10S*)-1-methyl-8-methylene-13-oxo-2,7-bis((triethylsilyl)oxy)-1,2,4b,5,6,7,8,9,10,10a-decahydro-4a,1-(epoxymethano)-7,9a-methanobenzo[a]azulene-10-carboxylate (*Z*-5n):

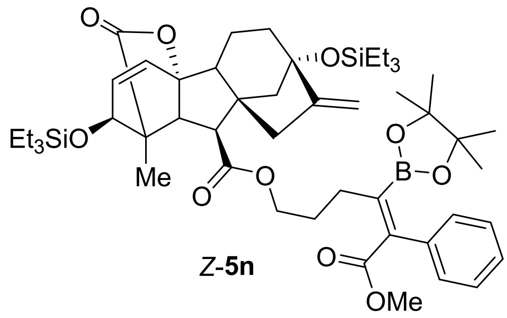

Z-**5n**

Following the general procedure for the *syn*-carboboration reaction, the treatment of alkyne **1p** (69.9 mg, 0.1 mmol, 1.0 equiv) in presence of CuCl (1 mg, 0.01 mmol, 10 mol%), $Pd_2(dba)_3·CHCl_3$ (2.6 mg, 0.0025 mmol, 5 mol% [Pd]), XantPhos (5.78 mg, 0.01 mmol, 10 mol%), and iodobenzene (16.8 µL, 0.15 mmol, 1.5 equiv) in toluene (1.0 mL) yielded the titled compound after purification by flash column chromatography (n-hexane:toluene:AcOEt (5:5:1)) as an oil (61.4 mg, 68%). **^{1}H-NMR** (300 MHz, CDCl3): δ 7.19 (s, 5H), 6.13 (d, J = 9.4 Hz, 1H), 5.67 (dd, J = 9.3, 3.6 Hz, 1H), 5.13 (s, 1H), 4.80 (s, 1H), 4.28 – 3.99 (m, 3H), 3.62 (s, 3H), 3.25 (d, J = 10.8 Hz, 1H), 2.67 (d, J = 10.8 Hz, 1H), 2.54 – 2.37 (m, 2H), 2.10 (s, 2H), 1.97 – 1.69 (m, 9H), 1.12 (s, 3H), 0.97 (s, 12H), 0.93 – 0.79 (m, 18H), 0.62 – 0.43 (m, 12H). **^{13}C-NMR** (75 MHz, CDCl3): δ 179.3, 172.0, 168.3, 157.2, 142.1, 138.8, 133.2, 131.6, 129.2, 128.0, 127.9, 107.4, 91.1, 84.0, 79.3, 70.2, 65.0, 54.3, 52.9, 51.9, 51.3, 51.0, 50.8, 44.5, 43.0, 40.8, 29.7, 28.4, 24.7, 17.2, 15.1, 7.2, 6.9, 6.6, 5.1. **HRMS** (ESI +) m/z $[M+H]^+$ calcd for $C_{50}H_{76}BO_{10}Si_2$ 903.5065, found 903.5056.

***rac*-(*R*)-2,5,7,8-Tetramethyl-2-((*4R,8R*)-4,8,12-trimethyltridecyl)chroman-6-yl (*Z*)-2-phenyl-3-(4,4,5,5-tetramethyl-1,3,2-dioxaborolan-2-yl)hex-2-enoate (*Z*-5o):**

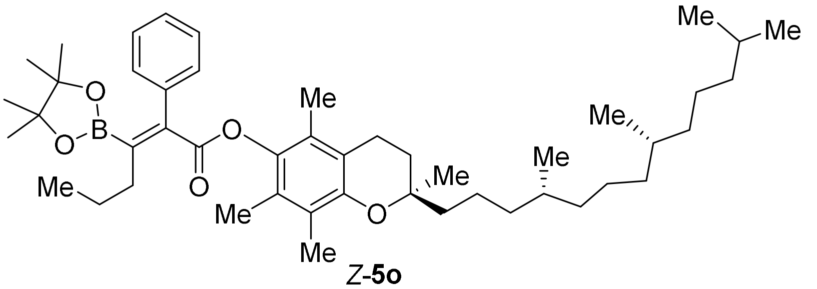

Z-**5o**

Following the general procedure for the *syn*-carboboration reaction, the treatment of alkyne **1g** (52.5 mg, 0.1 mmol, 1.0 equiv) in presence of CuCl (1 mg, 0.01 mmol, 10 mol%), $Pd_2(dba)_3 \cdot CHCl_3$ (2.6 mg, 0.0025 mmol, 5 mol% [Pd]), XantPhos (5.78 mg, 0.01 mmol, 10 mol%), and iodobenzene (16.8 µL, 0.15 mmol, 1.5 equiv) in toluene (1.0 mL) yielded the titled compound after purification by flash column chromatography (n-hexane:toluene:AcOEt (10:10:1)) as an oil (53.9 mg, 74%). **^{1}H-NMR** (300 MHz, $CDCl_3$): δ 7.45 – 7.42 (m, 1H), 7.39-7.23 (m, 4H), 2.70 – 2.62 (m, 2H), 2.61 – 2.53 (m, 2H), 2.06 (s, 3H), 1.91 (s, 3H), 1.87 (s, 3H), 1.46 – 1.26 (m, 25H), 1.05 (s, 12H), 1.00 (d, J = 6.3 Hz, 3H), 0.93-0.83 (m, 15H). **^{13}C-NMR** (75 MHz, $CDCl_3$): δ 165.9, 149.4, 140.6, 140.1, 139.3, 129.8, 127.9, 127.6, 126.9, 125.1, 123.1, 117.4, 83.9, 75.7, 39.5, 37.6, 37.5, 37.4, 35.1, 32.9, 32.8, 28.1, 25.3, 25.2, 25.0, 24.9, 24.6, 24.5, 22.9 (2C), 22.8, 21.2, 20.7, 19.9, 19.8, 19.8, 14.5, 13.2, 12.3, 11.9. **HRMS** (ESI +) m/z $[M+H]^+$ calcd for $C_{47}H_{74}BO_5$ 729.5624, found 729.5630.

4 Photoisomerization of tetrasubstituted olefins.

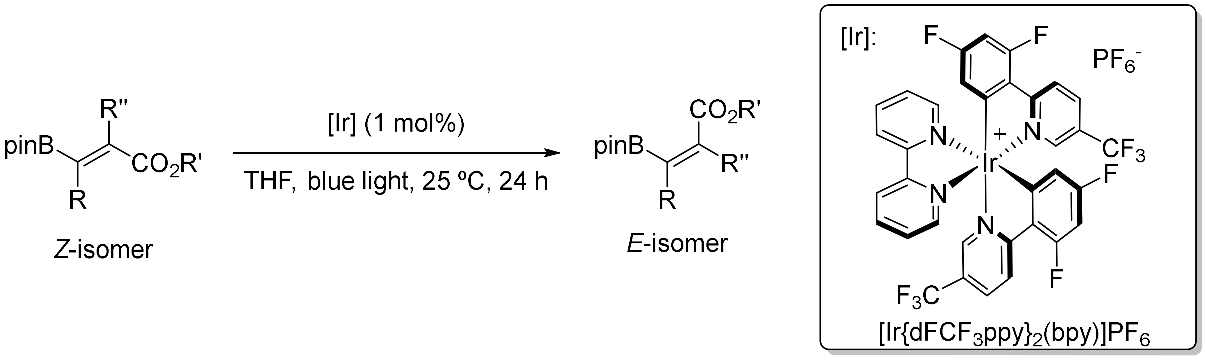

General procedure for the isomerization of alkenyl boronates: in an oven-dried scintillation vial provided with a stir bar, the corresponding *syn*-alkenyl boronate (*Z*-**3**, *Z*-**4**, *Z*-**5**) and the photosensitizer $[Ir\{dFCF_3ppy\}_2(bpy)]PF_6$ (1 mol%) were added and the air was replaced by an argon atmosphere. After that, THF (1 mL) was added, and the reaction vial was irradiated for 24 h under blue light irradiation while the temperature was kept at 25 ºC. Then, the resulting solution was filtered off through a pad of silica gel using n-hexane:AcOEt (7:3) as eluent and the solvent was removed to afford the corresponding *E*-alkenyl boronic ester.

Note: when the photoisomerization is not completed, the resulting residue was further purified by column chromatography (indicated in each case) to afford pure samples of the desired E-*isomers.*

Methyl (*E*)-2-methyl-3-(4,4,5,5-tetramethyl-1,3,2-dioxaborolan-2-yl)hex-2-enoate (*E*-3a):

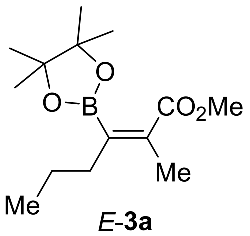

E-3a

Following the general procedure for the *photoisomerization reaction*, the treatment of alkenyl boronate *Z*-**3a** (23.3mg, 0.087 mmol, 1.0 equiv) in presence of [Ir{dFCF$_3$ppy}$_2$(bpy)]PF$_6$ (0.88 mg, 0.00088 mmol, 1 mol%) in THF afforded the titled compound as an oil after filtration through a pad of silica gel (23.3 mg, >99%). **^{1}H-RMN** (300 MHz, CDCl$_3$): δ 3.76 (s, 3H), 2.23 (t, J = 7.5 Hz, 2H), 1.82 (s, 3H), 1.48 (dq, J = 14.5, 7.2 Hz, 2H), 1.33 (s, 12H), 0.93 (t, J = 7.4 Hz, 3H). **^{13}C-RMN** (75 MHz, CDCl$_3$): δ 171.1, 132.0, 83.4, 52.5, 33.8, 25.1, 21.5, 14.6, 12.3. **^{11}B NMR** (96 MHz, CDCl$_3$) δ 29.9. **HRMS** (ESI+) m/z [M + H]+ calcd for $C_{14}H_{26}BO_4$ 269.1919, found 269.1911.

Methyl (*E*)-3-cyclohexyl-2-methyl-3-(4,4,5,5-tetramethyl-1,3,2-dioxaborolan-2-yl)acrylate (*E*-3b):

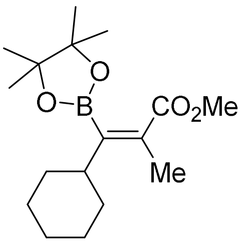

E-3b

Following the general procedure for the *photoisomerization reaction*, the treatment of alkenyl boronate *Z*-**3b** (23.7 mg, 0.077 mmol, 1.0 equiv) in presence of [Ir{dFCF$_3$ppy}$_2$(bpy)]PF$_6$ (0.78 mg, 0.00078 mmol, 1 mol%) in THF afforded a mixture of *E*- and *Z*-isomers of the titled compound (*E:Z* = 85:15). The titled compound was further purified by column chromatography (n-hexane:toluene:AcOEt 15:15:1) to afford the spectroscopically pure *E*-isomer (*E:Z > 98:2)* as an oil (17.1 mg, 72%). **^{1}H-RMN** (300 MHz, CDCl$_3$): δ 3.73 (s, 3H), 2.45 (tt, *J* = 11.7, 3.5 Hz, 1H), 1.83 (s, 3H), 1.81 – 1.59 (m, 6H), 1.36 (s, 12H), 1.30 – 1.11 (m, 4H). **^{13}C-RMN** (75 MHz, CDCl$_3$): δ 171.0, 130.9, 83.5, 52.3, 42.0, 31.4, 26.6, 26.3, 25.7, 12.5. **HRMS** (ESI+) m/z [M + H]+ calcd for $C_{17}H_{30}BO_4$ 309.2232, found 309.2228.

Cycloheptyl (*E*)-2-methyl-3-(4,4,5,5-tetramethyl-1,3,2-dioxaborolan-2-yl)hex-2-enoate (*E*-3c):

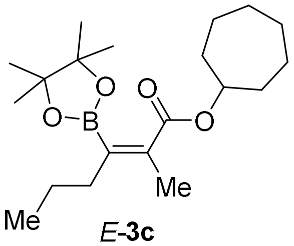

E-3c

Following the general procedure for the *photoisomerization reaction*, the treatment of alkenyl boronate *Z*-**3c** (31.9 mg, 0.091 mmol, 1.0 equiv) in presence of [Ir{dFCF$_3$ppy}$_2$(bpy)]PF$_6$ (0.92 mg, 0.00092 mmol, 1 mol%) in THF afforded the titled compound as an oil after filtration through a pad of silica gel (30.9 mg, 97%). **^{1}H-RMN** (300 MHz, CDCl$_3$): δ 5.08-4.97 (m, 1H), 2.20(t, J = 7.6 Hz, 2H), 1.91-1.83 (m, 2H), 1.78 (s, 3H), 1.67 – 1.40 (m, 12H), 1.31 (s, 12H), 0.92 (t, J = 7.3 Hz, 3H). **^{13}C-RMN** (75 MHz, CDCl$_3$): δ 171.3, 132.2, 82.9, 33.8, 28.5, 25.1, 22.9, 21.4, 14.6, 11.7. **HRMS** (ESI+) m/z [M + H]+ calcd for $C_{20}H_{36}BO_4$ 351.2701, found 351.2694.

Methyl (*E*)-7-chloro-2-methyl-3-(4,4,5,5-tetramethyl-1,3,2-dioxaborolan-2-yl)hept-2-enoate (*Z*-3d):

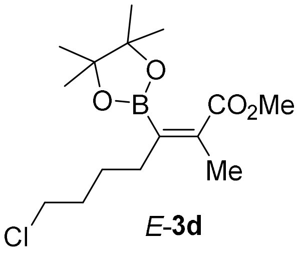

E-3d

Following the general procedure for the *photoisomerization reaction*, the treatment of alkenyl boronate *Z*-**3d** (21.5 mg, 0.068 mmol, 1.0 equiv) in presence of [Ir{dFCF$_3$ppy}$_2$(bpy)]PF$_6$ (0.69 mg, 0.00069 mmol, 1 mol%) in THF afforded the titled compound as an oil after filtration through a pad of silica gel (19.6 mg, 91%). **^{1}H-RMN** (300 MHz, CDCl$_3$): δ 3.77 (s, 3H), 3.53 (t, *J* = 6.5 Hz, 2H), 2.27 (t, *J* = 7.7 Hz, 2H), 1.87 – 1.73 (m, 5H), 1.68-1.56 (m, 2H), 1.34 (s, 12H). **^{13}C-RMN** (75 MHz, CDCl$_3$): δ 171.1, 132.4, 83.5, 52.6, 44.9, 32.6, 30.7, 25.3, 25.2, 12.3. **HRMS** (ESI+) m/z [M + H]+ calcd for $C_{15}H_{27}BClO_4$ 317.1685, found 317.1679.

Methyl (*E*)-6-cyano-2-methyl-3-(4,4,5,5-tetramethyl-1,3,2-dioxaborolan-2-yl)hex-2-enoate (*E*-3e):

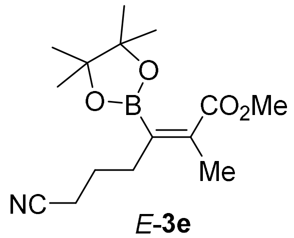

E-**3e**

Following the general procedure for the *photoisomerization reaction*, the treatment of alkenyl boronate *Z*-**3e** (25.2 mg, 0.086 mmol, 1.0 equiv) in presence of [Ir{dFCF$_3$ppy}$_2$(bpy)]PF$_6$ (0.87 mg, 0.00087 mmol, 1 mol%) in THF afforded the titled compound as an oil after filtration through a pad of silica gel (25.2 mg, >99%). **^{1}H-RMN** (300 MHz, CDCl$_3$): δ 3.78 (s, 3H), 2.53 – 2.14 (m, 4H), 1.96 – 1.71 (m, 5H), 1.34 (s, 12H). **^{13}C-RMN** (75 MHz, CDCl$_3$): δ 171.1, 133.9, 119.7, 83.6, 52.8, 29.8, 25.2, 24.0, 16.9, 12.4. **HRMS** (ESI+) m/z [M+H]$^+$ calcd for $C_{15}H_{25}BNO_4$ 294.1871, found 294.1873.

(*8R,9S,13S,14S*)-13-methyl-17-oxo-7,8,9,11,12,13,14,15,16,17-decahydro-6*H*-cyclopenta[a]phenanthren-3-yl (*E*)-2-methyl-3-(4,4,5,5-tetramethyl-1,3,2-dioxaborolan-2-yl)hex-2-enoate (*E*-3g):

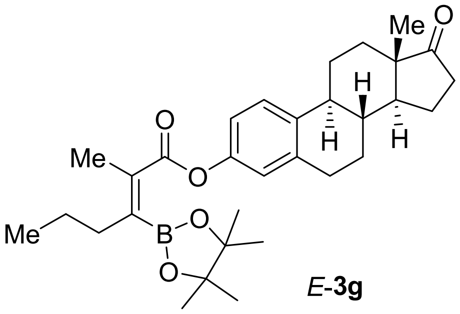

E-**3g**

Following the general procedure for the *photoisomerization reaction*, the treatment of alkenyl boronate *Z*-**3g** (41 mg, 0.081 mmol, 1.0 equiv) in presence of [Ir{dFCF$_3$ppy}$_2$(bpy)]PF$_6$ (0.82 mg, 0.00082 mmol, 1 mol%) in THF afforded the titled compound as an oil after filtration through a pad of silica gel (37.3 mg, 91%). **^{1}H-RMN** (300 MHz, CDCl$_3$): δ 7.27-7.24 (m, 1H), 6.94 – 6.85 (m, 1H), 6.85 (bs, 1H), 2.95-2.84 (m, 2H), 2.50 (dd, *J* = 18.3, 8.3 Hz, 1H), 2.46 – 2.34 (m, 1H), 2.30 (t, *J* = 7.5 Hz, 2H), 2.24 – 2.02 (m, 3H), 1.97 (s, 3H), 1.69 – 1.40 (m, 10H), 1.26 (s, 12H), 0.98 (t, *J* = 7.3 Hz, 3H), 0.91 (s, 3H). **^{13}C-RMN** (75 MHz, CDCl$_3$): δ 220.9, 168.2, 149.0, 137.8, 137.2, 132.3, 126.2, 121.9, 119.1, 83.6, 50.6, 48.1, 44.3, 38.1, 36.0, 34.4, 31.7, 29.5, 26.5, 25.9, 25.1, 21.7, 21.5, 14.6, 14.0, 13.1. **HRMS** (APCI+) m/z [M]+ calcd for $C_{31}H_{44}BO_5$ 507.3276, found 507.3281.

Methyl (*E*)-2-benzyl-3-(4,4,5,5-tetramethyl-1,3,2-dioxaborolan-2-yl)hex-2-enoate (*E*-4a):

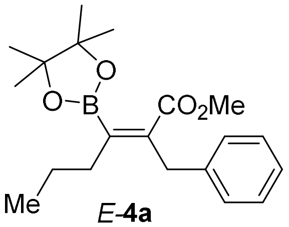

E-**4a**

Following the general procedure for the *photoisomerization reaction*, the treatment of alkenyl boronate *Z*-**4a** (29.6 mg, 0.086 mmol, 1.0 equiv) in presence of [Ir{dFCF$_3$ppy}$_2$(bpy)]PF$_6$ (0.87 mg, 0.00087 mmol, 1 mol%) in THF afforded the titled compound as an oil after filtration through a pad of silica gel (29.6 mg, >99%). **^{1}H-RMN** (300 MHz, CDCl$_3$): δ 7.20 – 7.03 (m, 5H), 3.65-3.59 (m, 5H), 2.30 – 2.17 (m, 2H), 1.50-1.36 (m, 2H), 1.28 (s, 12H), 0.85 (t, J = 7.3 Hz, 3H). **^{13}C-RMN** (75 MHz, CDCl$_3$): δ 170.9, 139.6, 134.4, 128.4, 126.0, 83.5, 52.5, 34.2, 32.2, 25.1, 21.7, 14.7.**^{11}B NMR** (96 MHz, CDCl$_3$) δ 30.2. **HRMS** (TOF MS EI$^+$) m/z [M]+ calcd for $C_{20}H_{29}BO_4$ 344.2159, found 344.2152.

Methyl (*E*)-2-(2-bromobenzyl)-3-(4,4,5,5-tetramethyl-1,3,2-dioxaborolan-2-yl)hex-2-enoate (*E*-4b):

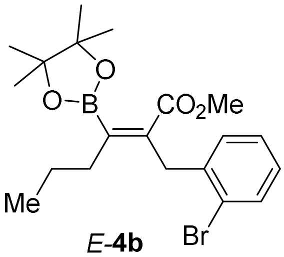

E-**4b**

Following the general procedure for the *photoisomerization reaction*, the treatment of alkenyl boronate *Z*-**4b** (27 mg, 0.064 mmol, 1.0 equiv) in presence of [Ir{dFCF$_3$ppy}$_2$(bpy)]PF$_6$ (0.65 mg, 0.00065 mmol, 1 mol%) in THF afforded a mixture of *E*- and *Z*-isomers of the titled compound (*E:Z* = 75:25). The titled compound was further purified by column chromatography (n-hexane:toluene:AcOEt 15:15:1) to afford the spectroscopically pure *E*-isomer (*E:Z > 98:2)* as an oil (18.6 mg, 69%). **^{1}H-RMN** (300 MHz, CDCl$_3$): δ 7.53 (d, J = 8.1 Hz, 1H), 7.20 – 7.11 (m, 1H), 7.03 (t, J = 7.5 Hz, 2H), 3.74 (s, 2H), 3.68 (s, 3H), 2.24-2.15 (m, 2H), 1.54 – 1.42 (m, 2H), 1.37 (s, 12H), 0.89 (t, J = 7.3 Hz, 3H). **^{13}C-RMN** (75 MHz, CDCl$_3$): δ 170.5, 138.6, 133.2, 132.6, 129.0, 127.7, 127.5, 124.8, 83.6, 52.7, 34.3, 32.5, 25.1, 21.6, 14.6. **HRMS** (ESI$^+$) m/z [M+H]$^+$ calcd for $C_{20}H_{29}BBrO_4$ 423.1337, found 423.1334.

Methyl (*E*)-2-(4-bromobenzyl)-3-(4,4,5,5-tetramethyl-1,3,2-dioxaborolan-2-yl)hex-2-enoate (*E*-4c):

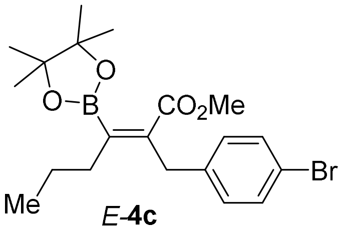

E-**4c**

Following the general procedure for the *photoisomerization reaction*, the treatment of alkenyl boronate *Z*-**4c** (30 mg, 0.071 mmol, 1.0 equiv) in presence of [Ir{dFCF$_3$ppy}$_2$(bpy)]PF$_6$ (0.72 mg, 0.00072 mmol, 1 mol%) in THF afforded the titled compound as an oil after filtration through a pad of silica gel (30 mg, >99%). **^{1}H-RMN** (300 MHz, CDCl$_3$): δ 7.34 (d, J = 8.3 Hz, 2H), 7.02 (d, J = 8.3 Hz, 2H), 3.68 (s, 3H), 3.62 (s, 2H), 2.34 – 2.25 (m, 2H), 1.55 – 1.42 (m, 2H), 1.35 (s, 12H), 0.92 (t, J = 7.3 Hz, 3H). **^{13}C-RMN** (75 MHz, CDCl$_3$): δ 170.5, 138.6, 133.8, 131.4, 130.1, 119.8, 83.6, 52.6, 34.2, 31.7, 25.1, 21.7, 14.7. **HRMS** (ESI$^+$) m/z [M+H]$^+$ calcd for $C_{20}H_{29}BBrO_4$ 423.1337, found 423.1337.

Methyl (*E*)-2-(2,3-difluorobenzyl)-3-(4,4,5,5-tetramethyl-1,3,2-dioxaborolan-2-yl)hex-2-enoate (*E*-4d):

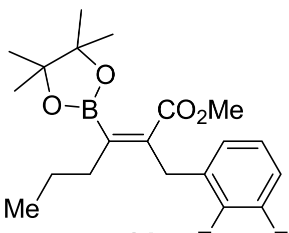

E-**4d**

Following the general procedure for the *photoisomerization reaction*, the treatment of alkenyl boronate *Z*-**4d** (28.9 mg, 0.076 mmol, 1.0 equiv) in presence of [Ir{dFCF$_3$ppy}$_2$(bpy)]PF$_6$ (0.77 mg, 0.00077 mmol, 1 mol%) in THF afforded the titled compound as an oil after filtration through a pad of silica gel (26.6 mg, 92%). **^{1}H-RMN** (300 MHz, CDCl$_3$): δ 7.11 – 6.69 (m, 3H), 3.70 (s, 5H), 2.37 – 2.20 (m, 2H), 1.54 – 1.43 (m, 2H), 1.36 (s, 12H), 0.92 (t, J = 7.3 Hz, 3H). **^{13}C-RMN** (75 MHz, CDCl$_3$): δ 170.30, 151.4 (dd, J = 118.6, 13.4), 148.12 (dd, J = 118.6, 13.2), 132.5, 129.01 (d, J = 12.4 Hz), 124.52 (t, J = 3.2 Hz), 123.68 (dd, J = 6.9, 4.7 Hz), 114.96 (d, J = 17.2 Hz), 83.6, 52.6, 34.2, 25.1, 24.9 – 24.7 (m), 21.6, 14.6. **HRMS** (ESI$^+$) m/z [M+H]$^+$ calcd for $C_{20}H_{28}BF_2O_4$ 381.2043, found 381.2042.

Methyl (*E*)-2-(2-cyanobenzyl)-3-(4,4,5,5-tetramethyl-1,3,2-dioxaborolan-2-yl)hex-2-enoate (*E*-4e):

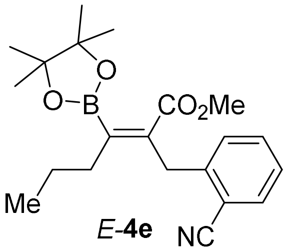

E-**4e**

Following the general procedure for the *photoisomerization reaction*, the treatment of alkenyl boronate *Z*-**4e** (29.5 mg, 0.080 mmol, 1.0 equiv) in presence of [Ir{dFCF$_3$ppy}$_2$(bpy)]PF$_6$ (0.81 mg, 0.00081 mmol, 1 mol%) in THF afforded the titled compound as an oil after filtration through a pad of silica gel (26.6 mg, 90%). **^{1}H-RMN** (300 MHz, CDCl$_3$): δ 7.61 (d, J = 7.7 Hz, 1H), 7.48 – 7.39 (m, 1H), 7.25 (t, J = 7.6 Hz, 1H), 7.16 (d, J = 7.8 Hz, 1H), 3.90 (s, 2H), 3.68 (s, 3H), 2.37 – 2.24 (m, 2H), 1.54 – 1.47 (m, 2H), 1.36 (s, 12H), 0.92 (t, J = 7.3 Hz, 3H). **^{13}C-RMN** (75 MHz, CDCl$_3$): δ 170.0, 168.4, 143.4, 132.9, 132.8, 128.5, 126.6, 118.2, 112.7, 83.7, 52.7, 34.3, 30.6, 25.1, 21.7, 14.6. **HRMS** (ESI$^+$) m/z [M+H]$^+$ calcd for $C_{21}H_{29}BNO_4$ 370.2184, found 370.2192.

Methyl (*E*)-2-(naphthalen-1-ylmethyl)-3-(4,4,5,5-tetramethyl-1,3,2-dioxaborolan-2-yl)hex-2-enoate (*E*-4f):

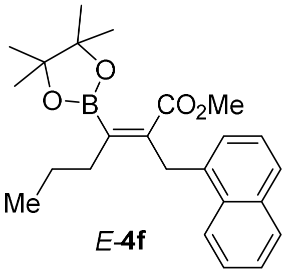

E-**4f**

Following the general procedure for the *photoisomerization reaction*, the treatment of alkenyl boronate *Z*-**4f** (31.2 mg, 0.079 mmol, 1.0 equiv) in presence of [Ir{dFCF$_3$ppy}$_2$(bpy)]PF$_6$ (0.80 mg, 0.00080 mmol, 1 mol%) in THF afforded the titled compound as an oil after filtration through a pad of silica gel (27.8 mg, 89%). **^{1}H-RMN** (300 MHz, CDCl$_3$): δ 8.12 (d, J = 8.2 Hz, 1H), 7.87 (d, J = 9.0 Hz, 1H), 7.70 (d, J = 8.2 Hz, 1H), 7.60 – 7.45 (m, 2H), 7.39 – 7.29 (m, 1H), 7.19 – 7.12 (m, 1H), 4.13 (s, 2H), 3.64 (s, 3H), 2.29 – 2.19 (m, 2H), 1.58-1.46 (m, 2H), 1.40 (s, 12H), 0.87 (t, J = 7.3 Hz, 3H). **^{13}C-RMN** (75 MHz, CDCl$_3$): δ 170.9, 134.7, 133.8, 133.5, 132.1, 128.9, 126.7, 126.0, 125.8, 125.6, 124.2, 123.4, 83.6, 52.6, 34.2, 28.7, 25.2, 21.7, 14.7. **HRMS** (ESI$^+$) m/z [M+H]$^+$ calcd for $C_{24}H_{32}BO_4$ 395.2388, found 395.2384.

Methyl (*E*)-3-(4,4,5,5-tetramethyl-1,3,2-dioxaborolan-2-yl)-2-(2,4,6-trimethylbenzyl)hex-2-enoate (*E*-4g):

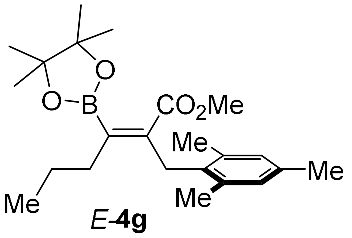

E-**4g**

Following the general procedure for the *photoisomerization reaction*, the treatment of alkenyl boronate *Z*-**4g** (25.5 mg, 0.066 mmol, 1.0 equiv) in presence of [Ir{dFCF$_3$ppy}$_2$(bpy)]PF$_6$ (0.67 mg, 0.00067 mmol, 1 mol%) in THF afforded a mixture of *E*- and *Z*-isomers of the titled compound (*E:Z* = 70:30). The titled compound was further purified by column chromatography (n-hexane:toluene:AcOEt 15:15:1) to afford the spectroscopically pure *E*-isomer (*E:Z > 98:2)* as an oil (15 mg, 69%). ^{1}**H-RMN** (300 MHz, CDCl$_3$): δ 6.76 (s, 2H), 3.63 (s, 3H), 3.59 (s, 2H), 2.30-2.18 (m, 11H), 1.45-1.36 (m, 2H), 1.30 (s, 12H), 0.84 (t, J = 7.2 Hz, 3H). ^{13}**C-RMN** (75 MHz, CDCl$_3$): δ 172.1, 137.1, 135.3, 135.1, 133.0, 129.0, 83.1, 52.4, 33.4, 27.9, 25.1, 21.6, 20.9, 20.7, 14.6. **HRMS** (ESI$^+$) m/z [M+H]$^+$ calcd for $C_{23}H_{36}BO_4$ 387.2701, found 387.2714.

(*S*)-(4-(prop-1-en-2-yl)cyclohex-1-en-1-yl)methyl (*E*)-2-benzyl-3-(4,4,5,5-tetramethyl-1,3,2-dioxaborolan-2-yl)hex-2-enoate (*E*-4i):

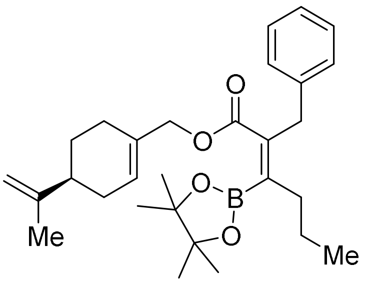

E-**4i**

Following the general procedure for the *photoisomerization reaction*, the treatment of alkenyl boronate *Z*-**4i** (33.9 mg, 0.073 mmol, 1.0 equiv) in presence of [Ir{dFCF$_3$ppy}$_2$(bpy)]PF$_6$ (0.74 mg, 0.00074 mmol, 1 mol%) in THF afforded the titled compound as an oil after filtration through a pad of silica gel (32.9 mg, 97%). ^{1}**H-RMN** (300 MHz, CDCl$_3$): δ 7.26 – 7.07 (m, 5H), 5.56 (s, 1H), 4.74 – 4.69 (m, 1H), 4.68 (s, 1H), 4.46 (s, 2H), 3.68 (s, 2H), 2.42 – 2.29 (m, 2H), 2.14 – 1.62 (m, 10H), 1.56 – 1.49 (m, 2H), 1.35 (s, 12H), 0.93 (t, J = 7.3 Hz, 3H). ^{13}**C-RMN** (75 MHz, CDCl$_3$): δ 170.8, 149.8, 139.7, 134.4, 132.4, 128.5, 128.3, 126.0, 125.9, 108.8, 83.3, 69.7, 40.9, 34.0, 32.2, 30.5, 27.4, 26.2, 25.1, 21.8, 20.9, 14.7. **HRMS** (ESI$^+$) m/z [M+H]$^+$ calcd for $C_{29}H_{42}BO_4$ 465.3171, found 465.3173.

But-3-en-1-yl (*E*)-2-benzyl-3-(4,4,5,5-tetramethyl-1,3,2-dioxaborolan-2-yl)hex-2-enoate (*E*-4j):

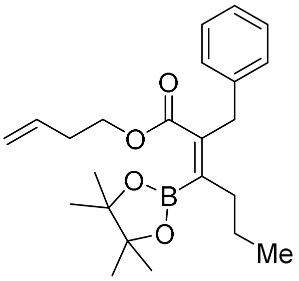

E-**4j**

Following the general procedure for the *photoisomerization reaction*, the treatment of alkenyl boronate *Z*-**4j** (35 mg, 0.091 mmol, 1.0 equiv) in presence of [Ir{dFCF$_3$ppy}$_2$(bpy)]PF$_6$ (0.92 mg, 0.00092 mmol, 1 mol%) in THF afforded the titled compound as an oil after filtration through a pad of silica gel (34.3 mg, 98%). ^{1}**H-RMN** (300 MHz, CDCl$_3$): δ 7.25 – 7.12 (m, 5H), 5.60 (ddt, J = 17.0, 10.2, 6.7 Hz, 1H), 5.07 – 4.91 (m, 2H), 4.13 (t, J = 6.7 Hz, 2H), 3.66 (s, 2H), 2.40 – 2.23 (m, 4H), 1.55 – 1.46 (m, 2H), 1.35 (s, 12H), 0.93 (t, J = 7.3 Hz, 3H). ^{13}**C-RMN** (75 MHz, CDCl$_3$): δ 170.7, 139.7, 134.5, 134.0, 128.5, 128.3, 125.9, 117.2, 83.3, 64.8, 34.1, 33.0, 32.1, 25.1, 21.7, 14.7. **HRMS** (ESI$^+$) m/z [M+H]$^+$ calcd for $C_{23}H_{34}BO_4$ 385.2545, found 385.2543.

5-(Trimethylsilyl)pent-4-yn-1-yl (*E*)-2-benzyl-3-(4,4,5,5-tetramethyl-1,3,2-dioxaborolan-2-yl)hex-2-enoate (*E*-4k):

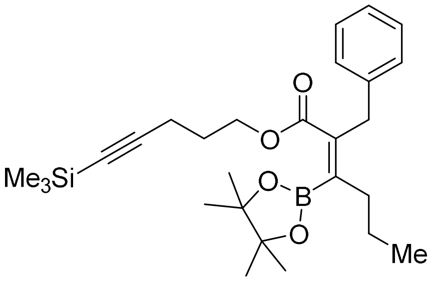

E-**4k**

Following the general procedure for the *photoisomerization reaction*, the treatment of alkenyl boronate *Z*-**4k** (35.1 mg, 0.075 mmol, 1.0 equiv) in presence of [Ir{dFCF$_3$ppy}$_2$(bpy)]PF$_6$ (0.76 mg, 0.00076 mmol, 1 mol%) in THF afforded the titled compound as an oil after filtration through a pad of silica gel (32.3 mg, 92%). ^{1}**H-RMN** (300 MHz, CDCl$_3$): δ 7.25 – 7.13 (m, 5H), 4.16 (t, J = 6.1 Hz, 2H), 3.66 (s, 2H), 2.37 – 2.30 (m, 2H), 2.02 (h, J = 7.4, 6.6 Hz, 2H), 1.70 (p, J = 6.6 Hz, 2H), 1.55 – 1.47 (m, 2H), 1.35 (s, 12H), 0.93 (t, J = 7.3 Hz, 3H), 0.14 (s, 9H). ^{13}**C-RMN** (75 MHz, CDCl$_3$): δ 170.6, 139.6, 134.4, 128.4, 126.0,

106.0, 85.2, 83.4, 64.2, 34.1, 32.2, 28.1, 27.7, 25.1, 21.7, 16.5, 14.7, 0.2. **HRMS** (ESI^+) m/z $[M+H]^+$ calcd for $C_{27}H_{42}BO_4Si$ 469.2940, found 469.2935.

Methyl (*E*)-2-benzyl-6-(2-(1-(4-chlorobenzoyl)-5-methoxy-2-methyl-1H-indol-3-yl)acetoxy)-3-(4,4,5,5-tetramethyl-1,3,2-dioxaborolan-2-yl)hex-2-enoate (*E*-4n):

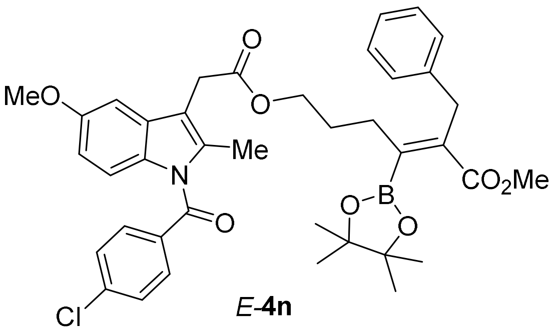

E-**4n**

Following the general procedure for the *photoisomerization reaction*, the treatment of alkenyl boronate *Z*-**4n** (62.3 mg, 0.089 mmol, 1.0 equiv) in presence of $[Ir\{dFCF_3ppy\}_2(bpy)]PF_6$ (0.90 mg, 0.00090 mmol, 1 mol%) in THF afforded the titled compound as yellow solid after filtration through a pad of silica gel (53.6 mg, 86%). **^{1}H-RMN** (300 MHz, $CDCl_3$): δ 7.61 (d, J = 8.6 Hz, 2H), 7.45 (d, J = 8.6 Hz, 2H), 7.20 – 7.05 (m, 5H), 6.93 (d, J = 2.5 Hz, 1H), 6.82 (d, J = 9.0 Hz, 1H), 6.64 (dd, J = 9.0, 2.5 Hz, 1H), 4.09 (t, J = 6.3 Hz, 2H), 3.80 (s, 3H), 3.71 (s, 3H), 3.62 (s, 2H), 3.58 (s, 2H), 2.38 (t, J = 7.1 Hz, 2H), 2.34 (s, 3H), 1.85 – 1.75 (m, 2H), 1.32 (s, 12H). **^{13}C-RMN** (75 MHz, $CDCl_3$): δ 170.9, 170.8, 168.4, 156.2, 139.3, 139.2, 136.0, 135.3, 134.1, 131.3, 130.9, 130.8, 129.2, 128.5, 128.3, 126.1, 115.1, 112.7, 111.8, 101.4, 83.6, 64.8, 55.8, 52.8, 32.1, 30.5, 28.2, 27.3, 25.1, 13.5. **HRMS** (ESI^+) m/z $[M+H]^+$ calcd for $C_{39}H_{44}BClNO_8$ 700.2843, found 700.2850.

Methyl (*E*)-2-phenyl-3-(4,4,5,5-tetramethyl-1,3,2-dioxaborolan-2-yl)hex-2-enoate (*E*-5a):

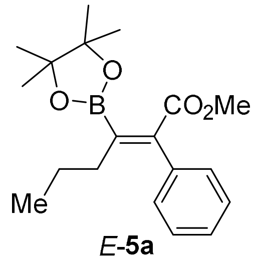

E-**5a**

Following the general procedure for the *photoisomerization reaction*, the treatment of alkenyl boronate *Z*-**5a** (30 mg, 0.091 mmol, 1.0 equiv) in presence of $[Ir\{dFCF_3ppy\}_2(bpy)]PF_6$ (0.92 mg, 0.00092 mmol, 1 mol%) in THF afforded the titled compound as an oil after filtration through a pad of silica gel (29.1 mg, 97%). **^{1}H-RMN** (300 MHz, $CDCl_3$): δ 7.38-7.25 (m, 3H), 7.17 – 7.09 (m, 2H), 3.71 (s, 3H), 2.13 – 2.06 (m, 2H), 1.52-1.42 (m, 2H), 1.43 (s, 12H), 0.80 (t, J = 7.3 Hz, 3H). **^{13}C-RMN** (75 MHz, $CDCl_3$): δ 170.4, 137.6, 135.9, 129.7, 128.1, 127.4, 83.7, 52.9, 34.7, 25.2, 21.8, 14.5. **^{11}B NMR** (96 MHz, $CDCl_3$) δ 30.0. **HRMS** (ESI^+) m/z $[M+H]^+$ calcd for $C_{19}H_{28}BO_4$ 331.2075, found 331.2080.

Methyl (*E*)-2-(4-methoxyphenyl)-3-(4,4,5,5-tetramethyl-1,3,2-dioxaborolan-2-yl)hex-2-enoate (*E*-5b):

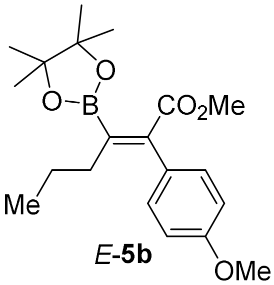

E-**5b**

Following the general procedure for the *photoisomerization reaction*, the treatment of alkenyl boronate *Z*-**5b** (31.3 mg, 0.087 mmol, 1.0 equiv) in presence of $[Ir\{dFCF_3ppy\}_2(bpy)]PF_6$ (0.88 mg, 0.00088 mmol, 1 mol%) in THF afforded the titled compound as an oil after filtration through a pad of silica gel (31.3 mg, >99%). **^{1}H-RMN** (300 MHz, $CDCl_3$): δ 7.04 (d, J = 8.6 Hz, 2H), 6.88 (d, J = 8.6 Hz, 2H), 3.81 (s, 3H), 3.72 (s, 3H), 2.15 – 2.05 (m, 2H), 1.49-1.42 (m, 2H), 1.39 (s, 12H), 0.81 (t, J = 7.3 Hz, 3H). **^{13}C-RMN** (75 MHz, $CDCl_3$): δ 170.9, 158.8, 137.1, 130.8, 127.9, 113.5, 83.6, 55.3, 53.0, 34.7, 25.2, 21.8, 14.6. **HRMS** (ESI^+) m/z $[M+H]^+$ calcd for $C_{20}H_{30}BO_5$ 361.2181, found 361.2180.

Methyl (*E*)-2-(9-phenyl-9*H*-carbazol-3-yl)-3-(4,4,5,5-tetramethyl-1,3,2-dioxaborolan-2-yl)hex-2-enoate (*Z*-5c):

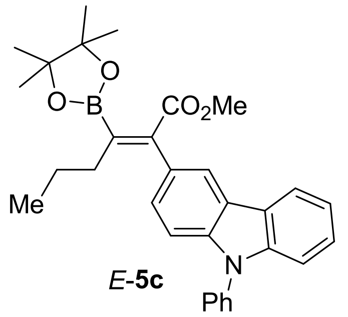

E-**5c**

Following the general procedure for the *photoisomerization reaction*, the treatment of alkenyl boronate *Z*-**5c** (44.6 mg, 0.090 mmol, 1.0 equiv) in presence of [Ir{dFCF$_3$ppy}$_2$(bpy)]PF$_6$ (0.91 mg, 0.00091 mmol, 1 mol%) in THF afforded the titled compound as an oil after filtration through a pad of silica gel (41.5 mg, 93%). **^{1}H-NMR** (300 MHz, CDCl$_3$): δ 8.10 (d, J = 8.5 Hz, 1H), 7.94 – 7.88 (m, 1H), 7.63 – 7.42 (m, 9H), 7.18 (dd, J = 8.4, 1.6 Hz, 1H), 3.74 (s, 3H), 2.22 – 2.16 (m, 2H), 1.56-1.47 (m, 2H), 1.43 (s, 12H), 0.82 (t, J = 7.3 Hz, 3H). **^{13}C-NMR** (75 MHz, CDCl$_3$): δ 171.0, 141.2, 140.2, 138.1, 137.8, 130.0, 127.7, 127.6, 127.4, 127.2, 126.2, 123.4, 123.2, 121.4, 120.4, 120.1, 110.0, 109.4, 83.7, 53.0, 34.9, 25.2, 21.9, 14.6. **HRMS** (ESI$^+$) m/z [M+H]$^+$ calcd for $C_{31}H_{35}BNO_4$ 496.2654, found 496.2659.

Methyl (*E*)-2-(4-(4-fluorophenoxy)phenyl)-3-(4,4,5,5-tetramethyl-1,3,2-dioxaborolan-2-yl)hex-2-enoate (*E*-5d):

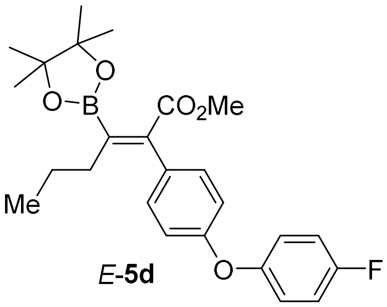

E-**5d**

Following the general procedure for the *photoisomerization reaction*, the treatment of alkenyl boronate *Z*-**5d** (35.7 mg, 0.081 mmol, 1.0 equiv) in presence of [Ir{dFCF$_3$ppy}$_2$(bpy)]PF$_6$ (0.82 mg, 0.00082 mmol, 1 mol%) in THF afforded the titled compound as an oil after filtration through a pad of silica gel (30.7 mg, 86%). **^{1}H-NMR** (300 MHz, CDCl$_3$): δ 7.33 – 7.26 (m, 1H), 7.10 – 6.94 (m, 4H), 6.93 (ddd, J = 8.2, 2.5, 0.9 Hz, 1H), 6.83 (dt, J = 7.6, 1.2 Hz, 1H), 6.68 (dd, J = 2.5, 1.5 Hz, 1H), 3.71 (s, 3H), 2.11 – 2.04 (m, 2H), 1.52-1.46 (m, 2H), 1.38 (s, 12H), 0.81 (t, J = 7.3 Hz, 3H). **^{13}C-NMR** (75 MHz, CDCl$_3$): δ 170.0, 159.1 (d, J = 242.0 Hz), 157.7, 152.7 (d, J = 2.6 Hz), 137.0, 129.5, 124.3, 121.0 (d, J = 8.3 Hz), 119.2, 116.5 (d, J = 23.3 Hz), 83.8, 52.9, 34.8, 25.2, 21.8, 14.6. **^{19}F-NMR** (75 MHz, CDCl$_3$): δ -119.82. **HRMS** (ESI$^+$) m/z [M+H]$^+$ calcd for $C_{25}H_{31}BFO_5$ 441.2243, found 441.2245.

Methyl (*Z*)-3-(4,4,5,5-tetramethyl-1,3,2-dioxaborolan-2-yl)-2-(thiophen-2-yl)hex-2-enoate (*E*-5e):

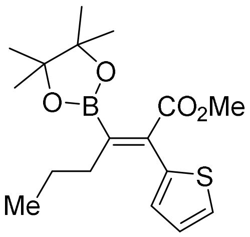

E-**5e**

Following the general procedure for the *photoisomerization reaction*, the treatment of alkenyl boronate *Z*-**5e** (24.2 mg, 0.072 mmol, 1.0 equiv) in presence of [Ir{dFCF$_3$ppy}$_2$(bpy)]PF$_6$ (0.73 mg, 0.00073 mmol, 1 mol%) in THF afforded a mixture of *E*- and *Z*-isomers of the titled compound (*E:Z* = 66:34). The titled compound was further purified by column chromatography (n-hexane:toluene:AcOEt 15:15:1) to afford the spectroscopically pure *E*-isomer (*E:Z* > *98:2)* as an oil (12.6 mg, 52%). **^{1}H-NMR** (300 MHz, CDCl$_3$): δ 7.33 (dd, J = 5.1, 1.1, 1H), 7.02 (dd, J = 5.1, 3.5 Hz, 1H), 6.84 (dd, J = 3.5, 1.1, 1H), 3.76 (s, 3H), 2.29 – 2.17 (m, 2H), 1.53 – 1.46 (m, 2H), 1.38 (s, 12H), 0.88 (d, J = 7.3 Hz, 3H). **^{13}C-NMR** (75 MHz, CDCl$_3$): δ 170.1, 135.8, 130.3, 128.0, 126.7, 126.1, 83.8, 53.2, 35.2, 25.2, 22.0, 14.6. **HRMS** (ESI$^+$) m/z [M+H]$^+$ calcd for $C_{17}H_{26}BO_4S$ 337.1639, found 337.1640.

Ethyl (*E*)-3-(1-methoxy-1-oxo-3-(4,4,5,5-tetramethyl-1,3,2-dioxaborolan-2-yl)hex-2-en-2-yl)benzoate (*E*-5g):

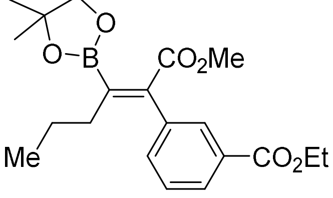

E-**5g**

Following the general procedure for the *photoisomerization reaction*, the treatment of alkenyl boronate *Z*-**5g** (28.2 mg, 0.070 mmol, 1.0 equiv) in presence of [Ir{dFCF$_3$ppy}$_2$(bpy)]PF$_6$ (0.71 mg, 0.00071 mmol, 1 mol%) in THF afforded the titled compound as an oil after filtration through a pad of silica gel (28.2 mg, >99%). **^{1}H-NMR** (300 MHz, CDCl$_3$): δ 7.99 – 7.94 (m, 1H), 7.81 (bs, 1H), 7.41 (t, J = 7.7 Hz, 1H), 7.31 – 7.26 (m, 1H), 4.38 – 4.30 (m, 2H), 3.68 (s, 3H), 2.09 – 2.00 (m, 2H), 1.50-1.42 (m, 2H), 1.38 (s, 12H), 1.36-1.29 (m, 3H), 0.78 (t, J = 7.3 Hz, 3H). **^{13}C-NMR** (75 MHz, CDCl$_3$): δ 169.7, 166.5, 136.7, 136.1, 134.0, 130.8, 130.4,

128.6, 128.2, 83.8, 61.0, 52.9, 34.7, 25.1, 21.6, 14.4, 14.3. **HRMS** (ESI^+) m/z $[M+H]^+$ calcd for $C_{22}H_{32}BO_6$ 403.2286, found 403.2286.

Methyl (*E*)-3-(4,4,5,5-tetramethyl-1,3,2-dioxaborolan-2-yl)-2-(4-(trifluoromethyl)phenyl)hex-2-enoate (*E*-5h):

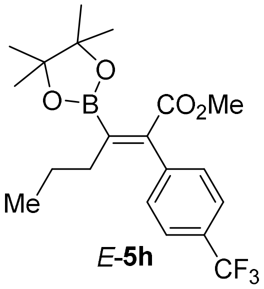

E-**5h**

Following the general procedure for the *photoisomerization reaction*, the treatment of alkenyl boronate *Z*-**5h** (24.7 mg, 0.062 mmol, 1.0 equiv) in presence of [Ir{dFCF$_3$ppy}$_2$(bpy)]PF$_6$ (0.63 mg, 0.00063 mmol, 1 mol%) in THF afforded the titled compound as an oil after filtration through a pad of silica gel (23.7 mg, 96%). **^{1}H-NMR** (300 MHz, $CDCl_3$): δ 7.61 (d, *J* = 7.9 Hz, 2H), 7.23 (d, *J* = 7.9 Hz, 2H), 3.71 (s, 3H), 2.11 – 2.01 (m, 2H), 1.46 (dd, *J* = 15.6, 7.6 Hz, 2H), 1.40 (s, 12H), 0.81 (t, *J* = 7.3 Hz, 3H). **^{13}C-NMR** (75 MHz, $CDCl_3$): δ 169.5, 139.8, 136.4, 130.1, 129.8, 125.1 (q, *J* = 3.7 Hz), 124.1 (q, J = 271.9 Hz), 84.0, 53.0, 34.8, 25.1, 21.7, 14.5. **HRMS** (ESI^+) m/z $[M+H]^+$ calcd for $C_{20}H_{27}BF_3O_4$ 399.1949, found 399.1960.

3-(Naphthalen-1-yl)prop-2-yn-1-yl (*E*)-2-phenyl-3-(4,4,5,5-tetramethyl-1,3,2-dioxaborolan-2-yl)hex-2-enoate (*E*-5i):

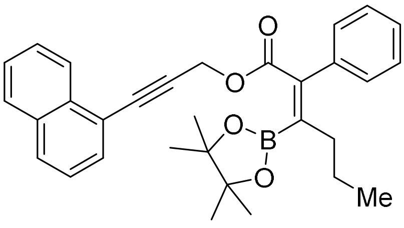

E-**5i**

Following the general procedure for the *photoisomerization reaction*, the treatment of alkenyl boronate *Z*-**5i** (36 mg, 0.075 mmol, 1.0 equiv) in presence of [Ir{dFCF$_3$ppy}$_2$(bpy)]PF$_6$ (0.76 mg, 0.00076 mmol, 1 mol%) in THF afforded the titled compound as an oil after filtration through a pad of silica gel (35.3 mg, 98%). **^{1}H-NMR** (300 MHz, $CDCl_3$): δ 8.22 (d, *J* = 7.8 Hz, 1H), 7.85 – 7.80 (m, 2H), 7.63 (d, *J* = 7.1 Hz, 1H), 7.53 (td, *J* = 7.1, 1.5 Hz, 2H), 7.41 – 7.33 (m, 4H), 7.24 – 7.20 (m, 2H), 5.11 (s, 2H), 2.19-2.12 (m, 2H), 1.53 – 1.46 (m, 2H), 1.43 (s, 12H), 0.84 (t, *J* = 7.3 Hz, 3H). **^{13}C-NMR** (75 MHz, $CDCl_3$): δ 168.9, 137.6, 135.6, 133.5, 133.1, 130.8, 129.8, 129.2, 128.3, 128.1, 127.4, 127.0, 126.5, 126.3, 125.2, 88.0, 84.6, 83.9, 54.0, 34.9, 25.2, 21.8, 14.5. **HRMS** (ESI^+) m/z $[M+H]^+$ calcd for $C_{31}H_{34}BO_4$ 481.2545, found 481.2552.

Methyl (*E*)-2-phenyl-3-(4,4,5,5-tetramethyl-1,3,2-dioxaborolan-2-yl)-3-(trimethylsilyl)acrylate (*E*-5k):

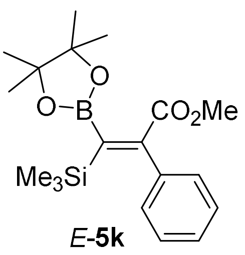

E-**5k**

Following the general procedure for the *photoisomerization reaction*, the treatment of alkenyl boronate *Z*-**5k** (33.5 mg, 0.093 mmol, 1.0 equiv) in presence of [Ir{dFCF$_3$ppy}$_2$(bpy)]PF$_6$ (0.94 mg, 0.00094 mmol, 1 mol%) in THF afforded the titled compound as a mixture of *E*- and *Z*-isomers (*E*:*Z* = 90:10) as an oil after filtration through a pad of silica gel (33.5 mg, >99%). Spectroscopic data for *E*-**5k***:* **^{1}H-NMR** (300 MHz, $CDCl_3$): δ 7.32 – 7.28 (m, 3H), 7.20 – 7.12 (m, 2H), 3.66 (s, 3H), 1.42 (s, 12H), -0.15 (s, 9H). **^{13}C-NMR** (75 MHz, $CDCl_3$): δ 168.5, 149.6, 139.4, 129.5, 127.7, 127.6, 83.8, 52.9, 25.9. **HRMS** (ESI^+) m/z $[M+H]^+$ calcd for $C_{19}H_{30}BO_4Si$ 361.2001, found 361.2015.

(*8R,9S,13S,14S*)-13-methyl-17-oxo-7,8,9,11,12,13,14,15,16,17-decahydro-6*H*-cyclopenta[a]phenanthren-3-yl (*E*)-2-phenyl-3-(4,4,5,5-tetramethyl-1,3,2-dioxaborolan-2-yl)hex-2-enoate (*E*-5l):

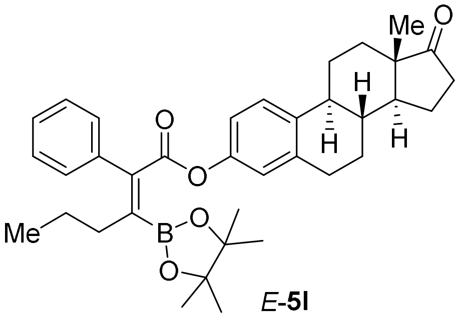

E-**5l**

Following the general procedure for the *photoisomerization reaction*, the treatment of alkenyl boronate *Z*-**5l** (45.5 mg, 0.080 mmol, 1.0 equiv) in presence of [Ir{dFCF$_3$ppy}$_2$(bpy)]PF$_6$ (0.80 mg, 0.00080 mmol, 1 mol%) in THF afforded the titled compound as an oil after filtration through a pad of silica gel (44.1 mg, 97%). **^{1}H-NMR** (300 MHz, CDCl$_3$): δ 7.45 – 7.28 (m, 4H), 7.24 – 7.18 (m, 2H), 6.84 (dd, J = 8.4, 2.3 Hz, 1H), 6.77 (d, J = 2.2 Hz, 1H), 2.84 (dd, J = 8.9, 4.2 Hz, 2H), 2.49 (dd, J = 18.6, 8.6 Hz, 1H), 2.41 – 2.34 (m, 1H), 2.28 – 2.21 (m, 1H), 2.19 – 2.11 (m, 2H), 2.09 – 1.90 (m, 4H), 1.66 – 1.43 (m, 8H), 1.34 (s, 12H), 0.89 (s, 3H), 0.84 (t, J = 7.3 Hz, 3H). **^{13}C-NMR** (75 MHz, CDCl$_3$): δ 220.1, 167.5, 148.9, 138.1, 137.7, 137.1, 136.0, 129.6, 128.1, 127.4, 126.1, 121.5, 118.8, 84.0, 50.5, 48.0, 44.2, 38.1, 35.9, 35.2, 31.6, 29.4, 26.4, 25.8, 25.1, 24.7, 21.7, 14.5, 13.9. **HRMS** (ESI$^+$) m/z [M+H]$^+$ calcd for $C_{36}H_{46}BO_5$ 569.3433, found 569.3458.

Methyl (*E*)-6-(2-(1-(4-chlorobenzoyl)-5-methoxy-2-methyl-1*H*-indol-3-yl)acetoxy)-2-phenyl-3-(4,4,5,5-tetramethyl-1,3,2-dioxaborolan-2-yl)hex-2-enoate (*E*-5m):

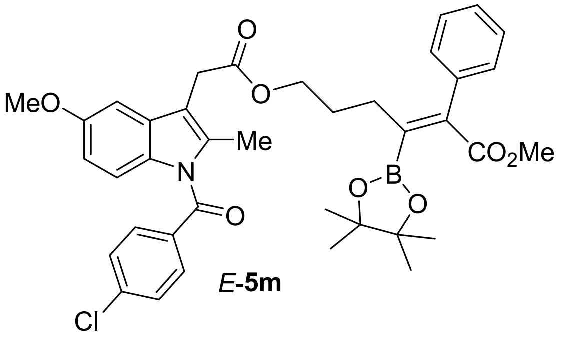

E-**5m**

Following the general procedure for the *photoisomerization reaction*, the treatment of alkenyl boronate *Z*-**5m** (62.4 mg, 0.091 mmol, 1.0 equiv) in presence of [Ir{dFCF$_3$ppy}$_2$(bpy)]PF$_6$ (0.92 mg, 0.00092 mmol, 1 mol%) in THF afforded the titled compound as an oil after filtration through a pad of silica gel (59.3 mg, 95%). **^{1}H-RMN** (300 MHz, CDCl$_3$): δ 7.64 (d, J = 8.5 Hz, 2H), 7.46 (d, J = 8.5 Hz, 2H), 7.41 – 7.27 (m, 4H), 7.14 – 7.07 (m, 2H), 6.89 – 6.86 (m, 1H), 6.65 (dd, J = 9.0, 2.5 Hz, 1H), 3.97 (t, J = 6.5 Hz, 2H), 3.80 (s, 3H), 3.72 (s, 3H), 3.51 (s, 2H), 2.29 (s, 3H), 2.24 – 2.15 (m, 2H), 1.78 (dd, J = 9.2, 6.5 Hz, 2H), 1.37 (s, 12H). **^{13}C-RMN** (75 MHz, CDCl$_3$): δ 170.7, 170.4, 168.3, 156.1, 139.3, 138.3, 135.9, 135.3, 134.0, 131.2, 130.8, 130.7, 129.4, 129.2, 128.2, 127.6, 115.0, 112.6, 111.7, 101.3, 83.6, 64.7, 55.7, 53.1, 31.0, 28.7, 27.3, 25.1, 13.4. **HRMS** (ESI +) m/z [M+H]$^+$ calcd for $C_{38}H_{42}BClNO_8$ 686.2687, found 686.2694.

(*E*)-6-methoxy-6-oxo-5-phenyl-4-(4,4,5,5-tetramethyl-1,3,2-dioxaborolan-2-yl)hex-4-en-1-yl (*2S,4aR,7S,9aS,10S*)-1-methyl-8-methylene-13-oxo-2,7-bis((triethylsilyl)oxy)-1,2,4b,5,6,7,8,9,10,10a-decahydro-4a,1-(epoxymethano)-7,9a-methanobenzo[a]azulene-10-carboxylate (*E*-5n):

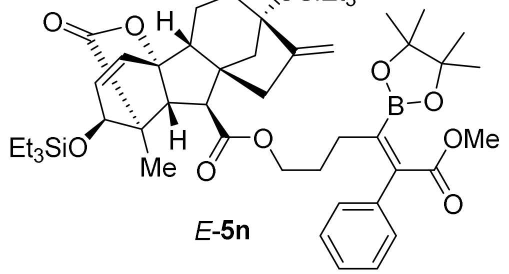

E-**5n**

Following the general procedure for the *photoisomerization reaction*, the treatment of alkenyl boronate *Z*-**5n** (61.4 mg, 0.068 mmol, 1.0 equiv) in presence of [Ir{dFCF$_3$ppy}$_2$(bpy)]PF$_6$ (0.69 mg, 0.00069 mmol, 1 mol%) in THF afforded the titled compound as an oil after filtration through a pad of silica gel (57.7 mg, 94%). **^{1}H-NMR** (300 MHz, CDCl$_3$): δ 7.38 – 7.30 (m, 3H), 7.10 (dd, J = 7.6, 1.7 Hz, 2H), 6.20 (d, J = 9.3 Hz, 1H), 5.74 (dd, J = 9.3, 3.6 Hz, 1H), 5.18 (s, 1H), 4.83 (s, 1H), 4.14 – 4.09 (m, 2H), 3.91-3.80 (m, 1H), 3.72 (s, 3H), 3.22 (d, J = 10.8 Hz, 1H), 2.56 (d, J = 10.8 Hz, 1H), 2.21 – 2.13 (m, 2H), 1.91 – 1.70 (m, 11H), 1.39 (s, 12H), 1.10 (s, 3H), 0.97 – 0.90 (m, 18H), 0.57 (dd, J = 15.7, 7.9 Hz, 12H). **^{13}C-NMR** (75 MHz, CDCl$_3$): δ 179.2, 171.9, 170.3, 157.1, 138.7, 135.3, 133.2, 131.5, 129.4, 128.3, 127.7, 107.4, 91.0, 83.8, 79.3, 70.1, 64.5, 54.2, 53.1, 52.8, 50.9, 50.7, 44.3,

42.9, 40.7, 31.0, 28.6, 27.4, 25.1, 17.1, 15.0, 7.2, 6.9, 6.5, 5.1. **HRMS** (ESI^+) m/z $[M+H]^+$ calcd for $C_{50}H_{76}BO_{10}Si_2$ 903.5065, found 903.5084.

***rac*-(*R*)-2,5,7,8-tetramethyl-2-((*4R,8R*)-4,8,12-trimethyltridecyl)chroman-6-yl (*E*)-2-phenyl-3-(4,4,5,5-tetramethyl-1,3,2-dioxaborolan-2-yl)hex-2-enoate (*E*-5o):**

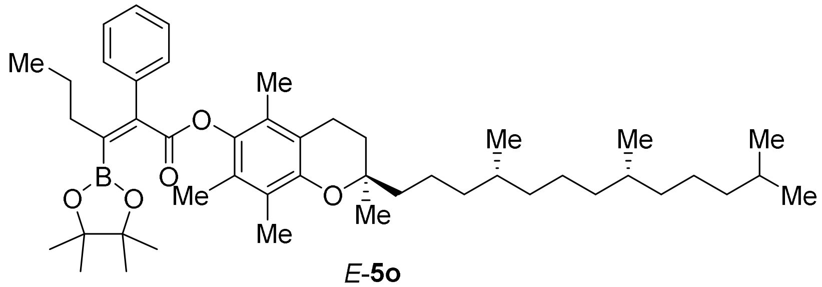

E-**5o**

Following the general procedure for the *photoisomerization reaction*, the treatment of alkenyl boronate *Z*-**5o** (53.9 mg, 0.074 mmol, 1.0 equiv) in presence of [Ir{dFCF$_3$ppy}$_2$(bpy)]PF$_6$ (0.75 mg, 0.00075 mmol, 1 mol%) in THF afforded the titled compound as an oil after filtration through a pad of silica gel (53.9 mg, >99%). **^{1}H-RMN** (300 MHz, $CDCl_3$): δ 7.44 – 7.26 (m, 5H), 2.54 (t, J = 6.3 Hz, 2H), 2.17 – 2.11 (m, 2H), 2.05 (s, 3H), 1.94 (s, 3H), 1.90 (s, 3H), 1.62-1.42 (m, 10H), 1.31 (s, 12H), 1.28-1.10 (m, 15H), 0.89-0.78 (m, 18H). **^{13}C-RMN** (75 MHz, $CDCl_3$): δ 167.0, 149.3, 141.0, 138.2, 136.6, 129.6, 128.1, 127.3, 127.0, 125.2, 122.9, 117.3, 83.9, 75.1, 39.5, 37.7, 37.6, 37.5, 37.4, 35.3, 32.9, 32.8, 28.1, 25.2, 24.9, 24.6, 24.6, 22.9, 22.8, 22.0, 21.1, 20.7, 19.9, 19.8 (2C), 19.7, 14.7, 13.3, 12.5, 11.9. **HRMS** (ESI +) m/z $[M+H]^+$ calcd for $C_{47}H_{74}BO_5$ 729.5624, found 729.5645.

5 Determination of stereochemistry

The determination of the configuration of the alkenes was determined performing quantitative nOe experiments. The corresponding correlation between vicinal proton is given in each case:

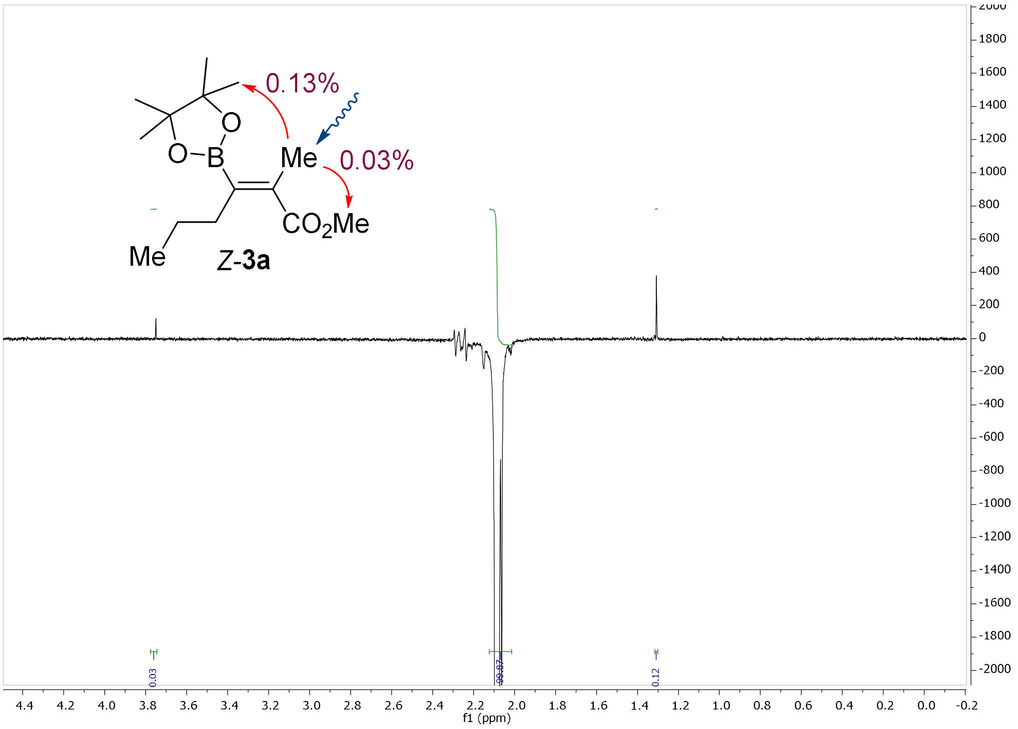

Figure S2. Observed nOe effect for *Z*-**3a.**

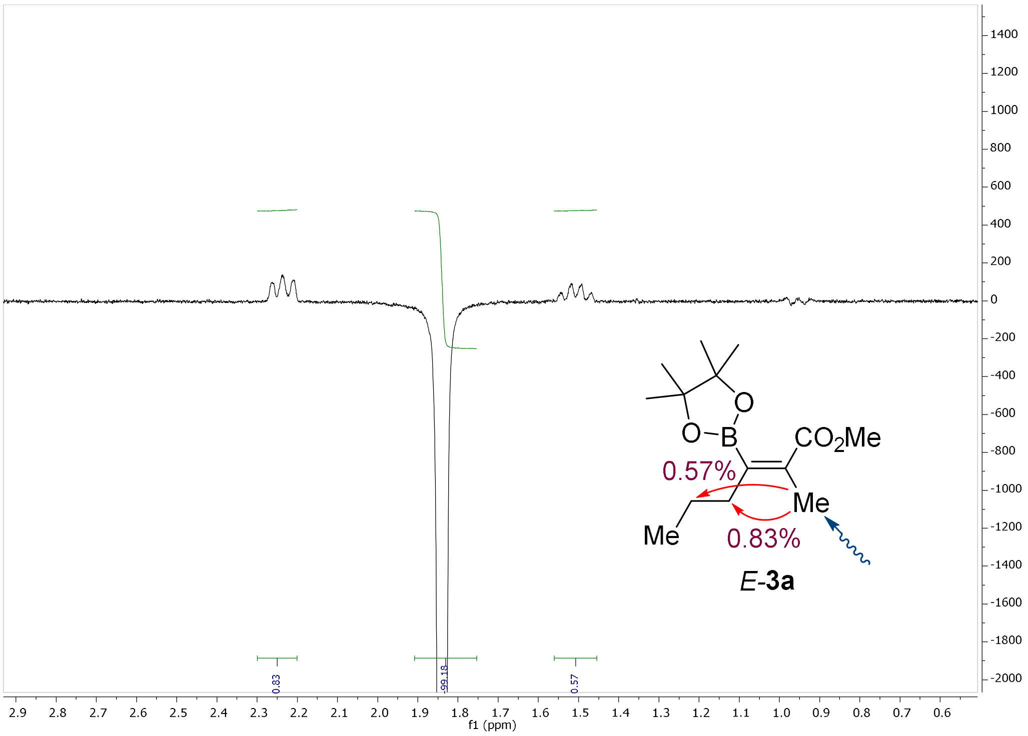

Figure S3. Observed nOe effect for *E*-**3a.**

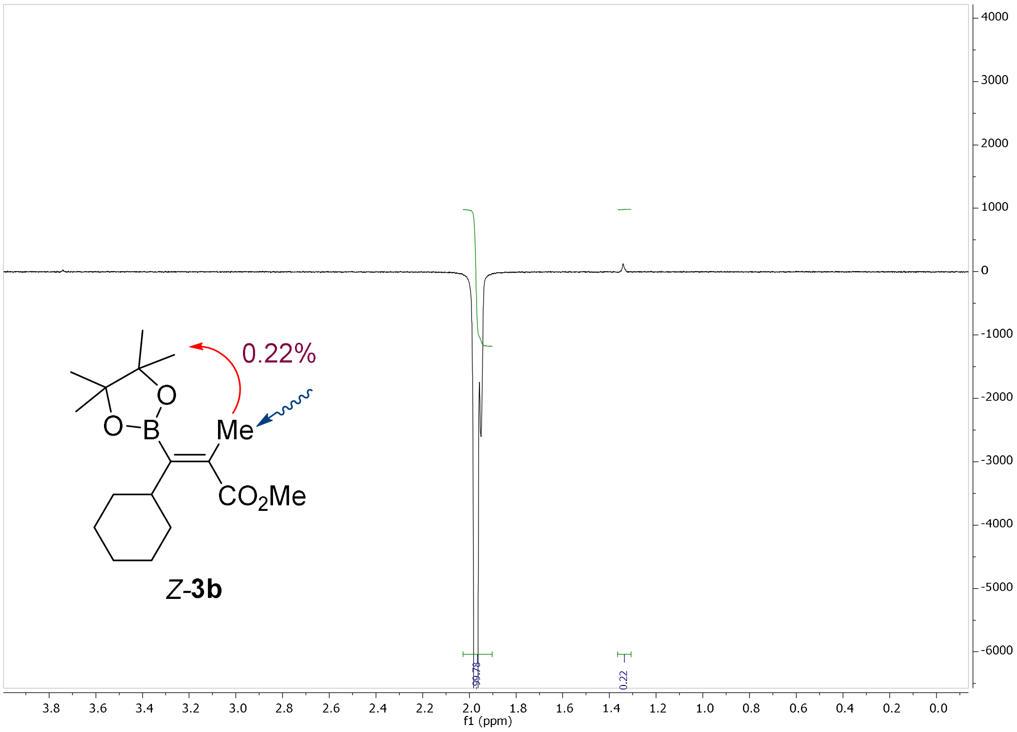

Figure S4. Observed nOe effect for *Z*-**3b.**

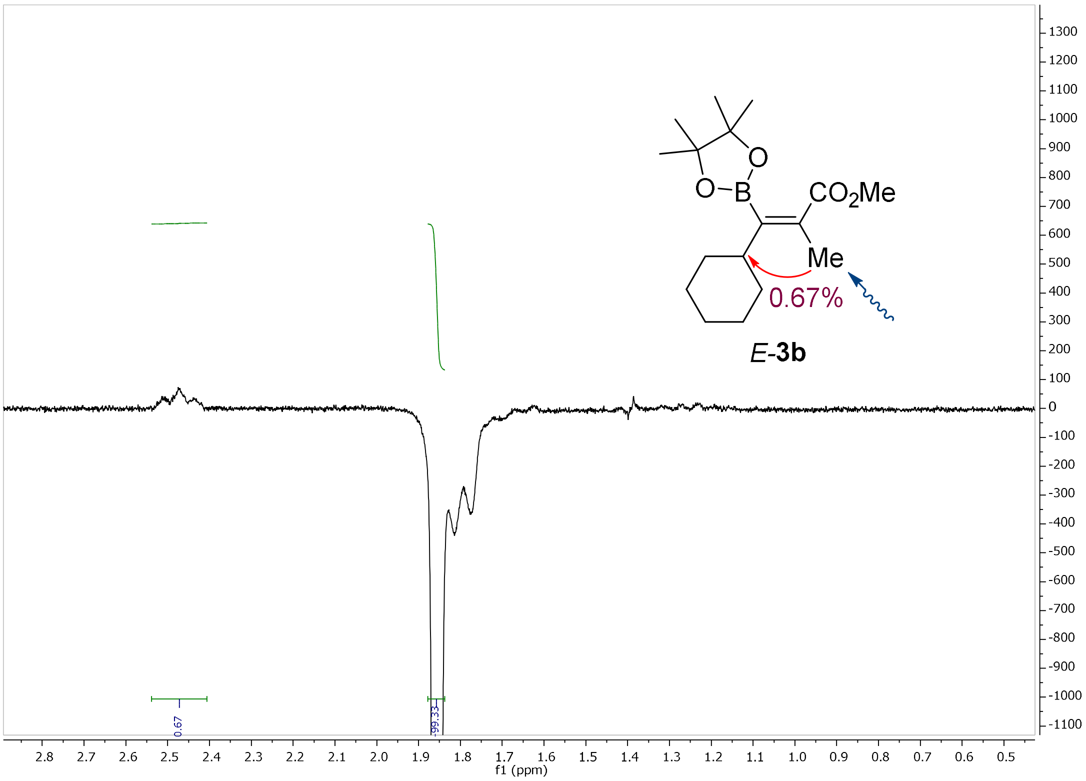

Figure S5. Observed nOe effect for *E*-**3b.**

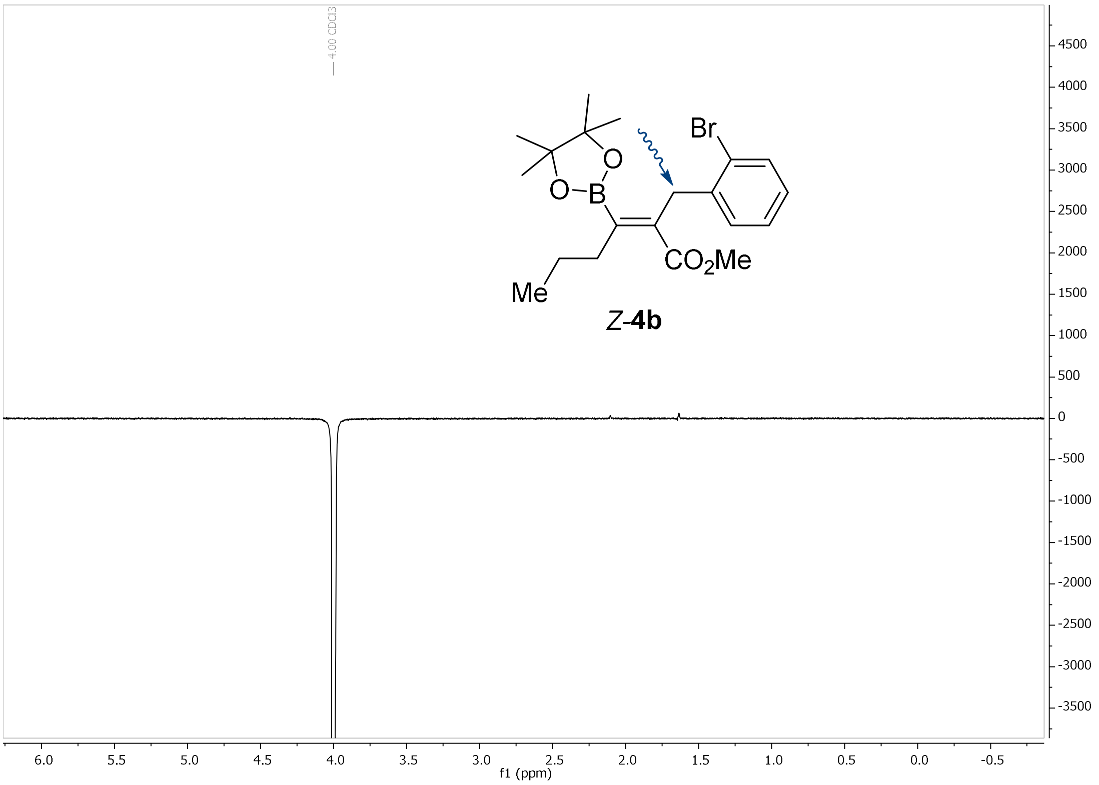

Figure S6. Observed nOe effect for *Z*-**4b.**

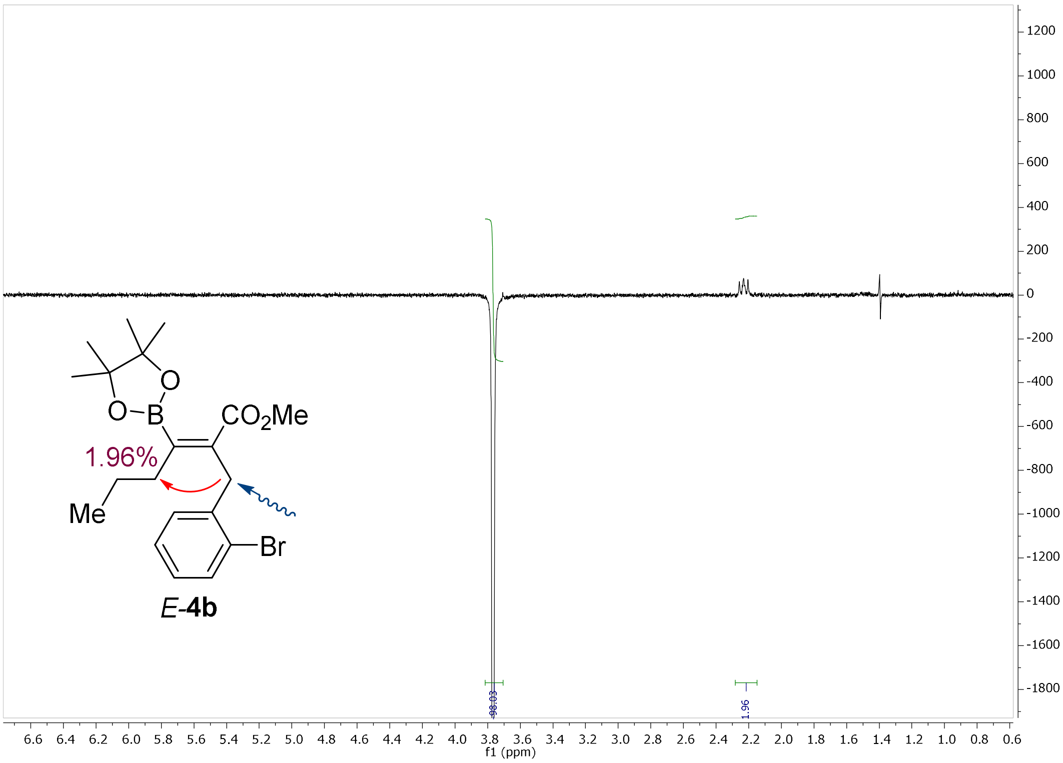

Figure S7. Observed nOe effect for *E*-**4b.**

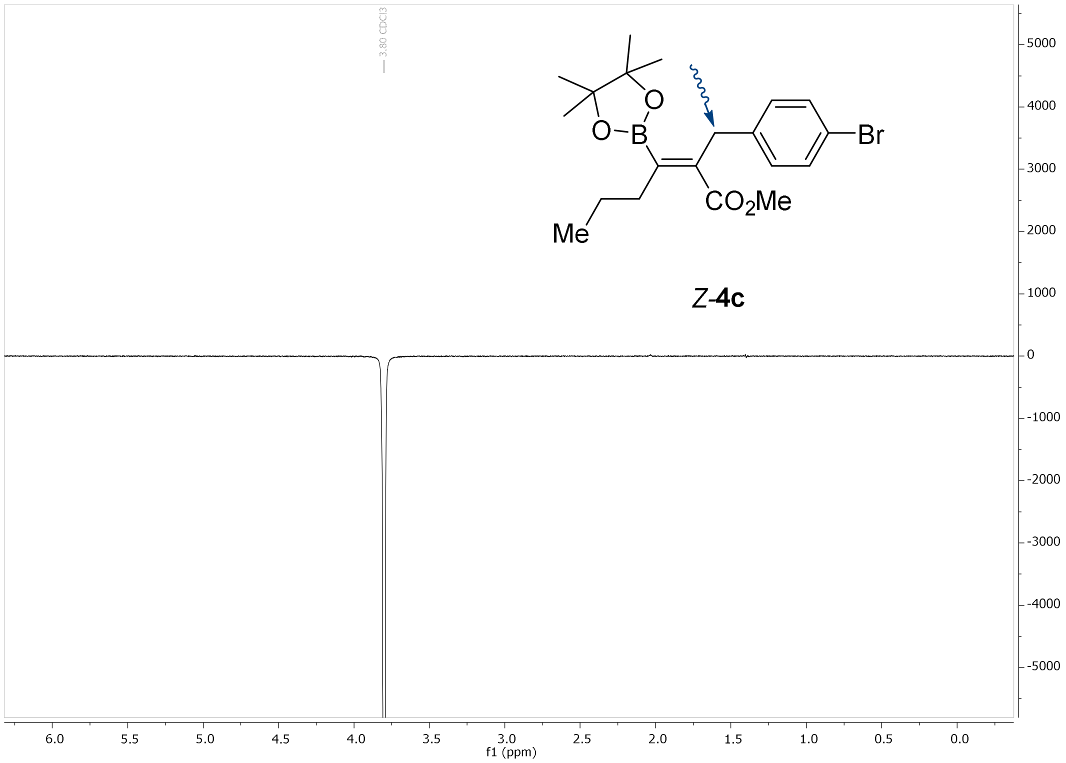

Figure S8. Observed nOe effect for *E*-**4c.**

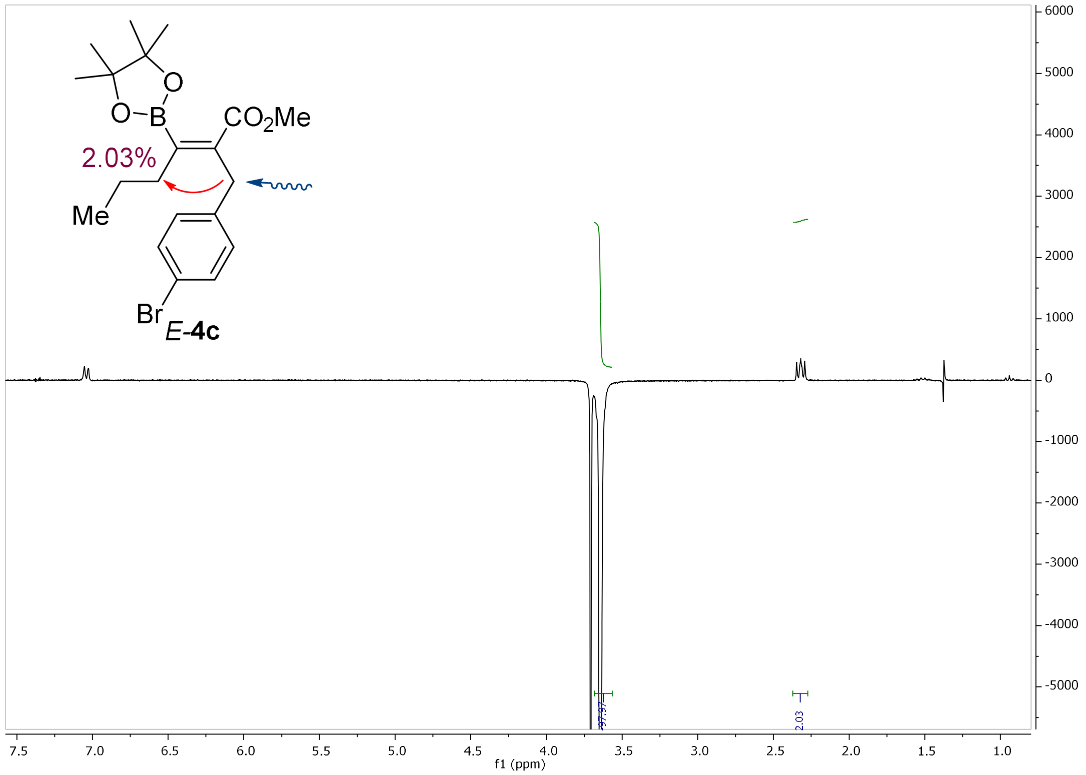

Figure S9. Observed nOe effect for *E*-**4c.**

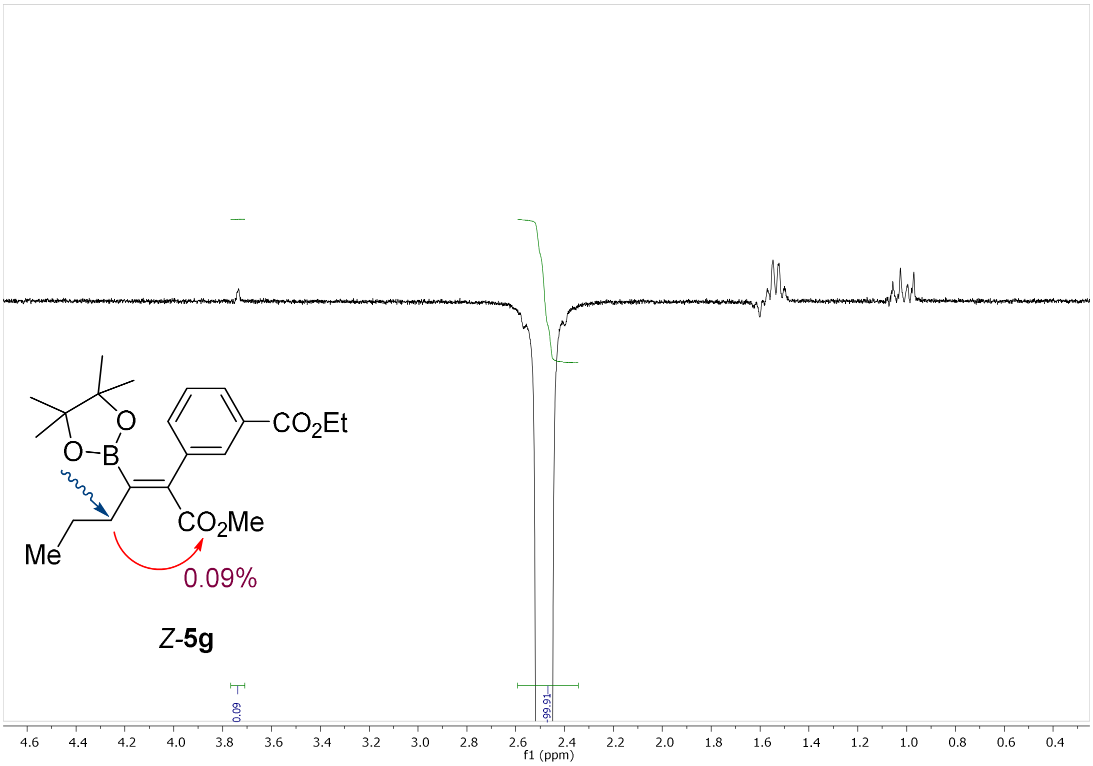

Figure S10. Observed nOe effect for *Z*-**5g.**

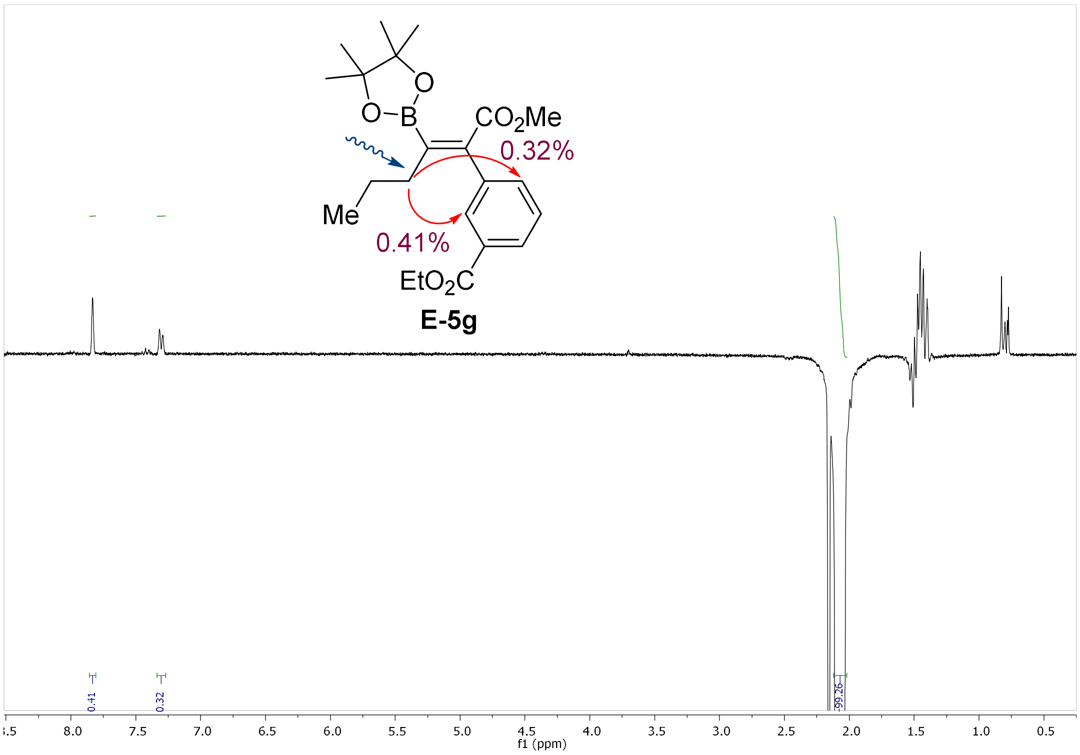

Figure S11. Observed nOe effect for *E*-**5g.**

6 Computational Studies

Computational details

All calculations corresponding to the modelling of the carboboration reaction have been carried out within the framework of density functional theory, using the *Gaussian 16* package.[10] We employed the meta-GGA M06-L[11] exchange-correlation functional for gas-phase geometry optimizations and frequency calculations, in combination with the cc-pVDZ[12] basis set for H, B, C, O, and P atoms, and the def2-SVP[13] basis set and effective core potential for Cu and Pd atoms. More accurate electronic energies were obtained through single-point energy calculations at the M06-L/cc-pVTZ(H,B,C,O,P),def2-TZVP(Cu,Pd) level of theory, including solvent effects (toluene) using the SMD[14] model.

The condensed Fukui functions[15] can be used to estimate the relative electrophilicity or nucleophilicity of a given atomic position in a molecule. In contrast to the "global" Fukui functions, which are obtained from the difference between the unperturbed electron density (no additional electrons) and the electron density upon addition/removal of one electron, condensed Fukui functions reflect the variation in the atomic charges (populations) under the same circumstances. Therefore, to evaluate the nucleophilicity of the C_α position of alkenyl-Cu^I species, a natural bond orbital (NBO) analysis allowed us to calculate its population variation upon removal of an electron, $f_- = P(N) - P(N\text{-}1) = q(N\text{-}1) - q(N)$. Larger values of f_- are related to higher nucleophilicities.

The photoisomerization reaction was investigated employing a multiconfigurational approach using *OpenMolcas 19.11*.[16] Gas-phase geometry optimizations and frequency calculations were performed at the SA-CASSCF[17]/cc-pVDZ (state-average complete active space self-consistent field) level of theory, averaged over 3 singlet or 2 triplet states. A (6,5) active space, 6 electrons in 5 orbitals, was used, including the n_{CO}, π_{CC}, π_{CO}, π_{CC}*, and π_{CO}* orbitals shown in Figure **S12** below. Final electronic energies were computed at the MS-CASPT2[18]/cc-pVTZ (multistate complete active space with second-order perturbation theory) level.

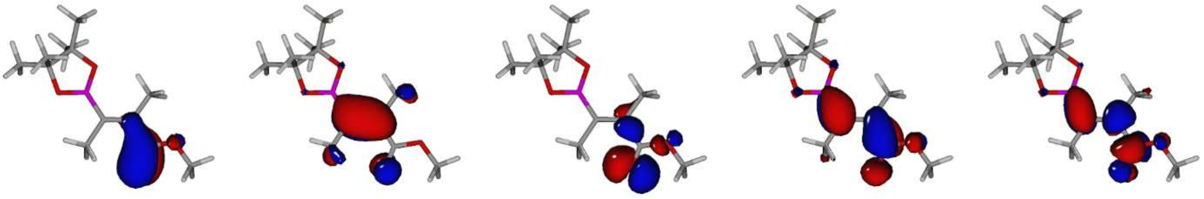

Figure S12. CASSCF orbitals included in the (6,5) active space of *Z*,*s-cis*-3a'.

Stability of alkenyl boronates.

As explained in the main text, the stereoselectivity of the Ir-catalyzed photoisomerization can be understood in terms of the relative stabilities of the *E* and *Z* isomers of alkenyl boronates. In all cases, the *E*,*s-cis* isomer is the most stable (see Table **S6**), *ca.* 4 kcal mol^{-1} lower in energy than the *Z* isomers, suggesting that photoisomerization is a thermodynamically-driven process. This stability comes from the onset of a $n_O \rightarrow p_B$ interaction in the *E* isomers, shown in Figure **S13** below, which is absent in the *Z* ones.

Table S6. Relative Gibbs free energies (in kcal mol^{-1}) of the different isomers of alkenyl boronates 3a', 4a', and 5a'

Molecule	*E*, *s-cis*	*E*, *s-trans*	*Z*, *s-cis*	*Z*, *s-trans*
3a'	0.0	0.9	4.5	5.2
4a'	0.0	1.5	4.4	5.0
5a'	0.0	0.7	2.8	4.0

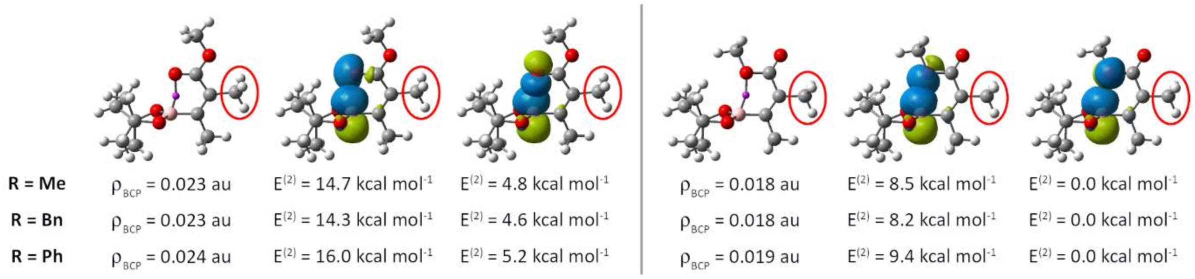

Figure S13. Electron density at the B-O bond critical point, and second-order interaction energies between representative NBO orbital pairs for *s-cis* (left) and *s-trans* (right) alkenyl boronates. The *E* isomers of 3a' are shown as an example, with the methyl to be replaced by a benzyl (in 4a') or phenyl (in 5a') group highlighted in a red circle.

Comparison of the photoisomerization of 3a’ and 5a’

We presented in the main text the potential energy profile for the photoisomerization of *Z,s-cis*-3a’, bearing a methyl group at the C_α position, into *E,s-cis*-3a’ through the T_1 state. This substrate can also be used as a model for the benzyl boronate *Z,s-cis*-4a’, as the methylene in the benzyl group blocks the conjugation between the double bond and the aromatic ring. However, it is feasible that the photoisomerization of phenyl boronates evolves *via* different pathways, due to the extension of the π system. To explore this possibility, we have calculated the photoisomerization of *Z,s-cis*-5a’, and compared it with that of *Z,s-cis*-3a’. The results, shown in Figure **S14** below, show that the replacement of the methyl group in 3a' by a phenyl group in 5a' does not affect the electronic character of the T1 state (> 88 kcal·mol^{-1}). Moreover, the vertical triplet energies are still much higher than that of the photocatalyst PC-7. This means that phenyl substitution does not allow vertical triplet sensitization, and that both 3a' and 5a' are photoexcited through a same alternative pathway through the hot-band model explained in the main text.

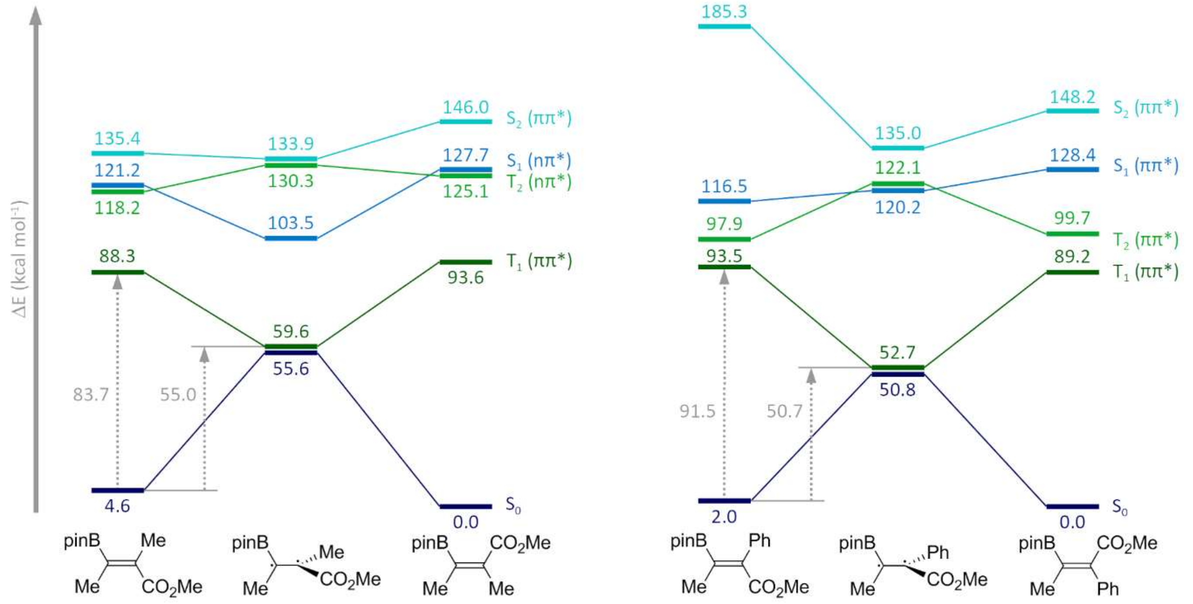

Figure S14. Potential energy profiles for the photoisomerization of *Z,s-cis*-3a’ (left) and *Z,s-cis*-5a’ (right) to their corresponding *E* isomers. Vertical, dashed arrows show the vertical and adiabatic triplet energies of the *Z* isomers.

To rule out the possibility of solvatochromism, we calculated the S_0-T_1 energies including implicit solvation. As there is no reliable way of including solvent effects on multiconfigurational calculations, we resorted to single-point DFT calculations on top of the CASSCF geometries. While DFT is less accurate than MS-CASPT2 in predicting excited-state energies, the comparison of the DFT energies in the gas-phase and in solvation will allow us to clarify the effect of the environment on the singlet-triplet gap. The results (see Table S7 below) show that the solvent has no substantial effect on the S_0-T_1 energies of both isomers, thus suggesting that inclusion of solvent effects on the MS-CASPT2 results would not have had any impact as well.

Table S7 Vertical S_0-T_1 energies (in kcal mol^{-1}) of the *s-cis* isomers of **3a’** in the gas-phase and in solvation at different levels of theory, calculated over the CASSCF/cc-pVDZ optimized geometries.

Level of theory	*Z,s-cis*-**3a’**		*E,s-cis*-**3a’**	
	Gas-phase	SMD (THF)	Gas-phase	SMD (THF)
B3LYP/cc-pVTZ	73.6	73.7	83.0	82.9

CAM-B3LYP/cc-pVTZ	73.4	73.6	83.0	82.9
LC-ωPBE/cc-pVTZ	70.3	70.6	79.9	79.9
ωB97X-D/cc-pVTZ	74.2	74.4	83.7	83.6
M06-L/cc-pVTZ	70.9	71.1	80.1	80.0
M06-2X/cc-pVTZ	76.5	76.7	86.4	86.3
M06-HF/cc-pVTZ	80.4	80.8	90.9	90.9
MS-CASPT2/cc-pVTZ	83.7	---	93.6	---

As discussed in the main text, a plausible mechanism for photosensitization is the non-classical energy transfer. At room temperature, the population of excited rovibrational states can modulate the singlet-triplet energy difference, thus possibly enabling the transition at some points of the potential energy surface around the ground-state minima. To account for this effect, we generated a sampling of 100 structures for *Z,s-cis*-**3a'** and *E,s-cis*-**3a'** assuming a Wigner distribution at 298.15 K using the CASSCF/cc-pVDZ harmonic frequencies, and carried out MS-CASPT2/cc-pVTZ single-point calculations on top of each of them. The resulting S_0-T_1 energies were grouped in the histogram shown in Figure S15 below. First, the average energies, indicated as dashed lines, are in good agreement with the values of 83.7 and 93.6 kcal mol^{-1} obtained for each isomer respectively. These results also corroborate that the S_0-T_1 gap is larger on the *E* isomer than on the *Z*. Moreover, some structures show an energy gap close to the triplet energy of **PC-7** (62 kcal mol^{-1}), being more evident for the *Z* isomer than for the *E*. Thus, a non-classical photosensitization mechanism driven by thermal fluctuations is expected to be taking place.

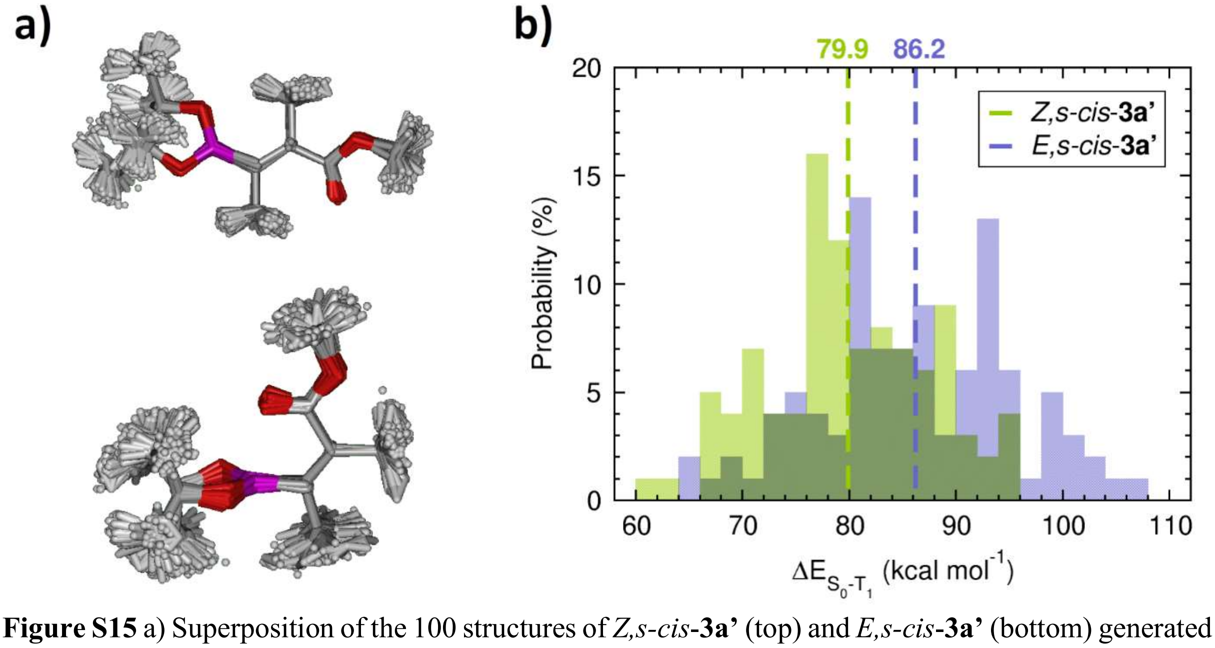

Figure S15 a) Superposition of the 100 structures of *Z,s-cis*-**3a'** (top) and *E,s-cis*-**3a'** (bottom) generated from a Wigner distribution. b) Probability distribution of the singlet-triplet energy gap of *Z,s-cis*-**3a'** (green) and *E,s-cis*-**3a'** (purple). Vertical dashed lines indicate the average values of the two data sets.

Geometries (in Å) and absolute energies

Table S8. Electronic energy (E), thermal correction to enthalpy (H_{corr}), and vibrational component of the entropy (S_v) for all the reagents, intermediates, transition states, and products of the carboboration reaction.

Molecule	E, au	H_{corr}, au	S_v, cal mol^{-1} K^{-1}
PhI	-529.566248	0.096757	10.240
Toluene	-271.616646	0.134878	12.583
I01-Cu	-4581.618496	0.877297	221.032
I01-Pd	-2584.747703	0.710611	177.884
I02	-5111.179996	0.976043	260.067
I03	-5111.187281	0.976812	258.542
I04	-5111.260288	0.977880	261.663
I05	-4395.170281	0.714238	183.552
I06	-756.637129	0.313884	62.061
I07	-2842.702770	0.672411	177.418
I08	-2842.727770	0.672357	165.500
I09	-7424.367026	1.552681	410.527
I10	-3300.834932	0.973385	239.523
I11	-3300.871386	0.973984	235.913
TS1	-5111.160958	0.974611	257.555
TS2	-5111.187101	0.975700	255.379
TS3	-2842.694912	0.671176	173.655
TS4	-3300.820154	0.971584	237.933

Table S9. Electronic energy (E), thermal correction to enthalpy (H_{corr}), and vibrational component of the entropy (S_v) for all the reagents and products of the photoisomerization of alkenyl boronates.

Molecule	E, au	H_{corr}, au	S_v, cal mol^{-1} K^{-1}
3a.E_s-cis	-795.962344	0.342762	65.931
3a.E_s-trans	-795.959367	0.342795	69.117
3a.Z_s-cis	-795.955490	0.343363	66.637
3a.Z_s-trans	-795.953827	0.343431	67.916
4a.E_s-cis	-1027.054664	0.428641	87.751
4a.E_s-trans	-1027.051659	0.428987	89.740
4a.Z_s-cis	-1027.048315	0.429214	87.676
4a.Z_s-trans	-1027.047787	0.429455	87.037
5a.E_s-cis	-987.732952	0.398820	79.862
5a.E_s-trans	-987.729953	0.398897	83.926
5a.Z_s-cis	-987.728144	0.398852	80.643
5a.Z_s-trans	-987.726231	0.398972	80.884

Table S10. Electronic energies for the ground and lowest-lying excited singlet and triplet states of the reagents, intermediates, and products of the photoisomerization of alkenyl boronates.

Molecule	E (S_0), au	E (S_1), au	E (S_2), au	E (T_1), au	E (T_2), au
3a.E_s-cis	-794.221958	-794.018470	-794.989265	-794.072825	-794.022524
3a.T1min	-794.133289	-794.057027	-794.008613	-794.126993	-794.014232
3a.Z_s-cis	-794.214693	-794.028822	-794.006113	-794.081192	-794.033585
5a.E_s-cis	-985.539588	-985.334987	-985.303356	-985.397440	-985.380635
5a.T1min	-985.458705	-985.347964	-985.324373	-985.455649	-985.345033
5a.Z_s-cis	-985.536411	-985.353945	-985.244309	-985.390573	-985.383500

Carboboration reaction

12
PhI.xyz

C	-0.561689	-0.000050	-0.000002
C	-1.251250	1.214040	-0.000008
C	-2.645748	1.205421	0.000012
C	-3.346141	0.000011	-0.000001
C	-2.645836	-1.205372	-0.000010
C	-1.251272	-1.214050	0.000007
H	-0.707205	2.159005	-0.000013
H	-3.184947	2.154526	0.000006
H	-4.437175	0.000107	-0.000000
H	-3.184967	-2.154516	-0.000002
H	-0.707378	-2.159101	0.000018
I	1.555345	-0.000000	0.000000

15
Toluene.xyz

C	-2.414218	0.000074	0.008209
H	-2.829042	-0.885018	-0.492721
H	-2.829012	0.891974	-0.480429
H	-2.801976	-0.007249	1.039381
C	-0.915915	0.000159	-0.010896
C	-0.195365	-1.201012	-0.008678
C	-0.195173	1.201124	-0.008670
C	1.198230	-1.204020	0.001993
H	-0.739242	-2.149652	-0.018466
C	1.198493	1.203877	0.001988
H	-0.738846	2.149875	-0.018455
C	1.901821	-0.000112	0.008564

H	1.738497	-2.153156	0.001001
H	1.738893	2.152935	0.000992
H	2.993486	-0.000244	0.013647

103
I01-Cu.xyz

C	1.666667	-2.255925	-1.944690
C	0.953210	-1.109780	-2.144635
B	1.690565	-2.766129	-0.498970
C	0.834283	-0.476269	-3.447666
O	-0.044913	-0.658246	-4.281274
O	1.795199	0.489658	-3.622739
C	1.622774	1.297347	-4.781846
H	2.413923	2.054157	-4.744035
H	1.710163	0.704204	-5.703375
H	0.637794	1.786909	-4.780785
C	2.430792	-2.985023	-3.018082
H	2.301614	-2.530179	-4.011269
H	3.512243	-3.010854	-2.799098
O	2.212840	-3.958031	-0.042375
O	1.157917	-2.004036	0.547709
C	2.222081	-3.898476	1.407796
C	1.100467	-2.858093	1.720407
C	-0.293719	-3.464329	1.755714
H	-0.495715	-4.067286	0.858412
H	-0.446039	-4.095666	2.643127
H	-1.038181	-2.654323	1.785814
C	1.352302	-2.014628	2.953105
H	1.429733	-2.649014	3.849168
H	2.276026	-1.428906	2.861829
H	0.520319	-1.314316	3.106426
C	1.959554	-5.286159	1.952861
H	1.871362	-5.265623	3.049450
H	1.040678	-5.718396	1.538878
H	2.790166	-5.957021	1.695922
C	3.602691	-3.404217	1.814230
H	3.787306	-2.388617	1.435563
H	3.731896	-3.395692	2.906241
H	4.365546	-4.067497	1.385233
H	2.122654	-4.040931	-3.091481
Cu	0.071637	-0.243683	-0.577842
P	-2.160504	-0.402835	-0.253508
P	1.299418	1.409249	0.396989
C	3.039549	0.888634	0.628048
C	3.692214	0.851537	1.866397
C	3.701349	0.398128	-0.511690
C	4.989621	0.345137	1.964078
H	3.186695	1.215862	2.764409
C	4.997459	-0.098825	-0.407884
H	3.191473	0.401414	-1.480005
C	5.645994	-0.127528	0.828920
H	5.488461	0.322618	2.935614
H	5.501641	-0.474227	-1.300999

H	6.660497	-0.523787	0.907590
C	1.410639	2.847625	-0.741513
C	0.266236	3.143495	-1.496343
C	2.565701	3.619027	-0.918606
C	0.267985	4.210433	-2.391532
H	-0.627437	2.518792	-1.389017
C	2.568852	4.678842	-1.824941
H	3.473166	3.380033	-0.356919
C	1.420422	4.979329	-2.557827
H	-0.630352	4.428669	-2.972956
H	3.475681	5.272053	-1.960957
H	1.427409	5.808829	-3.268025
C	-2.907491	0.788519	-1.449559
C	-3.646236	1.912970	-1.055515
C	-2.536636	0.659786	-2.798089
C	-4.029259	2.870254	-1.996562
H	-3.918485	2.059528	-0.008443
C	-2.929228	1.613644	-3.732834
H	-1.904372	-0.166449	-3.129781
C	-3.679160	2.722500	-3.337379
H	-4.604796	3.740031	-1.672098
H	-2.626958	1.487386	-4.774835
H	-3.980173	3.474820	-4.069603
C	-2.773345	-2.079836	-0.704554
C	-2.243326	-2.656995	-1.872029
C	-3.604820	-2.855336	0.115064
C	-2.580640	-3.960363	-2.227513
H	-1.532818	-2.102865	-2.492726
C	-3.924089	-4.166408	-0.238223
H	-3.992122	-2.449729	1.051582
C	-3.421488	-4.720216	-1.413962
H	-2.162333	-4.388982	-3.140273
H	-4.568964	-4.757366	0.415823
H	-3.672468	-5.746697	-1.688823
C	-3.017125	0.029040	1.318061
C	-4.412093	0.006383	1.476827
C	-2.262468	0.559974	2.372322
C	-5.012884	0.502772	2.630586
H	-5.033029	-0.374304	0.662087
C	-2.840678	1.087126	3.532735
C	-4.228767	1.052800	3.646525
H	-6.099161	0.481733	2.731050
H	-4.700930	1.464520	4.542148
C	0.842070	2.198510	2.004205
C	1.481382	3.337928	2.513499
C	-0.251932	1.702649	2.724521
C	1.036355	3.950029	3.682088
H	2.331724	3.757793	1.969542
C	-0.724779	2.299420	3.898243
C	-0.067249	3.435676	4.365488
H	1.543892	4.839527	4.058589
H	-0.424631	3.922239	5.276572
O	-0.891068	0.579737	2.236938

C	-1.913533	1.664583	4.566640
H	-2.438425	2.384837	5.209848
H	-1.566711	0.857226	5.239254

84
I01-Pd.xyz

C	-3.617611	-0.257807	-0.332889
C	-1.807260	0.553361	1.741529
C	-1.949790	2.035303	-0.705638
C	-4.496175	-0.550303	0.718106
C	-4.036140	-0.517941	-1.648462
C	-1.334408	-0.499271	2.537403
C	-2.232703	1.734837	2.361734
C	-3.089530	2.646454	-1.245370
C	-0.763229	2.785584	-0.705854
C	-5.761965	-1.078931	0.457748
H	-4.192055	-0.367211	1.751555
C	-5.305032	-1.025479	-1.908718
H	-3.346473	-0.324588	-2.477142
C	-1.310921	-0.382125	3.925132
H	-0.976403	-1.412597	2.050322
C	-2.188937	1.858161	3.750381
H	-2.605018	2.565027	1.755366
C	-3.028387	3.929286	-1.789110
H	-4.037170	2.104524	-1.249824
C	-0.674796	4.070897	-1.242669
O	0.347719	2.201347	-0.132055
C	-6.173643	-1.313134	-0.852825
H	-6.433180	-1.303276	1.289868
H	-5.615395	-1.209860	-2.939893
C	-1.735147	0.798407	4.535071
H	-0.940837	-1.211212	4.532057
H	-2.518597	2.786827	4.221771
C	-1.825835	4.632919	-1.797150
H	-3.928449	4.381876	-2.208826
C	0.651519	4.773260	-1.172902
C	1.567253	2.510885	-0.700567
H	-7.165739	-1.722594	-1.053483
H	-1.704197	0.895765	5.622613
H	-1.774986	5.636353	-2.227298
C	1.779086	3.782040	-1.238355
H	0.741571	5.521934	-1.973522
H	0.716318	5.342701	-0.226060
C	2.549262	1.506796	-0.694942
C	3.027013	4.061263	-1.796496
P	2.092495	-0.197448	-0.140242
C	3.794683	1.834882	-1.250709
C	4.031452	3.095862	-1.795281
H	3.208891	5.048540	-2.228534
C	2.128655	-0.082906	1.700684
C	3.673908	-1.103783	-0.465715
H	4.587053	1.084789	-1.275196
H	5.007854	3.323083	-2.226527

C	2.197310	-1.285767	2.423800
C	2.022347	1.119995	2.408229
C	3.751301	-1.866570	-1.637935
C	4.786101	-1.065801	0.387883
C	2.188441	-1.282299	3.815112
H	2.276798	-2.234106	1.882489
C	1.994646	1.119780	3.803180
H	1.965151	2.068725	1.871121
C	4.913006	-2.566240	-1.961431
H	2.880387	-1.905111	-2.300318
C	5.943518	-1.775613	0.072743
H	4.746101	-0.473178	1.305934
C	2.084826	-0.076247	4.511619
H	2.259754	-2.226797	4.359718
H	1.905038	2.067451	4.338954
C	6.010916	-2.525219	-1.102796
H	4.957506	-3.152507	-2.881720
H	6.801679	-1.739636	0.747586
H	2.068625	-0.071351	5.603622
H	6.918915	-3.080573	-1.346636
P	-1.874664	0.296852	-0.084649
Pd	-0.001421	-0.963647	-0.680892
C	-0.787290	-3.257581	-1.964751
C	-1.951335	-3.803417	0.545480
C	-0.181047	-3.025693	-3.316571
C	0.023982	-3.494025	-0.835226
C	-2.181889	-3.325258	-1.807919
C	-0.564427	-3.753039	0.412841
C	-2.762019	-3.604377	-0.574458
H	-2.400406	-3.998772	1.521951
H	0.841290	-2.631256	-3.240445
H	-0.770603	-2.310087	-3.906309
H	-0.130227	-3.958645	-3.901049
H	1.110291	-3.545913	-0.948891
H	-2.820300	-3.148647	-2.678316
H	0.074509	-3.926493	1.282446
H	-3.849762	-3.634150	-0.480164

115
I02.xyz

C	4.215538	-2.245701	-1.429129
C	2.974287	-2.270199	-0.856372
B	4.799999	-0.839924	-1.630626
C	2.255354	-3.527829	-0.645168
O	1.124889	-3.795961	-1.034824
O	2.949992	-4.424301	0.125541
C	2.221761	-5.601638	0.459232
H	2.871871	-6.184150	1.121387
H	1.973833	-6.187953	-0.436437
H	1.280687	-5.355957	0.973076
C	5.000627	-3.442318	-1.897407
H	4.386187	-4.345939	-2.004179
H	5.805039	-3.687612	-1.181680

O	5.888572	-0.513895	-2.408362
O	4.219488	0.303404	-1.053105
C	6.153902	0.900235	-2.225579
C	4.769199	1.451091	-1.764130
C	3.824356	1.737717	-2.920617
H	3.793881	0.897918	-3.628995
H	4.106859	2.646797	-3.470148
H	2.804453	1.876262	-2.531429
C	4.857919	2.633325	-0.824355
H	5.394784	3.465293	-1.304354
H	5.376780	2.380037	0.108130
H	3.857459	2.998116	-0.554751
C	6.649678	1.476713	-3.534821
H	6.772950	2.567336	-3.459894
H	5.961153	1.261359	-4.360357
H	7.626186	1.046071	-3.792607
C	7.221098	1.011152	-1.146991
H	6.859518	0.610768	-0.189204
H	7.542565	2.050352	-0.990011
H	8.099442	0.424043	-1.445281
H	5.501678	-3.247092	-2.857848
Cu	2.128948	-0.518066	-0.526423
I	2.644715	1.903311	2.562427
C	3.359117	-0.087889	2.406808
C	4.685062	-0.310407	2.036860
C	2.498130	-1.148382	2.695294
C	5.156315	-1.620513	1.963432
H	5.342820	0.528245	1.805201
C	2.990293	-2.451168	2.617222
H	1.461062	-0.965718	2.983378
C	4.314530	-2.690334	2.257126
H	6.191396	-1.800711	1.662347
H	2.317294	-3.285346	2.828589
H	4.680597	-3.714043	2.173373
P	0.149855	0.447015	-0.416934
C	-0.806080	-0.058913	-1.911252
C	-0.602934	-0.377114	1.045242
C	-0.222748	2.233352	-0.253931
C	-1.628958	0.808871	-2.640576
C	-0.635648	-1.377137	-2.360510
C	-0.954999	0.333535	2.197729
C	-0.606752	-1.781074	1.076661
C	0.832793	3.130116	-0.443774
C	-1.509933	2.761933	-0.072622
C	-2.308965	0.353657	-3.768694
H	-1.743266	1.853156	-2.345358
C	-1.329891	-1.830167	-3.480334
H	0.056651	-2.056615	-1.852410
C	-1.310351	-0.346340	3.363740
H	-0.937138	1.425651	2.197596
C	-0.976010	-2.452715	2.238567
H	-0.293440	-2.356798	0.201721
C	0.618706	4.505464	-0.474820

H	1.833379	2.712152	-0.575625
C	-1.755339	4.138557	-0.114033
O	-2.535856	1.864345	0.121199
C	-2.171293	-0.970164	-4.185547
H	-2.951369	1.042258	-4.322514
H	-1.191864	-2.862438	-3.808656
C	-1.319192	-1.739055	3.388798
H	-1.584770	0.220401	4.256014
H	-0.982051	-3.545572	2.246673
C	-0.674762	4.998593	-0.321567
H	1.454897	5.189120	-0.630313
C	-3.165767	4.621500	0.053355
C	-3.796053	2.236291	-0.319167
H	-2.706834	-1.325095	-5.069434
H	-1.601724	-2.267949	4.301449
H	-0.860930	6.074982	-0.362399
C	-4.144502	3.585322	-0.418569
H	-3.357880	4.847919	1.119593
H	-3.315889	5.573153	-0.477687
C	-4.687390	1.193944	-0.617114
C	-5.411505	3.901856	-0.912303
P	-4.121646	-0.554228	-0.364722
C	-5.951092	1.555979	-1.099673
C	-6.306077	2.894748	-1.262433
H	-5.694854	4.952822	-1.010692
C	-5.557791	-1.466809	-1.089903
C	-4.468510	-0.763543	1.438960
H	-6.669166	0.774901	-1.356116
H	-7.293929	3.150570	-1.649150
C	-5.428036	-1.895883	-2.418478
C	-6.751838	-1.742252	-0.406845
C	-4.436134	-2.071972	1.947758
C	-4.716467	0.290601	2.326813
C	-6.469143	-2.570084	-3.055043
H	-4.494672	-1.698089	-2.953568
C	-7.787917	-2.426646	-1.040288
H	-6.873285	-1.415286	0.629246
C	-4.677429	-2.321017	3.295520
H	-4.228493	-2.907526	1.272446
C	-4.936460	0.042154	3.682111
H	-4.753392	1.319027	1.959798
C	-7.650975	-2.839681	-2.366130
H	-6.351561	-2.896260	-4.090701
H	-8.711466	-2.634820	-0.495377
C	-4.924895	-1.262745	4.170977
H	-4.660959	-3.348429	3.666767
H	-5.131627	0.878062	4.357927
H	-8.463950	-3.376321	-2.859636
H	-5.109076	-1.455667	5.229932

115

I03.xyz

C	-2.818205	1.005713	2.270205

C	-2.697494	-0.275627	1.868212
B	-3.140309	2.105423	1.232882
C	-2.429345	-1.455311	2.706702
O	-1.620935	-2.323568	2.408820
O	-3.203859	-1.528518	3.810074
C	-3.025469	-2.721414	4.579446
H	-3.724849	-2.643747	5.416458
H	-1.994211	-2.804451	4.946818
H	-3.250428	-3.609647	3.974096
C	-2.651725	1.454310	3.697375
H	-2.275427	0.667856	4.361694
H	-3.615850	1.803331	4.100481
O	-3.587670	3.352013	1.605293
O	-3.036870	1.950422	-0.138097
C	-4.082358	3.995670	0.399613
C	-3.273664	3.272843	-0.723039
C	-1.912730	3.908031	-0.958160
H	-1.368094	4.045283	-0.013153
H	-2.008767	4.887535	-1.447805
H	-1.308558	3.263016	-1.610101
C	-4.027930	3.104618	-2.024138
H	-4.329914	4.084743	-2.422434
H	-4.929264	2.491318	-1.902280
H	-3.390912	2.624249	-2.777722
C	-3.826904	5.484381	0.503474
H	-4.105492	5.993863	-0.430714
H	-2.775394	5.705983	0.719276
H	-4.432571	5.914251	1.312003
C	-5.576015	3.713504	0.334571
H	-5.779616	2.636109	0.259698
H	-6.049666	4.217107	-0.519636
H	-6.054529	4.077832	1.252884
H	-1.970579	2.317437	3.753226
Cu	-2.578771	-0.927461	0.023382
I	-2.778473	-2.366288	-2.121506
C	-4.444646	-1.267965	0.507447
C	-5.413676	-0.357866	0.088693
C	-4.816232	-2.458533	1.129537
C	-6.765372	-0.655365	0.274056
H	-5.118836	0.570845	-0.404309
C	-6.170331	-2.747509	1.312336
H	-4.058256	-3.180320	1.446490
C	-7.147554	-1.847068	0.889646
H	-7.523606	0.053289	-0.070256
H	-6.459403	-3.689443	1.785201
H	-8.204989	-2.074825	1.037066
P	-0.312117	-0.344268	-0.343611
C	0.410707	0.788823	0.907783
C	0.752668	-1.832426	-0.464500
C	-0.065976	0.521990	-1.936658
C	0.830953	2.084252	0.582362
C	0.445663	0.391123	2.253003
C	1.015204	-2.368408	-1.733341

C	1.185637	-2.525443	0.671529
C	-1.183779	0.849761	-2.709869
C	1.204739	0.897572	-2.410557
C	1.306336	2.946695	1.569271
H	0.790942	2.435379	-0.450328
C	0.935441	1.246906	3.235280
H	0.067844	-0.591319	2.539110
C	1.720158	-3.561918	-1.859841
H	0.656694	-1.857483	-2.629182
C	1.888612	-3.720162	0.536802
H	0.968962	-2.148661	1.669865
C	-1.047547	1.541233	-3.911566
H	-2.172093	0.559992	-2.348673
C	1.359094	1.612284	-3.602656
O	2.291523	0.540617	-1.644753
C	1.366175	2.529820	2.897976
H	1.634103	3.951245	1.292225
H	0.961071	0.914084	4.275227
C	2.159683	-4.241800	-0.726024
H	1.922052	-3.963330	-2.855012
H	2.227754	-4.245826	1.431692
C	0.216948	1.927535	-4.341772
H	-1.929387	1.778191	-4.509058
C	2.736604	1.990092	-4.055600
C	3.446127	1.298107	-1.759783
H	1.738714	3.206516	3.670385
H	2.714676	-5.176715	-0.825842
H	0.335617	2.480375	-5.277469
C	3.688486	2.069837	-2.899940
H	3.106929	1.243302	-4.783799
H	2.713835	2.942045	-4.607485
C	4.362889	1.213369	-0.698218
C	4.846559	2.848237	-2.938437
P	3.999328	0.078747	0.724898
C	5.516658	2.002386	-0.783891
C	5.751408	2.827142	-1.882418
H	5.037255	3.466681	-3.819236
C	5.334712	0.596722	1.897968
C	4.707222	-1.504400	0.084490
H	6.245277	1.972553	0.027919
H	6.653913	3.439318	-1.919766
C	4.989567	1.547407	2.869124
C	6.652903	0.116843	1.868668
C	4.977620	-2.520002	1.017122
C	4.929857	-1.769789	-1.271392
C	5.935411	2.015460	3.780062
H	3.962660	1.922036	2.905380
C	7.595549	0.575795	2.787351
H	6.947240	-0.618964	1.116157
C	5.492243	-3.746788	0.609638
H	4.795281	-2.338157	2.080897
C	5.428454	-3.007124	-1.681466
H	4.725158	-1.001830	-2.020930

C	7.240852	1.527247	3.744189
H	5.648309	2.757940	4.527864
H	8.617367	0.191540	2.750787
C	5.720374	-3.995865	-0.744860
H	5.711110	-4.515790	1.354113
H	5.597539	-3.192800	-2.744577
H	7.981138	1.885185	4.462660
H	6.121172	-4.959040	-1.067383

115
I04.xyz

C	-5.804233	-0.441608	0.811859
C	-5.422223	0.719490	1.416510
B	-4.823408	-1.646226	0.651085
C	-6.320784	1.900109	1.440976
O	-7.444200	1.965220	0.983797
O	-5.734750	2.956408	2.066991
C	-6.545186	4.128723	2.107516
H	-5.950405	4.886912	2.625112
H	-6.799235	4.466788	1.094044
H	-7.481170	3.940702	2.650379
C	-7.152726	-0.645569	0.185176
H	-7.303117	0.052905	-0.650185
H	-7.256635	-1.669175	-0.192605
O	-5.295276	-2.921856	0.487218
O	-3.429798	-1.599303	0.595986
C	-4.194126	-3.743167	0.007122
C	-2.944709	-2.987606	0.546219
C	-2.569104	-3.353084	1.970756
H	-3.442673	-3.329991	2.637438
H	-2.118499	-4.354360	2.024285
H	-1.838057	-2.624572	2.350174
C	-1.747824	-3.044713	-0.374477
H	-1.474025	-4.086995	-0.597305
H	-1.943961	-2.523881	-1.322464
H	-0.875302	-2.572271	0.096369
C	-4.358216	-5.144146	0.554238
H	-3.498528	-5.771478	0.276624
H	-4.454416	-5.151097	1.646493
H	-5.259951	-5.608457	0.134616
C	-4.280675	-3.728113	-1.510746
H	-4.161808	-2.708291	-1.908034
H	-3.517767	-4.373192	-1.968032
H	-5.267379	-4.096671	-1.819906
H	-7.970583	-0.436325	0.887126
Cu	-2.263227	0.051145	-0.251680
P	-0.037157	0.315522	-0.312763
C	0.977156	0.210811	1.206956
C	0.667835	-0.875497	-1.523853
C	0.401007	1.932380	-1.063879
C	1.546009	1.353387	1.784241
C	1.066421	-1.005489	1.898286
C	0.013731	-0.941706	-2.764383

C	1.743654	-1.729234	-1.268187
C	-0.650645	2.803916	-1.367364
C	1.705600	2.292528	-1.455412
C	2.201355	1.275377	3.011271
H	1.476443	2.315883	1.270657
C	1.742232	-1.087748	3.113381
H	0.608616	-1.905937	1.478028
C	0.440487	-1.846967	-3.732177
H	-0.846917	-0.293078	-2.963493
C	2.160984	-2.641659	-2.237886
H	2.266486	-1.678582	-0.311114
C	-0.423387	4.002808	-2.037885
H	-1.667758	2.506164	-1.094705
C	1.942119	3.465186	-2.178713
O	2.730953	1.436546	-1.104061
C	2.312920	0.054174	3.675849
H	2.632830	2.178449	3.449829
H	1.816442	-2.048397	3.627574
C	1.511668	-2.702766	-3.469732
H	-0.075671	-1.888265	-4.693564
H	3.007384	-3.299497	-2.026742
C	0.867028	4.316830	-2.446627
H	-1.256093	4.669870	-2.262957
C	3.315525	3.747718	-2.702726
C	4.033574	1.900457	-1.160610
H	2.843490	-0.007501	4.628129
H	1.841365	-3.417439	-4.226813
H	1.059224	5.237014	-3.004910
C	4.370448	3.029319	-1.919319
H	3.370557	3.442031	-3.764873
H	3.513198	4.830848	-2.707461
C	5.006979	1.186043	-0.436391
C	5.701167	3.447167	-1.945672
P	4.535112	-0.259500	0.619116
C	6.334790	1.630678	-0.509877
C	6.687182	2.752046	-1.253537
H	5.958426	4.333448	-2.531582
C	5.888269	-0.179496	1.879635
C	5.084628	-1.688079	-0.408973
H	7.103646	1.085208	0.041976
H	7.726268	3.082582	-1.290454
C	5.816175	0.862796	2.819102
C	6.935398	-1.103256	1.989420
C	4.881466	-2.968810	0.133646
C	5.623738	-1.584882	-1.696777
C	6.766702	0.984908	3.828010
H	5.005779	1.594079	2.745907
C	7.880565	-0.988167	3.010410
H	7.025151	-1.918330	1.267868
C	5.247468	-4.111339	-0.573030
H	4.437044	-3.065020	1.130408
C	5.969735	-2.731394	-2.414048
H	5.777714	-0.600895	-2.145944

C	7.802102	0.053981	3.931283
H	6.696852	1.808867	4.541565
H	8.690829	-1.717565	3.077450
C	5.793073	-3.995185	-1.853639
H	5.096900	-5.097213	-0.127759
H	6.387157	-2.632657	-3.418461
H	8.544633	0.143361	4.726565
H	6.072616	-4.889340	-2.414317
I	-3.838793	0.549627	-2.237621
C	-4.093957	0.844661	2.063723
C	-3.141149	1.769171	1.600788
C	-3.753281	0.017652	3.141234
C	-1.886860	1.855102	2.208328
H	-3.404221	2.435747	0.775960
C	-2.501449	0.106523	3.747938
H	-4.491747	-0.702171	3.505736
C	-1.563642	1.027814	3.285423
H	-1.154821	2.576412	1.833188
H	-2.257566	-0.546231	4.589580
H	-0.578893	1.096398	3.752818

85
I05.xyz

P	1.999300	0.231966	0.020883
P	-1.834885	0.129977	0.430927
C	-3.420491	-0.791601	0.425854
C	-3.503551	-1.927709	-0.384891
C	-4.516415	-0.431276	1.223293
C	-4.676056	-2.681741	-0.423040
H	-2.625205	-2.228215	-0.961366
C	-5.687158	-1.184168	1.182501
H	-4.449762	0.439454	1.882451
C	-5.769188	-2.307613	0.356012
H	-4.726704	-3.572047	-1.053547
H	-6.538332	-0.899142	1.804370
H	-6.686770	-2.899192	0.331712
C	-1.523778	0.361074	2.230670
C	-1.878539	1.519054	2.932730
C	-0.927046	-0.705426	2.918160
C	-1.648831	1.605175	4.305779
H	-2.339155	2.359652	2.406472
C	-0.709977	-0.617941	4.290509
H	-0.629988	-1.606730	2.369249
C	-1.069974	0.535751	4.987197
H	-1.928463	2.512975	4.845054
H	-0.243670	-1.453973	4.815683
H	-0.890562	0.604470	6.062468
C	2.269466	0.317505	1.834492
C	2.112324	1.486592	2.586331
C	2.595782	-0.881941	2.488000
C	2.300791	1.461418	3.967920
H	1.849218	2.426090	2.095440
C	2.794249	-0.897438	3.865503

H	2.692718	-1.806906	1.911084
C	2.650021	0.274599	4.609309
H	2.174409	2.380038	4.544978
H	3.058419	-1.834237	4.360761
H	2.802850	0.260749	5.690627
C	3.542500	-0.578100	-0.551891
C	4.802448	-0.266055	-0.019277
C	3.440987	-1.562164	-1.541311
C	5.941575	-0.914593	-0.488799
H	4.887175	0.485070	0.771458
C	4.583242	-2.209971	-2.011148
H	2.451058	-1.843511	-1.911335
C	5.832911	-1.884527	-1.488169
H	6.919156	-0.667198	-0.069841
H	4.490977	-2.983134	-2.776009
H	6.727103	-2.396187	-1.850133
C	2.178691	1.966260	-0.575098
C	3.278877	2.441764	-1.302159
C	1.075739	2.823828	-0.455569
C	3.263941	3.712996	-1.874575
H	4.149779	1.798944	-1.442239
C	1.035801	4.104339	-1.011079
C	2.148984	4.537323	-1.732489
H	4.129193	4.059409	-2.441847
H	2.135825	5.530513	-2.188058
C	-2.295216	1.817472	-0.122207
C	-3.556206	2.209580	-0.584684
C	-1.254877	2.749779	-0.220677
C	-3.750068	3.479865	-1.129266
H	-4.388107	1.503480	-0.542752
C	-1.416245	4.022859	-0.764410
C	-2.686631	4.376081	-1.226344
H	-4.738928	3.768135	-1.489752
H	-2.838659	5.366472	-1.662575
O	-0.016589	2.338248	0.236795
C	-0.211938	4.920574	-0.816676
H	-0.136837	5.492075	0.127697
H	-0.315493	5.673552	-1.611092
Cu	0.033337	-0.887101	-0.447304
I	0.258348	-3.450024	-0.142663
C	-1.462741	-1.262149	-3.338116
C	-0.074452	-1.098137	-3.187563
C	-2.260055	-0.112081	-3.387008
C	0.495050	0.176337	-3.123668
H	0.560770	-1.987943	-3.155985
C	-1.692719	1.162329	-3.328533
H	-3.344777	-0.219366	-3.483469
C	-0.313485	1.313079	-3.200267
H	1.580636	0.282573	-3.039560
H	-2.335217	2.044999	-3.373230
H	0.135735	2.308770	-3.162220
C	-2.041917	-2.634424	-3.477962
H	-3.138335	-2.624964	-3.419505

H	-1.767563	-3.080003	-4.446556
H	-1.650169	-3.312079	-2.701326

35
I06.xyz

C	0.993696	0.819494	0.085416
C	1.942464	-0.141113	-0.016088
B	-0.493304	0.355481	0.036762
C	3.401081	0.051440	-0.005183
O	4.029456	1.086665	0.100013
O	4.012978	-1.158738	-0.136431
C	5.436791	-1.094345	-0.138911
H	5.785809	-2.124963	-0.250917
H	5.802283	-0.475370	-0.969454
H	5.813801	-0.663672	0.798595
C	1.255181	2.283208	0.234566
H	2.317653	2.535525	0.268383
H	0.776125	2.834163	-0.589874
O	-1.543901	1.234603	0.147791
O	-0.887744	-0.952415	-0.119926
C	-2.752071	0.483153	-0.168268
C	-2.321386	-0.987253	0.133994
C	-2.496836	-1.366617	1.596223
H	-2.058549	-0.610106	2.261576
H	-3.556604	-1.486679	1.861417
H	-1.986976	-2.319365	1.789389
C	-2.947614	-2.028203	-0.768896
H	-4.042755	-2.025015	-0.666060
H	-2.698365	-1.858315	-1.822796
H	-2.588775	-3.029499	-0.496805
C	-3.884679	1.001763	0.691361
H	-4.793291	0.400010	0.542328
H	-3.630369	0.986015	1.757533
H	-4.121838	2.038588	0.418965
C	-3.040631	0.723935	-1.641745
H	-2.230572	0.339430	-2.276537
H	-3.979958	0.247271	-1.955106
H	-3.128603	1.802743	-1.824938
H	0.761115	2.659881	1.143822
H	1.624098	-1.181740	-0.119733

81
I07.xyz

Pd	0.031158	-0.582508	-0.766844
I	-0.079482	-3.566883	-1.694336
P	-1.931618	0.584413	-0.438574
C	-1.766509	2.386362	-0.802946
C	-3.597572	0.206171	-1.147434
C	-2.301060	0.593193	1.371148
C	-2.232116	3.395453	0.049812
C	-1.142510	2.749347	-2.004371
C	-4.638981	1.139651	-1.239870
C	-3.812380	-1.096796	-1.614285

C	-3.527607	0.214066	1.932640
C	-1.252506	0.875918	2.261075
C	-2.086779	4.738564	-0.298963
H	-2.713699	3.131062	0.995536
C	-1.013969	4.089263	-2.359525
H	-0.743406	1.964933	-2.655329
C	-5.871886	0.769750	-1.774536
H	-4.483673	2.164791	-0.892087
C	-5.048422	-1.470160	-2.140406
H	-2.985435	-1.814560	-1.573198
C	-3.685588	0.114310	3.315560
H	-4.367742	-0.021541	1.275466
C	-1.383402	0.783184	3.646458
O	-0.040818	1.251049	1.711040
C	-1.484469	5.087943	-1.506560
H	-2.450440	5.515665	0.377313
H	-0.524874	4.355963	-3.298758
C	-6.080794	-0.536203	-2.220862
H	-6.675699	1.506047	-1.844201
H	-5.201171	-2.489919	-2.500191
C	-2.618259	0.391233	4.167426
H	-4.650595	-0.184559	3.729311
C	-0.178597	1.103534	4.485620
C	1.092094	0.814077	2.369426
H	-1.370114	6.139675	-1.778491
H	-7.047323	-0.822554	-2.640809
H	-2.738470	0.307371	5.250731
C	1.082127	0.717410	3.763101
H	-0.159823	2.187785	4.706134
H	-0.237264	0.604008	5.464145
C	2.207854	0.477209	1.583766
C	2.235585	0.262037	4.402360
P	2.035447	0.420466	-0.260450
C	3.349055	0.031318	2.267587
C	3.365156	-0.074036	3.658092
H	2.243511	0.173899	5.491833
C	3.707570	-0.183136	-0.774525
C	2.156661	2.191786	-0.763551
H	4.236398	-0.256583	1.700902
H	4.267340	-0.425136	4.162250
C	4.868262	0.602074	-0.703311
C	3.804486	-1.491110	-1.263075
C	1.903977	3.263833	0.100229
C	2.467670	2.467074	-2.105221
C	6.097433	0.081911	-1.102003
H	4.806138	1.628959	-0.331562
C	5.036138	-2.015258	-1.656683
H	2.891435	-2.091316	-1.349475
C	1.975235	4.577737	-0.363263
H	1.656122	3.079320	1.147171
C	2.554029	3.778968	-2.561083
H	2.656281	1.640048	-2.796477
C	6.184116	-1.228894	-1.576177

H	6.994624	0.702084	-1.042768
H	5.095742	-3.036939	-2.037582
C	2.307751	4.841790	-1.689977
H	1.770409	5.401463	0.324344
H	2.812531	3.972675	-3.604753
H	7.148495	-1.633587	-1.890409
H	2.368726	5.871991	-2.047440
C	-0.285044	-3.088161	0.376860
C	0.863639	-2.842419	1.137658
C	-1.556302	-3.069139	0.957804
C	0.723820	-2.556807	2.497237
H	1.854363	-2.871505	0.680636
C	-1.673844	-2.783985	2.318590
H	-2.445430	-3.270336	0.358097
C	-0.539044	-2.522411	3.086133
H	1.617288	-2.344481	3.089240
H	-2.665959	-2.755728	2.773477
H	-0.639988	-2.290304	4.148917

81
I08.xyz

Pd	0.246121	-0.936432	-0.593722
I	-1.177229	-3.237899	-0.382515
P	-1.937046	0.266191	-0.275524
C	-2.247334	2.090107	-0.268585
C	-3.402667	-0.244191	-1.261291
C	-2.310894	-0.199435	1.460569
C	-2.670837	2.798029	0.862382
C	-2.137699	2.769166	-1.489989
C	-4.688957	0.204271	-0.926943
C	-3.224184	-1.008616	-2.419428
C	-3.376759	-0.987956	1.901109
C	-1.330177	0.139827	2.395977
C	-2.997888	4.151019	0.767284
H	-2.769573	2.288568	1.823549
C	-2.469721	4.116966	-1.583761
H	-1.803800	2.229182	-2.381375
C	-5.781210	-0.135017	-1.721671
H	-4.834988	0.830130	-0.041706
C	-4.317058	-1.333882	-3.221893
H	-2.223752	-1.359816	-2.682510
C	-3.435960	-1.408071	3.230186
H	-4.139898	-1.311115	1.190806
C	-1.354515	-0.268394	3.726480
O	-0.278146	0.907746	1.920211
C	-2.905733	4.812234	-0.455259
H	-3.337501	4.686796	1.656553
H	-2.381562	4.629440	-2.544213
C	-5.595814	-0.905317	-2.870663
H	-6.779827	0.211323	-1.447833
H	-4.166214	-1.932117	-4.122350
C	-2.431976	-1.058630	4.132557
H	-4.265278	-2.035724	3.559261

C	-0.219466	0.163915	4.614070
C	0.956842	0.645611	2.475313
H	-3.169506	5.869331	-0.530016
H	-6.451708	-1.166920	-3.496307
H	-2.477653	-1.408099	5.166857
C	1.052912	0.282042	3.818902
H	-0.459855	1.141130	5.073964
H	-0.089057	-0.534112	5.453362
C	2.062290	0.735195	1.617964
C	2.326899	0.030087	4.331049
P	1.741395	0.852565	-0.195856
C	3.324002	0.482817	2.172431
C	3.453788	0.146311	3.519530
H	2.431147	-0.266826	5.377474
C	3.436267	0.962587	-0.915827
C	1.226785	2.595387	-0.499239
H	4.209179	0.501090	1.535010
H	4.443927	-0.056283	3.930358
C	4.282419	2.013646	-0.522334
C	3.874856	0.068036	-1.894775
C	0.838241	3.483673	0.508276
C	1.300872	3.054387	-1.823281
C	5.551976	2.141646	-1.076481
H	3.940317	2.738899	0.221608
C	5.147802	0.201049	-2.451832
H	3.221904	-0.739956	-2.221929
C	0.548261	4.812150	0.196625
H	0.773841	3.147992	1.544817
C	1.022528	4.382673	-2.128159
H	1.607587	2.369060	-2.618992
C	5.989586	1.230882	-2.041006
H	6.201552	2.959595	-0.758370
H	5.477144	-0.511122	-3.211102
C	0.649774	5.267508	-1.115811
H	0.244661	5.496232	0.991898
H	1.100424	4.729078	-3.160877
H	6.986236	1.332241	-2.475448
H	0.429204	6.310639	-1.352107
C	1.842429	-2.211969	-0.670610
C	2.149415	-2.863688	-1.873330
C	2.650826	-2.444634	0.445985
C	3.277118	-3.681634	-1.968656
H	1.507951	-2.733831	-2.749232
C	3.779917	-3.263982	0.347775
H	2.401892	-1.998571	1.412690
C	4.104836	-3.875068	-0.861554
H	3.502961	-4.178605	-2.916028
H	4.404039	-3.426514	1.230860
H	4.986959	-4.514331	-0.937668

184
I09.xyz

C	2.996417	0.600526	-2.684396

O	2.813766	-0.452387	-3.270542
O	4.153094	1.325423	-2.881300
C	4.996490	0.830902	-3.914683
H	4.540005	0.989905	-4.903130
H	5.932943	1.394553	-3.838531
H	5.187690	-0.247107	-3.805768
I	0.818082	0.123479	1.865548
B	1.307774	3.323242	-0.390030
O	0.358516	4.323077	-0.526955
O	1.765600	3.219968	0.907198
C	-0.046640	4.703833	0.820409
C	1.213427	4.333252	1.659833
C	-0.403761	6.174892	0.814253
C	-1.252432	3.854974	1.188894
C	2.265738	5.433162	1.651142
C	0.930394	3.875286	3.074488
H	-0.633257	6.524256	1.831932
H	0.406004	6.794043	0.409717
H	-1.295706	6.342083	0.194746
H	-1.004853	2.780531	1.144485
H	-1.610969	4.079989	2.203798
H	-2.074358	4.052824	0.487204
H	2.474034	5.775800	0.626673
H	1.950400	6.302254	2.246096
H	3.203055	5.052529	2.081609
H	0.456393	4.682118	3.654289
H	0.282362	2.991170	3.095024
H	1.869986	3.602631	3.574646
C	2.267175	3.493498	-2.759731
H	3.129344	4.146342	-2.536927
H	2.495392	2.958873	-3.690481
H	1.416055	4.169380	-2.947216
Cu	2.744473	-0.112679	-0.123275
Pd	0.203500	0.332490	-0.951132
P	4.910254	0.715627	0.500733
C	6.334652	-0.421968	0.266315
C	5.620742	2.307946	-0.078778
C	4.814284	0.881963	2.323352
C	6.561804	-0.902961	-1.028551
C	7.230698	-0.764833	1.284628
C	6.897736	2.412376	-0.647999
C	4.812498	3.451895	-0.013559
C	4.988582	2.043278	3.078253
C	4.417519	-0.280365	2.997171
C	7.680025	-1.683419	-1.310292
H	5.853285	-0.651790	-1.821931
C	8.335786	-1.568885	1.007952
H	7.071274	-0.394760	2.300396
C	7.349288	3.633414	-1.147307
H	7.551719	1.540459	-0.706035
C	5.275287	4.672509	-0.501165
H	3.818097	3.376983	0.429100
C	4.763233	2.021652	4.456315

H	5.294843	2.968683	2.584857
C	4.222365	-0.345048	4.372998
O	4.252709	-1.385627	2.193441
C	8.569250	-2.020756	-0.290432
H	7.848597	-2.039873	-2.328379
H	9.026401	-1.832645	1.811951
C	6.540363	4.766679	-1.079711
H	8.346685	3.697536	-1.587826
H	4.635723	5.556476	-0.437709
C	4.386034	0.840800	5.094686
H	4.892262	2.935485	5.039095
C	3.883430	-1.670452	4.996809
C	3.437285	-2.395045	2.640090
H	9.441430	-2.641380	-0.506834
H	6.896911	5.721028	-1.472644
H	4.223236	0.831098	6.175384
C	3.253716	-2.612768	4.006383
H	4.803195	-2.124797	5.411782
H	3.217299	-1.530536	5.862220
C	2.817641	-3.165012	1.645822
C	2.473924	-3.706657	4.383464
P	2.826757	-2.524526	-0.089637
C	2.079873	-4.277681	2.068312
C	1.918896	-4.552952	3.425152
H	2.308306	-3.898096	5.446865
C	1.370620	-3.418501	-0.785458
C	4.242386	-3.370656	-0.896539
H	1.597141	-4.916840	1.326998
H	1.335120	-5.420850	3.735940
C	0.134247	-3.230298	-0.143291
C	1.425429	-4.249698	-1.908913
C	4.456183	-3.131133	-2.265070
C	5.107179	-4.230588	-0.210380
C	-1.005406	-3.883277	-0.601929
H	0.081422	-2.584431	0.739393
C	0.270907	-4.875590	-2.384615
H	2.371680	-4.423635	-2.422722
C	5.482938	-3.788518	-2.938429
H	3.810623	-2.427579	-2.801459
C	6.150589	-4.864504	-0.885347
H	4.961681	-4.413009	0.857336
C	-0.947133	-4.698515	-1.734148
H	-1.951155	-3.755910	-0.072160
H	0.333871	-5.513906	-3.268637
C	6.330691	-4.659953	-2.252154
H	5.626030	-3.610699	-4.007223
H	6.820820	-5.530732	-0.337683
H	-1.848939	-5.192085	-2.102340
H	7.138947	-5.170235	-2.780918
P	-2.188086	0.286298	-0.766514
C	-3.165014	-0.948649	-1.730879
C	-2.835847	0.195340	0.946534
C	-2.851806	1.866060	-1.441263

C	-3.272183	-0.760720	-3.116989
C	-3.751675	-2.082050	-1.157530
C	-2.570906	-0.955931	1.700775
C	-3.495341	1.265541	1.563295
C	-1.944917	2.810723	-1.924681
C	-4.225664	2.172662	-1.514301
C	-3.931502	-1.697896	-3.909099
H	-2.819648	0.116993	-3.586779
C	-4.405275	-3.022092	-1.953455
H	-3.723555	-2.228546	-0.077771
C	-3.013873	-1.063176	3.016476
H	-1.990301	-1.768935	1.258060
C	-3.906383	1.170985	2.891454
H	-3.682390	2.188334	1.010359
C	-2.369682	4.027682	-2.452111
H	-0.880182	2.589086	-1.845667
C	-4.675928	3.376217	-2.065022
O	-5.104587	1.238172	-1.011646
C	-4.490280	-2.838213	-3.332639
H	-3.989767	-1.538081	-4.987890
H	-4.867802	-3.894391	-1.484794
C	-3.682088	0.001751	3.617004
H	-2.810863	-1.974954	3.581918
H	-4.420124	2.014719	3.358419
C	-3.730041	4.298936	-2.521986
H	-1.633362	4.757499	-2.791275
C	-6.147438	3.629854	-2.190627
C	-6.385784	1.636760	-0.687297
H	-5.002922	-3.575769	-3.954629
H	-4.030496	-0.078691	4.648971
H	-4.084821	5.244009	-2.942579
C	-6.940897	2.807257	-1.221624
H	-6.367141	4.700644	-2.056982
H	-6.474231	3.397618	-3.222248
C	-7.117945	0.797101	0.167241
C	-8.246991	3.144286	-0.868477
P	-6.376609	-0.784016	0.786658
C	-8.442467	1.150723	0.458483
C	-9.004402	2.322209	-0.037332
H	-8.679679	4.061101	-1.277696
C	-7.606703	-1.926506	0.002260
C	-6.852997	-0.804181	2.563570
H	-9.035989	0.487234	1.092888
H	-10.033205	2.587384	0.211118
C	-7.529723	-2.022136	-1.397345
C	-8.554689	-2.704927	0.676679
C	-6.651533	-2.008635	3.260699
C	-7.214444	0.332100	3.300198
C	-8.376788	-2.871874	-2.101853
H	-6.793155	-1.418883	-1.938177
C	-9.398894	-3.562328	-0.031627
H	-8.647318	-2.638322	1.763287
C	-6.843055	-2.083946	4.637208

H	-6.326767	-2.899122	2.713529
C	-7.392152	0.260515	4.682182
H	-7.342123	1.291862	2.794847
C	-9.312993	-3.651157	-1.419606
H	-8.300866	-2.929288	-3.190284
H	-10.135185	-4.160431	0.510122
C	-7.215191	-0.946489	5.355960
H	-6.689979	-3.034082	5.153673
H	-7.672572	1.159757	5.235494
H	-9.974628	-4.323433	-1.969516
H	-7.358939	-1.000874	6.436866
C	-0.073974	0.005975	-2.940488
C	-0.042989	0.988972	-3.935128
C	-0.234585	-1.330864	-3.319480
C	-0.165225	0.635885	-5.282953
H	0.096957	2.042018	-3.680394
C	-0.334342	-1.683441	-4.664951
H	-0.290559	-2.113306	-2.559852
C	-0.304544	-0.700623	-5.654338
H	-0.142044	1.418575	-6.046492
H	-0.451517	-2.737056	-4.934887
H	-0.390530	-0.973752	-6.708617
C	1.945435	2.575960	-1.603470
C	2.174903	1.220829	-1.616480

114
I10.xyz

C	0.653679	1.714551	2.404863
C	2.629777	2.193024	0.420769
C	-0.047333	3.241921	0.035846
C	0.389015	2.860971	3.161965
C	0.880309	0.496596	3.056828
C	3.522258	2.098965	1.496958
C	3.128787	2.550783	-0.843468
C	0.546263	4.420907	-0.425887
C	-1.440591	3.145499	-0.065575
C	0.364929	2.788454	4.554160
H	0.212666	3.816300	2.659940
C	0.877454	0.429866	4.447875
H	1.059566	-0.399743	2.451682
C	4.882866	2.348605	1.312735
H	3.162903	1.826766	2.491738
C	4.484697	2.814623	-1.016882
H	2.443097	2.625370	-1.691518
C	-0.226612	5.421572	-1.013741
H	1.627418	4.546377	-0.353268
C	-2.237000	4.113347	-0.674335
O	-2.015666	2.015574	0.481089
C	0.619976	1.577358	5.198198
H	0.156077	3.686003	5.140430
H	1.059435	-0.524431	4.947603
C	5.368792	2.705067	0.057288
H	5.563910	2.264866	2.162611

H	4.854024	3.093123	-2.006411
C	-1.602131	5.257501	-1.160995
H	0.255243	6.329300	-1.379900
C	-3.717843	3.865299	-0.767565
C	-3.144396	1.525690	-0.144018
H	0.606380	1.524840	6.289047
H	6.433883	2.899125	-0.085181
H	-2.199043	6.029768	-1.652490
C	-4.014968	2.391411	-0.805231
H	-4.226119	4.318658	0.104732
H	-4.141089	4.370853	-1.647898
C	-3.326582	0.137707	-0.083945
C	-5.111449	1.825389	-1.458459
P	-1.931194	-0.828327	0.630252
C	-4.432817	-0.391668	-0.758147
C	-5.317629	0.447016	-1.436150
H	-5.805598	2.476467	-1.995661
C	-2.401223	-2.597190	0.483795
C	-2.146456	-0.654245	2.464337
H	-4.579074	-1.471826	-0.797353
H	-6.168574	0.015751	-1.965662
C	-3.644849	-3.084710	0.913045
C	-1.418986	-3.503578	0.071804
C	-2.698656	0.479239	3.073623
C	-1.707927	-1.708257	3.282658
C	-3.915164	-4.450100	0.877750
H	-4.399506	-2.393026	1.296884
C	-1.687380	-4.872492	0.047595
H	-0.436851	-3.120690	-0.216598
C	-2.818926	0.549341	4.461089
H	-3.048602	1.315435	2.468523
C	-1.836613	-1.638257	4.666992
H	-1.278374	-2.606262	2.831145
C	-2.938122	-5.345752	0.438188
H	-4.889301	-4.819090	1.205065
H	-0.915520	-5.570746	-0.283911
C	-2.393738	-0.507684	5.263174
H	-3.254676	1.441527	4.916067
H	-1.501422	-2.476341	5.282270
H	-3.151590	-6.416305	0.411928
H	-2.496701	-0.450745	6.348835
C	1.488461	0.096450	-2.099342
C	2.719607	-0.450808	-1.959148
C	1.079645	0.962215	-3.218472
B	3.036025	-1.384169	-0.771447
C	3.844116	-0.173112	-2.926210
O	0.770257	2.142458	-3.137404
O	1.010654	0.273401	-4.384571
O	4.319681	-1.803175	-0.471790
O	2.094421	-1.959784	0.080093
H	3.583127	0.585102	-3.678453
H	4.744924	0.169409	-2.392065
H	4.143157	-1.085056	-3.468736

C	0.439921	1.001631	-5.470515
C	4.249837	-2.539589	0.777495
C	2.775429	-3.038033	0.777910
H	0.376230	0.294660	-6.303462
H	-0.561786	1.368830	-5.208771
H	1.065602	1.862117	-5.745691
C	5.293581	-3.635758	0.753842
C	4.526443	-1.549537	1.898842
C	2.585792	-4.296690	-0.057380
C	2.149511	-3.211684	2.145701
H	5.201161	-4.283593	1.638313
H	5.209169	-4.259695	-0.143847
H	6.301127	-3.199119	0.762587
H	3.741118	-0.781357	1.951469
H	4.591675	-2.049291	2.876327
H	5.479591	-1.038522	1.708935
H	3.040237	-4.186126	-1.052089
H	3.024711	-5.180176	0.427879
H	1.513017	-4.486758	-0.198961
H	2.669450	-3.996264	2.715698
H	2.176778	-2.287259	2.735942
H	1.098160	-3.517087	2.040684
C	-0.743809	-1.329374	-2.263718
C	-0.124736	-2.511974	-2.694338
C	-1.859500	-0.877186	-2.983459
C	-0.628994	-3.244330	-3.768531
H	0.779556	-2.861953	-2.184409
C	-2.368472	-1.607286	-4.063169
H	-2.341616	0.069582	-2.716302
C	-1.758950	-2.797319	-4.456294
H	-0.129094	-4.165957	-4.080396
H	-3.245618	-1.235057	-4.601344
H	-2.153776	-3.368120	-5.300019
P	0.854219	1.731109	0.583003
Pd	-0.009685	-0.159448	-0.721282

114
I11.xyz

B	-3.192915	0.255743	-0.743381
C	-0.378958	2.603211	-2.127503
O	-0.446048	3.766292	-2.471958
O	0.556460	1.760715	-2.666762
C	1.311311	2.322637	-3.735937
H	1.968276	1.521972	-4.094714
H	0.654390	2.670194	-4.545816
H	1.916362	3.176375	-3.396525
C	-1.725887	0.098185	-2.877835
H	-0.735676	-0.343103	-3.056504
H	-2.484635	-0.675361	-3.057908
O	-4.178994	-0.498506	-1.368856
O	-3.532631	0.574664	0.556291
C	-5.362981	-0.391146	-0.531452
C	-4.748802	-0.142839	0.878449

C	-5.585393	0.725547	1.795062
H	-5.733660	1.730004	1.380196
H	-6.570616	0.270057	1.976916
H	-5.084212	0.839779	2.765960
C	-4.338259	-1.432263	1.571513
H	-5.207736	-1.992529	1.946110
H	-3.776679	-2.083340	0.888289
H	-3.687628	-1.197882	2.425882
C	-6.148079	0.809547	-1.041313
H	-7.099418	0.933657	-0.504494
H	-5.566455	1.736608	-0.933152
H	-6.372920	0.673642	-2.107581
C	-6.178812	-1.659573	-0.657500
H	-5.590600	-2.551646	-0.410808
H	-7.052704	-1.625113	0.010361
H	-6.549373	-1.771031	-1.685996
H	-1.871717	0.861982	-3.660487
Pd	-0.213547	0.214313	-0.191170
P	-0.015615	-2.121830	0.177243
C	0.679042	-2.553604	1.831399
C	-1.455570	-3.267968	0.052996
C	1.134571	-2.949777	-1.029175
C	1.462560	-3.683113	2.098053
C	0.397968	-1.659719	2.874677
C	-1.842819	-4.157374	1.063268
C	-2.228860	-3.186838	-1.114271
C	1.188613	-4.332935	-1.252774
C	2.012630	-2.155040	-1.779656
C	1.955348	-3.911584	3.382361
H	1.702365	-4.386119	1.296046
C	0.884634	-1.892963	4.159121
H	-0.191851	-0.761331	2.658145
C	-2.978201	-4.953735	0.901162
H	-1.263350	-4.225498	1.986999
C	-3.352925	-3.989077	-1.278258
H	-1.952272	-2.472369	-1.894190
C	2.123114	-4.879089	-2.132211
H	0.490471	-4.988738	-0.725530
C	2.958549	-2.668068	-2.670971
O	1.896863	-0.801170	-1.623941
C	1.668185	-3.017950	4.414365
H	2.572456	-4.791845	3.576706
H	0.664950	-1.183791	4.959916
C	-3.732728	-4.875751	-0.268224
H	-3.273240	-5.639743	1.698264
H	-3.948446	-3.902182	-2.189750
C	3.012103	-4.053221	-2.823459
H	2.158452	-5.959412	-2.281822
C	3.813880	-1.685867	-3.430734
C	3.032287	-0.040895	-1.739429
H	2.063669	-3.194131	5.416853
H	-4.622022	-5.498034	-0.389891
H	3.743694	-4.488656	-3.508985

C	4.028606	-0.420919	-2.640190
H	4.776026	-2.139224	-3.711253
H	3.314773	-1.440673	-4.388146
C	3.125087	1.074449	-0.900794
C	5.148910	0.404065	-2.742679
P	1.896748	1.216434	0.489109
C	4.261715	1.880015	-1.037211
C	5.253622	1.558284	-1.963650
H	5.949538	0.137652	-3.437481
C	1.738053	3.014206	0.791415
C	3.045960	0.589843	1.804258
H	4.382721	2.751614	-0.388922
H	6.133867	2.196572	-2.056277
C	1.831231	3.934113	-0.261868
C	1.264204	3.467566	2.034076
C	3.423644	-0.757410	1.690261
C	3.565707	1.342932	2.862765
C	1.472321	5.267491	-0.077747
H	2.158758	3.600123	-1.248184
C	0.934852	4.806041	2.224102
H	1.114443	2.757238	2.852266
C	4.286525	-1.340098	2.611265
H	3.037192	-1.358952	0.860574
C	4.420836	0.752891	3.796189
H	3.324078	2.402860	2.957744
C	1.033108	5.710827	1.167450
H	1.519642	5.954935	-0.924053
H	0.562815	5.136410	3.196141
C	4.781255	-0.587112	3.677662
H	4.566918	-2.389520	2.499088
H	4.816775	1.355948	4.616360
H	0.745430	6.754026	1.309827
H	5.451948	-1.043158	4.408700
C	-1.892891	2.868122	-0.147951
C	-2.711795	3.904722	-0.622216
C	-1.755648	2.723318	1.235488
C	-3.371682	4.757675	0.260415
H	-2.831871	4.039756	-1.698113
C	-2.415681	3.570587	2.122322
H	-1.137392	1.901264	1.612517
C	-3.226341	4.595403	1.638801
H	-4.006720	5.554997	-0.133134
H	-2.292644	3.428119	3.199247
H	-3.744540	5.263757	2.330450
C	-1.901237	0.690607	-1.484219
C	-1.239558	1.920589	-1.116765

115
TS1.xyz

C	3.241228	-0.178625	2.673687
C	2.846677	0.830065	1.850578
B	3.567067	-1.532655	2.026822
C	2.471593	2.140103	2.384360

O	1.334793	2.593236	2.432881
O	3.544697	2.868775	2.800487
C	3.218546	4.173262	3.274871
H	4.172928	4.656787	3.509650
H	2.590355	4.120912	4.175585
H	2.677815	4.748998	2.510493
C	3.358162	-0.061043	4.171861
H	3.124504	0.946621	4.542814
H	4.372659	-0.318040	4.516579
O	4.046119	-2.630189	2.711768
O	3.424511	-1.785837	0.661992
C	4.463431	-3.602266	1.720452
C	3.580469	-3.226100	0.491860
C	2.189840	-3.835840	0.571034
H	1.730401	-3.652874	1.553199
H	2.209932	-4.920525	0.391674
H	1.541054	-3.381126	-0.190502
C	4.228454	-3.507528	-0.847158
H	4.486663	-4.573904	-0.931863
H	5.140921	-2.917174	-0.993021
H	3.541147	-3.272012	-1.671122
C	4.222029	-4.992863	2.268738
H	4.430987	-5.757137	1.505377
H	3.189730	-5.120632	2.614917
H	4.886546	-5.182097	3.122234
C	5.946660	-3.371946	1.469574
H	6.127967	-2.375741	1.042761
H	6.368437	-4.123297	0.786989
H	6.489777	-3.433872	2.421629
H	2.687204	-0.774909	4.678359
Cu	2.578457	0.509279	-0.104961
I	3.687367	0.562459	-2.482944
C	3.495877	2.274825	-0.861459
C	4.704946	2.717012	-0.308096
C	2.439039	3.166278	-1.089111
C	4.817254	4.044327	0.088903
H	5.531447	2.021765	-0.157828
C	2.568422	4.489720	-0.665820
H	1.519832	2.836031	-1.575268
C	3.755315	4.936454	-0.088076
H	5.747118	4.380591	0.553661
H	1.728257	5.172278	-0.813337
H	3.858233	5.978582	0.220502
P	0.331399	-0.254319	-0.466629
C	-0.437373	-1.016379	1.016859
C	-0.598372	1.275865	-0.878914
C	-0.093539	-1.411573	-1.821173
C	-1.093067	-2.254618	0.972827
C	-0.274918	-0.377213	2.255628
C	-0.844123	1.589110	-2.224108
C	-0.862472	2.236738	0.105332
C	0.951671	-2.097549	-2.447178
C	-1.408607	-1.704372	-2.223076

C	-1.598544	-2.829811	2.136505
H	-1.207213	-2.790472	0.028230
C	-0.788485	-0.954152	3.415643
H	0.265358	0.570050	2.323239
C	-1.353474	2.837717	-2.575361
H	-0.627155	0.857199	-3.006786
C	-1.370940	3.482766	-0.254455
H	-0.639019	2.036734	1.153147
C	0.705566	-3.058144	-3.425825
H	1.971655	-1.875075	-2.127585
C	-1.680511	-2.662735	-3.204782
O	-2.424140	-1.026212	-1.589996
C	-1.448986	-2.180969	3.362306
H	-2.110564	-3.793173	2.081888
H	-0.655446	-0.440722	4.370385
C	-1.616933	3.790417	-1.591938
H	-1.548542	3.064136	-3.626062
H	-1.573678	4.220648	0.525012
C	-0.607328	-3.336659	-3.792862
H	1.534955	-3.590371	-3.894579
C	-3.107690	-2.924179	-3.582428
C	-3.659002	-1.649674	-1.506899
H	-1.840936	-2.635473	4.275332
H	-2.019854	4.767274	-1.867404
H	-0.817243	-4.092908	-4.553831
C	-4.029599	-2.624401	-2.437356
H	-3.383185	-2.294769	-4.450242
H	-3.234760	-3.961924	-3.925153
C	-4.508727	-1.225683	-0.472527
C	-5.268320	-3.248204	-2.281541
P	-3.933351	0.123925	0.665393
C	-5.746027	-1.871886	-0.358980
C	-6.119361	-2.882852	-1.243144
H	-5.564244	-4.022559	-2.993847
C	-5.245931	0.036487	1.966248
C	-4.482743	1.617894	-0.274478
H	-6.428280	-1.581327	0.441804
H	-7.085780	-3.376081	-1.126867
C	-4.936015	-0.684114	3.128500
C	-6.513870	0.628494	1.860657
C	-4.596165	2.826851	0.432564
C	-4.720458	1.629329	-1.653836
C	-5.871183	-0.822078	4.153393
H	-3.945391	-1.137725	3.226106
C	-7.444349	0.499809	2.890271
H	-6.776926	1.192253	0.961871
C	-4.971994	4.000562	-0.213238
H	-4.398105	2.843093	1.508732
C	-5.081081	2.810364	-2.304081
H	-4.636269	0.705907	-2.231617
C	-7.126845	-0.227466	4.038177
H	-5.612859	-1.387703	5.051268
H	-8.426951	0.966979	2.793165

C	-5.216498	3.997293	-1.587727
H	-5.069548	4.925497	0.359893
H	-5.266494	2.796371	-3.380626
H	-7.857167	-0.326159	4.843984
H	-5.509803	4.917717	-2.096884

115
TS2.xyz

C	-2.803064	0.996103	2.275486
C	-2.738370	-0.293814	1.876704
B	-3.121065	2.107589	1.249309
C	-2.418023	-1.462150	2.718747
O	-1.615840	-2.323080	2.389895
O	-3.131745	-1.525261	3.861699
C	-2.900511	-2.705584	4.637115
H	-3.549380	-2.618871	5.512918
H	-1.848706	-2.778117	4.942897
H	-3.156589	-3.603218	4.058520
C	-2.557922	1.445096	3.690611
H	-2.156372	0.657272	4.338204
H	-3.498026	1.803413	4.140700
O	-3.542615	3.360035	1.630112
O	-3.022165	1.958175	-0.121946
C	-4.020778	4.025529	0.428755
C	-3.236553	3.286878	-0.702339
C	-1.864649	3.893177	-0.947411
H	-1.308886	4.015473	-0.006972
H	-1.944228	4.876055	-1.433121
H	-1.280287	3.237632	-1.606745
C	-4.003340	3.133125	-1.997967
H	-4.290673	4.118370	-2.394354
H	-4.913634	2.534405	-1.870178
H	-3.379782	2.641160	-2.755480
C	-3.720519	5.505320	0.542753
H	-3.987985	6.030171	-0.386109
H	-2.662035	5.694059	0.755325
H	-4.309690	5.946372	1.357403
C	-5.522770	3.792136	0.366741
H	-5.763556	2.722952	0.287754
H	-5.981253	4.313965	-0.484703
H	-5.987199	4.168393	1.287462
H	-1.867036	2.301819	3.710855
Cu	-2.581902	-0.897980	0.016945
I	-2.940395	-2.272143	-2.154952
C	-4.411946	-1.185197	0.670530
C	-5.398743	-0.275572	0.290117
C	-4.766523	-2.418276	1.219856
C	-6.743749	-0.616056	0.431607
H	-5.118899	0.684792	-0.145804
C	-6.115059	-2.749667	1.359072
H	-3.997428	-3.141234	1.504093
C	-7.107312	-1.850974	0.969806
H	-7.512940	0.092514	0.113152

H	-6.387651	-3.725122	1.769003
H	-8.160975	-2.112384	1.083432
P	-0.310207	-0.344373	-0.365330
C	0.429074	0.777737	0.882615
C	0.730688	-1.849156	-0.486367
C	-0.045273	0.507210	-1.962859
C	0.875353	2.064219	0.554849
C	0.450205	0.383718	2.229453
C	0.833189	-2.471273	-1.739843
C	1.294182	-2.470529	0.632521
C	-1.159967	0.852080	-2.733553
C	1.231229	0.847837	-2.446936
C	1.361637	2.920843	1.540947
H	0.847781	2.410770	-0.480020
C	0.953304	1.233118	3.211042
H	0.055624	-0.592670	2.514694
C	1.500064	-3.686153	-1.867743
H	0.374366	-2.011501	-2.617928
C	1.955170	-3.689580	0.497638
H	1.224964	-2.010808	1.618203
C	-1.015780	1.525819	-3.944297
H	-2.151702	0.586205	-2.363168
C	1.394035	1.537129	-3.652947
O	2.316205	0.488762	-1.677206
C	1.408362	2.506766	2.871423
H	1.709690	3.918240	1.262622
H	0.969185	0.903323	4.252221
C	2.060117	-4.301202	-0.749200
H	1.572420	-4.157473	-2.850021
H	2.393807	-4.161541	1.378741
C	0.255339	1.871372	-4.389127
H	-1.895873	1.777710	-4.538317
C	2.777621	1.869188	-4.121686
C	3.479059	1.232324	-1.808835
H	1.790139	3.178956	3.643289
H	2.580965	-5.255681	-0.848815
H	0.382355	2.406465	-5.334024
C	3.730550	1.971028	-2.969108
H	3.131096	1.087536	-4.821172
H	2.774565	2.800397	-4.708058
C	4.394186	1.169730	-0.743941
C	4.894080	2.739541	-3.027612
P	4.018055	0.090304	0.719057
C	5.553191	1.949079	-0.849520
C	5.796666	2.741061	-1.969712
H	5.090229	3.331989	-3.924905
C	5.366147	0.620948	1.871673
C	4.688548	-1.529188	0.131705
H	6.279328	1.939521	-0.035328
H	6.703554	3.345673	-2.021299
C	5.028978	1.568622	2.848076
C	6.688426	0.152958	1.820983
C	5.027435	-2.490705	1.098559

C	4.793204	-1.879086	-1.219339
C	5.985454	2.044788	3.744104
H	3.999532	1.933799	2.901194
C	7.641955	0.620211	2.723622
H	6.975929	-0.580923	1.063894
C	5.494158	-3.747363	0.724623
H	4.938483	-2.244019	2.161029
C	5.244685	-3.145163	-1.593036
H	4.527948	-1.157697	-1.995264
C	7.294180	1.568417	3.686616
H	5.704016	2.784375	4.496882
H	8.666457	0.245195	2.670283
C	5.605740	-4.080499	-0.626068
H	5.768498	-4.472924	1.493905
H	5.318874	-3.397175	-2.653330
H	8.042943	1.932788	4.392987
H	5.968867	-5.066910	-0.921816

81

TS3.xyz

Pd	0.048113	-0.746595	-0.816672
I	0.058594	-3.182539	-2.044350
P	-2.031239	0.474532	-0.349503
C	-1.987150	2.303215	-0.560898
C	-3.665014	0.032838	-1.077614
C	-2.357272	0.286322	1.453849
C	-2.432251	3.207464	0.411955
C	-1.488097	2.801430	-1.772960
C	-4.752576	0.915150	-1.138399
C	-3.810868	-1.266549	-1.582179
C	-3.557113	-0.194727	1.992484
C	-1.293090	0.504681	2.342845
C	-2.393047	4.580681	0.169293
H	-2.818811	2.835631	1.364751
C	-1.463127	4.171220	-2.018844
H	-1.111159	2.103924	-2.527673
C	-5.966585	0.497131	-1.680434
H	-4.648542	1.935618	-0.759321
C	-5.029349	-1.685888	-2.114130
H	-2.950653	-1.944958	-1.569830
C	-3.673627	-0.453857	3.358335
H	-4.405254	-0.384625	1.331117
C	-1.382744	0.253684	3.711631
O	-0.119681	0.981930	1.793797
C	-1.916268	5.064639	-1.047801
H	-2.743425	5.275503	0.935971
H	-1.070941	4.544096	-2.967259
C	-6.108949	-0.804678	-2.163400
H	-6.807943	1.192077	-1.725604
H	-5.130220	-2.701285	-2.502570
C	-2.592206	-0.235914	4.209140
H	-4.616059	-0.831330	3.758799
C	-0.180343	0.522600	4.570806

C	1.061453	0.617349	2.410728
H	-1.885828	6.140052	-1.236443
H	-7.061511	-1.128858	-2.587615
H	-2.680189	-0.446944	5.278006
C	1.091608	0.374913	3.784804
H	-0.242864	1.546567	4.985968
H	-0.172690	-0.146526	5.444661
C	2.194202	0.503691	1.587796
C	2.308821	0.004523	4.358025
P	1.966023	0.597663	-0.240727
C	3.397333	0.136902	2.204425
C	3.456011	-0.105714	3.576077
H	2.350529	-0.198535	5.431239
C	3.634745	0.168003	-0.895929
C	1.910429	2.405370	-0.592475
H	4.297215	0.015090	1.598855
H	4.404014	-0.393319	4.033937
C	4.751179	1.006600	-0.752794
C	3.776050	-1.041192	-1.587136
C	1.526111	3.361304	0.356214
C	2.203851	2.830729	-1.898595
C	5.985460	0.630631	-1.276921
H	4.648165	1.961698	-0.229652
C	5.014017	-1.418518	-2.108846
H	2.896468	-1.678578	-1.731625
C	1.458065	4.710424	0.011064
H	1.284229	3.055981	1.376055
C	2.147007	4.180002	-2.235951
H	2.497108	2.096352	-2.654679
C	6.119339	-0.584556	-1.952658
H	6.848451	1.289778	-1.160426
H	5.109169	-2.363000	-2.648133
C	1.775775	5.126688	-1.280370
H	1.154977	5.442183	0.763258
H	2.394591	4.493225	-3.252752
H	7.087656	-0.875513	-2.365219
H	1.726935	6.185270	-1.544499
C	0.218750	-2.527034	0.320531
C	1.465936	-2.740211	0.929019
C	-0.964673	-2.740605	1.045339
C	1.518870	-3.033997	2.290763
H	2.387998	-2.636395	0.353485
C	-0.890995	-3.026947	2.408919
H	-1.936045	-2.649935	0.555134
C	0.346432	-3.165660	3.037989
H	2.494212	-3.149555	2.769773
H	-1.818049	-3.143592	2.975616
H	0.398888	-3.393294	4.105028

114
TS4.xyz

Pd	-0.169866	0.253196	-0.508388
P	-0.632143	-1.885405	0.528665

C	-0.047071	-2.261255	2.228573
C	-2.384771	-2.431702	0.599259
C	0.190544	-3.168005	-0.501739
C	0.446700	-3.516458	2.604010
C	-0.159211	-1.254900	3.195682
C	-3.033252	-2.758915	1.798597
C	-3.132232	-2.407346	-0.590466
C	-0.441167	-4.253537	-1.115287
C	1.529266	-2.932128	-0.834066
C	0.812574	-3.760489	3.927028
H	0.538951	-4.309502	1.857052
C	0.189378	-1.504777	4.520862
H	-0.515294	-0.264377	2.893446
C	-4.397393	-3.049253	1.807982
H	-2.473427	-2.786779	2.736292
C	-4.492741	-2.705553	-0.576477
H	-2.630606	-2.172580	-1.532139
C	0.236574	-5.026748	-2.057588
H	-1.483997	-4.474639	-0.879871
C	2.222558	-3.664174	-1.794372
O	2.154353	-1.895883	-0.165056
C	0.676653	-2.758964	4.888228
H	1.199533	-4.741904	4.210147
H	0.098252	-0.711339	5.265649
C	-5.131763	-3.019203	0.623516
H	-4.887122	-3.303264	2.750724
H	-5.057648	-2.686917	-1.511039
C	1.548475	-4.719280	-2.412732
H	-0.273753	-5.864152	-2.536077
C	3.636836	-3.258725	-2.105899
C	3.071330	-1.162303	-0.891064
H	0.962216	-2.952192	5.924615
H	-6.200295	-3.243959	0.633691
H	2.063667	-5.306120	-3.177298
C	3.824251	-1.781584	-1.889283
H	4.336670	-3.816199	-1.454585
H	3.905314	-3.539109	-3.134868
C	3.167379	0.201655	-0.579087
C	4.702367	-0.983873	-2.625337
P	1.915855	0.877872	0.598010
C	4.055948	0.966522	-1.343837
C	4.817116	0.378160	-2.354137
H	5.296115	-1.439268	-3.421935
C	2.240503	2.686527	0.664940
C	2.570156	0.412719	2.265933
H	4.123595	2.043067	-1.180672
H	5.495932	0.995721	-2.944764
C	3.502710	3.224662	0.961629
C	1.148151	3.548903	0.524148
C	3.340168	-0.732966	2.501238
C	2.226309	1.226684	3.357988
C	3.672696	4.601703	1.072419
H	4.354225	2.557564	1.122108

C	1.319133	4.929388	0.641846
H	0.159455	3.120936	0.333285
C	3.766868	-1.045873	3.791478
H	3.619175	-1.387757	1.674834
C	2.661474	0.916304	4.643193
H	1.622982	2.125181	3.197318
C	2.580584	5.457018	0.906422
H	4.659579	5.011085	1.298086
H	0.461481	5.595615	0.521200
C	3.435591	-0.222618	4.865876
H	4.368808	-1.942698	3.954071
H	2.394916	1.571271	5.475955
H	2.715376	6.537107	0.994378
H	3.778295	-0.467756	5.873331
C	-1.595010	0.331552	-2.003792
C	-2.872351	0.707552	-1.669064
C	-1.341744	-0.500194	-3.207981
B	-3.156439	1.451220	-0.355088
C	-4.064772	0.342166	-2.519591
O	-0.847517	-1.613903	-3.204661
O	-1.693451	0.135856	-4.351779
O	-4.437270	1.686365	0.116518
O	-2.189924	1.957679	0.511080
H	-3.903030	-0.552826	-3.138978
H	-4.951442	0.164662	-1.893878
H	-4.329362	1.158113	-3.214181
C	-1.369050	-0.576901	-5.544555
C	-4.303122	2.123975	1.494065
C	-2.883632	2.764825	1.497335
H	-1.693009	0.059407	-6.373370
H	-0.286750	-0.757964	-5.604760
H	-1.885207	-1.545873	-5.581641
C	-5.432593	3.078809	1.815776
C	-4.379896	0.875050	2.359942
C	-2.885335	4.196835	0.981938
C	-2.133722	2.667656	2.809398
H	-5.307976	3.507151	2.821511
H	-5.490192	3.901481	1.093031
H	-6.394003	2.548619	1.794757
H	-3.546173	0.190514	2.143038
H	-4.363478	1.117150	3.432541
H	-5.312514	0.337340	2.143459
H	-3.435464	4.277083	0.033621
H	-3.336479	4.892831	1.703617
H	-1.851422	4.522178	0.800554
H	-2.660676	3.215056	3.605347
H	-2.010157	1.626517	3.131114
H	-1.131685	3.109208	2.705482
C	-0.049815	1.609999	-2.113349
C	-0.510396	2.938566	-2.149656
C	0.983613	1.254583	-3.005321
C	0.080246	3.883510	-2.981015
H	-1.351047	3.227627	-1.513104

C	1.581923	2.205308	-3.833307
H	1.331260	0.215860	-3.045589
C	1.139376	3.527132	-3.821533
H	-0.291557	4.911956	-2.977651
H	2.401045	1.904688	-4.492618
H	1.599282	4.270924	-4.475467

Photoisomerization of alkenyl boronates, DFT analysis

38
3a.E_s-cis.xyz

B	-0.268612	0.583821	-0.040194
C	1.009486	1.495782	-0.091713
O	-0.969824	0.361063	1.126062
C	0.715013	2.955743	-0.192197
C	2.255730	0.967796	-0.043107
O	-0.973739	0.252645	-1.176180
C	-2.315678	-0.023293	0.734546
H	1.601159	3.601910	-0.219199
H	0.116015	3.159747	-1.094845
H	0.085013	3.274604	0.654363
C	3.556912	1.702409	-0.076526
C	2.291416	-0.504218	0.050615
C	-2.120214	-0.526025	-0.731590
C	-2.819826	-1.074380	1.700656
C	-3.165443	1.236489	0.819027
H	4.172455	1.383785	-0.930442
H	4.156671	1.491656	0.820918
H	3.422335	2.787482	-0.144710
O	3.549113	-0.994796	0.090112
O	1.306446	-1.226544	0.088762
C	-3.282006	-0.234813	-1.658936
C	-1.729371	-1.993471	-0.808435
H	-2.926179	-0.644334	2.705616
H	-3.805942	-1.451211	1.390466
H	-2.129792	-1.923117	1.771040
H	-4.225060	1.034617	0.606642
H	-2.813140	2.002141	0.112863
H	-3.094495	1.655538	1.831693
C	3.626676	-2.418292	0.180944
H	-3.065814	-0.618410	-2.664991
H	-4.199089	-0.727644	-1.303029
H	-3.478656	0.840362	-1.747082
H	-1.400414	-2.225059	-1.830326
H	-2.578067	-2.649367	-0.566444
H	-0.896461	-2.221807	-0.132798
H	3.153273	-2.891676	-0.689080
H	3.124328	-2.779725	1.087619
H	4.692769	-2.659895	0.212932

38
3a.E_s-trans.xyz

B	0.098746	-0.595664	-0.021373

C	-1.275342	-1.345881	-0.041885
O	0.744951	-0.185554	1.122126
C	-1.083135	-2.832032	-0.092705
C	-2.492011	-0.750055	-0.027980
O	0.897052	-0.562986	-1.142036
C	2.149933	-0.031960	0.780106
H	-2.011923	-3.412740	-0.130182
H	-0.474534	-3.103211	-0.970381
H	-0.507292	-3.175653	0.782746
C	-3.822297	-1.435362	-0.036689
C	-2.618166	0.722369	0.020026
C	2.115784	0.140098	-0.774343
C	2.701206	1.158897	1.535784
C	2.842615	-1.313247	1.219215
H	-4.420585	-1.116339	-0.902489
H	-4.410316	-1.147917	0.846614
H	-3.743533	-2.526561	-0.057822
O	-1.423244	1.373645	-0.061451
O	-3.670315	1.319276	0.125876
C	3.277956	-0.498520	-1.506652
C	1.942076	1.583954	-1.215109
H	2.686233	0.960710	2.615645
H	3.743054	1.360185	1.245494
H	2.109968	2.064754	1.355291
H	3.929504	-1.268414	1.062170
H	2.452720	-2.184812	0.674506
H	2.658699	-1.477196	2.289148
C	-1.521436	2.788653	0.088467
H	3.173216	-0.341382	-2.588289
H	4.231705	-0.048365	-1.193682
H	3.331471	-1.579158	-1.330246
H	1.730127	1.612189	-2.291980
H	2.845541	2.181183	-1.027777
H	1.097471	2.055080	-0.697587
H	-2.082754	3.046968	0.995190
H	-2.028482	3.240555	-0.774783
H	-0.494670	3.161918	0.159759

38
3a.Zs-cis.xyz

B	0.607695	0.263259	-0.038354
C	-0.904782	0.656022	-0.094372
O	1.157610	-1.002588	-0.060616
C	-1.146796	2.129664	-0.291289
C	-1.917825	-0.250944	0.033284
O	1.573049	1.247978	0.020472
C	2.592120	-0.845253	-0.259719
H	-1.757392	2.557864	0.515342
H	-0.200148	2.678622	-0.341261
H	-1.718433	2.329513	-1.208065
C	-1.723311	-1.720409	0.251179
C	-3.335321	0.198692	-0.027514
C	2.844157	0.594350	0.280716

C	3.309063	-1.943523	0.495872
C	2.841734	-0.968971	-1.754728
H	-2.210976	-2.299325	-0.546788
H	-2.215323	-2.041390	1.180908
H	-0.670002	-2.001820	0.291683
O	-4.185026	-0.852510	0.151189
O	-3.750343	1.326454	-0.211115
C	3.938354	1.357762	-0.434813
C	3.057507	0.633199	1.786290
H	3.078670	-2.921449	0.052919
H	4.398728	-1.802053	0.445490
H	3.013011	-1.976093	1.550839
H	2.339321	-0.166773	-2.312399
H	3.914396	-0.934309	-1.991575
H	2.444648	-1.927539	-2.113213
C	-5.564774	-0.499233	0.100921
H	4.048957	2.358848	0.002275
H	4.903750	0.840011	-0.335159
H	3.720405	1.481379	-1.502027
H	2.278675	0.067310	2.315672
H	4.036412	0.221618	2.069463
H	3.011853	1.673430	2.133927
H	-5.823700	-0.056898	-0.870458
H	-5.814559	0.229139	0.884114
H	-6.119840	-1.429175	0.255670

38
3a.Zs-trans.xyz

B	0.554504	-0.178942	-0.004704
C	-0.991745	-0.415905	-0.000865
O	1.416078	-1.251703	-0.124728
C	-1.370592	-1.871560	0.119667
C	-1.894116	0.602378	-0.109809
O	1.230696	1.015800	0.134583
C	2.754493	-0.714997	-0.304054
H	-2.086648	-2.183712	-0.651153
H	-0.483685	-2.509959	0.045323
H	-1.860835	-2.083745	1.080420
C	-1.530995	2.044442	-0.292908
C	-3.377487	0.441431	-0.105760
C	2.635023	0.694219	0.349206
C	3.744126	-1.643346	0.367315
C	3.006094	-0.653276	-1.802860
H	-1.983092	2.429626	-1.218213
H	-1.968507	2.656245	0.510108
H	-0.453952	2.212673	-0.316727
O	-3.820587	-0.790250	0.248377
O	-4.144513	1.346655	-0.372367
C	3.481887	1.769237	-0.297659
C	2.852253	0.670867	1.854181
H	3.763114	-2.612673	-0.148034
H	4.760083	-1.223496	0.328516
H	3.485584	-1.827459	1.416610

H	2.303019	0.029834	-2.298937
H	4.028655	-0.320839	-2.030202
H	2.867834	-1.651768	-2.237556
C	-5.243292	-0.909255	0.268477
H	3.336834	2.727604	0.218099
H	4.549767	1.513274	-0.233950
H	3.223555	1.914743	-1.353024
H	3.908395	0.506257	2.109834
H	2.254596	-0.116882	2.332989
H	2.544134	1.633613	2.282156
H	-5.685707	-0.207136	0.987390
H	-5.670104	-0.699542	-0.721058
H	-5.452855	-1.941372	0.564425

48
4a.E_s-cis.xyz

B	1.495521	-0.310937	-0.422131
C	0.126899	-0.666487	-1.107963
O	1.867695	-0.848479	0.791021
C	0.123789	-1.972570	-1.831540
C	-0.942411	0.158614	-1.015222
O	2.541417	0.247952	-1.121356
C	3.318158	-0.768772	0.852113
H	-0.837441	-2.243608	-2.287036
H	0.891467	-1.973176	-2.622591
H	0.413961	-2.783966	-1.143525
C	-2.313438	-0.064111	-1.592062
C	-0.710892	1.385954	-0.228274
C	3.647413	0.356584	-0.180242
C	3.726768	-0.457813	2.276265
C	3.841967	-2.132984	0.427248
H	-2.644111	0.845164	-2.114345
H	-2.267154	-0.854493	-2.355843
O	-1.789813	2.194904	-0.192125
O	0.337637	1.663504	0.333713
C	4.941306	0.155588	-0.941545
C	3.592402	1.750380	0.424470
H	3.455903	-1.291293	2.938096
H	4.814887	-0.311453	2.348156
H	3.229148	0.443684	2.651835
H	4.936465	-2.198123	0.507120
H	3.559030	-2.362196	-0.610297
H	3.406395	-2.906945	1.073319
C	-1.617146	3.383455	0.581123
H	5.097153	0.985009	-1.644229
H	5.799882	0.134147	-0.253865
H	4.937992	-0.775782	-1.520291
H	3.619545	2.494487	-0.382754
H	4.449583	1.935015	1.087860
H	2.664222	1.905998	0.987176
H	-0.817103	4.008315	0.163174
H	-1.360902	3.137997	1.619633
H	-2.574534	3.910248	0.539048

C	-3.345077	-0.435342	-0.551712
C	-4.523250	0.302797	-0.404151
C	-3.139881	-1.538343	0.286255
C	-5.479534	-0.057070	0.544634
H	-4.685538	1.177505	-1.039782
C	-4.092710	-1.902180	1.235056
H	-2.214392	-2.114725	0.197299
C	-5.268405	-1.162878	1.366874
H	-6.393257	0.532890	0.644673
H	-3.914970	-2.766693	1.878203
H	-6.015079	-1.445293	2.111637

48
4a.E_s-trans.xyz

B	1.378087	-0.334635	-0.365642
C	-0.033136	-0.619317	-0.980664
O	1.764748	-0.812109	0.865912
C	-0.097468	-2.002186	-1.556682
C	-1.067300	0.253551	-1.010282
O	2.425105	0.127947	-1.127575
C	3.218906	-0.881019	0.842781
H	-1.040704	-2.247173	-2.059742
H	0.722813	-2.158066	-2.276562
H	0.063265	-2.747606	-0.759834
C	-2.428976	-0.002761	-1.607981
C	-0.948514	1.601561	-0.413952
C	3.591307	0.163405	-0.256099
C	3.743933	-0.562872	2.226346
C	3.576302	-2.305738	0.446519
H	-2.739460	0.895533	-2.159178
H	-2.374872	-0.823413	-2.335273
O	0.209807	1.788326	0.278161
O	-1.787620	2.475057	-0.510365
C	4.813563	-0.187123	-1.077823
C	3.697647	1.577042	0.289732
H	3.435461	-1.341951	2.935855
H	4.843330	-0.524017	2.227603
H	3.363014	0.395378	2.598024
H	4.661550	-2.478310	0.468053
H	3.210667	-2.542135	-0.563025
H	3.105346	-3.007498	1.147544
C	0.327927	3.083580	0.868120
H	5.003890	0.595398	-1.824224
H	5.705235	-0.264301	-0.438230
H	4.688417	-1.135572	-1.613283
H	3.712892	2.291579	-0.544078
H	4.613698	1.717623	0.880812
H	2.835751	1.815835	0.924886
H	-0.560146	3.318034	1.467774
H	0.441891	3.856630	0.096097
H	1.217323	3.051816	1.504255
C	-3.468792	-0.322890	-0.559086
C	-4.392439	0.639272	-0.136976

C	-3.510634	-1.593291	0.029505
C	-5.339916	0.335760	0.840299
H	-4.354754	1.638177	-0.576505
C	-4.456159	-1.899714	1.005335
H	-2.790758	-2.354770	-0.284481
C	-5.376315	-0.934113	1.414280
H	-6.054863	1.098840	1.155791
H	-4.476147	-2.897860	1.448212
H	-6.119202	-1.171654	2.178434

48
4a.Z_s-cis.xyz

B	1.405919	0.818028	-0.004955
C	0.070314	1.604321	0.205414
O	1.616600	-0.284402	-0.806863
C	0.206517	2.822539	1.079407
C	-1.123785	1.193983	-0.315217
O	2.544183	1.189544	0.679850
C	3.049326	-0.551646	-0.812741
H	-0.378654	2.725751	2.004641
H	1.254026	2.995838	1.349159
H	-0.189970	3.723179	0.592410
C	-1.294119	-0.030858	-1.183392
C	-2.375108	1.935215	0.002381
C	3.514899	0.119543	0.515230
C	3.253762	-2.050191	-0.874780
C	3.616322	0.124728	-2.050481
H	-1.964205	0.211162	-2.018521
H	-0.327093	-0.311973	-1.611780
O	-3.445831	1.383350	-0.633207
O	-2.495019	2.903860	0.726350
C	4.901929	0.723903	0.468644
C	3.366212	-0.792063	1.723796
H	2.904016	-2.440962	-1.839303
H	4.319898	-2.302666	-0.777562
H	2.699912	-2.570804	-0.084381
H	3.478173	1.213881	-2.011373
H	4.688942	-0.082666	-2.169738
H	3.097017	-0.249044	-2.942453
C	-4.689620	2.012683	-0.336544
H	5.149857	1.175897	1.437945
H	5.655741	-0.048453	0.256540
H	4.983020	1.505993	-0.295082
H	2.364678	-1.242662	1.766001
H	4.108190	-1.602625	1.715901
H	3.509068	-0.207330	2.641699
H	-4.688612	3.061365	-0.662914
H	-4.893770	1.989060	0.741986
H	-5.450890	1.445530	-0.880570
C	-1.860844	-1.213033	-0.429661
C	-3.111606	-1.751662	-0.748467
C	-1.129578	-1.802009	0.609640
C	-3.615302	-2.852849	-0.056497

H	-3.697319	-1.293479	-1.549420
C	-1.629071	-2.900442	1.305328
H	-0.151076	-1.389183	0.869990
C	-2.876039	-3.431641	0.973933
H	-4.593755	-3.259180	-0.322500
H	-1.041560	-3.346240	2.111162
H	-3.269945	-4.292472	1.517948

48
4a.Z_s-trans.xyz

B	-1.422726	0.702352	-0.036011
C	-0.077474	1.492661	0.050635
O	-2.595061	1.276075	0.409229
C	-0.191673	2.800070	0.793540
C	1.082403	1.035777	-0.497598
O	-1.611723	-0.589707	-0.484553
C	-3.683408	0.385558	0.039015
H	0.411434	3.600397	0.349474
H	-1.236493	3.129381	0.833026
H	0.155996	2.698260	1.832707
C	1.214548	-0.231519	-1.307860
C	2.395909	1.741043	-0.409817
C	-2.958135	-0.988067	-0.093938
C	-4.743116	0.441015	1.118625
C	-4.235335	0.893864	-1.283808
H	1.766970	0.015746	-2.225219
H	0.227517	-0.605004	-1.594679
O	2.526288	2.530592	0.680945
O	3.294113	1.596126	-1.216506
C	-3.519193	-1.904903	-1.159407
C	-2.827085	-1.721767	1.231913
H	-5.202114	1.437973	1.147220
H	-5.540980	-0.289303	0.918924
H	-4.326918	0.236332	2.111913
H	-3.478891	0.844816	-2.078994
H	-5.113180	0.316705	-1.606316
H	-4.539570	1.942796	-1.173366
C	3.799911	3.169953	0.787355
H	-2.947409	-2.841608	-1.191226
H	-4.566797	-2.158986	-0.940891
H	-3.473888	-1.451657	-2.156411
H	-3.795467	-2.106761	1.580913
H	-2.413789	-1.068265	2.013025
H	-2.147834	-2.576292	1.111975
H	4.605042	2.426123	0.847216
H	3.990445	3.814810	-0.080418
H	3.760030	3.764349	1.704613
C	1.970146	-1.303332	-0.556487
C	3.292849	-1.624669	-0.879068
C	1.355279	-1.981961	0.504100
C	3.980421	-2.609447	-0.169402
H	3.786343	-1.086396	-1.691574
C	2.039631	-2.965728	1.213958

H	0.323642	-1.730425	0.763025
C	3.356313	-3.284419	0.878441
H	5.011898	-2.849325	-0.437075
H	1.542900	-3.488989	2.034411
H	3.893866	-4.055910	1.433683

45
5a.E_s-cis.xyz

B	-1.187444	-0.423273	0.083282
C	0.312624	-0.886363	0.162508
O	-1.923694	-0.501731	-1.079981
C	0.502899	-2.342778	0.428269
C	1.332415	-0.001132	0.018591
O	-1.958870	-0.272389	1.214054
C	-3.321809	-0.542520	-0.683114
H	1.539466	-2.625388	0.652262
H	-0.138235	-2.655602	1.267947
H	0.164379	-2.939138	-0.435711
C	0.896484	1.398584	-0.175500
C	-3.294020	0.076908	0.751135
C	-4.134519	0.240103	-1.692719
C	-3.728532	-2.008838	-0.685542
O	1.912641	2.266008	-0.337565
O	-0.274253	1.749532	-0.198063
C	-4.301511	-0.516431	1.714061
C	-3.389388	1.594390	0.744582
H	-4.100094	-0.255759	-2.672032
H	-5.188803	0.300978	-1.383869
H	-3.749987	1.258703	-1.819338
H	-4.796638	-2.140568	-0.461141
H	-3.149239	-2.583733	0.051194
H	-3.532551	-2.439182	-1.676687
C	1.509323	3.622849	-0.534539
H	-4.216663	-0.029914	2.694915
H	-5.328170	-0.360031	1.350678
H	-4.146241	-1.591972	1.860564
H	-3.149034	1.974100	1.746644
H	-4.402851	1.933275	0.485730
H	-2.673710	2.037311	0.041782
H	0.929198	3.986867	0.323313
H	0.891964	3.718127	-1.437006
H	2.434029	4.196260	-0.642367
C	2.772736	-0.346169	0.037767
C	3.665289	0.283110	0.918971
C	3.276243	-1.333712	-0.821910
C	5.010628	-0.072466	0.947403
H	3.293917	1.057184	1.593076
C	4.624281	-1.686266	-0.799454
H	2.597072	-1.819370	-1.527076
C	5.496660	-1.058409	0.087952
H	5.686170	0.424427	1.646819
H	4.994833	-2.453311	-1.482618
H	6.553003	-1.333423	0.108191

45
5a.E_s-trans.xyz

B	-1.077971	-0.416121	0.073712
C	0.452517	-0.742549	0.136355
O	-1.821462	-0.414417	-1.083991
C	0.696423	-2.194758	0.421423
C	1.457171	0.162286	-0.000869
O	-1.843202	-0.415637	1.217001
C	-3.207281	-0.620750	-0.692354
H	1.731398	-2.423076	0.704691
H	0.027300	-2.533739	1.228067
H	0.441999	-2.814580	-0.455024
C	1.158832	1.603138	-0.182676
C	-3.217909	-0.174507	0.807147
C	-4.092198	0.203554	-1.603581
C	-3.488031	-2.105170	-0.870644
O	-0.180569	1.852083	-0.250321
O	1.980043	2.490778	-0.273831
C	-4.133361	-0.985626	1.700859
C	-3.480461	1.310719	0.990144
H	-4.025120	-0.169579	-2.634014
H	-5.144982	0.139782	-1.290925
H	-3.796746	1.259611	-1.612195
H	-4.537747	-2.353949	-0.660613
H	-2.852259	-2.713691	-0.211909
H	-3.269477	-2.394042	-1.907084
C	-0.511953	3.213453	-0.518768
H	-4.077728	-0.614760	2.732803
H	-5.178900	-0.899574	1.369787
H	-3.860826	-2.047342	1.713097
H	-3.275535	1.590518	2.031857
H	-4.523536	1.572933	0.763852
H	-2.823479	1.910258	0.348473
H	0.079568	3.599798	-1.357694
H	-0.323619	3.843558	0.361244
H	-1.577596	3.225076	-0.769709
C	2.898944	-0.185065	0.019709
C	3.799484	0.483721	0.862448
C	3.397563	-1.193098	-0.818379
C	5.147322	0.136503	0.882390
H	3.434889	1.282168	1.509695
C	4.748081	-1.535306	-0.806096
H	2.713446	-1.702529	-1.501648
C	5.627987	-0.873818	0.049023
H	5.830091	0.663588	1.551963
H	5.114593	-2.318842	-1.472517
H	6.686849	-1.139804	0.061583

45
5a.Z_s-cis.xyz

B	0.883958	-0.921855	-0.009917
C	-0.570436	-1.492970	-0.075578

O	1.291086	0.042292	0.879285
C	-0.677962	-2.988829	-0.133819
C	-1.638200	-0.645398	-0.039002
O	1.907958	-1.421473	-0.781956
C	2.744283	0.082028	0.827976
H	-1.398806	-3.327893	-0.888716
H	0.299973	-3.441207	-0.343257
H	-1.049774	-3.399589	0.816726
C	-3.024840	-1.165869	0.031413
C	3.053068	-0.543582	-0.569829
C	3.184803	1.521917	0.990494
C	3.245000	-0.770201	1.983570
O	-3.938494	-0.164587	-0.065721
O	-3.352336	-2.329674	0.160841
C	4.315888	-1.376131	-0.629042
C	3.030913	0.479846	-1.694129
H	2.927307	1.881636	1.995412
H	4.274174	1.615509	0.868402
H	2.692738	2.182092	0.266243
H	2.934029	-1.818279	1.872758
H	4.340595	-0.742405	2.066427
H	2.822118	-0.392803	2.923657
C	-5.293564	-0.602689	0.008202
H	4.454426	-1.780864	-1.640260
H	5.197149	-0.762902	-0.389714
H	4.283574	-2.221620	0.067954
H	2.125622	1.101307	-1.648743
H	3.906239	1.143336	-1.656901
H	3.037776	-0.040500	-2.661006
H	-5.489852	-1.119795	0.957008
H	-5.528393	-1.293515	-0.812519
H	-5.905566	0.300589	-0.068285
C	-1.422669	0.822060	-0.115410
C	-1.878896	1.697210	0.879751
C	-0.686679	1.355154	-1.182576
C	-1.574739	3.053713	0.826980
H	-2.460895	1.301320	1.714173
C	-0.390055	2.715601	-1.244320
H	-0.348564	0.683030	-1.977314
C	-0.826648	3.569397	-0.233336
H	-1.921924	3.716743	1.622261
H	0.181605	3.109537	-2.088190
H	-0.594656	4.635542	-0.274191

45
5a.Z_s-trans.xyz

B	0.723036	-0.894495	-0.031749
C	-0.795203	-1.263802	-0.122002
O	1.673565	-1.498164	-0.823918
C	-1.070528	-2.739878	-0.203727
C	-1.735708	-0.276321	-0.075414
O	1.254559	-0.045425	0.907497
C	2.933271	-0.813068	-0.559195

H	-1.729045	-2.992132	-1.045130
H	-0.135770	-3.303339	-0.317377
H	-1.585632	-3.109038	0.694708
C	-3.204935	-0.473089	0.026406
C	2.698853	-0.211206	0.864203
C	4.057135	-1.823899	-0.644009
C	3.087495	0.250293	-1.634862
O	-3.584469	-1.775050	-0.036632
O	-4.010804	0.425358	0.166565
C	3.337897	1.142681	1.093018
C	3.059751	-1.175262	1.983588
H	4.149329	-2.200608	-1.671235
H	5.016914	-1.362136	-0.369124
H	3.886883	-2.684143	0.013805
H	2.288008	1.001215	-1.571154
H	4.053132	0.769202	-1.556931
H	3.032590	-0.221740	-2.624779
C	-4.995409	-1.971447	0.066761
H	3.125700	1.490223	2.112620
H	4.430460	1.085959	0.977060
H	2.949547	1.897331	0.398628
H	4.146987	-1.309483	2.072502
H	2.602405	-2.162004	1.825911
H	2.685512	-0.781179	2.937347
H	-5.374889	-1.597288	1.026601
H	-5.525219	-1.447176	-0.739225
H	-5.153312	-3.051098	-0.011271
C	-1.309159	1.144679	-0.139639
C	-1.698394	2.090281	0.819148
C	-0.457391	1.557243	-1.174027
C	-1.212154	3.393026	0.766127
H	-2.373588	1.792669	1.621653
C	0.022331	2.864060	-1.234790
H	-0.178544	0.834313	-1.947125
C	-0.348290	3.786018	-0.257642
H	-1.511127	4.111310	1.532384
H	0.680406	3.164281	-2.053878
H	0.023892	4.811718	-0.299010

4.3. Photoisomerization of alkenyl boronates, multireference analysis

38

3a.E_s-cis.xyz

B	-0.319288	0.618969	-0.039126
C	0.992172	1.502148	-0.080827
O	-0.995633	0.331247	1.107293
C	0.691080	2.984691	-0.167556
C	2.238750	0.969963	-0.041520
O	-1.031214	0.295598	-1.153588
C	-2.316949	-0.092972	0.762148
H	1.574425	3.617737	-0.193953
H	0.103044	3.189229	-1.065697
H	0.084778	3.290065	0.689104
C	3.548537	1.721960	-0.069858

C	2.332363	-0.519246	0.038250
C	-2.165510	-0.481177	-0.755367
C	-2.715901	-1.234256	1.688867
C	-3.234785	1.108183	0.998842
H	4.133495	1.505604	0.824907
H	4.150290	1.411705	-0.925068
H	3.401997	2.795985	-0.128976
O	3.589459	-0.966557	0.087450
O	1.388389	-1.252793	0.056135
C	-3.342905	-0.099472	-1.644417
C	-1.807466	-1.950691	-0.982384
H	-2.765588	-0.865335	2.714152
H	-3.698470	-1.630172	1.422418
H	-1.991155	-2.044423	1.660995
H	-3.131709	1.426050	2.037008
H	-4.282299	0.863589	0.816087
H	-2.960401	1.950876	0.363297
C	3.768972	-2.365081	0.164119
H	-3.123662	-0.386363	-2.673522
H	-4.251994	-0.619459	-1.334212
H	-3.531096	0.971863	-1.631211
H	-1.540827	-2.083284	-2.031747
H	-2.649627	-2.607406	-0.756997
H	-0.951147	-2.250161	-0.382460
H	3.304509	-2.767220	1.063435
H	3.341322	-2.859635	-0.706968
H	4.842402	-2.528351	0.194928

38
3a.T1min.xyz

B	0.542183	0.642791	-0.041720
C	-0.804742	1.397123	-0.090692
O	0.729727	-0.534568	0.625987
C	-0.967255	2.706246	-0.817854
C	-2.012347	0.791593	0.520508
O	1.687172	1.072442	-0.649215
C	2.010176	-1.054079	0.254860
H	-0.008664	3.091789	-1.162847
H	-1.434525	3.460408	-0.176061
H	-1.618875	2.576650	-1.687780
C	-2.304788	1.010524	1.977009
C	-2.784133	-0.138464	-0.296899
C	2.764839	0.241849	-0.207139
C	2.613862	-1.769051	1.456163
C	1.770155	-2.053020	-0.878721
H	-2.106266	2.048341	2.253401
H	-1.652311	0.379631	2.589865
H	-3.336584	0.770371	2.221269
O	-3.835109	-0.689367	0.332681
O	-2.512203	-0.388722	-1.442617
C	3.739920	0.053817	-1.361493
C	3.453893	0.984021	0.939630
H	2.003798	-2.638633	1.703477

H	3.625588	-2.116650	1.235683
H	2.648116	-1.126301	2.333098
H	1.069924	-2.813119	-0.531016
H	2.692839	-2.548352	-1.185168
H	1.328781	-1.568194	-1.749846
C	-4.619799	-1.592736	-0.412620
H	4.192298	1.013818	-1.613223
H	4.540517	-0.636967	-1.087748
H	3.241282	-0.322627	-2.251744
H	2.775776	1.134624	1.780391
H	4.335755	0.450232	1.297204
H	3.767165	1.965485	0.582089
H	-4.026829	-2.443809	-0.745922
H	-5.057094	-1.105900	-1.283893
H	-5.406993	-1.929962	0.256166

38
3a.Z_s-cis.xyz

B	0.620901	0.270049	-0.029934
C	-0.906812	0.670437	-0.089815
O	1.172990	-0.976903	-0.093293
C	-1.148344	2.169186	-0.218364
C	-1.926154	-0.244107	-0.023588
O	1.589008	1.226553	0.072522
C	2.589054	-0.846307	-0.263919
H	-1.926924	2.515392	0.458609
H	-0.232316	2.713565	-0.004060
H	-1.471497	2.430473	-1.226586
C	-1.734921	-1.736691	0.143039
C	-3.357518	0.188615	-0.108292
C	2.848184	0.590263	0.303003
C	3.277288	-1.979614	0.484733
C	2.864455	-0.970782	-1.763329
H	-2.157382	-2.061503	1.094651
H	-2.276817	-2.274900	-0.636508
H	-0.692064	-2.018163	0.101703
O	-4.191360	-0.774315	0.317669
O	-3.756122	1.235923	-0.507216
C	3.935877	1.387467	-0.404215
C	3.086359	0.614476	1.814084
H	3.016148	-2.932211	0.022266
H	4.363058	-1.869652	0.442563
H	2.970194	-2.020095	1.527328
H	2.462695	-1.921745	-2.114460
H	3.933003	-0.946771	-1.981964
H	2.377618	-0.173654	-2.325972
C	-5.571307	-0.490253	0.265920
H	4.017699	2.373519	0.054218
H	4.904103	0.890206	-0.313975
H	3.711520	1.528213	-1.459129
H	2.317959	0.054457	2.348174
H	4.061501	0.201962	2.077024
H	3.046148	1.648520	2.157564

H	-5.816575	0.372111	0.884577
H	-5.891509	-0.291144	-0.756105
H	-6.074161	-1.375016	0.646762

45
5a.E_s-cis.xyz

B	-1.231043	-0.468124	0.102979
C	0.291660	-0.889048	0.189869
O	-1.937423	-0.438563	-1.059900
C	0.503085	-2.369376	0.427761
C	1.323463	-0.002367	0.056139
O	-2.029306	-0.362962	1.199236
C	-3.332993	-0.449542	-0.742507
H	1.526380	-2.610623	0.705520
H	-0.163486	-2.709101	1.223733
H	0.247623	-2.938393	-0.470220
C	0.936508	1.438200	-0.116188
C	-3.343691	0.004152	0.764358
C	-4.055234	0.482052	-1.707225
C	-3.814791	-1.886120	-0.953837
O	1.957946	2.249811	-0.322139
O	-0.199161	1.818807	-0.072206
C	-4.357454	-0.709082	1.650541
C	-3.472794	1.516564	0.951275
H	-3.965189	0.091056	-2.721470
H	-5.117628	0.551024	-1.463055
H	-3.626433	1.481252	-1.695657
H	-4.889078	-1.983817	-0.790389
H	-3.299278	-2.581467	-0.290496
H	-3.594881	-2.179848	-1.980820
C	1.669445	3.624079	-0.498966
H	-4.261869	-0.341975	2.672990
H	-5.377629	-0.512031	1.313854
H	-4.196360	-1.784743	1.667258
H	-3.283620	1.752754	1.999194
H	-4.474355	1.867226	0.696208
H	-2.742822	2.056680	0.352547
H	1.174499	4.030407	0.381400
H	1.031975	3.774012	-1.368458
H	2.628280	4.110298	-0.648676
C	2.775653	-0.370002	0.044260
C	3.647802	0.093060	1.049446
C	3.294190	-1.189854	-0.963559
C	4.972089	-0.265422	1.057650
H	3.265168	0.736630	1.831032
C	4.650430	-1.542414	-0.967017
H	2.645264	-1.550557	-1.750811
C	5.494699	-1.087573	0.045026
H	5.620056	0.092660	1.847301
H	5.038580	-2.170692	-1.758056
H	6.541169	-1.360728	0.050089

45

5a.T1min.xyz

B	1.188283	-0.102280	0.575766
C	-0.166835	0.021457	1.307668
O	1.337936	-0.014024	-0.777845
C	-0.265332	-0.007067	2.810720
C	-1.392307	0.402516	0.545133
O	2.378873	-0.319631	1.208674
C	2.736730	0.074477	-1.062958
H	0.658625	-0.367915	3.261377
H	-1.090950	-0.642752	3.144140
H	-0.458177	1.000266	3.194067
C	-1.477044	1.864397	0.319261
C	3.370579	-0.584804	0.212227
C	3.013232	-0.644190	-2.376661
C	3.065496	1.562030	-1.203827
O	-2.537708	2.314255	-0.351736
O	-0.626141	2.610764	0.724158
C	4.692967	0.015491	0.669996
C	3.497943	-2.105895	0.108755
H	2.510808	-0.117724	-3.188925
H	4.083503	-0.661564	-2.594427
H	2.641510	-1.666336	-2.363556
H	2.861625	2.103910	-0.280046
H	4.108488	1.722703	-1.481444
H	2.432079	1.985987	-1.983612
C	-2.604686	3.709417	-0.576038
H	5.032547	-0.499873	1.569328
H	5.461099	-0.100475	-0.097878
H	4.593150	1.071734	0.909371
H	2.561842	-2.561951	-0.215536
H	4.285681	-2.401308	-0.585994
H	3.741441	-2.503974	1.094476
H	-1.747953	4.050064	-1.154458
H	-2.636142	4.252678	0.366659
H	-3.521827	3.876239	-1.133782
C	-2.326272	-0.606109	0.108242
C	-2.029324	-1.979617	0.394535
C	-3.535973	-0.354270	-0.586959
C	-2.859575	-2.990697	0.021341
H	-1.117657	-2.221710	0.919500
C	-4.381969	-1.402202	-0.964258
H	-3.813318	0.653967	-0.829529
C	-4.061253	-2.720898	-0.669739
H	-2.594901	-4.013438	0.256476
H	-5.297878	-1.172977	-1.493057
H	-4.716940	-3.529274	-0.962623

45

5a.Z_s-cis.xyz

B	-0.946795	-0.882402	0.036226
C	0.502924	-1.502291	0.040195
O	-1.671845	-0.655156	-1.091809
C	0.533352	-3.022044	0.079139

C	1.595782	-0.697605	0.022562
O	-1.652035	-0.611449	1.165841
C	-3.027101	-0.386300	-0.711002
H	0.926766	-3.380351	1.030123
H	-0.473761	-3.425368	-0.045284
H	1.166590	-3.434958	-0.703230
C	2.984948	-1.256227	0.047094
C	-2.869689	0.045429	0.791375
C	-3.588549	0.680584	-1.641346
C	-3.800231	-1.693195	-0.894427
O	3.901742	-0.319823	-0.143597
O	3.265826	-2.400352	0.215956
C	-3.978396	-0.423219	1.724486
C	-2.635914	1.545199	0.976528
H	-3.632621	0.286710	-2.657426
H	-4.600163	0.964724	-1.343451
H	-2.964023	1.570758	-1.655188
H	-3.411589	-2.481586	-0.248995
H	-4.863504	-1.567957	-0.684721
H	-3.689475	-2.022535	-1.928028
C	5.254515	-0.735494	-0.138105
H	-3.758364	-0.092400	2.739998
H	-4.940685	0.001536	1.430446
H	-4.064239	-1.507402	1.739266
H	-1.833388	1.908150	0.335361
H	-3.538750	2.121863	0.769008
H	-2.341869	1.726352	2.010681
H	5.438705	-1.456102	-0.932509
H	5.514573	-1.187225	0.817168
H	5.840542	0.163889	-0.301969
C	1.453281	0.798161	-0.034559
C	1.117921	1.439729	-1.235450
C	1.643937	1.580245	1.117938
C	0.973352	2.830906	-1.285539
H	0.964745	0.851142	-2.129471
C	1.498243	2.967995	1.070594
H	1.903453	1.099501	2.051787
C	1.163071	3.599258	-0.133024
H	0.716122	3.310493	-2.220936
H	1.644434	3.555287	1.967651
H	1.052294	4.674948	-0.171297

7 Photophysical Studies

Ultraviolet–visible spectra (UV–Vis). Sample solutions in acetonitrile were obtained by a Perkin Elmer Lambda 1050 UV/Vis/NIR spectrometer.

Steady-state luminescence. Experiments for acetonitrile solutions were carried out with a luminescence spectrometer Perkin Elmer LS 55, with an excitation wavelength of 400 nm. The triplet excited state energy (E_T) was calculated using the following equation:

$$E_T = N_A \frac{hc}{\lambda} \quad [J \cdot mol^{-1}]$$

where N_A is the Avogrado number, *h* the Planck's constant, *c* is the velocity of light and λ is the wavelength of the intersection of the normalized emission spectra and excitation expressed in meters. Then, the conversion between Jules to calories can be applied to obtain the value in terms of Kcal · mol^{-1}.

Luminescence quantum yield (ϕ_L) for **PC-7** was determined using the following standards: fluorescein[19] or riboflavin[20] using the following equation:

$$\phi_{Li} = \phi_s \frac{I_i}{I_s} \frac{n_i^2}{n_s^2} \frac{1 - 10^{Abs_s}}{1 - 10^{Abs_i}}$$

where the subscripts "i" and "s" refer to the sample of interest and the standard, respectively, *n* represents the refractive index of the corresponding solvents, *I* represent the integrals of the corresponding emission spectra and *Abs* represents the absorbance at the λexcitation.

Transient Absorption Spectroscopy (TAS). LP980 equipment from Edinburgh Instruments coupled with an optical parametric oscillator (OPO) pumped by the third harmonic of a Nd:YAG laser (EKSPLA). The single pulses were ca. 5 ns duration, and the energy was adjusted to 1 mJ by pulse at excitation wavelength (355 nm). A pulsed xenon flash lamp (150 W) was employed as detecting light source. A monochromator (TMS302-A, grating 150 lines/mm) disperses the probe light after it has passed the sample. The probe light is then passed on to a PMT detector (Hamamatsu Photonics) to obtain the transient signals.

The change in optical density in transitory measurements is defined as ΔOD. ΔOD correspond to the change in triplet absorbance as a function of time by monitoring the probe beam (xenon flash lamp) at the triplet-triplet transition wavelength. An experimental measurement involves the simultaneous combination of pump and probe beams. The "probe-only" spectrum corresponds to the ground state absorption, whereas the "pump + probe" spectrum contains contributions from both the ground and excited states. Transient data is presented as the change in absorption ΔOD, which is effectively the difference between the "pump + probe" and the "probe-only" spectra.

The absorbance of the solutions for **PC-7** in acetonitrile was kept at ~0.3 at λexc = 355 nm and the samples were recorded at room temperature using 1 × 1 cm^2 quartz cells with 3 mL capacity and were bubbled for 15 min with N_2 or O_2 before acquisition. All transient lifetimes were fitted both as mono-exponential functions for the decay traces registered at 450 or 500 nm.

Quenching Experiments. In a typical quenching experiment, the appropriate volumes of a freshly prepared of ***E***- or ***Z***-alkene **3a** in acetonitrile solution were added to the purged **PC-7** solution. All the photophysical measurements were performed at room temperature in a quartz cell of 1.0 cm optical path length.

Additional photophysical studies.

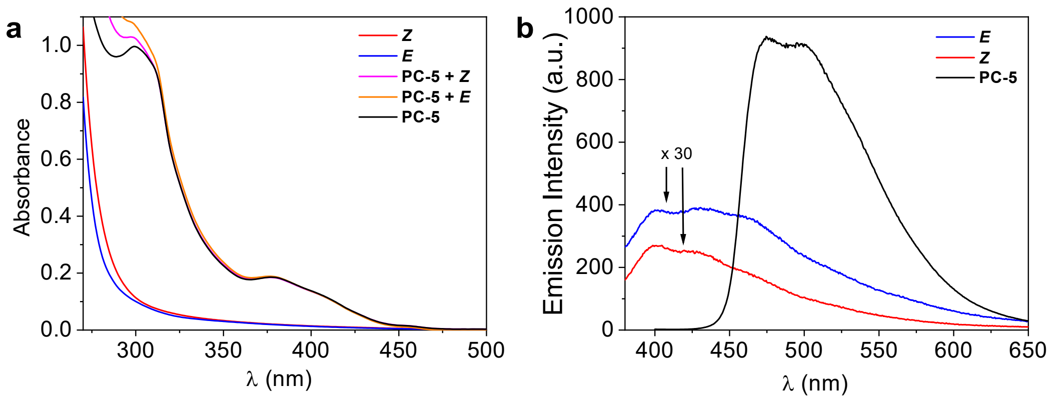

Figure S16. a) Absorption spectra for **PC-7** (black, 30 μM) and in the presence of ***Z*-3a** (pink) and ***E*-3a** (orange) alkenes (10 mM). Naked ***Z*-3a** (red) and ***E*-3a** (blue) alkenes at the same concentration are included for comparison. b) Emission spectra for **PC-7** (black, 30 μM), ***Z*-3a** (red) and ***E*-3a** (blue) alkenes (10 mM). All samples were measured in aerated acetonitrile.

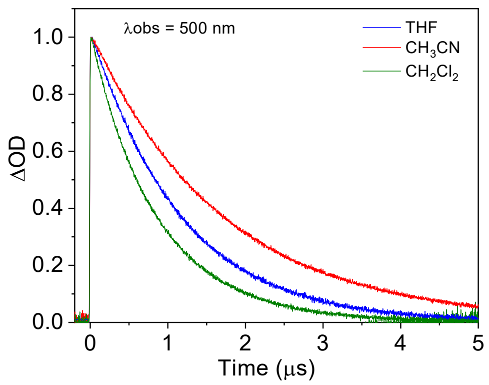

Figure S17. Normalized time-resolved luminescence traces (λexc = 355 nm, λobs = 500 nm) for **PC-7** in deaerated acetonitrile (red), tetrahydrofuran (blue) and methylene chloride (green). The measurements were performed by excitation with a laser flash photolysis equipment in the kinetic emission mode.

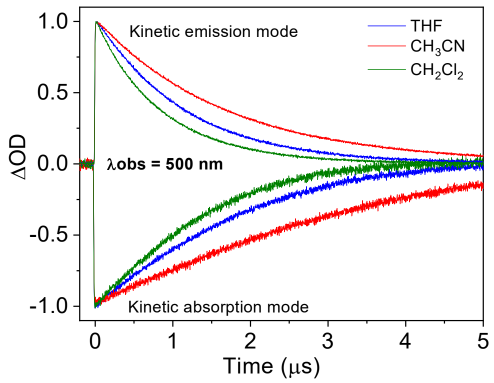

Figure S18. Comparative between the time-resolved luminescence traces (λexc = 355 nm, λobs = 500 nm) obtained from the kinetic emission (positive signals) and kinetic absorption (negative signals) software mode for

PC-7 in deaerated acetonitrile (red), tetrahydrofuran (blue) and methylene chloride (green). The measurements were performed by excitation with a laser flash photolysis equipment in the kinetic emission or absorption mode.

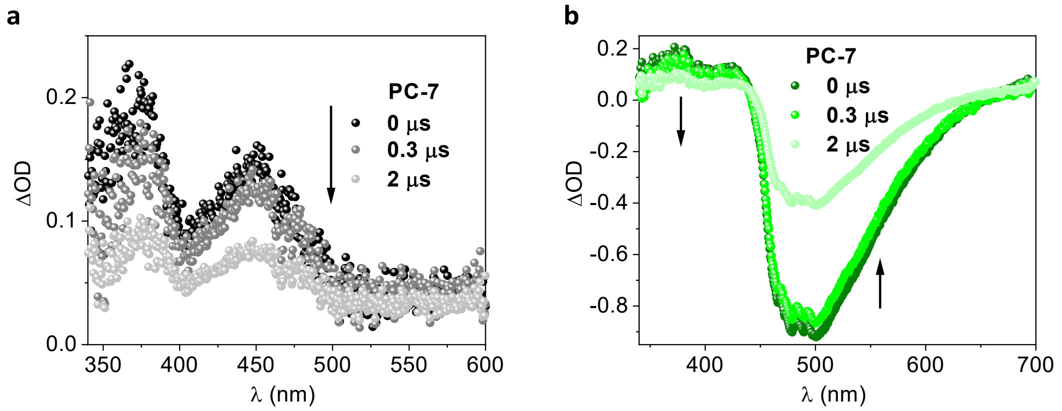

Figure S19. a and b) Transient absorption spectra (TAS, λ_{exc} = 355 nm, N_2) for **PC-7** (50 μM) at different timescales after laser pulse with (a) or without (b) luminescence subtraction.

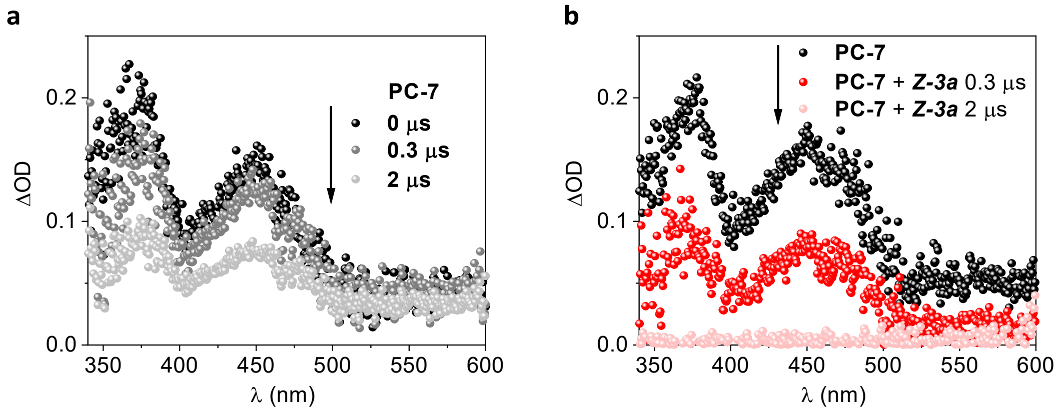

Figure S20. a and b) TAS (λ_{exc} = 355 nm, N_2) for **PC-7** at different timescales after laser pulse in absence (a) or presence (b) of ***Z-3a*** (10 mM).

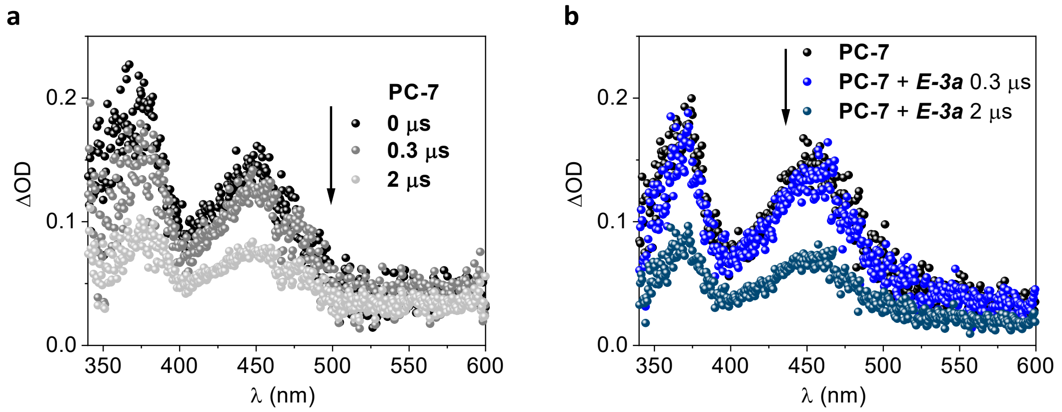

Figure S21. a and b) TAS (λ_{exc} = 355 nm, N_2) for **PC-7** at different timescales after laser pulse in absence (a) or presence (b) of ***E-3a*** (10 mM).

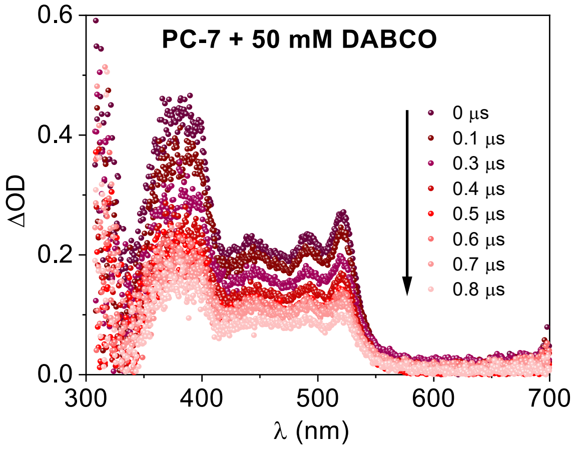

Figure S22. TAS (λ_{exc} = 355 nm, N_2) for **PC-7** at different timescales after laser pulse in presence of DABCO (50 mM) as electron donor to simulate the electron transfer process.

8 References

[1] Armarego, W.L.F.; Chai, C.L.L., in Purification of Laboratory Chemicals Elsevier, 2009, sixth Ed.; pp 461.

[2] Braunschweig, H.; Guethlein, F. Transition-Metal-Catalyzed Synthesis of Diboranes(4). *Angew. Chem. Int. Ed.* **2011**, *50*, 12613–12616.

[3] Wrackmeyer, B. Carbon-13 NMR spectroscopy of boron compounds. *Prog. NMR Spec.* **1979**, *12*, 227–259.

[4] Mackay, E. G.; Nörret, M.; Wong, L. S.-M.; Louis, I.; Lawrence, A. L.; Willis, A. C.; Sherburn, M. A Domino Diels–Alder Approach toward the Tetracyclic Nicandrenone Framework. *Org. Lett.* **2015**, *17*, 5517–5519.

[5] Neises, B.; Steglich, W. Simple Method for the Esterification of Carboxylic Acids. *Angew. Chem. Int. Ed.*, **1978**, *17*, 522-524.

[6] Leggio, A.; Belsito, E. L.; De Luca, G.; Di Gioia, M. L.; Leotta, V.; Romio, E.; Siciliano, C.; Liguori, A. One-pot synthesis of amides from carboxylic acids activated using thionyl chloride. *RSC Adv.* **2016**, *6*, 34468-34475.

[7] Martínez-Sarti, L.; Díez González, S. On the Unique Reactivity of $Pd(OAc)_2$ with Organic Azides: Expedient Synthesis of Nitriles and Imines. *ChemCatChem* **2013**, *5*, 1722-1724.

[8] Kim-Lee, S.-H.; Mauleón, P.; Gómez Arrayás, R.; Carretero, J. C. Dynamic Multiligand Catalysis: A Polar to Radical Cross-over Strategy Expands Alkyne Carboboration to Unactivated Secondary Alkyl Halides. *Chem* **2021**, *7*, 2212-2226.

[9] a) Alfaro, R.; Parra, A.; Alemán, J.; Ruano, J. L.; Tortosa, M. Copper(I)-Catalyzed Formal Carboboration of Alkynes: Synthesis of Tri- and Tetrasubstituted Vinylboronates. *J. Am. Chem. Soc.* **2012**, *134*, 15165-15168. b) Yoshida, H.; Kageyuki, I.; Takaki, K. Copper-Catalyzed Three-Component Carboboration of Alkynes and Alkenes. *Org. Lett.* **2013**, *15*, 952–955.

[10] Frisch, M. J.; et al., Gaussian 16, Revision C.01, Gaussian, Inc., Wallingford CT, USA, 2019.

[11] Zhao, Y.; Truhlar, D. G. The M06 suite of density functionals for main group thermochemistry, thermochemical kinetics, noncovalent interactions, excited states, and transition elements: two new functionals and systematic testing of four M06-class functionals and 12 other functionals *Theor. Chem. Acc.* **2008**, *120*, 215-241.

[12] Dunning, T. H. Gaussian basis sets for use in correlated molecular calculations. I. The atoms boron through neon and hydrogen. *J. Chem. Phys.* **1989**, *90*, 1007-1023.

[13] Weigend, F.; Ahlrichs, R. Balanced basis sets of split valence, triple zeta valence and quadruple zeta valence quality for H to Rn: Design and assessment of accuracy, *Phys. Chem. Chem. Phys.* **2005**, *7*, 3297-3305.

[14] Marenich, A. V.; Cramer, C. J.; Truhlar, D. G. Universal Solvation Model Based on Solute Electron Density and on a Continuum Model of the Solvent Defined by the Bulk Dielectric Constant and Atomic Surface Tensions. *J. Phys. Chem. B* **2009**, *113*, 6378-6396.

[15] Ayers, P. W.; Morrison, R. C.; Roy, R. K. Variational principles for describing chemical reactions: Condensed reactivity indices *J. Chem. Phys.* **2002**, *116*, 8731-8744.

[16] Fdez-Galván, I. *et al.*, OpenMolcas: From Source Code to Insight. *J. Chem. Theory Comput.* **2019**, *15*, 5925-5964.

[17] Roos, B. O.; Taylor, P. R.; Siegbahn, P. E. M. A complete active space SCF method (CASSCF) using a density matrix formulated super-CI approach. *Chem. Phys.* **1980**, *48*, 157-173.

[18] Finley, J.; Malmqvist, P.-Å.; Roos, B. O.; Serrano-Andrés, L. The multi-state CASPT2 method. *Chem. Phys. Lett.* **1998**, *288*, 299–306.

[19] Lakowicz, J. R. Principles of Fluorescence Spectroscopy, 2nd Ed., Kluwer Academic/Plenum Publishers, New York, London, Moscow, Dordrecht, 1999.

[20] Martinez-Haya, R.; Miranda, M. A.; Marin, M. L. Metal-Free Photocatalytic Reductive Dehalogenation Using Visible-Light: A Time-Resolved Mechanistic Study. *Eur. J. Org. Chem.* **2017**, 2164–2169.

9 NMR spectra

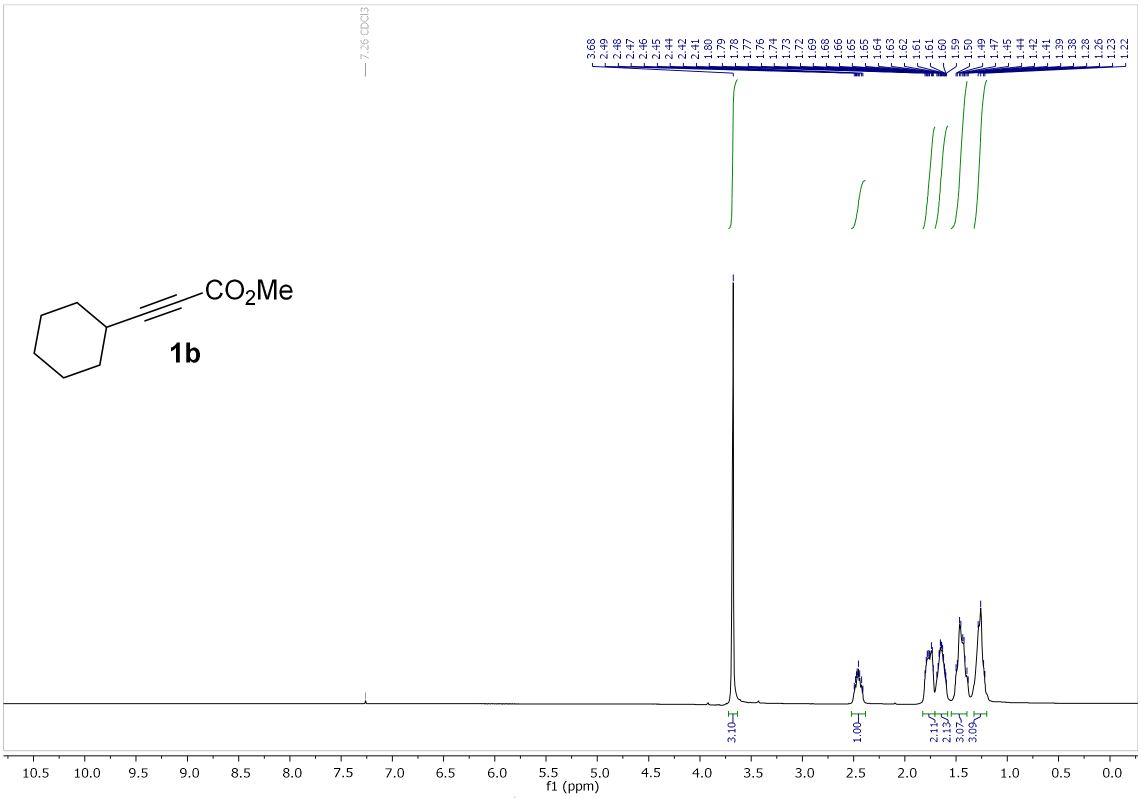

Figure S22. ^{1}H NMR spectrum of **1b.**

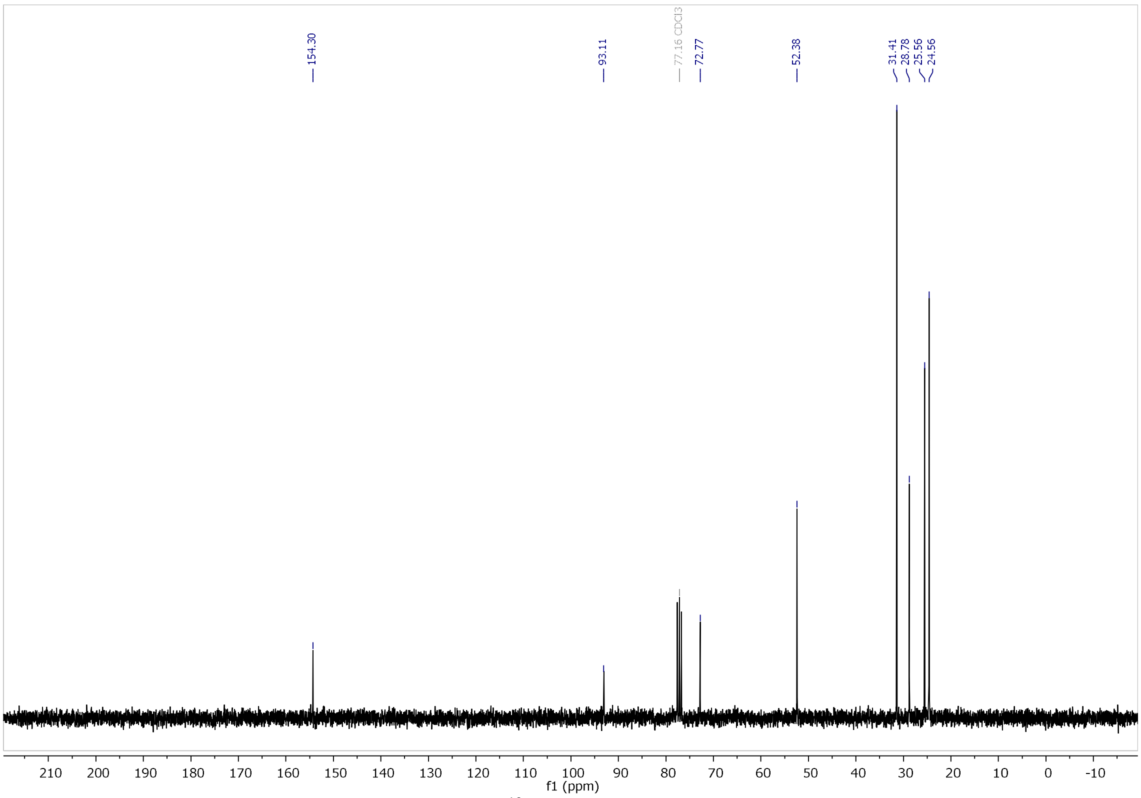

Figure S23. ^{13}C NMR spectrum of **1b.**

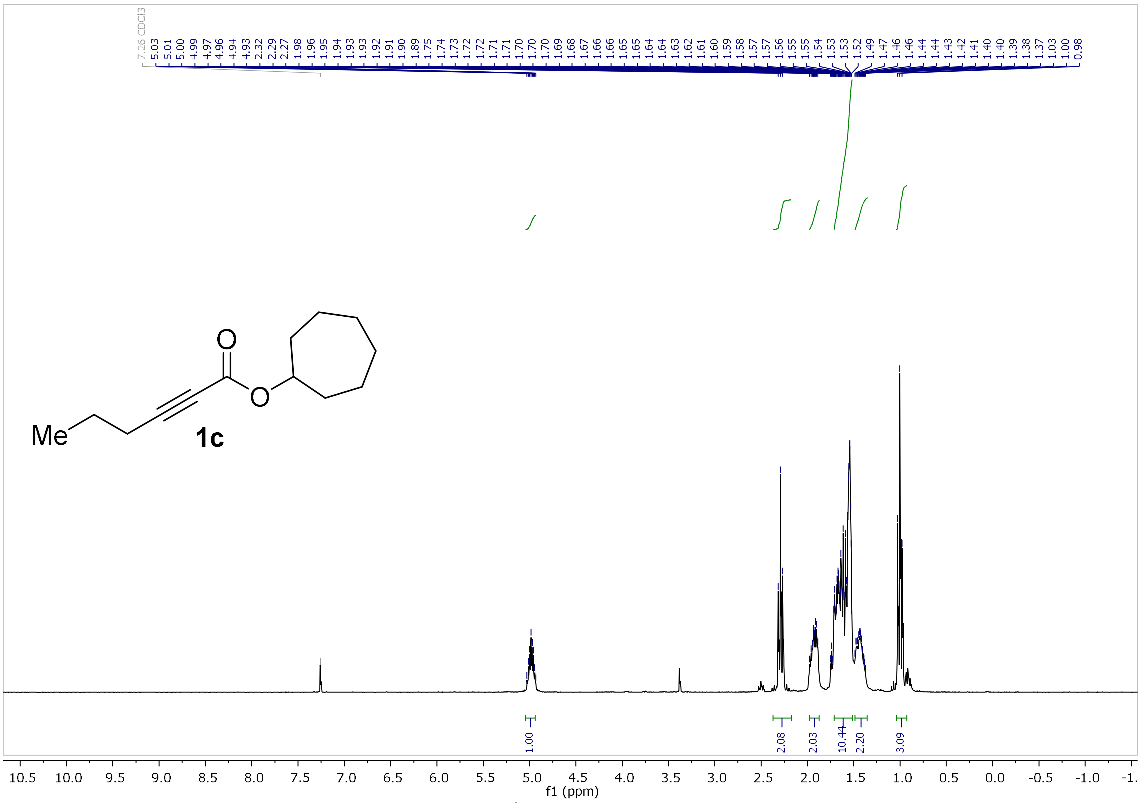

Figure S24. ^{1}H NMR spectrum of **1c.**

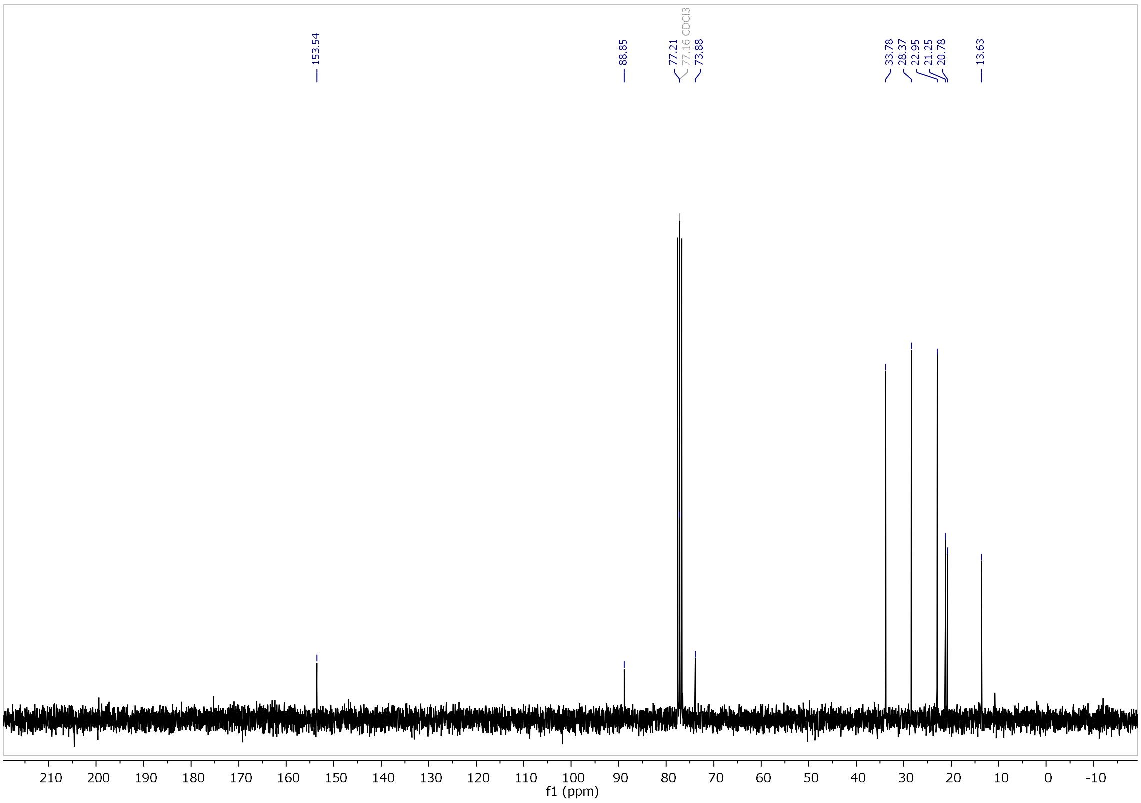

Figure S25. ^{13}C NMR spectrum of **1c.**

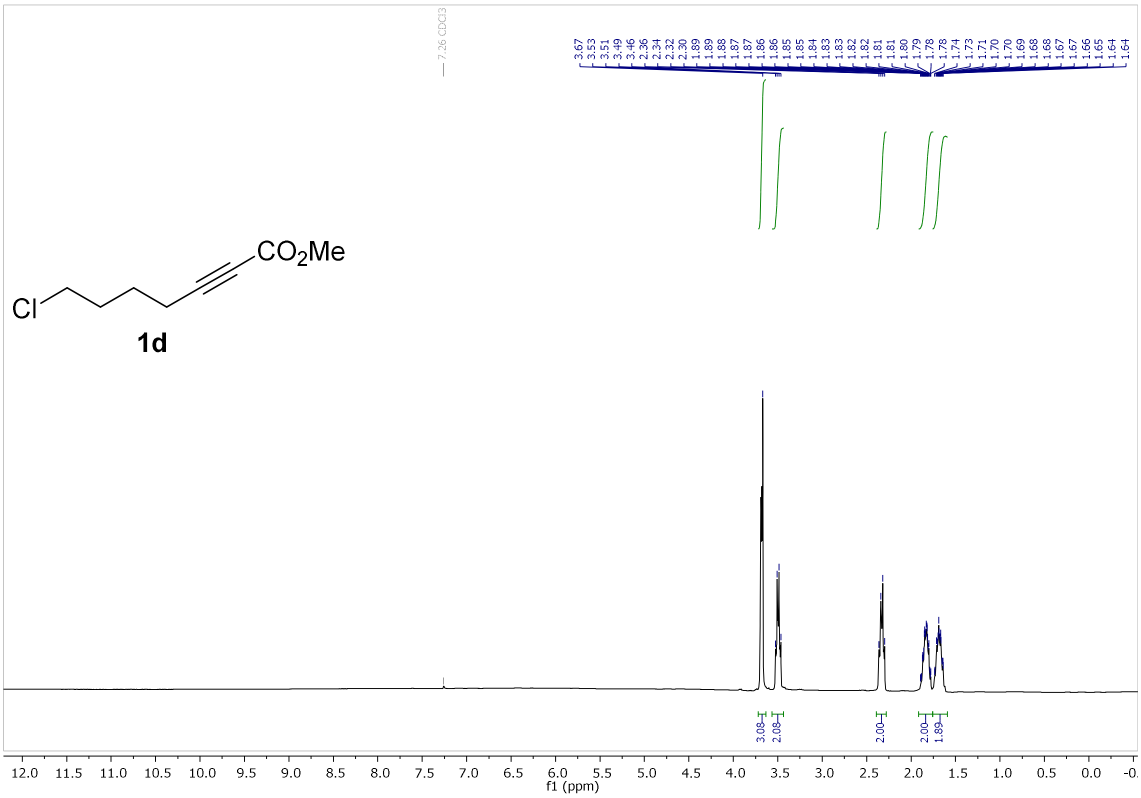

Figure S26. ^{1}H NMR spectrum of **1d.**

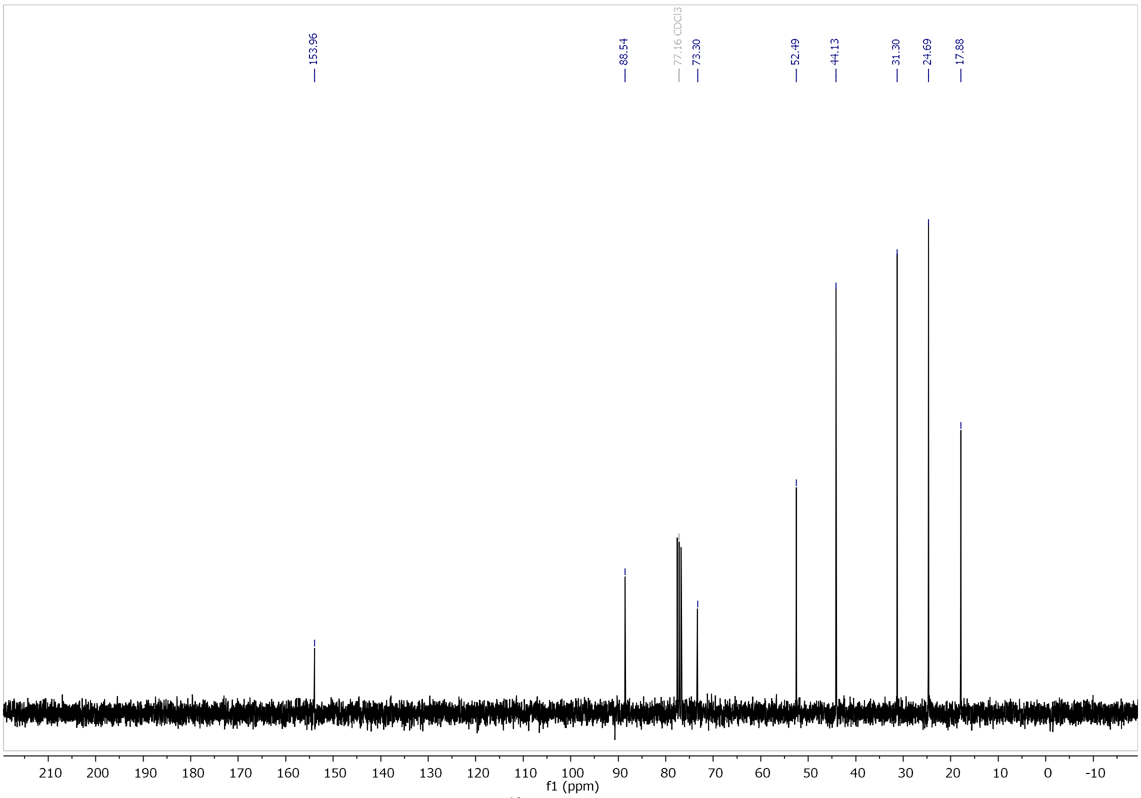

Figure S27. ^{13}C NMR spectrum of **1d.**

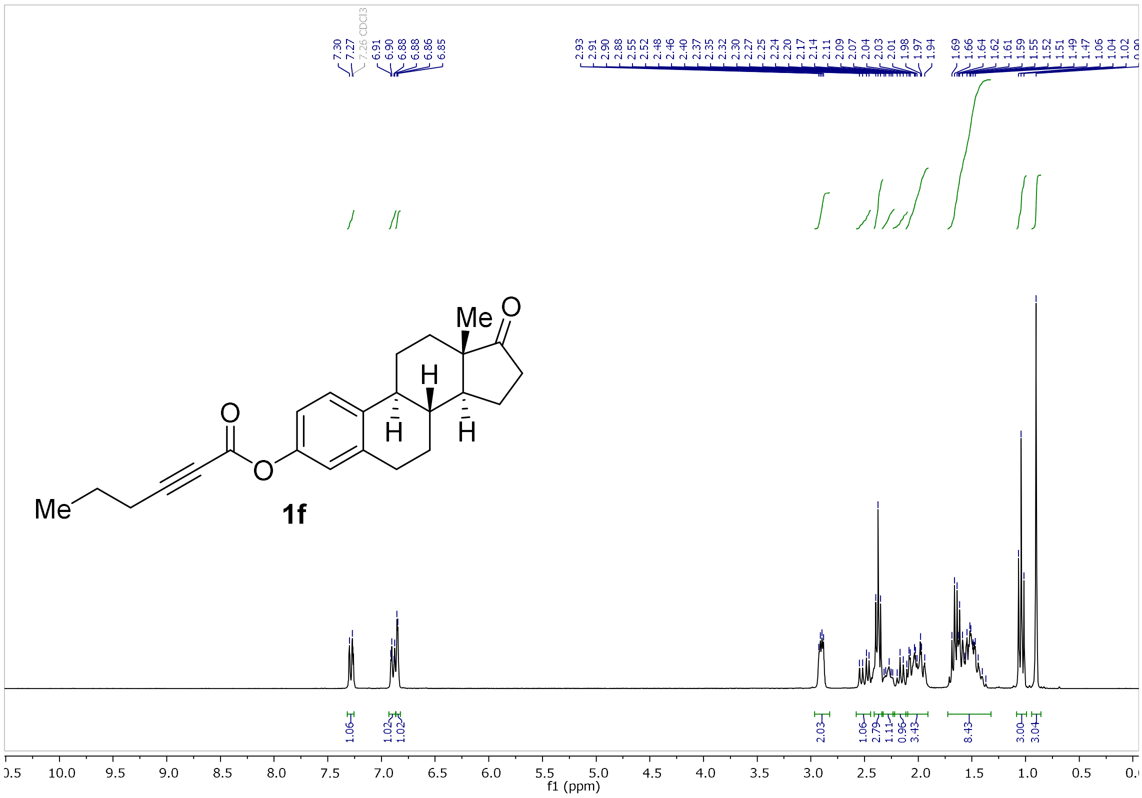

Figure S28. ^{1}H NMR spectrum of **1f.**

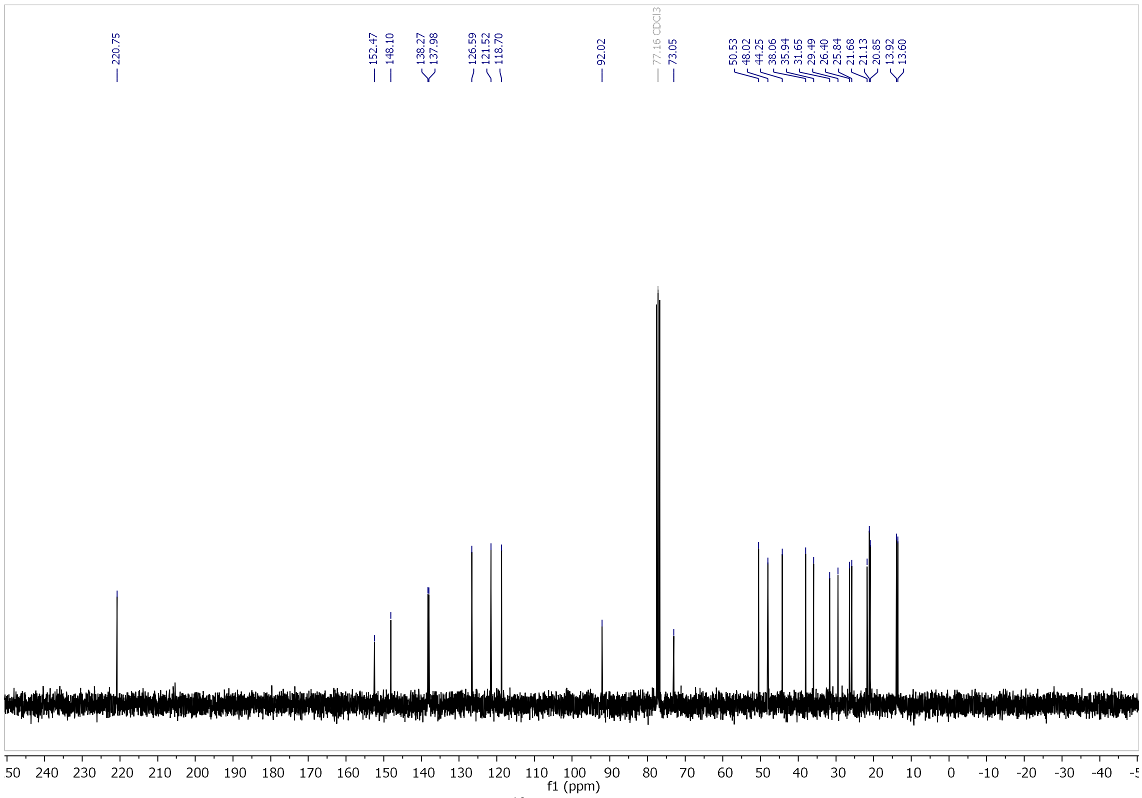

Figure S29. ^{13}C NMR spectrum of **1f.**

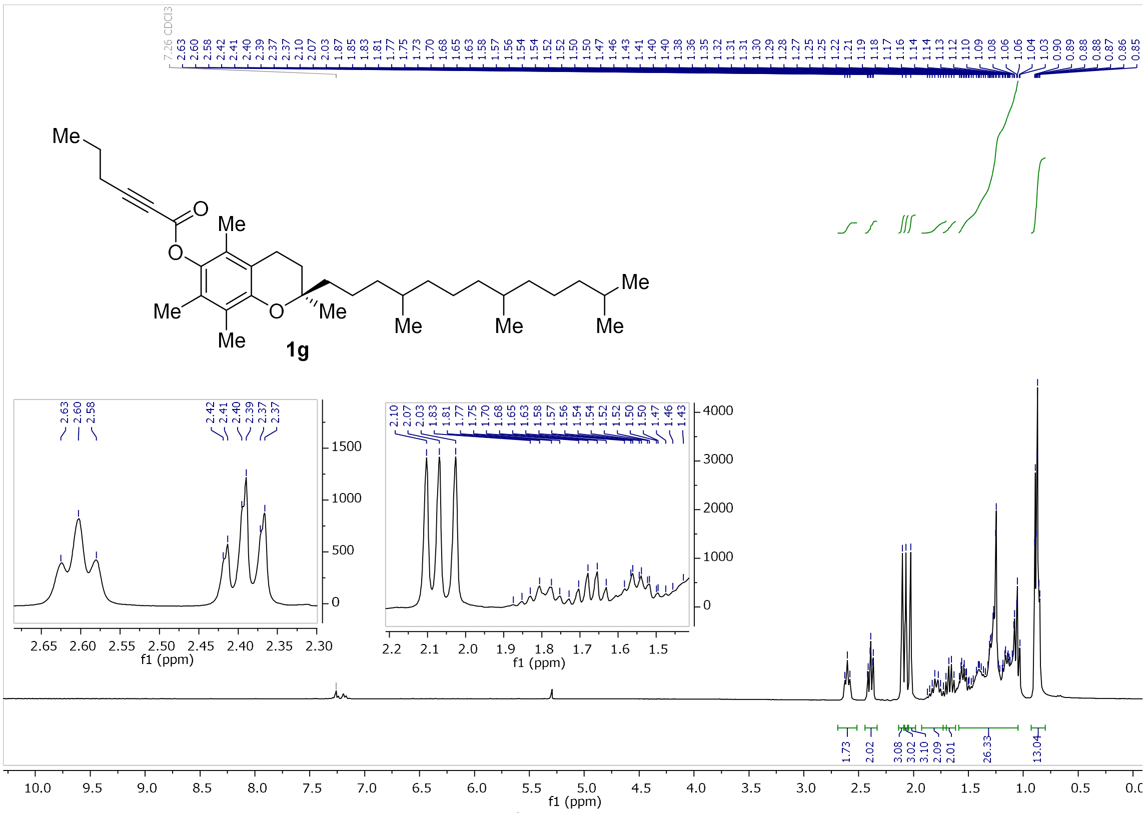

Figure S30. ^{1}H NMR spectrum of **1g.**

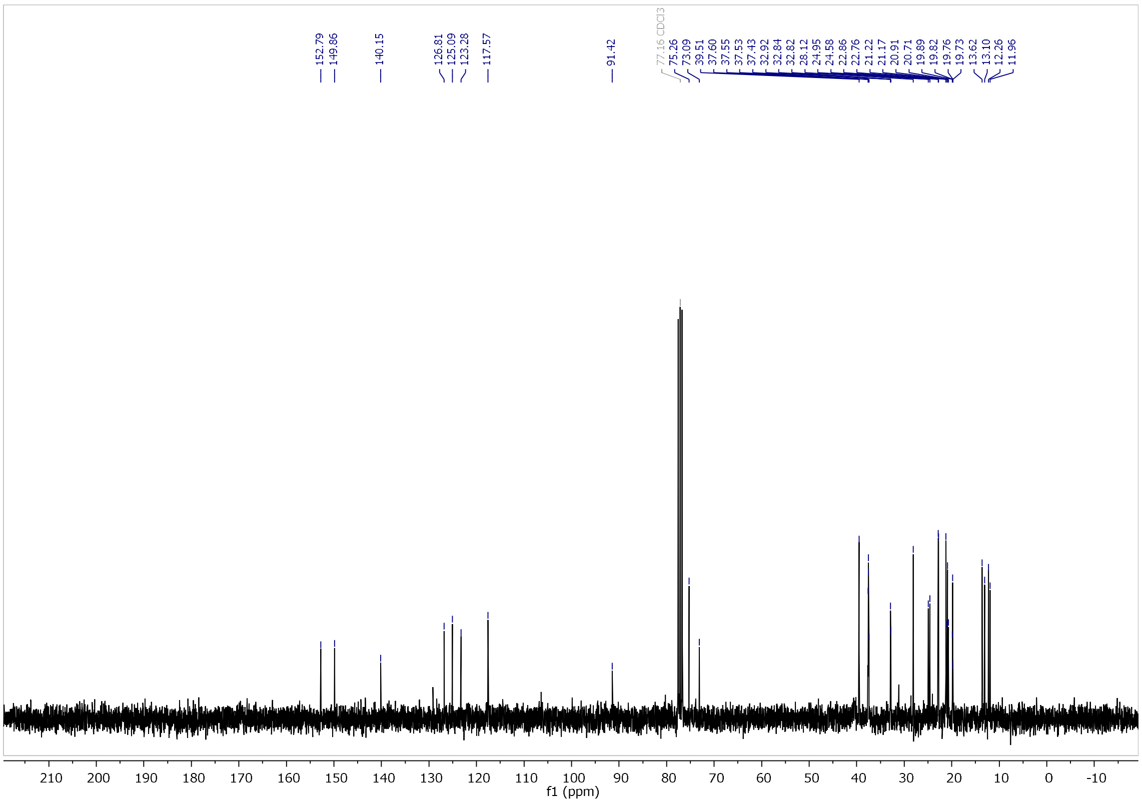

Figure S31. ^{13}C NMR spectrum of **1g.**

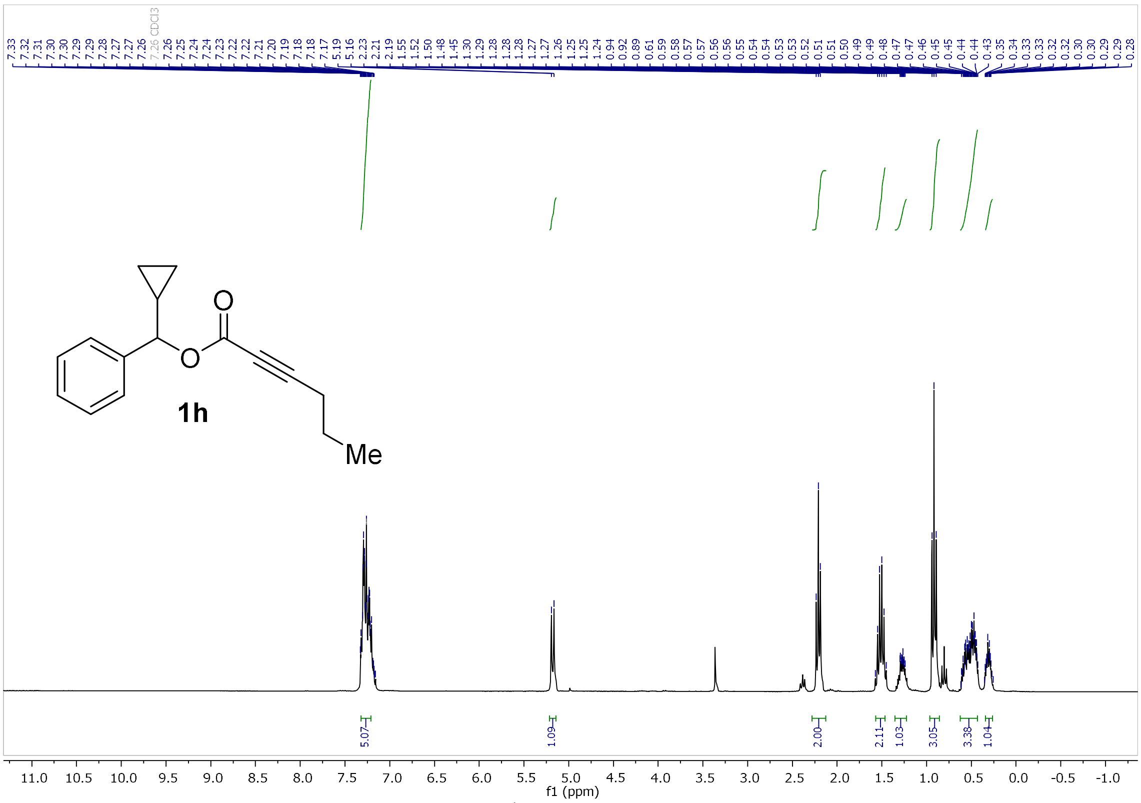

Figure S32. ^{1}H NMR spectrum of **1h.**

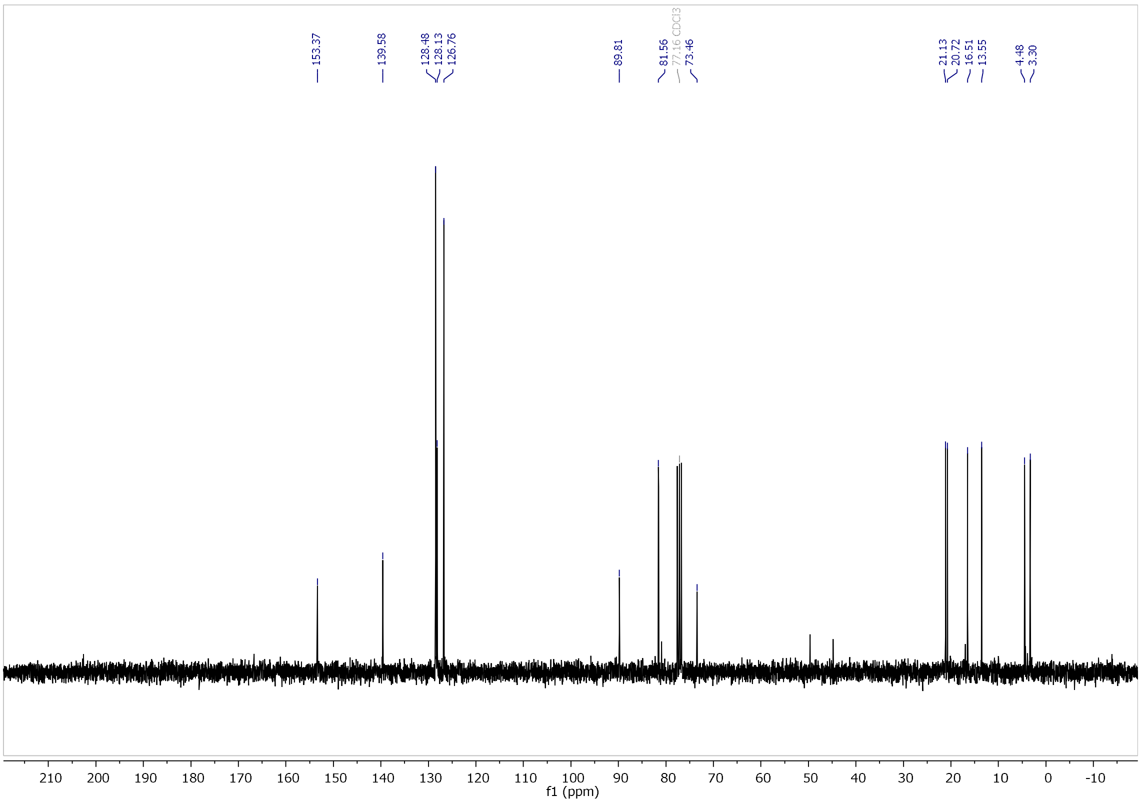

Figure S33. ^{13}C NMR spectrum of **1h.**

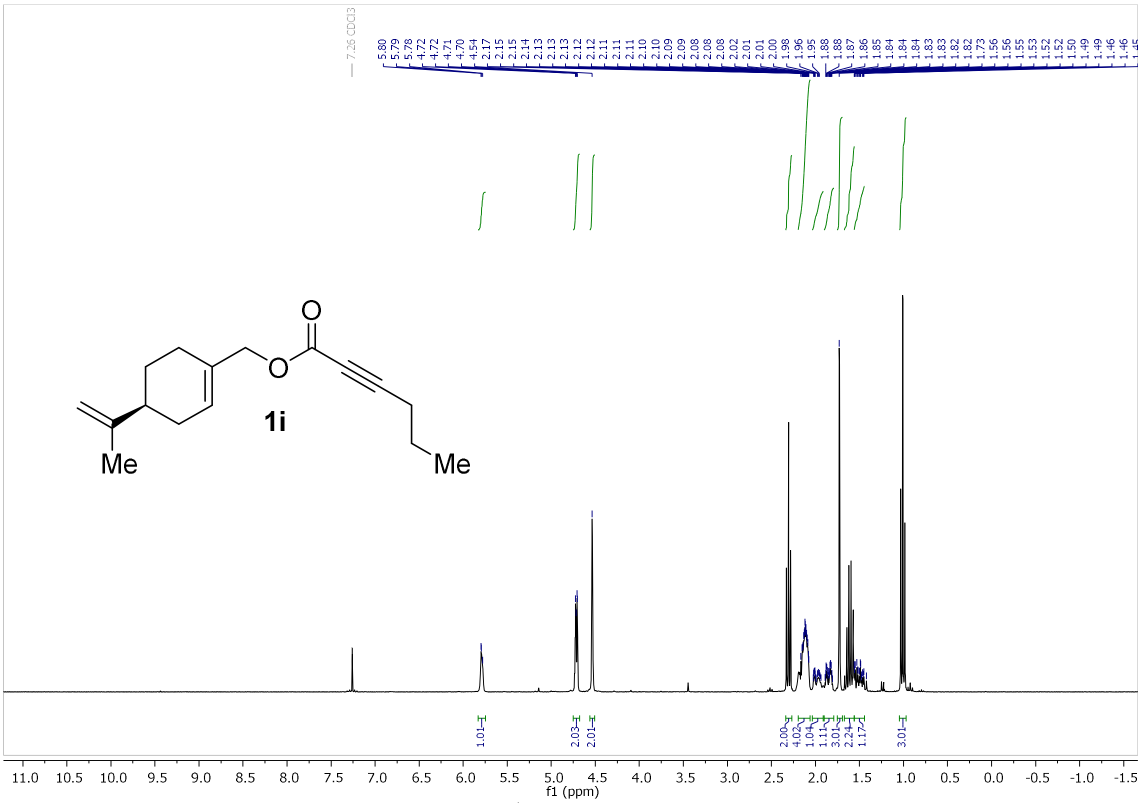

Figure S34. ^{1}H NMR spectrum of **1i.**

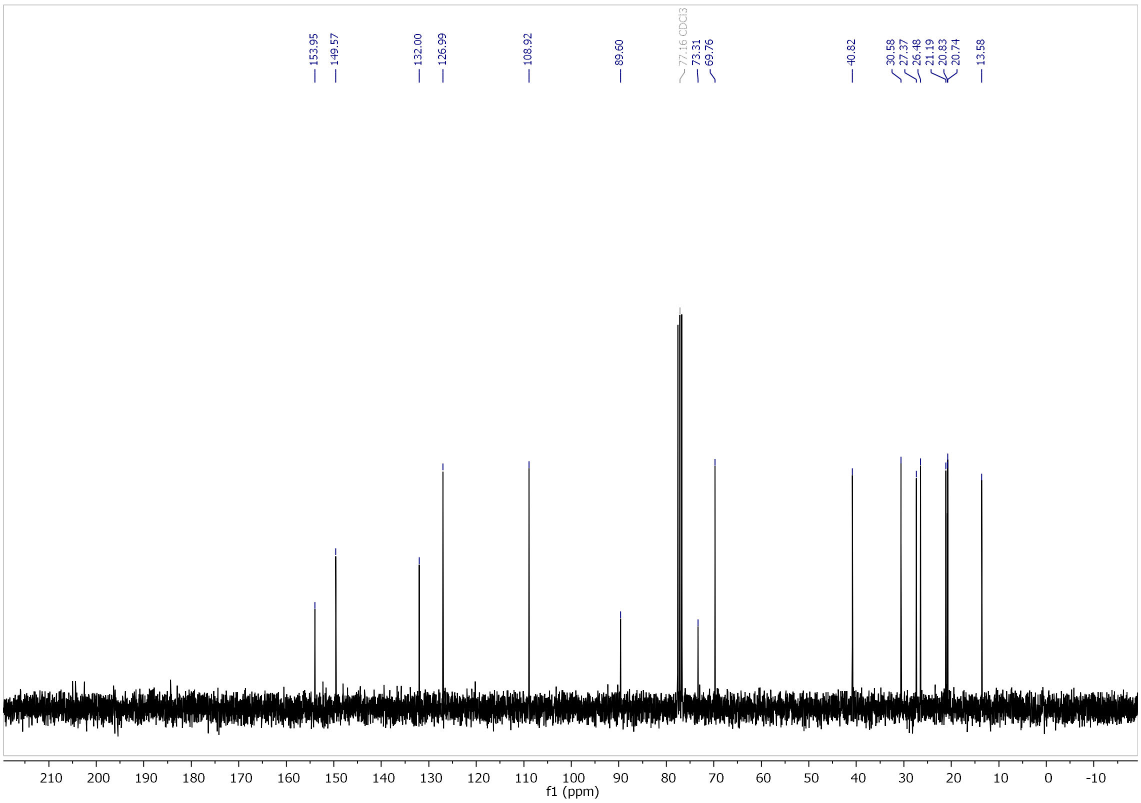

Figure S35. ^{13}C NMR spectrum of **1i.**

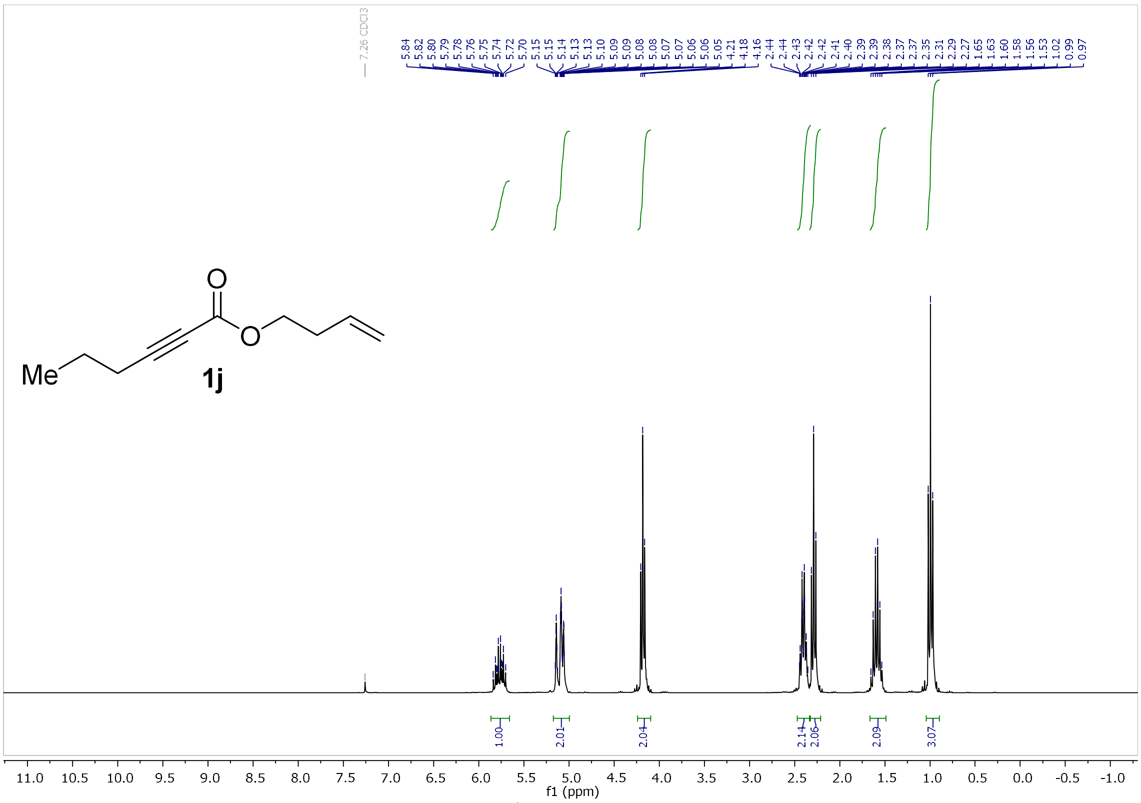

Figure S36. ^{1}H NMR spectrum of **1j.**

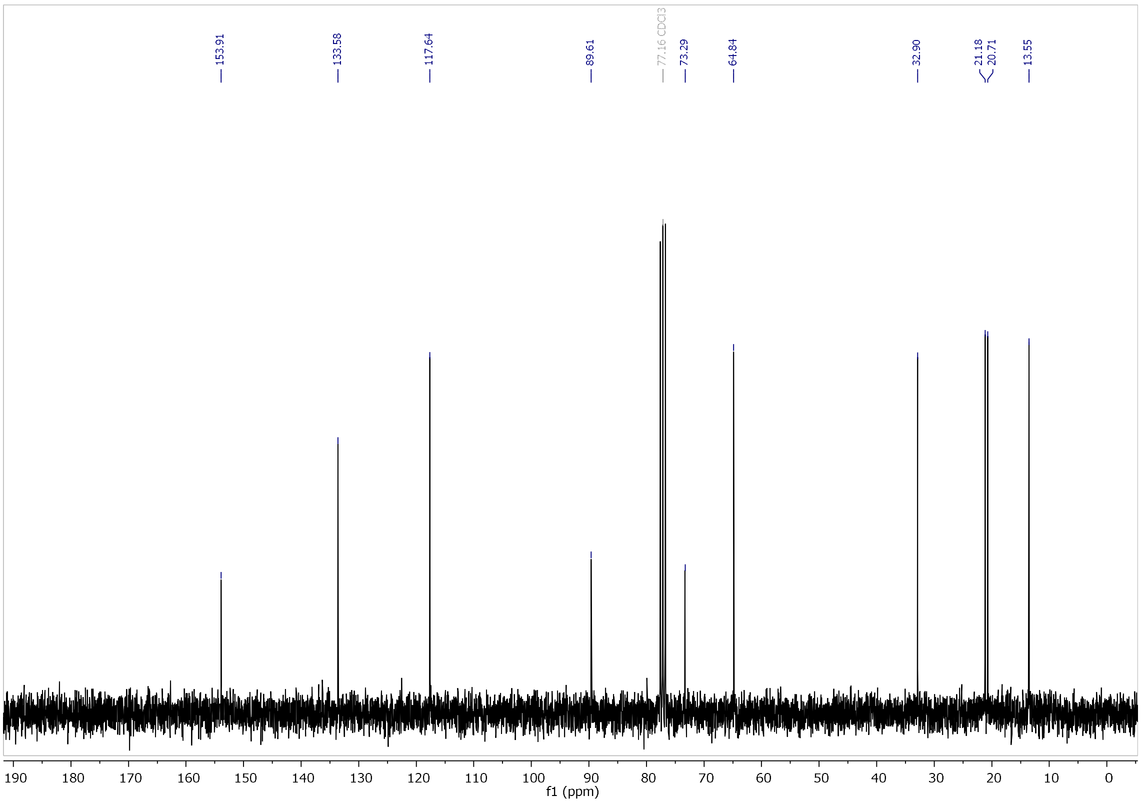

Figure S37. ^{13}C NMR spectrum of **1j.**

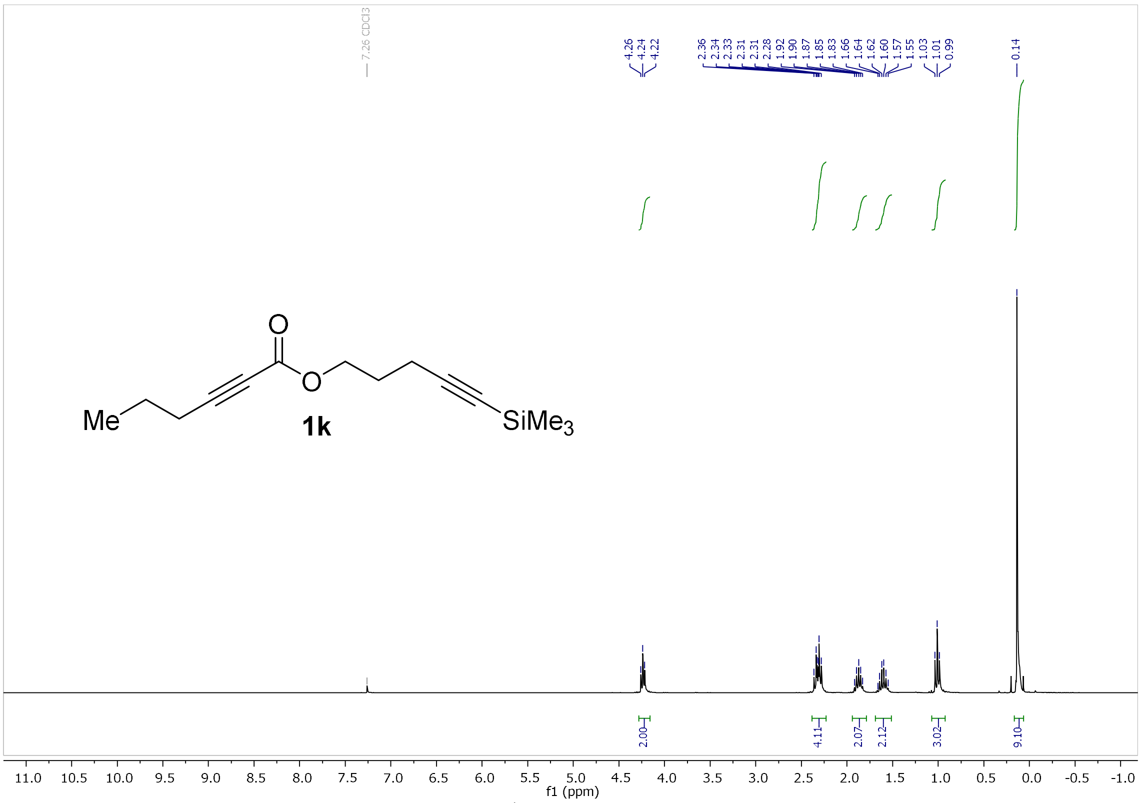

Figure S38. ^{1}H NMR spectrum of **1k.**

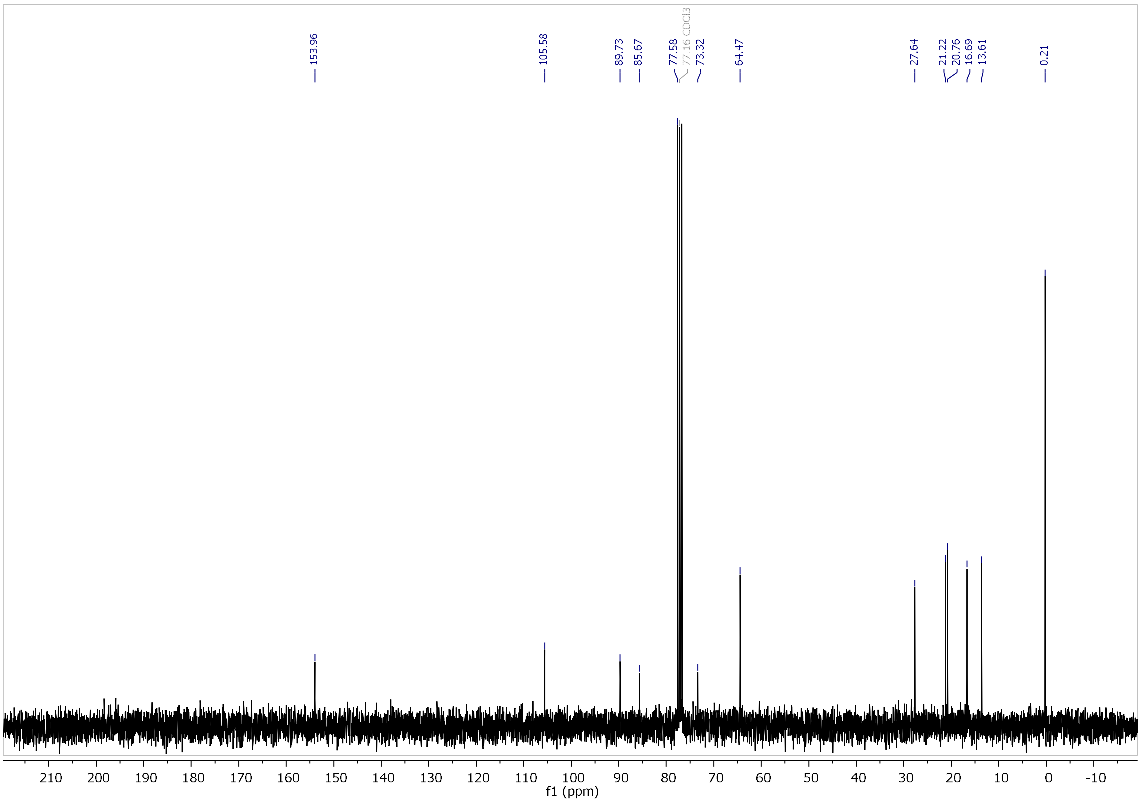

Figure S39. ^{13}C NMR spectrum of **1k.**

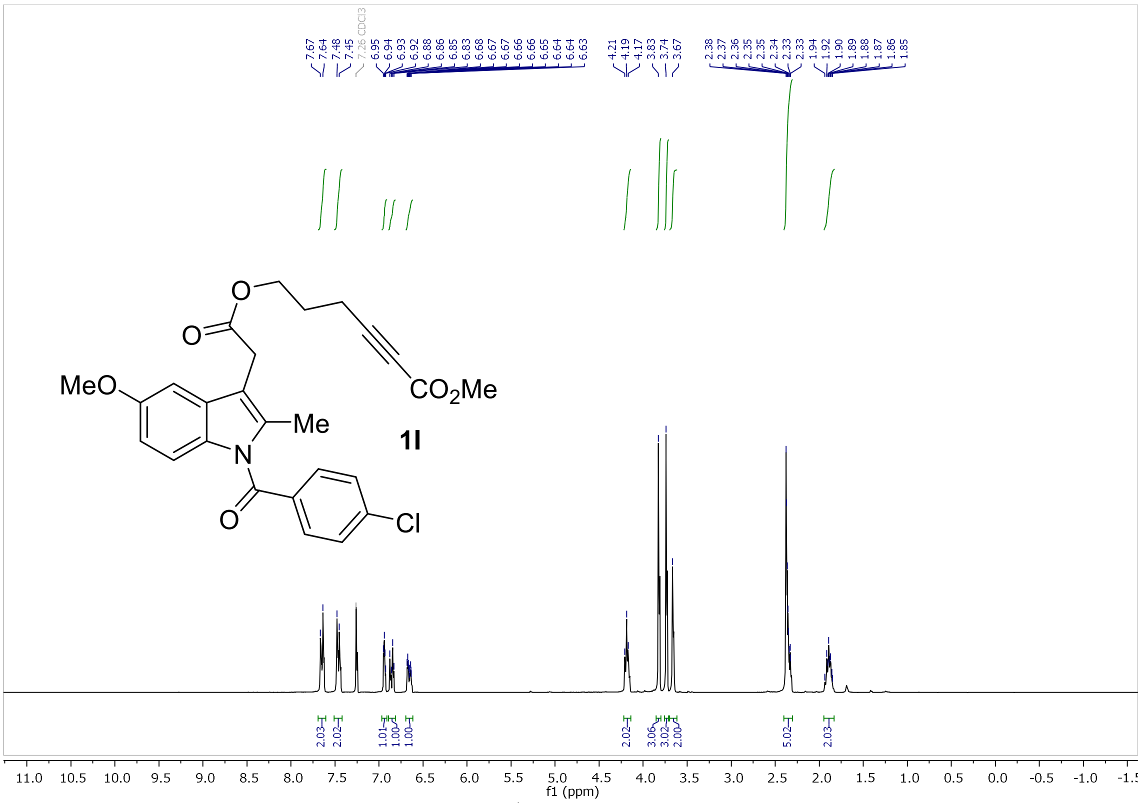

Figure S40. ^{1}H NMR spectrum of **1l.**

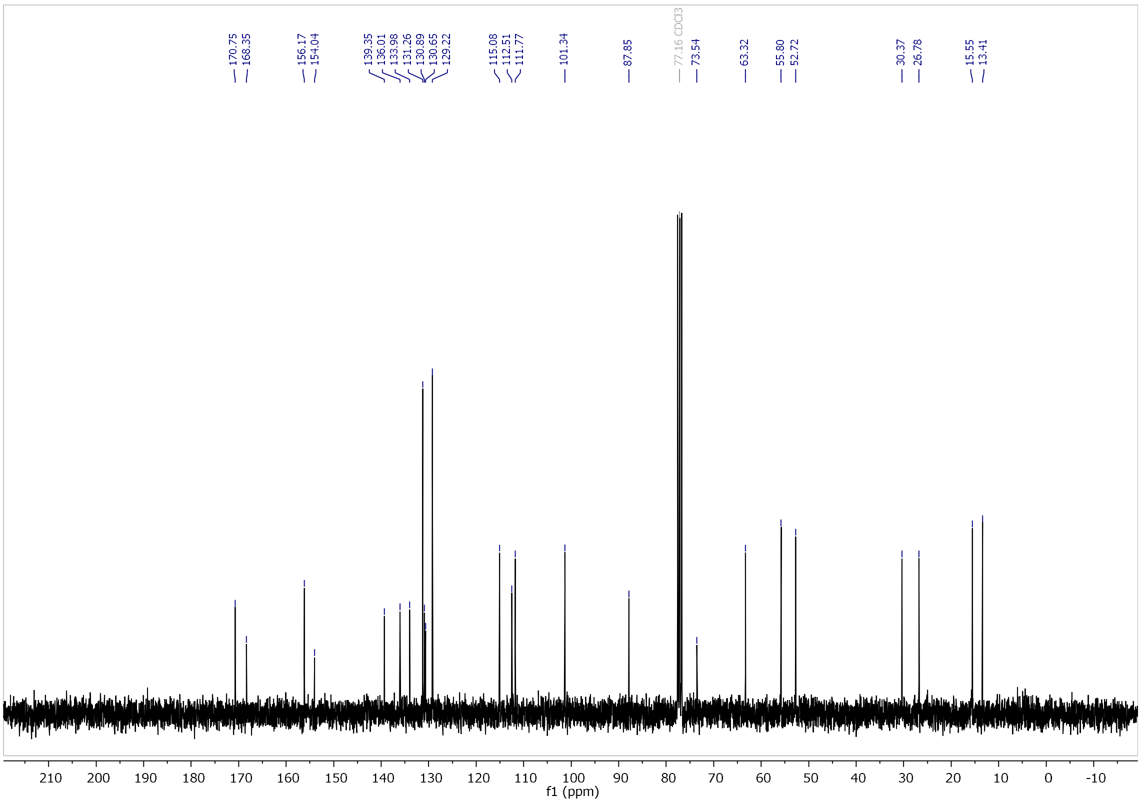

Figure S41. ^{13}C NMR spectrum of **1l.**

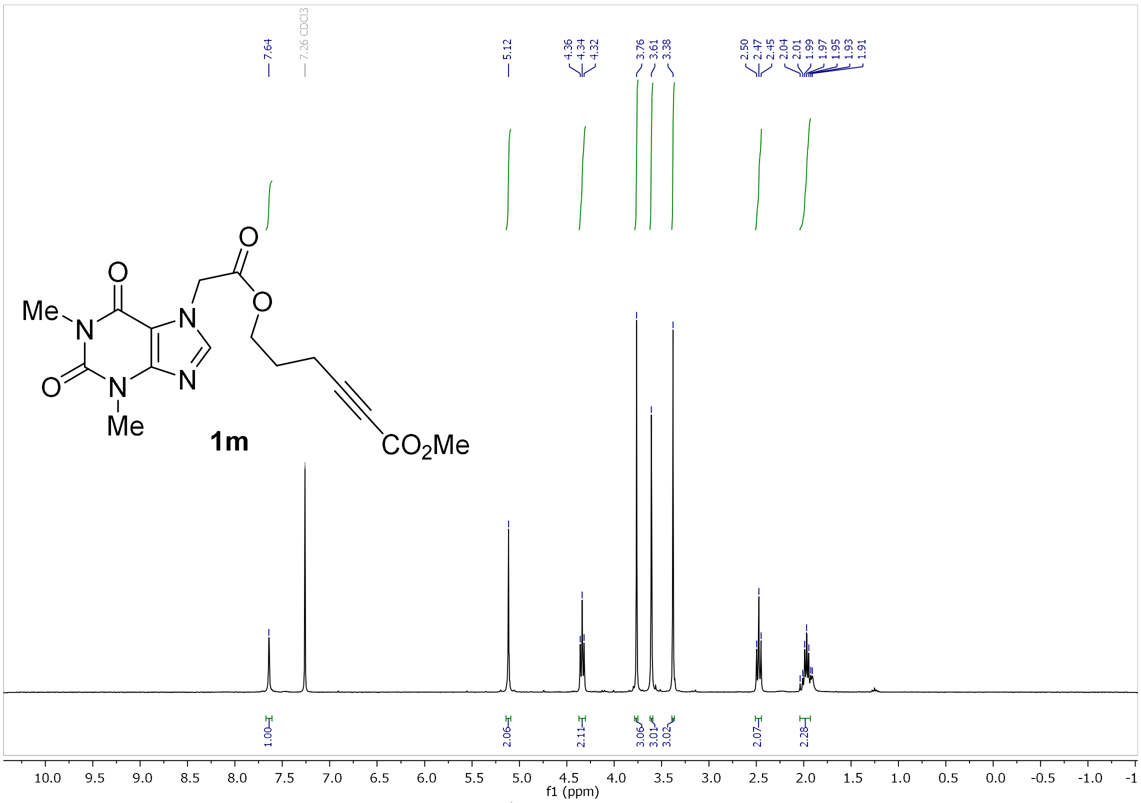

Figure S42. ^{1}H NMR spectrum of **1m.**

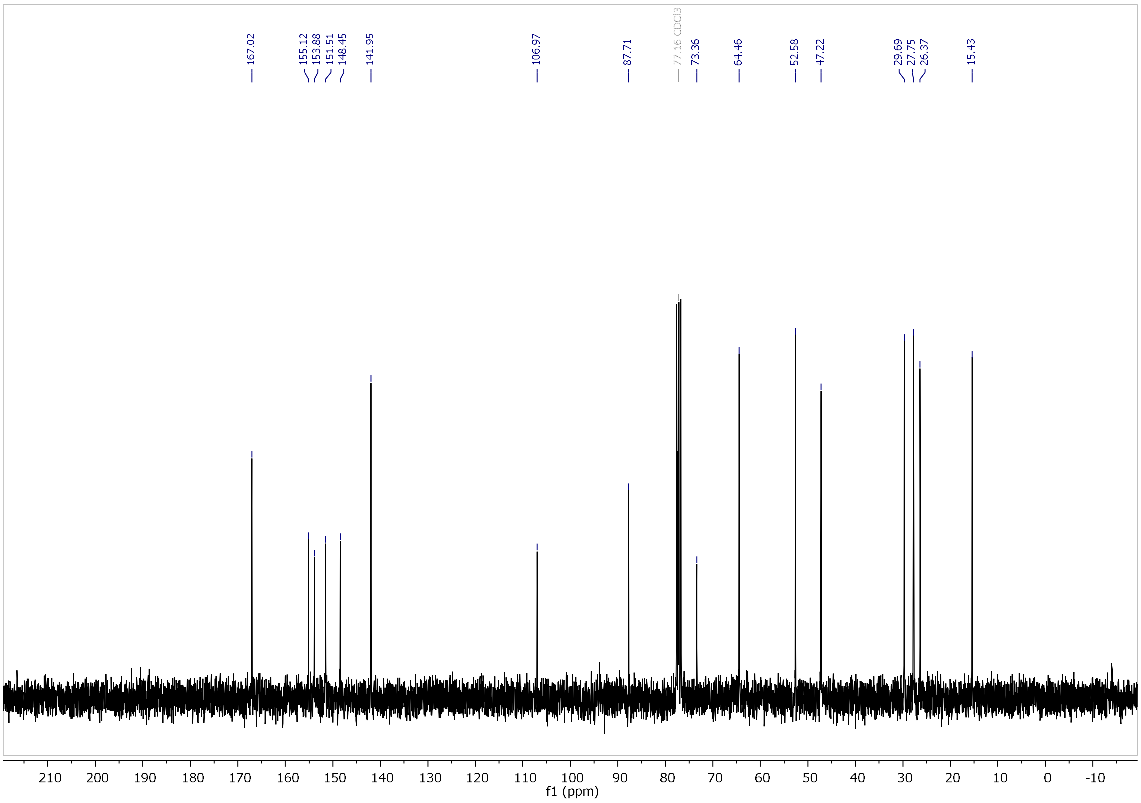

Figure S43. ^{13}C NMR spectrum of **1m.**

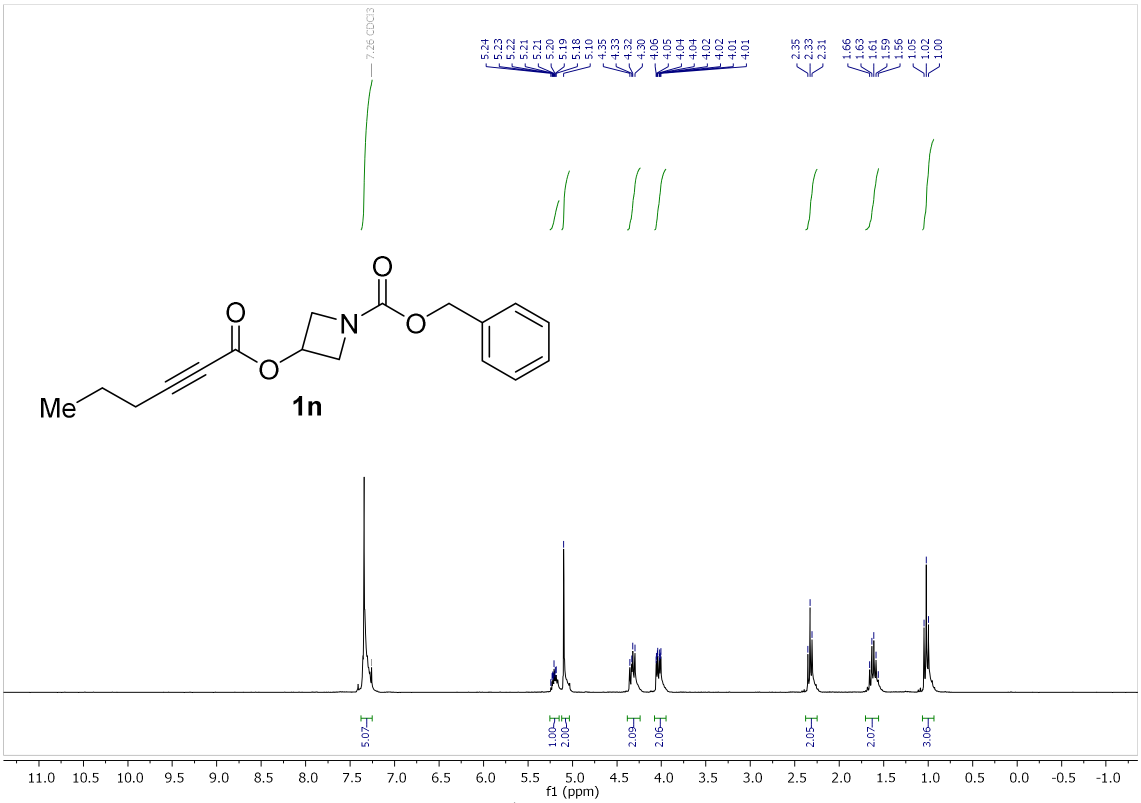

Figure S44. ^{1}H NMR spectrum of **1n.**

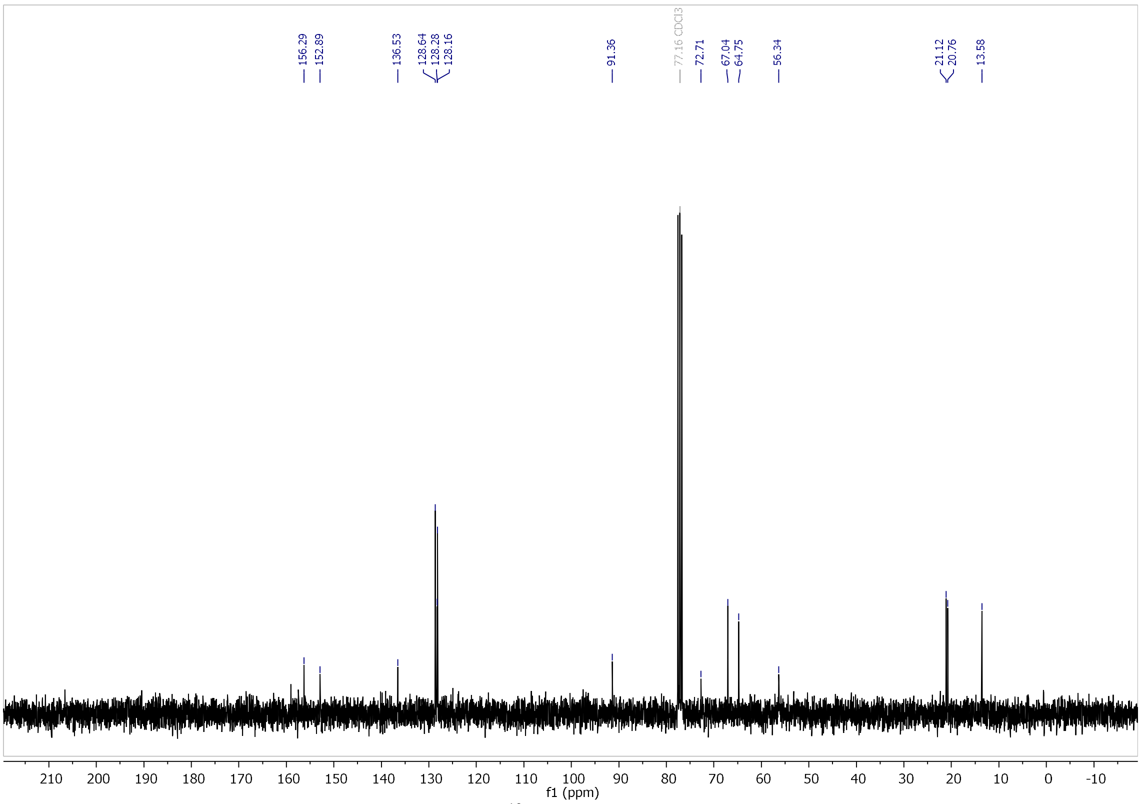

Figure S45. ^{13}C NMR spectrum of **1n.**

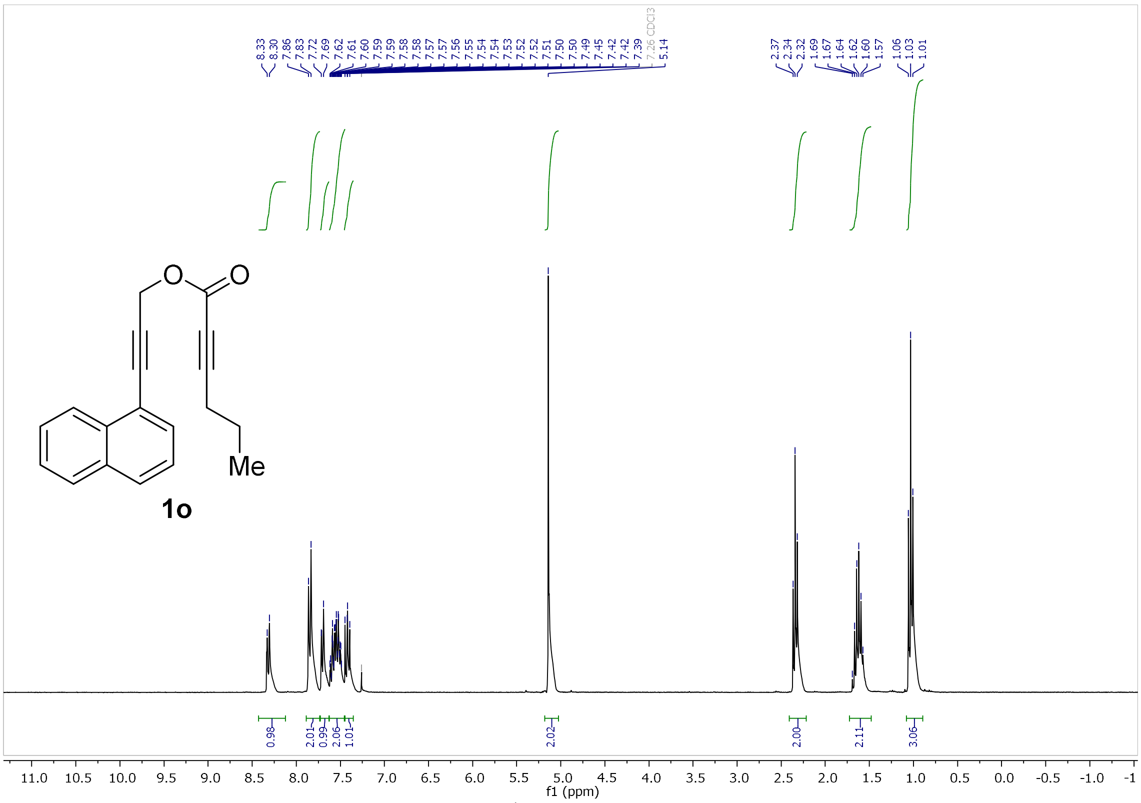

Figure S46. ^{1}H NMR spectrum of **1o.**

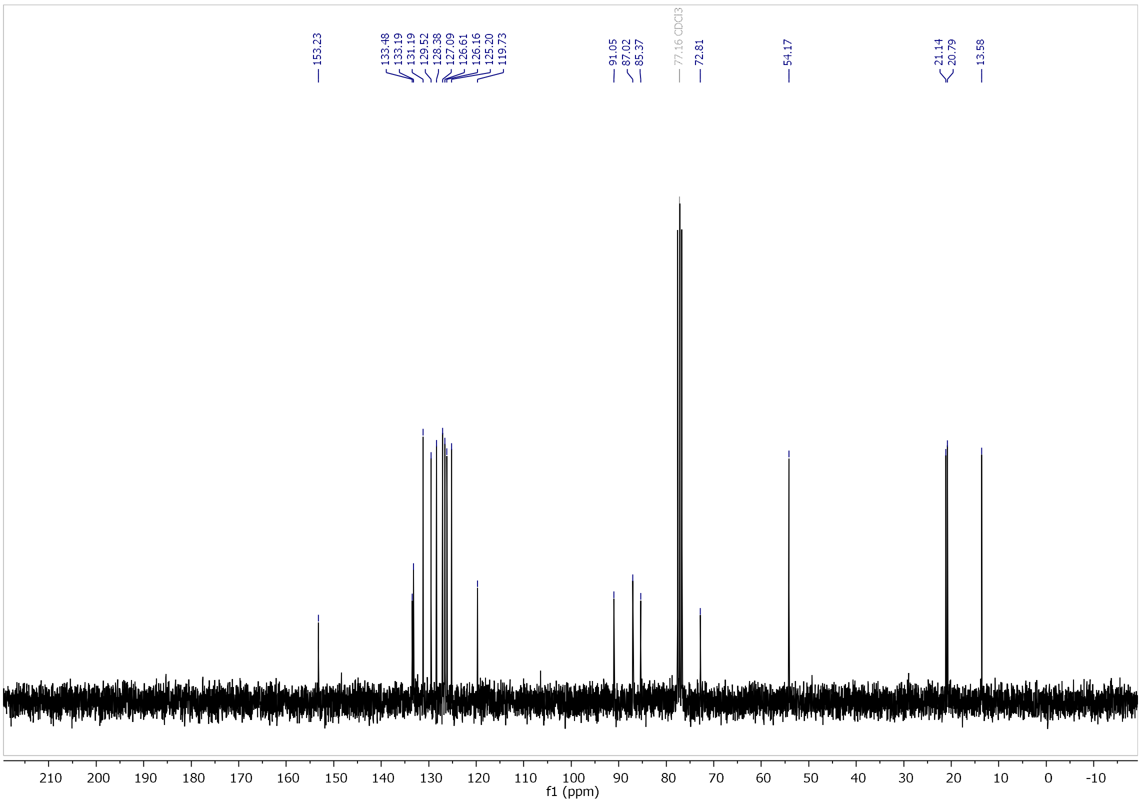

Figure S47. ^{13}C NMR spectrum of **1o.**

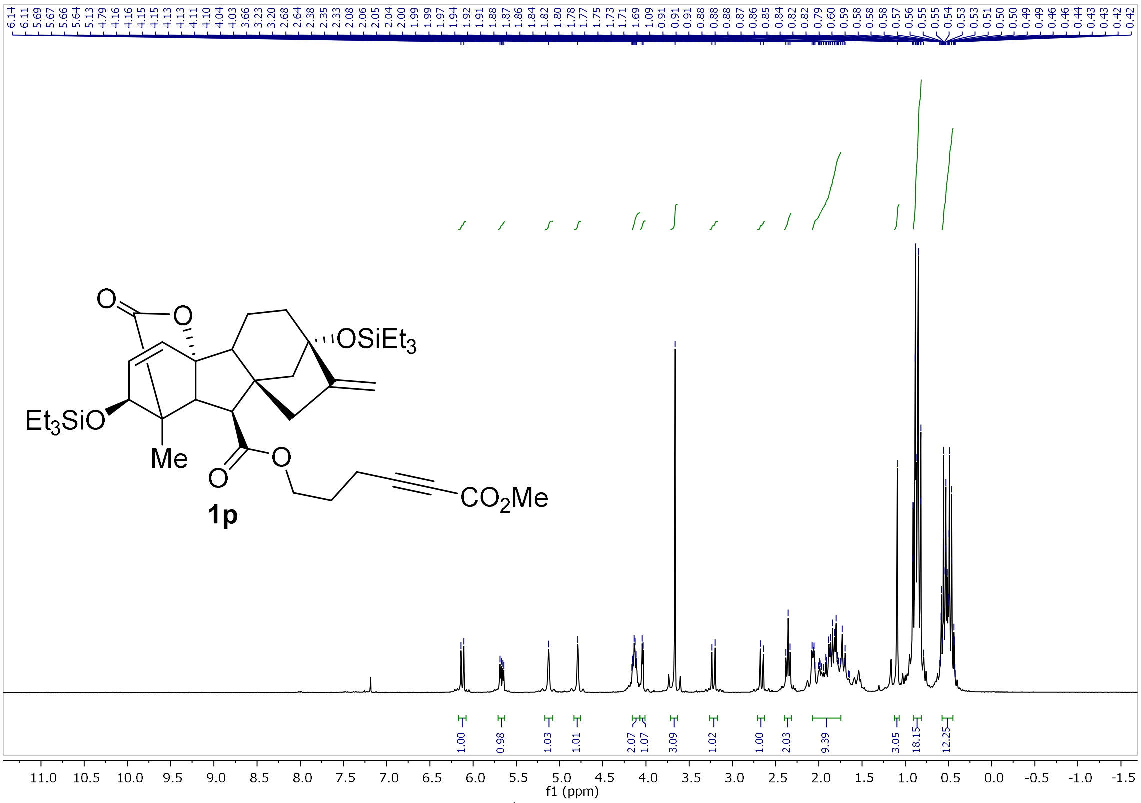

Figure S48. ^{1}H NMR spectrum of **1p.**

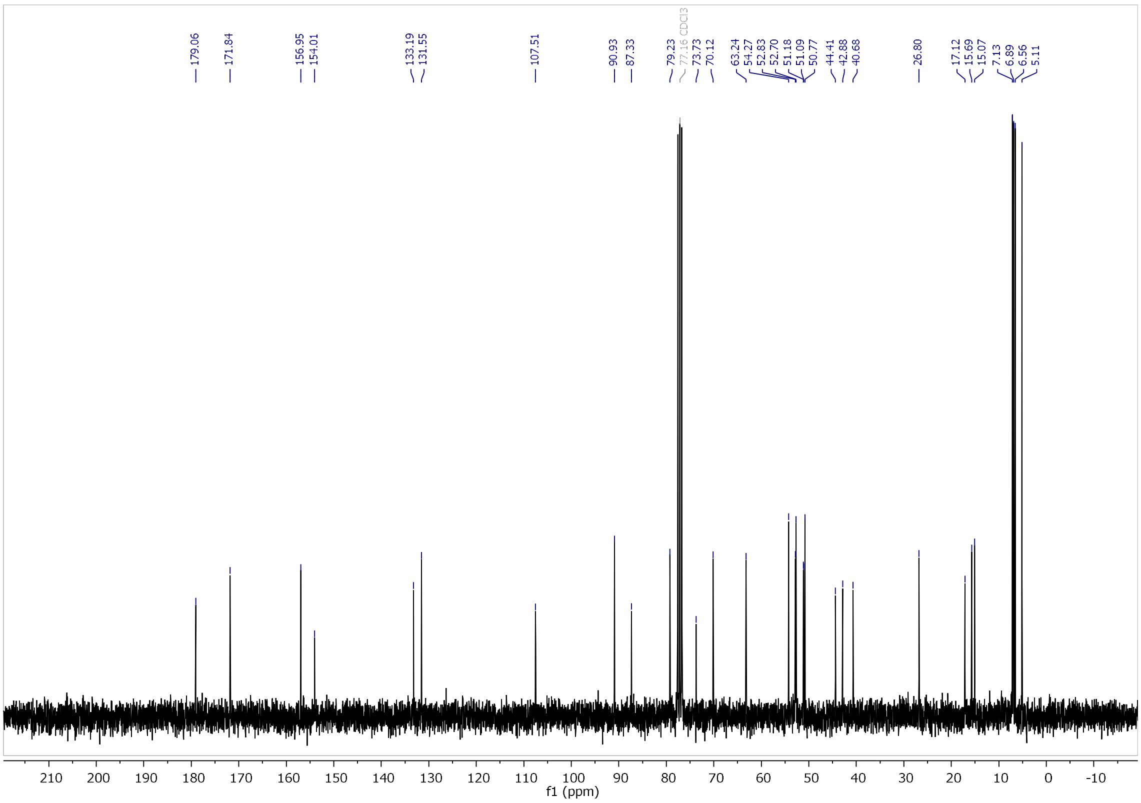

Figure S49. ^{13}C NMR spectrum of **1p.**

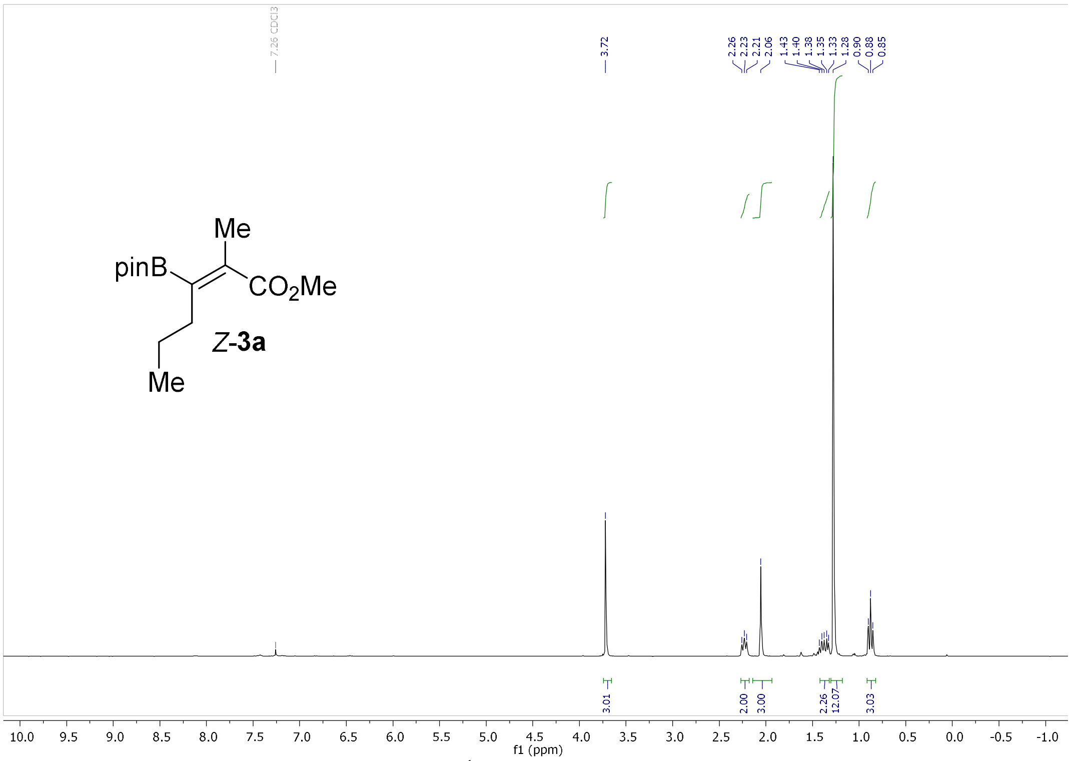

Figure S50. ^{1}H NMR spectrum of *Z*-**3a.**

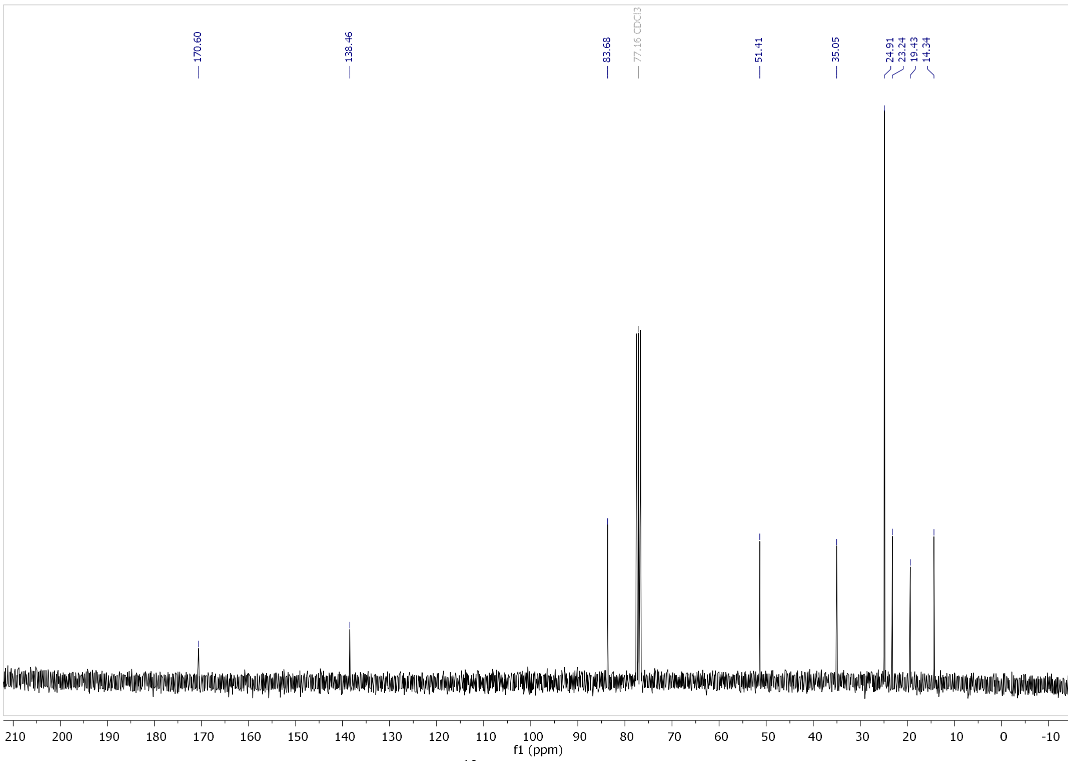

Figure S51. ^{13}C NMR spectrum of *Z*-**3a.**

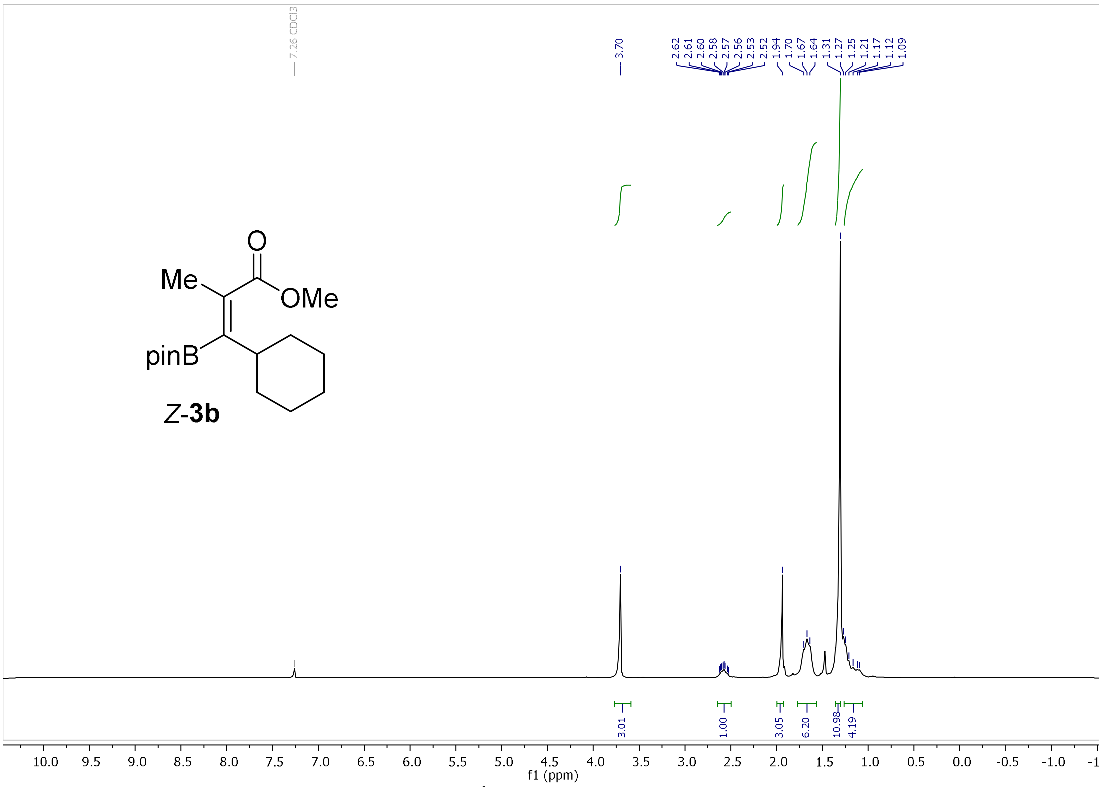

Figure S52. ^{1}H NMR spectrum of *Z*-**3b.**

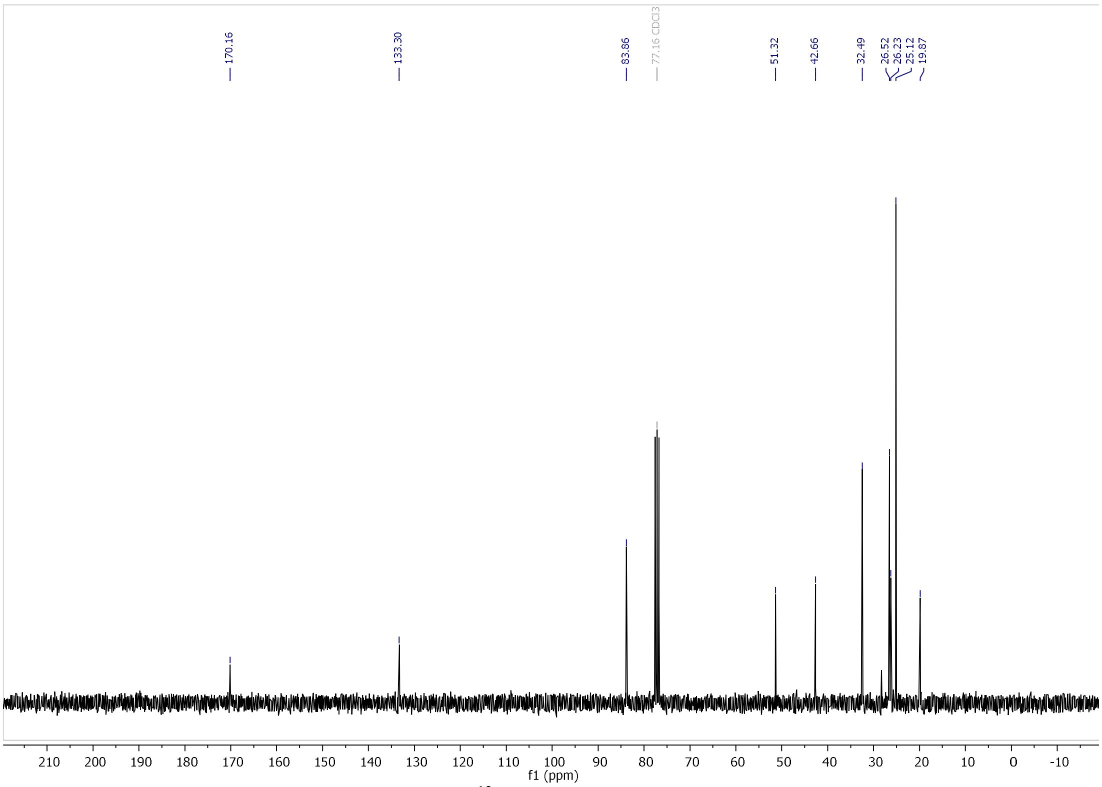

Figure S53. ^{13}C NMR spectrum of *Z*-**3b.**

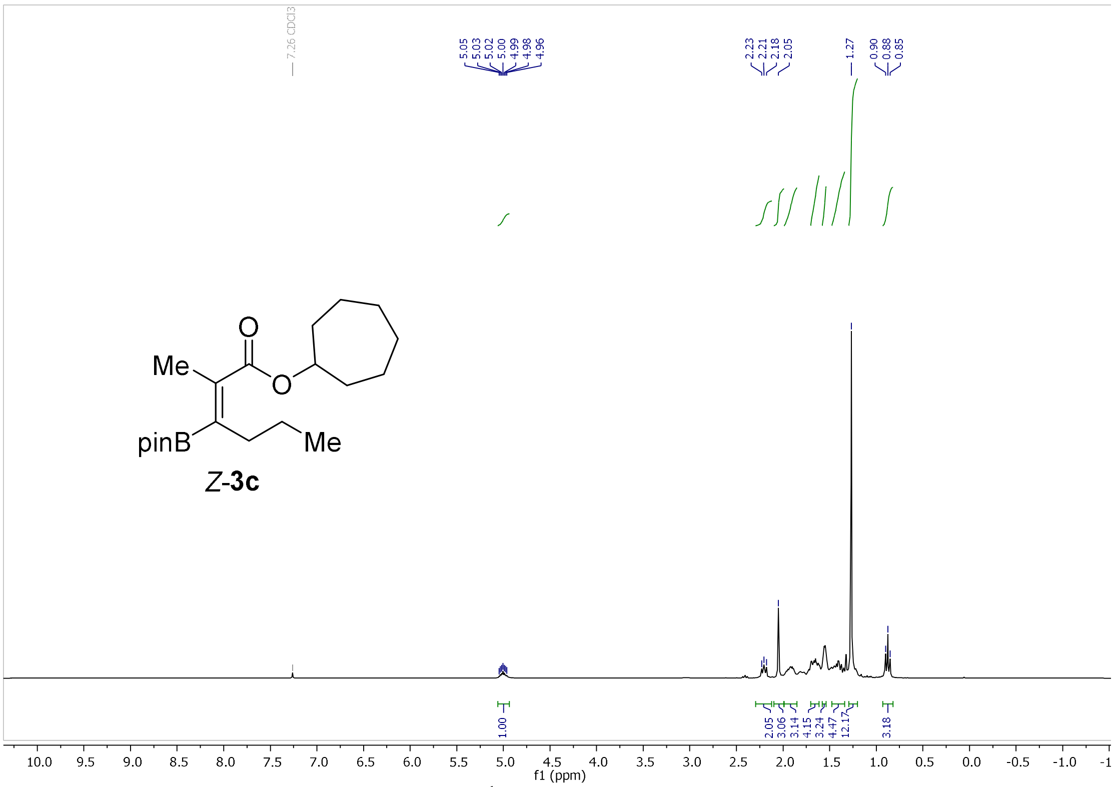

Figure S54. ^{1}H NMR spectrum of *Z*-**3c.**

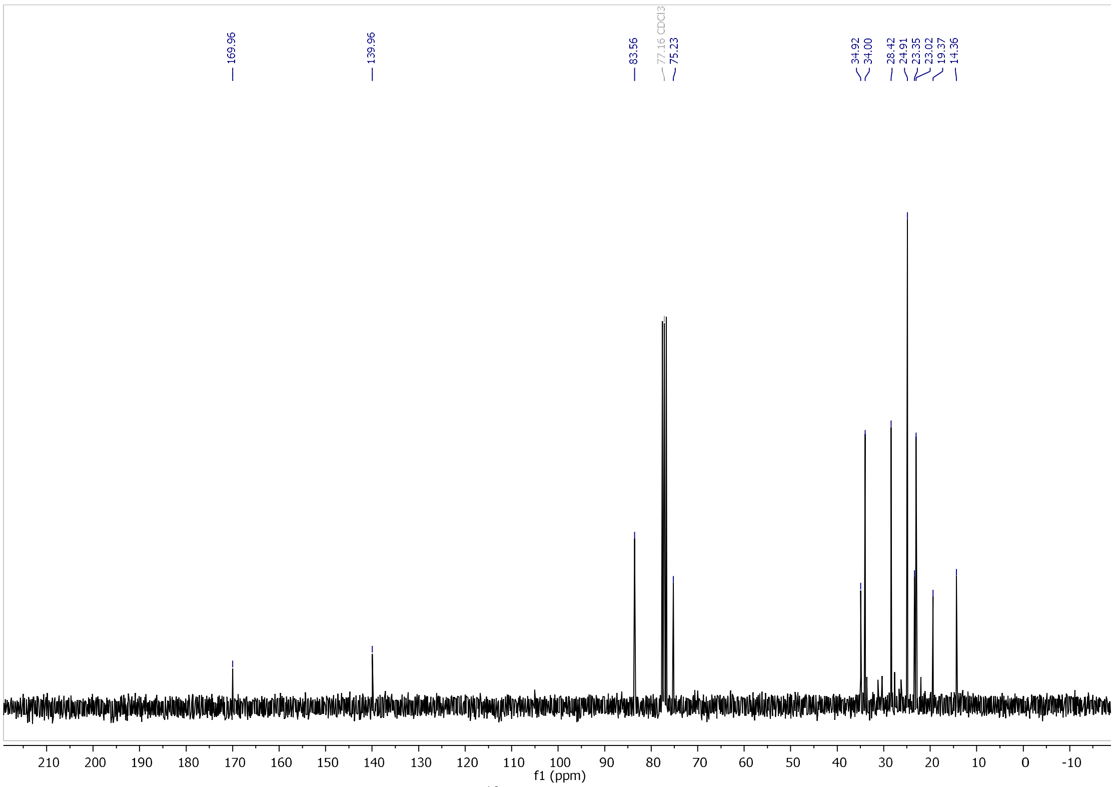

Figure S55. ^{13}C NMR spectrum of *Z*-**3c.**

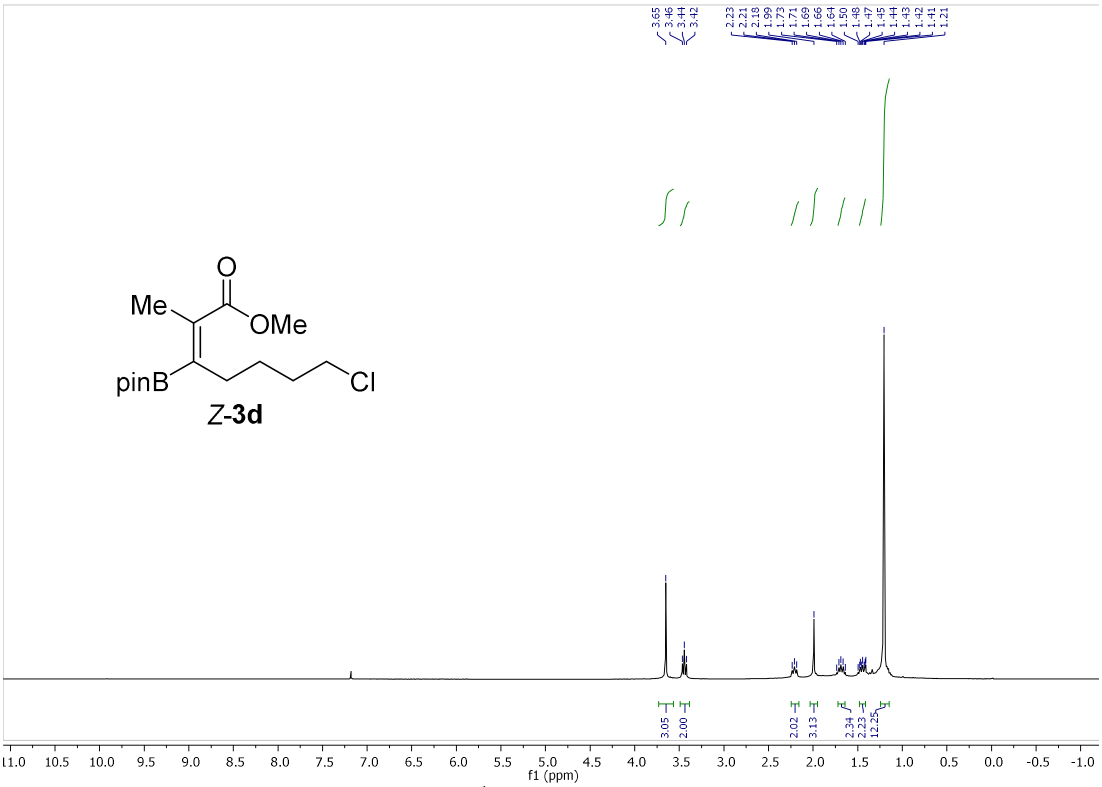

Figure S56. ^{1}H NMR spectrum of *Z*-**3d.**

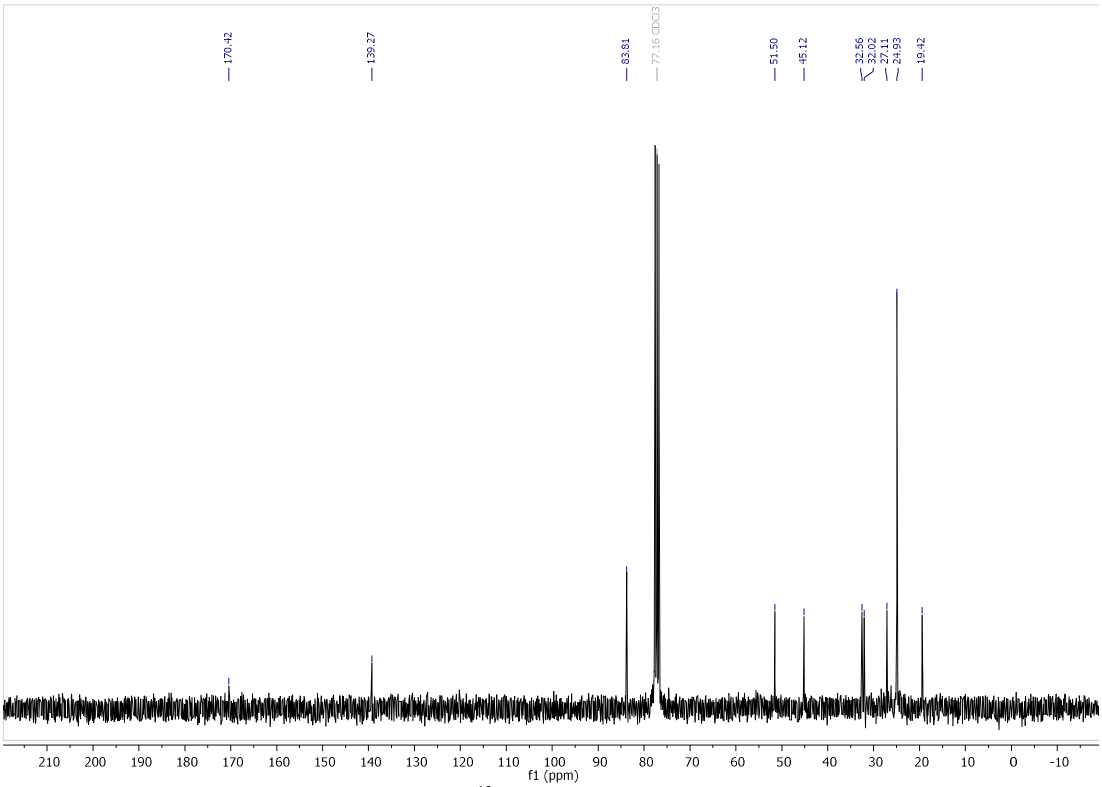

Figure S57. ^{13}C NMR spectrum of *Z*-**3d.**

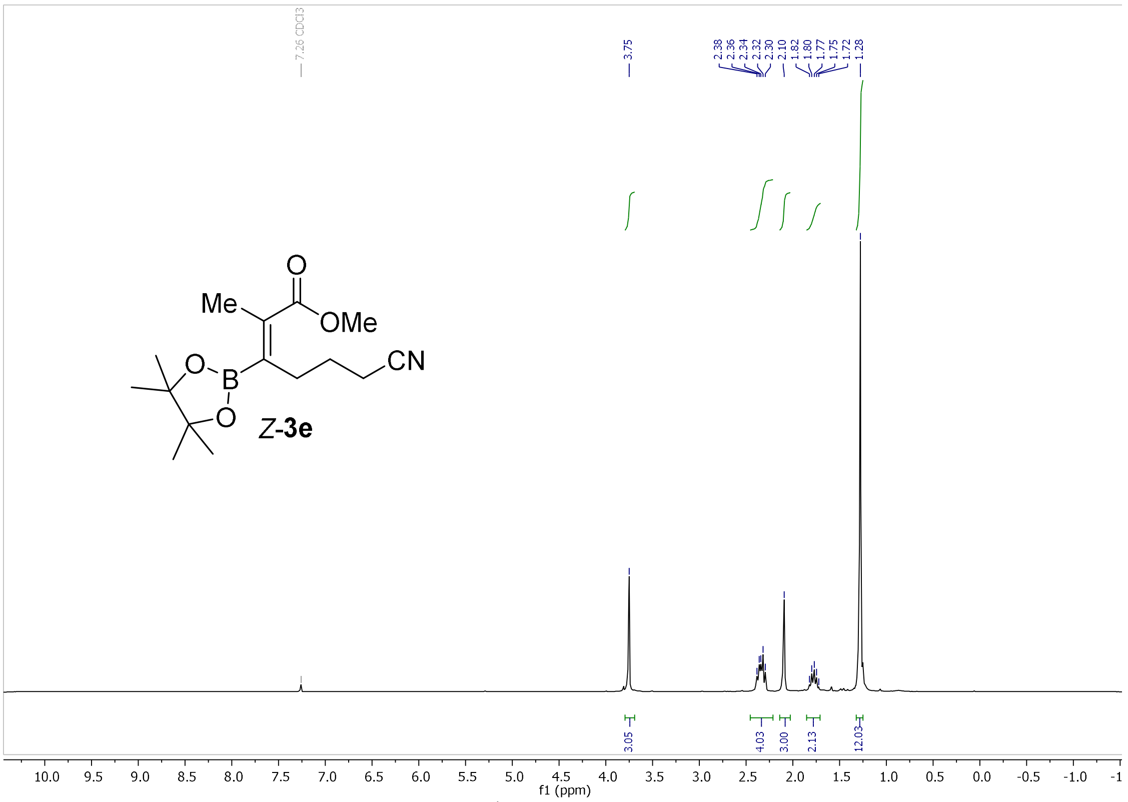

Figure S58. ^{1}H NMR spectrum of *Z*-**3e.**

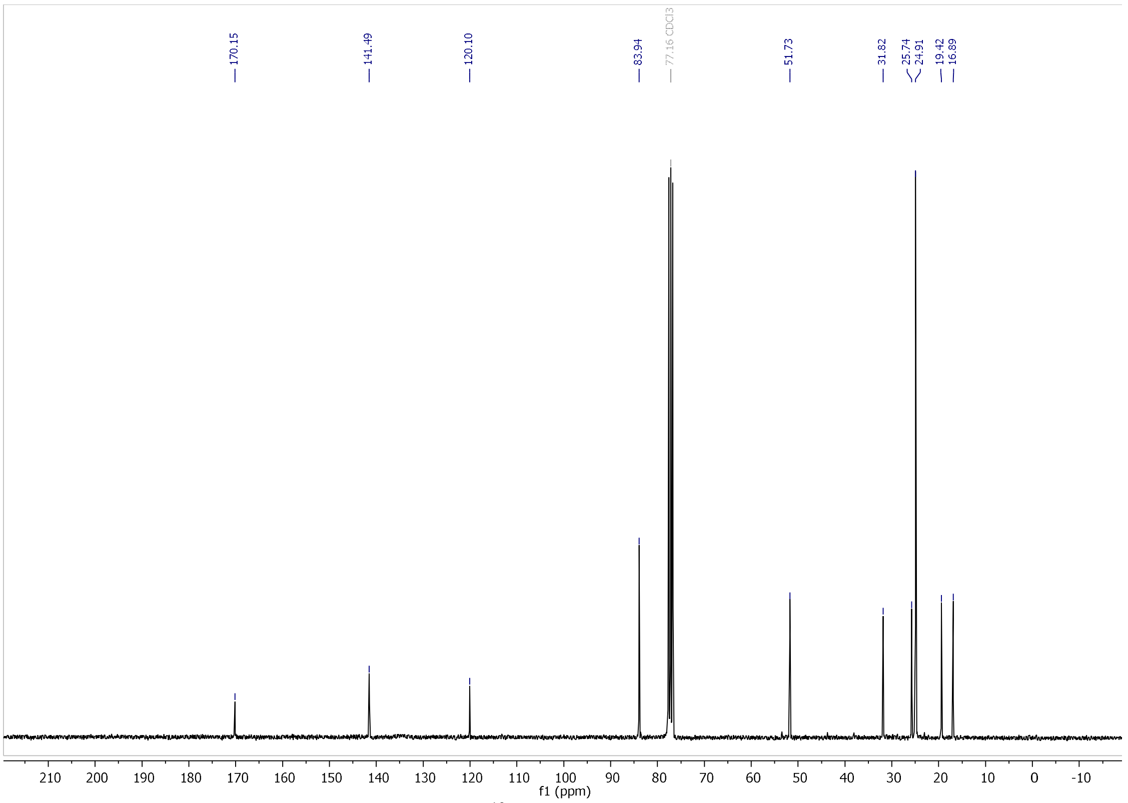

Figure S59. ^{13}C NMR spectrum of *Z*-**3e.**

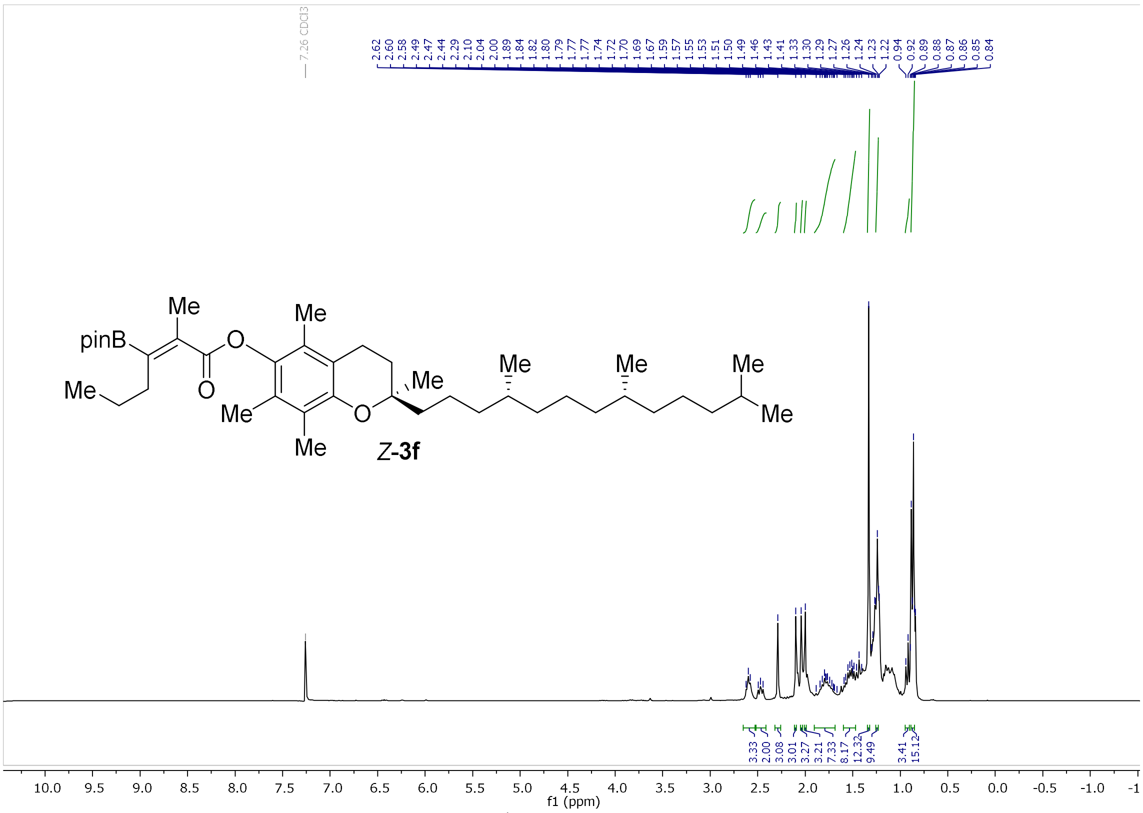

Figure S60. ^{1}H NMR spectrum of *Z*-**3f.**

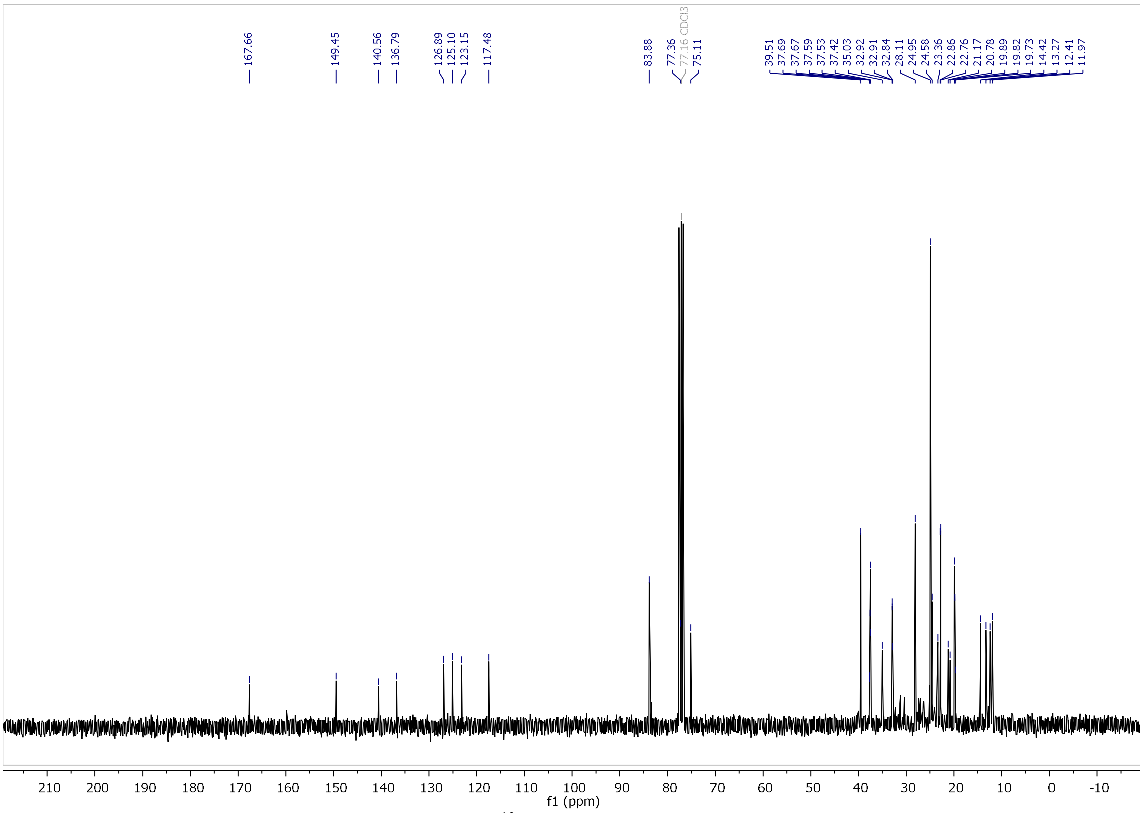

Figure S61. ^{13}C NMR spectrum of *Z*-**3f.**

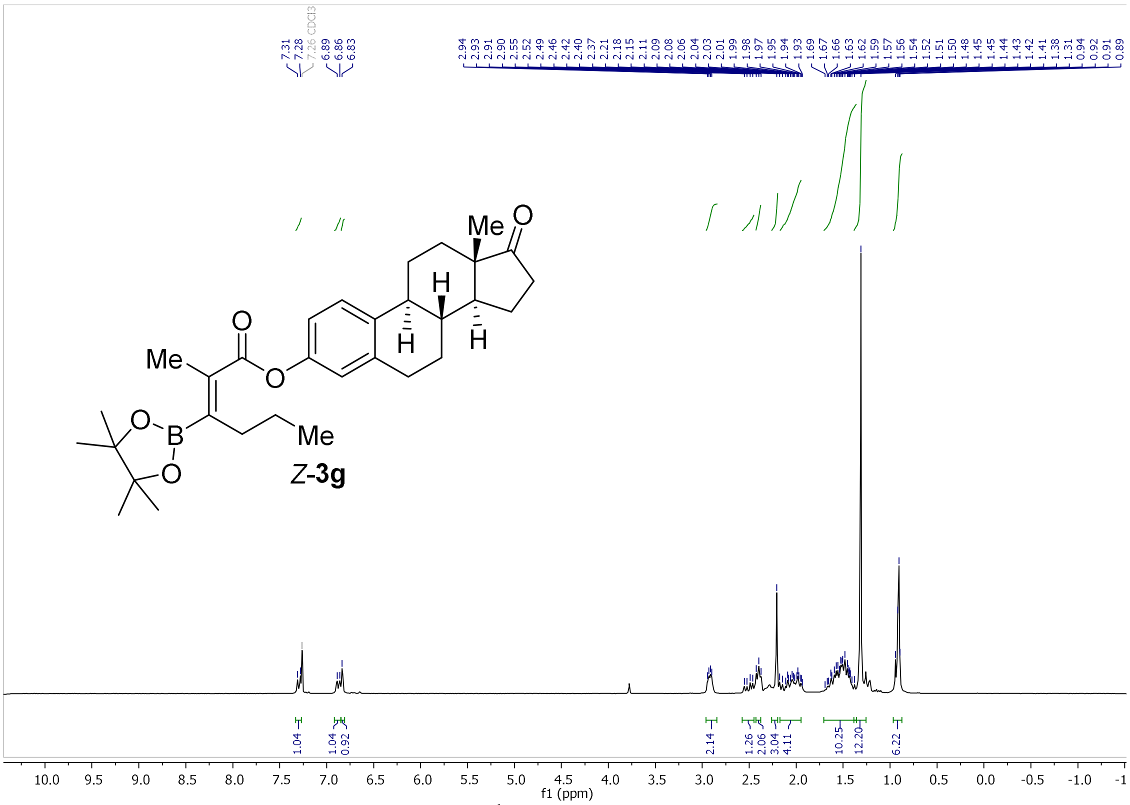

Figure S62. ^{1}H NMR spectrum of *Z*-**3g.**

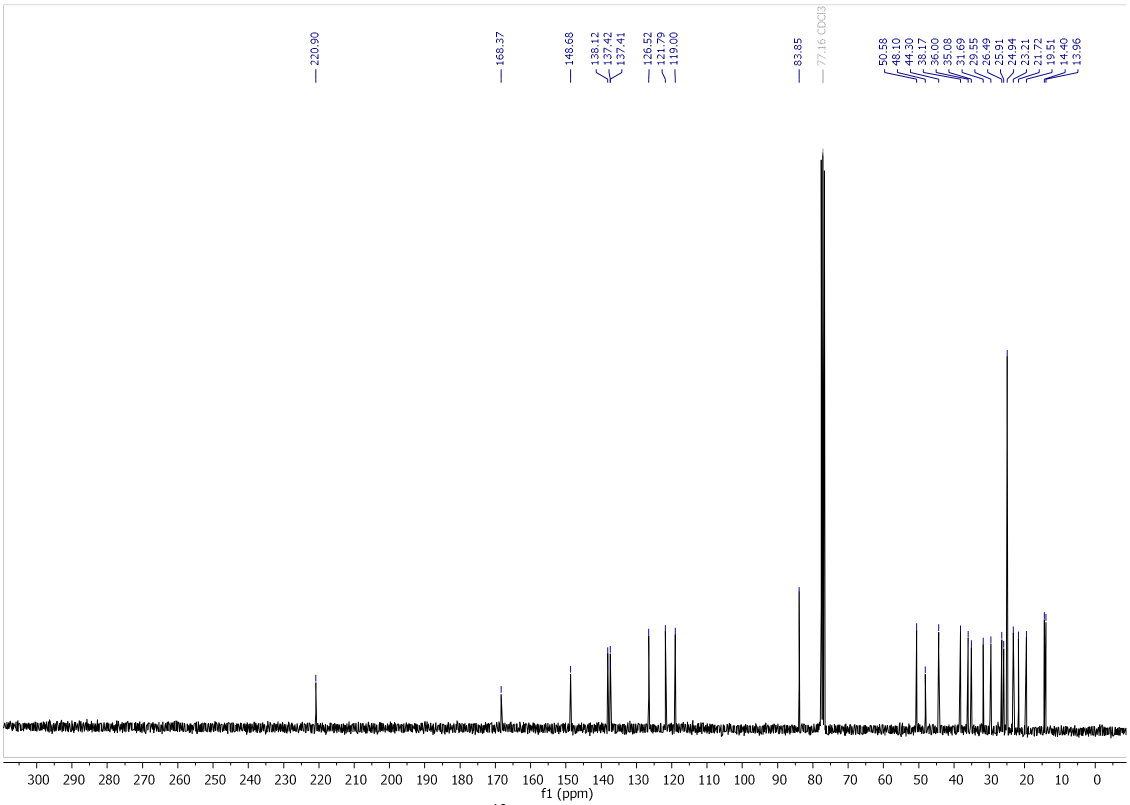

Figure S63. ^{13}C NMR spectrum of *Z*-**3g.**

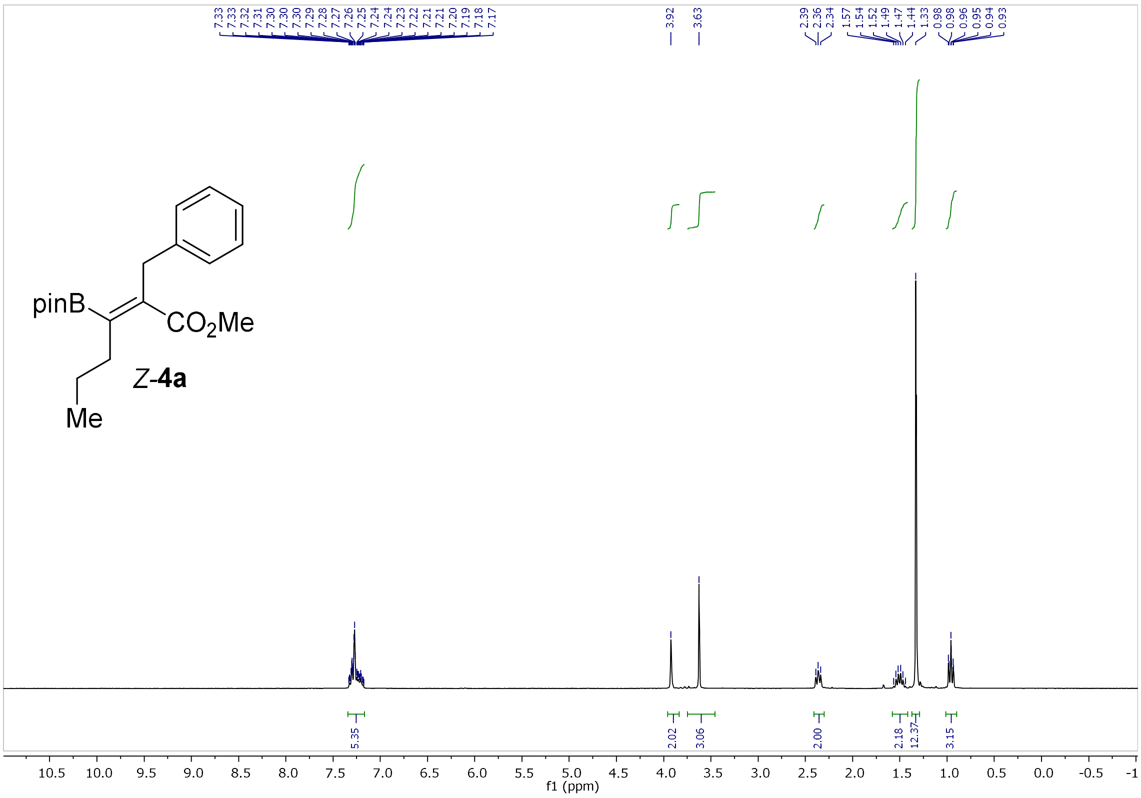

Figure S64. ^{1}H NMR spectrum of *Z*-**4a.**

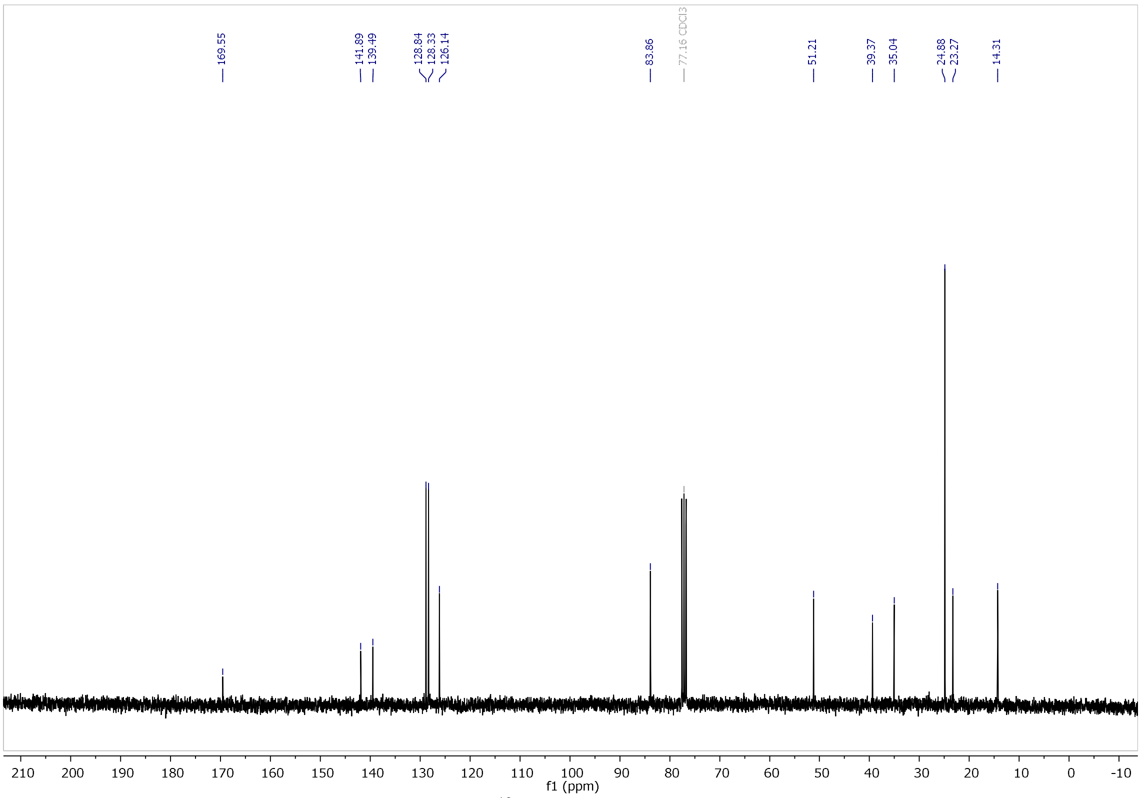

Figure S65. ^{13}C NMR spectrum of *Z*-**4a.**

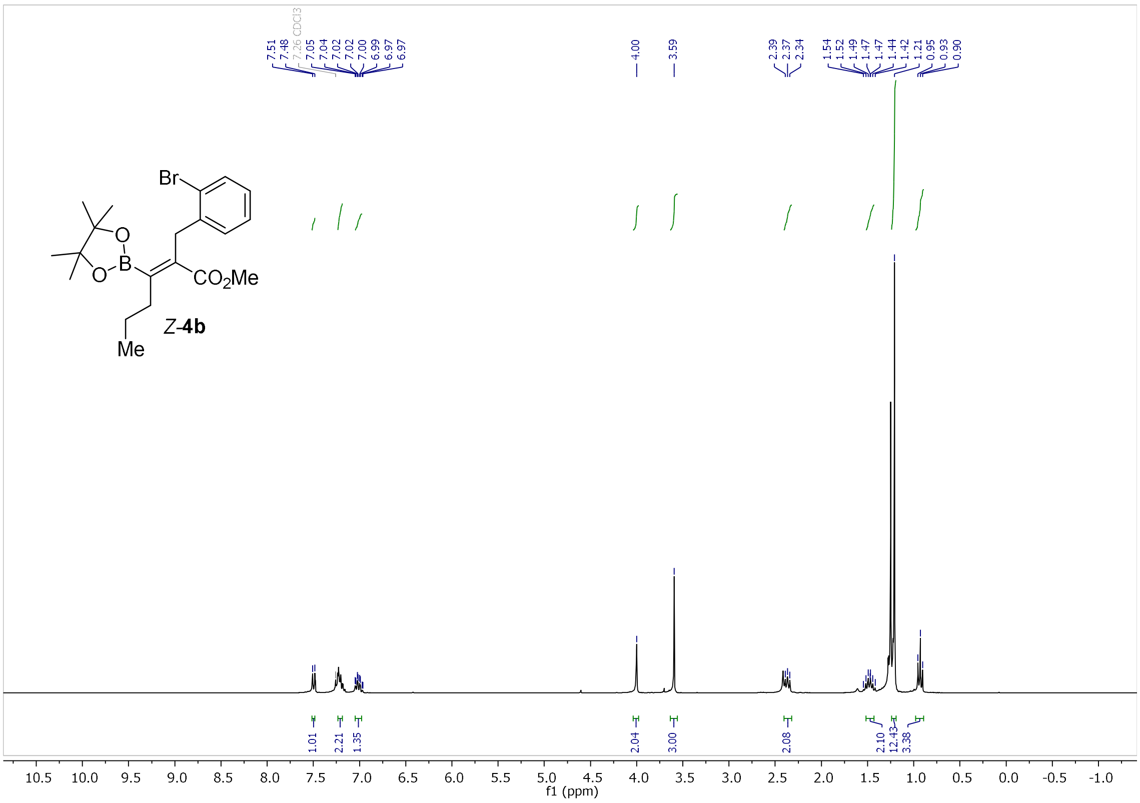

Figure S66. ^{1}H NMR spectrum of *Z*-**4b.**

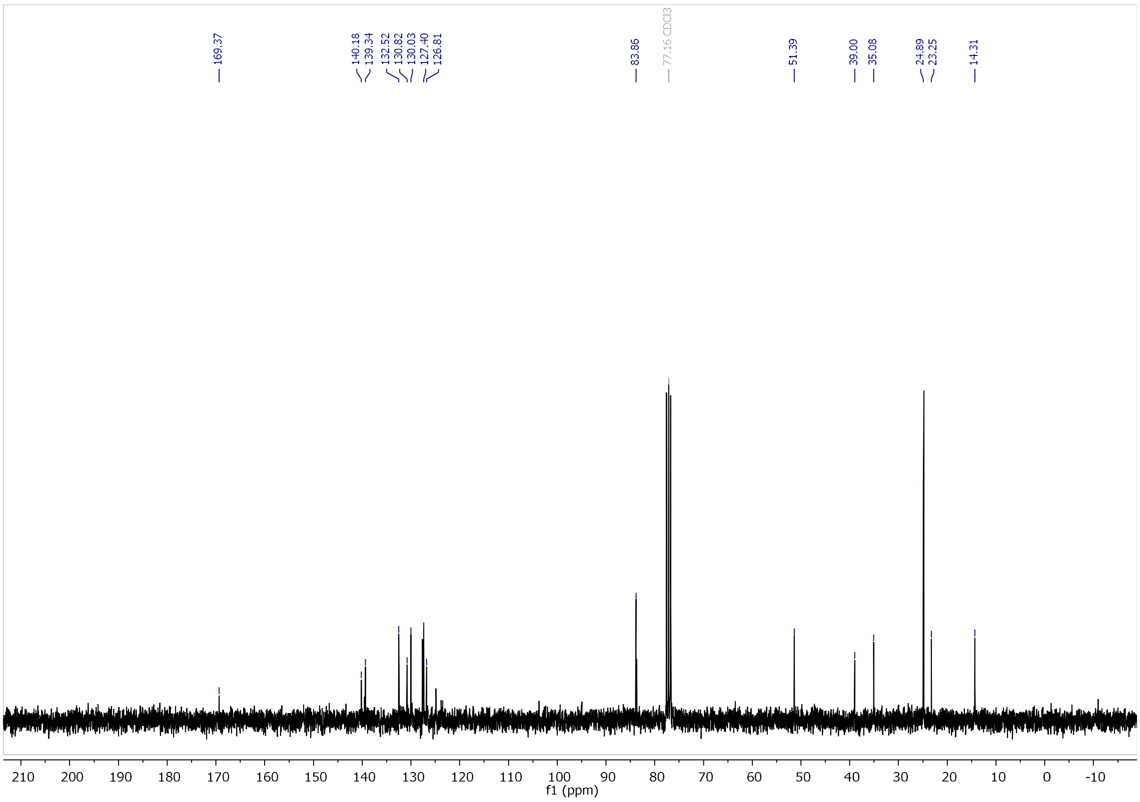

Figure S67. ^{13}C NMR spectrum of *Z*-**4b.**

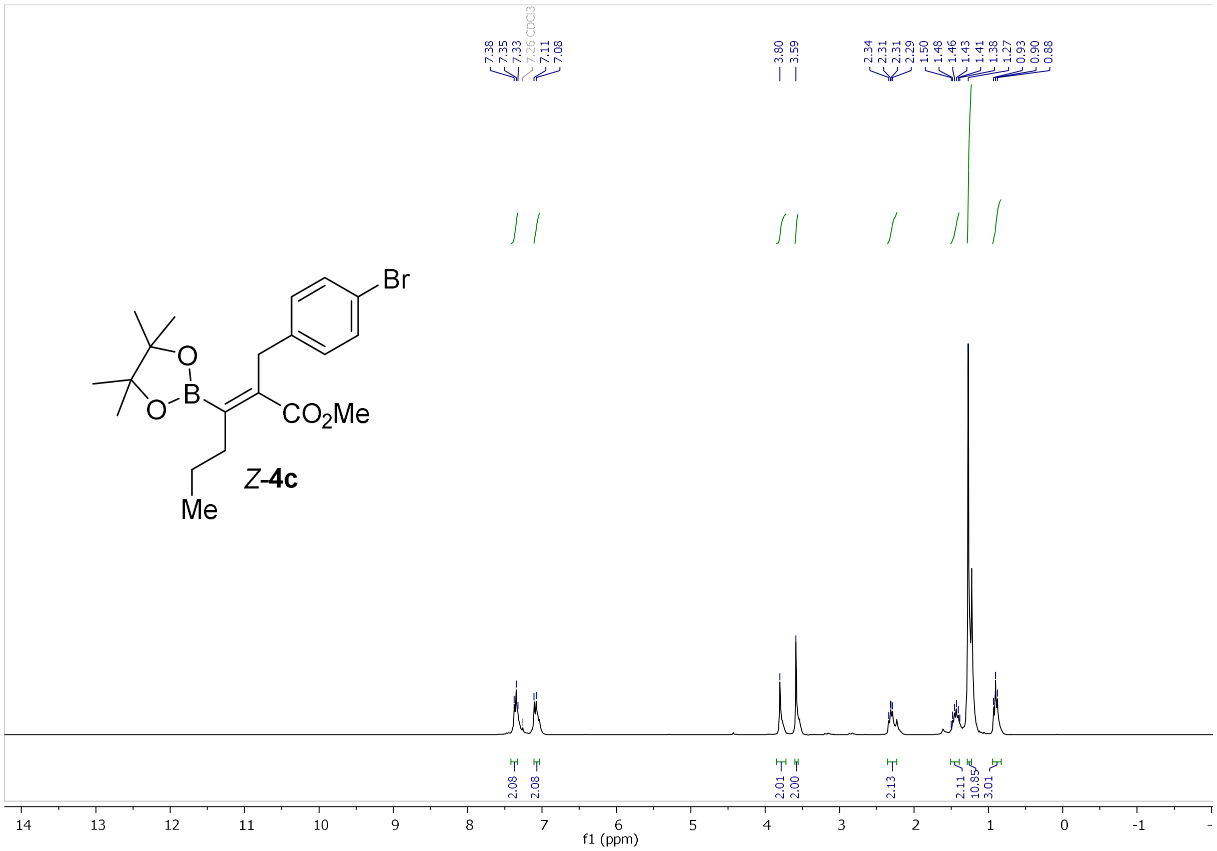

Figure S68. ^{1}H NMR spectrum of *Z*-**4c.**

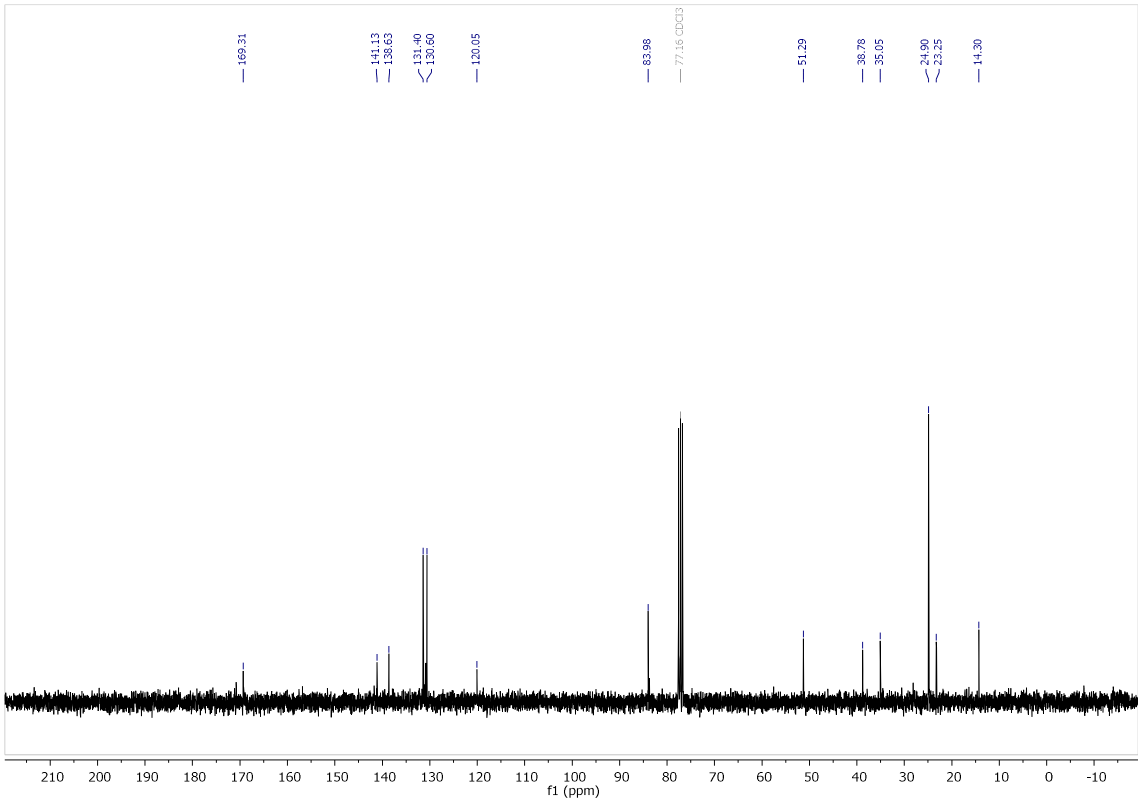

Figure S69. ^{13}C NMR spectrum of *Z*-**4c.**

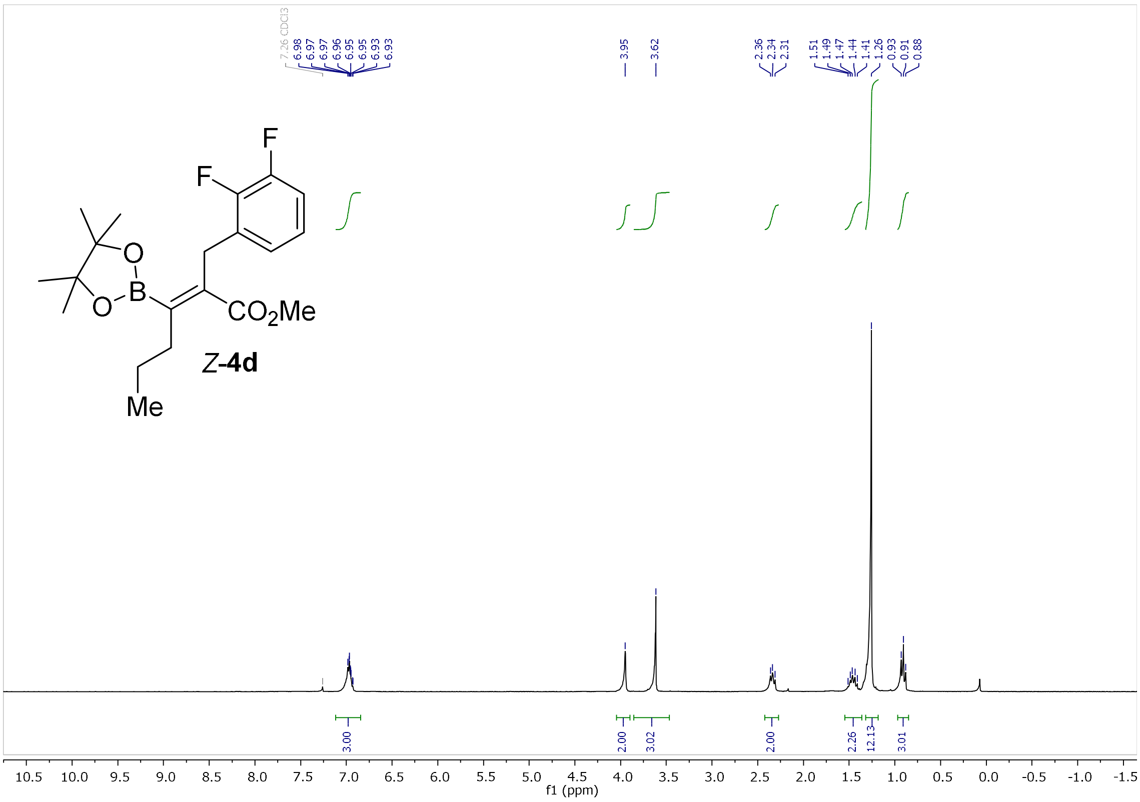

Figure S70. ^{1}H NMR spectrum of *Z*-**4d.**

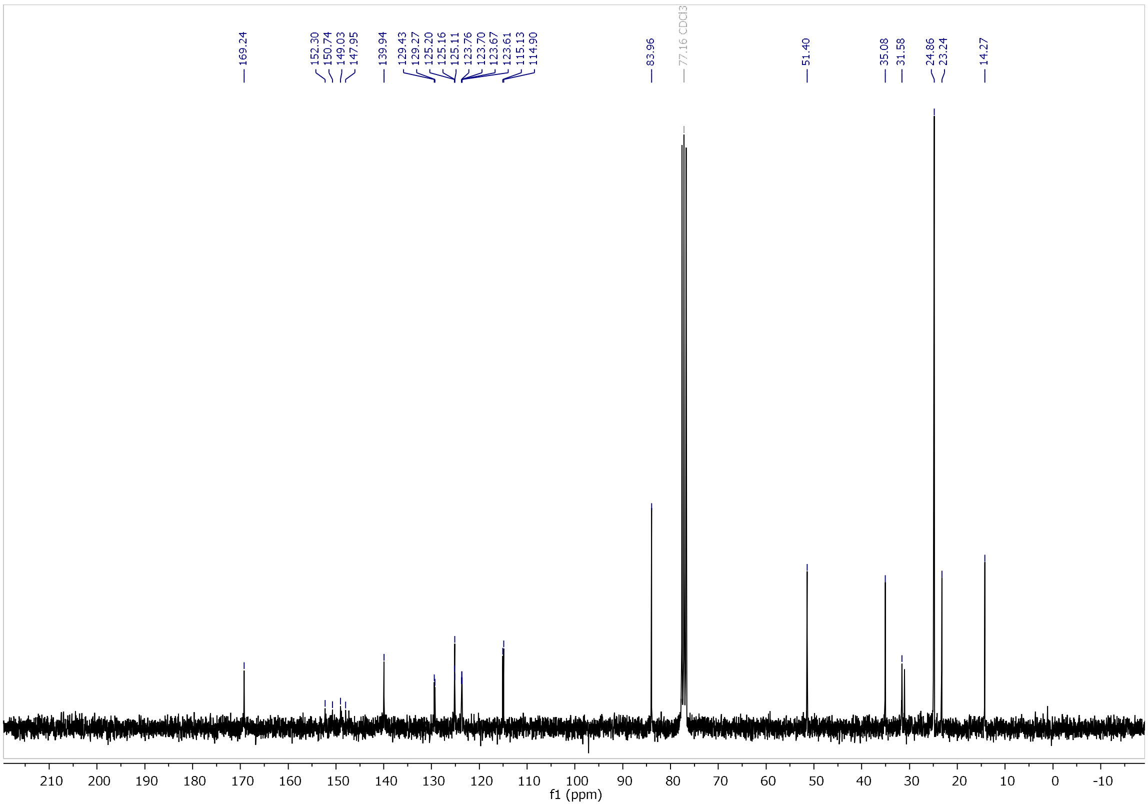

Figure S71. ^{13}C NMR spectrum of *Z*-**4d.**

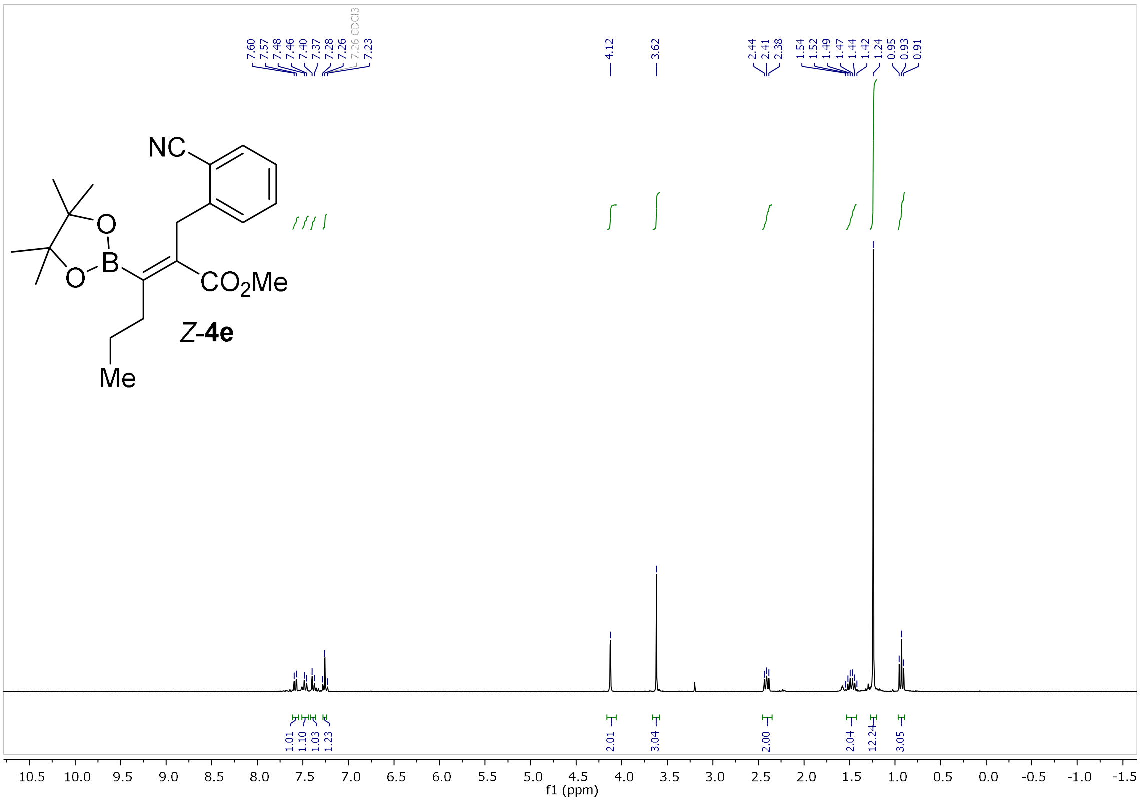

Figure S72. ^{1}H NMR spectrum of *Z*-**4e.**

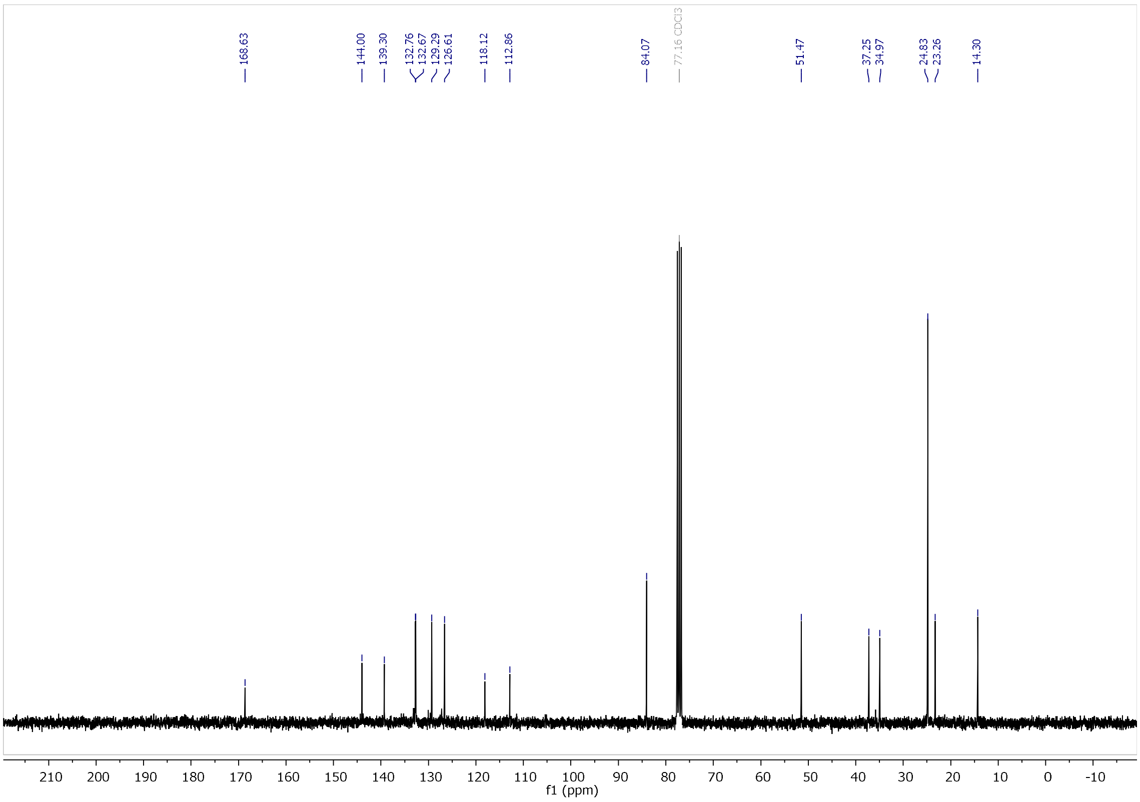

Figure S73. ^{13}C NMR spectrum of *Z*-**4e.**

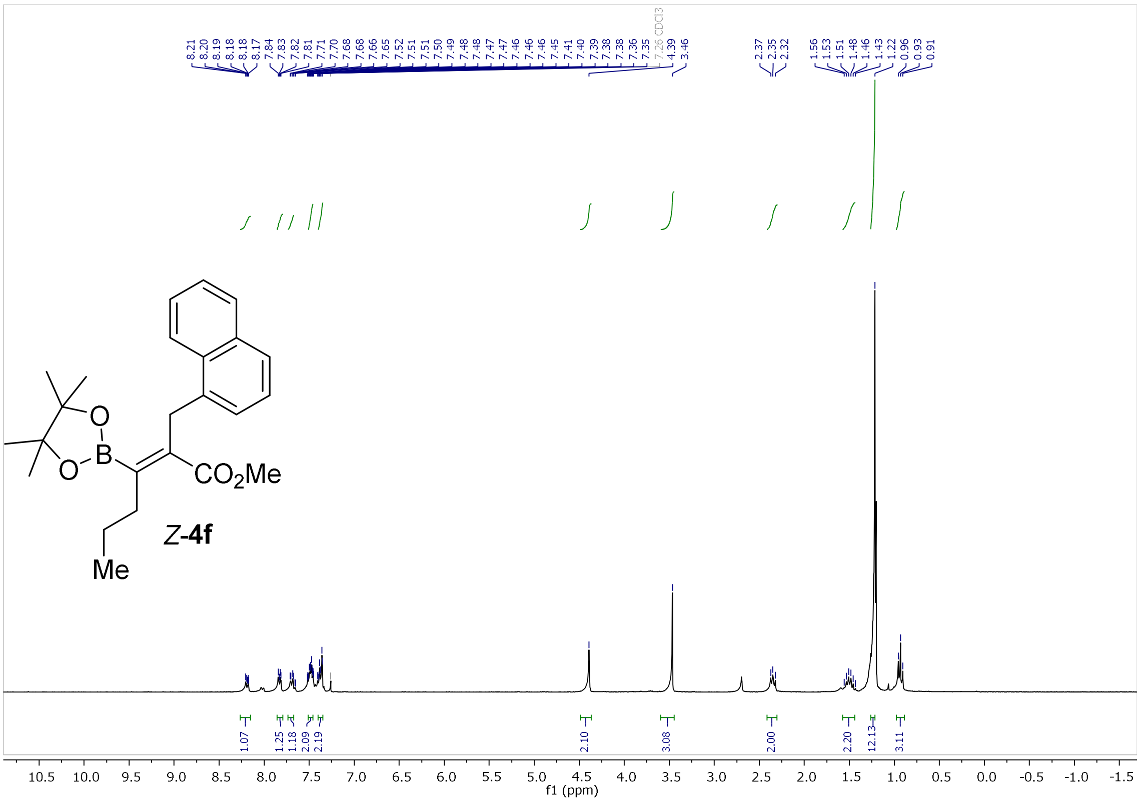

Figure S74. ^{1}H NMR spectrum of *Z*-**4f.**

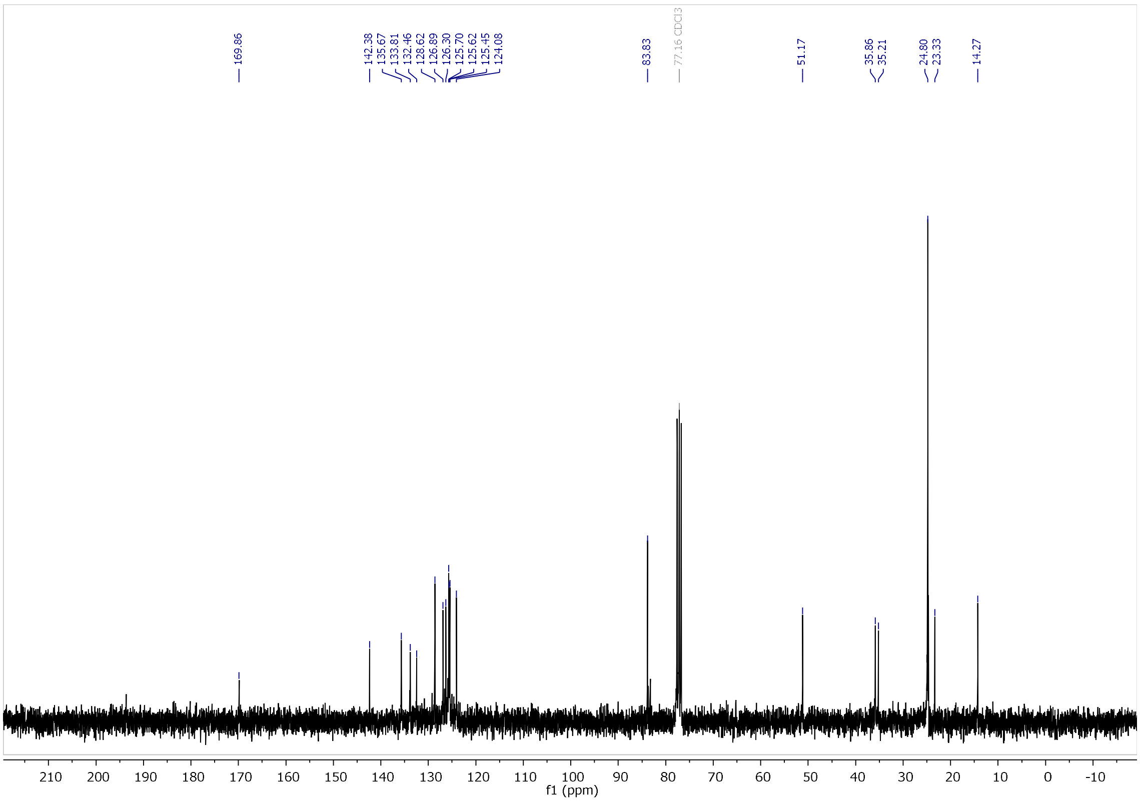

Figure S75. ^{13}C NMR spectrum of *Z*-**4f.**

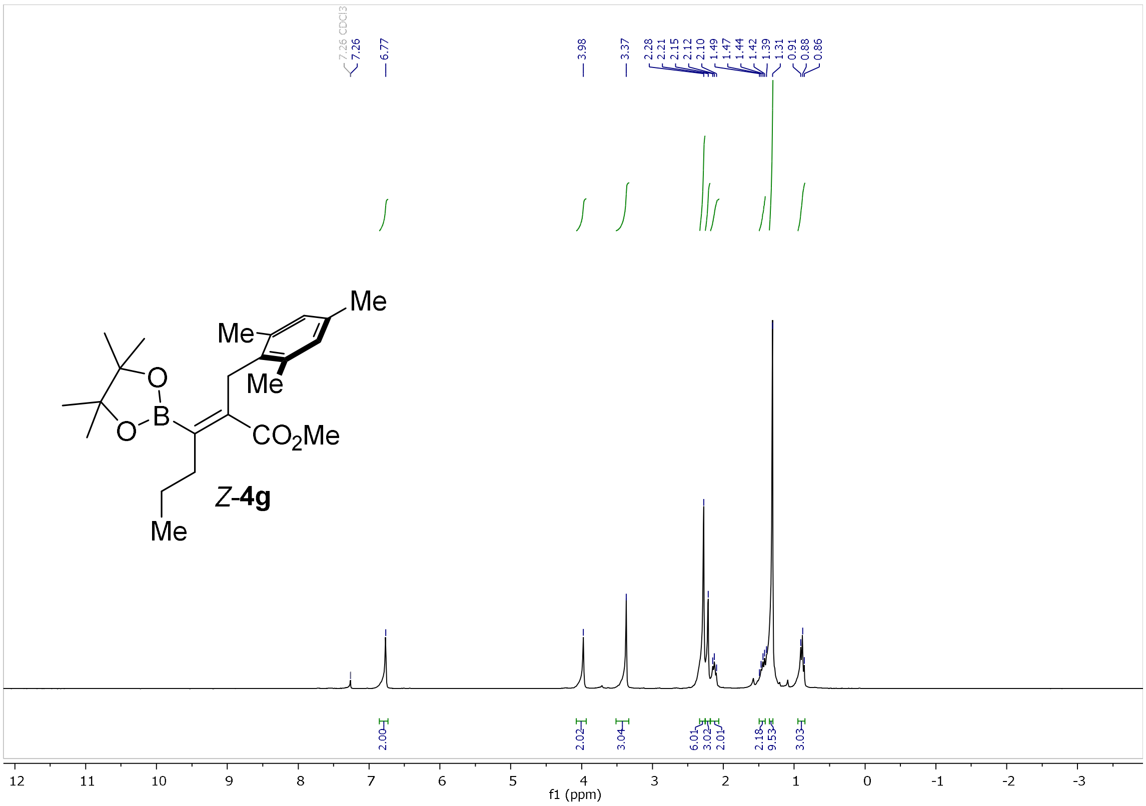

Figure S76. ^{1}H NMR spectrum of *Z*-**4g.**

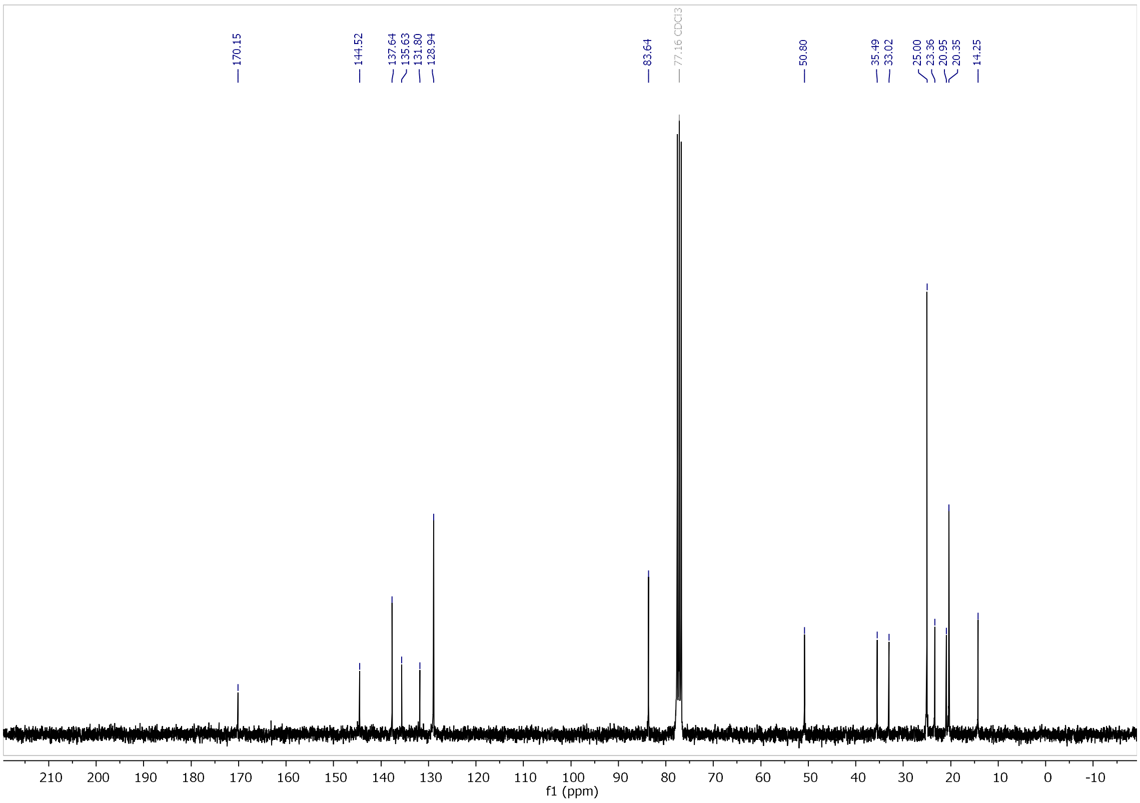

Figure S77. ^{13}C NMR spectrum of *Z*-**4g.**

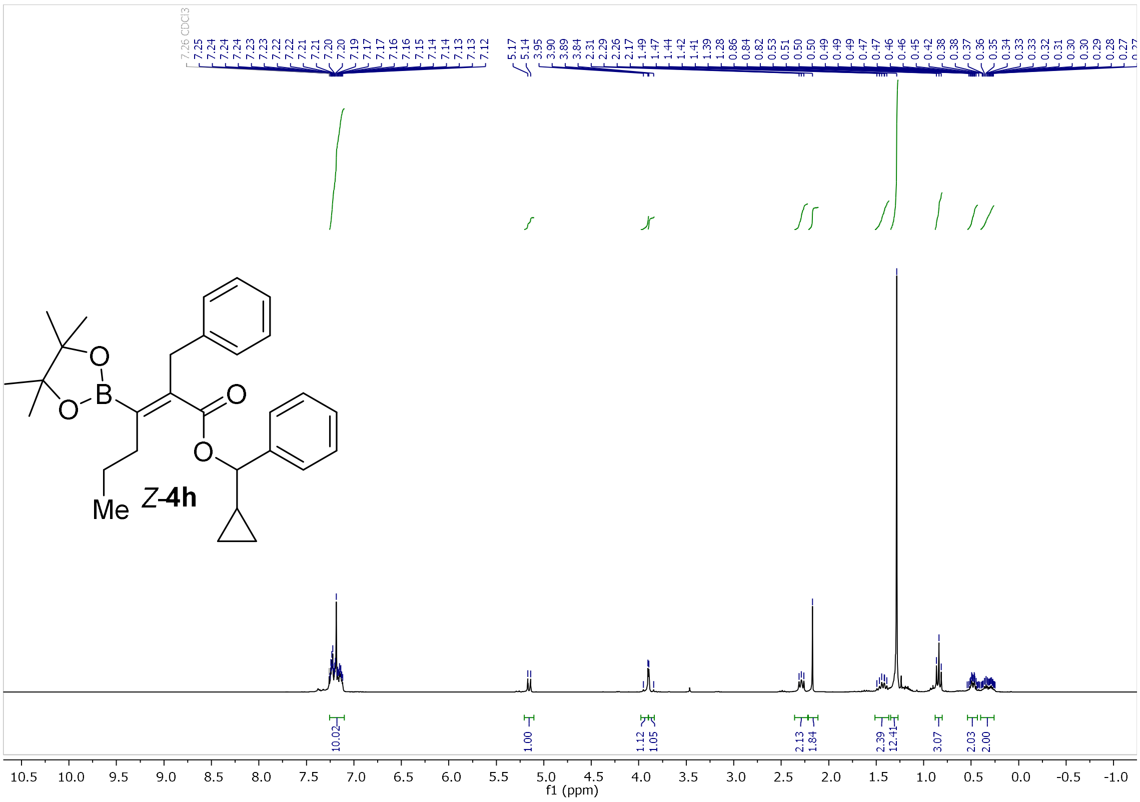

Figure S78. ^{1}H NMR spectrum of *Z*-**4h.**

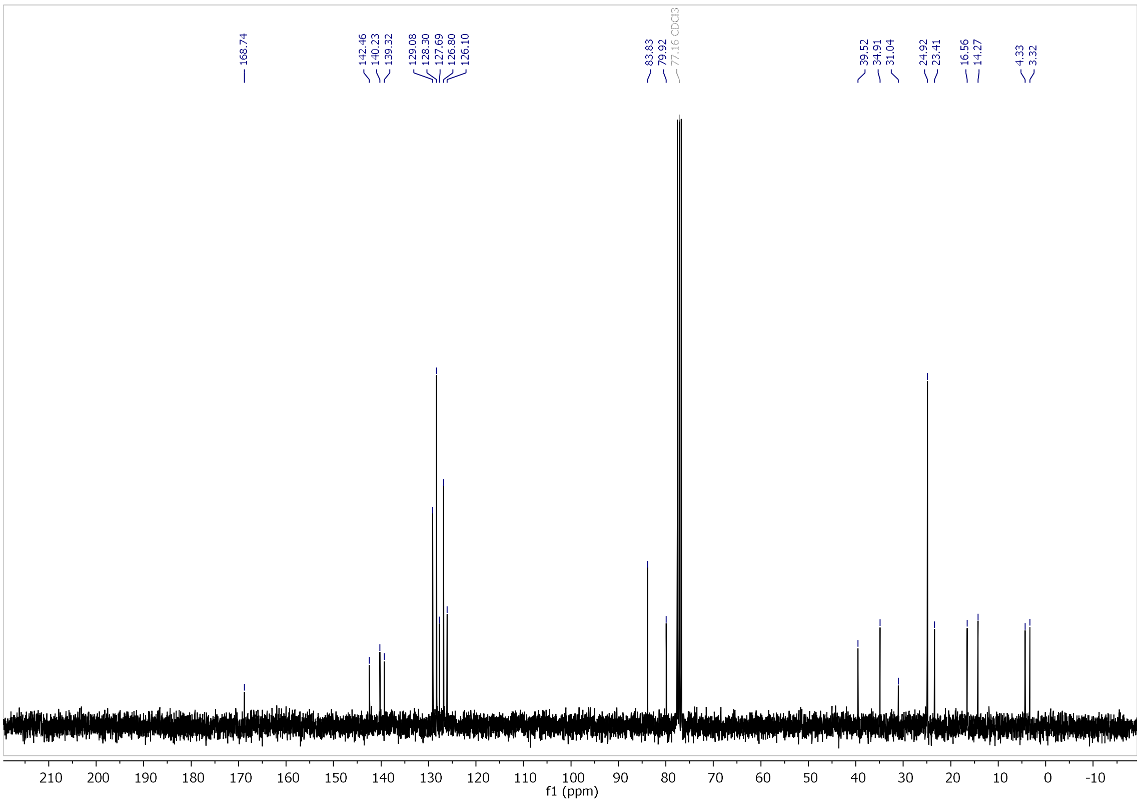

Figure S79. ^{13}C NMR spectrum of *Z*-**4h.**

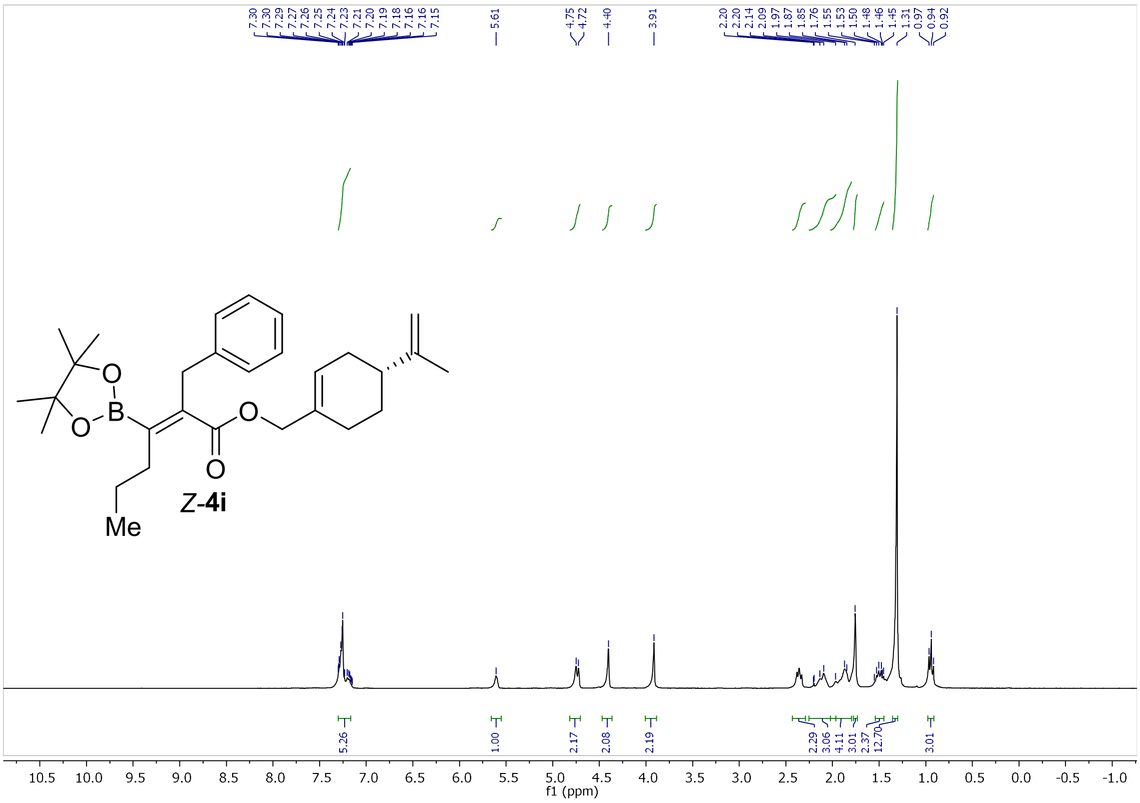

Figure S80. ^{1}H NMR spectrum of *Z*-**4i.**

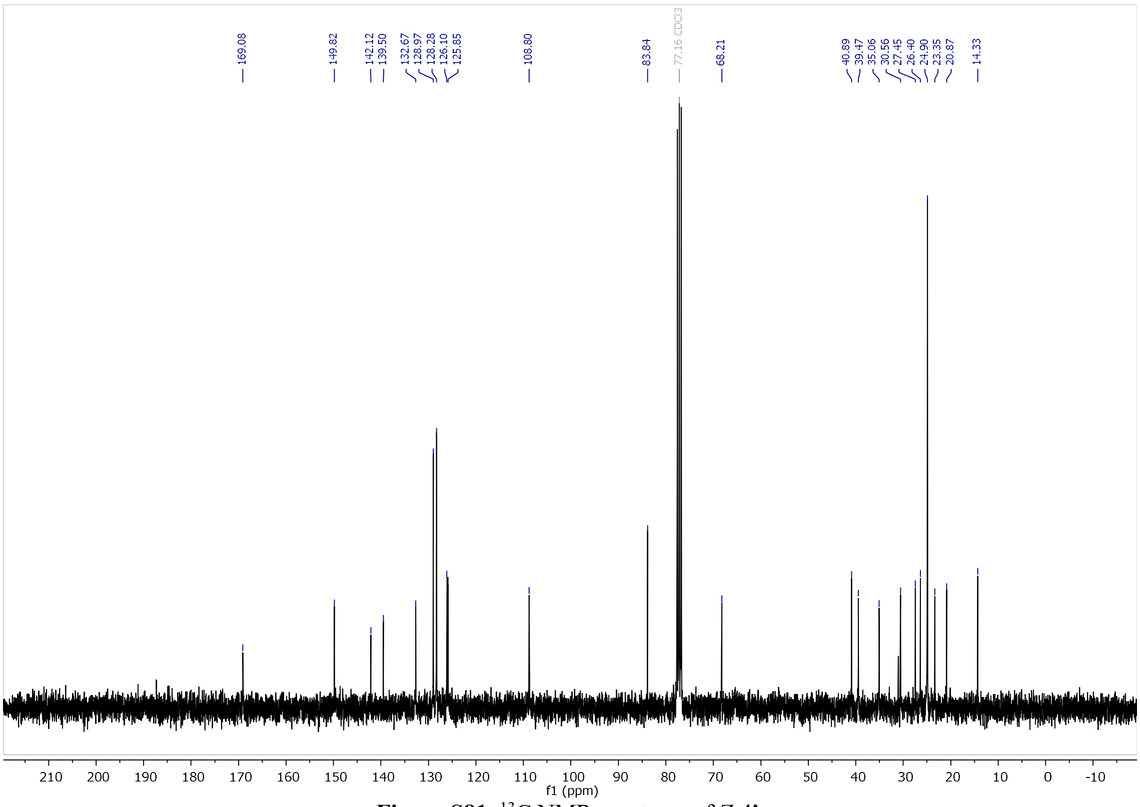

Figure S81. ^{13}C NMR spectrum of *Z*-**4i.**

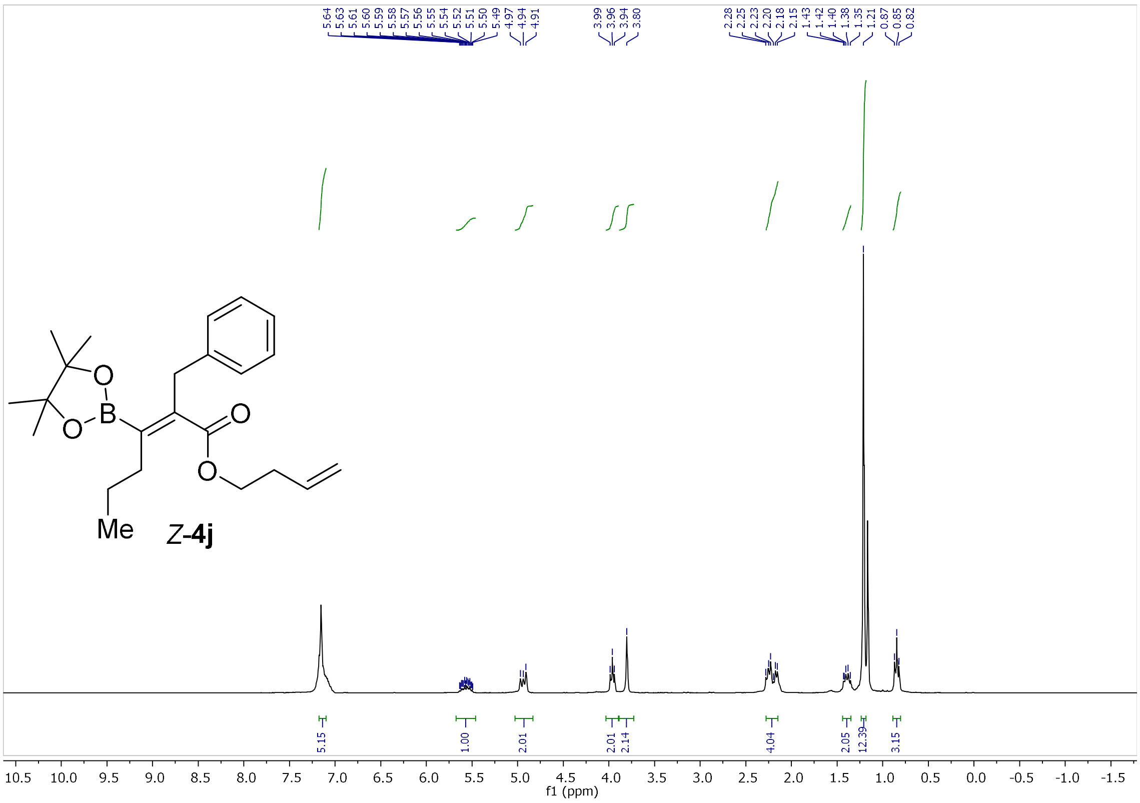

Figure S82. ^{1}H NMR spectrum of *Z*-**4j.**

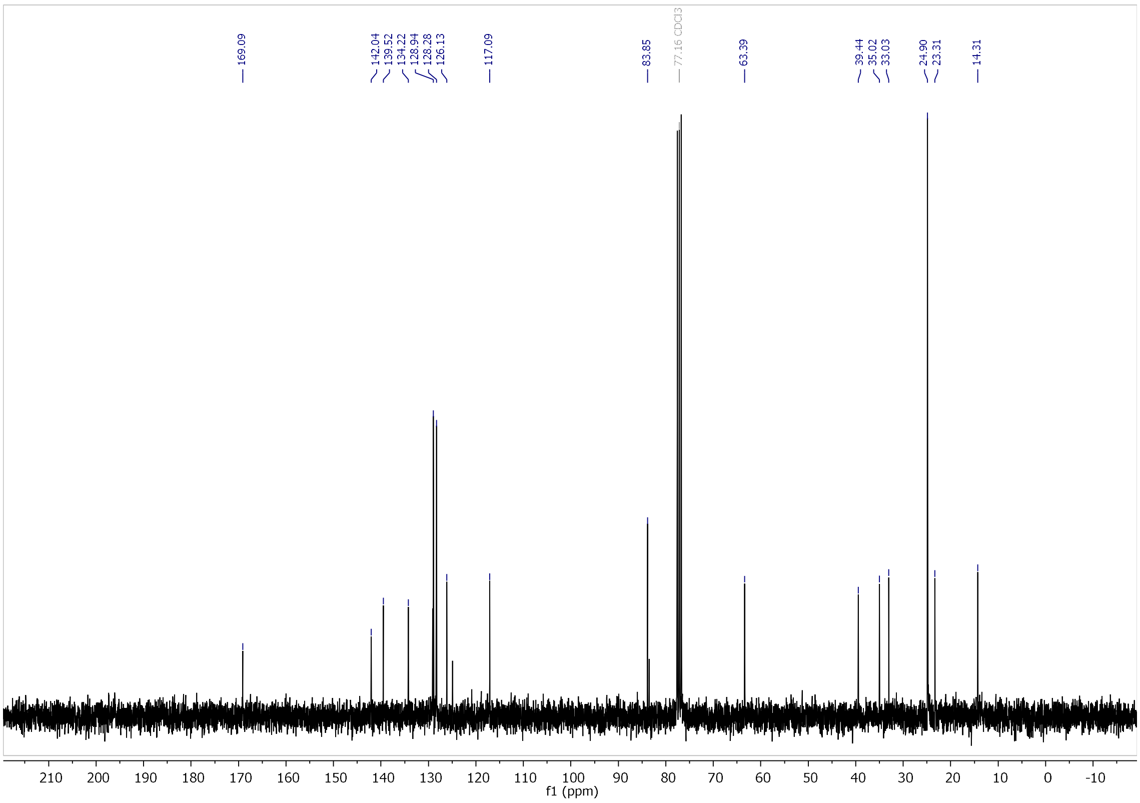

Figure S83. ^{13}C NMR spectrum of *Z*-**4j.**

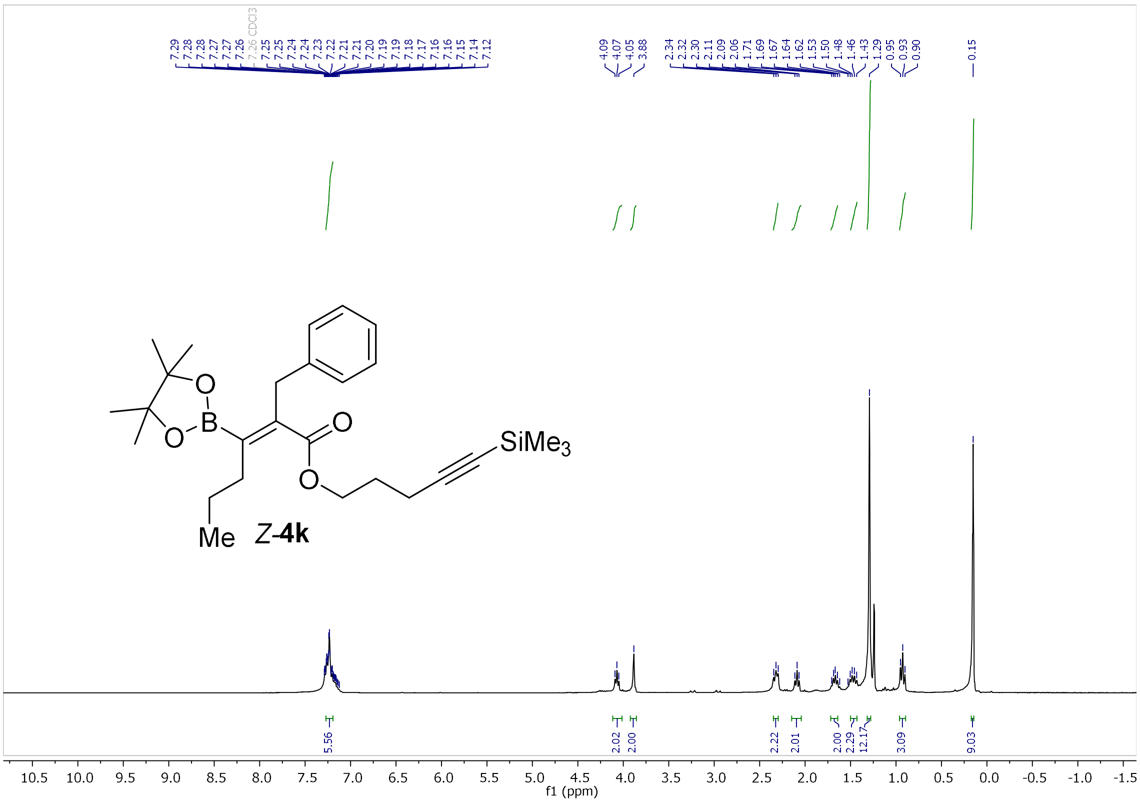

Figure S84. ¹H NMR spectrum of *Z*-**4k.**

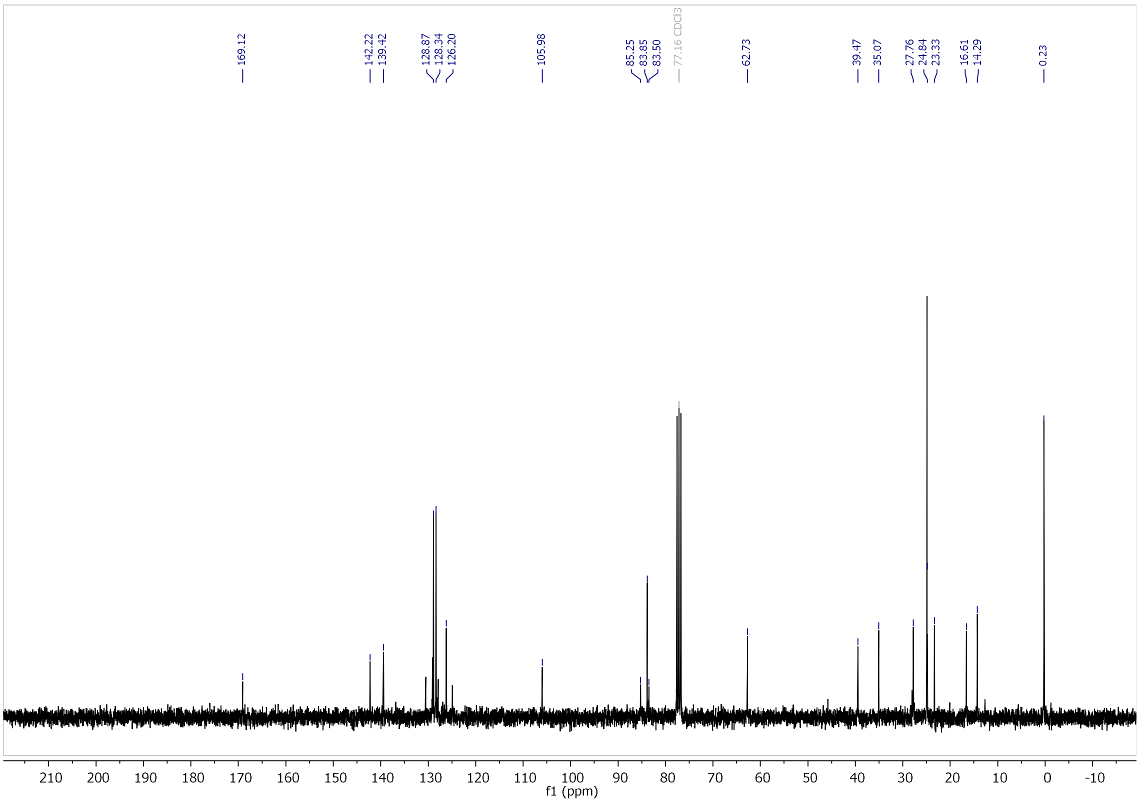

Figure S85. ¹³C NMR spectrum of *Z*-**4k.**

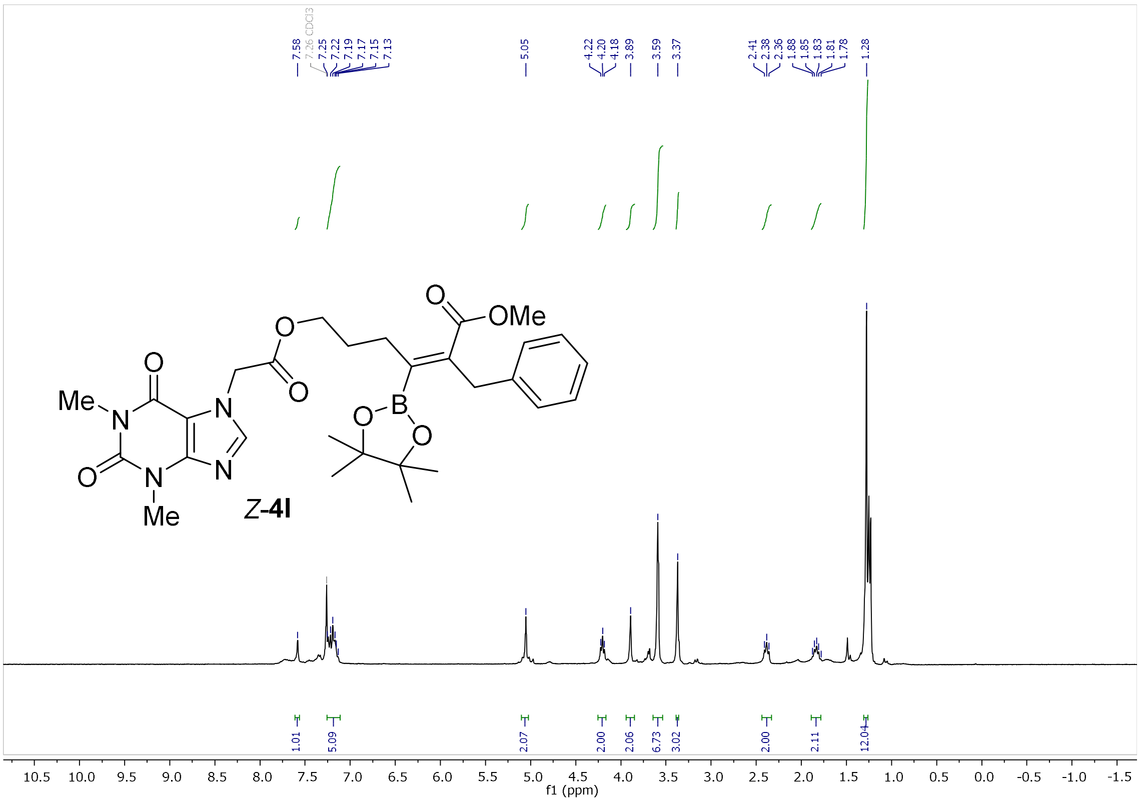

Figure S86. ^{1}H NMR spectrum of *Z*-**4l.**

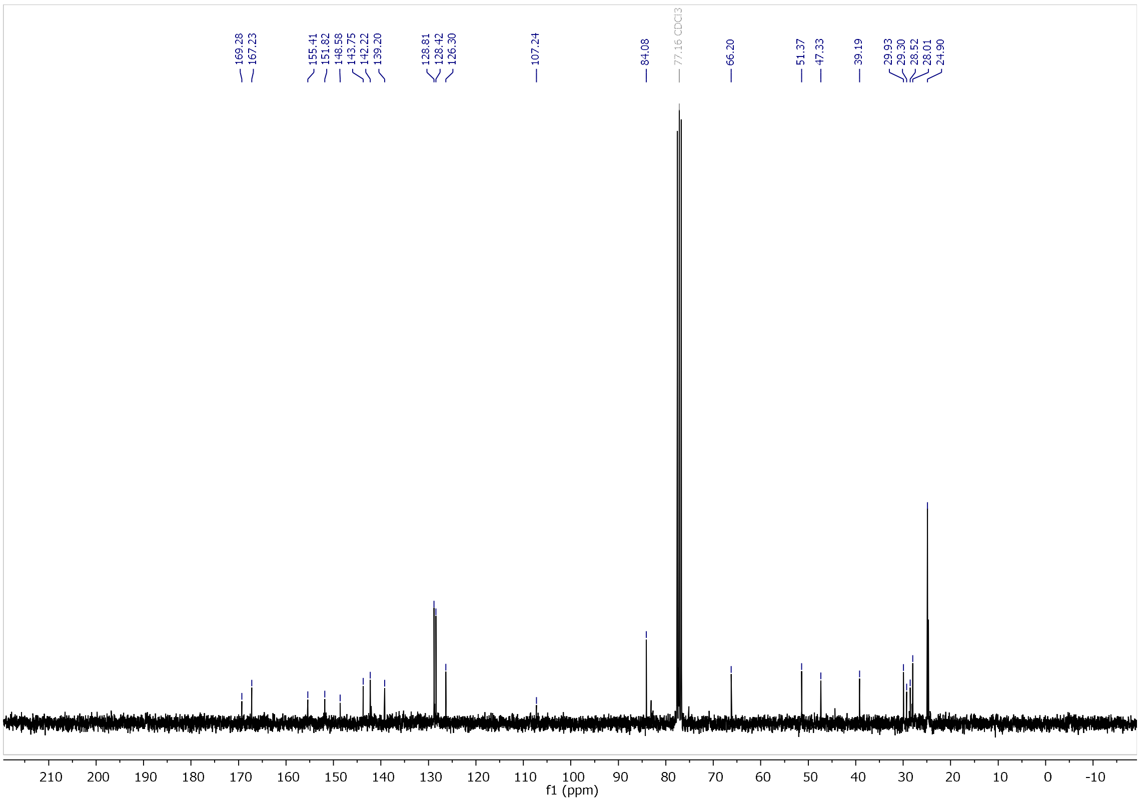

Figure S87. ^{13}C NMR spectrum of *Z*-**4l.**

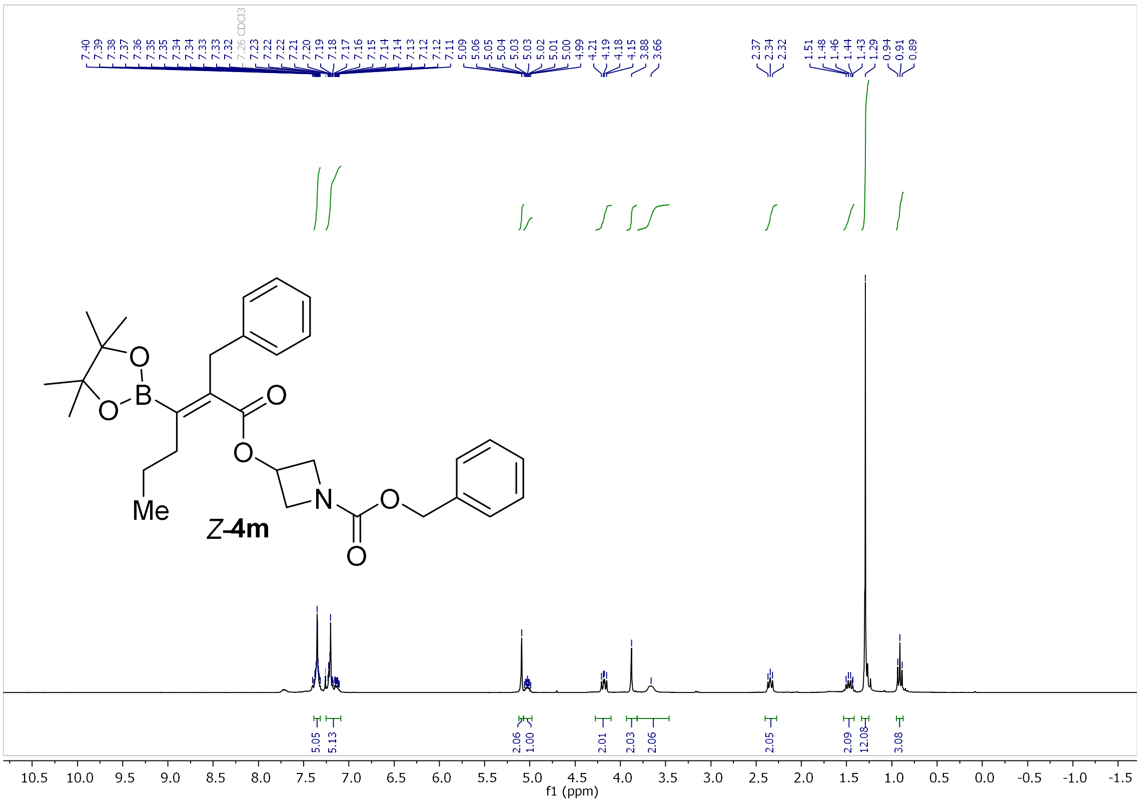

Figure S88. ^{1}H NMR spectrum of *Z*-**4m.**

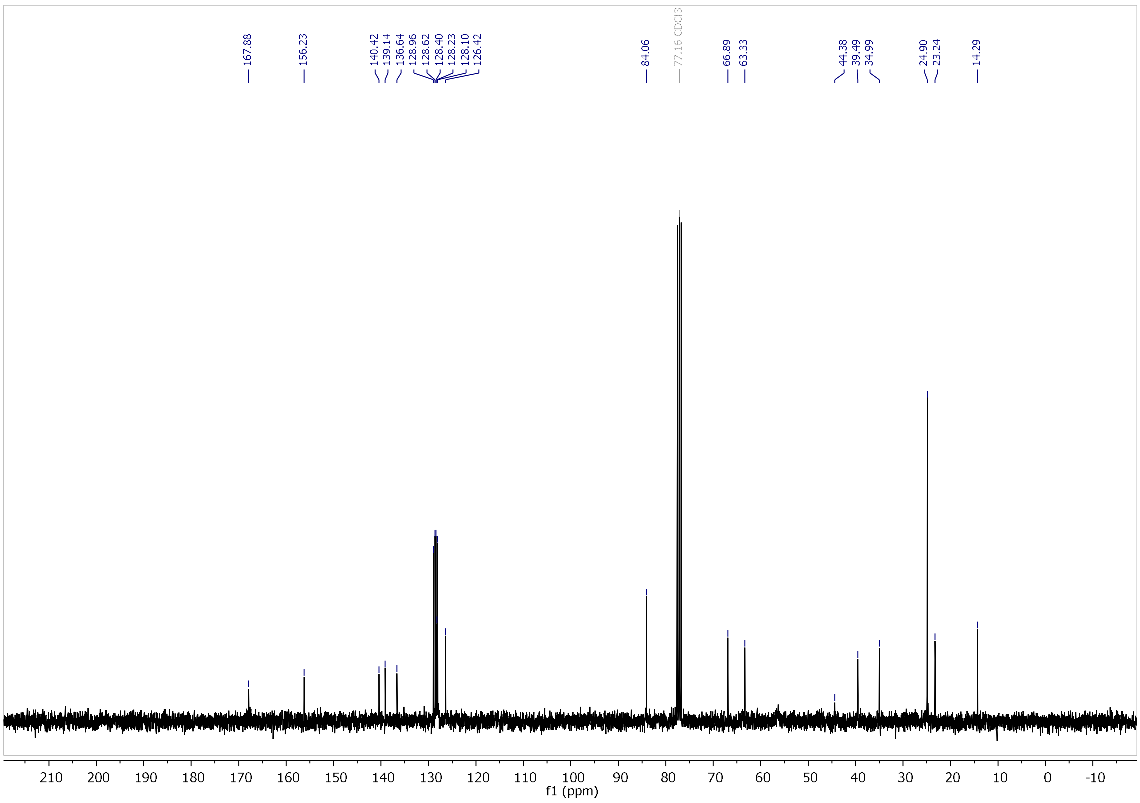

Figure S89. ^{13}C NMR spectrum of *Z*-**4m.**

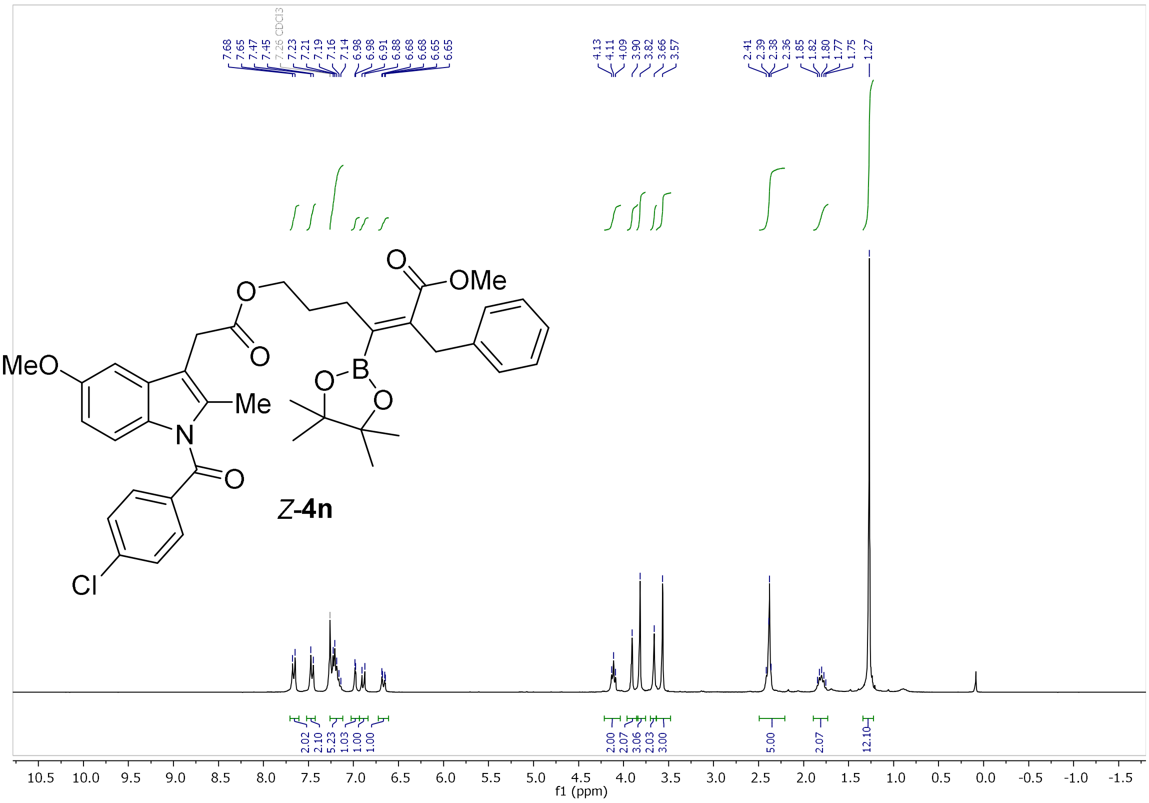

Figure S90. ^{1}H NMR spectrum of *Z*-**4n.**

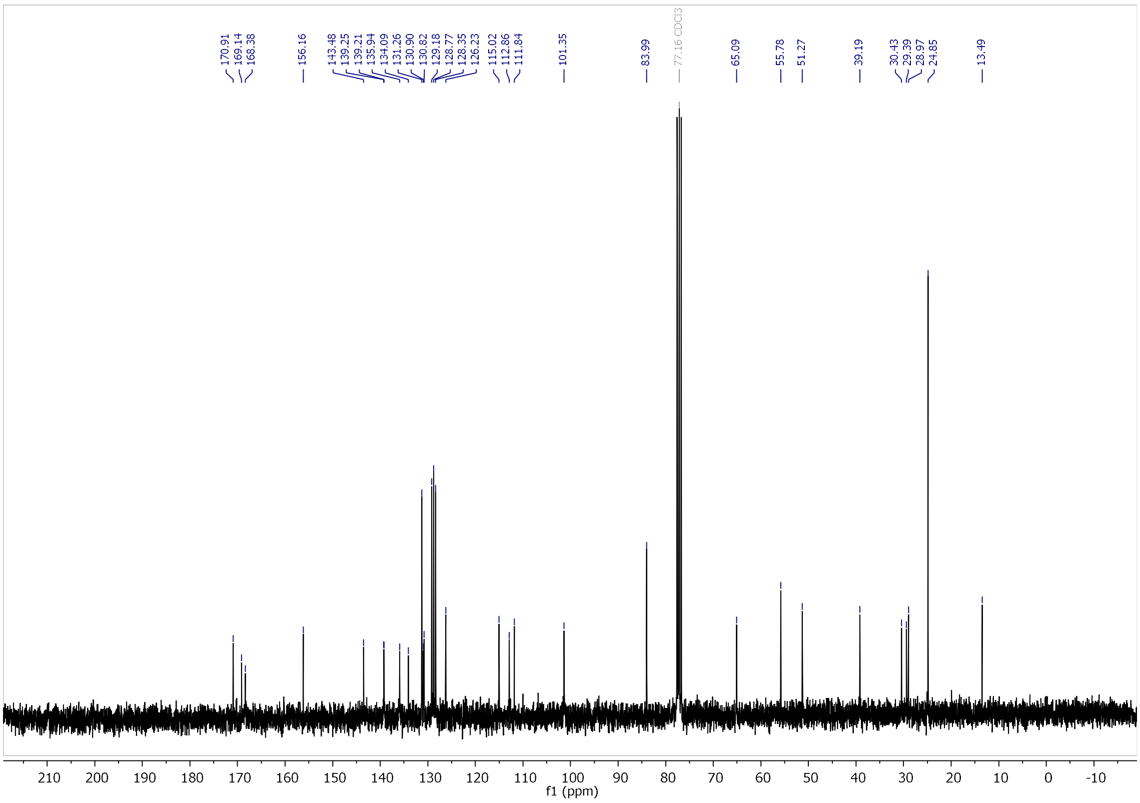

Figure S91. ^{13}C NMR spectrum of *Z*-**4n.**

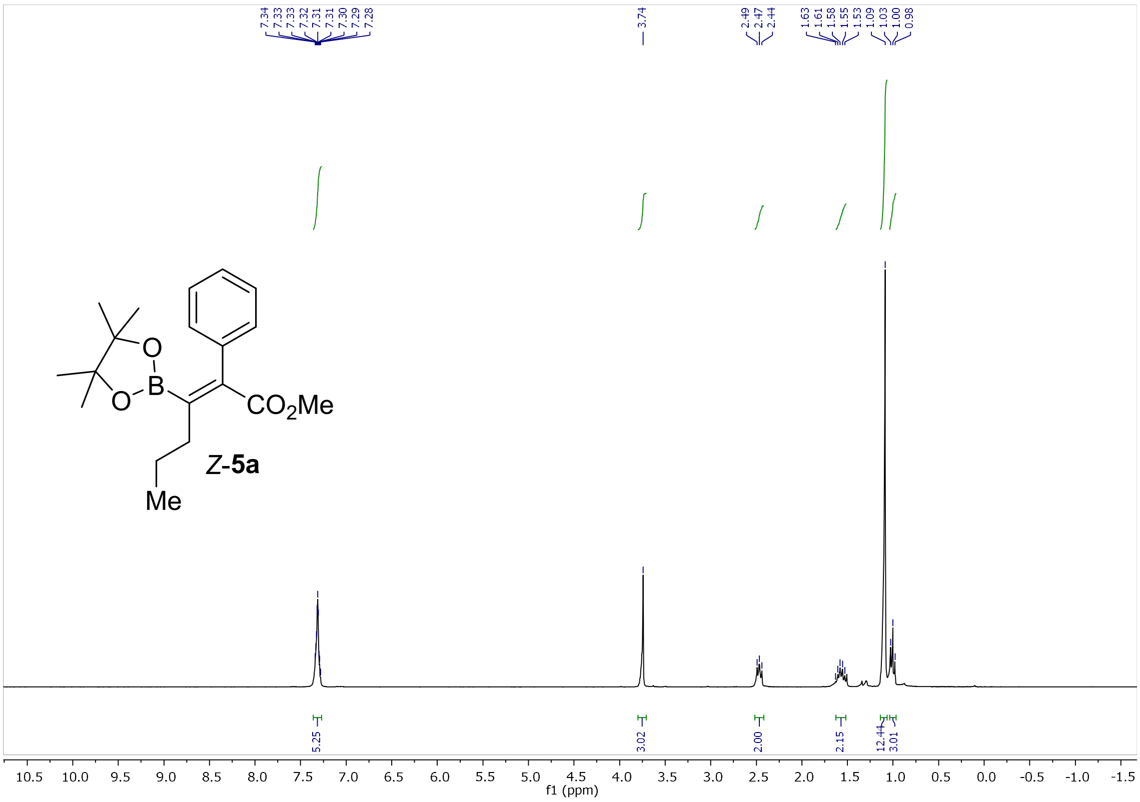

Figure S92. ^{1}H NMR spectrum of *Z*-**5a.**

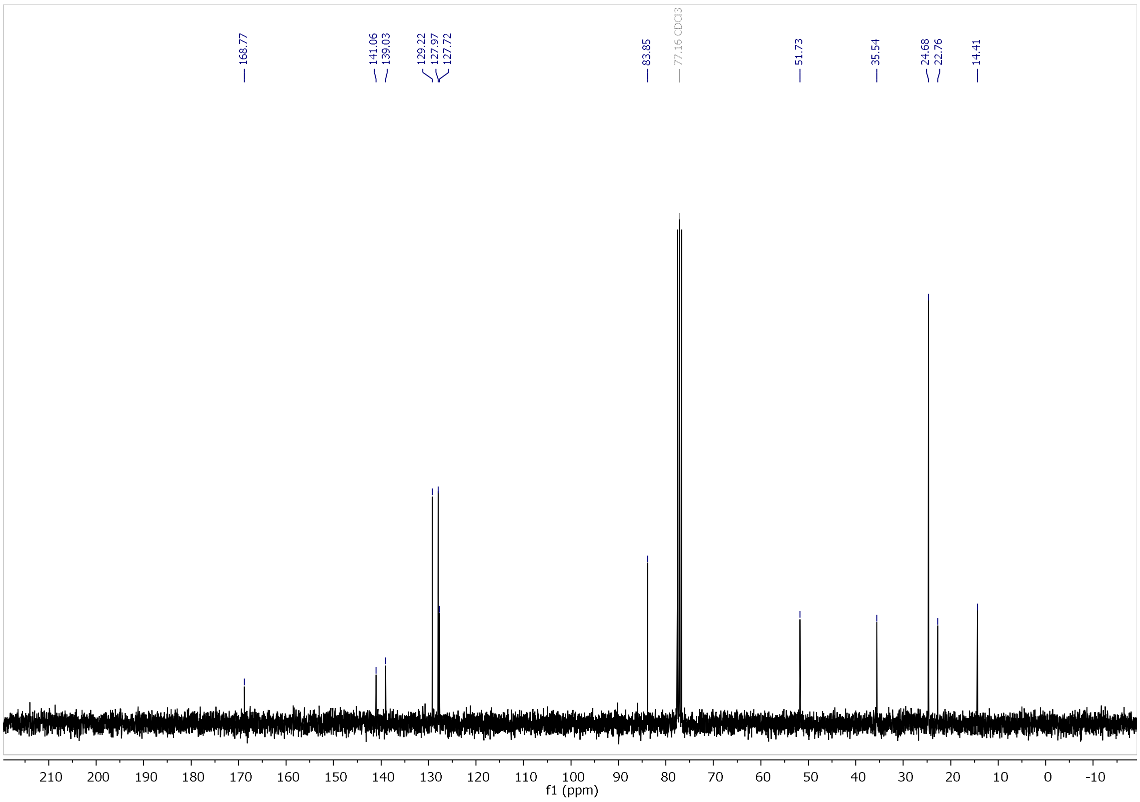

Figure S93. ^{13}C NMR spectrum of *Z*-**5a.**

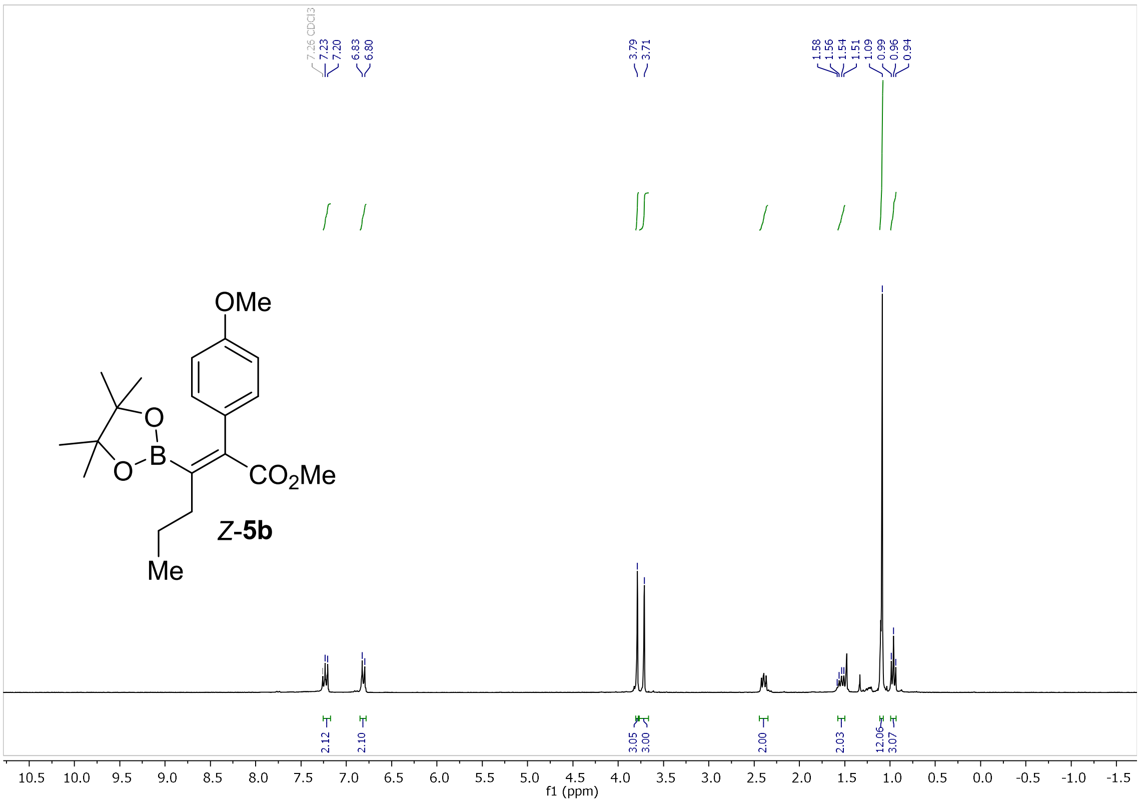

Figure S94. ^{1}H NMR spectrum of *Z*-**5b.**

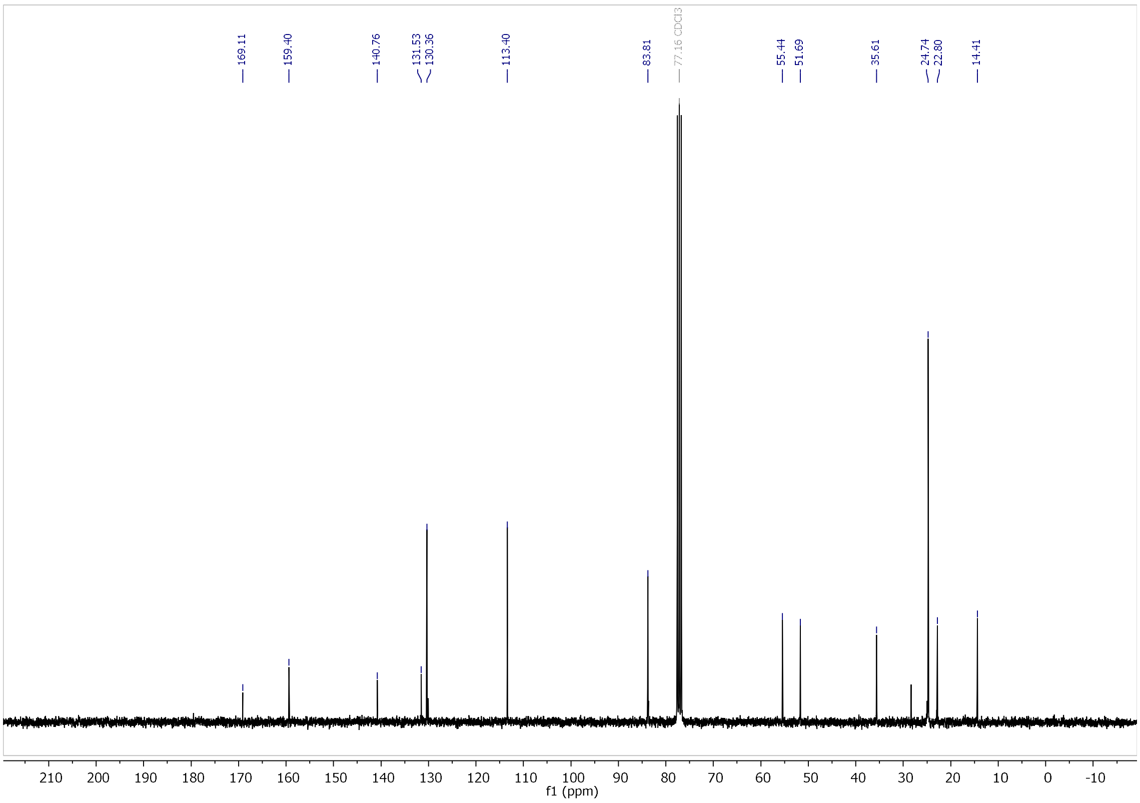

Figure S95. ^{13}C NMR spectrum of *Z*-**5b.**

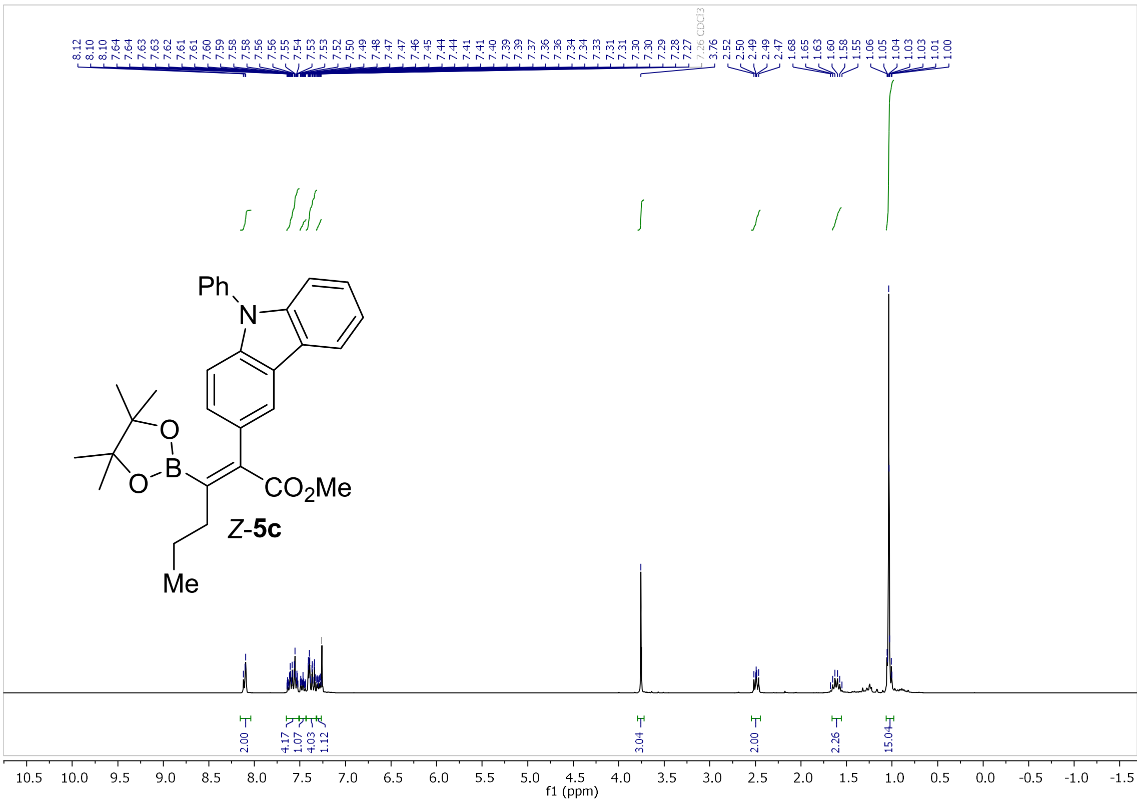

Figure S96. ^{1}H NMR spectrum of *Z*-**5c.**

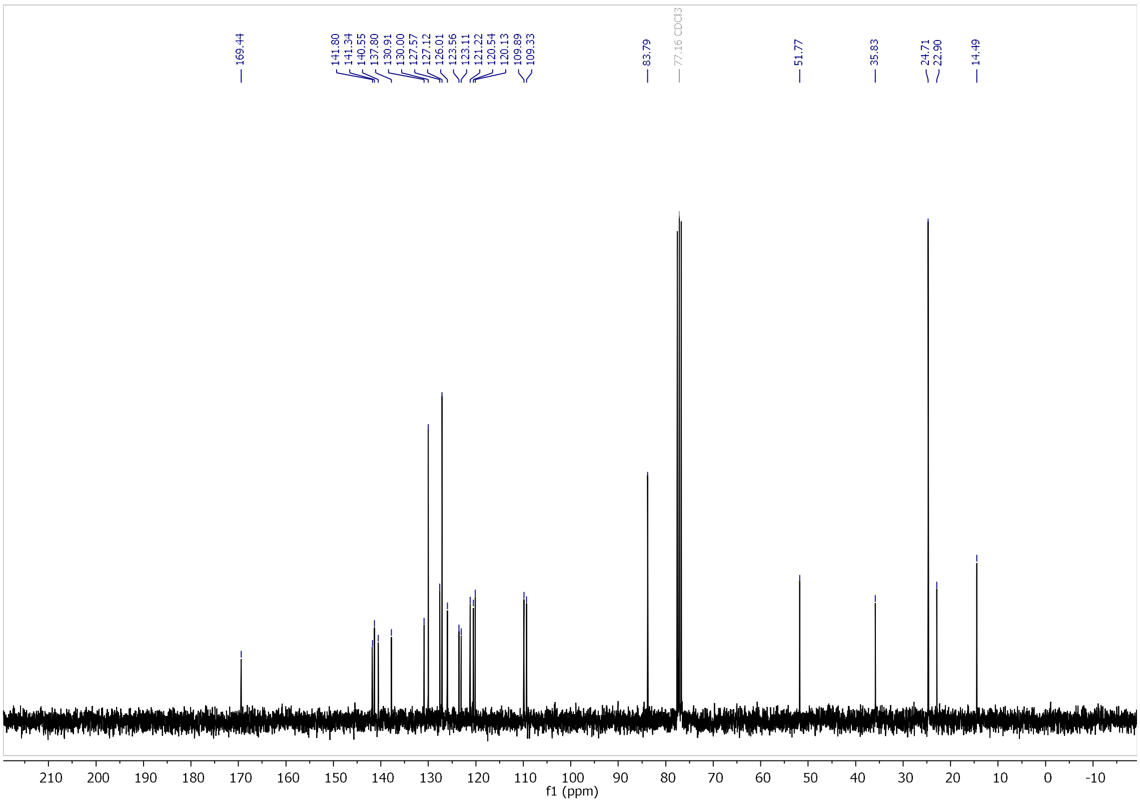

Figure S97. ^{13}C NMR spectrum of *Z*-**5c.**

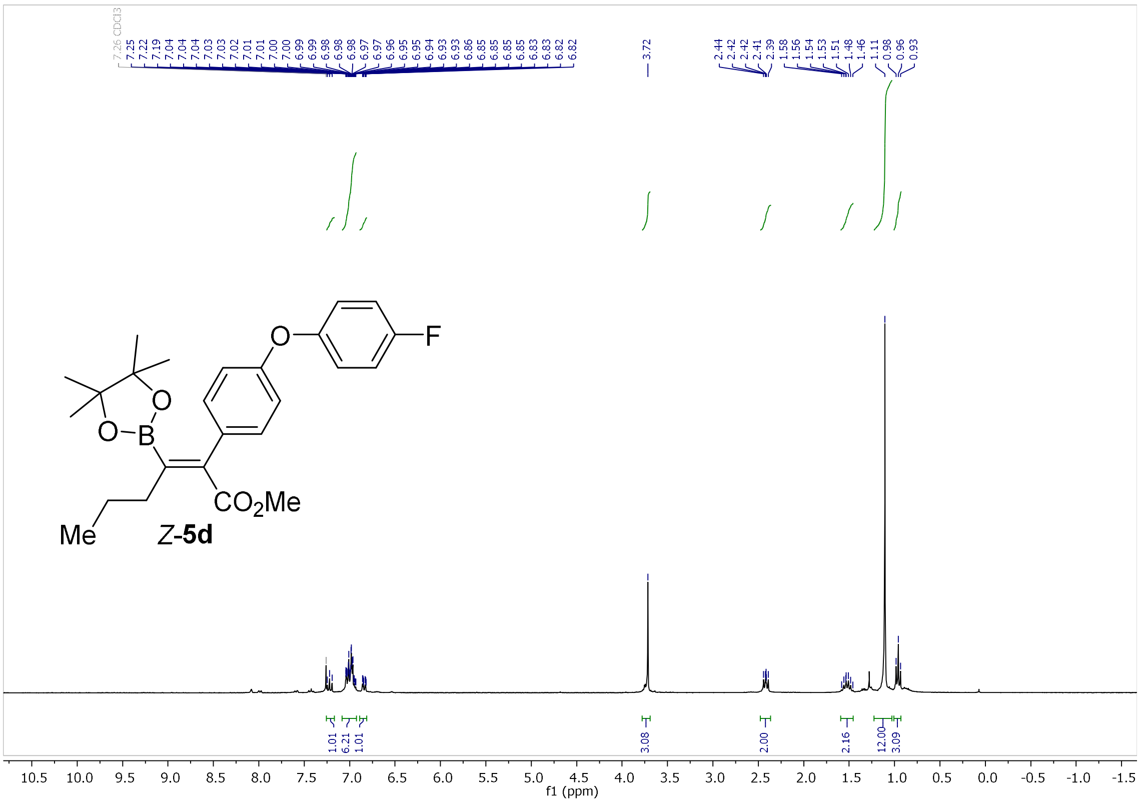

Figure S98. ^{1}H NMR spectrum of *Z*-**5d.**

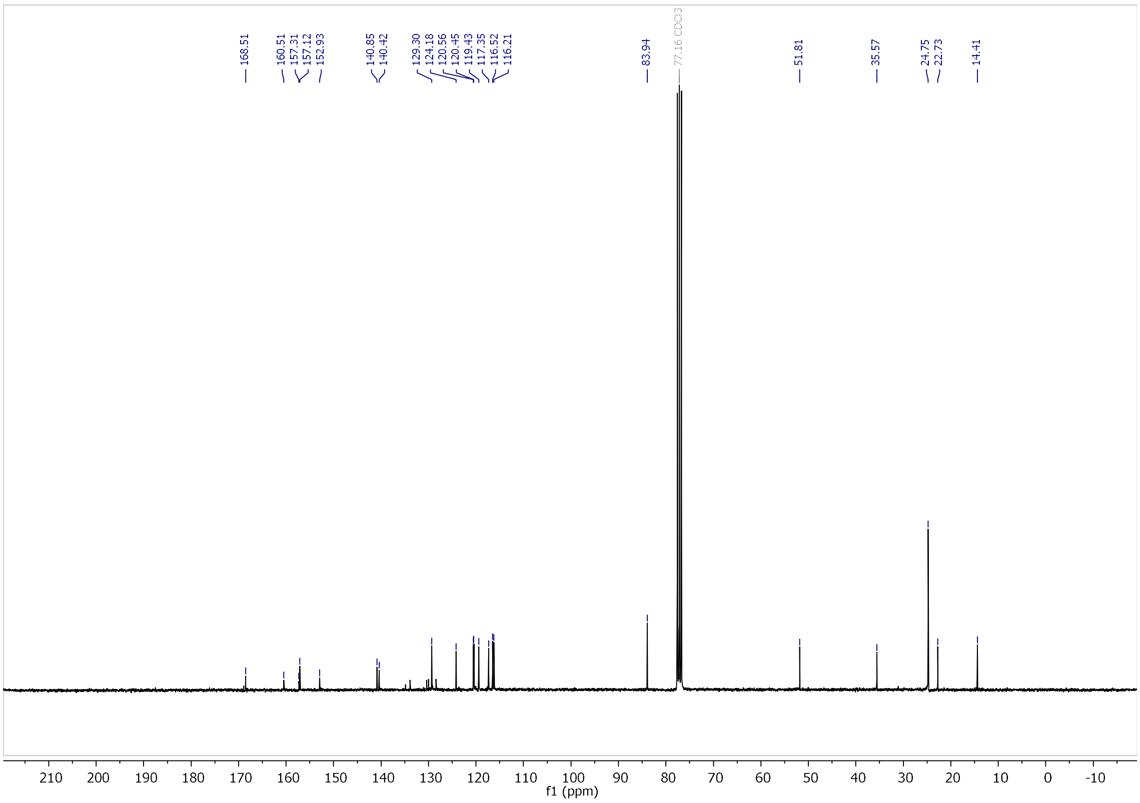

Figure S99. ^{13}C NMR spectrum of *Z*-**5d.**

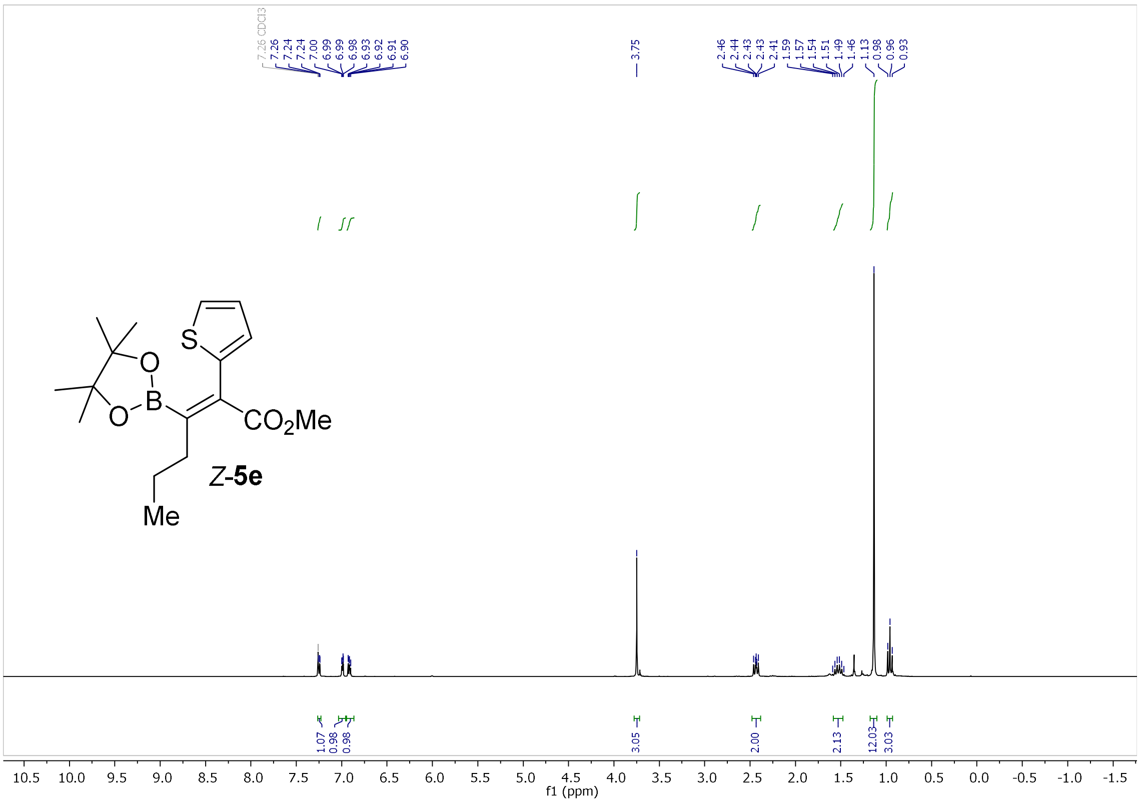

Figure S100. ^{1}H NMR spectrum of *Z*-**5e.**

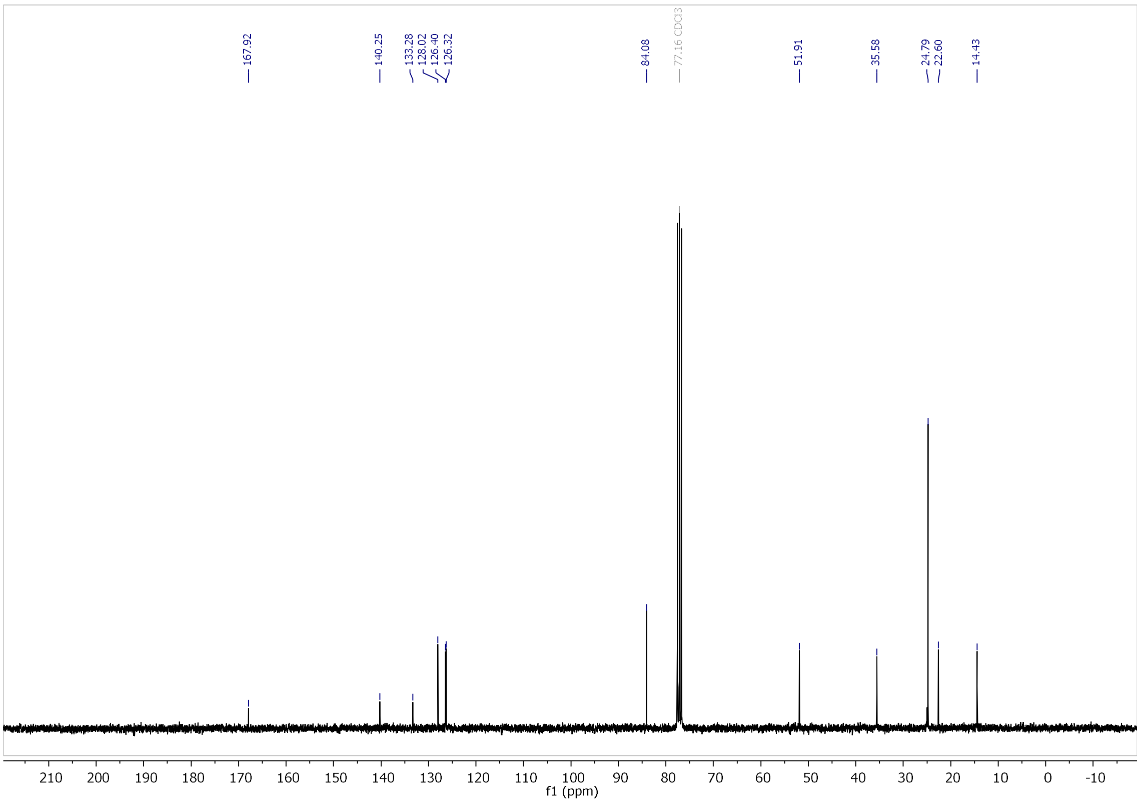

Figure S101. ^{13}C NMR spectrum of *Z*-**5e.**

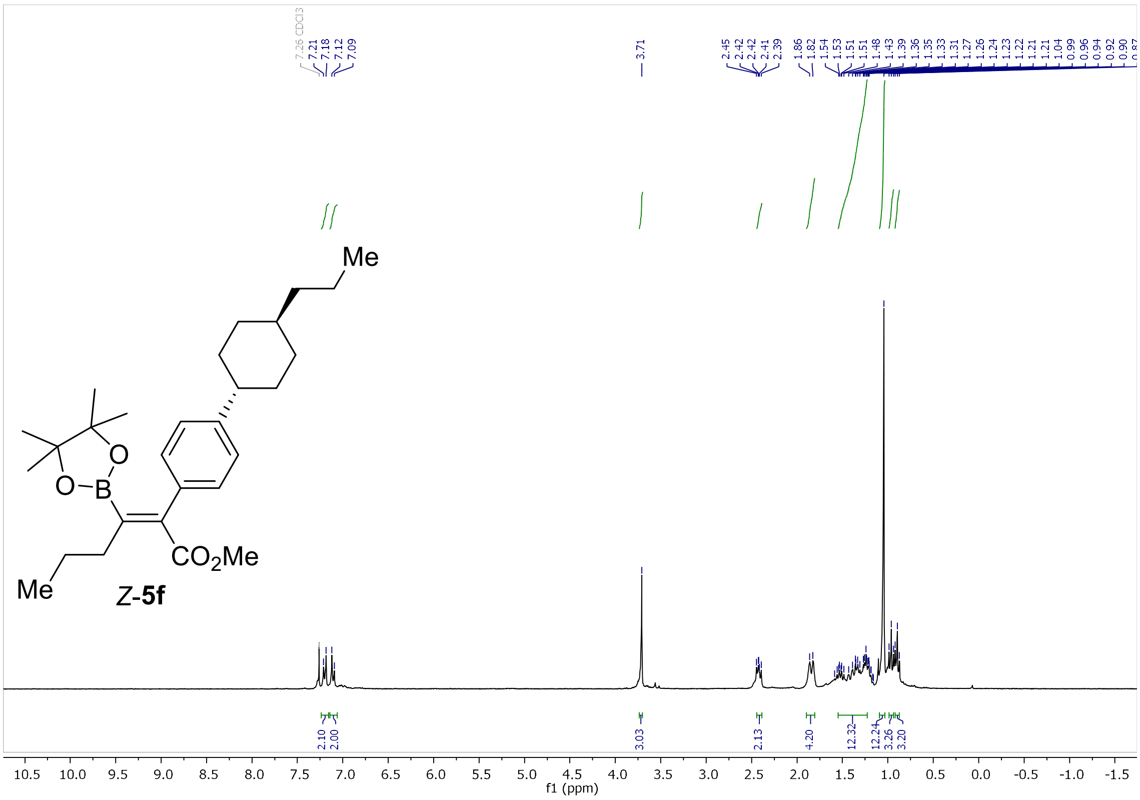

Figure S102. ^{1}H NMR spectrum of *Z*-**5f.**

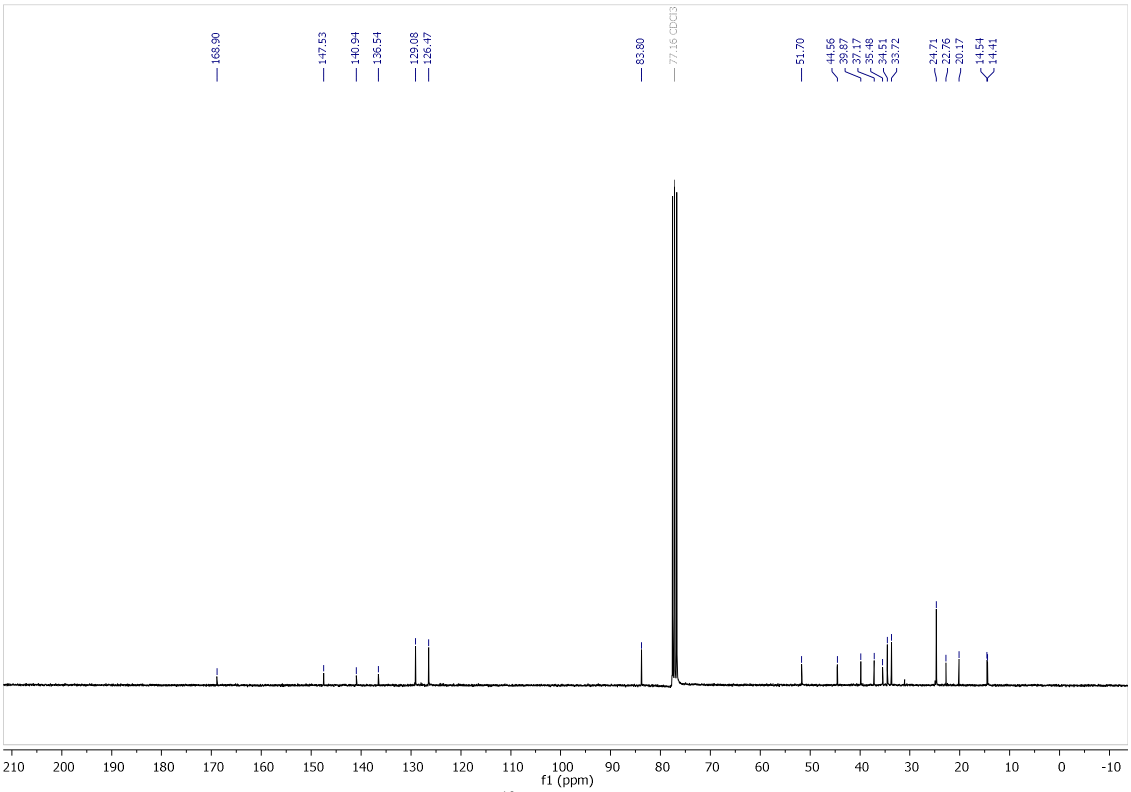

Figure S103. ^{13}C NMR spectrum of *Z*-**5f.**

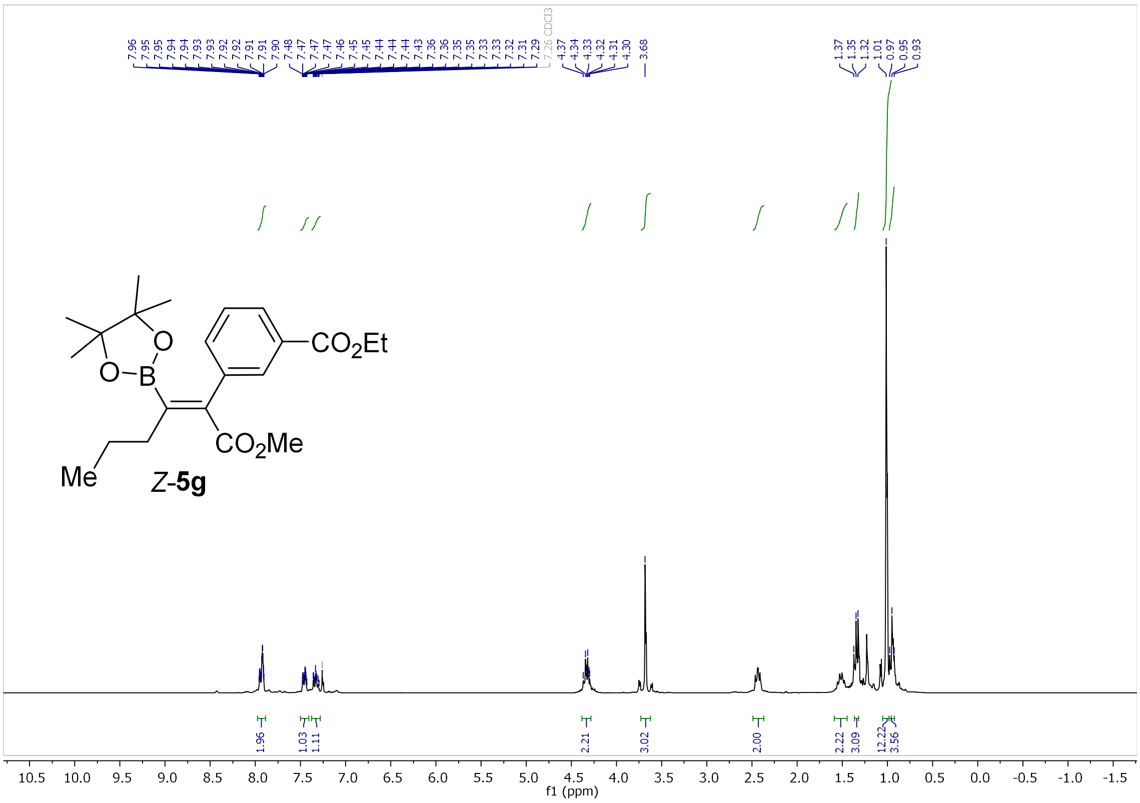

Figure S104. ^{1}H NMR spectrum of *Z*-**5g.**

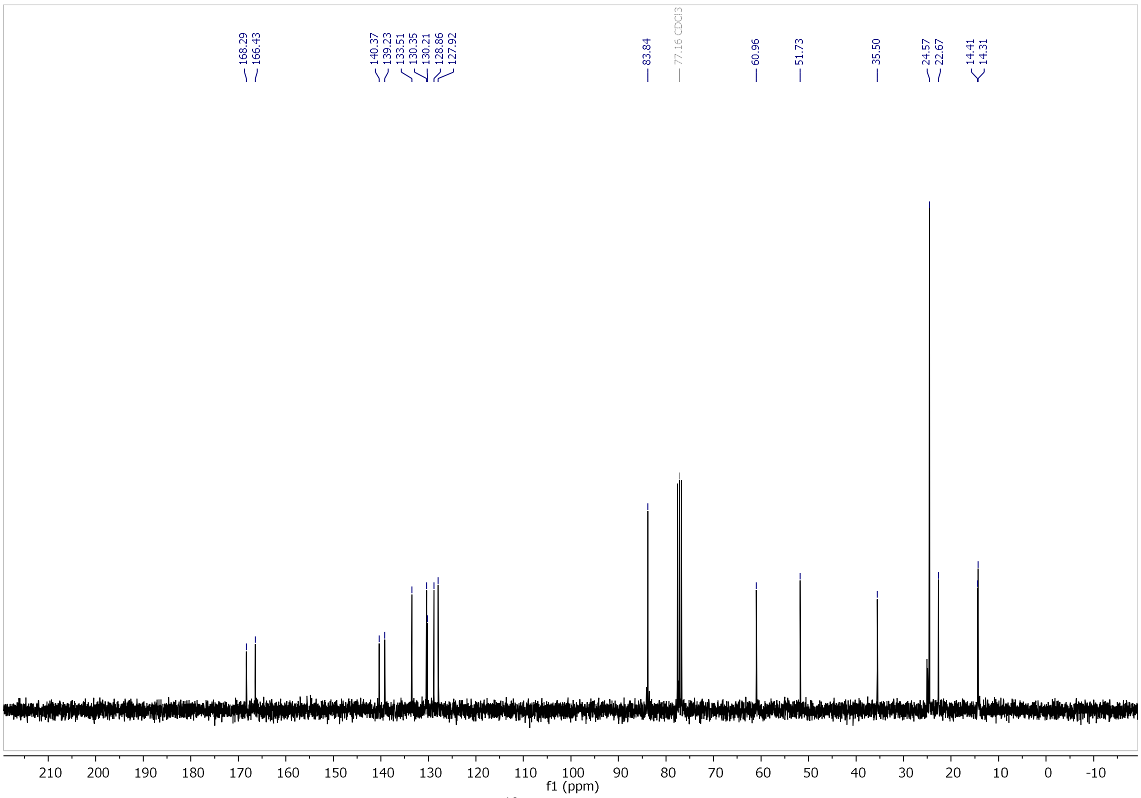

Figure S105. ^{13}C NMR spectrum of *Z*-**5g.**

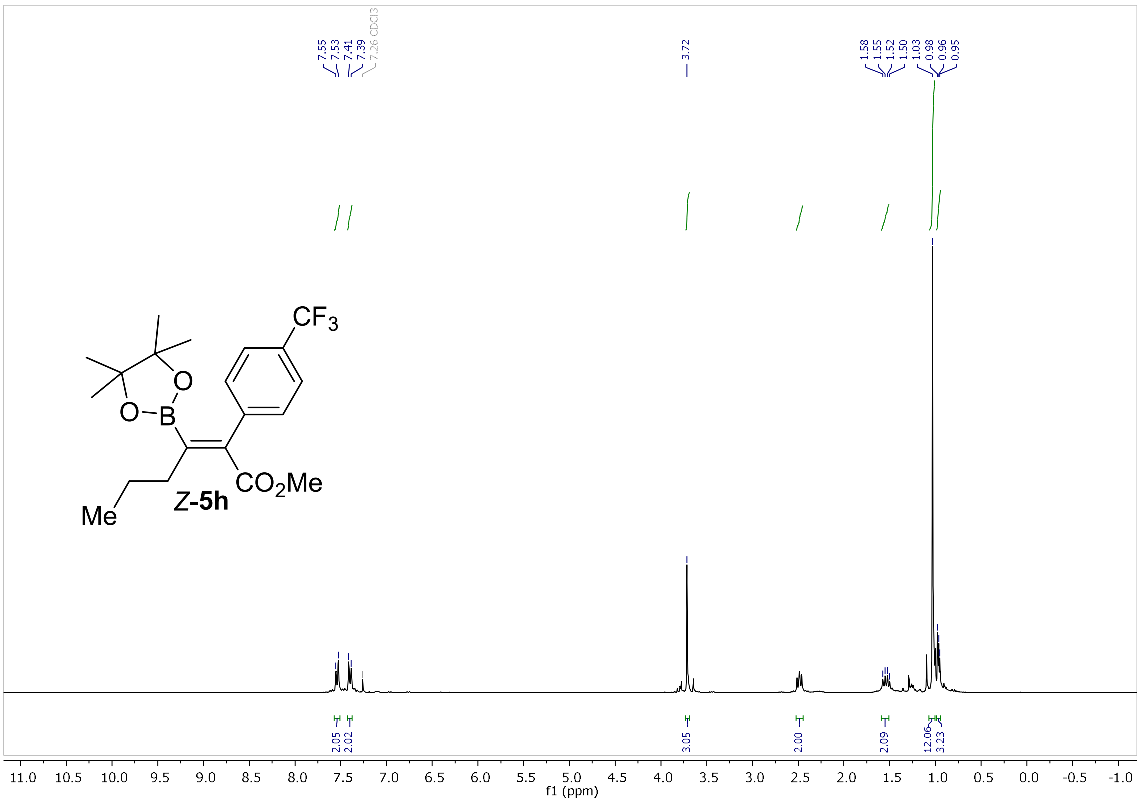

Figure S106. ^{1}H NMR spectrum of *Z*-**5h.**

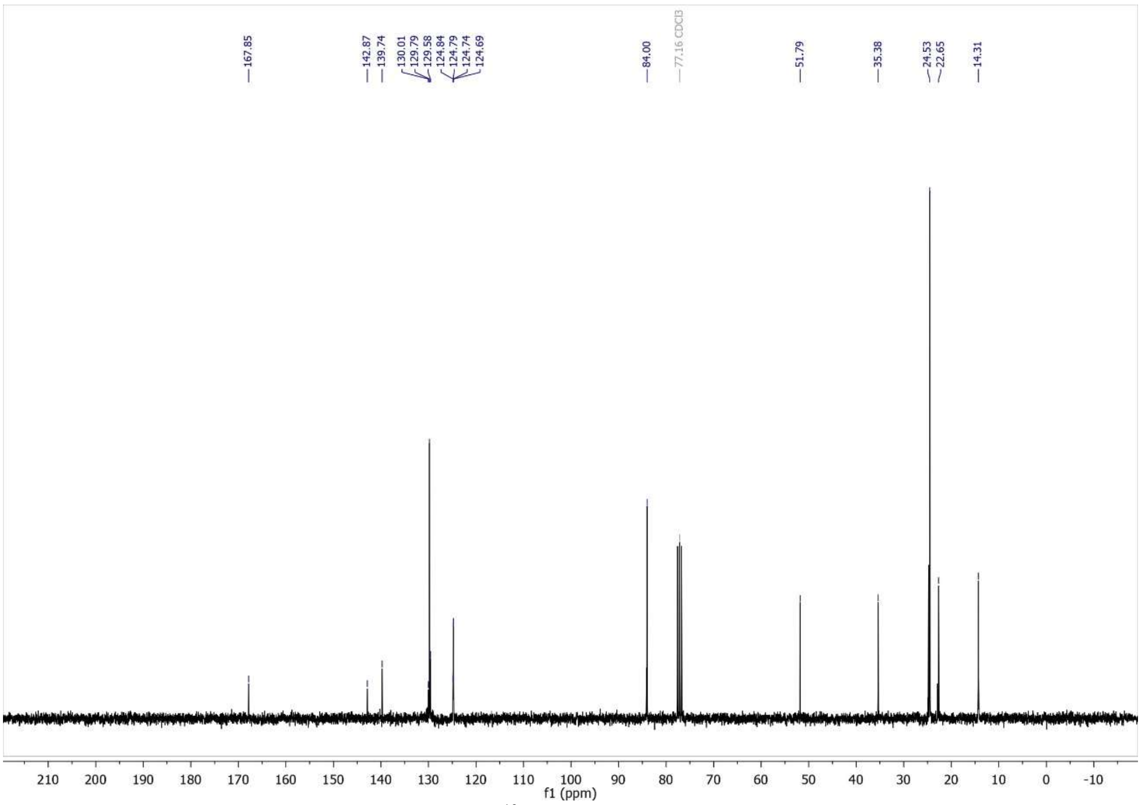

Figure S107. ^{13}C NMR spectrum of *Z*-**5h.**

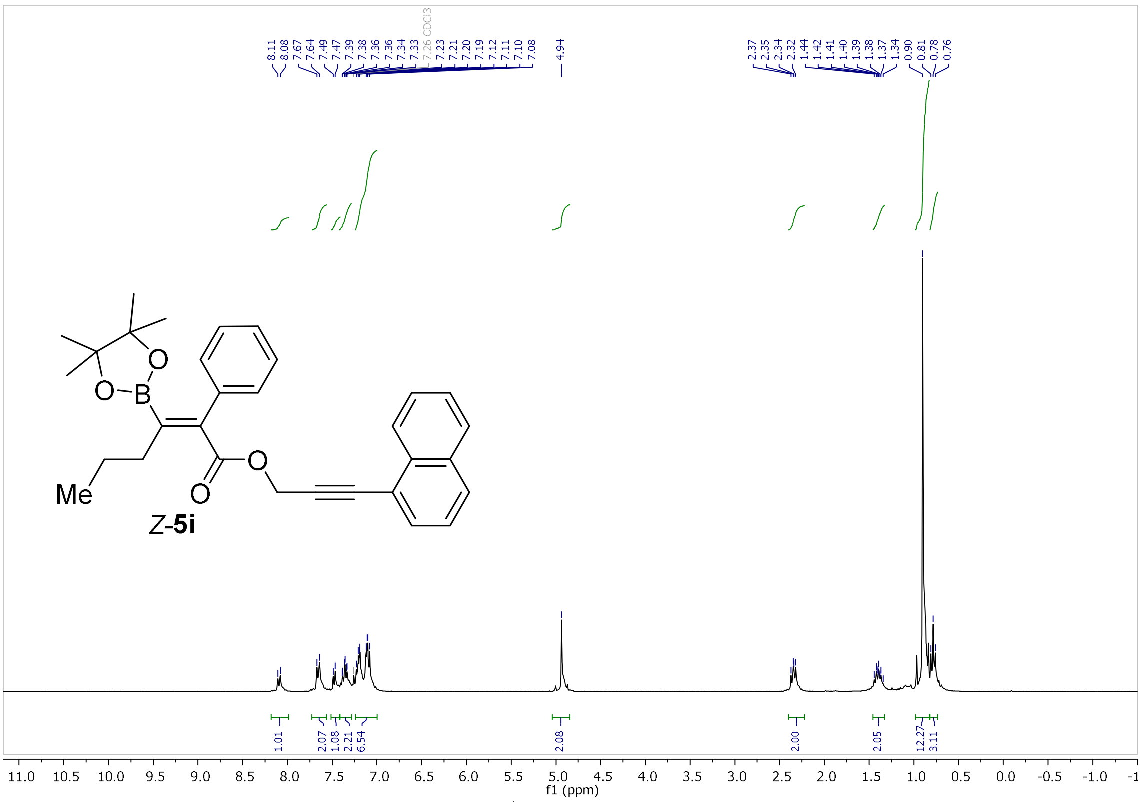

Figure S108. ^{1}H NMR spectrum of *Z*-**5i.**

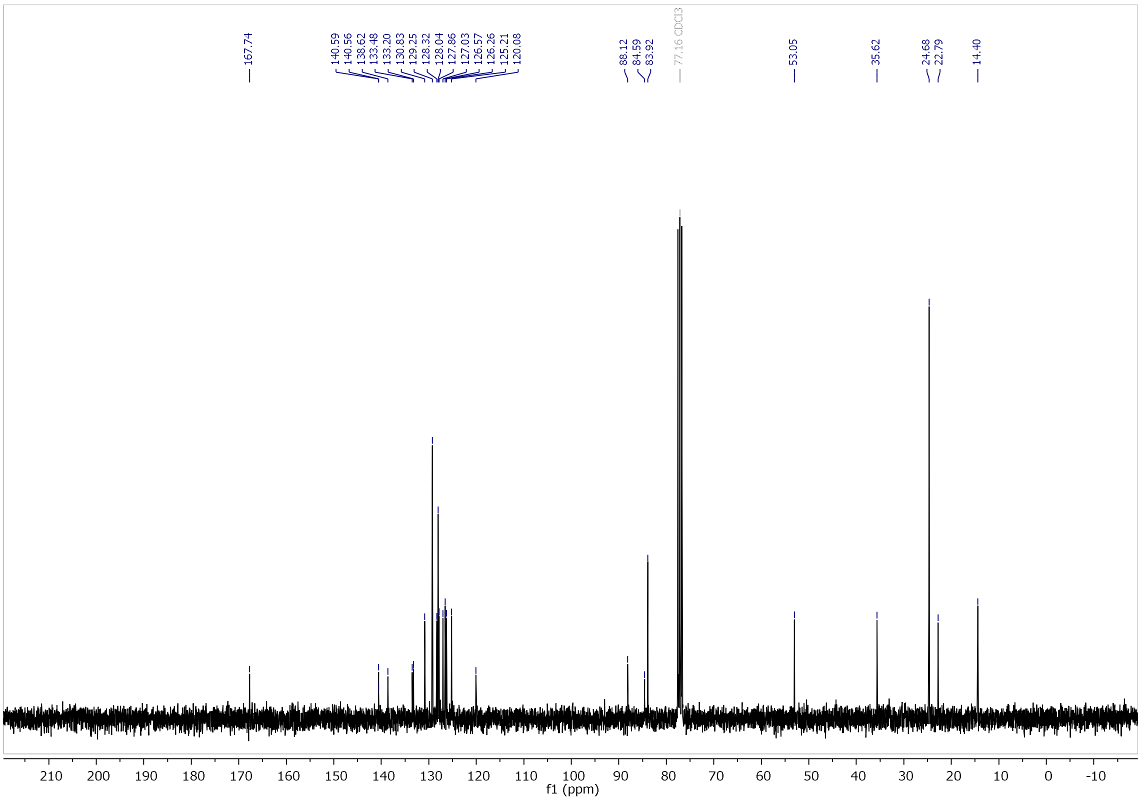

Figure S109. ^{13}C NMR spectrum of *Z*-**5i.**

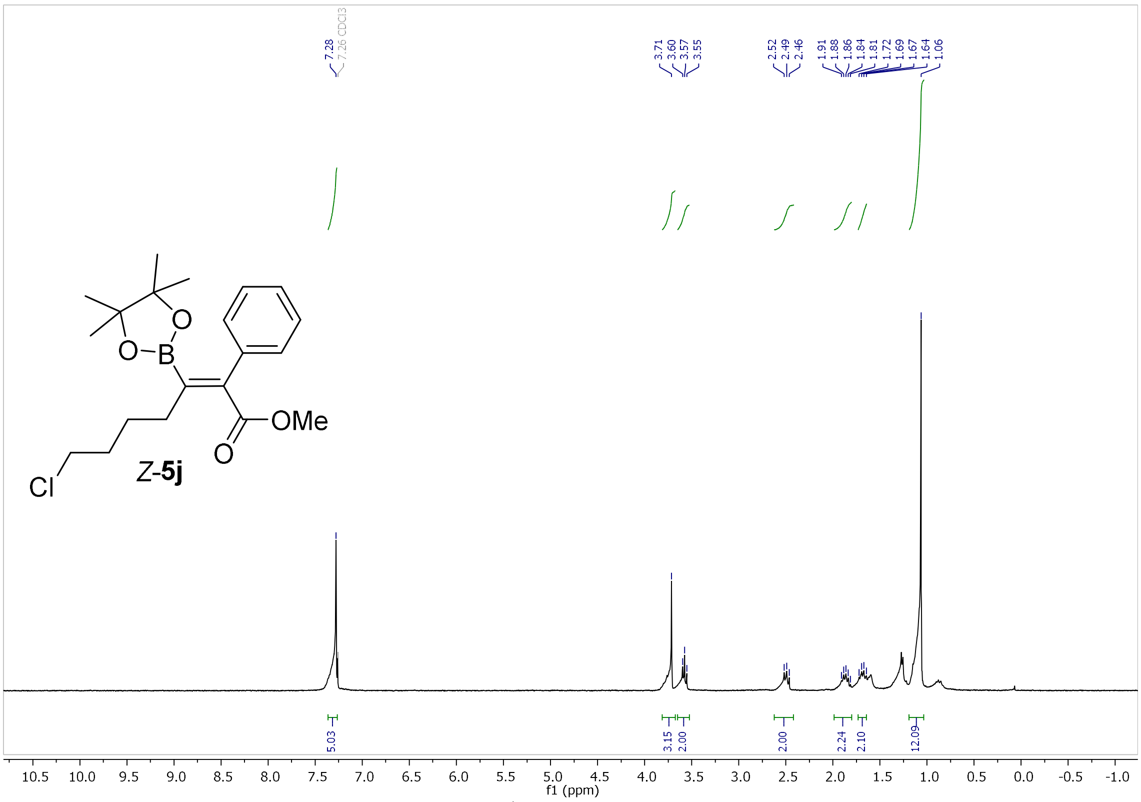

Figure S110. ^{1}H NMR spectrum of *Z*-**5j.**

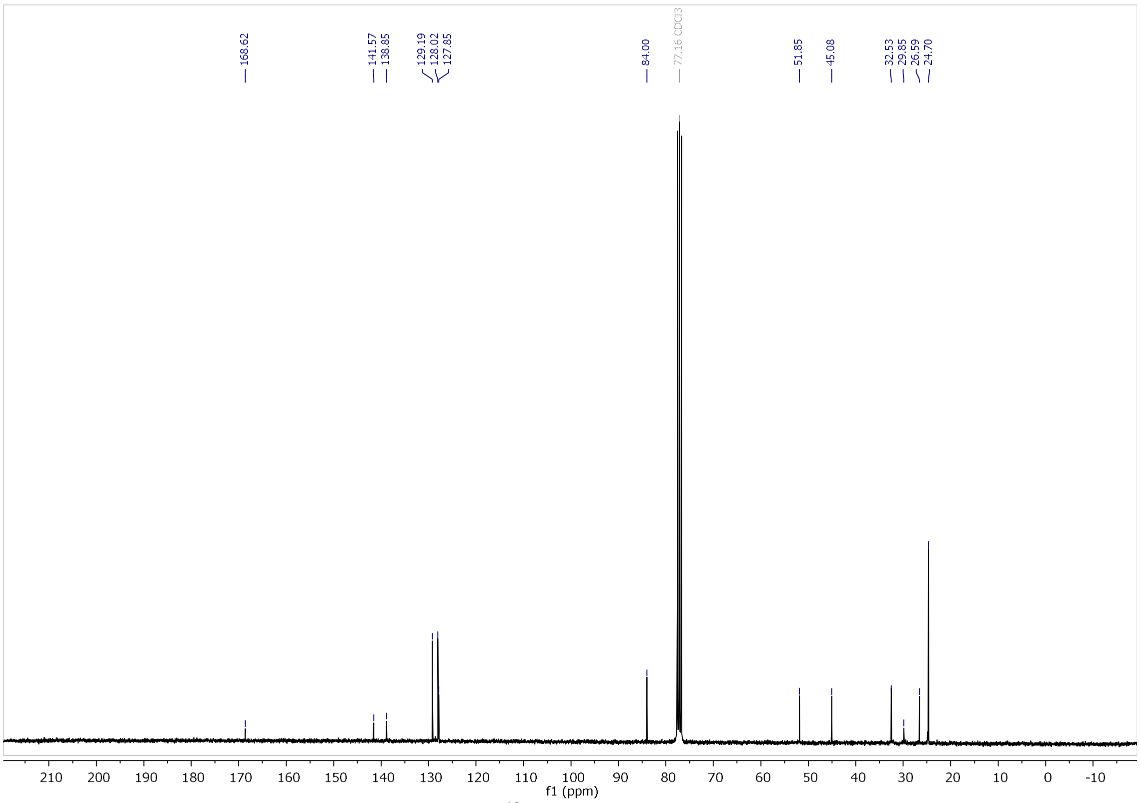

Figure S111. ^{13}C NMR spectrum of *Z*-**5j.**

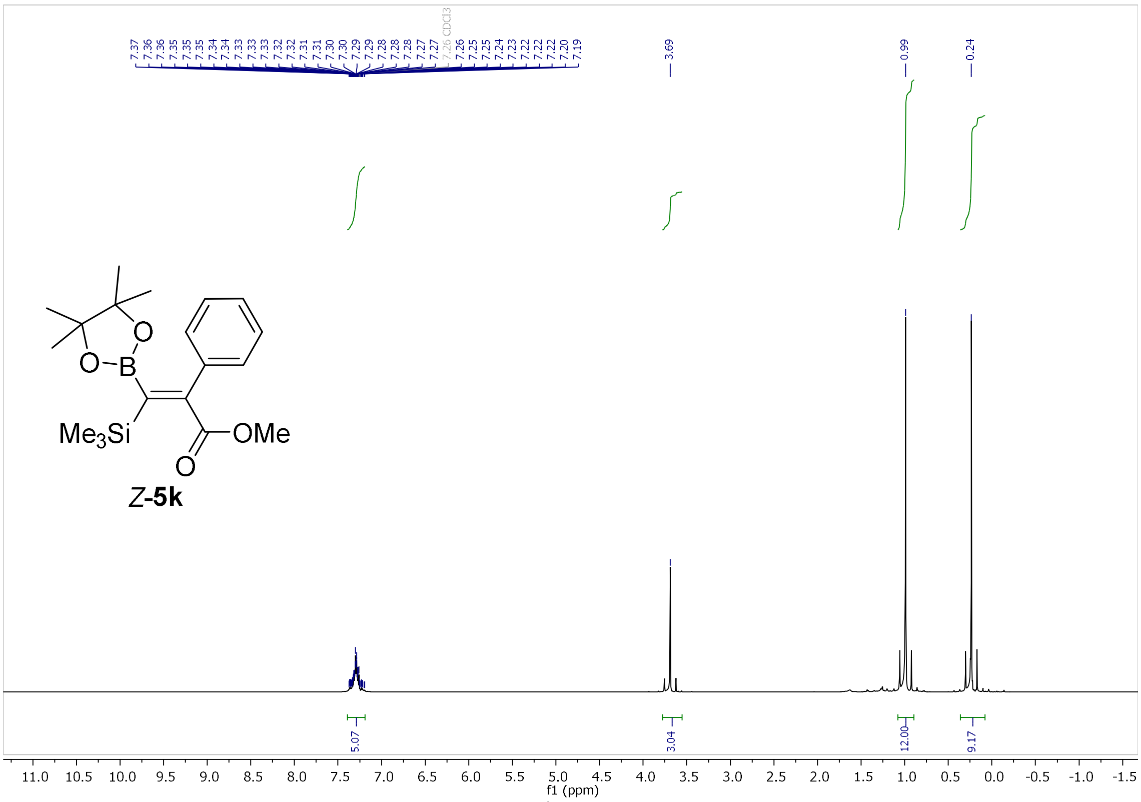

Figure S112. ^{1}H NMR spectrum of *Z*-**5k.**

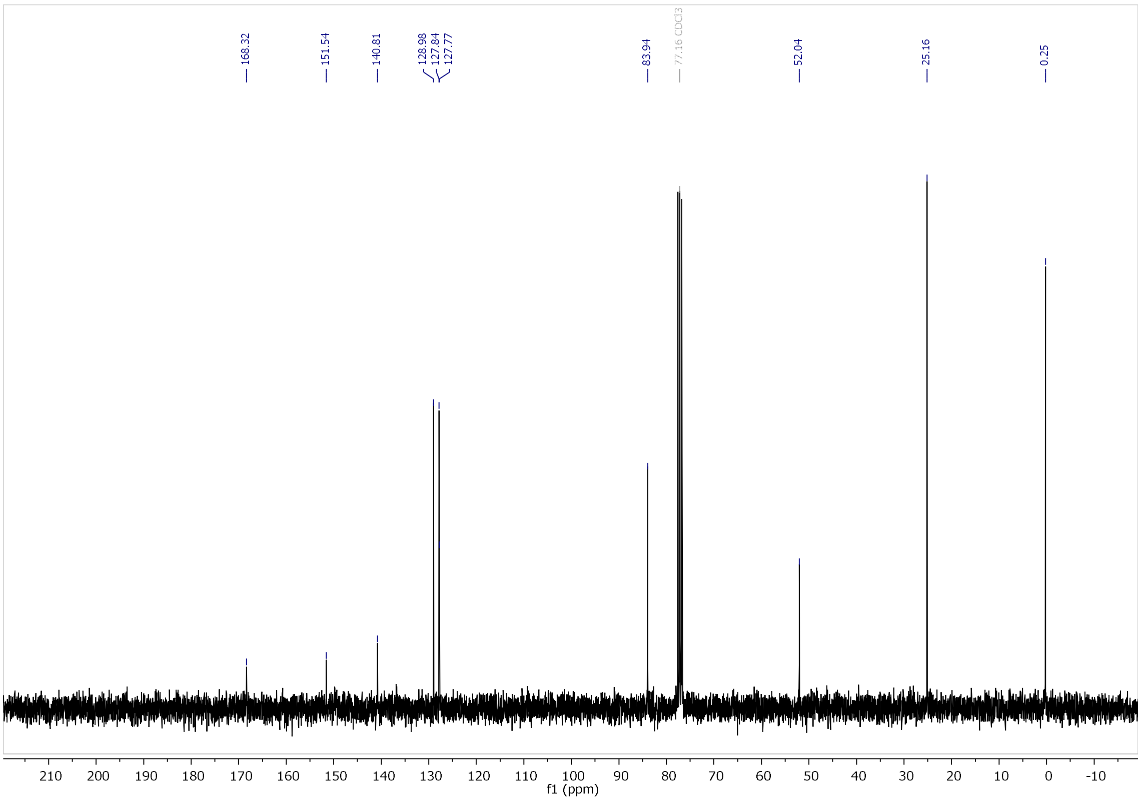

Figure S113. ^{13}C NMR spectrum of *Z*-**5k.**

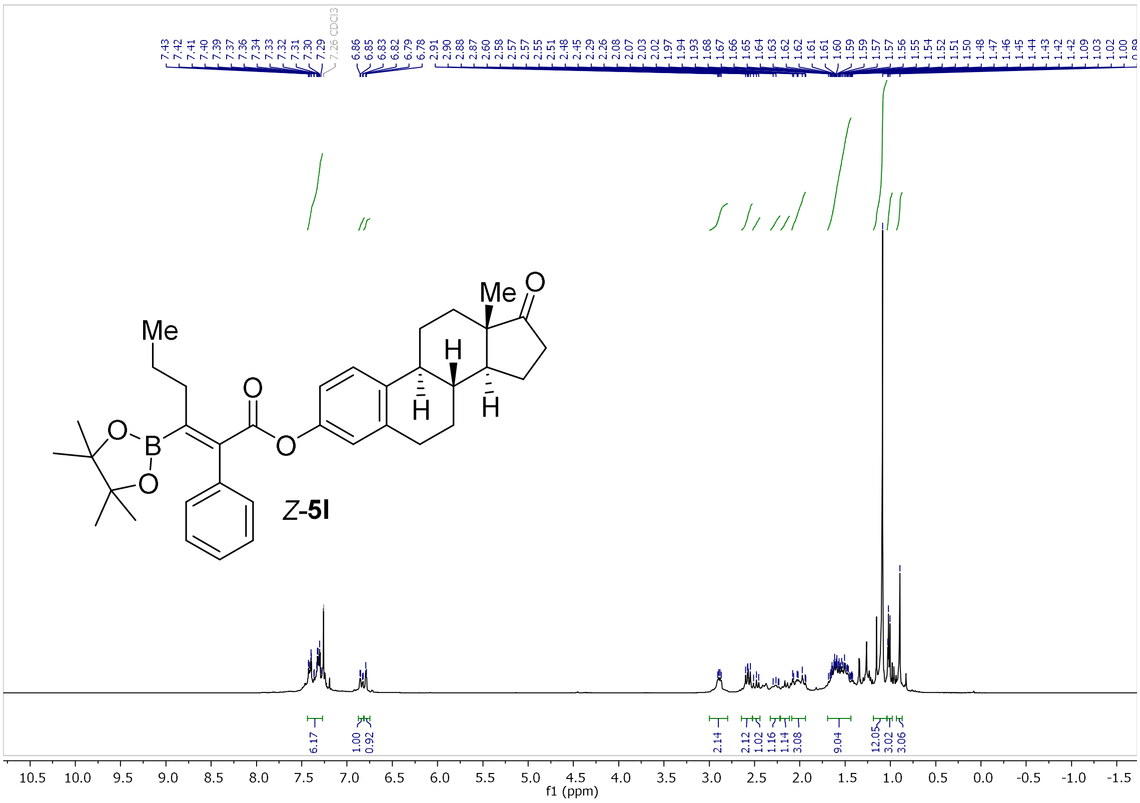

Figure S114. ^{1}H NMR spectrum of *Z*-**5l.**

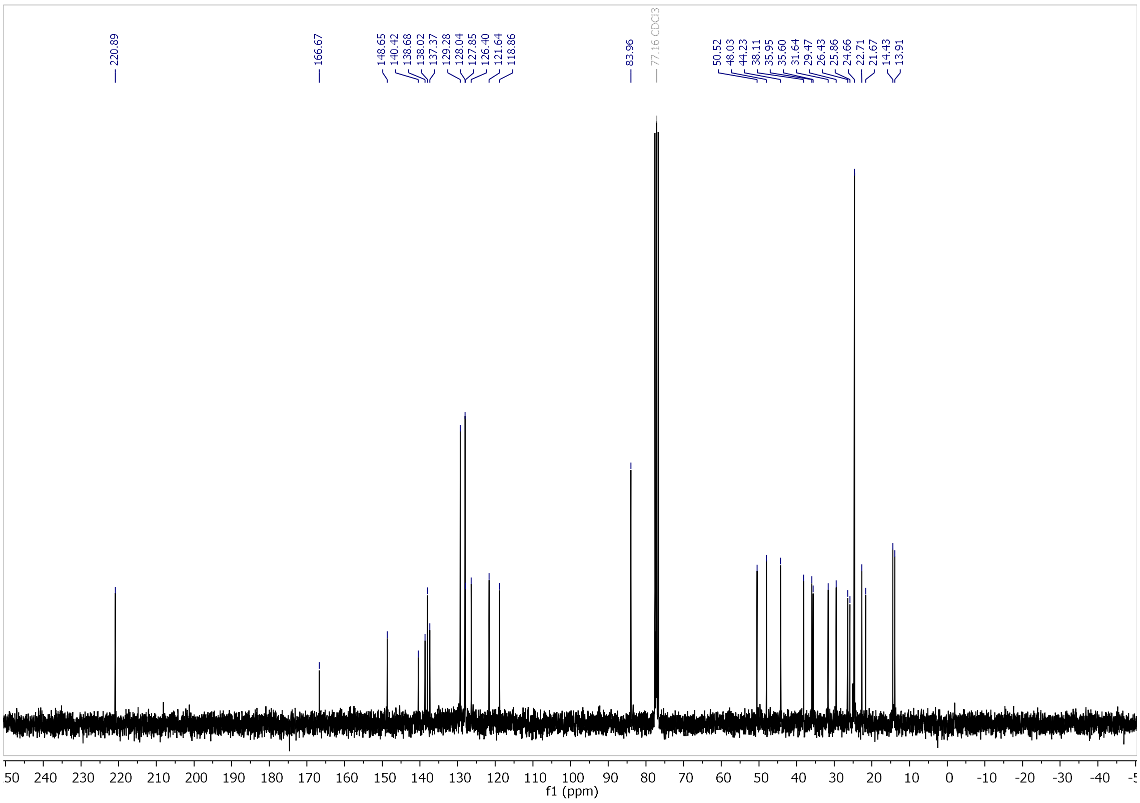

Figure S115. ^{13}C NMR spectrum of *Z*-**5l.**

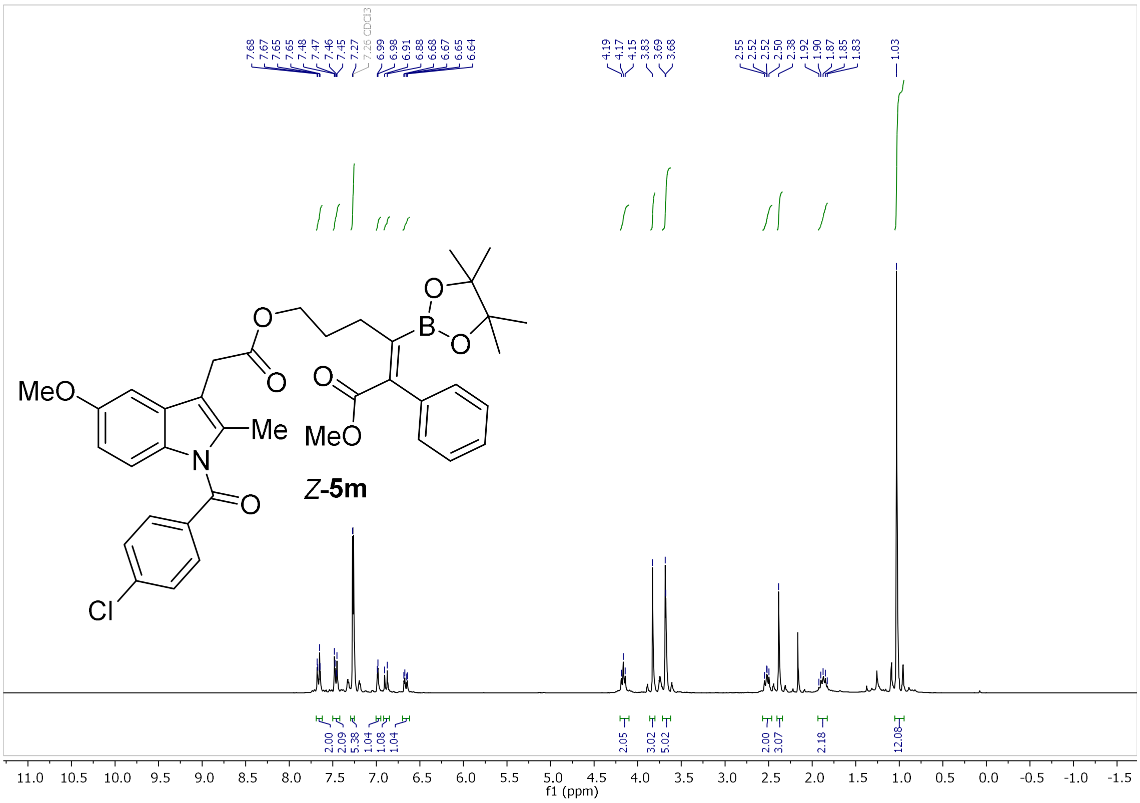

Figure S116. ^{1}H NMR spectrum of *Z*-**5m.**

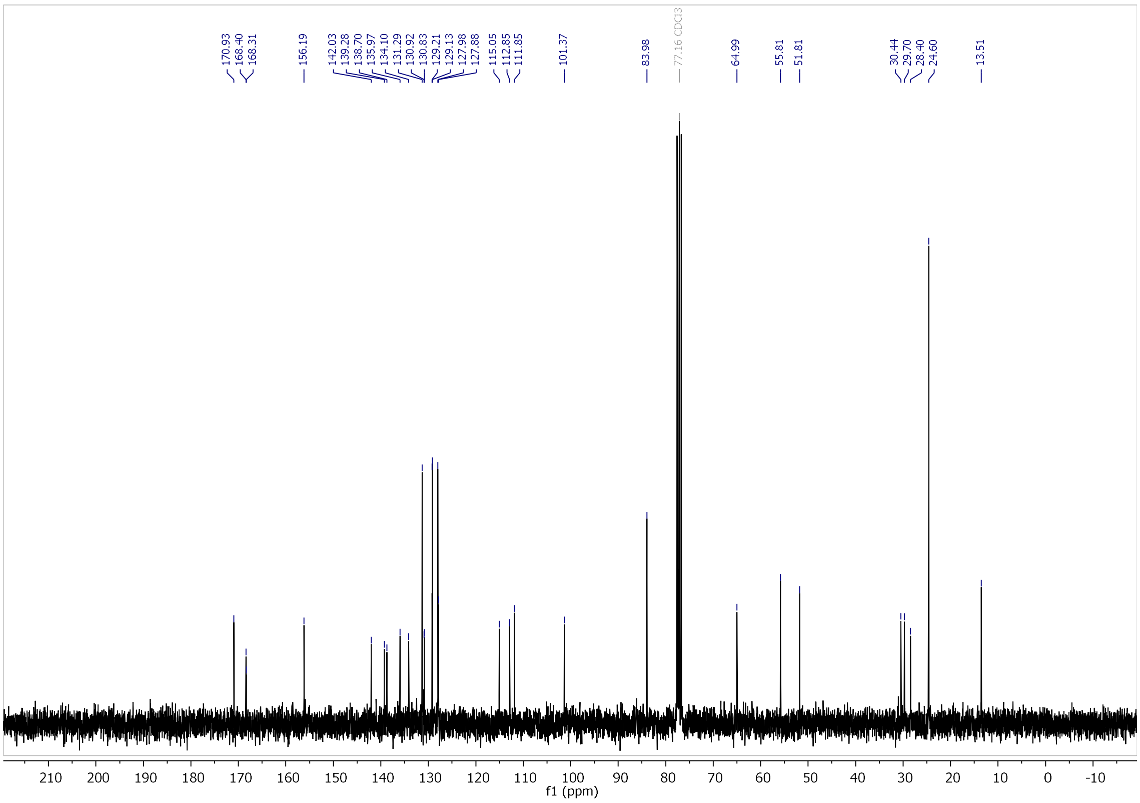

Figure S117. ^{13}C NMR spectrum of *Z*-**5m.**

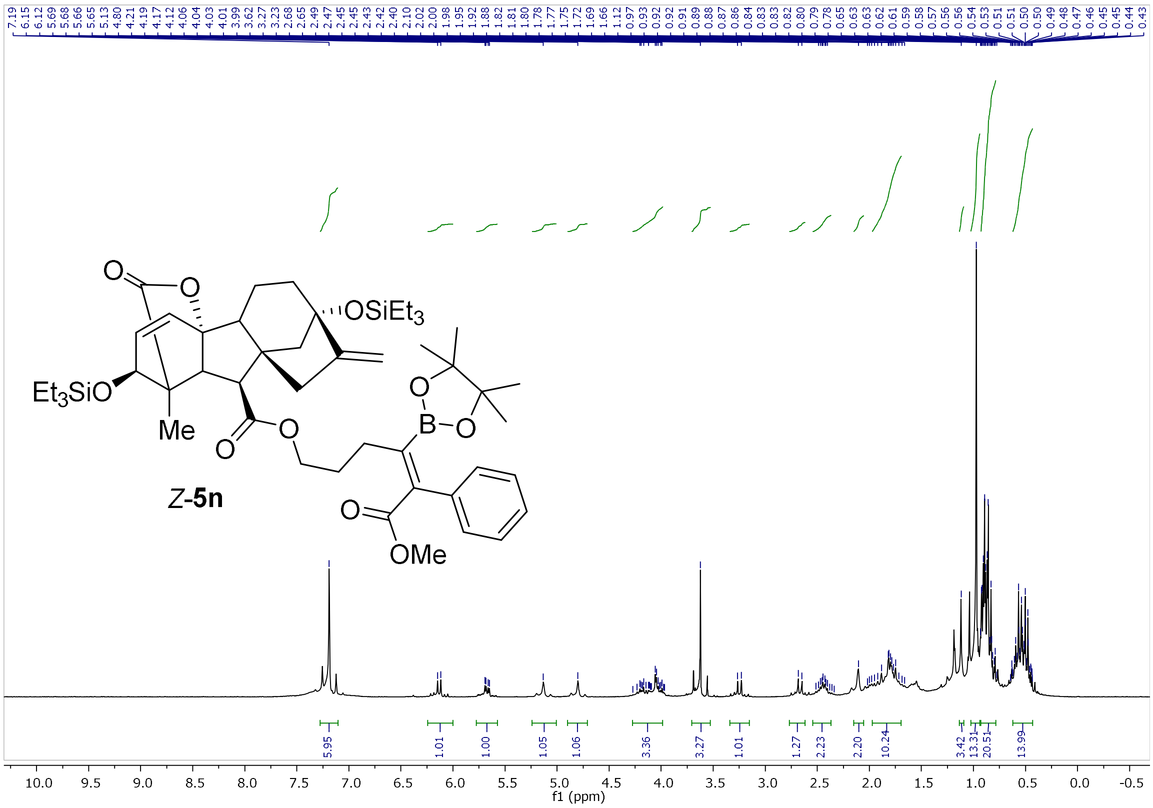

Figure S118. ^{1}H NMR spectrum of *Z*-**5n.**

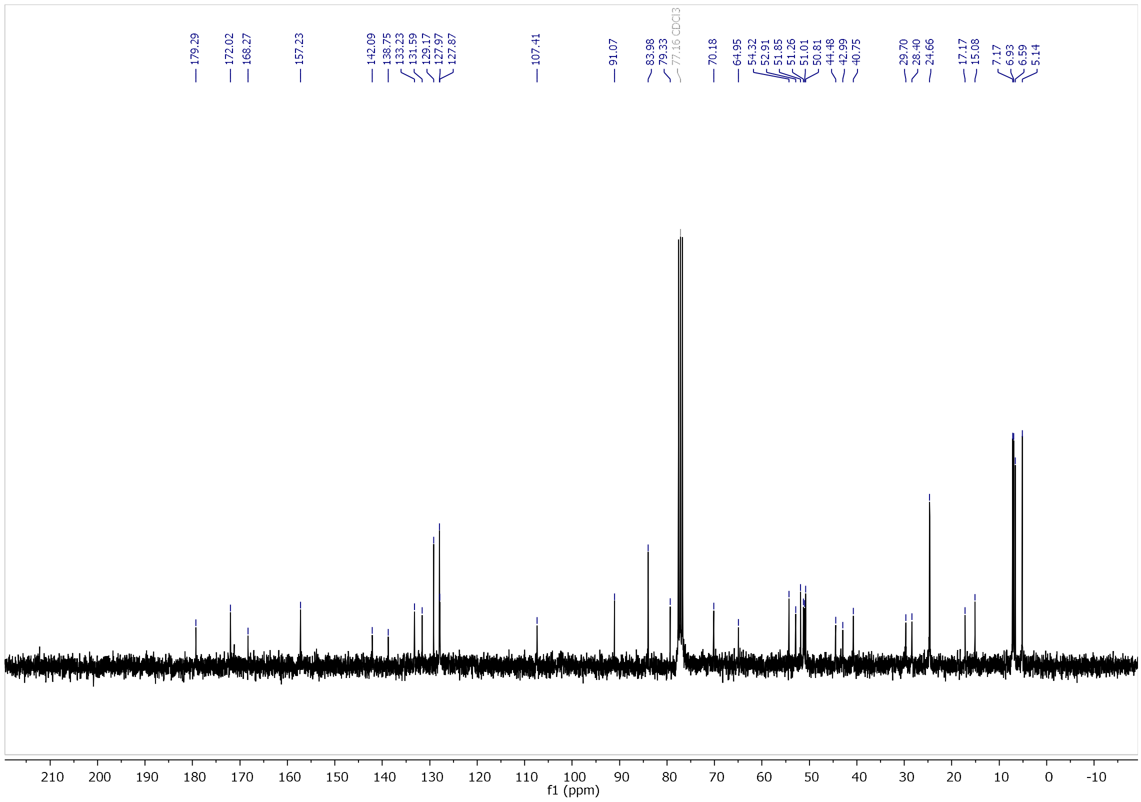

Figure S119. ^{13}C NMR spectrum of *Z*-**5n.**

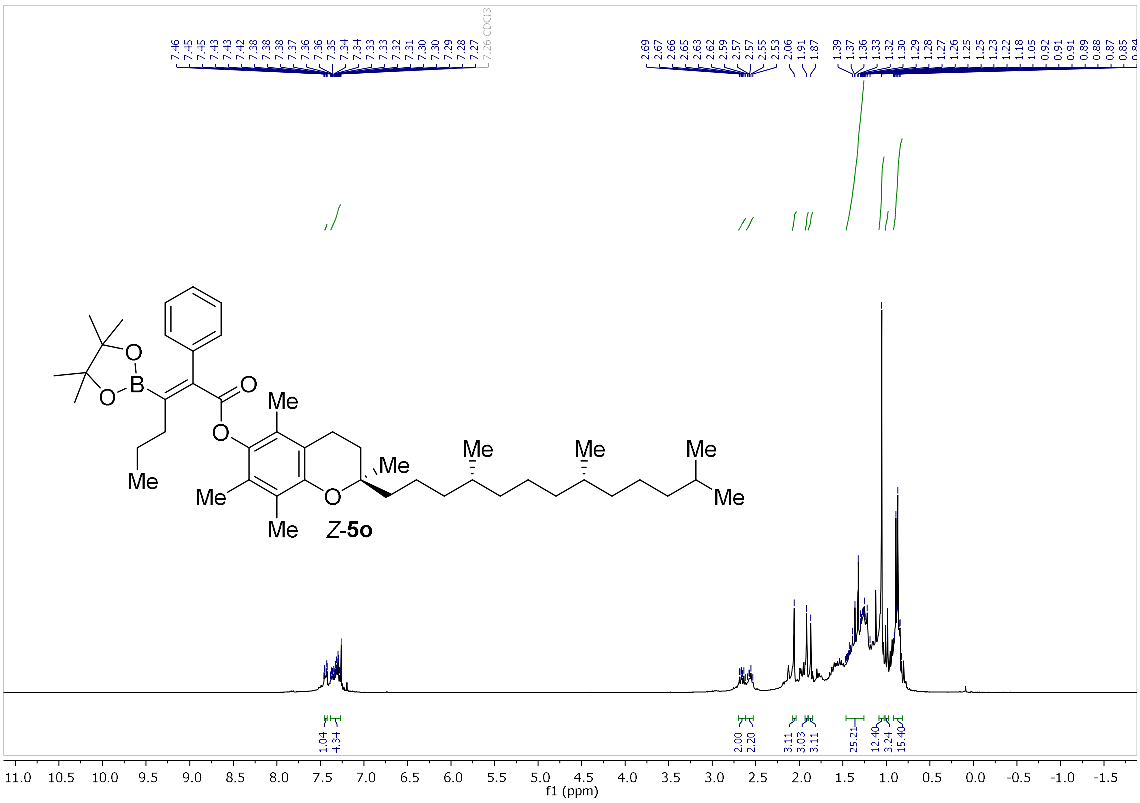

Figure S120. ^{1}H NMR spectrum of *Z*-**5o.**

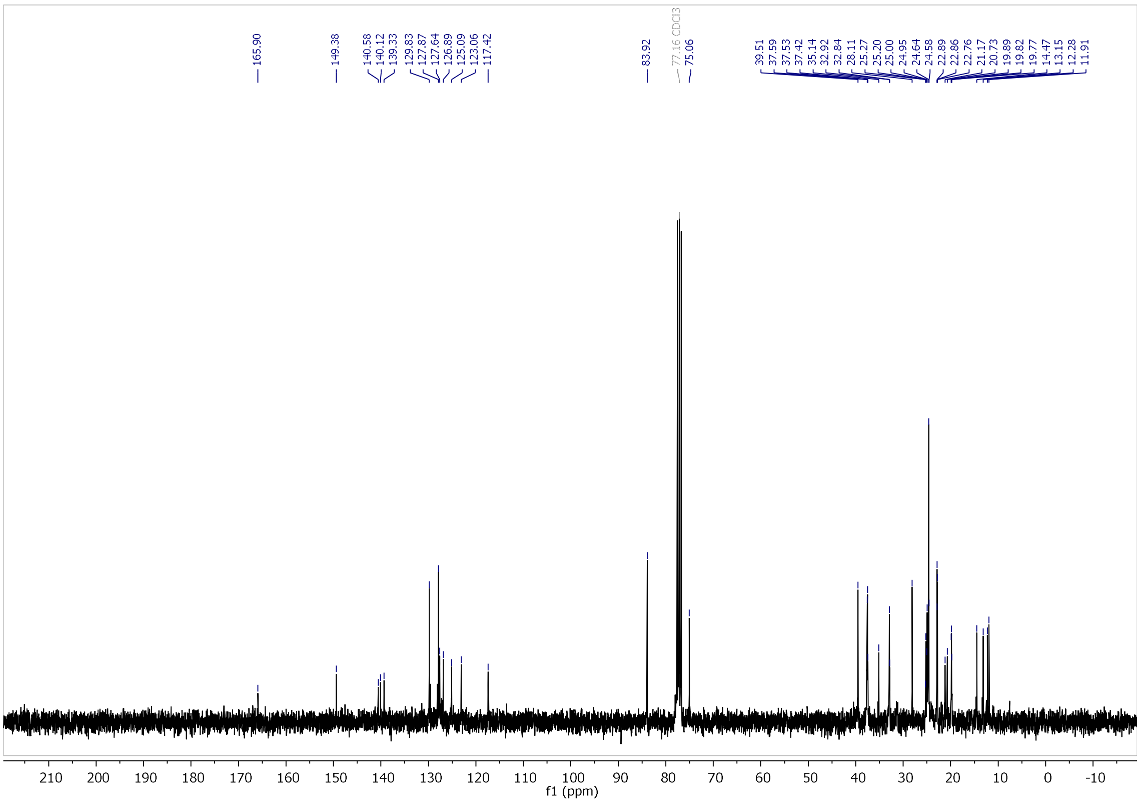

Figure S121. ^{13}C NMR spectrum of *Z*-**5o.**

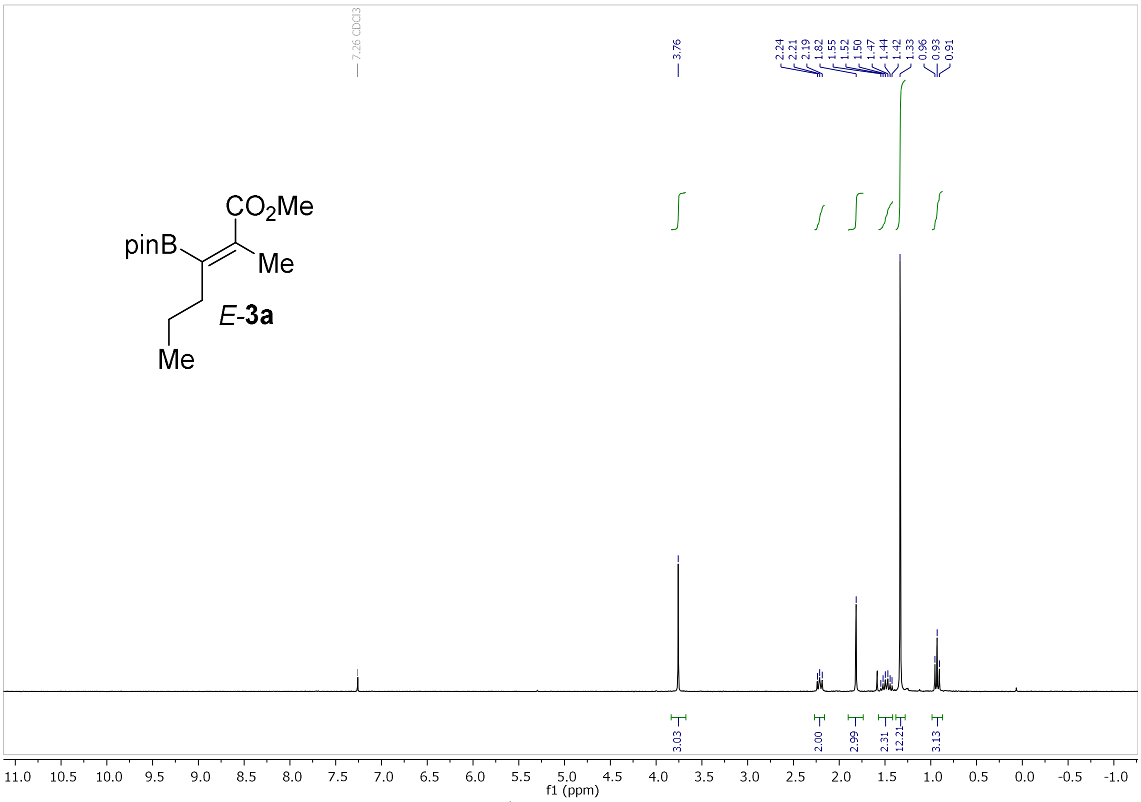

Figure S122. ^{1}H NMR spectrum of *E*-**3a.**

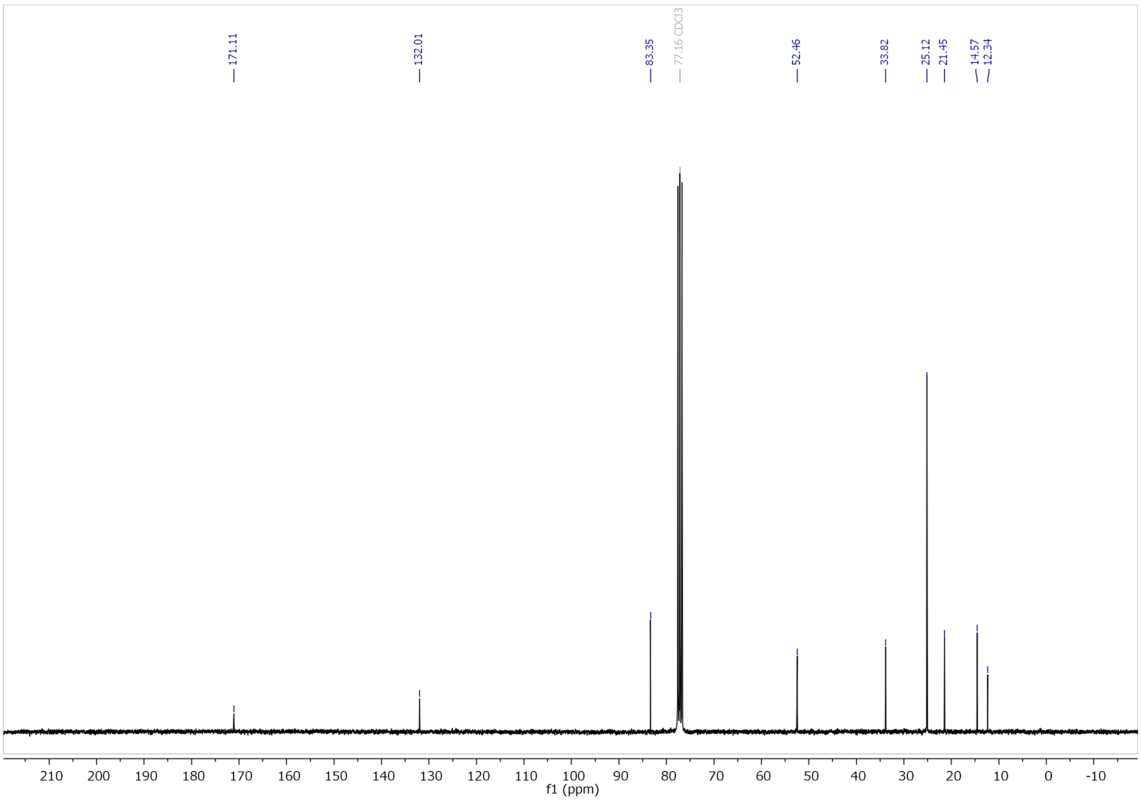

Figure S123. ^{13}C NMR spectrum of *E*-**3a.**

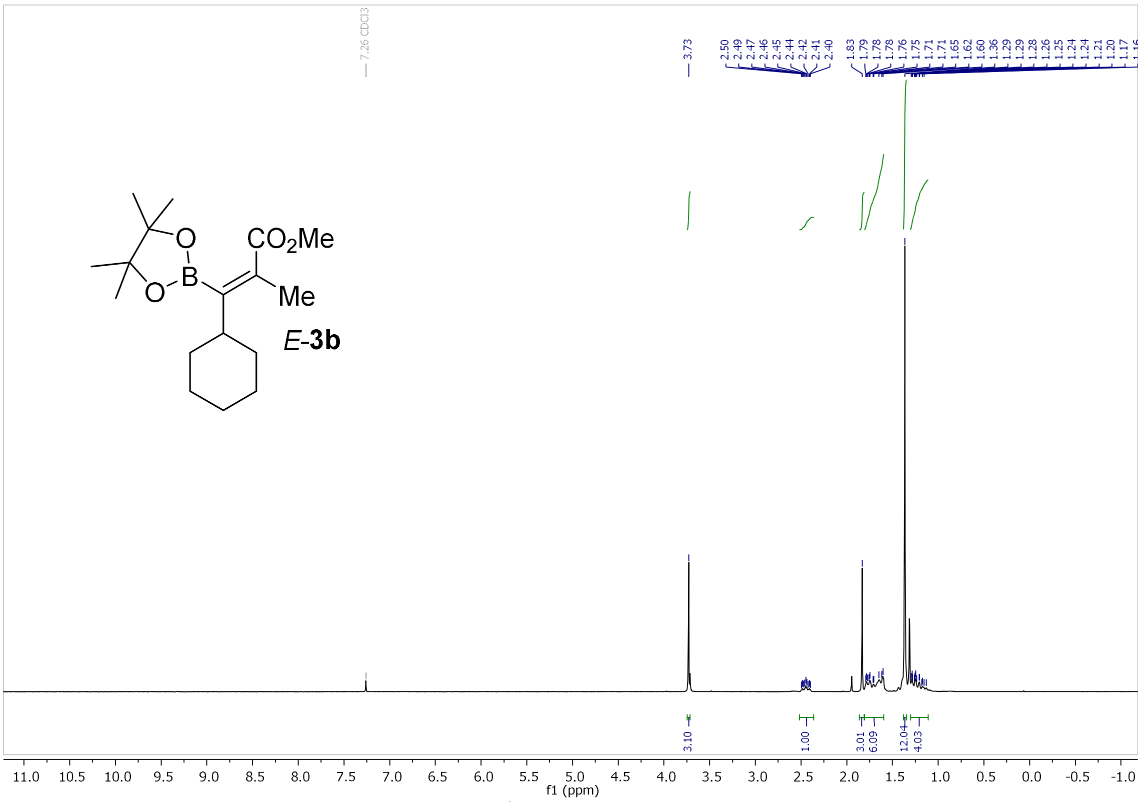

Figure S124. ^{1}H NMR spectrum of *E*-**3b.**

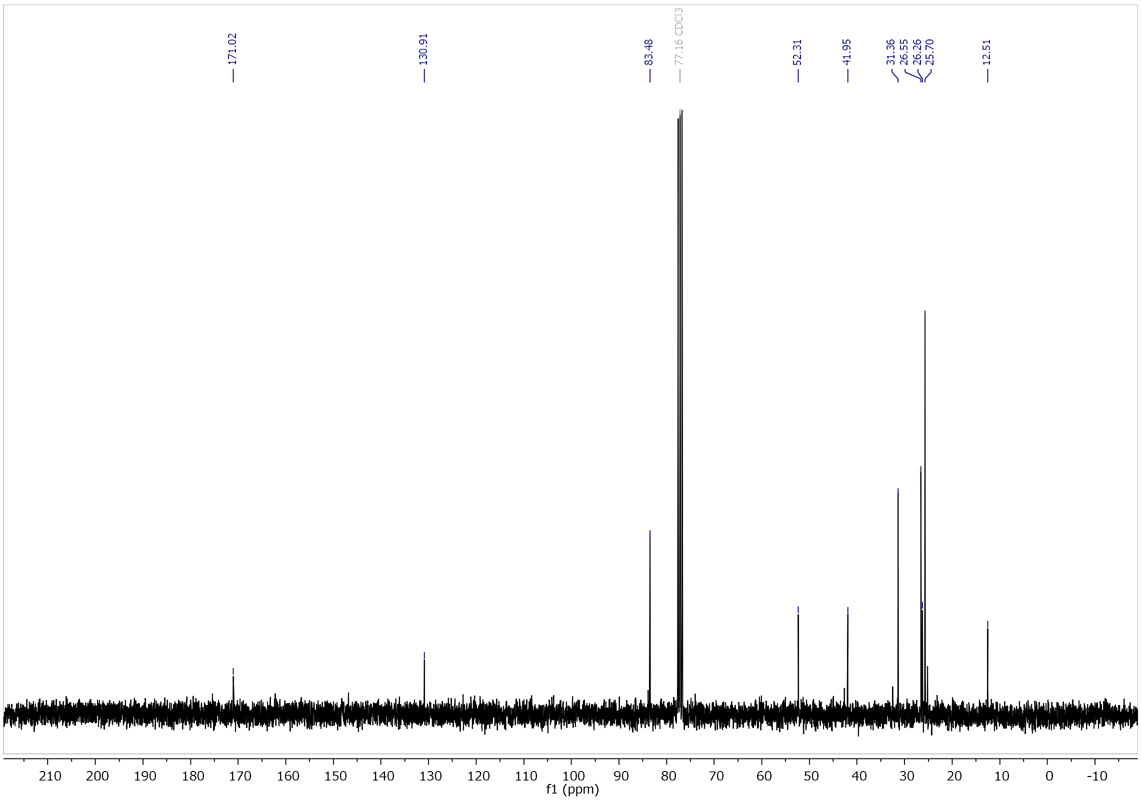

Figure S125. ^{13}C NMR spectrum of *E*-**3b.**

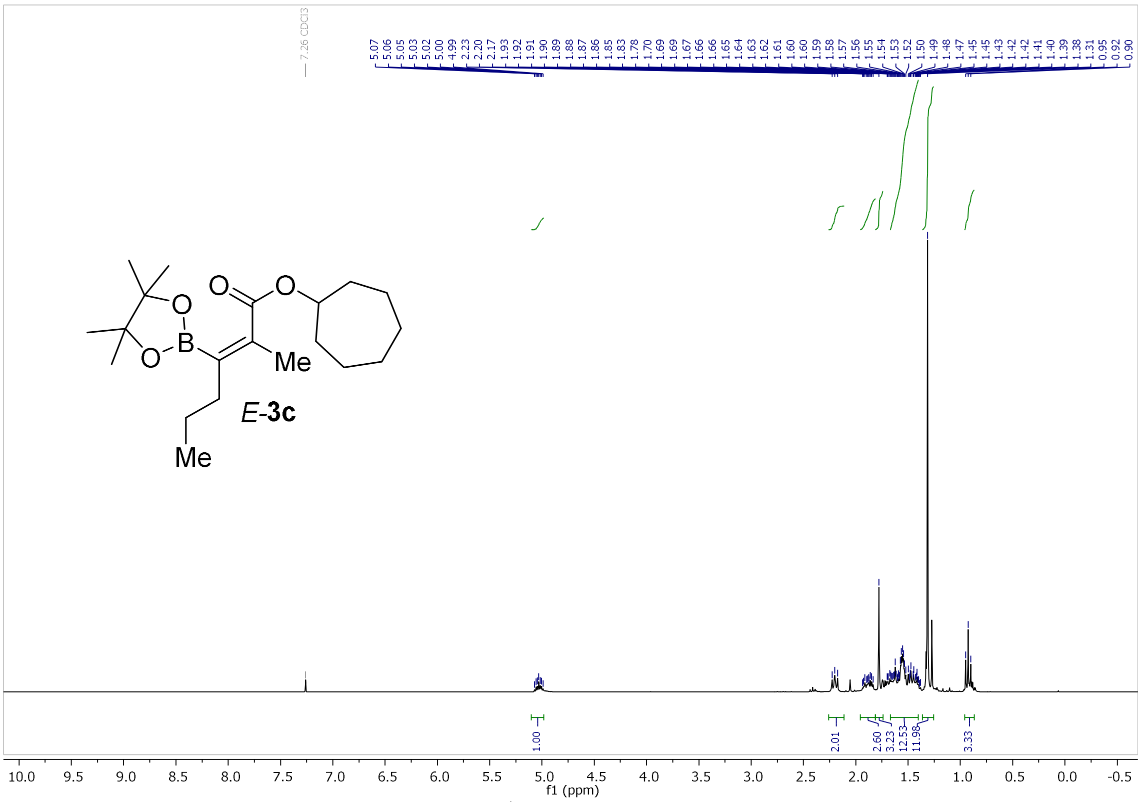

Figure S126. ^{1}H NMR spectrum of *E*-**3c.**

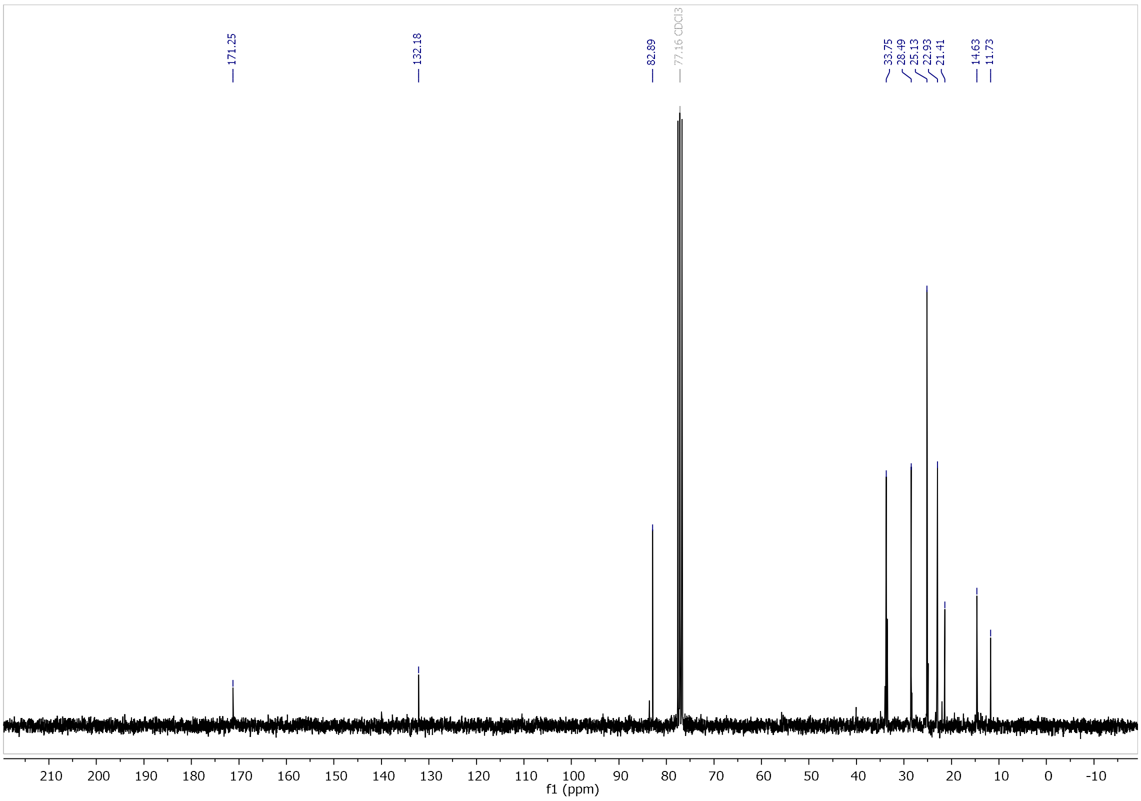

Figure S127. ^{13}C NMR spectrum of *E*-**3c.**

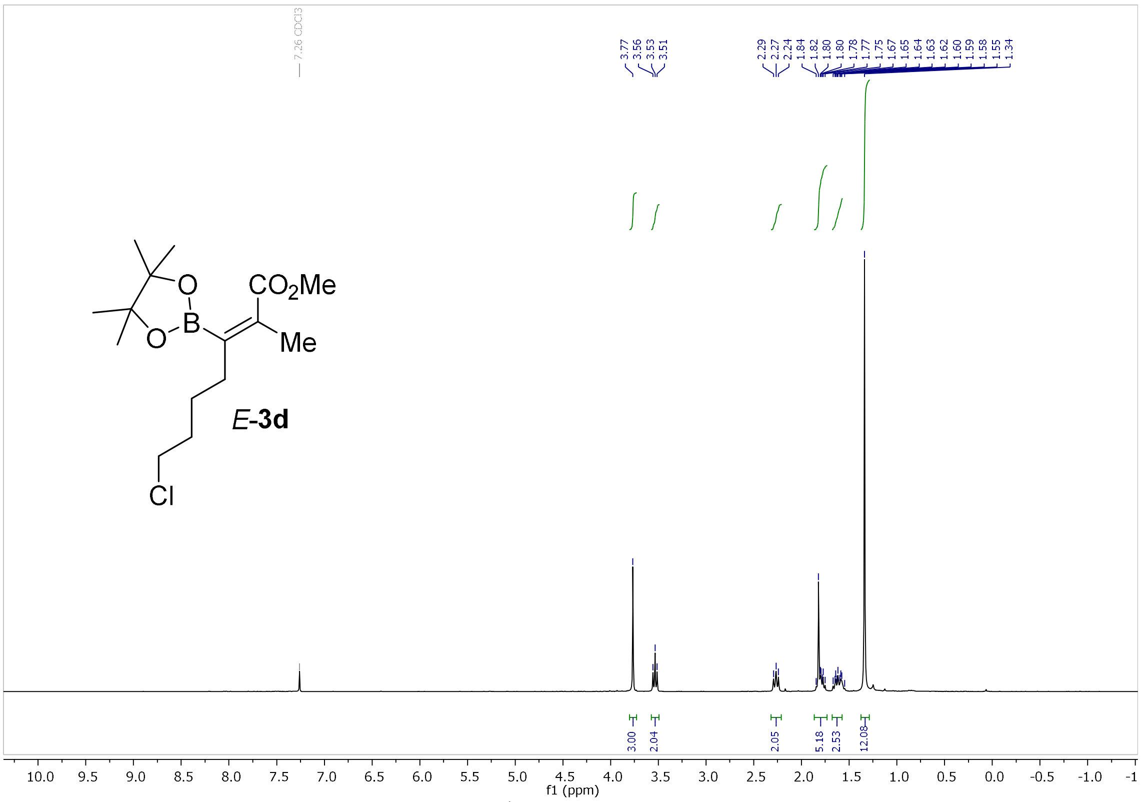

Figure S128. ^{1}H NMR spectrum of *E*-**3d.**

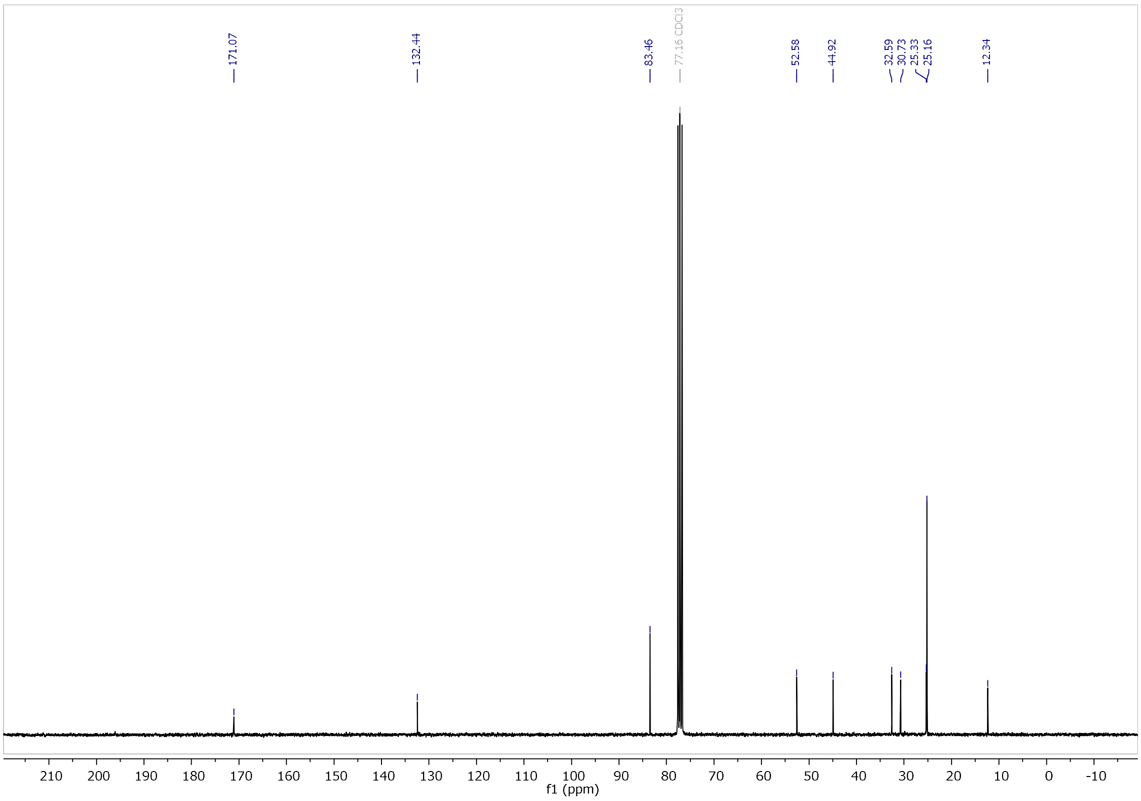

Figure S129. ^{13}C NMR spectrum of *E*-**3d.**

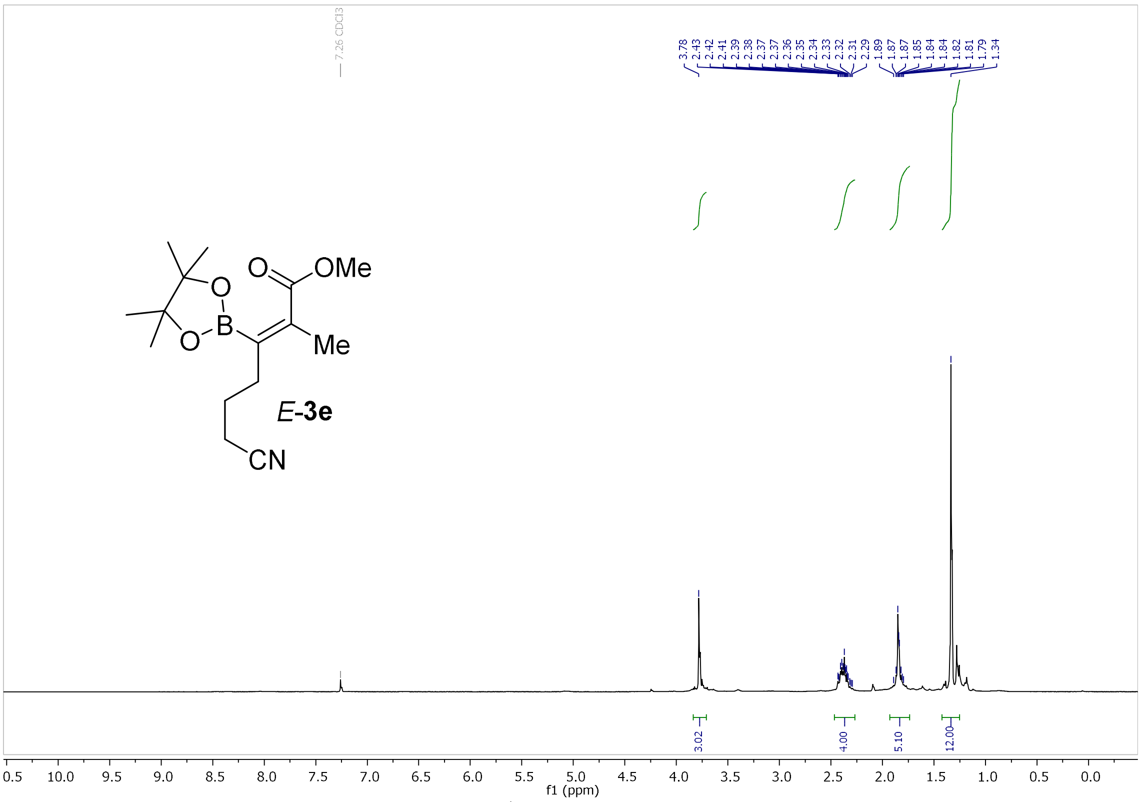

Figure S130. ^{1}H NMR spectrum of *E*-**3e.**

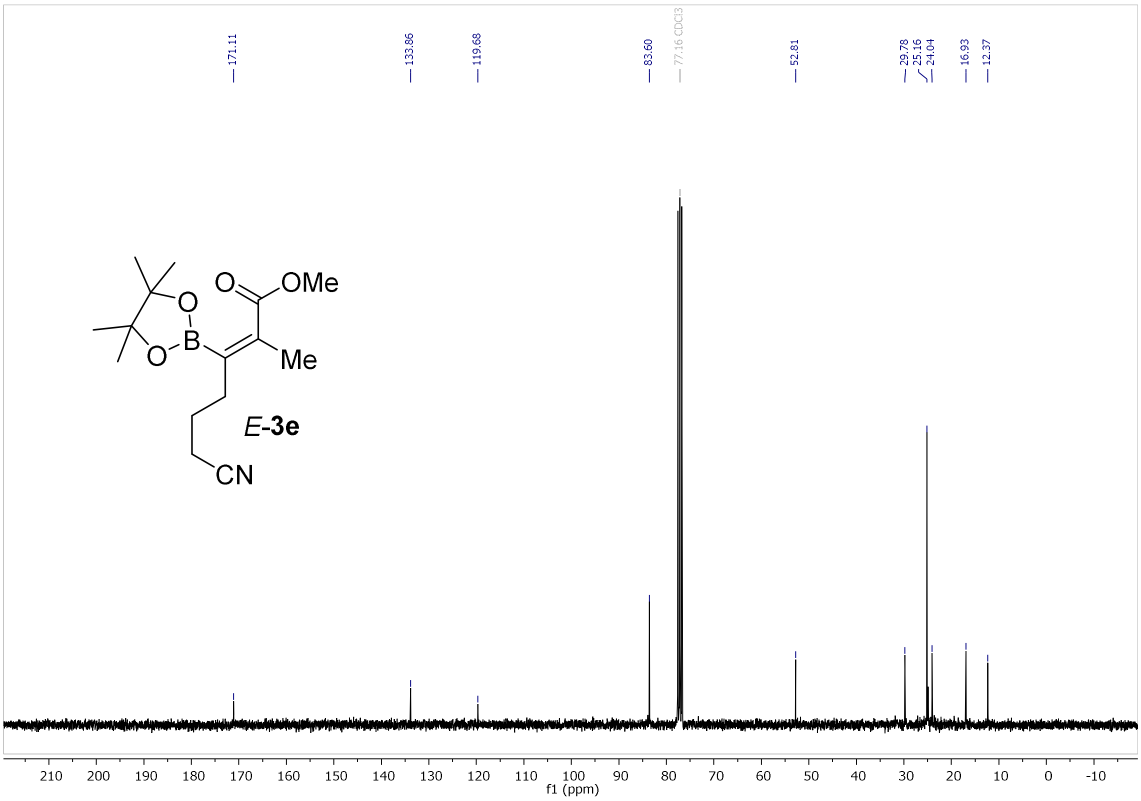

Figure S131. ^{13}C NMR spectrum of *E*-**3e.**

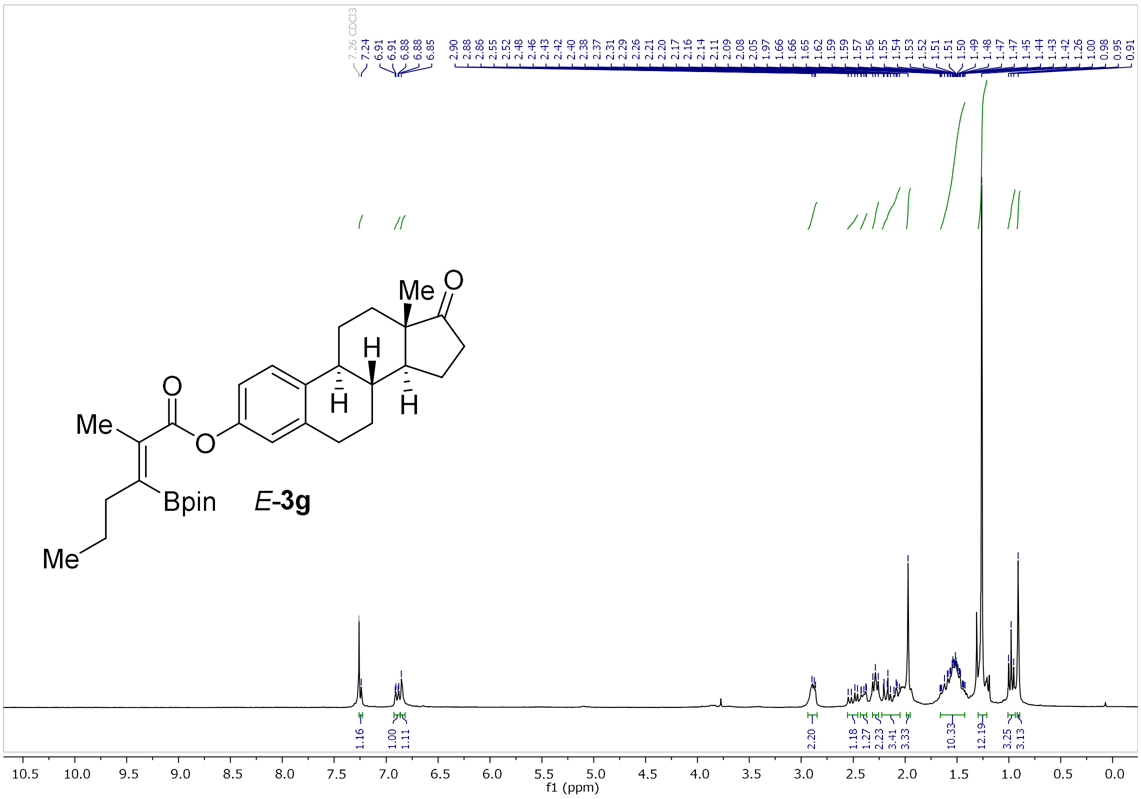

Figure S132. ^{1}H NMR spectrum of *E*-**3g.**

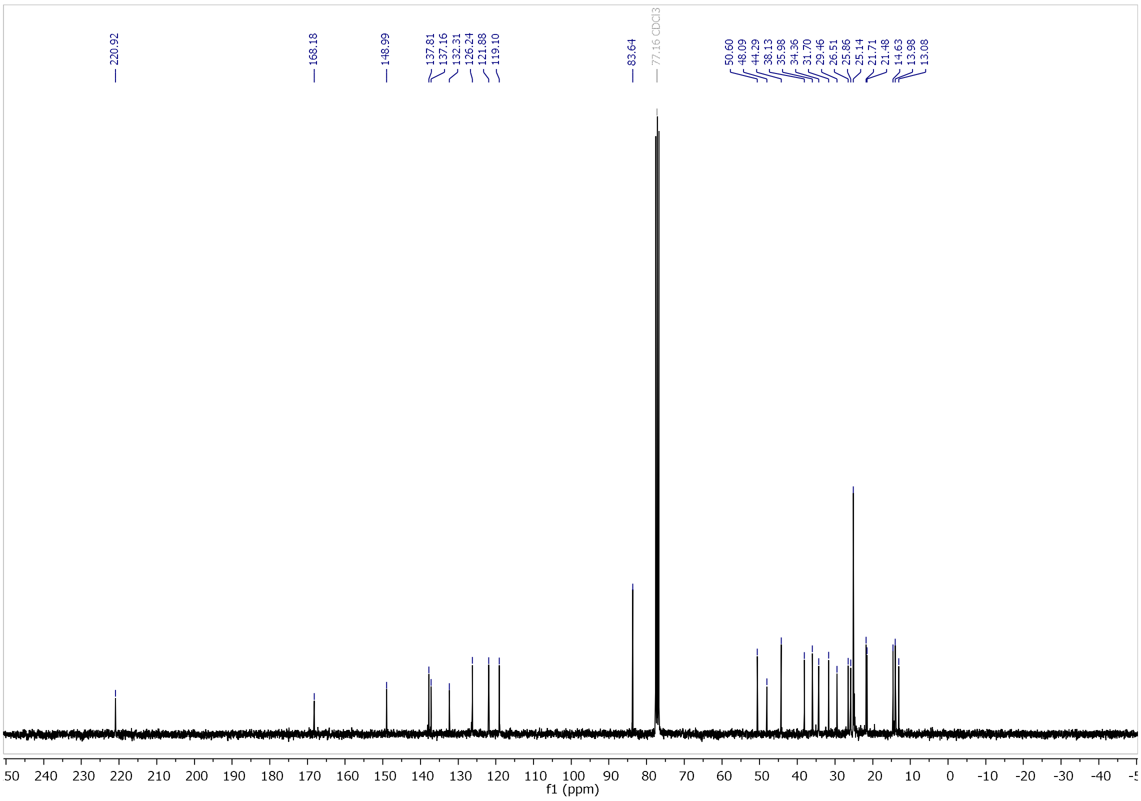

Figure S133. ^{13}C NMR spectrum of *E*-**3g.**

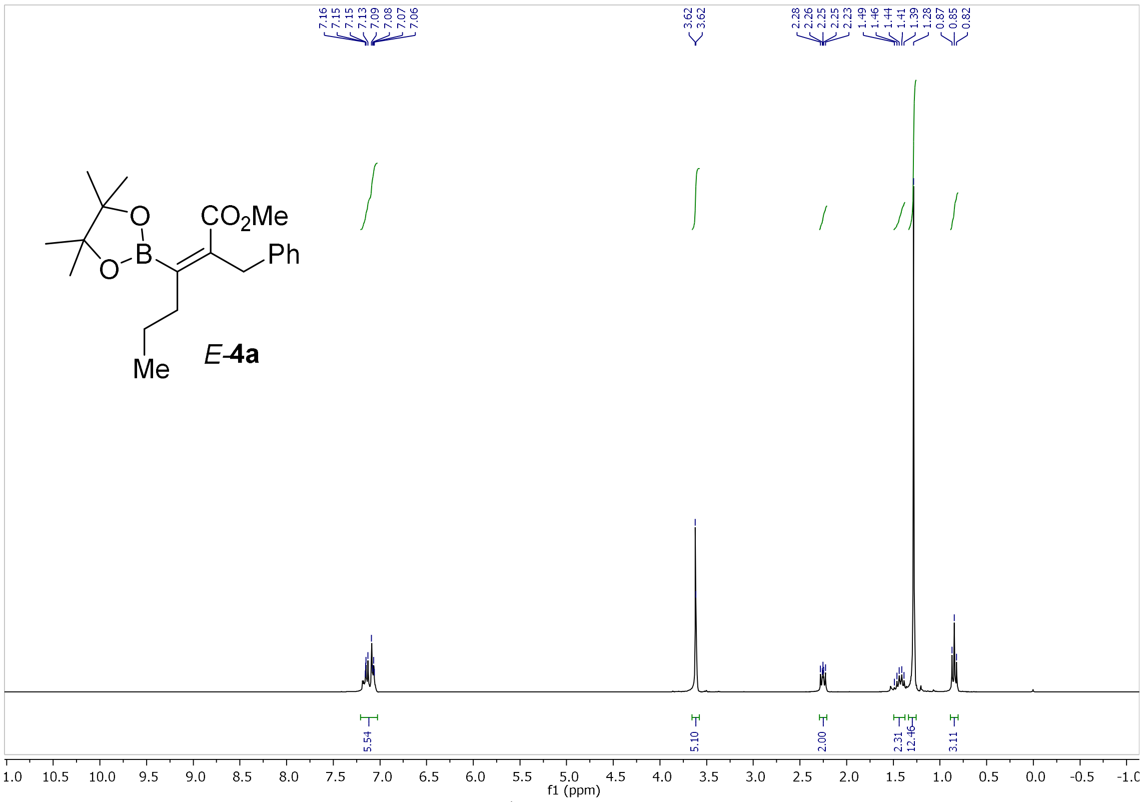

Figure S134. ^{1}H NMR spectrum of *E*-**4a.**

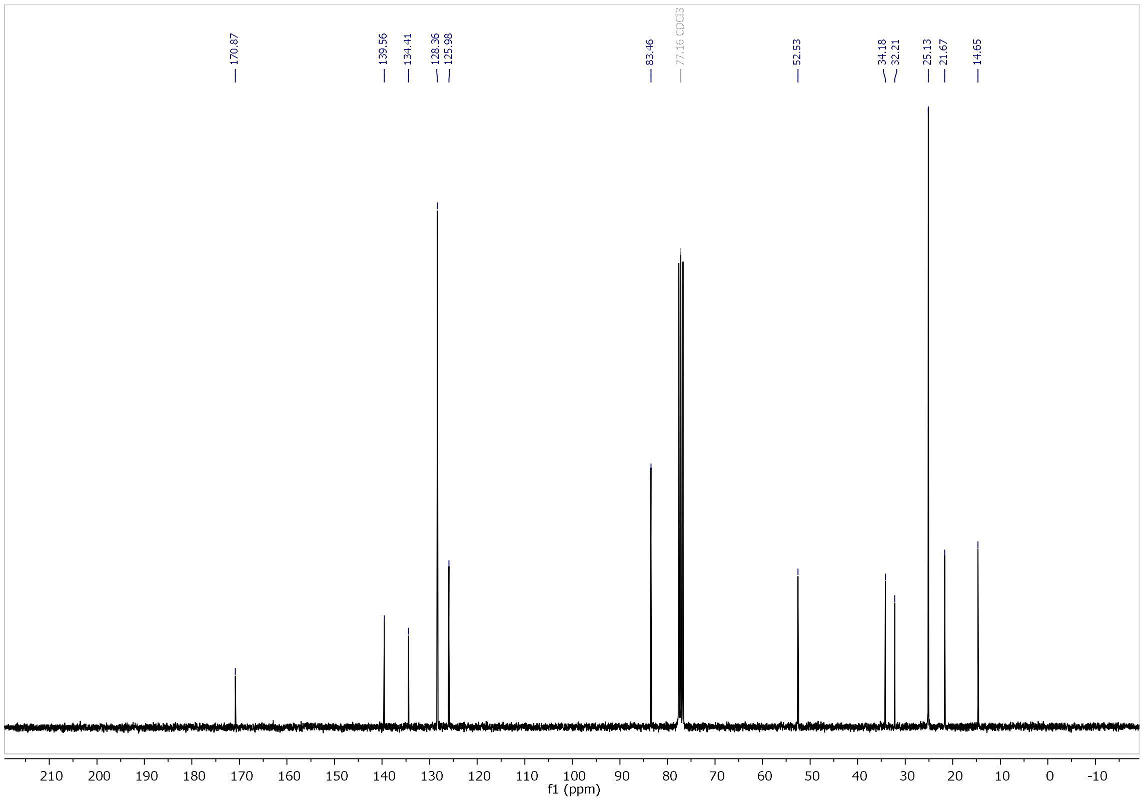

Figure S135. ^{13}C NMR spectrum of *E*-**4a.**

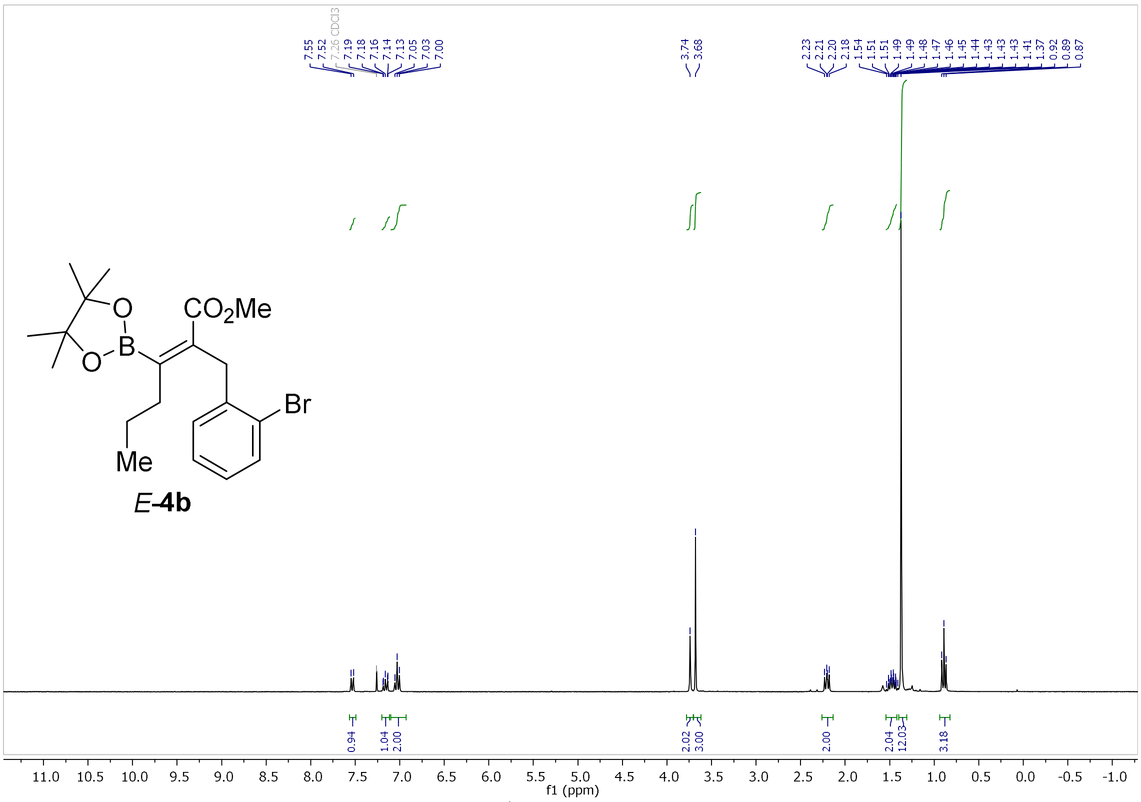

Figure S136. ^{1}H NMR spectrum of *E*-**4b.**

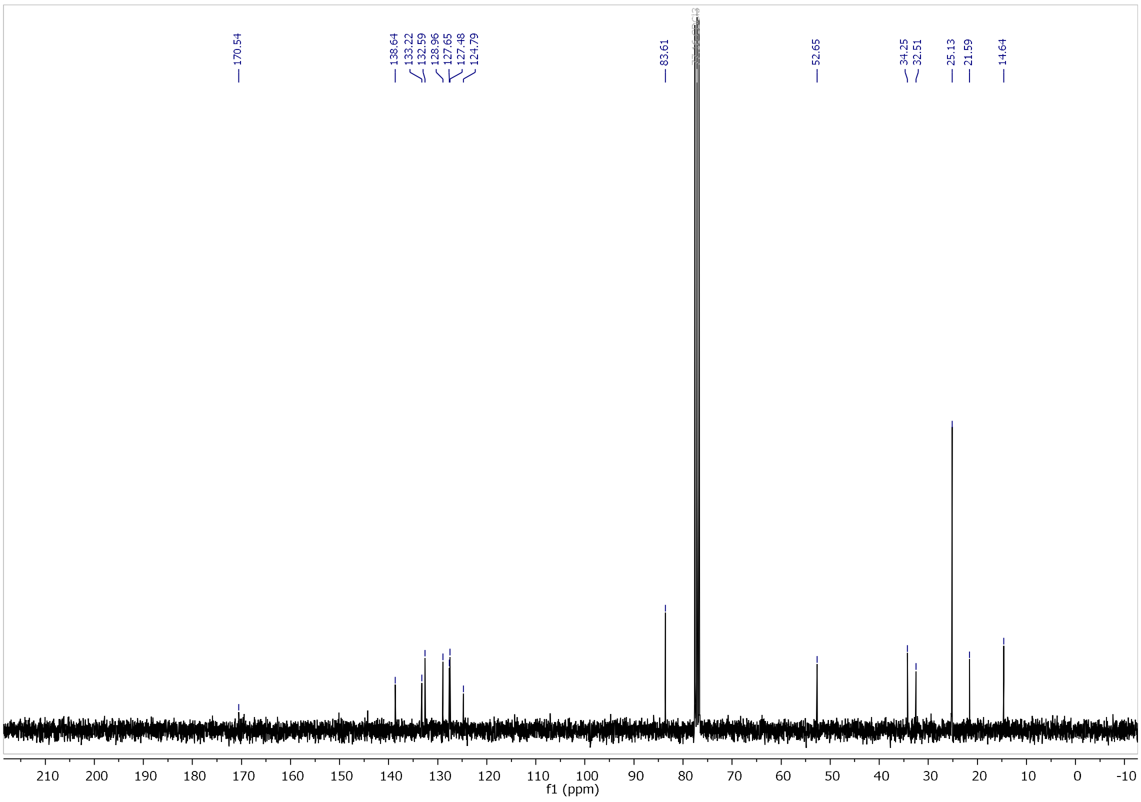

Figure S137. ^{13}C NMR spectrum of *E*-**4b.**

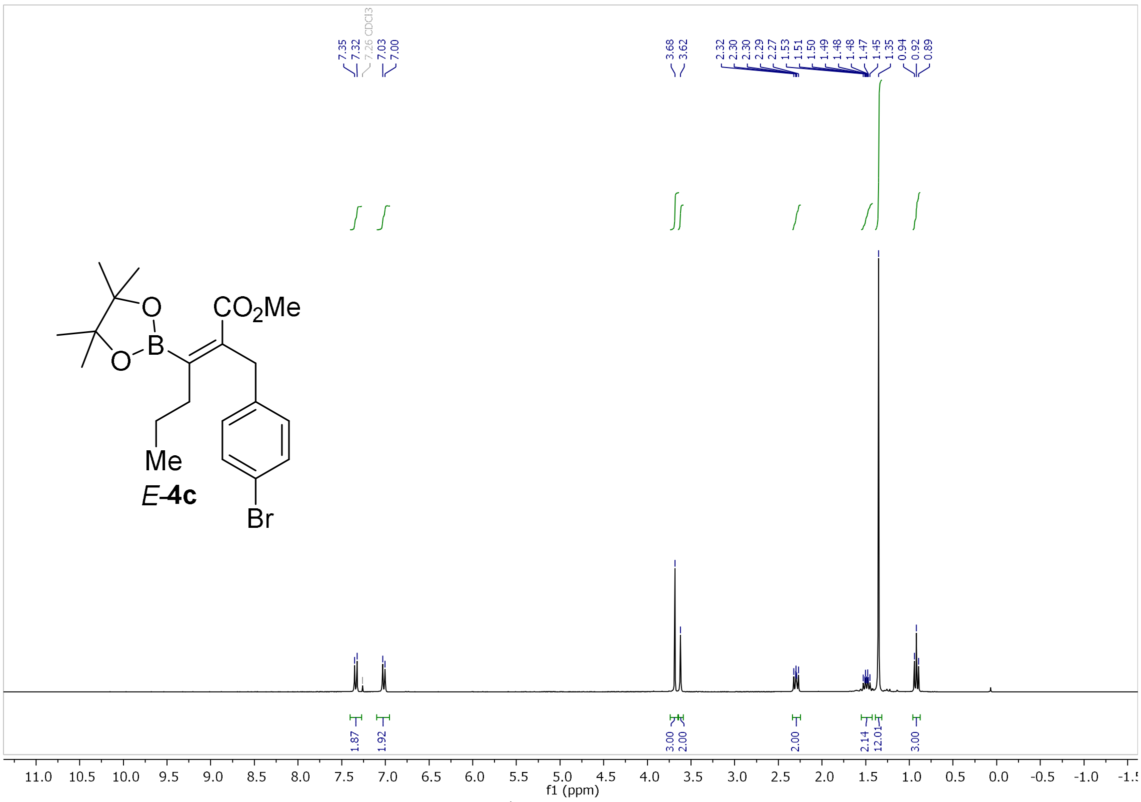

Figure S138. ^{1}H NMR spectrum of *E*-**4c.**

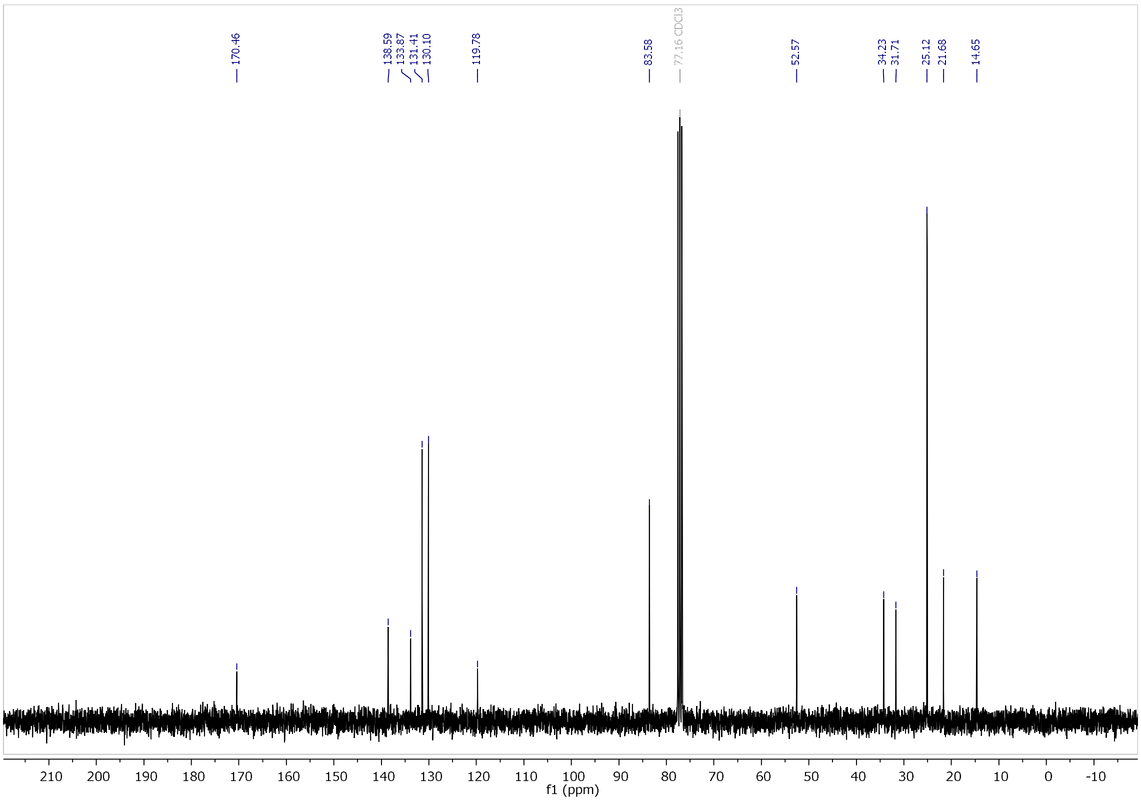

Figure S139. ^{13}C NMR spectrum of *E*-**4c.**

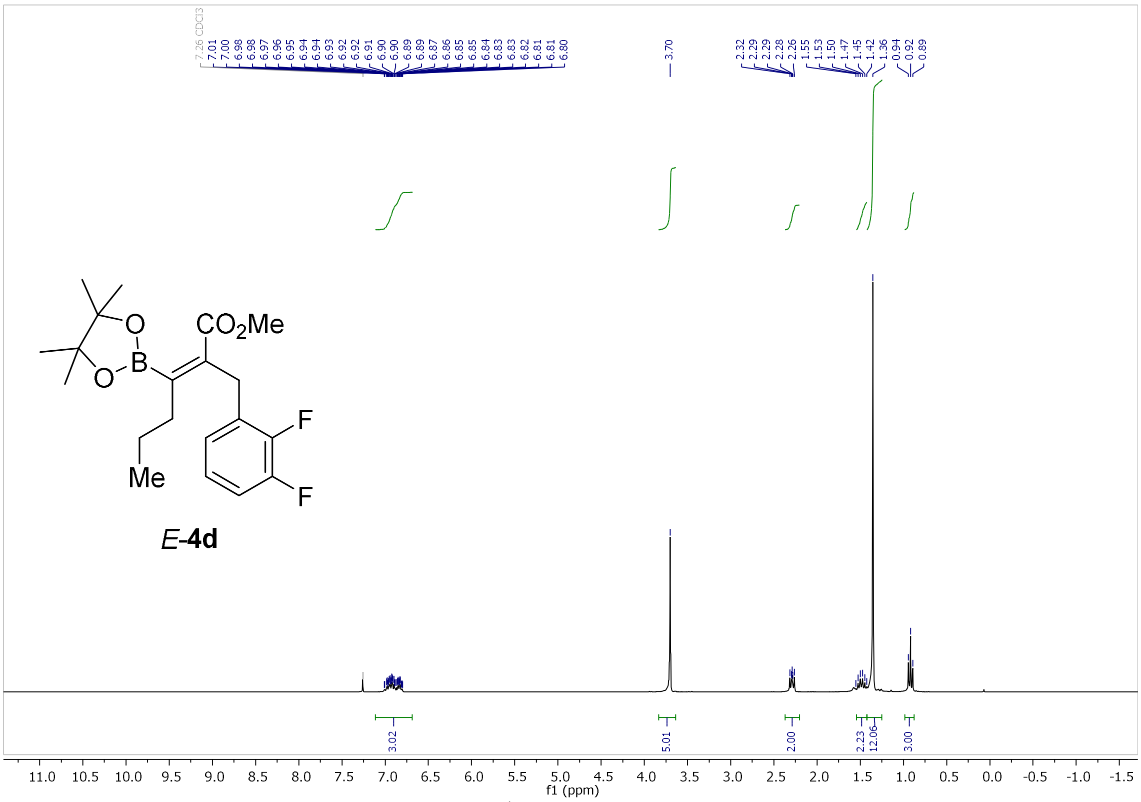

Figure S140. ^{1}H NMR spectrum of *E*-**4d.**

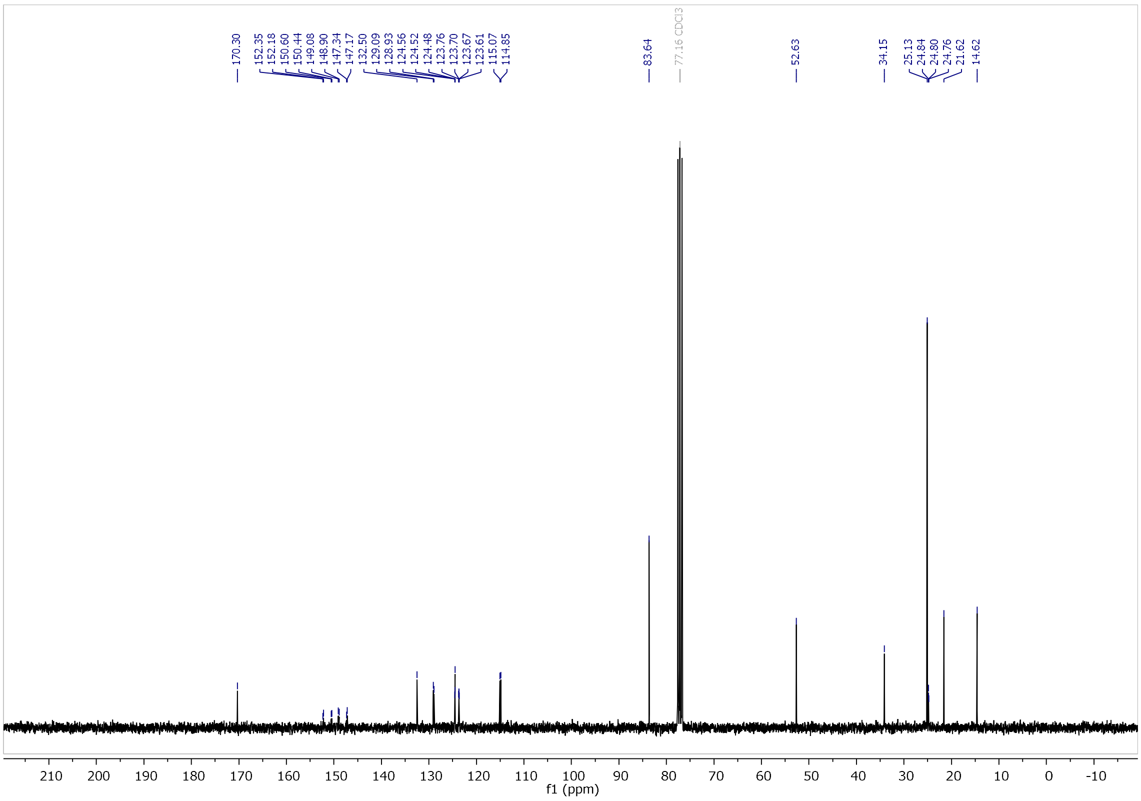

Figure S141. ^{13}C NMR spectrum of *E*-**4d.**

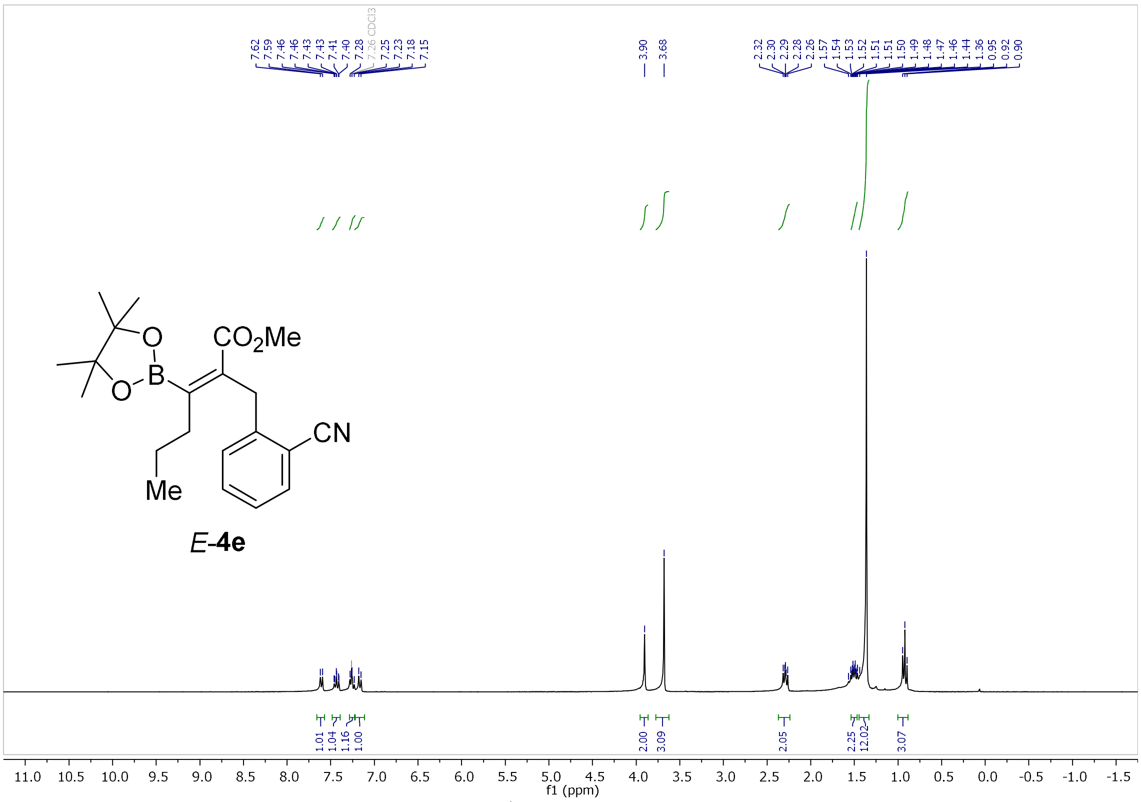

Figure S142. ^{1}H NMR spectrum of *E*-**4e.**

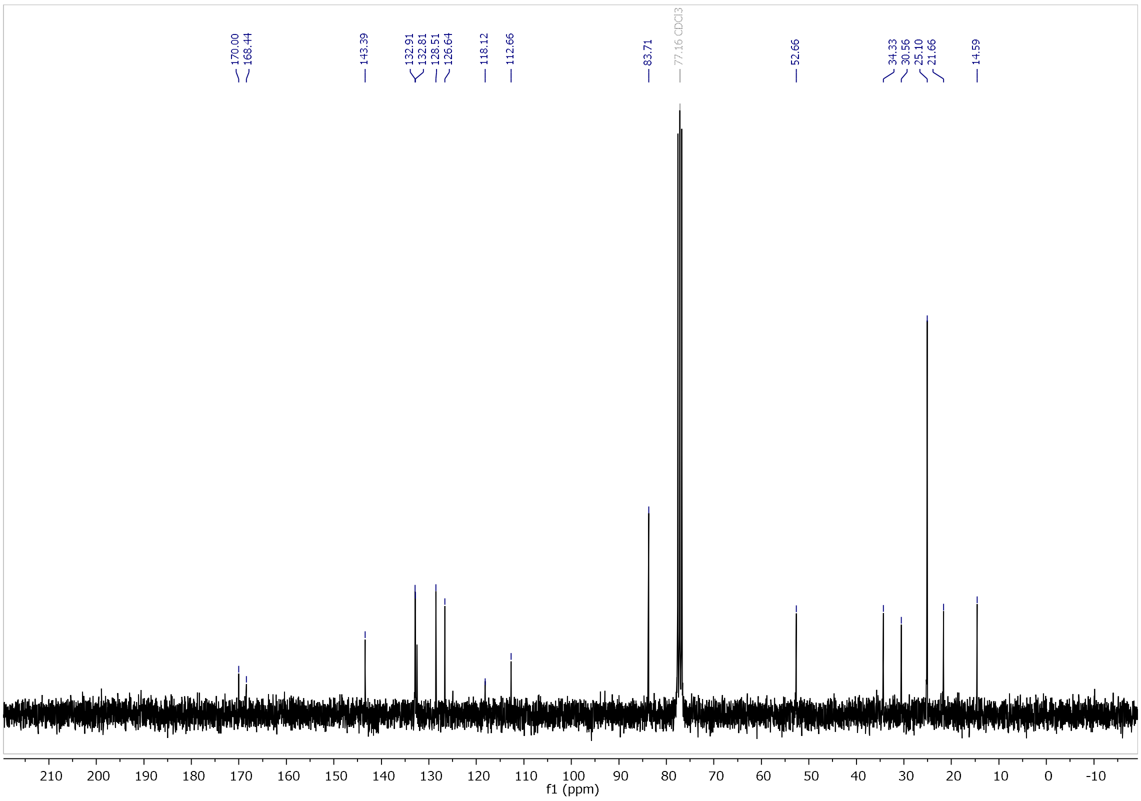

Figure S143. ^{13}C NMR spectrum of *E*-**4e.**

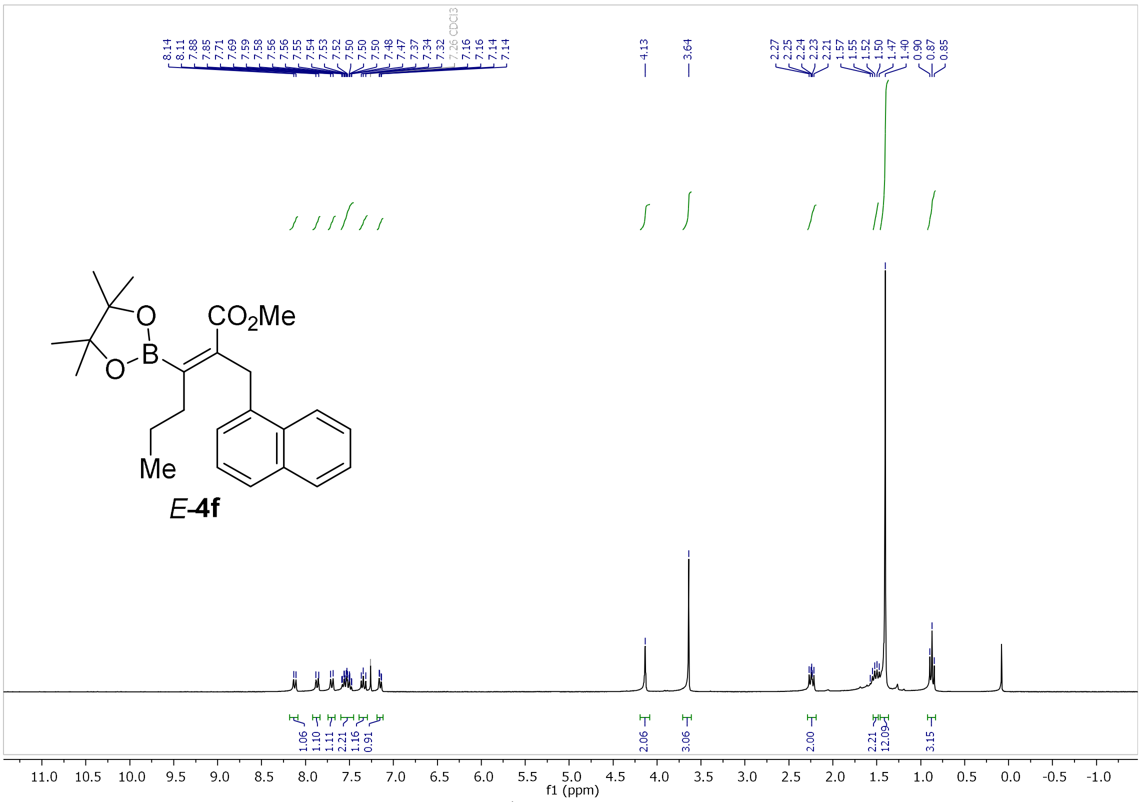

Figure S144. ^{1}H NMR spectrum of *E*-**4f.**

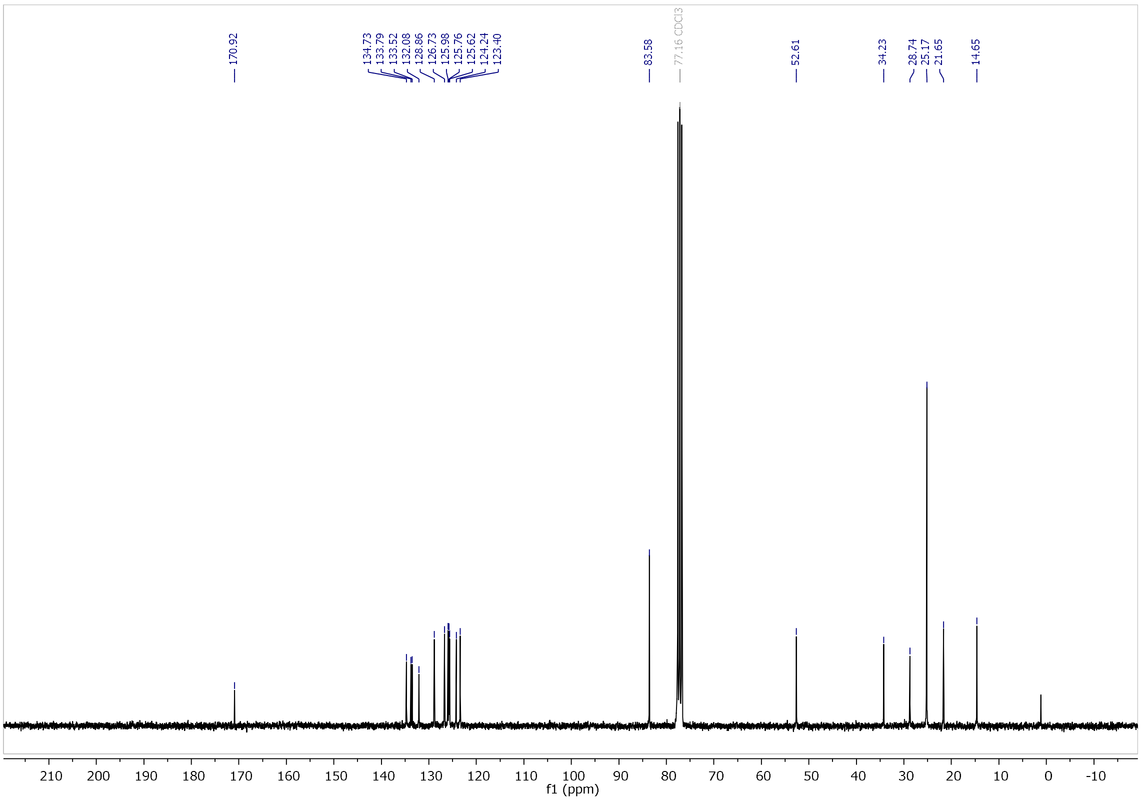

Figure S145. ^{13}C NMR spectrum of *E*-**4f.**

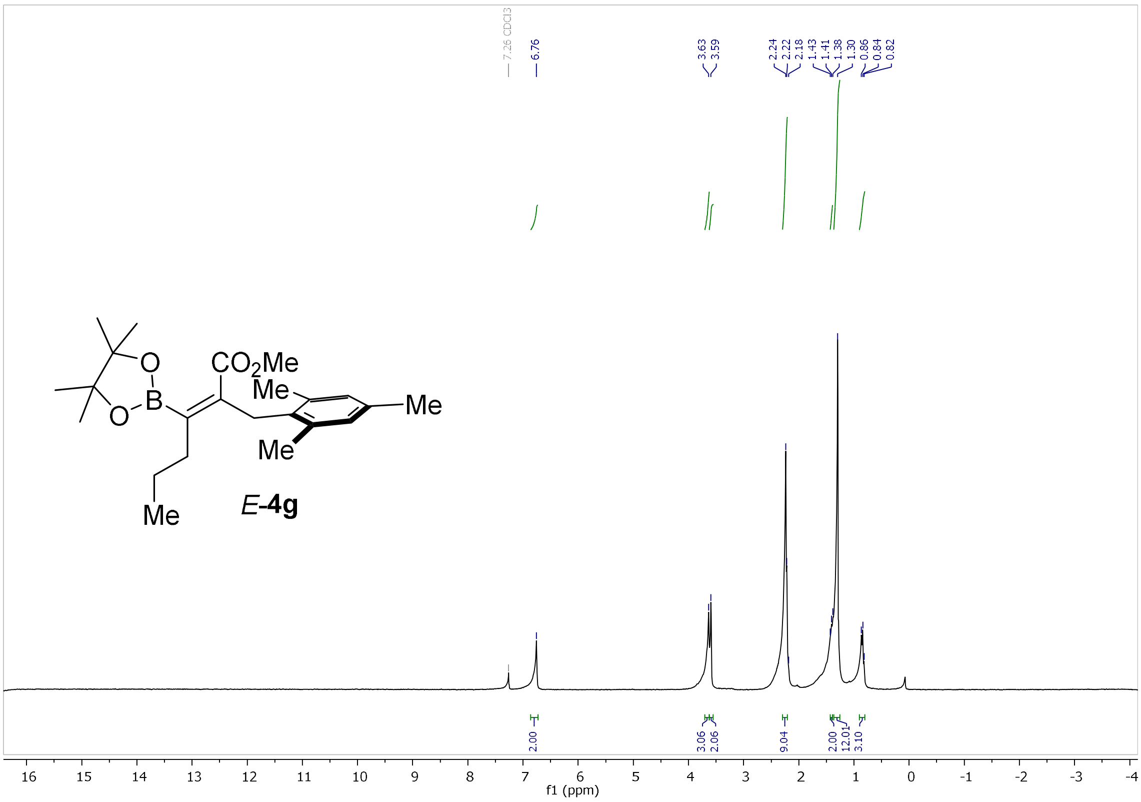

Figure S146. ^{1}H NMR spectrum of *E*-**4g.**

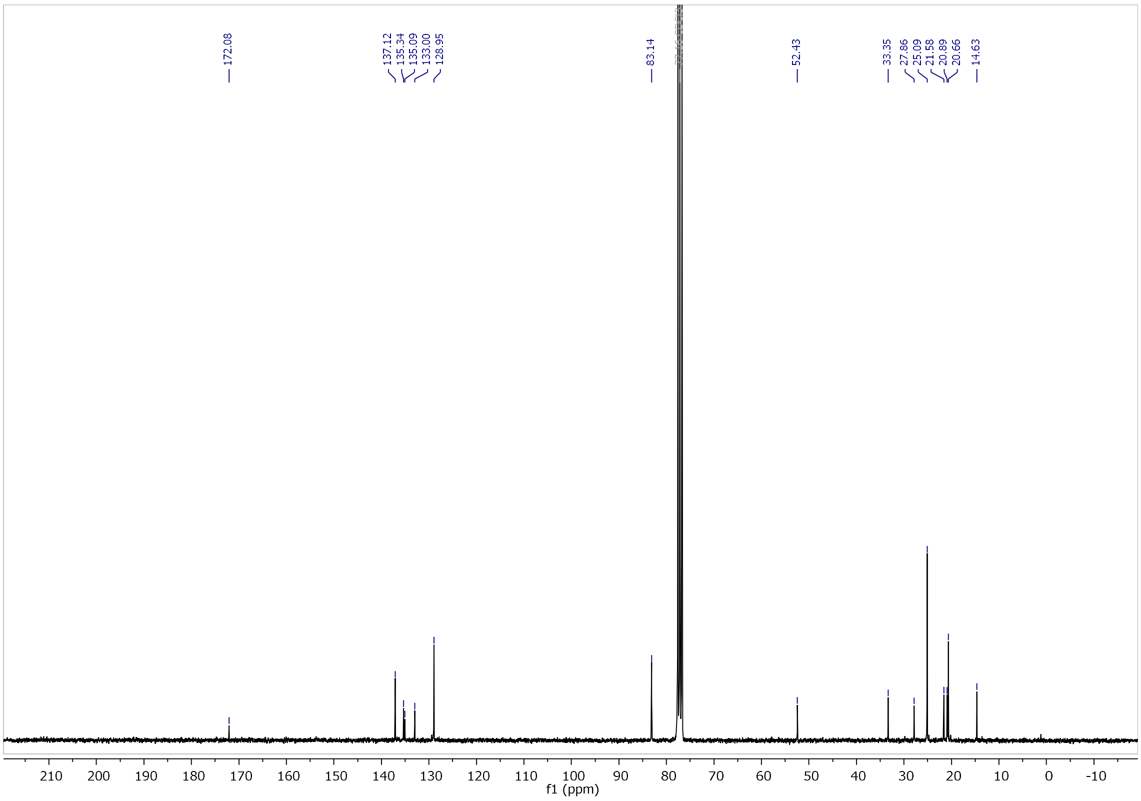

Figure S147. ^{13}C NMR spectrum of *E*-**4g.**

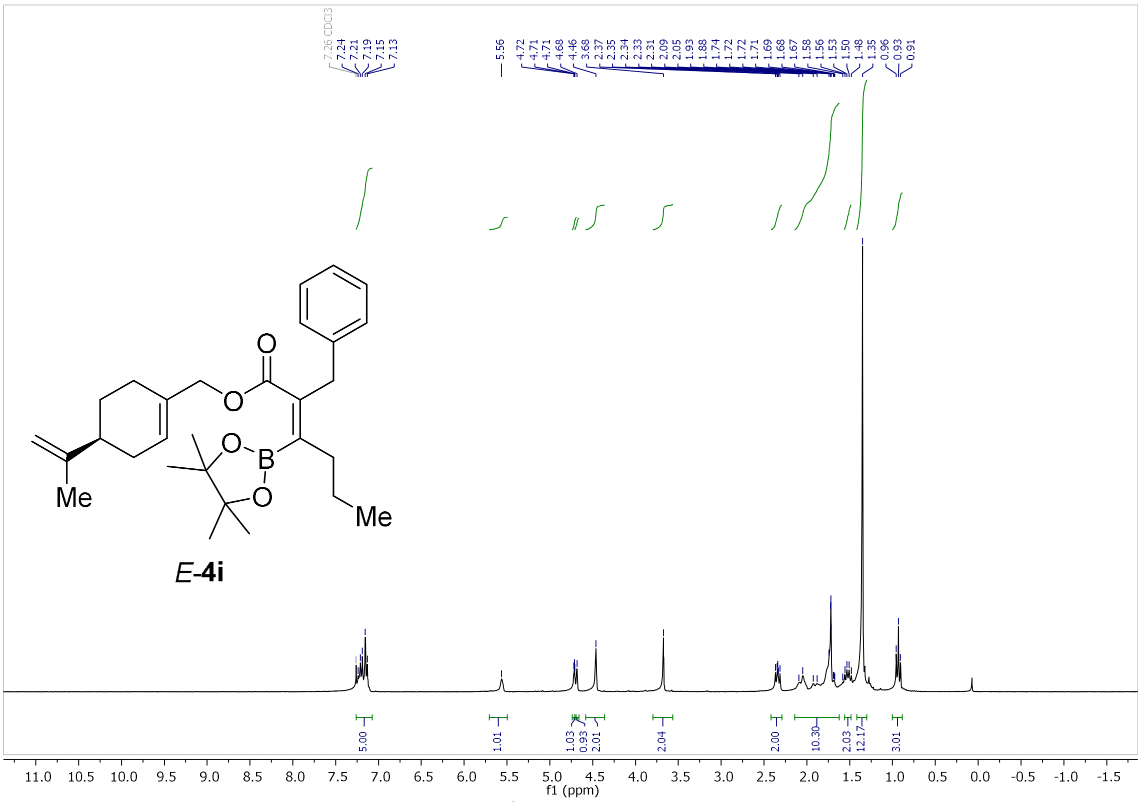

Figure S148. ^{1}H NMR spectrum of *E*-**4i.**

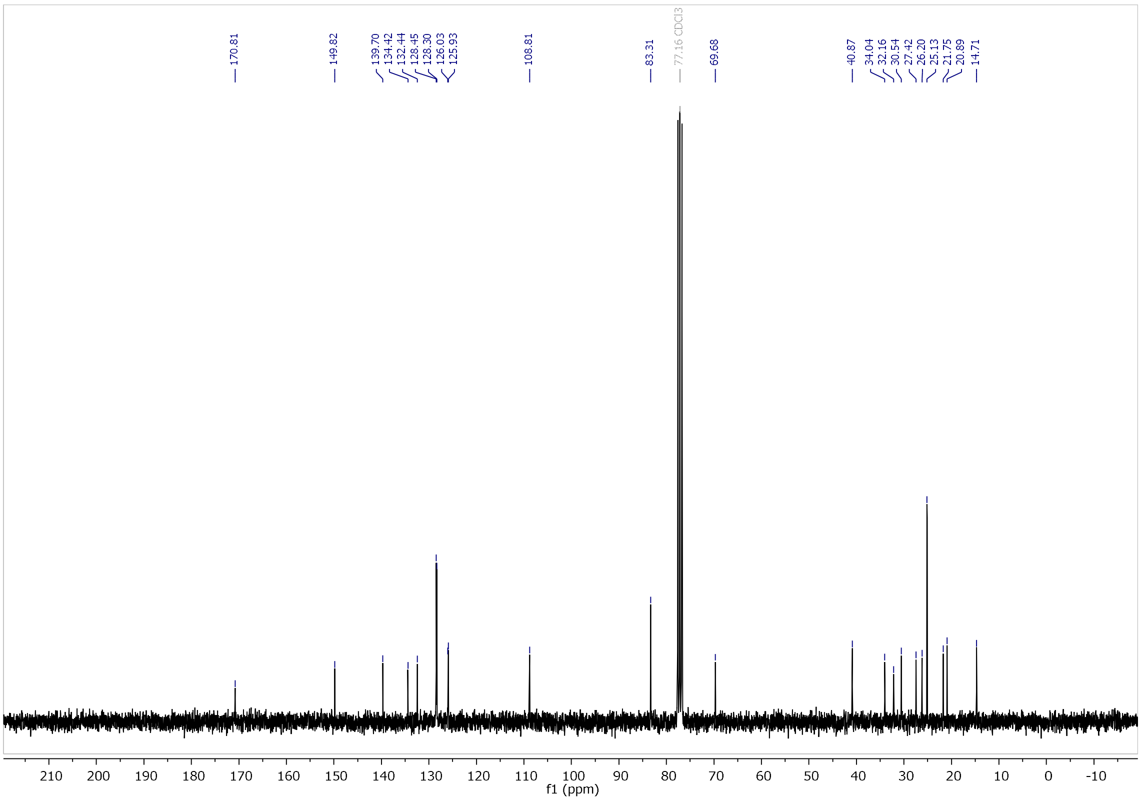

Figure S149. ^{13}C NMR spectrum of *E*-**4i.**

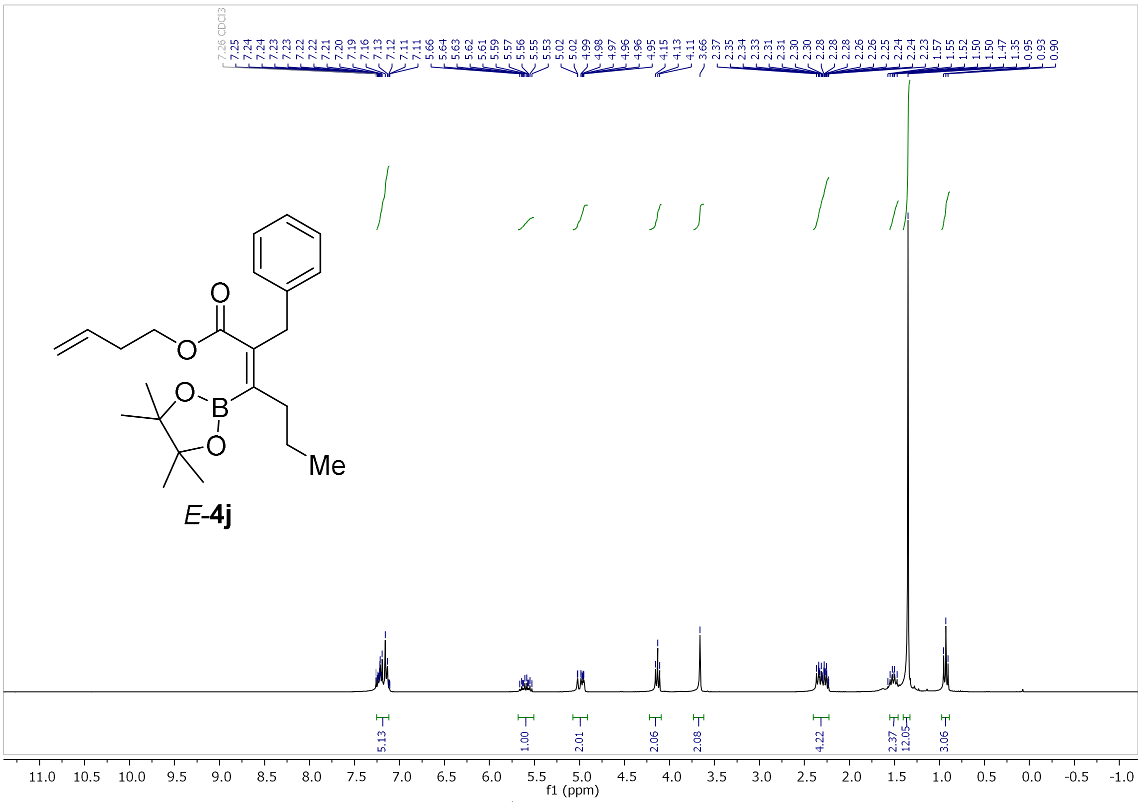

Figure S150. ^{1}H NMR spectrum of *E*-**4j.**

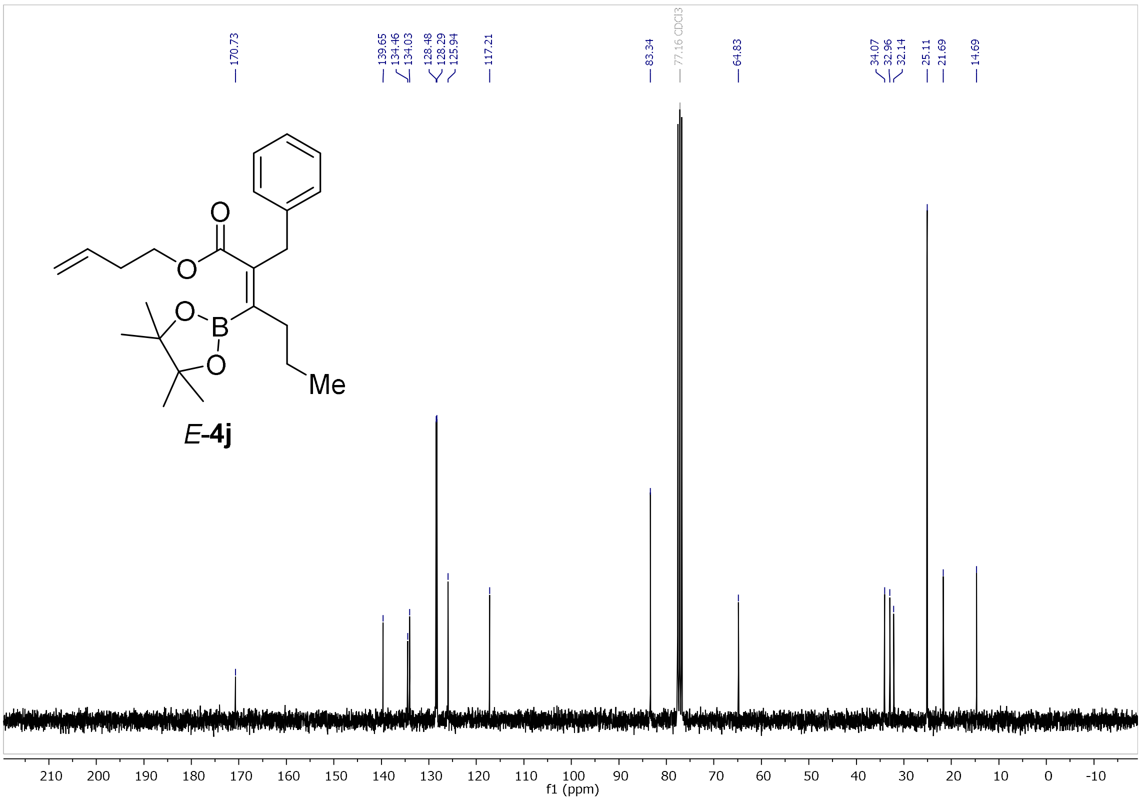

Figure S151. ^{13}C NMR spectrum of *E*-**4j.**

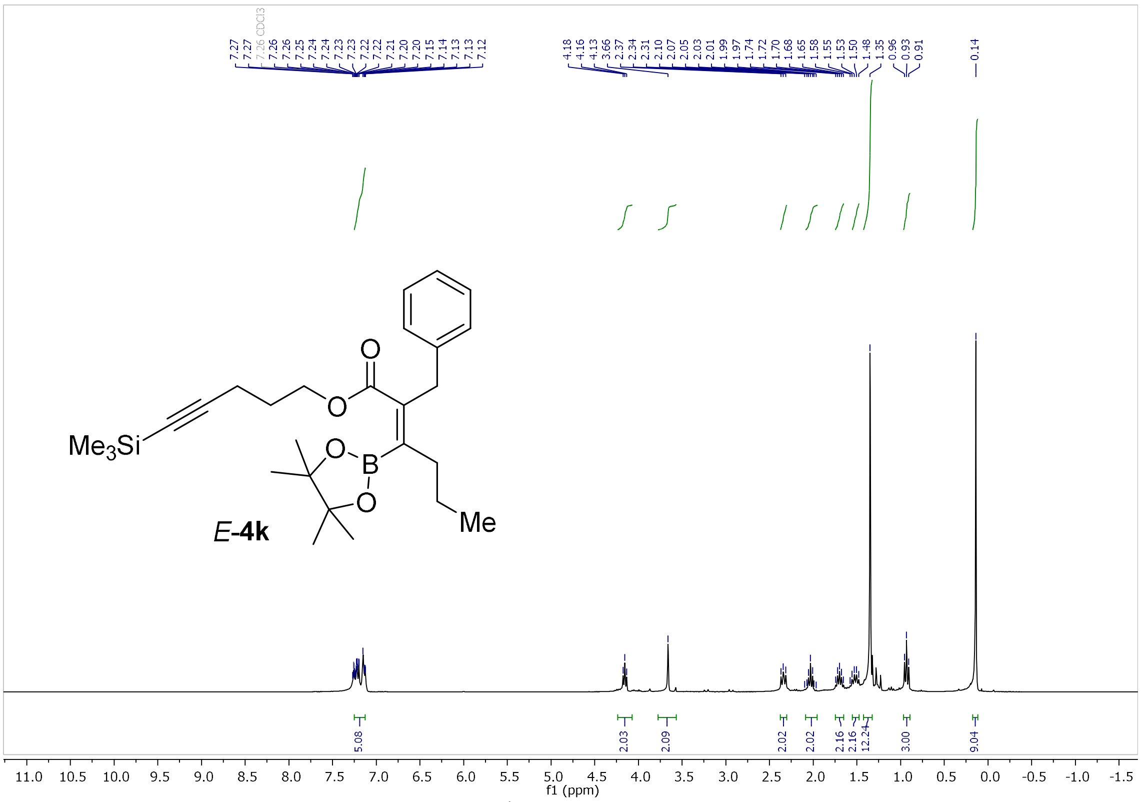

Figure S152. ^{1}H NMR spectrum of *E*-**4k.**

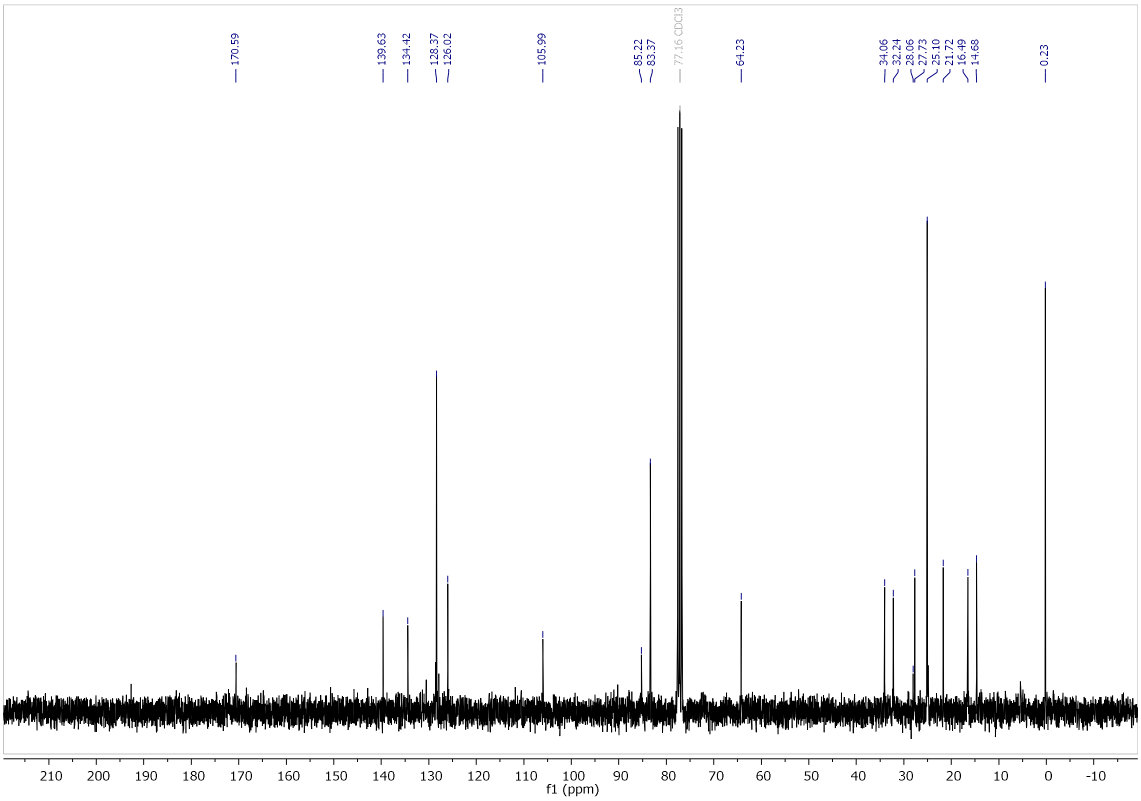

Figure S153. ^{13}C NMR spectrum of *E*-**4k.**

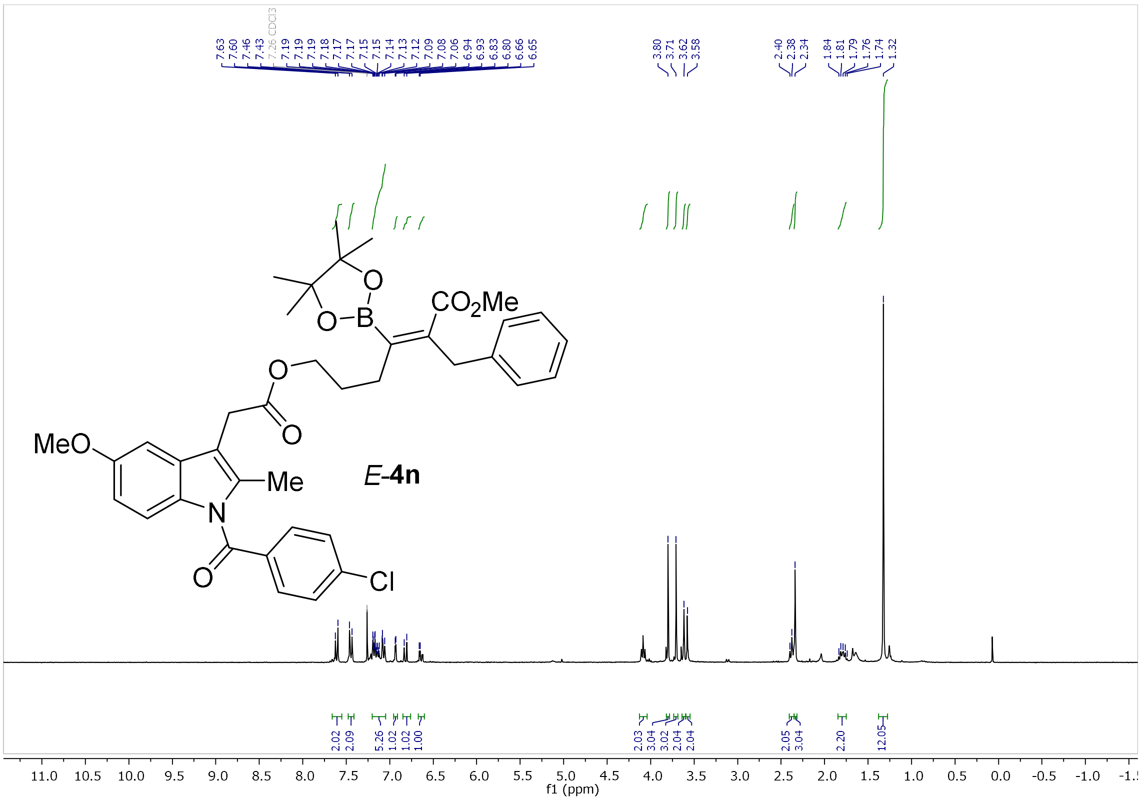

Figure S154. ^{1}H NMR spectrum of *E*-**4n.**

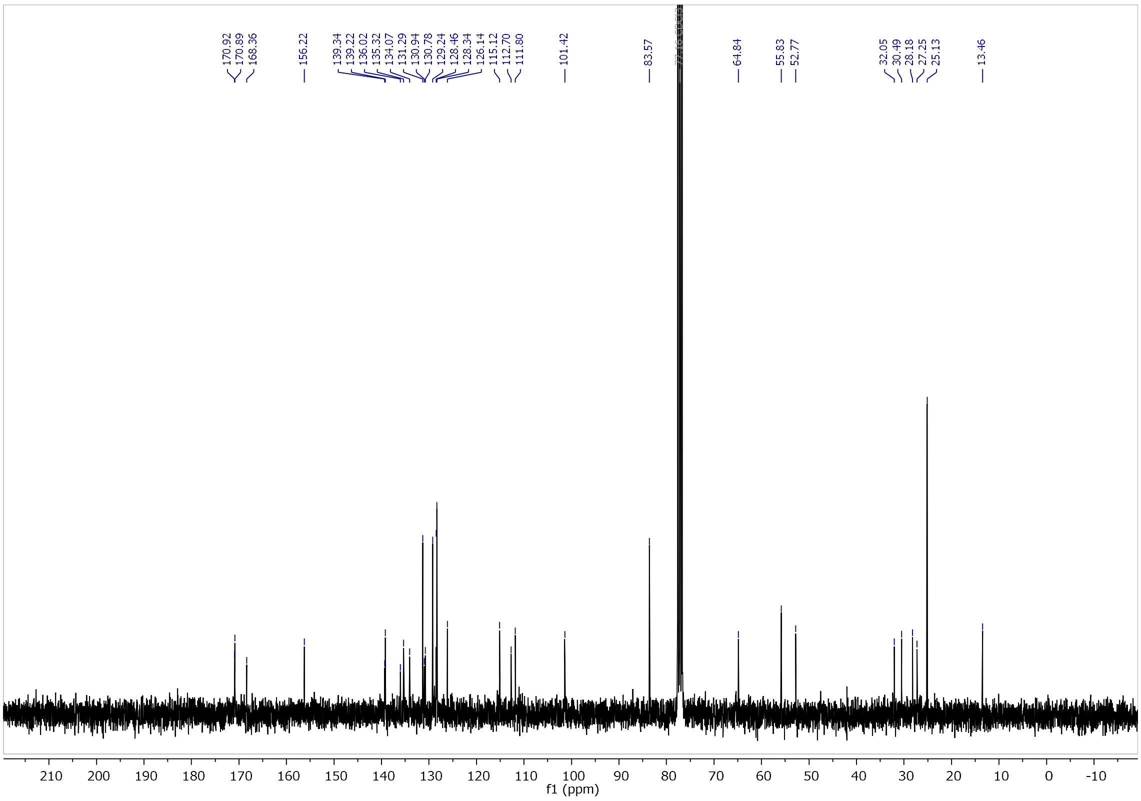

Figure S155. ^{13}C NMR spectrum of *E*-**4n.**

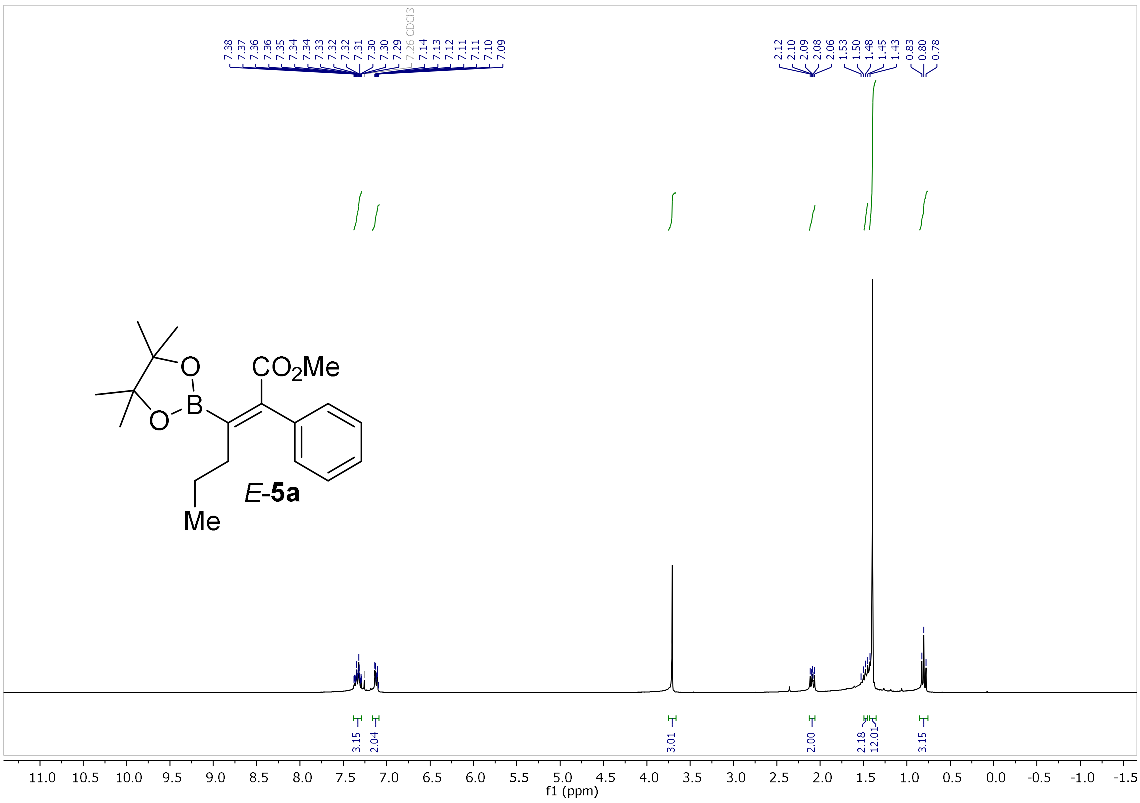

Figure S156. ^{1}H NMR spectrum of *E*-**5a.**

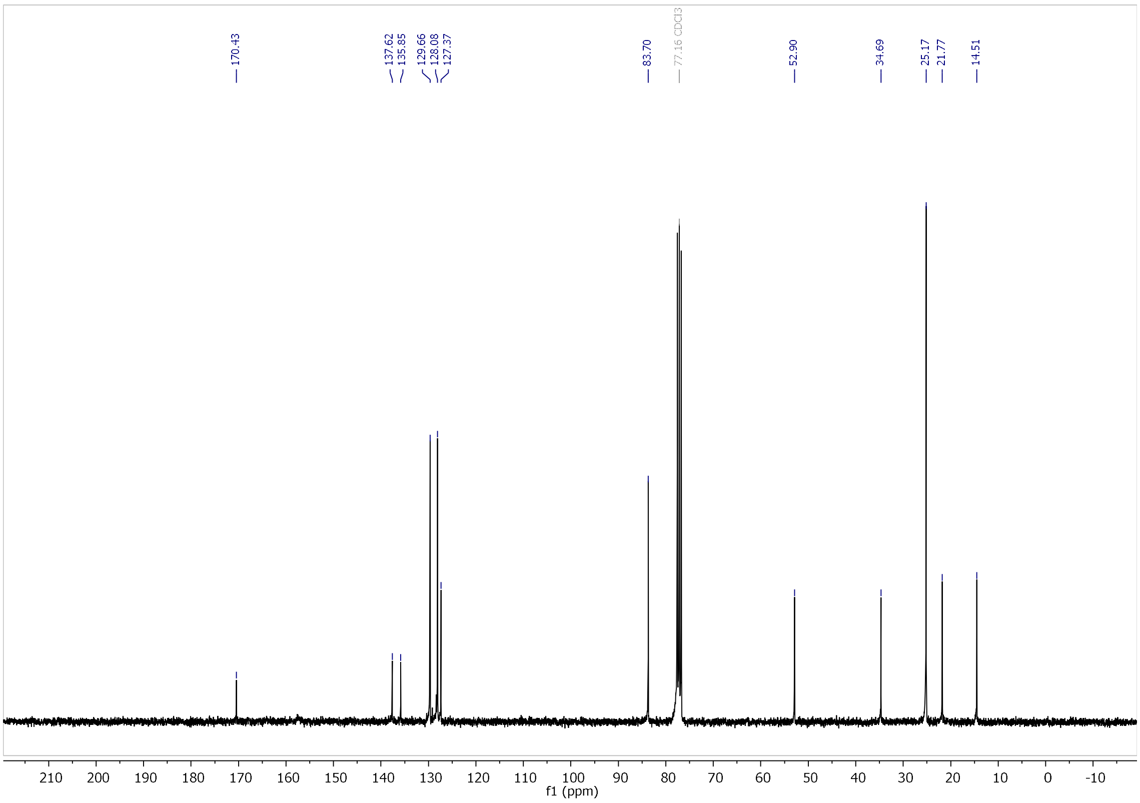

Figure S157. ^{13}C NMR spectrum of *E*-**5a.**

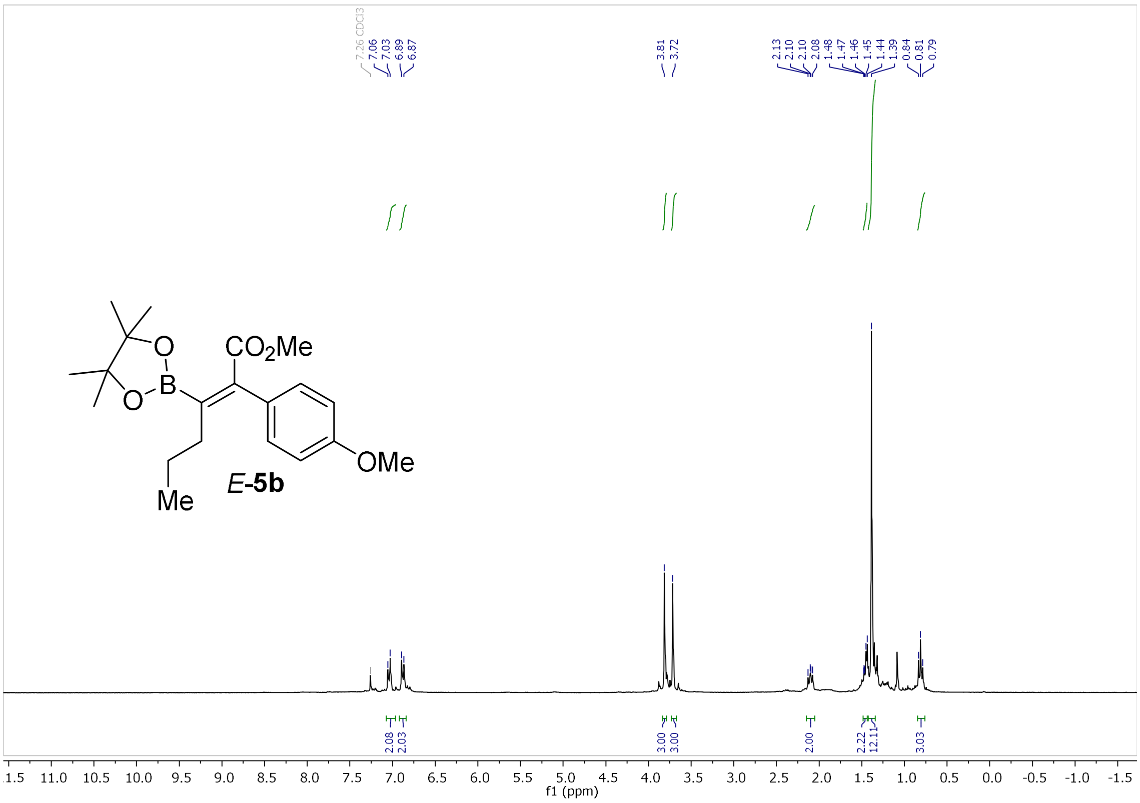

Figure S158. ^{1}H NMR spectrum of *E*-**5b.**

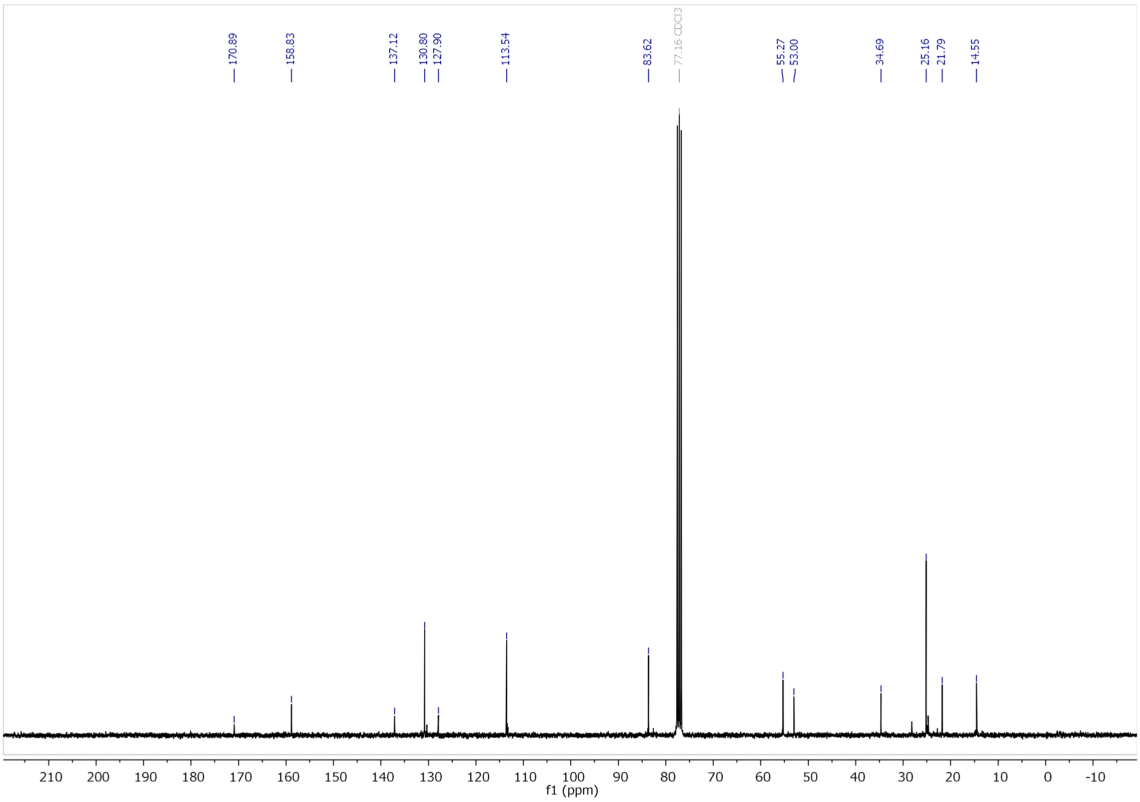

Figure S159. ^{13}C NMR spectrum of *E*-**5b.**

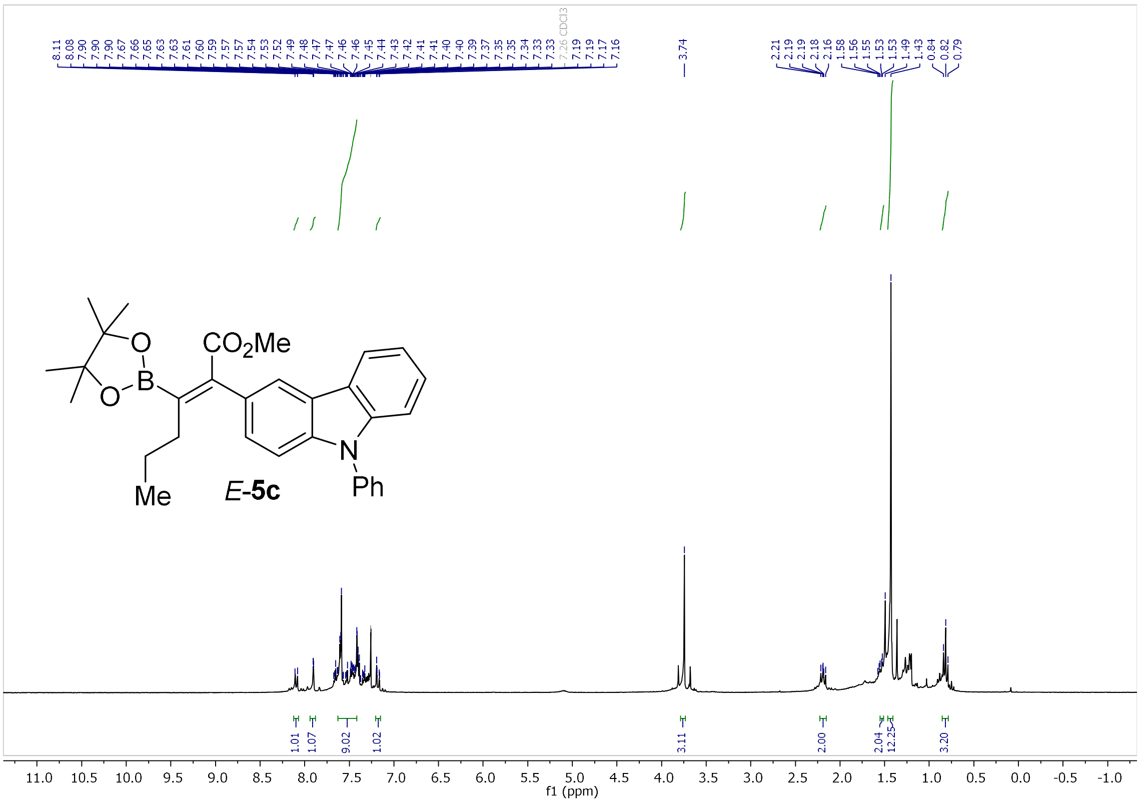

Figure S160. ^{1}H NMR spectrum of *E*-**5c.**

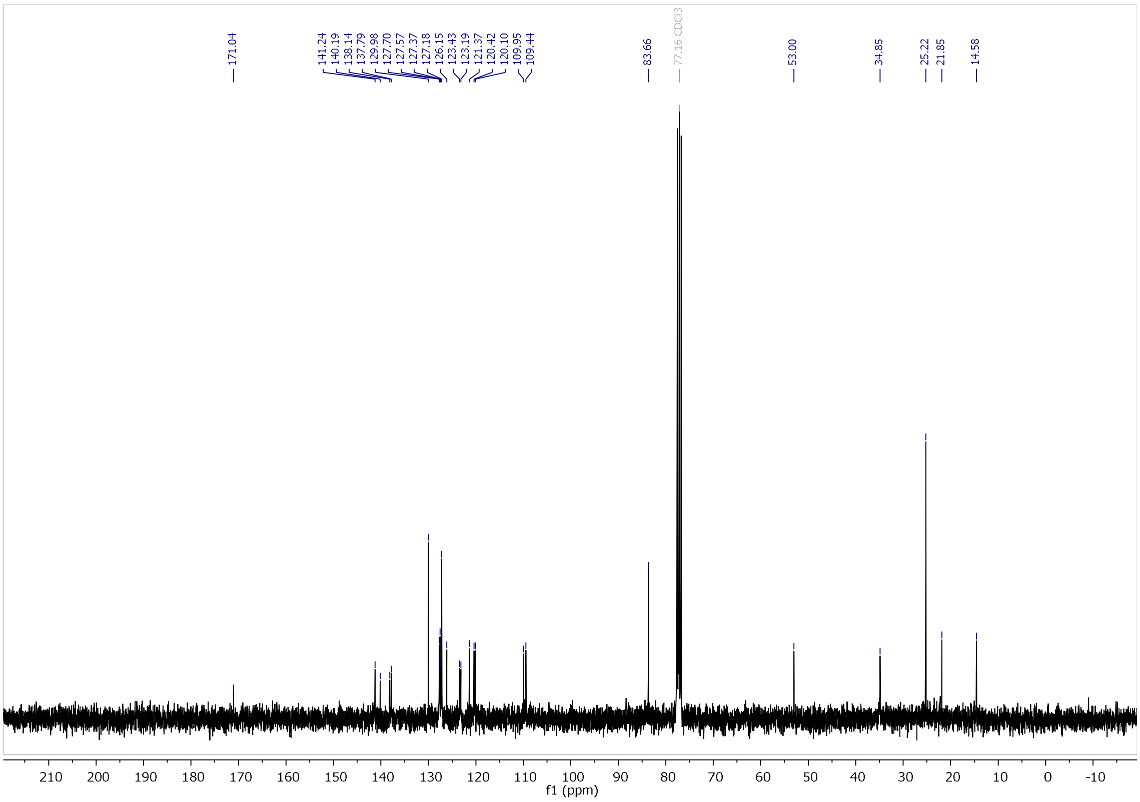

Figure S161. ^{13}C NMR spectrum of *E*-**5c.**

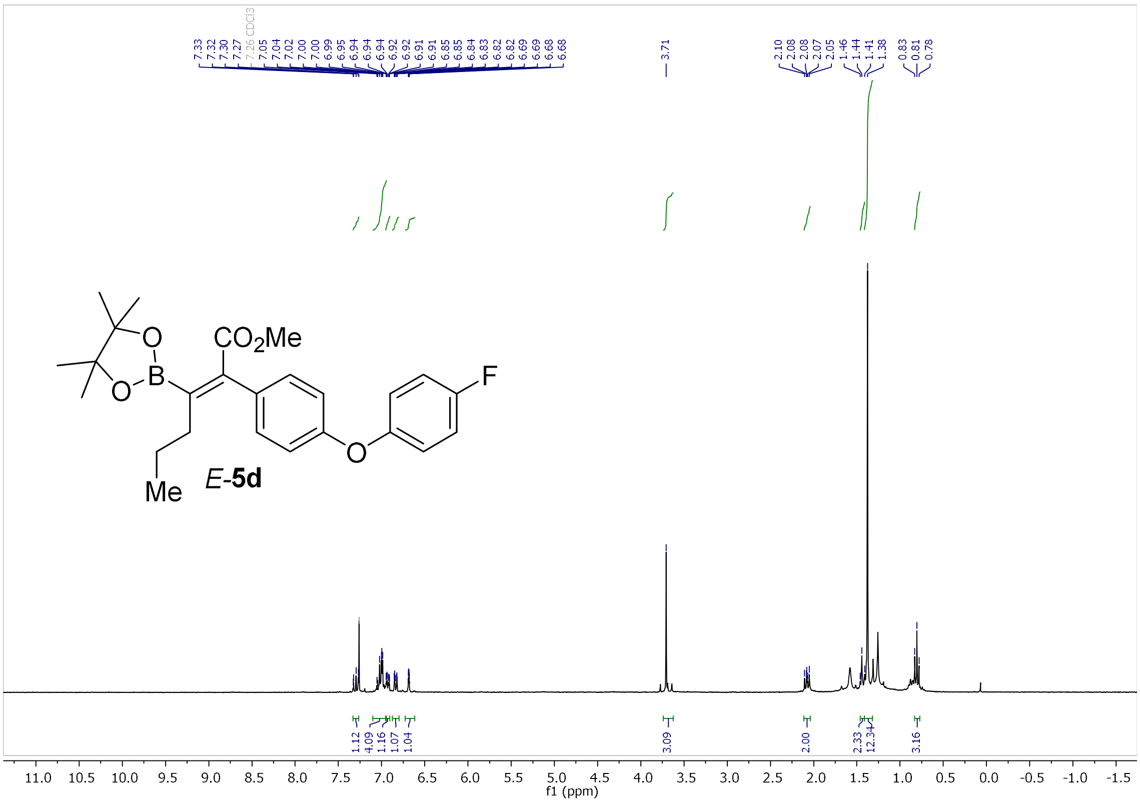

Figure S162. ^{1}H NMR spectrum of *E*-**5d.**

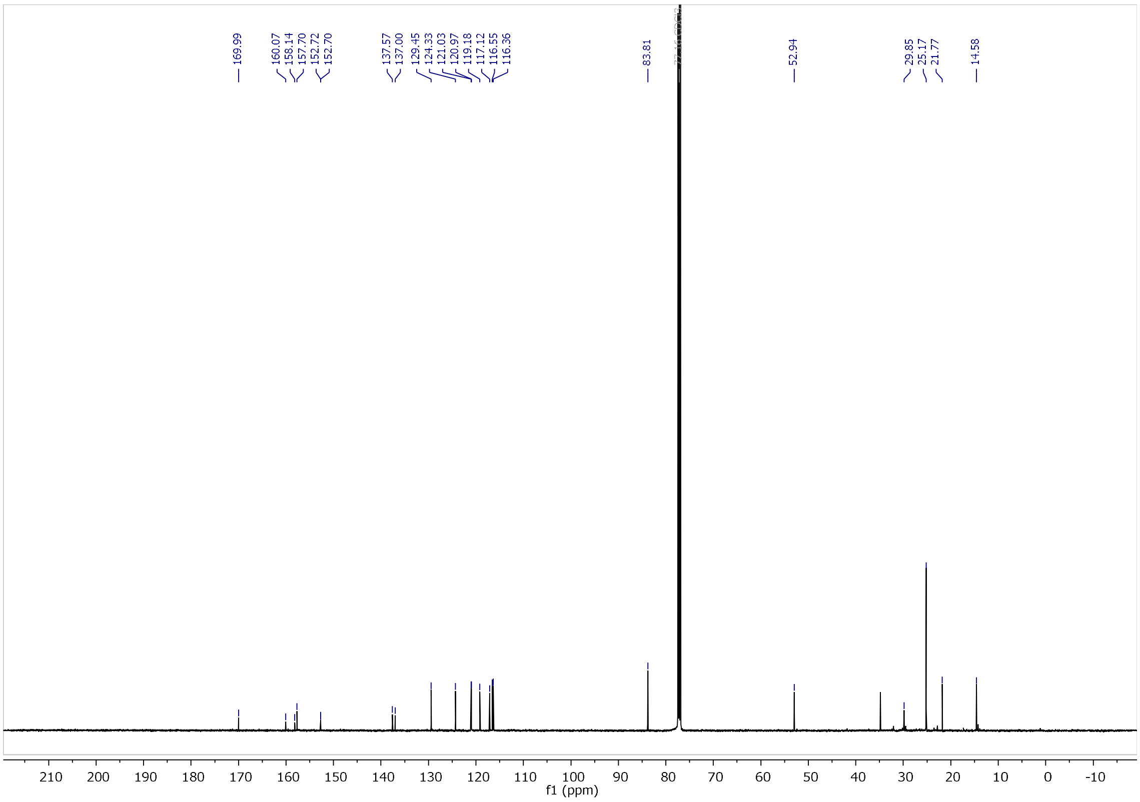

Figure S163. ^{13}C NMR spectrum of *E*-**5d.**

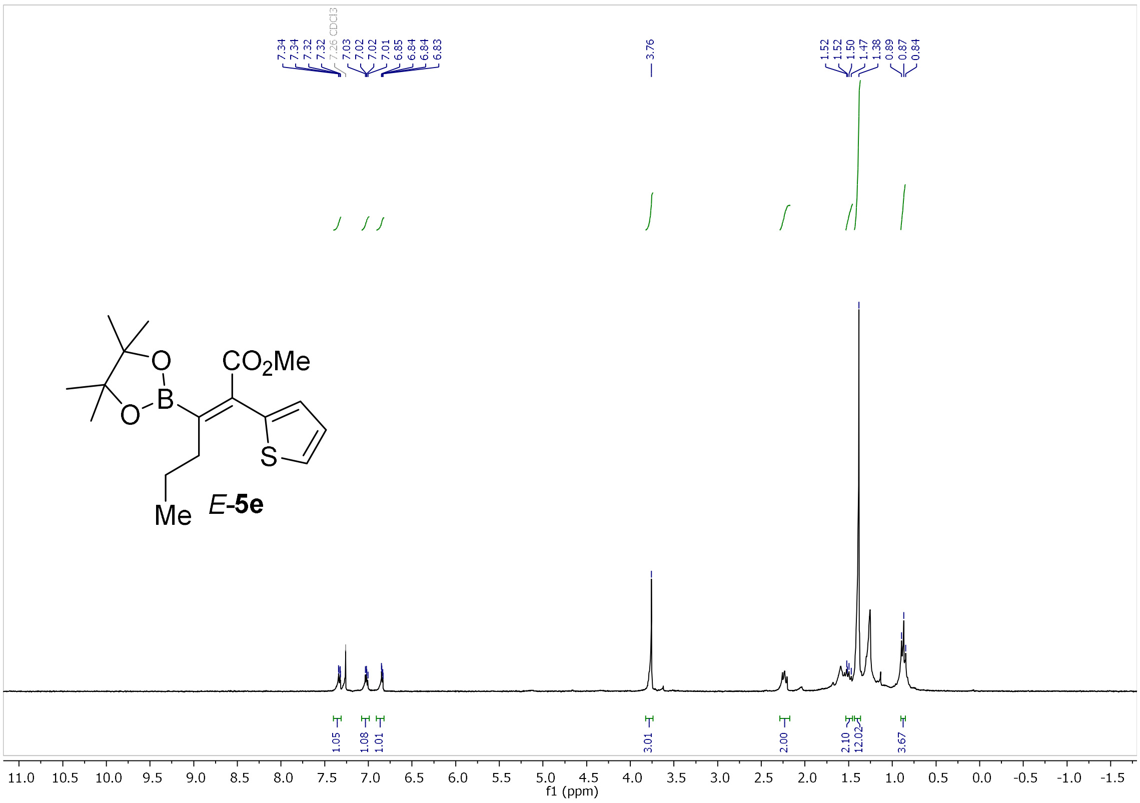

Figure S164. ^{1}H NMR spectrum of *E*-**5e.**

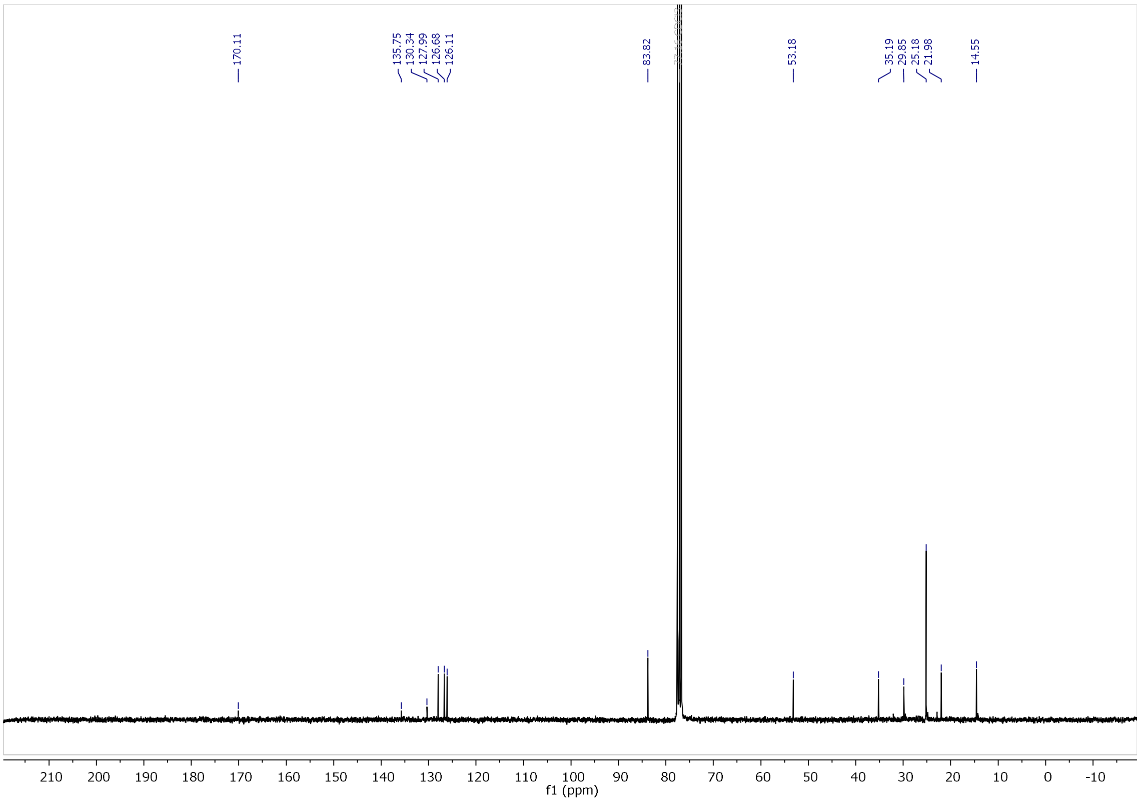

Figure S165. ^{13}C NMR spectrum of *E*-**5e.**

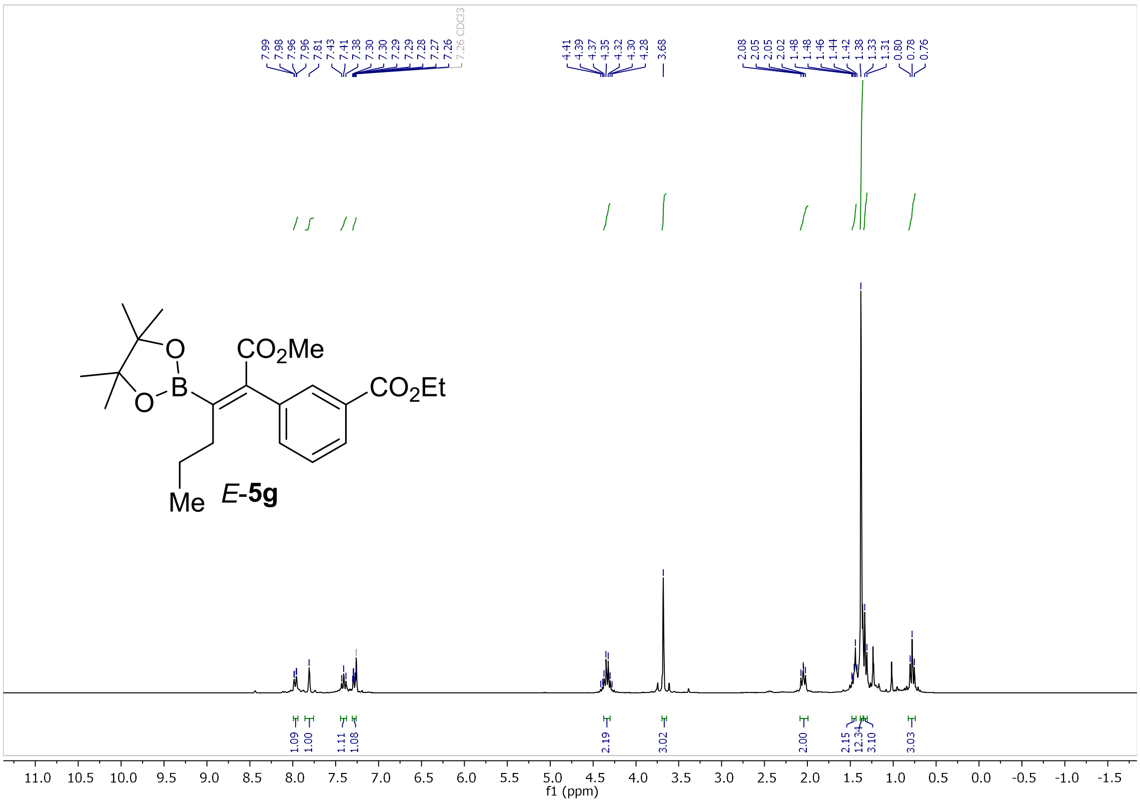

Figure S166. ^{1}H NMR spectrum of *E*-**5g.**

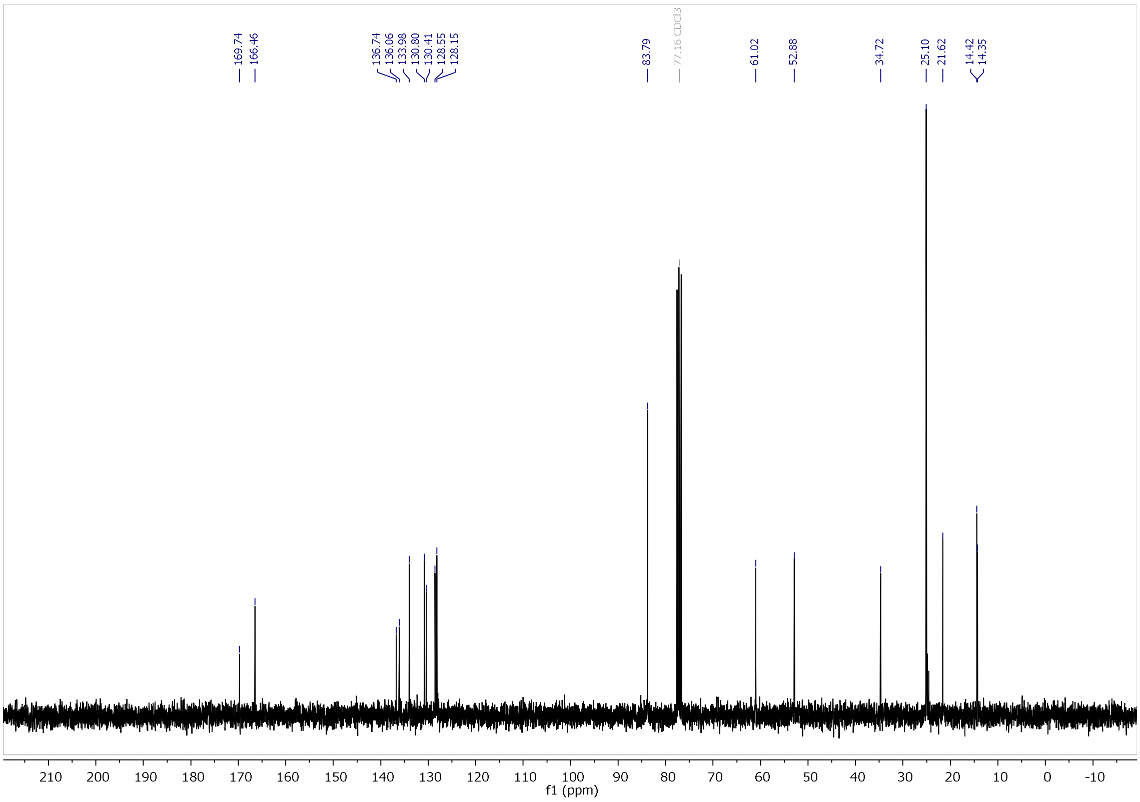

Figure S167. ^{13}C NMR spectrum of *E*-**5g.**

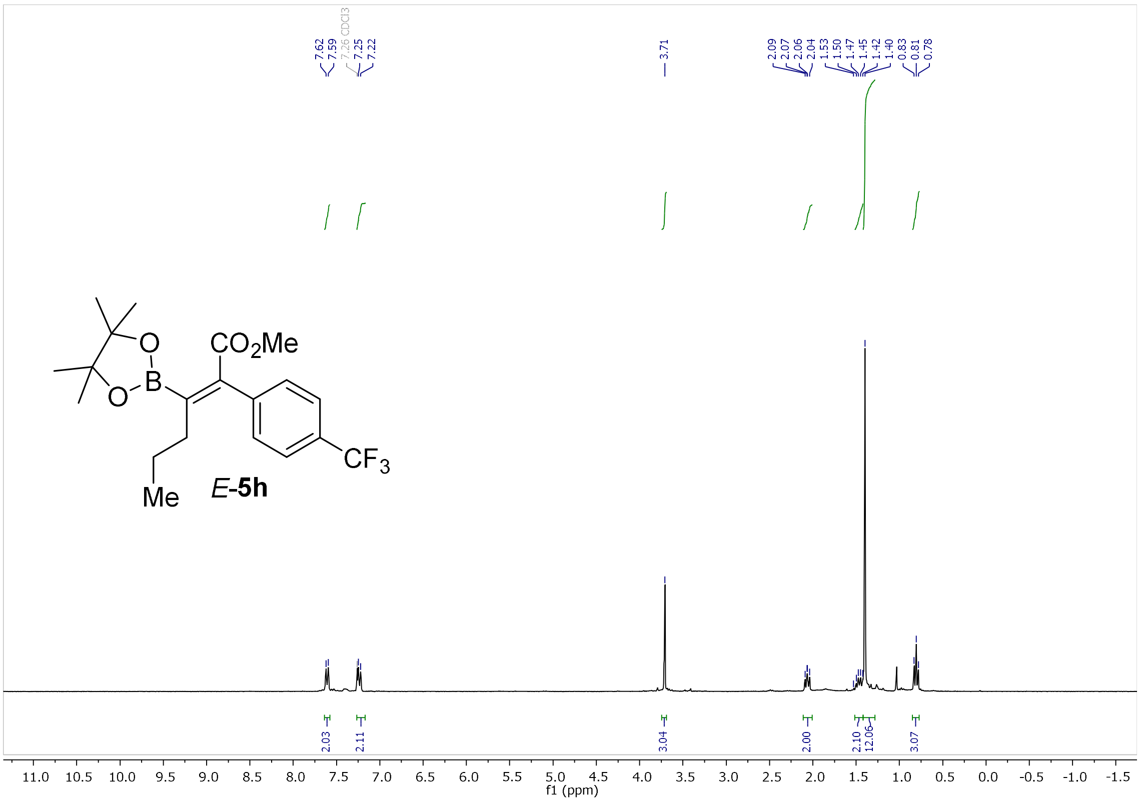

Figure S168. ^{1}H NMR spectrum of *E*-**5h.**

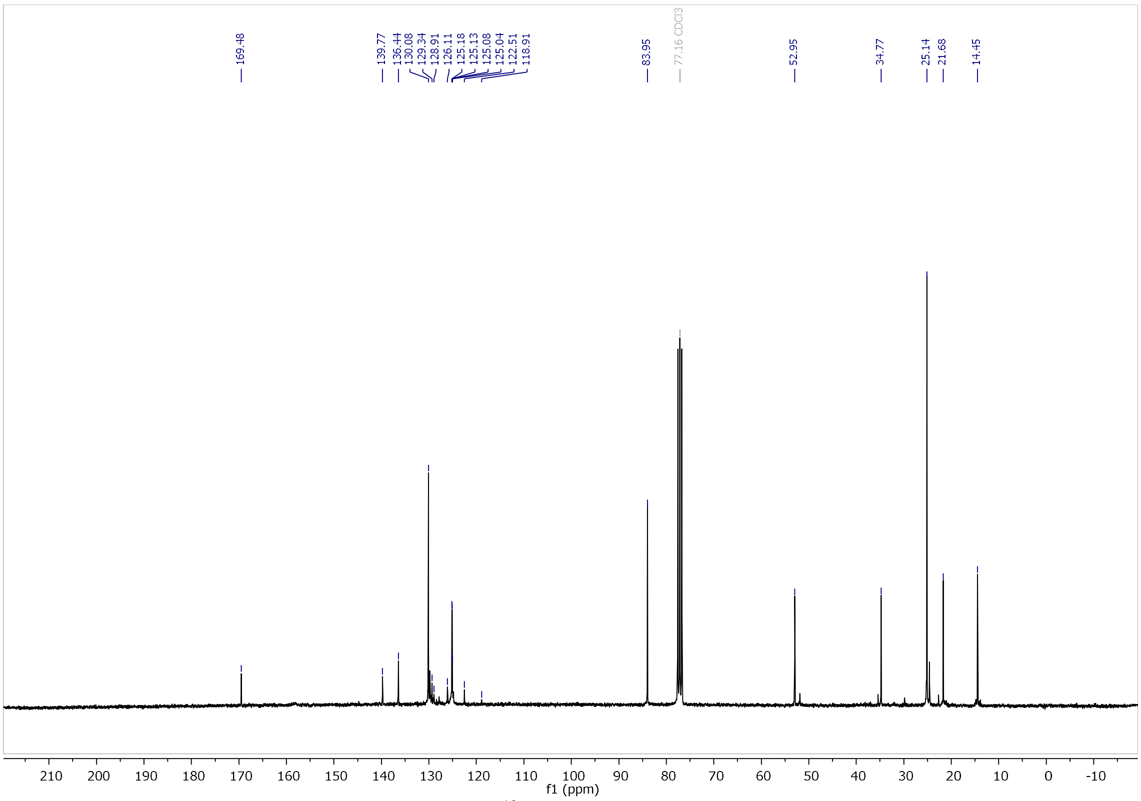

Figure S169. ^{13}C NMR spectrum of *E*-**5h.**

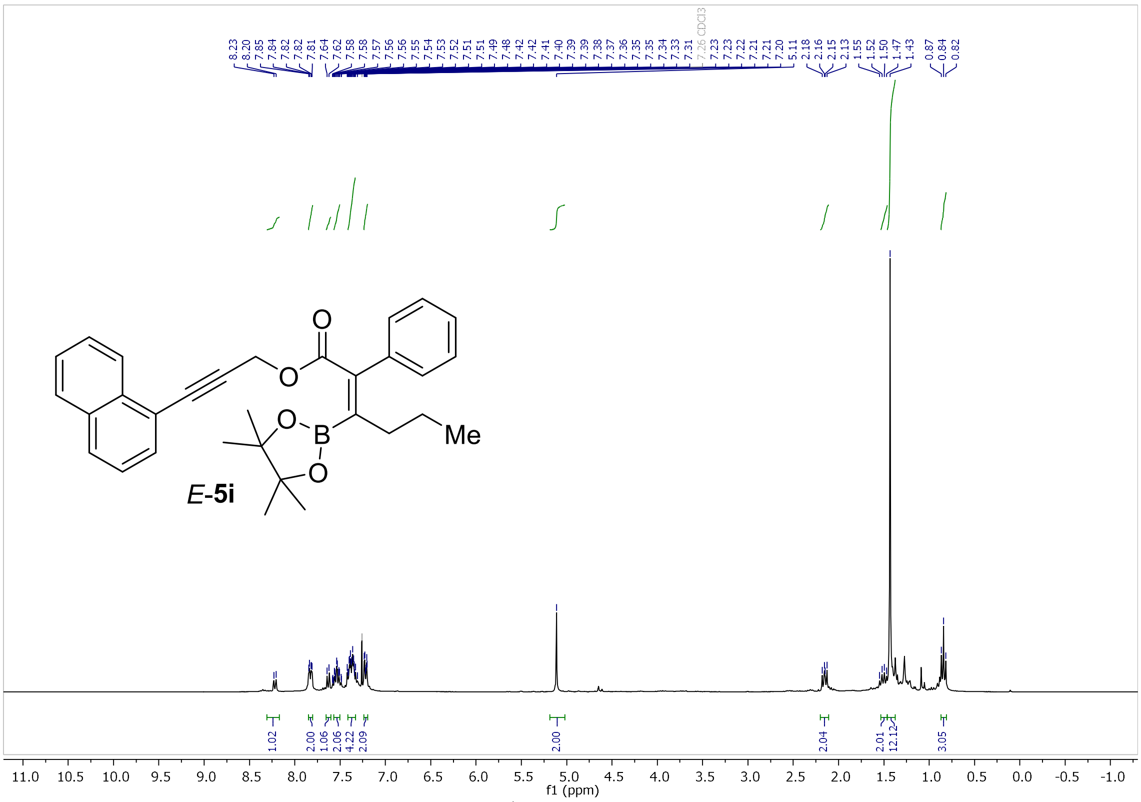

Figure S170. ^{1}H NMR spectrum of *E*-**5i.**

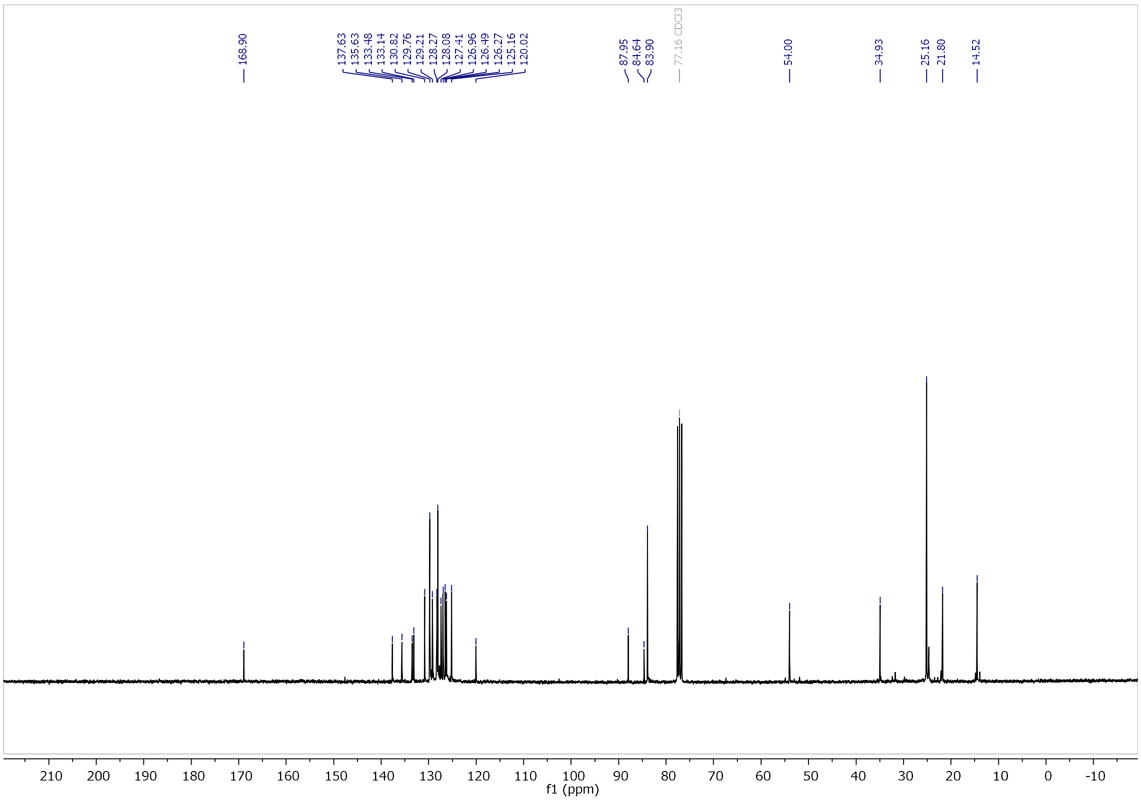

Figure S171. ^{13}C NMR spectrum of *E*-**5i.**

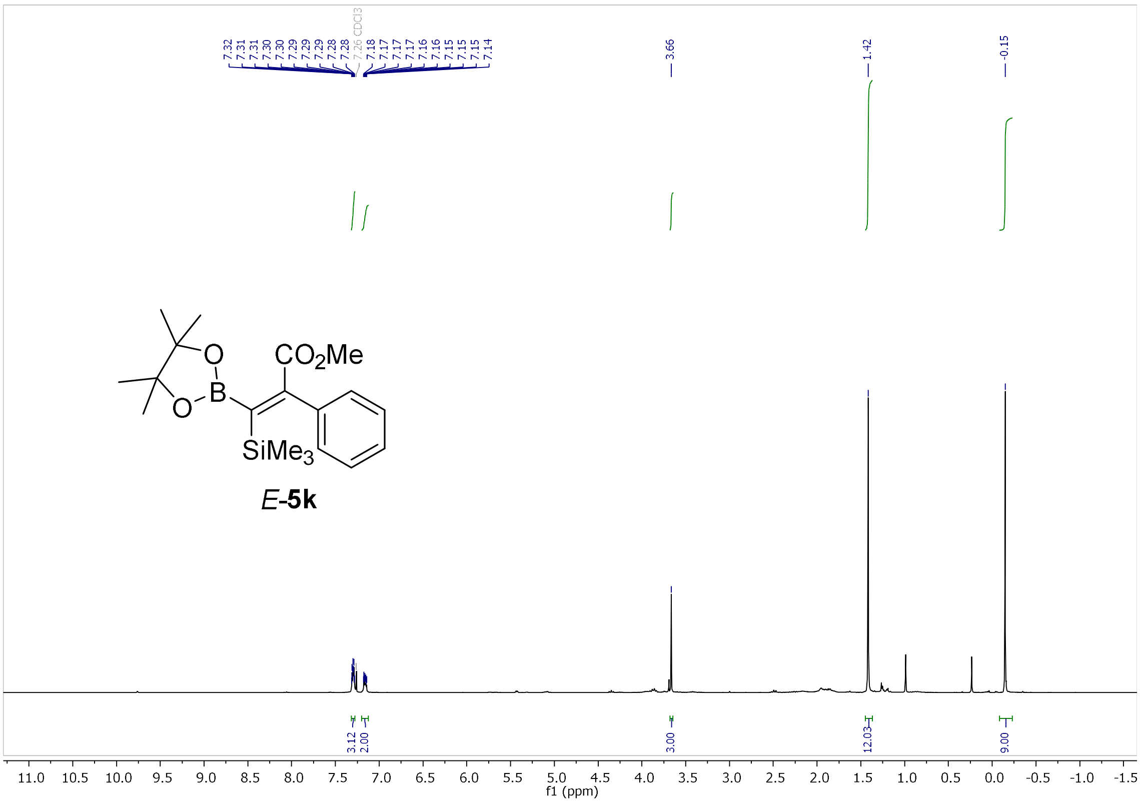

Figure S172. ^{1}H NMR spectrum of *E*-**5k.**

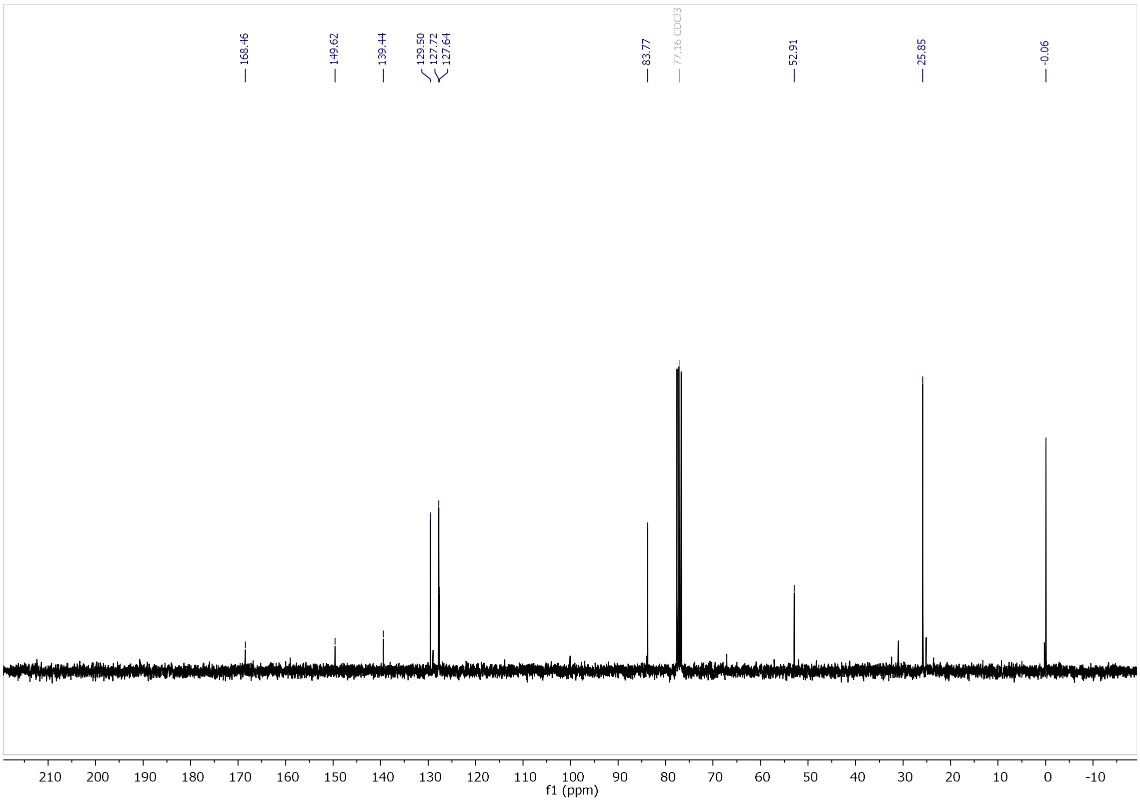

Figure S173. ^{13}C NMR spectrum of *E*-**5k.**

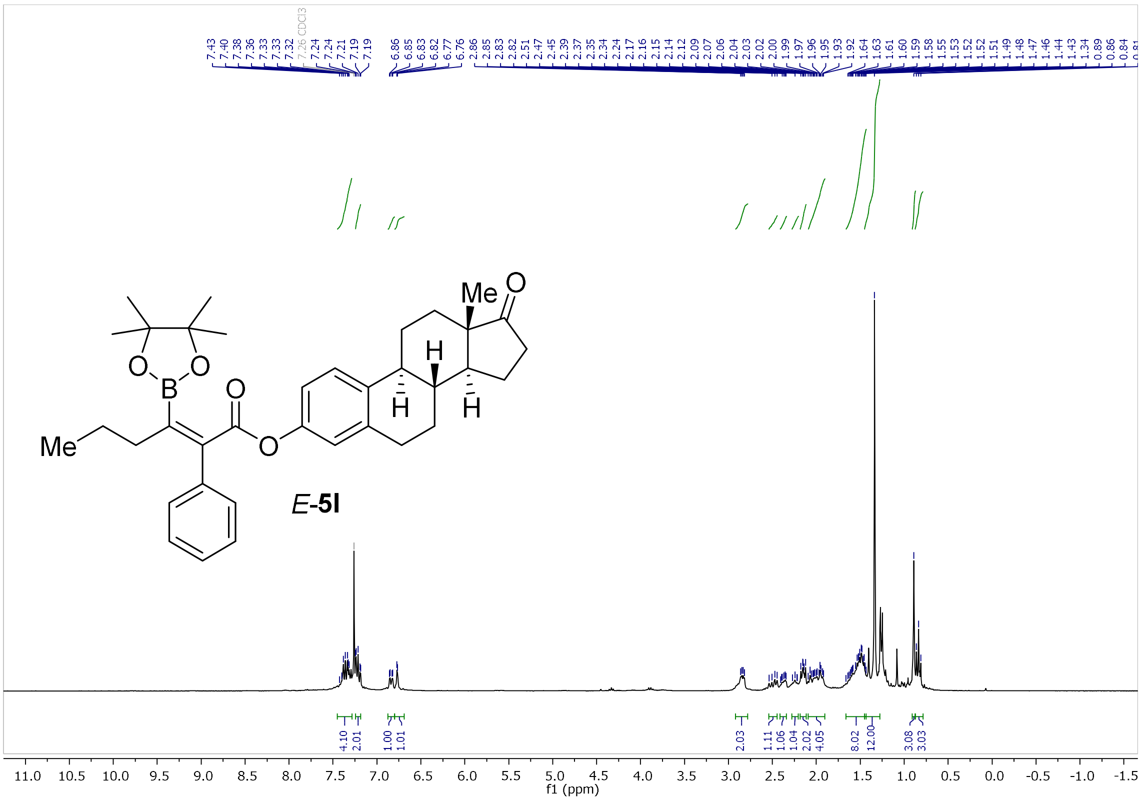

Figure S174. ^{1}H NMR spectrum of *E*-**5l.**

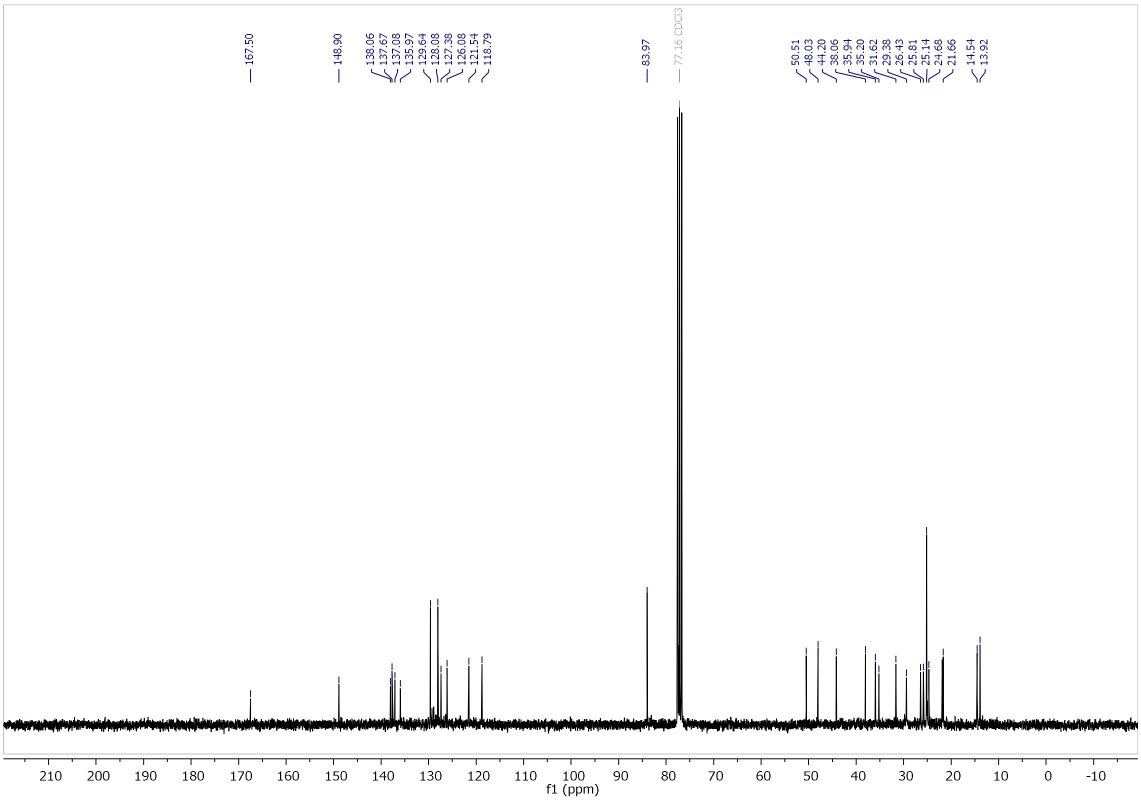

Figure S175. ^{13}C NMR spectrum of *E*-**5l.**

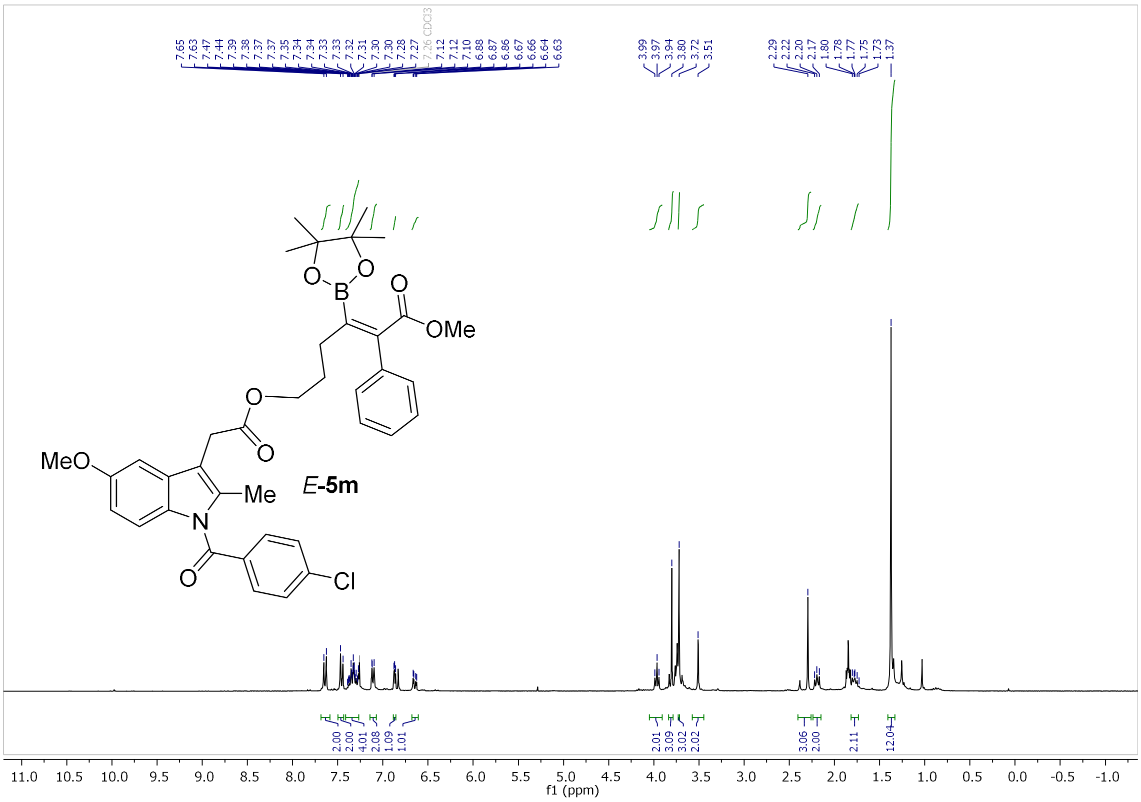

Figure S176. ¹H NMR spectrum of *E*-**5m.**

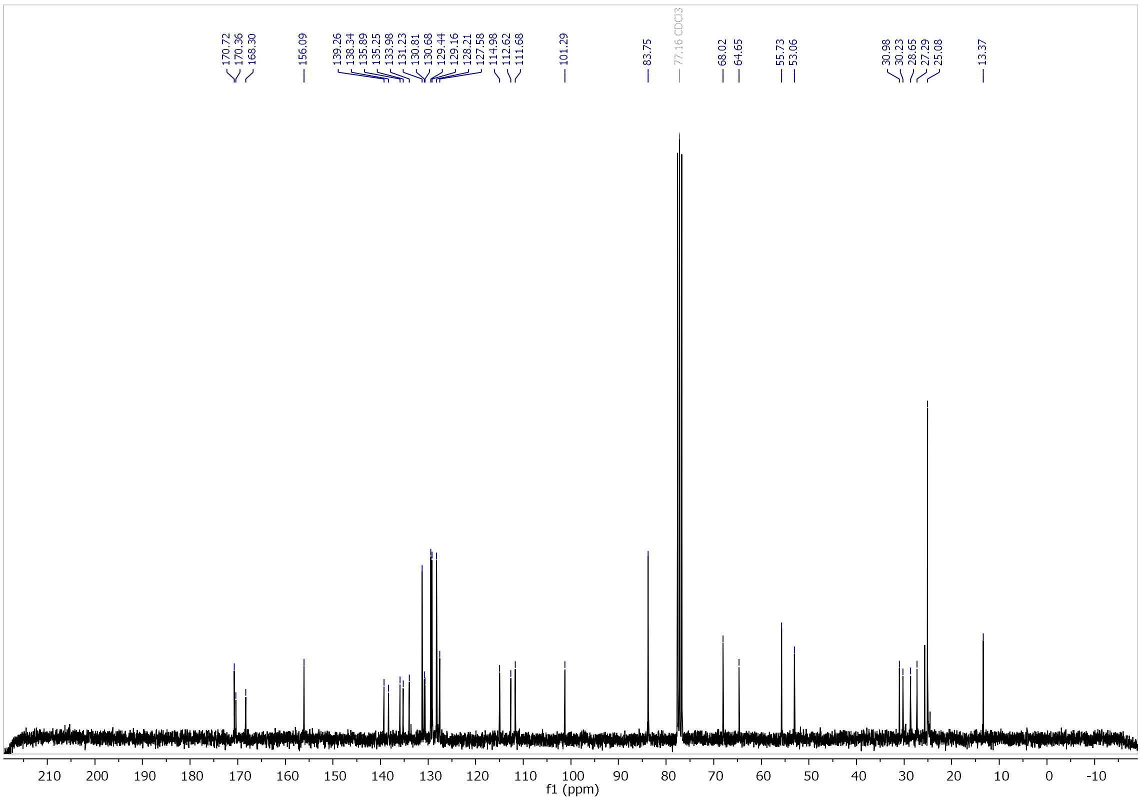

Figure S177. ¹³C NMR spectrum of *E*-**5m.**

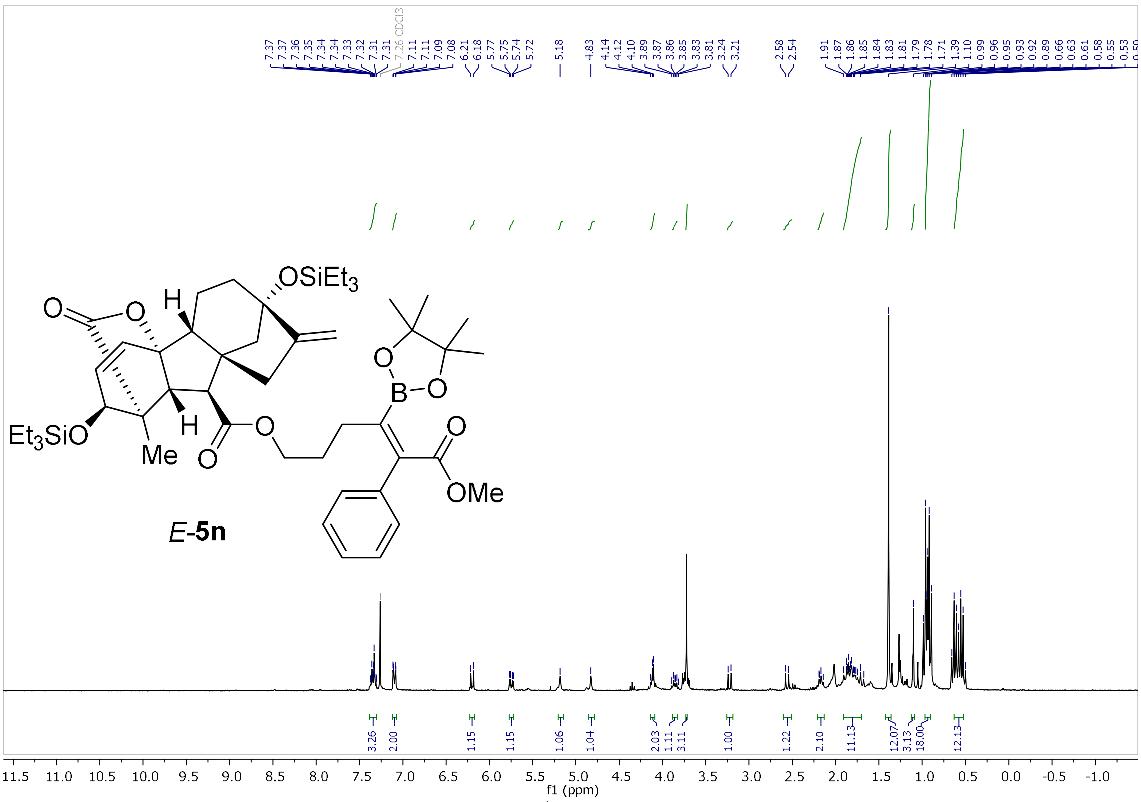

Figure S178. ^{1}H NMR spectrum of *E*-**5n.**

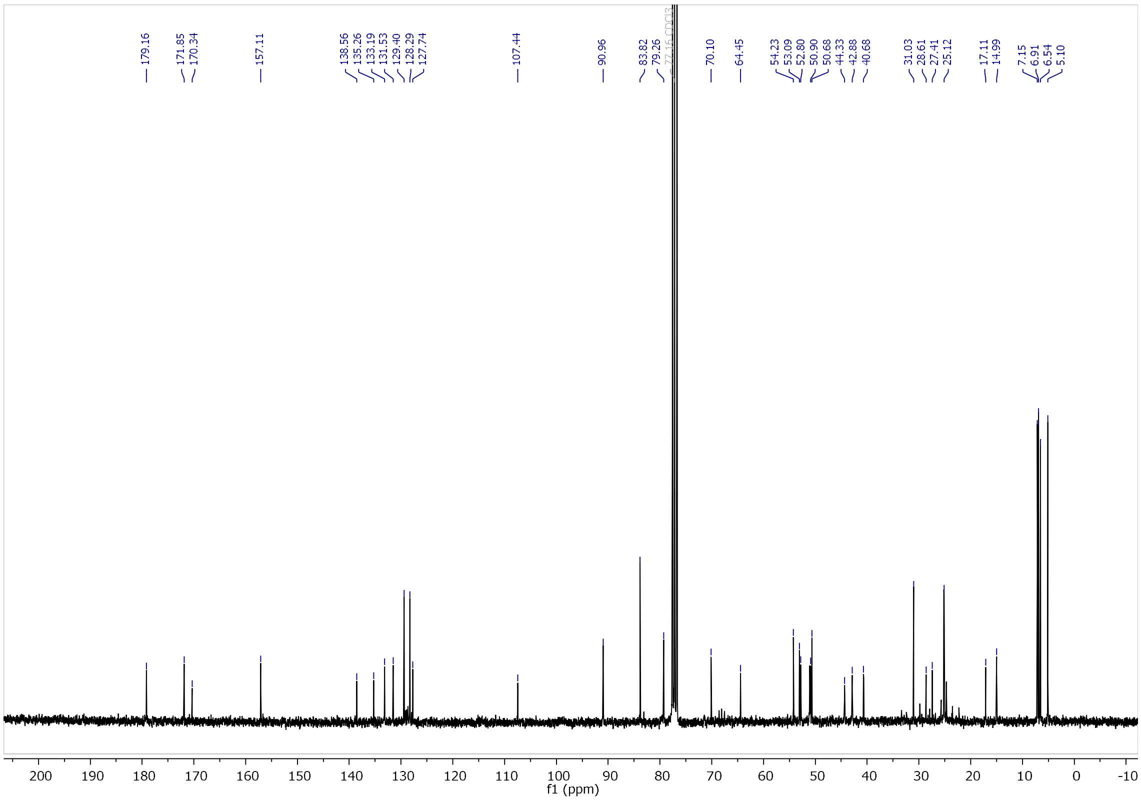

Figure S179. ^{13}C NMR spectrum of *E*-**5n.**

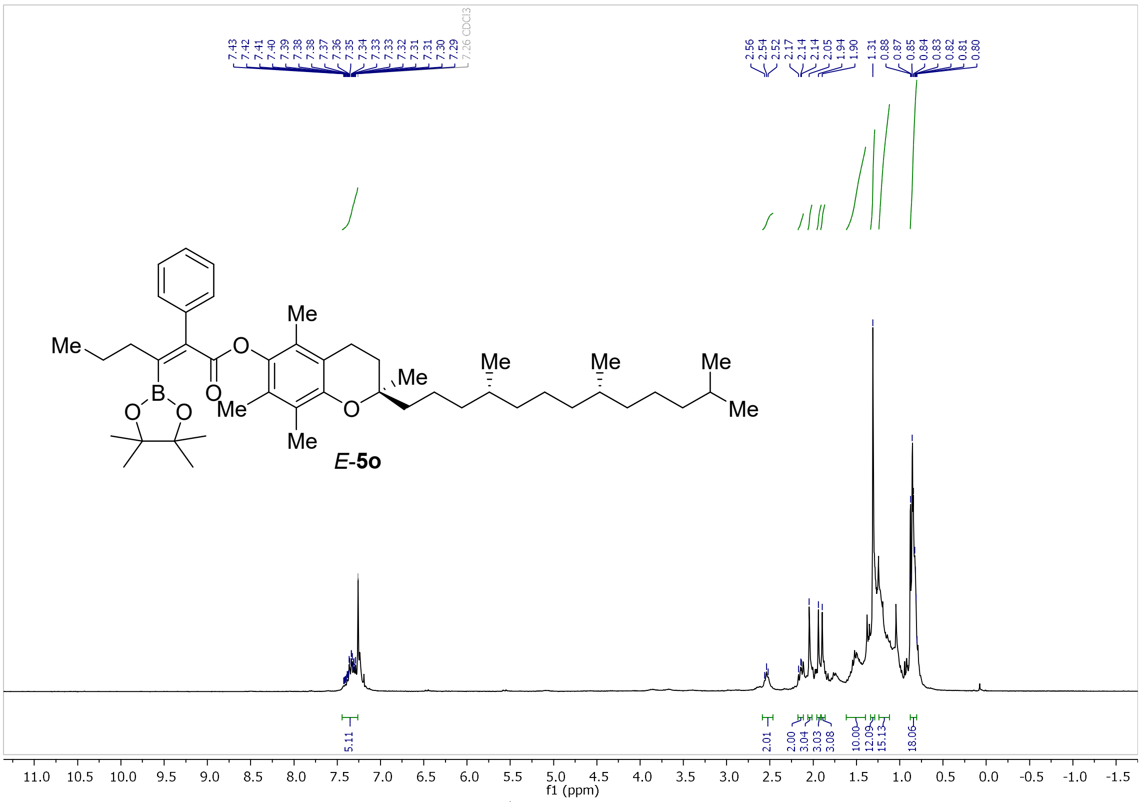

Figure S180. ^{1}H NMR spectrum of *E*-**5o.**

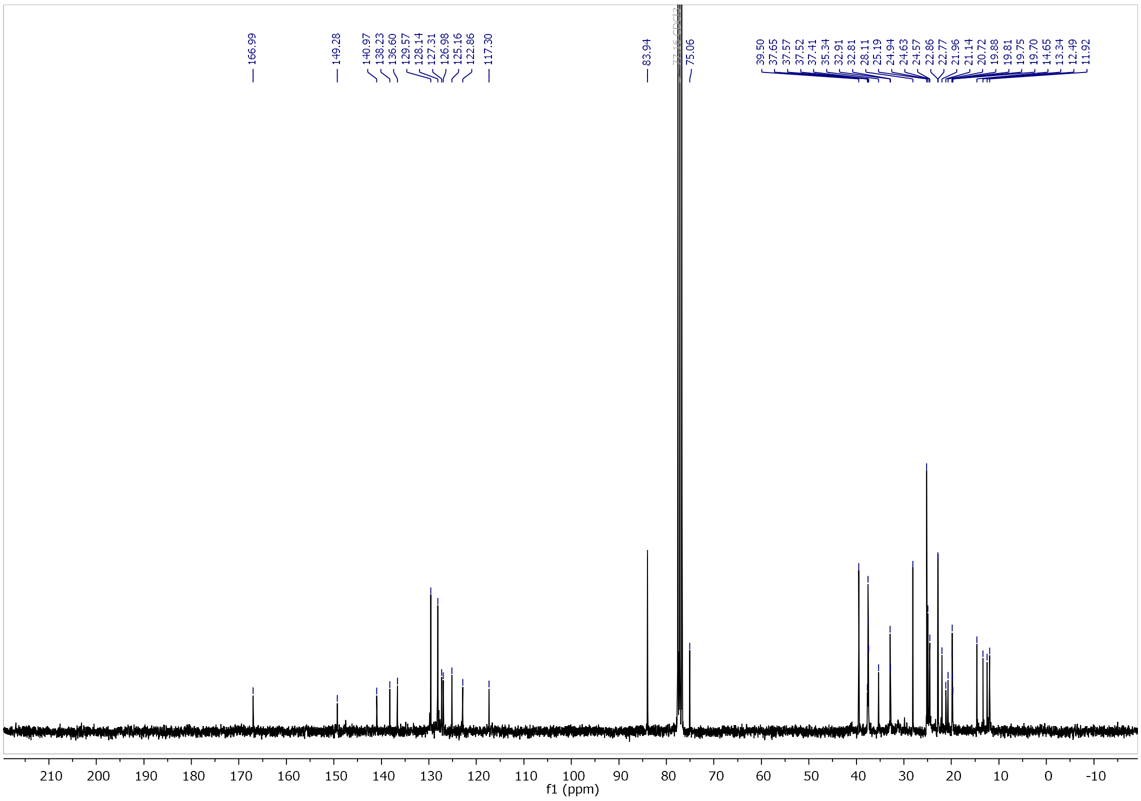

Figure S181. ^{13}C NMR spectrum of *E*-**5o.**
